# Phylogenomics and Comparative Genomic Studies Robustly Support Division of the Genus *Mycobacterium* into an Emended Genus *Mycobacterium* and Four Novel Genera

**DOI:** 10.3389/fmicb.2018.00067

**Published:** 2018-02-13

**Authors:** Radhey S. Gupta, Brian Lo, Jeen Son

**Affiliations:** Department of Biochemistry and Biomedical Sciences, McMaster University, Hamilton, CA, Canada

**Keywords:** *Mycobacterium* classification, slow-growing and fast-growing mycobacteria, conserved signature indels and signature proteins, phylogenomic analysis, fortuitum-vaccae clade, abscessus-chelonae clade, terrae clade, triviale clade

## Abstract

The genus *Mycobacterium* contains 188 species including several major human pathogens as well as numerous other environmental species. We report here comprehensive phylogenomics and comparative genomic analyses on 150 genomes of *Mycobacterium* species to understand their interrelationships. Phylogenetic trees were constructed for the 150 species based on 1941 core proteins for the genus *Mycobacterium*, 136 core proteins for the phylum Actinobacteria and 8 other conserved proteins. Additionally, the overall genome similarity amongst the *Mycobacterium* species was determined based on average amino acid identity of the conserved protein families. The results from these analyses consistently support the existence of five distinct monophyletic groups within the genus *Mycobacterium* at the highest level, which are designated as the “*Tuberculosis-Simiae*,” “*Terrae,”* “*Triviale*,” “*Fortuitum-Vaccae*,” and “*Abscessus-Chelonae*” clades. Some of these clades have also been observed in earlier phylogenetic studies. Of these clades, the “*Abscessus-Chelonae”* clade forms the deepest branching lineage and does not form a monophyletic grouping with the “*Fortuitum-Vaccae*” clade of fast-growing species. In parallel, our comparative analyses of proteins from mycobacterial genomes have identified 172 molecular signatures in the form of conserved signature indels and conserved signature proteins, which are uniquely shared by either all *Mycobacterium* species or by members of the five identified clades. The identified molecular signatures (or synapomorphies) provide strong independent evidence for the monophyly of the genus *Mycobacterium* and the five described clades and they provide reliable means for the demarcation of these clades and for their diagnostics. Based on the results of our comprehensive phylogenomic analyses and numerous identified molecular signatures, which consistently and strongly support the division of known mycobacterial species into the five described clades, we propose here division of the genus *Mycobacterium* into an emended genus *Mycobacterium* encompassing the “*Tuberculosis-Simiae*” clade, which includes all of the major human pathogens, and four novel genera viz. *Mycolicibacterium* gen. nov., *Mycolicibacter* gen. nov., *Mycolicibacillus* gen. nov. and *Mycobacteroides* gen. nov. corresponding to the “*Fortuitum-Vaccae,”* “*Terrae,”* “*Triviale*,” and “*Abscessus-Chelonae”* clades, respectively. With the division of mycobacterial species into these five distinct groups, attention can now be focused on unique genetic and molecular characteristics that differentiate members of these groups.

## Introduction

The genus *Mycobacterium* encompasses a large group of Gram-positive, rod-shaped, acid-fast organisms in the phylum Actinobacteria (Hartmans et al., 2006; Gao and Gupta, 2012; Magee and Ward, 2012). Many members are well-known human pathogens, most notably *Mycobacterium tuberculosis* and *Mycobacterium leprae* are causative agents of tuberculosis and leprosy, respectively (Medjahed et al., 2010; Magee and Ward, 2012; Lory, 2014). In addition, *Mycobacterium* species are found to inhabit a diverse range of environments including water bodies, soil, and metalworking fluids (Hartmans et al., 2006; Brzostek et al., 2009; Falkinham, 2009; Tortoli, 2012). At the time of writing, the genus *Mycobacterium* consists of 188 species with validly published names (www.namesforlife.com) (Parte, 2014). In view of the large numbers of both clinically important as well as environmental species present in a single genus, an understanding of the relationships between these organisms is of much importance (Gao and Gupta, 2012; Magee and Ward, 2012; Tortoli, 2012; Lory, 2014; Fedrizzi et al., 2017). Current understanding of the relationships within the genus *Mycobacterium* is primarily based on analysis of the 16S rRNA gene sequences and other physical and chemotaxonomic characteristics of the species (Runyon, 1965; Rogall et al., 1990; Stahl and Urbance, 1990; Goodfellow and Magee, 1998; Hartmans et al., 2006; Magee and Ward, 2012). Besides the 16S rRNA, the relationships among the mycobacterial species has also been examined using the 16S-23S spacer sequences (Roth et al., 1998) and several housekeeping genes including *hsp65* (Kim et al., 2005; Tortoli et al., 2015), *gyrB* (Kasai et al., 2000), *rpoB* (Tortoli, 2012), and *gyrA* (Guillemin et al., 1995). A number of studies have also been performed on a limited number of mycobacterial species using multilocus sequence analysis based on concatenated sequences of nucleotides or amino acid fragments from several gene sequences viz. 16S rRNA, *rpoB*, and *hsp65* (Kim and Shin, 2017); 16S rRNA, *hsp65, sodA, recA, rpoB* (Adékambi and Drancourt, 2004) and *hsp65, tuf*, *rpoB, smpB*, 16S rRNA, *sodA*, tmRNA (Mignard and Flandrois, 2008). The results of these studies have provided useful insights into the relationships between members of the genus *Mycobacterium*.

An important difference observed among the mycobacterial species very early was the differences in their growth rates (Tsukamura, 1967a; Wayne and Kubica, 1986; Magee and Ward, 2012). Based on their rates of growth, *Mycobacterium* species, in general, can be roughly divided into two groups; one group consists of slow-growing bacteria (i.e., requiring *more* than 7 days to form colonies), while the second group is comprised of rapid-growing bacteria which require <7 days to form colonies (Tsukamura, 1967a; Wayne and Kubica, 1986; Magee and Ward, 2012; Lory, 2014). The clades encompassing most of the slow-growing mycobacteria also branches distinctly from the fast-growing species in the 16S rRNA trees (Rogall et al., 1990; Stahl and Urbance, 1990; Goodfellow and Magee, 1998), and also in phylogenetic trees based on some other genes/proteins sequences (Adékambi and Drancourt, 2004; Kim et al., 2005; Adékambi et al., 2006a; Mignard and Flandrois, 2008; Tortoli, 2012; Tortoli et al., 2015). Although a broad separation of the slow-growing mycobacteria from the rapid-growing species is generally supported, the reliability of the methods used to discern these two groups, particularly the cohesiveness of the rapid-growing mycobacteria, remains of concern (Magee and Ward, 2012; Tortoli, 2012). Recent studies have also identified some distinct groupings within the slow- or rapid-growing mycobacteria. For example, a clade consisting of *Mycobacterium terrae* and its closely related members, which exhibits slow to intermediate rate of growth, can be differentiated from other slow-growing members by a characteristic 14 nt insert in the helix 18 of 16S rRNA gene and by means of phylogenetic analysis (Mignard and Flandrois, 2008; Kim et al., 2012; Tortoli, 2012; Tortoli et al., 2013; Ngeow et al., 2015; Vasireddy et al., 2016). Another clade of mycobacterial species closely related to *Mycobacterium abscessus*, can also be differentiated from other rapid-growing members based on phylogenetic branching and unique pathogenicity profile of its members (Adékambi and Drancourt, 2004; Medjahed et al., 2010; Tortoli, 2012; Wee et al., 2017). In light of the increased awareness of the diversity that exists within the mycobacterial species as well the clinical importance of many of the members from this genus, the need for more robust methods of delineation of different groups that exists within this important group of bacteria is warranted (Fedrizzi et al., 2017).

Due to rapid advances in genome sequencing technology, genome sequences for 150 members from the genus *Mycobacterium* are now publicly available in the NCBI genome database (https://www.ncbi.nlm.nih.gov/genome/). The analysis of whole genome sequences allows for construction of more robust phylogenetic trees providing greater resolution in identifying the relationships at various taxonomic levels (Wu et al., 2009; Segata et al., 2013; Gupta et al., 2015; Adeolu et al., 2016). A number of recent studies have reported phylogenomic analyses based on large datasets of core genes/proteins from the genomes of 28–47 *Mycobacterium* species in order to elucidate their relationships (Prasanna and Mehra, 2013; Wang et al., 2015; Fedrizzi et al., 2017; Wee et al., 2017). Based on genome sequences, the genomic relatedness among the organisms can also be determined and this approach is now widely applied in taxonomic studies (Konstantinidis and Tiedje, 2005; Thompson et al., 2013; Qin et al., 2014). In addition, the genome sequences provide a unique resource for comparative genomic studies in identifying molecular markers or signatures that are specifically shared by an evolutionarily related group of organisms and are useful in the demarcation of different taxa and for understanding interrelationships (Gao and Gupta, 2012; Gupta, 2014, 2016a; Adeolu et al., 2016). Of the two types of molecular markers that have proven particularly useful for evolutionary/taxonomic studies, conserved signature indels (CSIs) are amino acid insertions or deletions of fixed lengths that are present at a specific position within a conserved region in an evolutionarily related group of species (Gupta, 2014, 2016b; Naushad et al., 2014). Likewise, conserved signature proteins (CSPs) are proteins, whose homologs are exclusively found in a related-group of organisms (Gao et al., 2006; Gao and Gupta, 2012; Gupta et al., 2015; Gupta, 2016b). The presence of these clade-specific marker gene sequences (or synapomorphies) is most parsimoniously accounted by their initial introduction in a common ancestor of the group followed by vertical inheritance (Gupta, 1998, 2016b; Gao and Gupta, 2012; Naushad et al., 2014).

To reliably understand the relationships within the genus *Mycobacterium*, we have carried out comprehensive phylogenomic and comparative genomic studies on 150 mycobacterial species, whose genome sequences are now available. Based on genome sequences, robust phylogenetic trees have been constructed based on different large datasets of concatenated protein sequences including two trees based on 1941 and 136 core proteins for the genus *Mycobacterium* and the phylum Actinobacteria, respectively. Based on genome sequences, the pairwise average amino acid identity (AAI) was also determined for the mycobacterial species. Lastly, our detailed comparative genomic studies on mycobacterial genomes have identified 172 highly specific molecular markers in the forms of CSIs and CSPs, which are either uniquely shared by all members of the genus *Mycobacterium* or for a number of distinct clades within this genus at multiple phylogenetic levels. Based on the results of these comprehensive analyses, it is now possible to reliably divide the species from the genus *Mycobacterium* into five main monophyletic clades, which are referred to here as the “*Tuberculosis-Simiae*” clade, the “*Terrae”* clade, the “*Triviale*” clade, the “*Fortuitum-Vaccae*” clade, and the “*Abscessus-Chelonae*” clade. Based on the large body of evidence presented here which consistently and strongly supports the existence of these five clades, a proposal is made here to divide the genus *Mycobacterium* into an emended genus *Mycobacterium* encompassing the members of the “*Tuberculosis-Simiae*” clade and four new genera *Mycolicibacter* gen. nov. (“*Terrae”* clade), *Mycolicibacillus* gen. nov. (“*Triviale”* clade), *Mycolicibacterium* gen. nov. (“*Fortuitum-Vaccae”* clade), and *Mycobacteroides* gen. nov. (“*Abscessus-Chelonae”* clade).

## Methods

### Phylogenetic and genomic analyses of the genus *Mycobacterium*

Phylogenetic trees were constructed for 150 members of the genus *Mycobacterium* whose genomes are now sequenced (some characteristics of these genomes are listed in Supplementary Table 1) and six members from the order *Corynebacteriales* (viz. *Corynebacterium diphtheriae* NCTC 11397, *Gordonia bronchialis* DSM 43247, *Nocardia farcinica* NCTC 11134, *Rhodococcus erythropolis* PR4, *Segniliparus rotundus* DSM 44985 and *Tsukamurella paurometabola* DSM 20162), which served as outgroups. The first of these trees was based on 1941 core proteins from the genomes of *Mycobacterium* species and its construction was carried out by using a software pipeline (Adeolu et al., 2016). Briefly, the CD-HIT program was used (Li and Godzik, 2006; Fu et al., 2012) to identify protein families sharing a minimum of 50% in sequence identity and sequence length and which were found in at least 80% of the input genomes. The Clustal Omega (Sievers et al., 2011) algorithm was used to generate multiple sequence alignment (MSA) of these protein families. The aligned protein families were trimmed with TrimAl (Capella-Gutierrez et al., 2009) to remove poorly aligned regions (Talavera and Castresana, 2007) before concatenation to the other core proteins. The concatenated sequence alignment of 1941 core proteins consisted of 624,360 aligned amino acids. Another comprehensive phylogenetic tree was constructed based on concatenated sequences for 136 proteins, which comprise the phyloeco markers set for the phylum Actinobacteria (Wang and Wu, 2013). Information regarding these proteins is provided in Supplementary Table 2. The profile Hidden Markov Models of these protein families were used for the identification of members of these protein families in the input genomes using HMMer 3.1 (Eddy, 2011). The sequence alignments were trimmed using TrimAl (Capella-Gutierrez et al., 2009) before their concatenation into a single file. The combined sequence from the phyloeco set of proteins consisted of a total of 44,976 aligned amino acids. Maximum likelihood (ML) trees based on both these sequence alignments were constructed using the Whelan and Goldman model of protein sequence evolution (Whelan and Goldman, 2001) in FastTree 2 (Price et al., 2010) and the Le and Gascuel model of protein sequence evolution (Le and Gascuel, 2008) in RAxML 8 (Stamatakis, 2014). Optimization of the robustness of the tree was completed by conducting SH tests (Guindon et al., 2010) in RAxML 8 (Stamatakis, 2014). The identification of the conserved protein families and the construction of phylogenetic trees were completed using an internal software pipeline (Adeolu et al., 2016).

In addition to these two comprehensive trees, another phylogenetic tree was constructed based on concatenated sequences for 8 conserved housekeeping proteins (viz. RpoA, RpoB, RpoC, GyrA, GyrB, Hsp65, EF-Tu and RecA). After removal of non-conserved regions, the concatenated sequence alignment in this case consisted of 6052 aligned amino acids. A maximum likelihood phylogenetic tree based on this sequence was constructed as described above.

The sequence alignments of the 1941 core proteins identified by the above methods were also used to measure genome relatedness. Using the amino acid sequences from these conserved protein families, the amino acid sequence identity between each pair of *Mycobacterium* genomes was calculated (Thompson et al., 2013).

Information regarding branching of all type species from the genus *Mycobacterium* in a tree based on 16S rRNA sequences was obtained from the SILVA All Species Tree of Life Project 128 (Quast et al., 2013).

### Identification of conserved signature indels (CSIs)

The identification of CSIs was carried out as described in earlier work (Gao and Gupta, 2005; Bhandari et al., 2012; Gupta, 2014; Naushad et al., 2014; Sawana et al., 2014). All annotated proteins from the genomes of *M. tuberculosis* H37Rv and *M. sinense* JDM601 were used in these analyses. BLASTp (Altschul et al., 1997) searches were conducted on all protein sequences >100 amino acids in length against the NCBI non-redundant (nr) database. Multiple sequence alignments were generated by obtaining 15–25 homologs from diverse *Mycobacterium* species and 8–10 homologs from other groups of bacteria. The alignments were visually inspected for sequence gaps of fixed lengths which were flanked on both sides by at least 5 conserved amino acids in the neighboring 30–40 amino acids, and appeared to be shared by either some or all mycobacterial homologs. Query sequences encompassing the potential indel and flanking regions (60–100 amino acids long) were collected and subjected to a more detailed BLASTp search (500 or more hits) to determine the group specificities of the observed indels. Signature files for all CSIs of interest were created using SIG_CREATE and SIG_STYLE programs in the GLEANS software package (available on Gleans.net). Unless otherwise noted, the described CSIs are specific for the indicated groups of species.

### Identification of conserved signature proteins (CSPs)

The identification of conserved signature proteins was carried out using the protocol described in earlier work (Gao et al., 2006; Adeolu and Gupta, 2014; Naushad et al., 2014). BLASTp (Altschul et al., 1997) searches were conducted on all sequenced proteins from the genomes of *M. tuberculosis* H37Rv, *M. aurum* (LSHTM), *M. sinense* JDM601 (Zhang et al., 2011), *M. triviale* DSM 44153 (Fedrizzi et al., 2017), and *M. abscessus* ATCC 19977 (Ripoll et al., 2009) against the NCBI nr database. Proteins of interest were those where either all significant hits were limited to the genus *Mycobacterium* or the indicated groups/clades of mycobacteria, or where a large increase in E value was observed from the last hit belonging to these groups and the first hit from any other bacteria, and the *E*-values for the latter hits were >1e^−3^ (Gao et al., 2006; Gao and Gupta, 2012; Naushad et al., 2014). However, in some cases, a few proteins where an isolated significant hit from an unrelated group of bacteria was observed were also retained as CSPs specific for the group of interest.

## Results

### Phylogenomic analysis of the genus *Mycobacterium*

In the present work, two comprehensive phylogenomic trees were constructed based on the genome sequences of 150 *Mycobacterium* species. The first of these trees was a core genome tree of 1941 proteins, whose homologs are present in at least 80% of the input mycobacterial genomes as well as the outgroup species. The second genome sequence tree was based on 136 proteins, which are part of the phyloeco set for the phylum Actinobacteria. The trimmed concatenated sequence alignments for the two sets of core proteins, which were employed for phylogenetic analyses, consisted of 624,360 and 44,976 aligned amino acids, respectively. Although phylogenetic trees based on core genes/proteins for mycobacterial species have also been constructed in earlier studies (Prasanna and Mehra, 2013; Fedrizzi et al., 2017; Wee et al., 2017), they were based only on a small number (between 28 and 47) of *Mycobacterium* species. In contrast, the trees produced in this work include information for ~80% (150 of the 188) of all known mycobacterial species and thus constitute the most comprehensive phylogenetic trees constructed for the genus *Mycobacterium*. In addition to the two core genome protein trees, a maximum-likelihood tree was also constructed based on concatenated sequences of 8 conserved housekeeping proteins.

**Figure 1:**
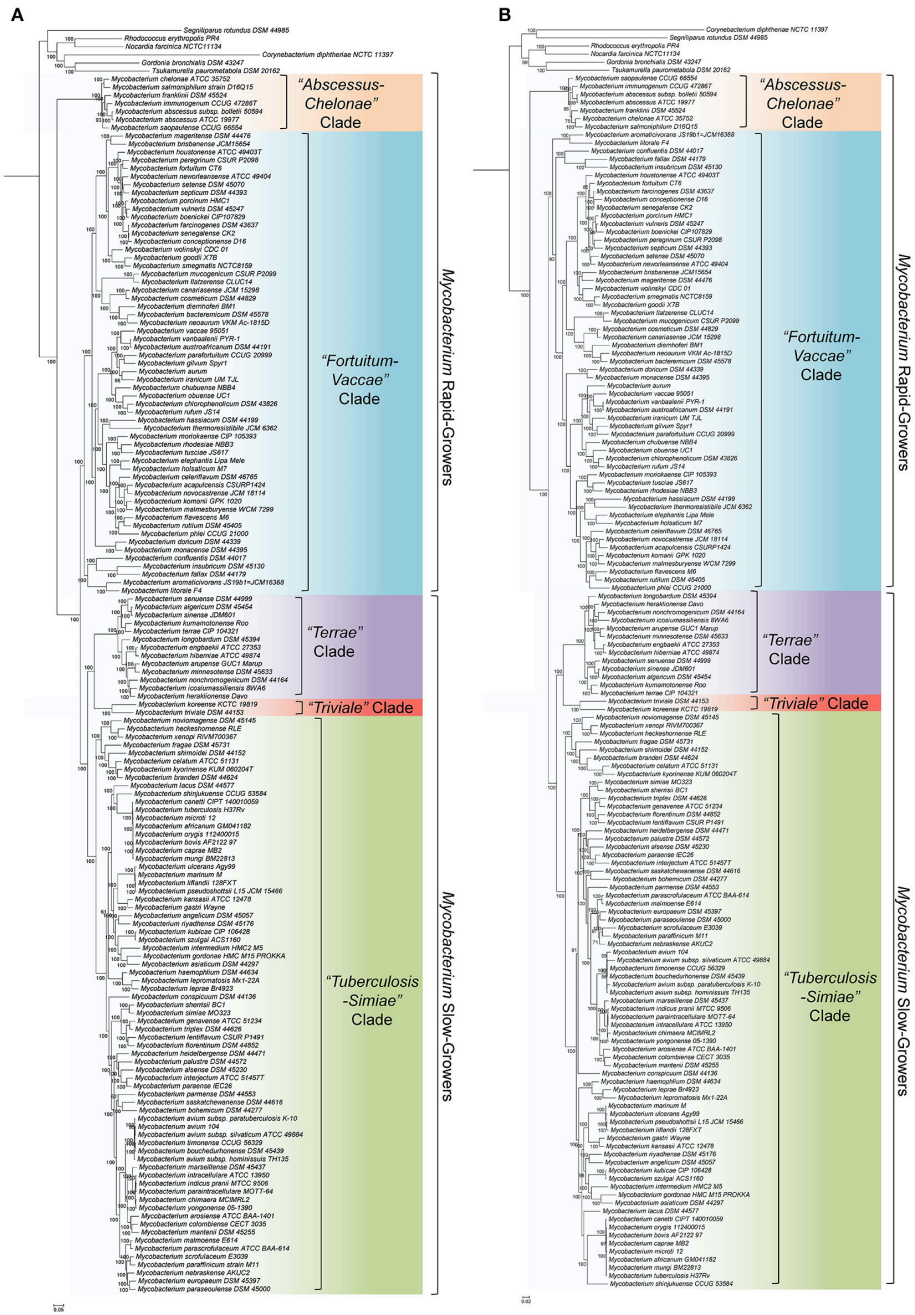
**(A)** Maximum-likelihood phylogenetic tree for 150 *Mycobacterium* species based on the concatenated sequence of 1941 core proteins from the genus *Mycobacterium*. **(B)** A maximum-likelihood phylogenetic tree based on the 136 proteins consistuting the phyloeco set for the phylum Actinobacteria. Both of these trees were rooted using the sequences from the *Corynebacteriales* species. Trees were constructed as described in the Methods section. SH-like statistical support values and the bootstrap value are marked on the nodes. The major clades as well as the clusters of slow-growing and fast-growing *Mycobacterium* species are labeled. Some slow-growing species, which branched within the rapid-growing species are marked with*.

The ML trees based on the core proteins from mycobacterial genomes and for the phylum Actinobacteria are shown in Figures 1A,B, respectively. The tree based on the 8 conserved proteins is provided as Supplementary Figure 1. In all of these phylogenetic trees, which were rooted using the sequences from the *Corynebacteriales* species, nearly all of the observed nodes were supported with high (100%) bootstrap scores or SH-values. Further, the majority of the interrelationships among the *Mycobacterium* species were highly similar and consistent in all constructed trees. In all of these trees, members of the genus *Mycobacterium* consistently grouped into four main clades and a clade consisting of the *M. triviale—M. koreense*, as indicated in Figure 1. Three of these clades are comprised of the slow-growing species, whereas the other two clades are mostly made up of the fast-growing species. Of the two clades of fast-growing species, the first clade referred to as the “*Abscessus-Chelonae”* clade, forms the earliest branching lineage within the genus *Mycobacterium*. The second clade of the fast-growing species designated as the “*Fortuitum-Vaccae*” clade, encompasses most of the other fast-growing species including those related to *M. fortuitum, M. vaccae, M. parafortuitum*, and *M. mucogenicum* (Hartmans et al., 2006; Magee and Ward, 2012; Lory, 2014). Of the three clades of slow-growing mycobacteria, the clade designated as “*Tuberculosis-Simiae*,” encompasses most of the clinically important *Mycobacterium* species including those related to *M. tuberculosis, M. avium, M. gordonae, M, kansasii* and *M. simiae* (Magee and Ward, 2012). The two other clades of the slow-growing species, often referred to as part of the “*M. terrae* complex,” group together and they form a sister clade to the “*Tuberculosis-Simiae*” clade. Of the two clades which form the “*M. terrae* complex,” most of the species closely related to *M. terrae* are part of a clade that is designated here as the “*Terrae”* clade (Magee and Ward, 2012; Tortoli, 2012; Ngeow et al., 2015). Adjacent to the “*Terrae”* clade, the species *M. koreense* and *M. triviale* form a distinct clade (designated here as the “*Triviale*” clade), which is separated from members of the “*Terrae”* clade by a long branch. It is important to note that in the phylogenetic trees shown in Figure 1, the two clades of fast-growing species do not form a monophyletic grouping, whereas the clades corresponding to the slow-growing mycobacteria group together and form a monophyletic lineage.

We have also compared the relationships observed in the aforementioned phylogenetic trees with the relationships observed in a tree based on 16S rRNA gene sequences, which was extracted from the SILVA Tree of Life Project 128 (Yarza et al., 2008; Quast et al., 2013). This tree is shown in Supplementary Figure 2 with the analogous groups labeled. Overall, in concordance with the core protein-based phylogenetic trees and the tree based on 8 conserved proteins, the slow-growing mycobacterial species corresponding to the “*Tuberculosis-Simiae*” clade formed a distinct clade in the 16S rRNA tree. The species corresponding to the “*Terrae”* clade also branched in the immediate proximity of the “*Tuberculosis-Simiae*” clade, with members of the “*Triviale*” clade (viz. *M. triviale, M. koreense*, and *M. parakoreense*) forming a deeper-branching lineage. However, in contrast to the different trees based on protein sequences, the rapid-growing *Mycobacterium* species exhibited extensive polyphyly and their interrelationships were poorly resolved. In particular, the members of the “*Abscessus-Chelonae”* clade formed a monophyletic lineage within the other rapid-growing *Mycobacterium* species, whereas the relationships among the other rapid growing species were difficult to discern.

### Genome relatedness of the members of the genus *Mycobacterium*

**Figure 2:**
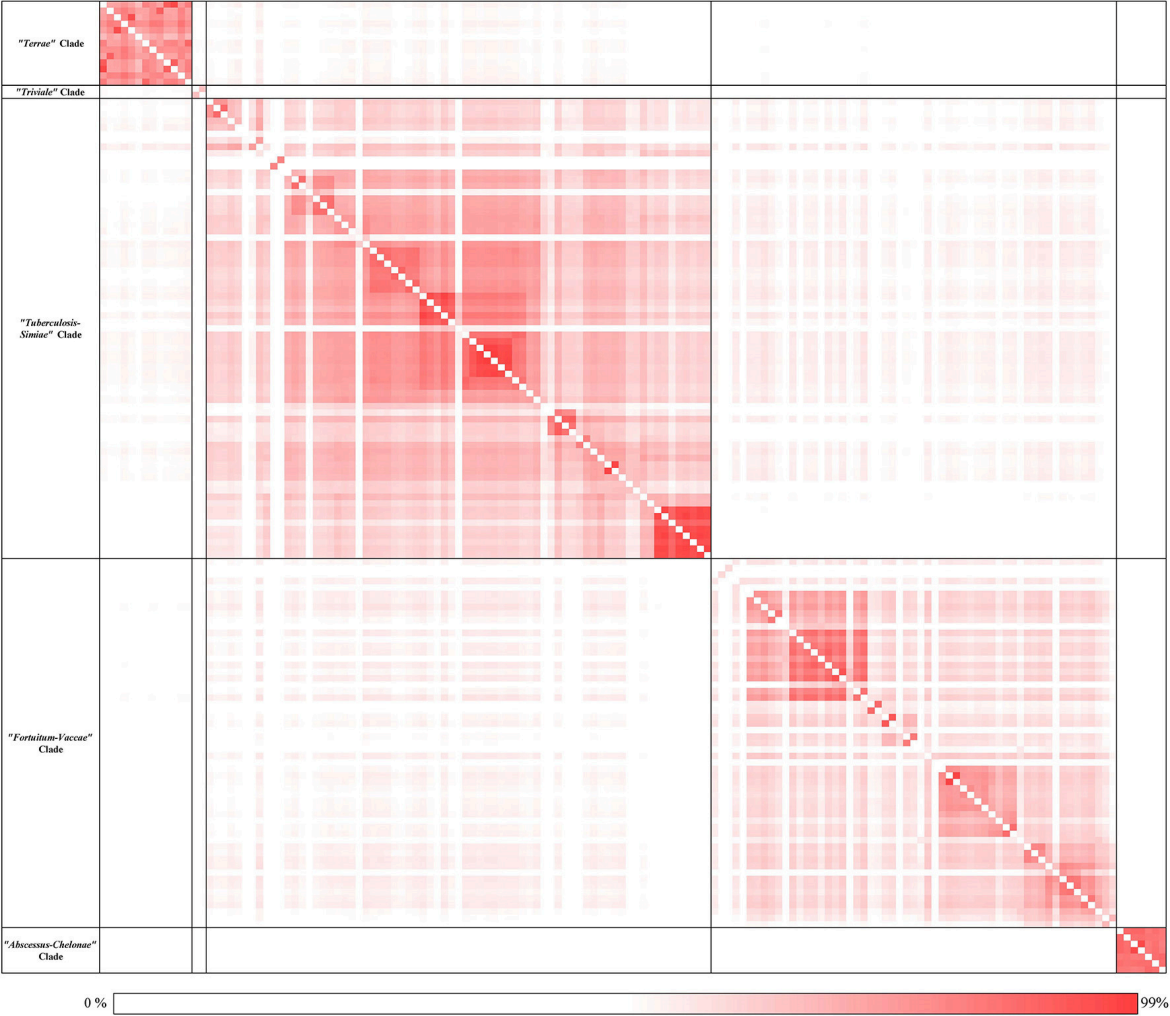
A matrix of the average Amino Acid Identity for the 150 *Mycobacterium* genomes used in this study. A darker shade represents higher similarity between the pair of genomes. The average amino acid identity between each pair of genomes was calculated as described in the Methods section. The numerical values underlying this matrix are provided in Supplementary Figure 3.

Based on genome sequences, the average amino acid identity between different species can be calculated to determine the overall genome relatedness of the species (Konstantinidis and Tiedje, 2007; Richter and Rossello-Mora, 2009; Thompson et al., 2013; Qin et al., 2014; Yarza et al., 2014). Pairwise amino acid identity was calculated based on the conserved protein families between each genome used in the analysis and the results of these analyses are presented in the form of a matrix in Figure 2. An expanded version of this matrix is provided in Supplementary Figure 3. As seen from the AAI matrix (Figure 2), the members of the four main clades observed in the phylogenetic trees (Figure 1 and Supplementary Figure 1) showed higher amino acid identity to members within each clade than to the other *Mycobacterium* species. Further, members of the “*Triviale*” clade could be clearly distinguished from the “*Terrae”* clade, based on their much lower amino acid identity to the members of this latter clade. In addition, members of the “*Abscessus-Chelonae”* clade exhibited a high degree of amino acid identity (Avg. 92%) to other members of this clade, but significantly lower similarity to members of the “*Fortuitum-Vaccae*” or the “*Tuberculosis-Simiae*” clades (Avg. 62%). The results of the genome relatedness analysis support the existence of the four main clades observed in the phylogenetic trees and also the distinctness of the “*Triviale”* clade from members of the “*Terrae*” clade.

### Molecular signatures specific for the genus *Mycobacterium* and its main clades

The results of phylogenomic studies and genomic similarity analysis indicated that the known mycobacterial species can be divided into five main groups including the “*Triviale*” clade. However, as the branching of species in phylogenetic trees can be affected by a large number of variables (Stackebrandt, 1992; Ludwig and Klenk, 2005; Klenk and Goker, 2010; Gupta, 2016b), it is important to confirm the genetic cohesiveness of the observed clades by independent means not involving phylogenetic analysis. Rare genetic changes, such as insertions and deletions in genes/proteins as well as novel genes/proteins (viz. CSIs and CSPs) which are uniquely shared by an evolutionary related group of organisms constitute synapomorphic characteristics, whose shared presence in a given group of organisms generally results from the occurrence of the genetic changes in a common ancestor of the group (Gupta, 1998, 2014, 2016b; Rokas and Holland, 2000; Dutilh et al., 2008). In our earlier work on Actinobacteria, we described large numbers of CSIs and CSPs which were distinctive characteristics of either the entire phylum or a number of different clades within this phylum at multiple phylogenetic/taxonomic levels (Gao and Gupta, 2005, 2012; Gao et al., 2006; Gupta et al., 2013b). Although the focus of this earlier study was not on mycobacteria, a limited number of CSIs and CSPs which were then specific for the genus *Mycobacterium* were also identified (Gao et al., 2006; Gao and Gupta, 2012). Since these earlier studies, genome sequences for a large number of other mycobacterial species have become available (Supplementary Table 1). In the present work, we have carried out comprehensive comparative genomic studies on members of the genus *Mycobacterium*, to identify molecular markers (CSIs and CSPs) that are specific characteristics of either all mycobacterial species or of the identified main clades within this genus. The results of these analyses have identified 172 molecular markers (CSIs and CSPs) that are uniquely found in either all mycobacteria or by the members of different main clades identified by phylogenomic studies. Brief descriptions of the characteristics of the identified molecular markers and their group specificities are provided below.

### Molecular signatures (CSIs and CSPs) specific for the genus *Mycobacterium*

**Figure 3:**
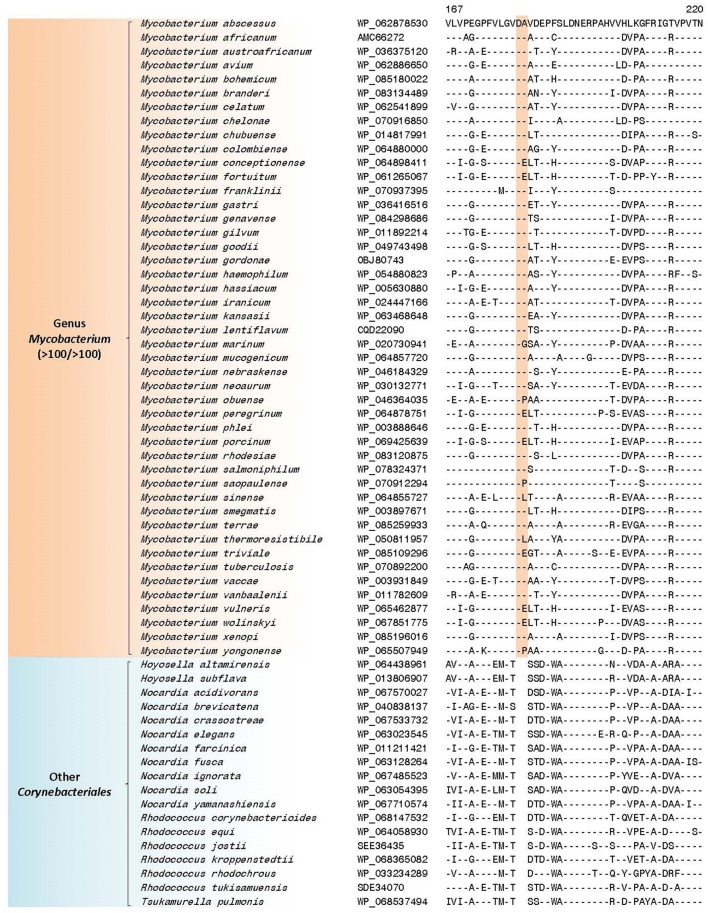
Partial sequence alignment of a conserved region of the ergothioneine biosynthesis protein EgtB showing a two amino acid insertion (boxed) exclusively found in members of the genus *Mycobacterium* and not present in other *Corynebacteriales*. Dashes (-) in all alignments denote identity with the amino acid shown in the top sequence. Sequence information for only limited numbers of species is presented in this figure; a detailed alignment for this CSI is shown in Supplementary Figure 4. Information for additional CSIs specific for the genus *Mycobacterium* are provided in Supplementary Figures 4–13 and summarized in Table 1.

Our analysis has identified 10 CSIs in proteins involved in diverse functions that are uniquely found in all available mycobacterial homologs. An example of a CSI that is specific for the genus *Mycobacterium* is shown in Figure 3. In the partial sequence alignment of the protein EgtB (ergothioneine biosynthesis protein), a two amino acid insertion in a conserved region is exclusively found in all members of the genus *Mycobacterium*, but it is not present in the top 500 homologs of this protein sequence in other bacteria. Ergothionine is a naturally occurring amino acid (thiourea derivative of histidine), whose synthesis is uniquely carried out by only certain groups of actinobacteria as well as some cyanobacteria and fungi (Fahey, 2001). More detailed sequenced information for this CSI as well as sequence information for 9 other CSIs in important proteins, which are also specific for the genus *Mycobacterium* is provided in Supplementary Figures 4–13 and their main characteristics are summarized in Table 1. Of the described CSIs, the CSI in the protein orotidine 5'-phosphate decarboxylase (Supplementary Figure 7) was identified in our earlier work (Gao and Gupta, 2012). Although the number of sequenced mycobacterial genomes has increased many folds, this CSI is still found only in members of the genus *Mycobacterium*.

**Table 1:** Conserved signature indels (CSIs) that are specific for different members of the genus *Mycobacterium* and those which are lacking only in members of the “*Abscessus-Chelonae”* clade.

**Protein name**	**Accession number**	**Figure number**	**Indel size**	**Indel position**	**Specificity**
Ergothioneine biosynthesis protein EgtB^a^	WP_062878530	Figure 3 Supplementary Figure 4	2aa ins	167–220	Genus *Mycobacterium*
Precorrin-4 C(11)-methyltransferase	WP_078061976	Supplementary Figure 5	1aa ins	159–206	
NAD(P)H-quinone dehydrogenase	WP_062879231	Supplementary Figure 6	3aa ins	9–56	
Orotidine 5′-phosphate decarboxylase	WP_062879058	Supplementary Figure 7	1aa del	39–71	
Deoxyribonuclease IV	WP_031666830	Supplementary Figure 8	4aa del	109–149	
Serine hydrolase	WP_062879883	Supplementary Figure 9	3aa ins	122–166	
Peptidase C69^a^^,^^b^	WP_070410295	Supplementary Figure 10	1aa del	279–327	
SGNH/GDSL hydrolase family protein^a^	WP_031743956	Supplementary Figure 11	1aa del	95–143	
Succinate dehydrogenase^a^	WP_062880084	Supplementary Figure 12	1aa ins	27–56	
N-dimethylarginine dimethylaminohydrolase^a^^,^^b^	SGA93253	Supplementary Figure 13	1aa del	109–153	
Nif3-like dinuclear metal center hexameric protein	WP_066808468	Figure 4 Supplementary Figure 14	2aa del	31–75	Genus *Mycobacterium* except the “*Abscessus-Chelonae”* Clade
Phosphoribosylamine-glycine ligase	CKM81105	Supplementary Figure 15	5 aa ins	106–161	
D-alanyl-D-alanine carboxypeptidase/D-alanyl-D-alanine-endopeptidase	WP_083039002	Supplementary Figure 16	1aa del	418–449	
Heat-inducible transcriptional repressor HrcA	WP_031668340	Supplementary Figure 17	2aa ins	193–228	

a *Only in comparison to other Corynebacteriales*.

b *Homologues of Hoyosella species were absent in BLASTp searches*.

We have previously described a number of CSPs, whose homologs were uniquely found in the then sequenced mycobacterial species (Gao et al., 2006; Gao and Gupta, 2012). In light of the large increase in the number of sequenced mycobacterial genomes, the group specificities of the previously described CSPs were re-examined. Results of these analyses reveal that despite >20-fold increase in the number of sequenced mycobacterial genomes since these CSPs were first identified (Gao et al., 2006), 9 of the CSPs reported in our earlier work are still specific for members of the genus *Mycobacterium* and no homologs showing significant similarities to these proteins are present in other bacteria (Table 2). In view of the unique shared presence of these 10 CSIs and 9 CSPs by either all or most members of the genus *Mycobacterium* (except for an isolated exception), the genetic changes leading to these genetic markers most likely initially occurred in a common ancestor of the genus *Mycobacterium* and then retained by all descendant species.

**Table 2:** Conserved signature proteins (CSPs) specific for the genus *Mycobacterium* and members of the “*Abscessus-Chelonae”* clade.

**Gene or protein**	**Accession number**	**Function**	**Length**	**Specificity**
Hypothetical protein^a^^,^^c^	WP_011723520.1	Hypothetical	277	Genus *Mycobacterium* (Gao and Gupta, 2012)
Hypothetical protein^a^	WP_011723901.1	Hypothetical	129	
Hypothetical protein^a^	WP_011723955.1	Hypothetical	220	
Membrane protein^a^^,^^c^	WP_011724283.1	Atrophin-1	253	
PE-PPE domain-containing protein^a^^,^ ^b^	WP_011724324.1	Hypothetical	376	
DUF2561 domain-containing protein^a^^,^^c^	WP_011724709.1	Hypothetical	210	
Membrane protein^a^^,^^c^	WP_009976570.1	Actinobacterial Holin-x	131	
Hypothetical protein^a^	WP_003876314.1	Hypothetical	61	
Hypothetical protein^a^	WP_003874755.1	Hypothetical	116	
MAB_0188^c^	YP_001700942.1	Hypothetical	60	“*Abscessus-Chelonae”* Clade
MAB_0375	YP_001701128.1	Hypothetical	99	
MAB_0601	YP_001701353.1	Hypothetical	98	
MAB_2852^c^	YP_001703585.1	Hypothetical	108	
MAB_3058	YP_001703790.1	Hypothetical	127	
MAB_3079^c^	YP_001703811.1	Hypothetical	193	
MAB_1107^c^	YP_001701850.1	Hypothetical	74	
MAB_1519	YP_001702259.1	tRNA synthetase class II	127	
MAB_1642	YP_001702381.1	Hypothetical	60	
MAB_0008	YP_001700765.1	Hypothetical	75	
MAB_0245^c^	YP_001700999.1	Hypothetical	74	
MAB_2487^b^	YP_001703222.1	Hypothetical	75	
MAB_3020^c^	YP_001703752.1	Hypothetical	55	
MAB_1440^c^	YP_001702180.1	Hypothetical	76	
MAB_0014	YP_001700771.1	Hypothetical	74	
MAB_0015	YP_001700772.1	Hypothetical	95	
MAB_0345	YP_001701098.1	Hypothetical	170	
MAB_0448^c^	YP_001701201.1	Hypothetical	67	
MAB_0456	YP_001701209.1	Hypothetical	94	
MAB_0460	YP_001701213.1	Hypothetical	146	
MAB_2549	YP_001703284.1	Hypothetical	69	
MAB_1765	YP_001702504.1	Hypothetical	98	
MAB_1767	YP_001702506.1	Hypothetical	81	
MAB_1806^b^	YP_001702544.1	Mycobacterial 2 TMS Phage Holin (M2) Holin Family	138	

a *Previously identified by Gao and Gupta (2012)*.

b *Some exceptions are present*.

c *A significant BLASTp hit was also observed for 1 to 2 other species of the genus Klebsiella*.

### Molecular signatures specific for the “*Abscessus-Chelonae”* clade and supporting the deep branching of this group within the genus *Mycobacterium*

**Figure 4:**
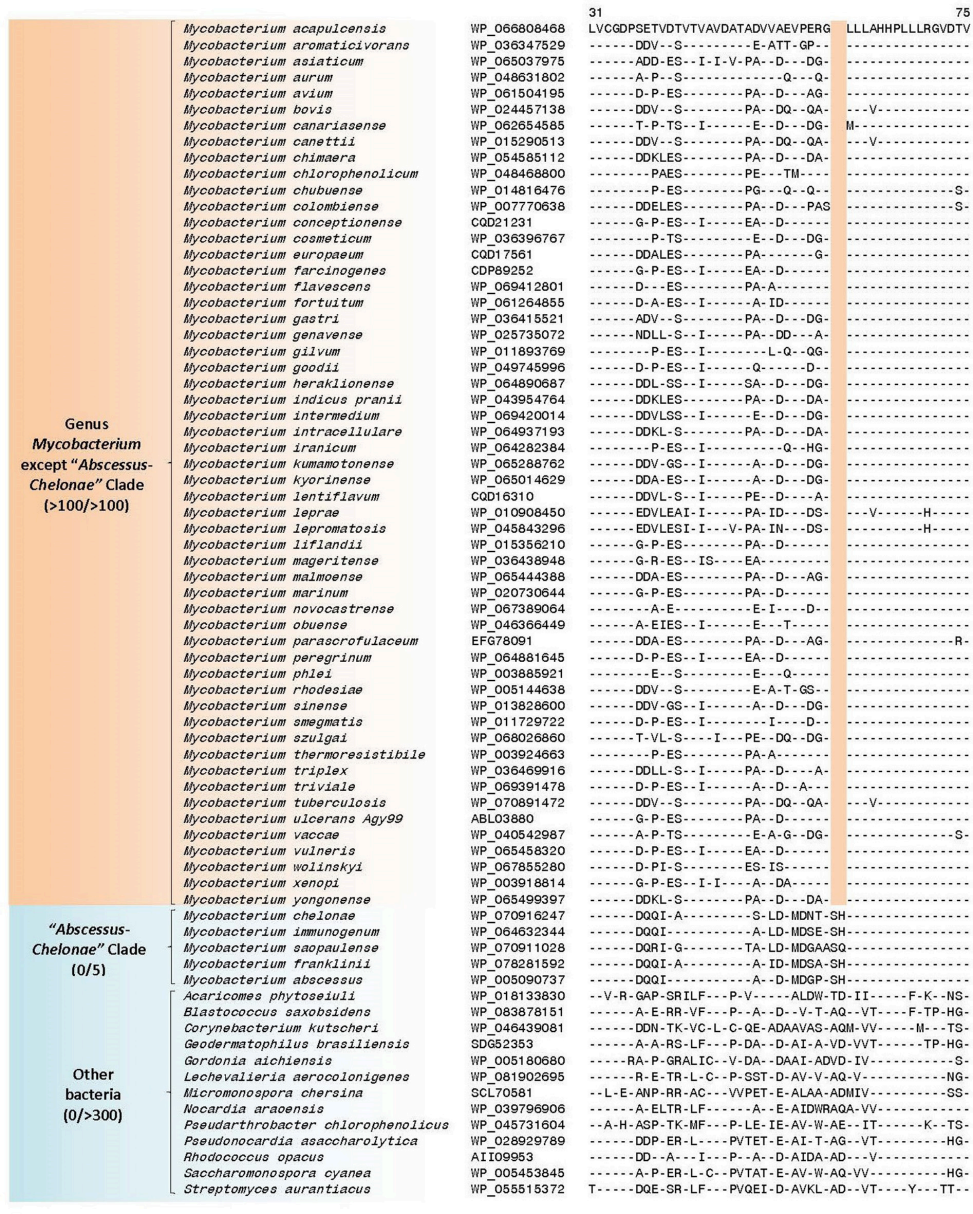
A partial sequence alignment of a conserved region of Nif3-like protein exhibiting a two amino acid deletion that is specific for members of the genus *Mycobacterium* except members of the “*Abscessus-Chelonae”* clade; a detailed alignment for this CSI is shown in Supplementary Figure 14. Information for additional CSIs specific for the genus *Mycobacterium* are provided in Supplementary Figures 14–17 and summarized in Table 1. Dashes (-) in all alignments denote identity with the amino acid shown in the top sequence.

The “*Abscessus-Chelonae”* clade, also referred to as *M. chelonae* or *M. abscessus* complex (Adékambi and Drancourt, 2004; Medjahed et al., 2010; Tortoli, 2012; Wee et al., 2017), consists of six members and it has recently gained clinical attention in light of its emerging pathogenicity to humans (Medjahed et al., 2010; Tortoli, 2014). In the phylogenetic trees constructed in our work, members of this clade form a monophyletic grouping which comprises the deepest branching lineage among the *Mycobacterium* species (Figures 1A,B and Supplementary Figure 1). The deep branching of the “*Abscessus-Chelonae”* clade in comparison to the other *Mycobacterium* species is also independently supported by 4 CSIs in four different proteins which are commonly shared by the homologs of all other mycobacterial species except those from the “*Abscessus-Chelonae”* clade. One example of a CSI depicting this pattern is presented in Figure 4, where in the partial sequence alignment of Nif3-like dinuclear metal center hexameric protein, a two amino acid deletion in a conserved region is present in all members of the genus *Mycobacterium* except members of the “*Abscessus-Chelonae”* clade. Additional information for this CSI and the sequence information for the three other CSIs exhibiting similar species distributions is provided in Supplementary Figures 14–17 and their main characteristics are summarized in Table 1. Based upon the species distributions of these CSIs, the genetic changes leading to them have likely occurred in a common ancestor of the other *Mycobacterium* species after the divergence of the “*Abscessus-Chelonae”* clade.

**Figure 5:**
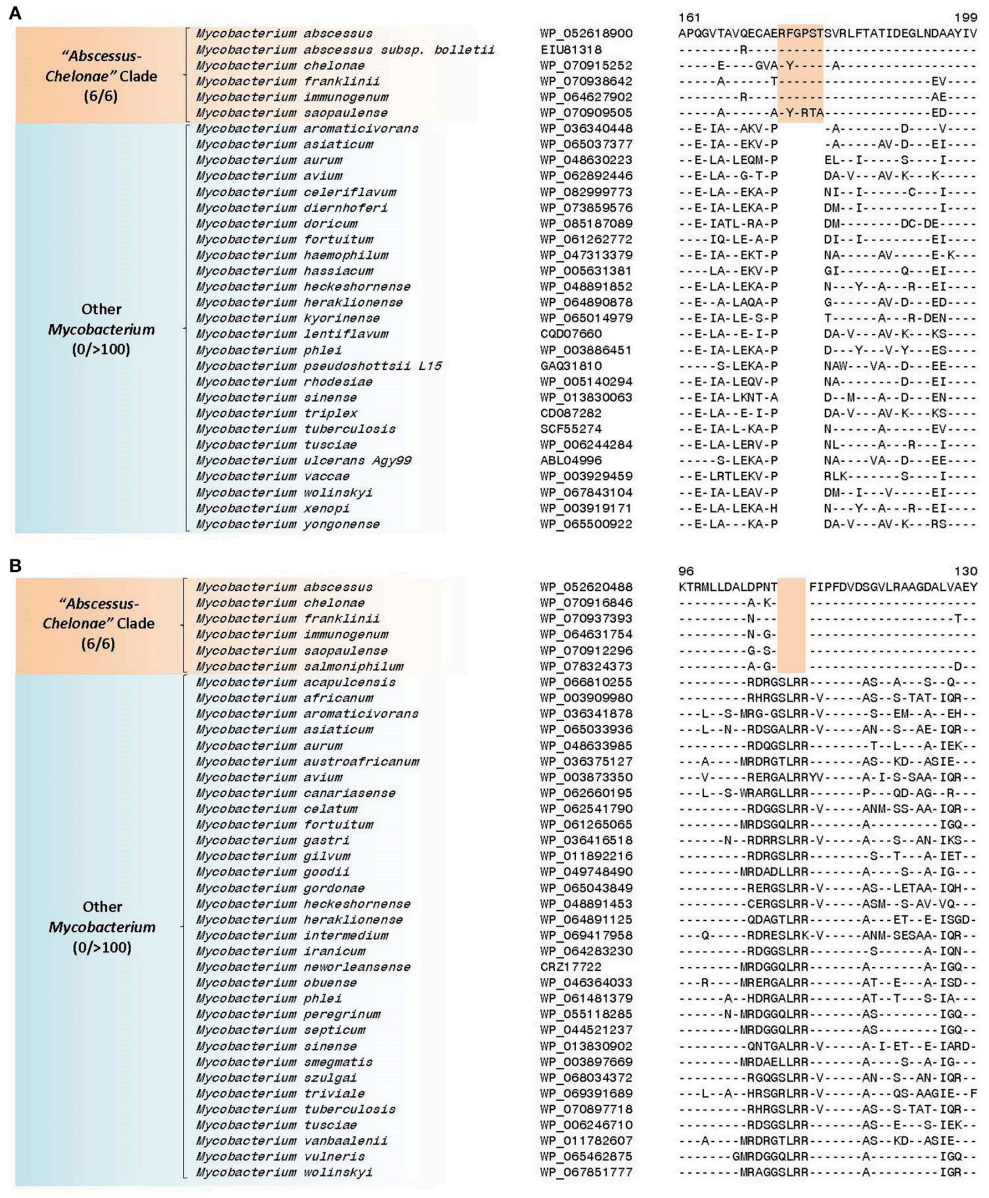
**(A)** Partial sequence alignment of the protein uracil phosphoribosyltransferase showing a six amino acid insertion that is specific for the “*Abscessus-Chelonae”* clade; **(B)** Sequence alignment of L-histidine N(alpha)-methyltransferase showing a four amino acid deletion that is also specific for the “*Abscessus-Chelonae”* clade. More detailed alignments for these CSIs are shown in Supplementary Figures 18, 19 respectively. Additional CSIs that are specific for this clade are summarized in Table 3 and sequences of these are provided in Supplementary Figures 15, 18–43.

Our analyses have also identified 27 CSIs in proteins involved in diverse functions that are uniquely shared by members of the “*Abscessus-Chelonae”* clade providing strong evidence of the genetic cohesiveness and distinctness of this group of mycobacteria. Two examples of the CSIs specific for the “*Abscessus-Chelonae”* clade are shown in Figure 5. Figure 5A shows a partial sequence alignment of the protein uracil phosphoribosyltransferase, where a six amino acid insertion in a conserved region is present in all members of the “*Abscessus-Chelonae”* clade but absent in the homologs from all other *Mycobacterium* species as well as other groups of bacteria. Likewise, Figure 5B shows a four amino acid deletion in the sequence alignment of protein L-histidine N(alpha)-methyltransferase, which is also specific for the “*Abscessus-Chelonae”* clade. More detailed information for these CSIs and the 25 other identified CSIs, which are also specific for the “*Abscessus-Chelonae”* clade, is provided in Supplementary Figures 15, 18–43 and their main characteristics are summarized in Table 3. In addition to these CSIs, our work has also identified 24 CSPs listed in Table 2, for which homologs exhibiting significant similarity are only found in members of the “*Abscessus-Chelonae”* clade. Thus, the distinctness of the “*Abscessus-Chelonae”* clade from all other mycobacteria is strongly supported by 51 highly-specific molecular signatures identified in this work.

**Table 3:** Conserved signature indels (CSIs) specific to members of the “*Abscessus-Chelonae”* clade.

**Protein name**	**Accession number**	**Figure number**	**Indel size**	**Indel position**	**Specificity**
Uracil phosphoribosyltransferase	WP_052618900	Figure 5A Supplementary Figure 18	6aa ins	161–199	“*Abscessus-Chelonae”* Clade
L-histidine N(alpha)-methyltransferase	WP_052620488	Figure 5B Supplementary Figure 19	4aa del	94–130	
DUF58 domain-containing protein	WP_074245867	Supplementary Figure 20	10aa ins	364–407	
NADH-quinone oxidoreductase subunit G	WP_062878914	Supplementary Figure 21	4aa ins	724–762	
ATP-dependent helicase	WP_052624897	Supplementary Figure 22	3aa ins	169–208	
tRNA (cytidine(34)-2′-O)-methyltransferase	WP_005056099	Supplementary Figure 23	1aa del	13–52	
Glutamine-fructose-6-phosphate transaminase (isomerizing)	WP_052618678	Supplementary Figure 24	1aa del	49–81	
Error prone DNA polymerase	WP_052620523	Supplementary Figure 25	1aa ins	674–715	
2-amino-4-hydroxy-6-hydroxymethyldihydropteridine diphosphokinase	WP_052622963	Supplementary Figure 26	2aa del	39–78	
DEAD/DEAH box helicase	WP_052619503	Supplementary Figure 27	1aa del	253–295	
Anion transporter	WP_052620306	Supplementary Figure 28	1aa del	51–90	
Membrane protein	WP_005081027	Supplementary Figure 29	6aa ins	331–379	
Nicotinate-nucleotide adenylyltransferase	WP_005074554	Supplementary Figure 30	2aa del	124–163	
CoA ester lyase	WP_052529870	Supplementary Figure 31	2aa del	172–207	
Hypothetical protein	WP_052613689	Supplementary Figure 32	6aa ins	342–382	
Hypothetical protein	WP_052613689	Supplementary Figure 33	2aa del	808–844	
Hypothetical protein	WP_057138049	Supplementary Figure 34	2aa ins	359–399	
Hypothetical protein	WP_052618664	Supplementary Figure 35	1aa del	199–235	
Bifunctional ADP-dependent (S)-NAD(P)H-hydrate dehydratase/NAD(P)H-hydrate epimerase	WP_052543860	Supplementary Figure 36	3aa ins	318–354	
Hypothetical protein	WP_052621243	Supplementary Figure 37	1aa del	181–208	
Pyridoxal phosphate-dependent aminotransferase	WP_057138073	Supplementary Figure 38	4aa ins	157–194	
Carotenoid oxygenase	WP_062880095	Supplementary Figure 39	1aa ins	221–259	
Hypothetical protein	WP_062879314	Supplementary Figure 40	1aa del	139–168	
Hypothetical protein	WP_062879407	Supplementary Figure 41	3aa ins	340–367	
SAM-dependent methyltransferase	WP_062879423	Supplementary Figure 42	2aa ins	92–128	
SAM-dependent methyltransferase	WP_062879423	Supplementary Figure 43	1aa ins	12–47	
Phosphoribosylamine-glycine ligase	CKM81105	Supplementary Figure 15	2aa ins	106–161	

### Molecular signatures specific for the “*Fortuitum-Vaccae”* clade

**Figure 6:**
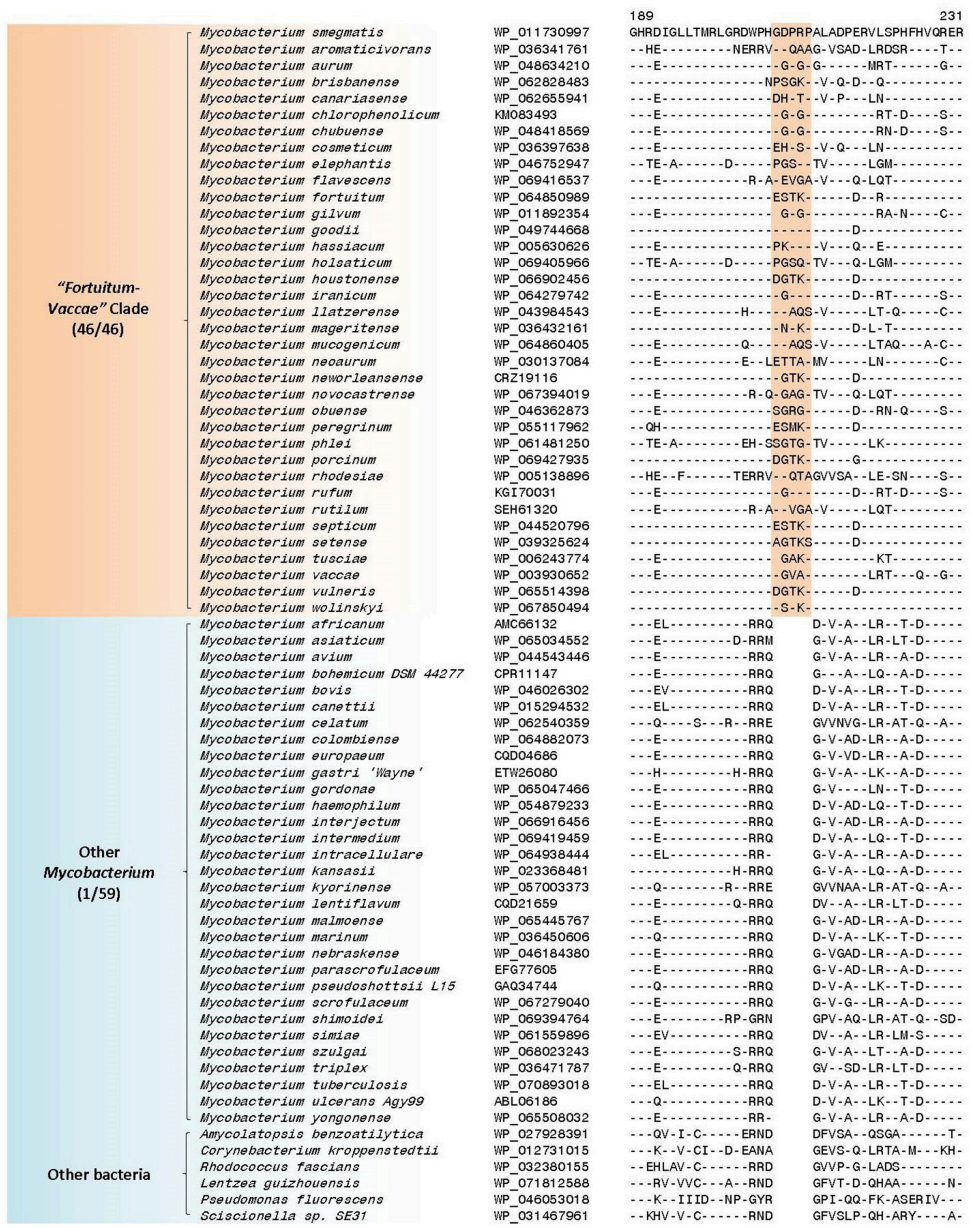
A partial sequence alignment of a conserved region of LacI family transcriptional regulator showing a five amino acid insertion that is specific for the “*Fortuitum-Vaccae”* clade; a more detailed alignment of this CSI is shown in Supplementary Figure 44. Sequence information for additional CSIs that are specific for this clade is shown in Supplementary Figures 44–47 and summarized in Table 4.

The “*Fortuitum-Vaccae*” clade as designated here (see Figure 1) encompasses all rapid-growing mycobacterial species, except those from the “*Abscessus-Chelonae”* clade. In the present work, 4 CSIs and 10 CSPs have been identified that are specific for either all or most members of the “*Fortuitum-Vaccae*” clade and support the monophyletic clustering of these species as observed in the phylogenomic trees (Figure 1). One of the identified CSIs, which are specific for the “*Fortuitum-Vaccae*” clade, is found in the *LacI* family transcriptional regulator. In the partial sequence alignment of this protein shown in Figure 6, a five amino acid insert in a conserved region is exclusively found in different members of the “*Fortuitum-Vaccae*” clade but it is not found in any other mycobacteria. Three other CSIs showing similar species specificities are present in three other proteins. Detailed sequence information for all of these CSIs is provided in the Supplementary Figures 44–47 and the main characteristics of all CSIs specific for the “*Fortuitum-Vaccae*” clade are summarized in Table 4.

**Table 4:** Conserved Signature Indels (CSIs) specific for members of the “*Fortuitum-Vaccae”* clade, Slow-Growing *Mycobacterium* (“*Tuberculosis-Simiae”* + “*Terrae”* clades), and “*Tuberculosis-Simiae”* clade.

**Protein name**	**Accession number**	**Figure number**	**Indel size**	**Indel position**	**Specificity**
LacI family transcriptional regulator	WP_036341761	Figure 6 Supplementary Figure 44	5aa ins	189–231	“*Fortuitum-Vaccae”* Clade
Cyclase	WP_066808156	Supplementary Figure 45	2aa ins	243–280	
CDP-x	WP_036344961	Supplementary Figure 46	1aa ins	49–75	
CDP-diacylglycerol–serine O-phosphatidyltransferase	WP_066811333	Supplementary Figure 47	1aa del	112–160	
Alkyl/aryl sulfatase	WP_083113621	Figure 7 Supplementary Figure 48	1aa ins	123–155	*Mycobacterium* Slow-Growers
Succinate dehydrogenase iron-sulfur subunit	WP_083139296	Supplementary Figure 49	4aa ins	30–68	
Hypothetical protein	WP_009976218	Supplementary Figure 50	1aa del	51–100	
Hypothetical protein	WP_031701648	Figure 8 Supplementary Figure 51	1aa del	81–119	“*Tuberculosis-Simiae”* Clade
Aldehyde dehydrogenase family protein	WP_080699385	Supplementary Figure 52	2aa del	403–450	
23S rRNA (guanosine(2251)-2′-O)-methyltransferase RlmB	WP_083139967	Supplementary Figure 53	1aa del	136–176	

BLASTp searches on the protein sequences from the genome of *Mycobacterium aurum* (LSHTM) have also identified 10 CSPs, whose homologs, except for rare exceptions, are only found in the “*Fortuitum-Vaccae*” clade of *Mycobacterium* species. Most of these CSPs are hypothetical proteins and their characteristics are summarized in Table 5. For the first four CSPs listed in Table 5, the homologs are present in different members of the “*Fortuitum-Vaccae*” clade, while for the remaining six CSPs, although they are specific for the “*Fortuitum-Vaccae*” clade, homologs were not detected in some members of this clade. In all, our identification of 14 molecular markers (4 CSIs and 10 CSPs), which are uniquely shared by members of the “*Fortuitum-Vaccae*” clade support its monophyletic origin and genetic cohesiveness.

**Table 5:** Conserved signature proteins (CSPs) specific for members of the “*Fortuitum-Vaccae”* clade, Slow-Growing *Mycobacterium* (“*Tuberculosis-Simiae”* + “*Terrae”* + “*Triviale*” clades), and “*Tuberculosis-Simiae”* clade.

**Gene or protein**	**Accession number**	**Function**	**Length**	**Specificity**
Hypothetical protein	WP_048630777.1	Hypothetical	91	“*Fortuitum-Vaccae”* Clade
Hypothetical protein^a^^,^^b^	WP_048632025.1	Hypothetical	124	
Hypothetical protein	WP_048632497.1	Hypothetical	79	
Hypothetical protein^b^	WP_048634851.1	Hypothetical	75	
Hypothetical protein^c^	WP_048633467.1	Hypothetical	200	
Hypothetical protein^c^	WP_048633322.1	Hypothetical	151	
Hypothetical protein^b^^,^^c^	WP_048631132.1	Ribonuclease E	320	
Hypothetical protein^c^	WP_048634509.1	Hypothetical	93	
Hypothetical protein^b^^,^^c^	WP_048630657.1	Hypothetical	153	
Hypothetical protein,^c^	WP_048632441.1	Prolipoprotein diacylglyceryl transferase	338	
PPE Family protein	YP_177721.1	Hypothetical	3,300	*Mycobacterium* Slow-Growers
PE Family protein PE36	YP_178025.1	Hypothetical	103	
PE Family protein^a^^,^^b^	WP_011725130.1	Hypothetical	99	
MAP_RS07685^a^^,^^b^	WP_003874405.1	WXG100 family	94	
Histone-like protein HNS	NP_218369.1	Histone-like protein	134	“*Tuberculosis-Simiae”* Clade
Rv4010	YP_004837050.1	Hypothetical Protein	83	
Membrane protein	NP_217322.1	Hypothetical Protein	63	

a *Previously identified by Gao and Gupta (2012)*.

b *Some exceptions are present*.

c *Homologues from all species were not observed in BLASTp searches*.

### Molecular signatures that are specific for the slow-growing *Mycobacterium*

**Figure 7:**
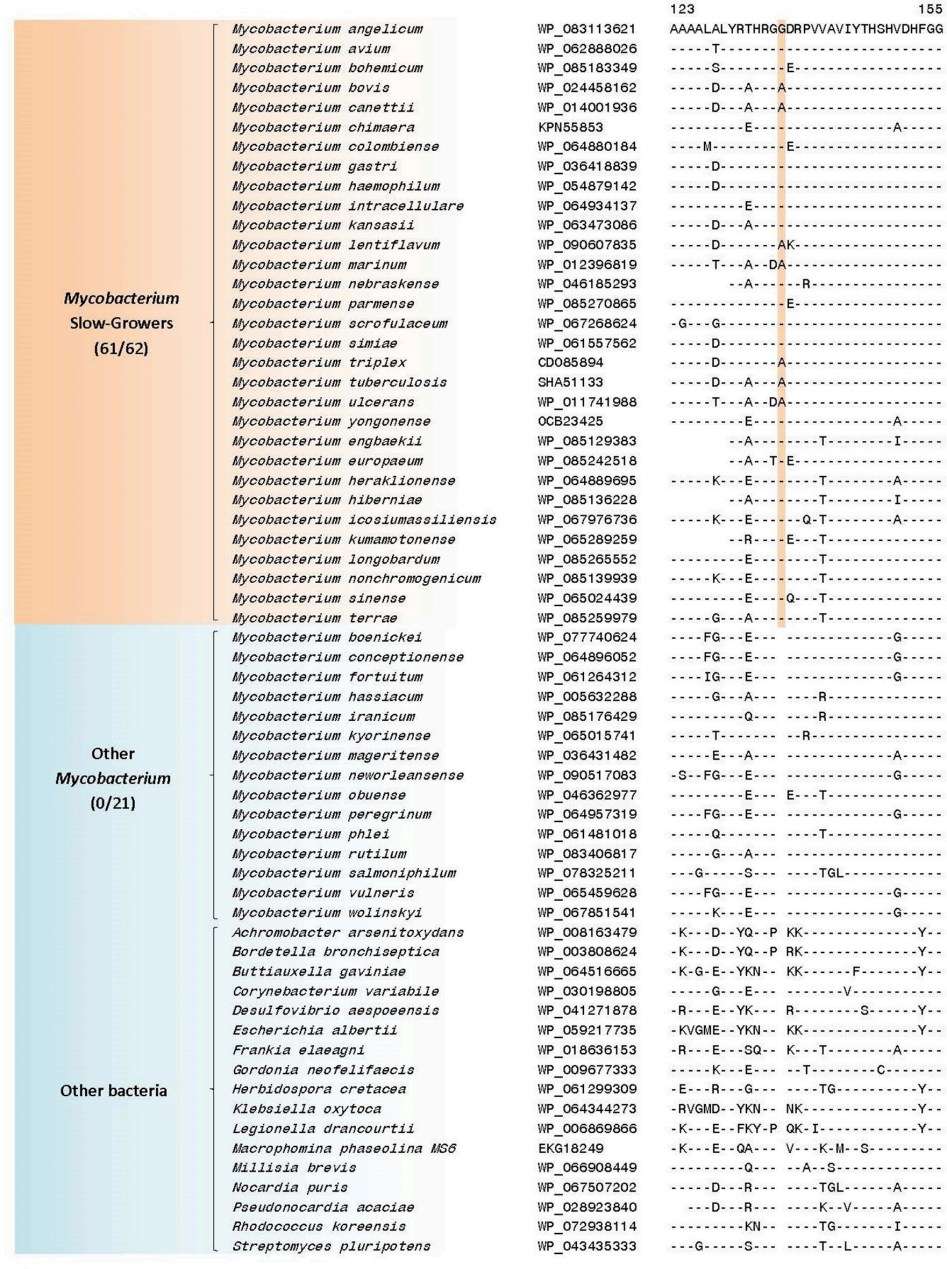
A partial sequence alignment of a conserved region of the protein alkyl/aryl sulfatase showing a one amino acid insertion that is specific for the *Mycobacterium* slow-growers (i.e., “*Tuberculosis-Simiae”* + “*Terrae”*) clade; a detailed alignment of this CSI is shown in Supplementary Figure 48. Additional CSIs that are specific for this clade are summarized in Table 4 and their sequence alignments are shown in Supplementary Figures 48–50.

The slow-growing *Mycobacterium* species generally form a monophyletic clade in most phylogenetic trees based on protein sequences (see Figure 1 and Supplementary Figure 1) as well as those based on the 16S rRNA gene sequences (see Supplementary Figure 2) (Devulder et al., 2005; Kim et al., 2005; Hartmans et al., 2006; Mignard and Flandrois, 2008; Magee and Ward, 2012; Tortoli, 2012; Quast et al., 2013; Lory, 2014; Wang et al., 2015; Wee et al., 2017). The monophyly of the slow-growing *Mycobacterium* clade is also supported by 3 CSIs and 4 CSPs that have been identified in this study. One example of a CSI that is largely specific for the slow-growing *Mycobacterium* clade is shown in Figure 7. In the sequence alignment of alkyl-aryl sulfatase protein, a one amino acid insert in a conserved region is present in all of the homologs from slow-growing *Mycobacterium* species, but it is not found in the homologs of other *Mycobacterium* species. Detailed sequence information for this CSI and the two other CSIs showing similar specificities is provided in Supplementary Figures 48–50 and their main characteristics are summarized in Table 4. As noted above, the homologs for four of the identified CSPs (Accession numbers: YP_177721.1, YP_178025.1, WP_011725130.1, WP_003874405.1) are also specifically found in slow-growing *Mycobacterium* species (Table 5). The last two of these CSPs were identified by our earlier work based on limited number of genomes (Gao and Gupta, 2012) and they continue to be specific for this large clade of mycobacteria. Further, of the identified CSPs, which are specific for the slow-growing mycobacterial clade, three of the CSPs correspond to the PE or PPE family of proteins, which are often involved in mycobacterial virulence (Mukhopadhyay and Balaji, 2011).

In our phylogenetic trees, the slow-growing mycobacterial species form three main clades including a clade consisting of *M. triviale* and *M. koreense* (“*Triviale*” clade). The genetic cohesiveness of these clades of slow-growing mycobacteria is also supported by a large number of molecular signatures that are described below.

### Molecular signatures for the “*Tuberculosis-Simiae*” clade

**Figure 8:**
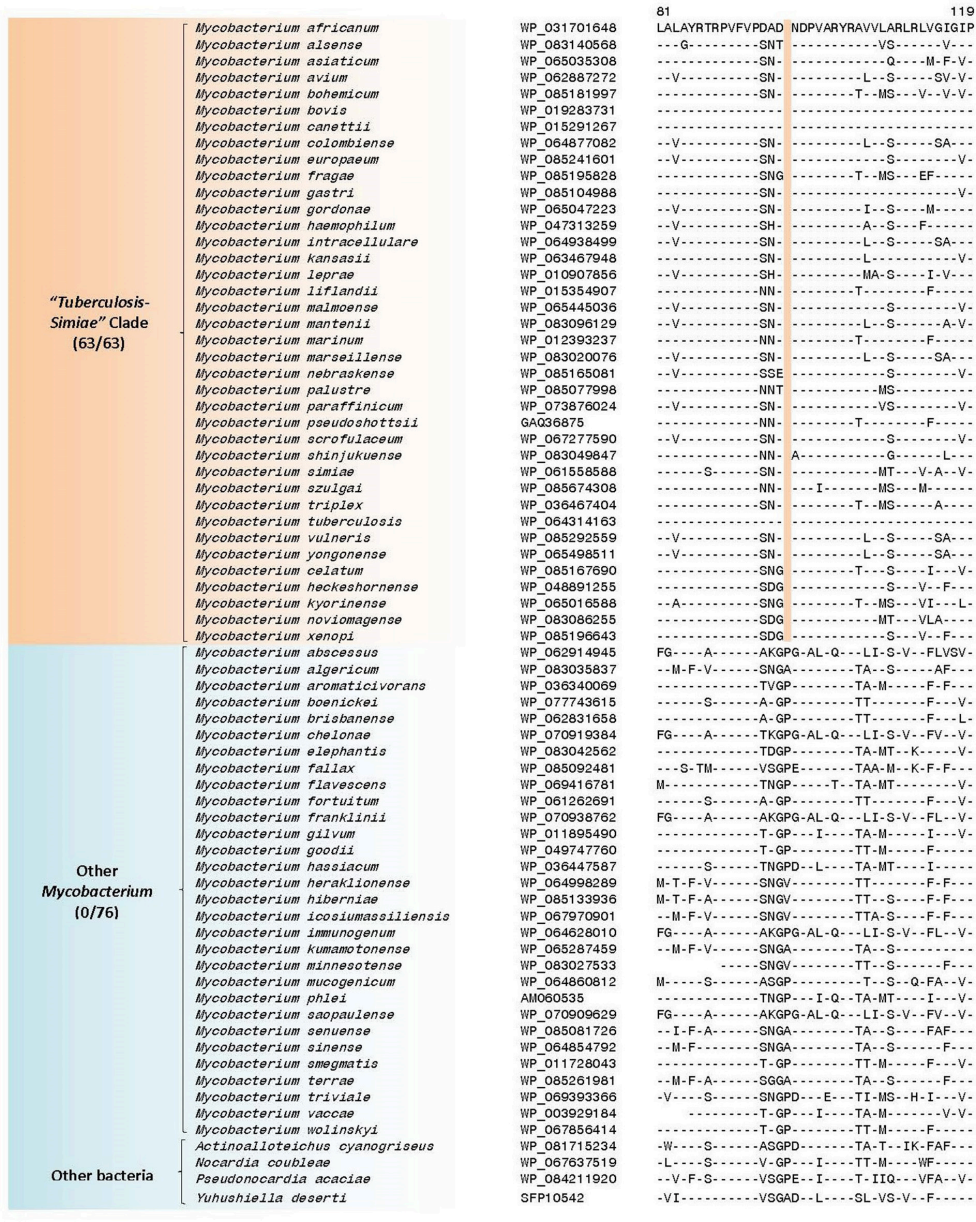
Partial sequence alignment of a conserved region of a hypothetical protein showing a one amino acid deletion exclusively found in members of the “*Tuberculosis-Simiae”* clade; a detailed alignment of this CSI is shown in Supplementary Figure 51. Additional CSIs that are specific for this clade are shown in Supplementary Figures 51–53 and information for them is summarized in Table 4.

The “*Tuberculosis-Simiae*” clade in our work is comprised of all other slow-growing mycobacteria except those from the “*Terrae*” and “*Triviale*” clades. This clade encompasses various pathogenic *Mycobacterium* species including those from the *M. tuberculosis* complex, *M. avium* complex, *M. gordonae* clade, *M. kansasii* clade, *M. simiae* clade, as well as several other slow-growing species (Magee and Ward, 2012; Lory, 2014). We have identified a total of 3 CSIs that are specific for the “*Tuberculosis-Simiae*” clade (Table 4, Supplementary Figures 51–53). One example of a CSI specific for this clade, which is found in a protein of unknown function is shown in Figure 8, where a single amino acid deletion is found in all members of the “*Tuberculosis-Simiae*” clade, but it is not present in any other mycobacterial homolog. In addition to these CSIs, BLASTp searches on the proteins found in the genome of *Mycobacterium tuberculosis* H37Rv have identified 3 CSPs, whose homologs are only found in either all or most members of the “*Tuberculosis-Simiae*” clade. A summary of the CSPs which are specific for the “*Tuberculosis-Simiae*” clade is provided in Table 5 and of these CSPs, one protein (Genbank Accession Number NP_218369.1) is annotated as a histone-like protein.

### Molecular signatures demarcating the “*Terrae*” and “*Triviale*” clades of mycobacteria

**Figure 9:**
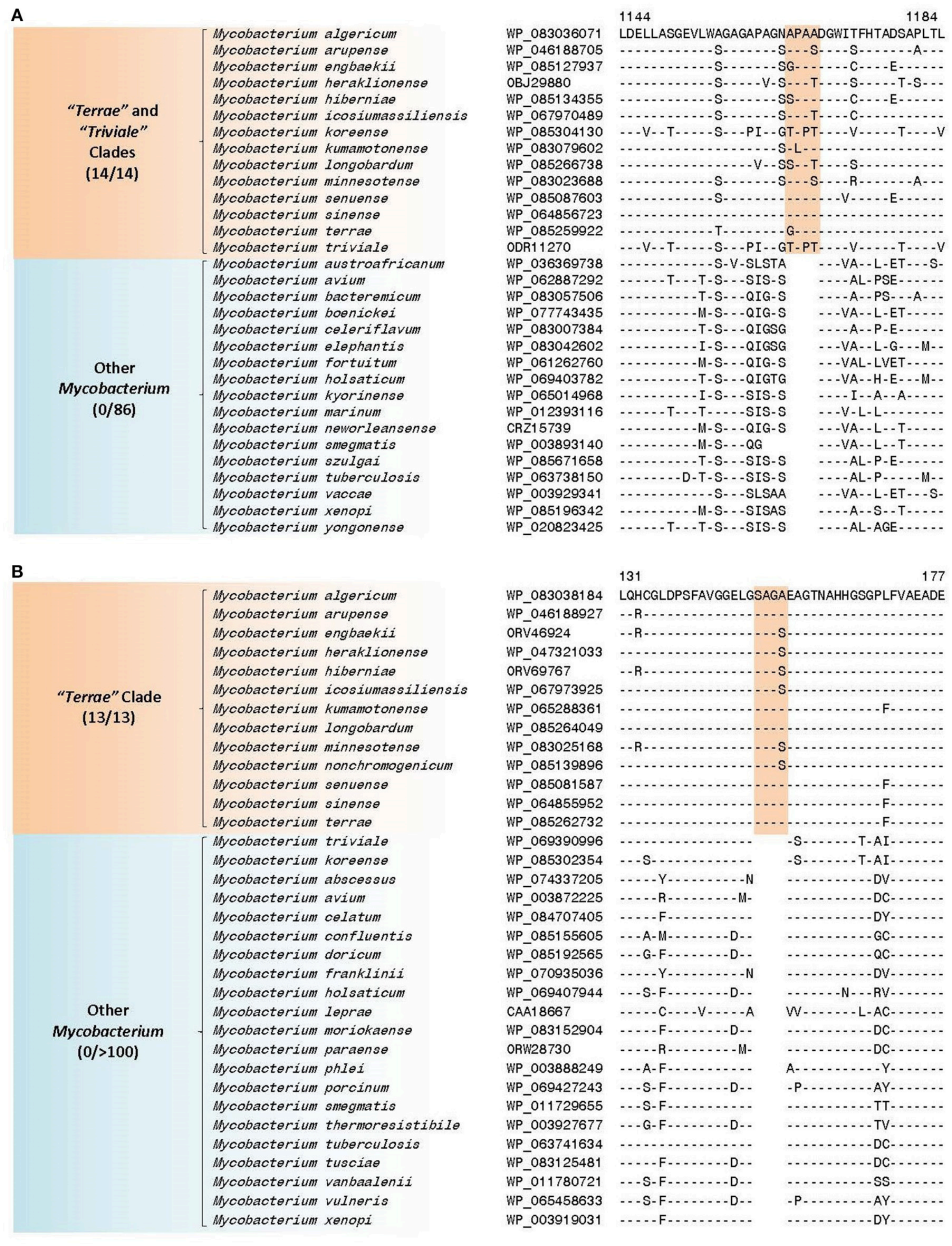
Partial sequence alignment of a conserved region of **(A)** ATP-dependent helicase showing a four amino acid insertion that is specific for the “*Terrae”* + “*Triviale”* clades and **(B)** UDP-N-acetylmuramate—L-alanine ligase showing a four amino acid insertion that is specific for only the members of the “*Terrae”* clade but lacking in members of the “*Triviale”* clade as well as other mycobacteria. More detailed alignments of these CSIs are shown in Supplementary Figures 54 and 74, respectively. Additional CSIs that are specific for this clade are shown in Supplementary Figures 35, 54–84 and summarized in Table 6.

The members of the “*M. terrae* complex” (Tortoli, 2012; Ngeow et al., 2015) has drawn attention recently as some members of this clade are opportunistic pathogens (Mignard and Flandrois, 2008; Kim et al., 2012, 2013; Tortoli, 2012, 2014; Tortoli et al., 2013; Ngeow et al., 2015; Vasireddy et al., 2016). In the core-genome protein trees and the tree based on 8 conserved proteins, members of the “*M. terrae* complex” form a monophyletic lineage consisting of two distinct subclades: a larger “*Terrae”* clade encompassing most of the species from the “*M. terrae* complex” and a deeper branching “*Triviale*” clade consisting of *M. triviale* and *M. koreense* (*M. parakoreense* also branches with these species in the 16S rRNA tree, Supplementary Figure 2). The phylogenetic distinctness of this larger “*Terrae”* + “*Triviale*” clade is also supported by a number of identified molecular signatures. In this work, we have identified 6 CSIs, which are specific for the larger “*Terrae* complex” consisting of the “*Terrae”* + “*Triviale*” clades (Table 6). Sequence information for one of the CSIs specific for the larger “*Terrae* complex” is presented in Figure 9A. In this case a four amino acid insertion in the protein ATP-dependent helicase is specifically present in all members of the “*Terrae* complex,” but it is not present in any other bacteria. Detailed sequence information for this CSI as well as other CSIs specific for this clade is presented in Supplementary Figures 54–59 and summarized in Table 6. In addition to these CSIs, which are commonly shared by the “*Terrae”* + “*Triviale*” clades, our analyses have also identified 26 other CSIs listed in Table 6, which are specifically shared by only the members of the “*Terrae”* clade and not present in *M. triviale* and *M. koreense*. An example of such a CSI consisting of a four amino acid insertion found in the protein UDP-N-acetylmuramate–L-alanine ligase is shown in Figure 9B. Sequence information for all the “*Terrae*” clade CSIs is presented in Supplemntary Figures 35, 60–84 and summarized in Table 6. These CSIs serve to indicate the distinctness of the species from the “*Terrae*” clade from the deeper branching *M. triviale* and *M. koreense* species, which are part of the “*Triviale*” clade.

**Table 6:** Conserved Signature Indels (CSIs) specific for members of the “*Terrae”* + “*Triviale”* clades or only the “*Terrae”* clade.

**Protein name**	**Accession number**	**Figure number**	**Indel size**	**Indel position**	**Specificity**
ATP-dependent helicase	WP_083036071	Figure 9A Supplementary Figure 54	4aa ins	1,144–1,184	“*Terrae” + “Triviale”* Clades
PDZ domain-containing protein	WP_083035840	Supplementary Figure 55	1aa del	290–324	
Ferredoxin reductase	WP_083035874	Supplementary Figure 56	3aa ins	199–263	
DUF2236 domain-containing protein	WP_083036515	Supplementary Figure 57	4aa del	37–80	
Hypothetical protein	WP_083040170	Supplementary Figure 58	1aa ins	100–141	
DUF4185 domain-containing protein	WP_083070918	Supplementary Figure 59	3aa ins	286–324	
Non-ribosomal peptide synthetase^a^	WP_083036306	Supplementary Figure 60	1aa del	497–532	“*Terrae”* Clade
Nucleoside hydrolase^a^	WP_085126548	Supplementary Figure 61	2aa del	134–174	
TetR/AcrR family transcriptional regulator^a^	WP_083037632	Supplementary Figure 62	1aa del	165–203	
Carbon starvation protein A^a^	WP_083035732	Supplementary Figure 63	4aa ins	593–639	
Error-prone DNA polymerase^a^	WP_046686430	Supplementary Figure 64	5aa ins	98–124	
TetR/AcrR family transcriptional regulator^a^	WP_083040593	Supplementary Figure 65	1aa ins	153–192	
TetR family transcriptional regulator^a^	WP_085129961	Supplementary Figure 66	1aa ins	157–199	
Hypothetical protein^a^	WP_083037591	Supplementary Figure 67	1aa del	47–92	
Amidohydrolase^a^	WP_083037811	Supplementary Figure 68	1aa del	4–32	
Carboxymuconolactone decarboxylase family protein^a^	WP_083037652	Supplementary Figure 69	1aa ins	1–29	
Polyketide cyclase^a^	WP_085128055	Supplementary Figure 70	3aa ins	107–152	
Spirocyclase AveC family protein^a^	WP_085128375	Supplementary Figure 71	1aa del	113–242	
Hypothetical protein^a^	WP_083036336	Supplementary Figure 72	3aa del	362–404	
TobH protein^a^	WP_083035918	Supplementary Figure 73	3aa ins	37–83	
UDP-N-acetylmuramate–L-alanine ligase	WP_083038184	Figure 9B Supplementary Figure 74	4aa ins	131–177	
DUF2236 domain-containing protein	WP_085129074	Supplementary Figure 75	1aa ins	145–190	
Cobaltochelatase subunit CobN	WP_083037938	Supplementary Figure 76	2aa ins	209–255	
Alpha/beta hydrolase	WP_083040154	Supplementary Figure 77	1aa ins	232–276	
Potassium transporter Kef	WP_083035846	Supplementary Figure 78	1aa ins	141–180	
Bifunctional tRNA (adenosine(37)-N6)-threonylcarbamoyltransferase I	WP_083040227	Supplementary Figure 79	1aa del	330–357	
Membrane protein	KKB98129	Supplementary Figure 80	1aa del	104–137	
DUF222 domain-containing protein	WP_083036231	Supplementary Figure 81	5aa ins	57–100	
MFS transporter	WP_083036343	Supplementary Figure 82	2aa del	232–267	
Adenylate/guanylate cyclase domain-containing protein	WP_083036337	Supplementary Figure 83	1aa ins	369–407	
DUF2029 domain-containing protein	WP_083037148	Supplementary Figure 84	3aa ins	154–187	
Hypothetical protein	WP_052618664	Supplementary Figure 35	1aa del	199–235	

a *Homologues of M. triviale and M. koreense were absent in BLASTp searches*.

Our BLASTp searches on the protein sequences from the genome of *M. sinense* JDM601 (Zhang et al., 2011) and *M. triviale* DSM 44153 (Fedrizzi et al., 2017) have also identified many CSPs whose homologs are found specifically in either members of the larger “*Terrae* complex” or uniquely by species which are part of either the “*Terrae*” clade or the “*Triviale*” clade. A summary of these CSPs is provided in Table 7. Of the identified CSPs, two CSPs (viz. accession numbers WP_013830140.1 and WP_013827845.1) are uniquely found in most members of the “*Terrae”* + “*Triviale”* clades. However, a large number of the other identified CSPs are specific for only either members of the “*Terrae”* clade (15 CSPs) or members of the “*Triviale*” clade (22 CSPs) and their homologs are not detected in other mycobacteria. Four of the CSPs specific for the “*Triviale*” clade included in Table 7 were also previously identified by Ngeow et al. (2015). The identification of a large number of CSPs, which are uniquely found in either all/most members of the “*Terrae”* clade or those from the “*Triviale*” clade again serve to clearly differentiate these two groups of mycobacteria and demarcate them in molecular terms.

**Table 7:** Summary of Conserved Signature Proteins (CSPs) that are specific for members of both “*Terrae”* + “*Triviale”* clades or only the “*Terrae”* clade or the “*Triviale”* clade.

**Gene or protein**	**Accession number**	**Function**	**Length**	**Specificity**
Hypothetical Protein	WP_013830140.1	Hypothetical	73	“*Terrae”* + “*Triviale”* Clades
Hypothetical protein	WP_013827845.1	Hypothetical	147	
Hypothetical Protein	WP_013828100.1	Hypothetical	267	“*Terrae”* Clade
CHAP domain-containing protein	WP_013830932.1	Amidase	209	
Hypothetical Protein	WP_013828443.1	Hypothetical	192	
Hypothetical Protein^a^	WP_013828919.1	MotB of proton channel complex MotA/MotB	159	
Hypothetical Protein	WP_013829267.1	Hypothetical	126	
Hypothetical Protein	WP_041317168.1	Hypothetical	172	
DUF732 domain-containing protein	WP_013827978.1	Hypothetical	179	
Hypothetical Protein^a^	WP_041318963.1	Hypothetical	231	
Hypothetical Protein	WP_013830185.1	Hypothetical	84	
Hypothetical Protein	WP_013828762.1	Hypothetical	1369	
Hypothetical Protein^a^	WP_013827315.1	Hypothetical	165	
Hypothetical Protein	WP_041318191.1	Hypothetical	69	
Hypothetical Protein^a^^,^^b^	WP_013829648.1	Glypican	207	
Hypothetical Protein^b^	WP_013829864.1	Hypothetical	131	
Hypothetical Protein^b^	WP_041317804.1	Hypothetical	133	
Hypothetical protein	WP_069390591.1	Hypothetical	199	“*Triviale”* Clade
Hypothetical protein	WP_069390644.1	Hypothetical	106	
Hypothetical protein	WP_069390667.1	Hypothetical	63	
Hypothetical protein	WP_069390717.1	Hypothetical	182	
Hypothetical protein	WP_069391089.1	Hypothetical	178	
Hypothetical protein	WP_069391367.1	PQQ enzyme repeat	152	
Hypothetical protein	WP_069391463.1	Hypothetical	68	
Hypothetical protein	WP_069391521.1	Hypothetical	441	
Hypothetical protein	WP_069391698.1	Hypothetical	63	
Hypothetical protein	WP_069391782.1	Hypothetical	180	
Hypothetical protein	WP_069391793.1	Hypothetical	188	
Hypothetical protein	WP_069392105.1	Hypothetical	129	
Hypothetical protein	WP_069392126.1	NT_Pol-beta-like Superfamily	272	
Hypothetical protein	WP_069392251.1	Hypothetical	319	
Hypothetical protein	WP_069392420.1	Hypothetical	116	
Hypothetical protein	WP_069392510.1	Hypothetical	71	
Hypothetical protein	WP_069392884.1	Hypothetical	126	
Hypothetical protein	WP_069392982.1	Hypothetical	79	
Hypothetical protein	WP_069392983.1	Hypothetical	104	
Hypothetical protein	WP_069393100.1	Hypothetical	105	
Hypothetical protein	WP_069393493.1	Hypothetical	128	
Hypothetical protein	WP_069393844.1	Hypothetical	128	

a *Previously also identified by Ngeow et al. (2015)*.

b *Some exceptions are present*.

## Discussion

**Figure 10:**
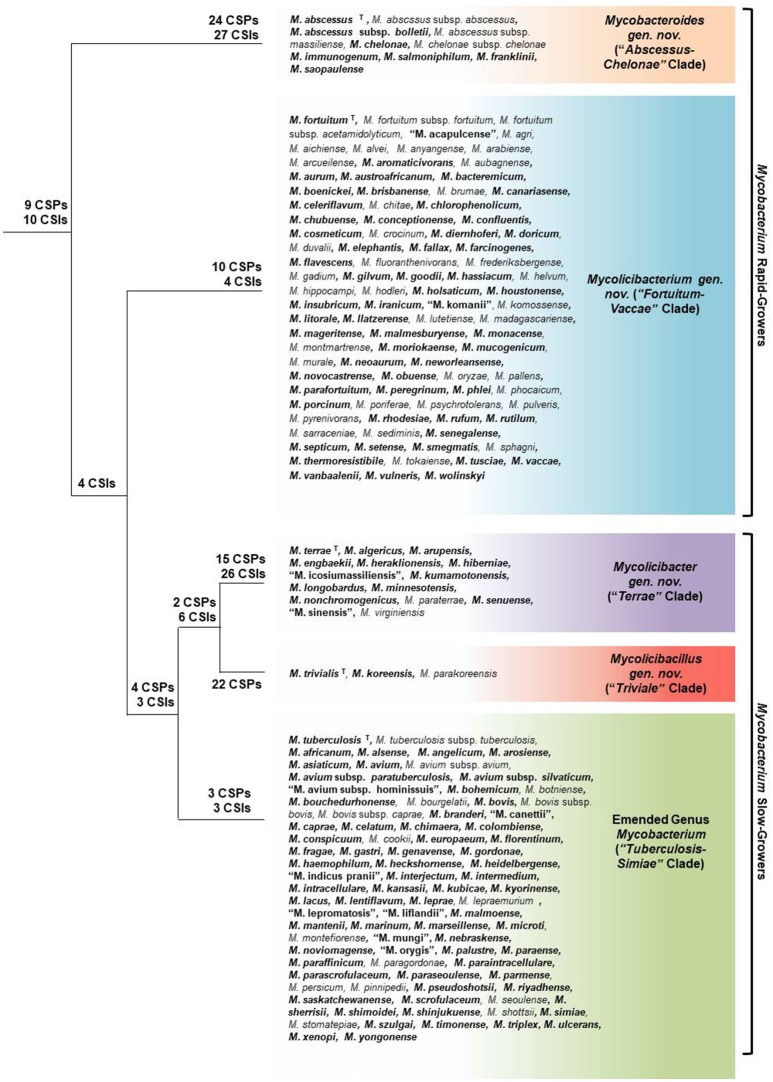
A summary diagram depicting the overall relationships among the major groups of mycobacterial species. The numbers of identified CSIs and CSPs, which are specific for different clades are marked on the nodes. The names of the five main clades of mycobacterial species identified in this work, viz. “*Tuberculosis-Simiae*,” “*Terrae,”* “*Triviale*,” “*Fortuitum-Vaccae*,” and “*Abscessus-Chelonae*”, along with their proposed or emended names and the species which are part of these clades are marked. Species which have had their genomes analyzed in this study are bolded. The superscript letter T beside a species indicates that it is the type species of the genus. The placements of other mycobacterial species, whose genomes have not been sequenced into these clades are based on their branching in the 16S rRNA tree (Supplmentary Figure 2). The species whose names are not italicized and are placed within quotation marks have not yet been validly published.

The genus *Mycobacterium* comprises a large group of species (currently 188 species have validly published names), which includes some of the most impactful human pathogens (viz. *M. tuberculosis* and *M. leprae*) as well as large numbers of species found in diverse environments (Magee and Ward, 2012; Lory, 2014). In view of the immense clinical importance of certain *Mycobacterium* species, it is of much interest to have a reliable understanding as to how different species within this large group are related (Tsukamura, 1967a; Rogall et al., 1990; Stahl and Urbance, 1990; Goodfellow and Magee, 1998; Magee and Ward, 2012; Tortoli, 2012; Lory, 2014). However, despite much work (reviewed in Introduction), all known mycobacterial species are currently part of a single genus and their interrelationships are generally poorly understood (Magee and Ward, 2012; Tortoli, 2012; Lory, 2014; Fedrizzi et al., 2017). Genome sequences are now available for 150 of the 188 known mycobacterial species providing a unique opportunity for reliably understanding the relationships among the *Mycobacterium* species through genomic approaches. Using genome sequences, comprehensive phylogenetic and comparative genome analyses were carried out on *Mycobacterium* species using multiple independent approaches. In the first approach, phylogenomic trees were constructed for *Mycobacterium* species based on several large datasets of protein sequences including 1941 core proteins for the genus *Mycobacterium*, 136 core proteins for the phylum Actinobacteria, and another set of 8 highly conserved essential proteins found in all mycobacteria. Based on the core proteins in mycobacterial genomes, pairwise amino acid identity was also determined amongst different *Mycobacterium* species, providing a measure of the overall genetic relatedness of the species. In the third approach, exhaustive comparative genomic analyses were carried out on protein sequences of mycobacterial genomes to identify highly specific markers in the forms of CSIs and CSPs that are distinctive characteristics of the genus *Mycobacterium* as a whole or of different major clades within this genus. The results from all of these comprehensive genomic approaches reveal a consistent picture of the overall evolutionary relationships among the mycobacterial species, a summary of which is presented in Figure 10.

In phylogenetic trees constructed based on different large datasets of protein sequences, the *Mycobacterium* consistently grouped into four main strongly supported clades at the highest level. Within the larger “*Terrae* complex,” the species *M. triviale* and *M. koreense* also consistently formed a deeper branching “*Triviale*” clade. The existence of these five clades is also supported by the high degree of genome relatedness amongst the members of each clade, as indicated by the results of average amino acid identity analysis. More importantly, our analyses of protein sequences from *Mycobacterium* species have resulted in the identification of a total of 172 novel molecular markers (CSIs and CSPs) that are distinctive characteristics of either the entire genus *Mycobacterium* or of the five clades identified within this genus at various phylogenetic levels. A graphical schematic of the identified molecular markers and the mycobacterial clades for which they are specific for is shown in Figure 10. Thus, the existence as well as the distinctness of the five main clades within the genus *Mycobacterium* is supported not only by comprehensive phylogenomic studies and by genome relatedness analysis, but also by the identification of large numbers of highly specific molecular markers, which serve to clearly demarcate these clades. Although it is difficult to specify how many characters are sufficient to divide a given taxon into more than one group, as this will depend upon the genetic diversity as well as phylogenetic depth of a taxon, in cases where the monophyly and distinctness of the described clades are strongly supported by multiple genome-scale phylogenetic trees as well as other independent approaches (e.g., AAI or ANI analysis), even 1–2 reliable molecular characters such as the CSIs and CSPs are sufficient for separation of a given group into distinct taxa (Gao and Gupta, 2012; Bhandari et al., 2013; Gupta et al., 2013a,b, 2015; Adeolu and Gupta, 2014; Bhandari and Gupta, 2014; Sawana et al., 2014; Adeolu et al., 2016; Alnajar and Gupta, 2017; Barbour et al., 2017).

It should be noted that molecular markers such as CSIs and CSPs represent synapomorphic characteristics and they provide important means for reliable identification/demarcation of different monophyletic clades of organisms (Baldauf and Palmer, 1993; Gupta, 1998, 2016b; Rokas and Holland, 2000; Dutilh et al., 2008; Chandra and Chater, 2014). Extensive earlier work on these markers show that they are highly reliable characteristics of different groups of organisms and species as relationships based on them are generally not affected by factors such as differences in evolutionary rates or lateral gene transfers (Bhandari et al., 2012; Gupta, 2014, 2016a,b). Further, each of these CSIs or CSPs, which are present in different genes/proteins, provide independent evidence supporting the monophyletic nature of the different identified clades, as well as providing novel and reliable means for the demarcation as well as diagnostics of species from these clades of bacteria (Ahmod et al., 2011; Wong et al., 2014). Extensive earlier work on CSIs/CSPs provides evidence that both large as well as small CSIs (even a one amino acid insert/deletion in protein sequence results from an in frame three nucleotides insertion/deletion within a conserved region) and CSPs provide reliable molecular markers for taxonomic and diagnostic studies, and they also exhibit a high degree of predictive ability to be present in other members of the indicated groups for which sequence information is lacking at present (Gao and Gupta, 2012; Adeolu and Gupta, 2014; Naushad et al., 2014; Sawana et al., 2014; Adeolu et al., 2016; Gupta, 2016b; Alnajar and Gupta, 2017). As noted earlier, some of the CSIs and CSPs specific for the genus *Mycobacterium* were identified when the sequence information was available for a limited number of mycobacterial genomes (Gao and Gupta, 2005, 2012; Gao et al., 2006). However, despite the large increase in the number of mycobacterial genomes, many of these CSIs and CSPs are still found to be specific for this genus. In view of their demonstrated specificity and reliability for the indicated group of organisms, the CSIs and CSPs in recent years have been used extensively for important taxonomic changes to a number of prokaryotic groups at various phylogenetic levels ranging from description of new classes, orders, families and genera including division of the original *Burkholderia, Borrelia* and *Thermotoga* genera into two or more genera (Gao and Gupta, 2012; Bhandari et al., 2013; Gupta et al., 2013a,b, 2015; Adeolu and Gupta, 2014; Bhandari and Gupta, 2014; Sawana et al., 2014; Adeolu et al., 2016; Alnajar and Gupta, 2017; Barbour et al., 2017).

It should be noted that a 12–14 nucleotide insert in the 16S rRNA sequences (in helix 18 between positions 451 and 482 in the *E. coli* sequence) is often used as a marker to differentiate between rapid-growing and slow-growing mycobacteria (Pitulle et al., 1992; Hartmans et al., 2006; Tortoli, 2012, 2014; Fedrizzi et al., 2017). The presence and absence of this insert in different sequenced mycobacterial species has been examined by us and this information is presented in Supplementary Figure 85. This insert, due to its presence in a conserved region, *also represents a CSI*. However, in contrast to the large numbers of CSIs described in this work, which are of fixed lengths and highly-specific characteristics of the described clades, this insert is of variable length (9-14 aa insertion) and it is lacking in many members of the slow-growing mycobacteria or the “*Tuberculosis-Simiae*” clade (Hartmans et al., 2006; Tortoli, 2012, 2014). Thus, unlike the different CSIs identified in the present work, this insert in the 16S RNA is not a distinguishing characteristic of either all slow-growing *Mycobacterium* species (i.e., “*Tuberculosis-Simiae”* + “*Terrae”* + “*Triviale”* clades) or of the “*Tuberculosis-Simiae*” clade. However, all of the species belonging to the “*Terrae*” clade contain a 14 nucleotide insert in this position, which provides a signature CSI for this clade, similar to the large numbers of other CSIs and CSPs reported here (see Figure 9, Tables 6, 7). In contrast to the molecular markers described here, which are discrete and highly specific characteristics of the different indicated clades of mycobacteria, other physical and chemotaxonomic characteristics described in literature for various groups of mycobacteria are not specific for the indicated groups (see Supplementary Table 3; Magee and Ward, 2012). The presence or absence of the described physical and chemotaxonomic characteristics is often based on subjective criteria and information for such characteristics is not available for large numbers of mycobacterial species (Magee and Ward, 2012). This makes it difficult to reliably ascertain the potential usefulness of such characteristics as reliable markers for any particular group of mycobacteria.

The results presented here also strongly indicate that the “*Abscessus-Chelonae”* clade comprises the earliest branching lineage within the genus *Mycobacterium*. Its early divergence within the genus *Mycobacterium* is strongly supported by phylogenetic studies and multiple identified CSIs which are commonly shared by all or most *Mycobacterium* species, but absent in this clade of species. The deeper branching of the “*Abscessus-Chelonae”* clade as well as the “*Fortuitum-Vaccae*” clade of fast-growing mycobacteria, in comparison to the clades of slow-growing mycobacteria, supports the inference from earlier work that the rapid-growing mycobacterial species are ancestral and the slow-growers have evolved from them (Pitulle et al., 1992; Hartmans et al., 2006; Magee and Ward, 2012; Tortoli, 2012, 2014; Fedrizzi et al., 2017). Another important inference from the present work is that while the two clades of slow-growing mycobacteria (i.e., “*Tuberculosis-Simiae*” and the larger “*Terrae* + *Triviale*” clade) group together in phylogenetic trees, the grouping together of the two clades of rapid-growing mycobacteria is not observed in any phylogenetic trees. Further, while in our work 3 CSIs and 4 CSPs were identified that are commonly shared by members of the “*Tuberculosis-Simiae*” clade plus the “*Terrae”* + “*Triviale*” clade, no molecular marker was identified that is uniquely shared by the “*Abscessus-Chelonae”* and “*Fortuitum-Vaccae*” clades. It should be noted that while the distribution of most *Mycobacterium* species into the clades of slow-growing and fast-growing bacteria is generally in concordance with their rate of growth (Hartmans et al., 2006; Magee and Ward, 2012; Fedrizzi et al., 2017), a few exceptions are observed in this regard. In particular, the species *M. doricum, M. vulneris* and *M. tusciae*, which are slow-growing mycobacterial species (Magee and Ward, 2012; Fedrizzi et al., 2017), consistently branch within the “*Fortuitum-Vaccae*” clade of fast-growing mycobacteria. These species are also found to share the molecular signatures specific for the “*Fortuitum-Vaccae*” clade, but they lack the signatures for the slow-growing clades of mycobacteria. The anomalous branching of *M. doricum* and *M. tusciae* with the rapid-growing mycobacteria has also been reported in earlier work (Magee and Ward, 2012; Fedrizzi et al., 2017). This observation in conjunction with our results showing that both the slow-growing and fast-growing *Mycobacterium* species form at least two distinct clades, and that the rapidly-growing species do not form a monophyletic lineage, indicates that the differentiation of the *Mycobacterium* species based solely on their growth rate is of limited use for developing a coherent taxonomic framework that is consistent with genomic and phylogenetic characteristics.

Of the main clades of mycobacteria described here, the “*Terrae*” + “*Triviale*” and the “*Abscessus-Chelonae”* clades are recognized from earlier phylogenetic studies (Adékambi and Drancourt, 2004; Mignard and Flandrois, 2008; Tortoli, 2012, 2014; Fedrizzi et al., 2017; Wee et al., 2017). In the present work, distinctness of the “*Abscessus-Chelonae”* clade is established by 51 molecular markers (CSIs and CSPs) which are specific for this clade. Although our work has identified some molecular markers that are specific for the larger “*Terrae”* + “*Triviale*” clade, our results strongly indicate that the species from the “*Triviale*” clade are phylogenetically and molecularly distinct from those of the “*Terrae*” clade. The distinctness of these two clades is also strongly supported by larger numbers of molecular markers identified in our work that are uniquely shared by the members of either the “*Terrae*” clade or the “*Triviale*” clades. The “*Terrae*” clade is also distinguished from others by the presence of a 14 nucleotide insertion in the helix 18 of the 16S rRNA gene (Tortoli, 2012, 2014; Ngeow et al., 2015). The other two main clades of mycobacteria described here namely the “*Tuberculosis-Simiae*” clade and the “*Fortuitum-Vaccae*” clade, harbor >85% of the known *Mycobacterium* species and no molecular markers or other characteristics specific for these clades are known from earlier work. However, both these large clades of mycobacteria can now be reliably demarcated on the basis of multiple highly-specific molecular signatures. In addition to the five clades described here, a number of other smaller clades are observed in the phylogenetic trees (Figure 1 and Supplementary Figure 1). However, the work on characterization of these smaller subclades could be undertaken in future studies.

The work presented here based on multiple lines of evidence provide compelling support that the species from the genus *Mycobacterium* are comprised of five phylogenetically coherent clades, which can now be robustly distinguished from each other based on their branching in phylogenomic trees and multiple highly specific molecular signatures (Figure 10). These results provide a strong phylogenetic and genomic framework for division of the existing genus *Mycobacterium* into five distinct genera, corresponding to the five main clades described here. On the basis of the presented results, we are proposing that the genus *Mycobacterium* should be emended to include only members of the “*Tuberculosis-Simiae*” clade, which includes *Mycobacterium tuberculosis*, the type species of the genus (Zopf, 1883; Lehmann and Neumann, 1896), (Approved Lists, 1980; Skerman et al., 1980). The species from the other four main clades “*Fortuitum-Vaccae”, “Terrae”, “Triviale”* and “*Abscessus-Chelonae”* are transferred to four new genera with the following proposed names, *Mycolicibacterium* gen. nov., *Mycolicibacter* gen. nov., *Mycolicibacillus* gen. nov. and *Mycobacteroides* gen. nov., respectively. In the proposed classification, all of the major human pathogens are retained within the emended genus *Mycobacterium*, whereas the genus *Mycolicibacterium* is primarily comprised of environmental species. Most members of the proposed genera *Mycolicibacter* and *Mycolicibacillus* are also non-pathogenic, except occasional association of some species with animal hosts or human patients (Tasler and Hartley, 1981; Smith et al., 2000; Tortoli, 2014). Some members from the proposed genus *Mycobacteroides* are known to be associated with lung, skin and soft tissue infections (Simmon et al., 2011; Magee and Ward, 2012; Tortoli, 2014), however, none of them are considered as major life-threatening pathogens (Magee and Ward, 2012; Tortoli, 2014). Nonetheless, all five of these genera will remain part of the family *Mycobacteriaceae* and their proposed names bear close similarity to the original genus name *Mycobacterium*. Thus, all of them can still be referred to as mycobacterial species or as *M*. (*species name*), causing minimum confusion with any other species.

The proposed division of the existing genus *Mycobacterium* into the five proposed genera will have many benefits in terms of understanding and clarifying the relationships among the known mycobacterial species. The proposed division clearly separates the major human and animal pathogenic species, which are now part of the emended genus *Mycobacterium*, from all other (i.e., a majority of) mycobacterial species, which are either non-pathogenic or are of lesser clinical significance. With the explicit division of the mycobacterial species into these groups, attention can now be focused on unique genetic and molecular characteristics that differentiate the members of these groups of microbes. For each of these proposed genera, multiple CSIs and CSPs that are specific for these groups have been identified. Based on these molecular markers, it should be possible to develop novel and more reliable diagnostic methods for the identification of members of these groups by either *in silico* analysis of genomic sequences (based on BLASTp searches examining the presence or absence of these molecular sequences) or by experimental means utilizing PCR-based assays (Ahmod et al., 2011; Wong et al., 2014). Further, although the cellular functions of most of the identified CSIs or CSPs are not known, earlier work on other CSIs/CSPs has shown that these molecular characteristics are essential or play important functional roles in the organisms where they are found (Singh and Gupta, 2009; Schoeffler et al., 2010; Chandra and Chater, 2014; Gupta, 2016c). For example, some of the CSPs which are specific for the slow-growing mycobacterial species belong to the PE or PPE family of proteins, which play a role in virulence determination (Mukhopadhyay and Balaji, 2011). Hence, further functional investigations on the identified CSIs/CSPs are expected to lead to discovery of novel biochemical and/or other properties that are specific for either the entire *Mycobacteriaceae* family or for members of different genera that are part of this family.

The descriptions of the emended family *Mycobacteriaceae*, the emended genus *Mycobacterium* and of the four newly proposed genera viz, *Mycolicibacter* gen. nov., *Mycobacteroides* gen. nov., *Mycolicibacillus* gen. nov. and *Mycolicibacterium* gen. nov. are given below. Brief descriptions of the new species names combinations as well as some new species names resulting from the proposed taxonomic changes are also given below.

### Emended description of the family *Mycobacteriaceae* chester 1897 (approved lists 1980) (Skerman et al., 1980)

*Mycobacteriaceae* (My.co.bac.te.ri.a.ce´ae. N.L. neut. n. *Mycobacterium* type genus of the family; suff. *-aceae* ending to denote a family; N.L. fem. pl. n. *Mycobacteriaceae* the *Mycobacterium* family).

The family *Mycobacteriaceae* contains the type genus *Mycobacterium* as well as the genera *Mycolicibacter* gen. nov., *Mycolicibacterium* gen. nov., *Mycolicibacillus* gen nov., and *Mycobacteroides* gen. nov. Additionally, the genus *Amycolicoccus* is also indicated to be a part of this family (Wang et al., 2010; Parte, 2014). However, the sole type species of this genus, *Amycolicoccus subflavus*, is now reclassified as *Hoyosella subflava* (Hamada et al., 2016). The general characteristics of the family *Mycobacteriaceae* are as described by Magee and Ward (2012) for the genus *Mycobacterium*. The members of this family are aerobic to microaerophilic, slightly curved or straight rods (0.2–0.6 × 1.0–10 μm), which are acid–alcohol-fast at some stage of growth. Difficult to stain by Gram's-method, but are usually considered Gram-stain-positive. Some species may exhibit filamentous or mycelium-like growth. Cells are nonmotile and asporogenous. Colonies may be white- to cream-colored; some strains produce yellow- or orange-pigmented colonies with or without light stimulation. Whole-organism hydrolysates are rich in *meso*-diaminopimelic acid, arabinose, and galactose. The peptidoglycan is of the A1g type. Muramic acid moieties are *N*-glycolated. Cells and cell walls are rich in lipids. These include waxes which have characteristic, chloroform-soluble, mycolic acids with long (60–90 carbon atoms) branched chains. The fatty acid esters released on pyrolysis MS of mycolic acid esters have 22–26 carbon atoms. Cells contain diphosphatidylglycerol, phosphatidyl-ethanolamine, phosphatidylinositol, and phospatidylinositol mannosides as predominant polar lipids, straight-chain saturated, unsaturated, and 10-methyloctadecanoic (tuberculostearic) fatty acids as major fatty acid components, and dihydrogenated menaquinones with nine isoprene units as the predominant isoprenolog. The family includes obligate parasites, saprophytes, and opportunistic forms. The G+C content of genome-sequenced species varies from 57 to 71 (mol %) and genome size ranges from 3.1 to 10.5 Mbp. The members of the family *Mycobacteriaceae* form a distinct clade in the 16S rRNA tree and they are distinguished from all other members of the order *Corynebacteriales* by their unique shared presence of conserved signature indels described in this work (Table 1) in the following 10 proteins (viz. serine hydrolase, precorrin-4 C(11)-methyltransferase, NAD(P)H-quinone dehydrogenase, orotidine 5′-phosphate decarboxylase, deoxyribonuclease IV, peptidase C69, SGNH/GDSL hydrolase family protein, succinate dehydrogenase, N-dimethylarginine dimethylaminohydrolase, ergothioneine biosynthesis protein EgtB). Additionally, the homologs of the following nine proteins (accession numbers are in parenthesis) are also uniquely found in members of the family *Mycobacteriaceae* viz. hypothetical protein (WP_011723520.1), hypothetical protein (WP_011723901.1), MAV_11221(WP_011723955.1), membrane protein (WP_011724283.1), PE-PPE domain-containing protein (WP_011724324.1), DUF2561 domain-containing protein (WP_011724709.1), Membrane protein (WP_009976570.1), hypothetical protein (WP_003876314.1) and hypothetical protein (WP_003874755.1) (see Table 2 in this work).

### Emended description of the genus *Mycobacterium* Lehmann and Neuman 1896 (approved lists 1980) (Skerman et al., 1980)

*Mycobacterium* (My.co.bac.te´ri.um. Gr. n. *mykes* a fungus; N.L. neut. n. *bacterium*, a small rod; N.L. neut. n. *Mycobacterium*, a fungus rodlet).

The type species is *Mycobacterium tuberculosis* (Zopf 1883) Lehmann and Neumann 1896 (Approved Lists 1980) (Skerman et al., 1980).

Members of this genus whose are slow-growing bacteria requiring at least 7 days of incubation at optimal temperatures to form colonies. Several species are obligate parasites of human and animals and the genus harbors a number of important human (e.g., *Mycobacterium tuberculosis, M. leprae, M. ulcerans*) and animal (e.g., *Mycobacterium bovis*) pathogens. Other phenotypic and chemotaxonomic characteristics of this genus are similar to that for the family *Mycobacteriaceae*.

Some species from this clade contain a 9–12 nucleotide long insert in helix 18 of the 16S rRNA gene sequence (Supplementary Figure 85; Hartmans et al., 2006; Tortoli, 2014). Species are indicated to generally lack the *LivFGMH* operon and the *shaACDEFG* cluster of genes, which encodes respectively for proteins allowing the transportation of leucine, isoleucine and valine into the bacteria and a Na^+^/H^+^ antiporter that is important for the homeostasis of Na^+^ and H^+^ (Wee et al., 2017). Presence of the components of Type VII secretion system has been reported in members of this genus (Wee et al., 2017). The members of this genus form a monophyletic clade in phylogenetic trees constructed based on 16S rRNA gene sequences as well as multiple large datasets of protein sequences described in this work including a tree based on 1941 core mycobacterial proteins, a tree based on 136 core proteins for the phylum Actinobacteria, and a tree based on concatenated sequences for eight conserved housekeeping proteins (viz. RpoA, RpoB, RpoC, GyrA, GyrB, Hsp65, EF-Tu, and RecA). Members of the genus *Mycobacterium* can be clearly distinguished from other genera within the *Mycobacteriaceae* family based on conserved signature indels described in this study (Table 4) in the following three proteins, a hypothetical protein, aldehyde dehydrogenase family protein and 23S rRNA (guanosine(2251)-2′-O)-methyltransferase, that are uniquely shared by the members of this genus. In addition, the homologs of the following three proteins (accession numbers are in parenthesis): a histone-like protein HNS (NP_218369.1), a hypothetical protein Rv4010 (YP_004837050.1) and a membrane protein (NP_217322.1), are also unique characteristics of the members of this genus.

The G-C content and genome sizes of the member species ranges from 57.8–69.3 (mol %) to 3.2–7.3 Mbp, respectively.

### Description of *Mycolicibacter* gen. nov.

*Mycolicibacter* (My.co.li.ci.bac´ter. N.L. n. *acidum mycolicum*, mycolic acid; N.L. masc. n. *bacter*, rod; N.L. masc. n. *Mycolicibacter*, a genus of mycolic acid containing rod-shaped bacteria).

The type species is *Mycolicibacter terrae*.

The members of the genus *Mycolicibacter* are commonly referred to as the *M. terrae* complex. This genus contains species that are slow-growing (more than 7 days) and nonchromogenic with some species that show intermediate growth duration (5–15 days) (Tortoli, 2014; Ngeow et al., 2015). In phylogenetic trees, the *Mycolicibacter* clade forms a sister clade to a clade comprising of the genus *Mycobacterium*, harboring other slow-growing mycobacteria. Most members of this genus are non-pathogenic, but some species have been isolated from animal hosts (Tasler and Hartley, 1981) and human patients (Smith et al., 2000). Multiple antibiotic resistance has been reported for many of the isolates (Milne et al., 2009; Zhang et al., 2013b).

The members of this genus form a monophyletic clade in phylogenetic trees based on 16S rRNA gene sequences as well as multiple datasets of gene/protein sequences including a tree based on 1941 core mycobacteria proteins and a tree based on 136 core proteins for the phylum Actinobacteria. The members of the genus *Mycolicibacter* exhibit a closer relationship to members of the genus *Mycolicibacillus* in phylogenetic trees, which is also supported by a number of CSIs listed (Table 6) in the proteins ATP-dependent helicase, PDZ domain-containing protein, Ferredoxin reductase, DUF2236 domain-containing protein and two hypothetical protein with the accession number WP_083040170 and DUF4185 domain-containing protein, as well as 2 CSPs (viz. accession numbers WP_013830140.1 and WP_013827845.1) that are commonly shared by the members from these two genera. All of the species from this genus contain a 14 nucleotide insertion in the helix 18 of the 16S rRNA gene (Supplementary Figure 85; Tortoli, 2014). Additionally, the members of this genus are distinguished from members of all other genera within the family *Mycobacteriaceae* due to their possession of 26 conserved signature indels described in this study (Table 6) present in the following proteins, non-ribosomal peptide synthetase, nucleoside hydrolase, three different indels in TetR family transcriptional regulator, carbon starvation protein A, error-prone DNA polymerase, amidohydrolase, carboxymunconolacton decarboxylase family protein, polyketide cyclase, spirocyclase AveC family protein, TobH protein, UDP-N-acetylmuramate–L-alanine ligase, DUF2236 domain-containing protein, cobaltochelatase subunit CobN, alpha/beta hydrolase, potassium transporter Kef, bifunctional tRNA (adenosine(37)-N6)-threonylcarbamoyltransferase complex dimerization subunit Type 1 TsaB/ribosomal protein alanine acetyltransferase RimI, a membrane protein, DUF222 domain-containing protein, MFS transporter, adenylate/guanylate cyclase domain-containing protein, DUF2029 domain-containing protein and the following hypothetical proteins with the accession numbers (WP_083037591, WP_083040170, WP_083036336 and WP_052618664), that are uniquely found in the members of this genus. In addition, the homologs of the 17 conserved signature proteins, whose accession numbers are as follows (viz. WP_013830140.1, WP_013827845.1, WP_013828100.1, WP_013830932.1, WP_013828443.1, WP_013828919.1, WP_013829267.1, WP_041317168.1, WP_013827978.1, WP_041318963.1, WP_013830185.1, WP_013828762.1, WP_013827315.1, WP_041318191.1, WP_013829648.1, WP_013829864.1, and WP_041317804.1) are also distinctive characteristics of either all or most members of this genus (Table 7).

The members of the genus *Mycolicibacter* are characterized by high G-C content (66.3–70.3 mol %) and they have relatively short genomes (range 3.87–5.11 Mbp).

The description of *Mycolicibacter terrae* comb. nov. as well as the descriptions of new name combinations for other species which are part of the genus *Mycolicibacter* are provided in Table 8.

**Table 8:** Descriptions of new name combinations for species in the genus *Mycolicibacter*.

**New name combinations**	**Description and type strain**
*Mycolicibacter terrae* comb. nov. (ter´rae. L. gen. n. *terrae*, of the earth)	Basonym: *Mycobacterium terrae* Wayne 1966 (Approved Lists 1980) (Skerman et al., 1980) The description of this taxon is as given by Wayne (1966). The type strain is ATCC 15755 = CCUG 27847 = CIP 104321 = DSM 43227 = JCM 12143 = LMG 10394.
*Mycolicibacter algericus* comb. nov. (al.ge´ri.cus. N.L. masc. adj. *algericus*, of or pertaining to Algeria, the country where the strain was first isolated)	Basonym: *Mycobacterium algericum* Sahraoui et al., 2011 The description of this taxon is as given by Sahraoui et al. (2011). The type strain is TBE 500028/10 = Bejaia = CIP 110121 = DSM 45454.
*Mycolicibacter arupensis* comb. nov. (a.rup.en´sis. N.L. masc. adj. *arupensis*, pertaining to the ARUP Institute for Clinical and Experimental Pathology, where the type strain was characterized)	Basonym: *Mycobacterium arupense* Cloud et al., 2006 The description of this taxon is as given by Cloud et al. (2006). The type strain is AR30097 = ATCC BAA-1242 = DSM 44942.
*Mycolicibacter engbaekii* comb. nov. (eng.bae´ki.i. N.L. gen. masc. n. *engbaekii*, of Engbaek, to honour of the Danish mycobacteriologist H. C. Engbaek)	Basonym: *Mycobacterium engbaekii* Tortoli et al., 2013 The description of this taxon is as given by Tortoli et al. (2013). The type strain is ATCC 27353 = DSM 45694.
*Mycolicibacter heraklionensis* comb. nov. (he.ra.kli.on.en´sis N.L. masc. adj. *heraklionensis* from Heraklion the city in Crete island where many such strains were isolated)	Basonym: *Mycobacterium heraklionense* Tortoli et al., 2013 The description of this taxon is as given by Tortoli et al. (2013). The type strain is GN-1 = CECT 7509 = LMG 24735 = NCTC 13432.
*Mycolicibacter hiberniae* comb. nov. (hi.ber´ni.ae. L. gen. n. *hiberniae*, of *Hibernia*, the Latin name for Ireland, the source of the strains)	Basonym: *Mycobacterium hiberniae* Kazda et al., 1993. The description of this taxon is as given by Kazda et al. (1993). The type strain is Hi 11 = ATCC 49874 = CIP 104537 = DSM 44241 = JCM 13571.
*Mycolicibacter kumamotonensis* comb. nov. (ku.ma.mo.to.nen´sis. N.L. masc. adj. *kumamotonensis*, of or pertaining to Kumamoto Prefecture in Japan, where the type strain was isolated)	Basonym: *Mycobacterium kumamotonense* Masaki et al., 2007 The description of this taxon is as given by Masaki et al. (2006, 2007). The type strain is CST 7247 = CCUG 51961 = JCM 13453.
*Mycolicibacter longobardus* comb. nov. (lon.go.bar´dus. N.L. masc. adj. *longobardus*, of or pertaining to Lombardy, the region where the strains were isolated)	Basonym: *Mycobacterium longobardum* Tortoli et al., 2013 The description of this taxon is as given by Tortoli et al. (2013). The type strain is FI-07034 = CCUG 58460 = DSM 45394.
*Mycolicibacter minnesotensis* comb. nov. (min.ne.so.ten´sis. N.L. masc. adj. *minnesotensis*, of or belonging to Minnesota)	Basonym: *Mycobacterium minnesotense* Hannigan et al., 2013 The description of this taxon is as given by Hannigan et al. (2013). The type strain is DL49 = DSM 45633 = JCM 17932 = NCCB 100399.
*Mycolicibacter nonchromogenicus* comb. nov. (non.chro.mo.ge´ni.cus. L. adv. *non*, not; Gr. n. *chroma*, color; Gr. v. *gennaio*, to produce; L. masc. suff. -*icus*, suffix used with the sense of pertaining to; N.L. masc. adj. *nonchromogenicus*, intended to mean not producing color)	Basonym: *Mycobacterium nonchromogenicum* Tsukamura 1965 (Approved Lists 1980) (Skerman et al., 1980) The description of this taxon is as given by Tsukamura (1965a). The type strain is ATCC 19530 = CCUG 28009 = CIP 106811 = DSM 44164 = JCM 6364 = NCTC 10424.
*Mycolicibacter paraterrae* comb. nov. (pa.ra.ter´rae. Gr. prep. *para* beside; *terrae* of the earth; N.L. gen. n. *paraterrae* specific epithet of a *Mycobacterium* species; N.L. gen. n. *paraterrae* a species similar to members of the *Mycobacterium terrae* complex)	Basonym: *Mycobacterium paraterrae* Lee et al., 2016 The description of this taxon is as given by Lee et al. (2010, 2016). The type strain is 05-2522 = DSM 45127 = KCTC 19556.
*Mycolicibacter senuensis* comb. nov. (se.nu.en´sis. N.L. masc. adj. *senuensis*, arbitrary name formed from the initial letters of Seoul National University, the organization that carried out the taxonomic investigation of the type strain)	Basonym: *Mycobacterium senuense* Mun et al., 2008 The description of this taxon is as given by Mun et al. (2008). The type strain is 05-832 = DSM 44999 = JCM 16017 = KCTC 19147.
*Mycolicibacter virginiensis* comb. nov. (vir.gi.ni.en´sis. N.L. masc. adj. *virginiensis* referring to the geographic location of the first recognized case)	Basonym: *Mycobacterium virginiense* Vasireddy et al., 2017 The description of this taxon is as given by Vasireddy et al. (2016, 2017). The type strain is MO-233 = DSM 100883 = CIP 110918.

In addition to the new name combinations for species which are part of the genus *Mycolicibacter*, we also provide below description of two new species that should also be placed in the genus *Mycolicibacter*.

**Description of *Mycolicibacter icosiumassiliensis* sp. nov**. (i.co.si.u.mas.si.li.en´sis; L. masc. n. *icosiumassiliensis*, from the combination of Icosium, the Latin name of Algiers where the strain was first isolated and Massilia, the Latin name of Marseille, where the strain was described).

The description of this taxon is as given by Djouadi et al. (2016) for “*Mycobacterium icosiumassilensis”*. The type strain is 8WA6 (= CSUR P1561 = DSM 100711).

**Description of *Mycolicibacter sinensis* sp. nov**. (sin.en´sis. N.L. masc. adj. *sinensis* means “belonging to China,” indicating the source of the type strain).

The description of this taxon is as given by Zhang et al. (2013b) for “*Mycobacterium sinense”*. The type strain is JDM601.

### Description of *Mycolicibacillus* gen. nov.

*Mycolicibacillus* (My.co.li.ci.ba.cil´lus. N.L. n. *acidum mycolicum*, mycolic acid; L. masc. n. *bacillus*, a small staff or rod; N.L. masc. n. *Mycolicibacillus*, a genus of mycolic acid containing rod-shaped bacteria).

The type species is *Mycolicibacillus trivialis*.

The genus *Mycolicibacillus* is comprised of slow-growing nonchromogenic bacteria requiring more than 7 days of incubation at optimal temperatures to form colonies. In phylogenetic trees, members of this genus form a deep-branching distinct clade that is most closely related to members of the genus *Mycolicibacter*. A close relationship of the species from the genera *Mycolicibacillus* and *Mycolicibacter* is also supported by a number of CSIs listed in Table 6 in the proteins ATP-dependent helicase, PDZ domain-containing protein, ferredoxin reductase, DUF2236 domain-containing protein, non-ribosomal peptide synthetase, hypothetical protein with accession number WP_083040170 and DUF4185 domain-containing protein and CSPs listed in Table 7 (viz. accession numbers WP_013830140.1 and WP_013827845.1) that are commonly shared by these two groups of bacteria. Unlike members of the genus *Mycolicibacter*, which contain a 14 nucleotide insertion in the helix 18 of the 16S rRNA gene, members of the genus *Mycolicibacillus* lack an insertion in this position (Tortoli, 2014) (Supplementary Figure 85). In addition, the homologs showing significant sequence similarity for the 22 proteins listed in Table 6 with the accession numbers WP_069390591.1, WP_069390644.1, WP_069390667.1, WP_069390717.1, WP_069391089.1, WP_069391367.1, WP_069391463.1, WP_069391521.1, WP_069391698.1, WP_069391782.1, WP_069391793.1, WP_069392105.1, WP_069392126.1, WP_069392251.1, WP_069392420.1, WP_069392510.1, WP_069392884.1, WP_069392982.1, WP_069392983.1, WP_069393100.1, WP_069393493.1, and WP_069393844.1, are uniquely present in members of this genus. This genus presently contains only three species (*M. trivialis*, *M. koreensis* and *M. parakoreensis*) and their genome sizes (3.89–4.08 Mbp) are among the smallest within the family *Mycobacteriaceae*. The G+C content of the two sequenced species is 69.4 mol %. Although some members of this genus have been isolated from human patients with pulmonary dysfunction, it is unclear whether they exhibit pathogenicity.

The description of *Mycolicibacillus trivialis* comb. nov. as well as the descriptions of new name combinations for other species which are part of the genus *Mycolicibacillus* are provided in Table 9.

**Table 9:** Descriptions of new name combinations for species in the genus *Mycolicibacillus*.

**New name combinations**	**Description and type strain**
*Mycolicibacillus trivialis* comb. nov. (tri.vi.a´lis. L. masc. adj. *trivialis*, common, commonplace, vulgar, ordinary, of little importance)	Basonym: *Mycobacterium triviale* Kubica 1970 (Approved Lists 1980) (Skerman et al., 1980) The description of this taxon is as given by Kubica et al. (1970). The type strain is ATCC 23292 = CCUG 42431 = DSM 44153.
*Mycolicibacillus koreensis* comb. nov. (ko.re.en´sis. N.L. masc. adj. *koreensis*, of or pertaining to the Republic of Korea, the geographical origin of the type strain)	Basonym: *Mycobacterium koreense* Kim et al., 2012 The description of this taxon is as given by Kim et al. (2012). The type strain is 01-305 = DSM 45576 = KCTC 19819.
*Mycolicibacillus parakoreensis* comb. nov. (pa.ra.ko.re.en´sis. Gr. prep. *para* beside, alongside of, near, like; N.L. masc. adj. *koreensis* of or belonging to Korea, and also a bacterial specific epithet; N.L. masc. adj. *parakoreensis* near (*Mycobacterium*) *koreensis* (*koreense*)	Basonym: *Mycobacterium parakoreense* Kim et al., 2013 The description of this taxon is as given by Kim et al. (2013). The type strain is 299 = DSM 45575 = KCTC 19818.

### Description of *Mycobacteroides* gen. nov.

*Mycobacteroides* (My.co.bac.te.ro´i.des. N.L. neut. n. *Mycobacterium*, a bacterial genus; L. neut. suff. -*oides*, resembling; N.L. neut. n. *Mycobacteroides*, a genus resembling *Mycobacterium*).

The type species is *Mycobacteroides abscessus*. The genus *Mycobacteriodes* is comprised of bacteria that are commonly referred to as members of the *Abscessus-Chelonae* clade. This is another genus within the family *Mycobacteriaceae* of rapidly-growing bacterial species (besides *Mycolicibacterium*) which take <7 days to form colonies. Phenotypic characteristics of this genus include a positive 3-day arylsulfatase test, better growth at 30°C than at a 35°C, negative nitrate reductase, negative iron uptake and resistance to polymyxin B (Brown-Elliott and Wallace, 2002). The genome size for the species within this clade ranges from 4.5 to 5.6 Mbp and their G+C content ranges from 63.9 to 64.8 mol %. Phylogenetic studies show that members of the genus *Mycobacteriodes* form a deep branching monophyletic clade within the family *Mycobacteriaceae* that is distinct from all other genera within this family. Some members from this genus are known to be involved in causing lung, skin and soft tissue infections (Magee and Ward, 2012; Tortoli, 2014) and some exhibit resistance to multiple antimicrobial drugs (Nessar et al., 2012).

The members of the genus *Mycobacteriodes* can be reliably distinguished from all other *Mycobacteriaceae* species as well as other bacteria based upon unique shared presence of 27 CSIs in different proteins listed in Table 3 (viz. uracil phosphoribosyltransferase, L-histidine N(alpha)-methyltransferase, DUF58 domain-containing protein, NADH-quinone oxidoreducatase subunit G, ATP-dependent helicase, tRNA (cytidine(34)-2′-O)-methyltransferase, glutamine-fructose-6-phosphate transaminase (isomerizing), error-prone DNA polymerase, 2-amino-4-hydroxy-6-hydroxymethyldihydropteridine diphosphokinase, DEAD/DEAH box helicase, anion transporter, a membrane protein, nicotinate-nucleotide adenylyltransferase, CoA ester lyase, bifunctional ADP-dependent (S)-NAD(P)H-hydrate dehydratase/NAD(P)H-hydrate epimerase, pyridoxal phosphate-dependent aminotransferase, carotenoid oxygenase, SAM-dependent methyltransferase, phosphoribosylamine-glycine ligase, and hypothetical proteins) and the presence of 24 conserved signature proteins listed in Table 2, (viz. MAB_0188c, MAB_0375, MAB_0601, MAB_2852c, MAB_3058, MAB_3079c, MAB_1107c, MAB_1519, MAB_1642, MAB_0008, MAB_0245c, MAB_2487, MAB_3020c, MAB_1440c, MAB_0014, MAB_0015, MAB_0345, MAB_0448c, MAB_0456, MAB_0460, MAB_2549, MAB_1765, MAB_1767, and MAB_1806) that are also specifically found in these bacteria.

The description of *Mycobacteroides abscessus* comb. nov. as well as the descriptions of new name combinations for other species which are part of the genus *Mycobacteroides* are provided in Table 10.

**Table 10:** Descriptions of new name combinations for species in the genus *Mycobacteroides*.

**New name combinations**	**Description and type strain**
*Mycobacteroides abscessus* comb. nov. (abs.ces´sus. L. gen. n. *abscessus*, of an abscess, referring to the ability of the organism to form abscesses)	Basonym: *Mycobacterium abscessus* (Moore and Frerichs, 1953) and Kusunoki Ezaki 1992 The description of this taxon is as given by Kusunoki and Ezaki (1992), Tortoli et al. (2013). The type strain is Hauduroy L948 = TMC 1543 = ATCC 19977 = CCUG 20993 = CIP 104536 = DSM 44196 = JCM 13569 = NCTC 13031.
*Mycobacteroides abscessus* subsp. *abscessus* comb. nov. (abs.ces´sus. L. gen. n. *abscessus*, of an abscess)	Basonym: *Mycobacterium abscessus* subsp. *abscessus* (Moore and Frerichs, 1953) Leao et al., 2011.The description of this taxon is as given by Leao et al. (2011); Tortoli et al. (2013). The type strain is Hauduroy L948 = TMC 1543 = ATCC 19977 = CCUG 20993 = CIP 104536 = DSM 44196 = JCM 13569 = NCTC 13031.
*Mycobacteroides abscessus* subsp. *bolletii* comb. nov. (bol.let´i.i. N.L. gen. masc. n. *bolletii* of Bollet, to honour Claude Bollet, a famous clinical microbiologist and taxonomist)	Basonym: *Mycobacterium abscessus* subsp. *bolletii* (Adékambi et al., 2006a) Leao et al., 2011. The description of this taxon is as given by Leao et al. (2011), Tortoli et al. (2013). The type strain is BD = CCUG 50184 = CIP 108541 = JCM 15297.
*Mycobacteroides abscessus* subsp. *massiliense* comb. nov. (mas.si.li.en´se. L. neut. adj. *massiliense*, of the French city of Massilia, now Marseilles, France)	Basonym: *Mycobacterium abscessus* subsp. *massiliense* Tortoli et al., 2016 The description of this taxon is as given by Tortoli et al. (2016). The type strain is CCUG 48898 = CIP 108297 = KCTC 19086 = DSM 45103.
*Mycobacteroides chelonae* comb. nov. (che.lo´nae. Gr. n. *khelone*, a tortoise; N.L. gen. n. *chelonae*, of a tortoise)	Basonym: *Mycobacterium chelonae* Bergey et al., 1923 (Approved Lists 1980) (Skerman et al., 1980) The description of this taxon is as given by Bergey et al. (1923). The type strain is CM 6388 = ATCC 35752 = CCUG 47445 = CIP 104535 = DSM 43804 = JCM 6388 = NCTC 946.
*Mycobacteroides immunogenum* comb. nov. (im.mu.no.ge´num. N. L. neut. adj. *immunogenum* eliciting an immune response)	Basonym: *Mycobacterium immunogenum* Wilson et al., 2001 The description of this taxon is as given by Wilson et al. (2001). The type strain is BH29 = MC 779 = ATCC 700505 = DSM 45595.
*Mycobacteroides salmoniphilum* comb. nov. (sal.mo.ni´phi.lum. L. n. *salmo*, -*onis* a salmon; Gr. adj. philos loving; N.L. neut. adj. *salmoniphilum* salmon-loving)	Basonym: *Mycobacterium salmoniphilum* (*ex* Ross 1960) Wilson et al., 2001 The description of this taxon is as given by Whipps et al. (2007). The type strain is SC = ATCC 13758 = DSM 43276.
*Mycobacteroides franklinii* comb. nov. (frank.li´ni.i. N.L. masc. gen. n.*franklinii* of Franklin, pertaining to Benjamin Franklin, famous USA statesman and scientist from Pennsylvania where the first isolates originated)	Basonym: *Mycobacterium franklinii* Nogueira et al. 2015 The description of this taxon is as given by Nogueira et al. (2015a). The type strain is DSM 45524 = ATCC BAA-2149.
*Mycobacteroides saopaulense* comb. nov. (sa.o.paul.en´se. N.L. neut. adj. *saopaulense* of or pertaining to the Brazilian state of São Paulo, where the first strains were isolated)	Basonym: *Mycobacterium saopaulense* Nogueira et al. 2015 The description of this taxon is as given by Nogueira et al. (2015b). The type strain is EPM 10906 = CCUG 66554 = LMG 28586 = INCQS 0733.

### Description of *Mycolicibacterium* gen. nov.

*Mycolicibacterium* (My.co.li.ci.bac.te´ri.um. N.L. n. *acidum mycolicum*, mycolic acid; N.L. neut. n. *bacterium*, a small rod; N.L. neut. n. *Mycolicibacterium*, a genus of mycolic acid containing rod-shaped bacteria).

The type species *Mycolicibacterium fortuitum*.

The genus is comprised of rapidly-growing bacterial species, which take <7 days to form colonies upon primary isolation (Parte, 2014). Some other phenotypic characteristics generally common to the members of this genus include absence of pigmentation, positive 3-day arylsulfatase activity (Brown-Elliott and Wallace, 2002), positive for nitrate reductase and iron uptake (Magee and Ward, 2012). Most species are saprophytic and considered non-pathogenic to humans, however some cases of infections and diseases by members of this group have been reported (Stahl and Urbance, 1990; Brown-Elliott and Wallace, 2002; Ripoll et al., 2009). The members of this genus form a monophyletic clade in phylogenetic trees based on concatenated sequences of multiple large datasets of conserved proteins including a tree based on 1941 core proteins from mycobacterial genomes, a tree based on 136 core proteins for the phylum Actinobacteria, and another tree based on concatenated sequences for 8 conserved proteins described in the present study.

The members of the genus *Mycolicibacterium* can be distinguished from other genera within the family *Mycobacteriaceae* as well as other bacteria based upon conserved signature indels in the following four proteins viz. LacI family transcriptional regulator, Cyclase, CDP-diacylglycerol–glycerol-3-phosphate 3-phosphatidyltransferase and CDP-diacylglycerol–serine O-phosphatidyltransferase (Table 4) that are uniquely shared by the members of this genus. Additionally, the homologs of the 10 conserved signature proteins, whose accession numbers are as follows (WP_048630777.1, WP_048632025.1, WP_048632497.1, WP_048634851.1, WP_048633467.1, WP_048633322.1 WP_048631132.1, WP_048634509.1, WP_048630657.1, and WP_048632441.1) are also uniquely found in the members of this genus (Table 5). The genome size for the members of this genus ranges from 3.95 to 8.0 Mbp and their G+C content ranges from 65.4 to 70.3 mol %.

The description of *Mycolicibacterium fortuitum* comb. nov. as well as the descriptions of new name combinations for other species which are part of the genus *Mycolicibacterium* are provided in Table 11.

**Table 11:** Descriptions of new name combinations for species in the genus *Mycolicibacterium*.

**New name combinations**	**Description and type strain**
*Mycolicibacterium fortuitum* comb. nov. (for.tu´i.tum. L. neut. adj. *fortuitum*, casual, accidental, fortuitous)	Basonym: *Mycobacterium fortuitum* da Costa Cruz 1938 (Approved Lists, 1980) (Skerman et al., 1980) The description of this taxon is as given by da Costa Cruz (1938). The type strain is ATCC 6841 = CCUG 20994 = CIP 104534 = DSM 46621 = IFO (now NBRC) 13159 = JCM 6387 = NCTC 10394.
*Mycolicibacterium fortuitum* subsp. *acetamidolyticum* comb. nov. (a.cet.a.mi.do.ly´ti.cum. N.L. neut. n. *acetamidum*, acetamide; N.L. neut. adj. *lyticum* (from Gr. neut. adj. *lytikon*), able to loosen, able to dissolve; N.L. neut. adj. *acetamidolyticum*, digesting acetamide)	Basonym: *Mycobacterium fortuitum* subsp. *acetamidolyticum* Tsukamura et al. 1986 The description of this taxon is as given by Tsukamura et al. (1986a,b). The type strain is NCH E11620 = ATCC 35931 = CIP 105423 = DSM 44220 = JCM 6368.
*Mycolicibacterium fortuitum* subsp. *fortuitum* comb. nov. (for.tu´i.tum. L. neut. adj. *fortuitum*, casual, accidental, fortuitous)	Basonym: *Mycobacterium fortuitum* subsp. *fortiutum* (da Costa Cruz, 1938) Tsukamura et al. 1986 The description of this taxon is as given by da Costa Cruz (1938). The type strain is ATCC 6841 = CCUG 20994 = CIP 104534 = DSM 46621 = IFO (now NBRC) 13159 = JCM 6387 = NCTC 10394.
*Mycolicibacterium agri* comb. nov. (ag´ri, L. gen. n. *agri*, of a field)	Basonym: *Mycobacterium agri* (*ex* Tsukamura 1972) Tsukamura 1981 The description of this taxon is as given by Tsukamura (1981). The type strain is 90012 = ATCC 27406 = CCUG 37673 A = CIP 105391 = DSM 44515 = JCM 6377.
*Mycolicibacterium aichiense* comb. nov. (ai.chi.en´se. N.L. neut. adj. *aichiense*, of or belonging to Aichi prefecture, Japan)	Basonym: *Mycobacterium aichiense* (*ex* Tsukamura et al. 1973) Tsukamura 1981 The description of this taxon is as given by Tsukamura et al. (1981). The type strain is 49005 (previously, strain 5545) = ATCC 27280 = CIP 106808 = DSM 44147 = JCM 6376 = LMG 19259 = NCTC 10820.
*Mycolicibacterium alvei* comb. nov. (al´ve.i. L. gen. n. *alvei*, of the bed of a river, referring to the place where this species was first isolated)	Basonym: *Mycobacterium alvei* Ausina et al. 1992 The description of this taxon is as given by Ausina et al. (1992). The type strain is CR-21 = ATCC 51304 = CIP 103464 = DSM 44176 = JCM 12272.
*Mycolicibacterium anyangense* comb. nov. (an.yang.en´se. N.L. neut. adj. *anyangense*, pertaining to Anyang, Republic of Korea, the geographical location of the agency isolating the type strain)	Basonym: *Mycobacterium anyangense* Kim et al. 2015 The description of this taxon is as given by Kim et al. (2015). The type strain is QIA-38 = JCM 30275 = KCTC 29443.
*Mycolicibacterium arabiense* comb. nov. (a.ra.bi.en´se. N.L. neut. adj. *arabiense*, of or belonging to Arabia, referring to the isolation of the type strain in Dubai, United Arab Emirates)	Basonym: *Mycobacterium arabiense* Zhang et al. 2013 The description of this taxon is as given by Zhang et al. (2013a). The type strain is YIM 121001T = DSM 45768T = JCM 18538.
*Mycolicibacterium arcueilense* comb. nov. (ar.cueil.en´se. N.L. neut. adj. *arcueilense*, of or belonging to Arcueil, pertaining to the town of Arcueil (south of Paris) where most isolates were recovered)	Basonym: *Mycobacterium arcueilense* Konjek et al. 2016 The description of this taxon is as given by Konjek et al. (2016). The type strain is 269 = ParisRGMnew_3 = CIP 110654 = DSM 46715.
*Mycolicibacterium aromaticivorans* comb. nov. [a.ro.ma.ti.ci.vo´rans. L. adj. *aromaticus*, aromatic, fragrant; L. pres. part. *vorans*, devouring; N.L. part. adj. *aromaticivorans*, devouring aromatic (compounds)]	Basonym: *Mycobacterium aromaticivorans* Hennessee et al. 2009 The description of this taxon is as given by Hennessee et al. (2009). The type strain is JS19b1 = ATCC BAA-1378 = CIP 109274 = JCM 16368.
*Mycolicibacterium aubagnense* comb. nov. (au.bag.nen´se. N.L. neut. adj. *aubagnense*, of or pertaining to Aubagne, the city from where the first patient originated)	Basonym: *Mycobacterium aubagnense* Adékambi et al. 2006 The description of this taxon is as given by Adékambi et al. (2006a). The type strain is U8 = CCUG 50186 = CIP 108543 = JCM 15296.
*Mycolicibacterium aurum* comb. nov. (au´rum. L. n. *aurum*, the gold, the color of gold, intended to mean gold-pigmented)	Basonym: *Mycobacterium aurum* Tsukamura 1966 (Approved Lists 1980) (Skerman et al., 1980) The description of this taxon is as given by Tsukamura (1966). The type strain is ATCC 23366 = CCUG 37666 = CIP 104465 = DSM 43999 = HAMBI 2275 = JCM 6366 = LMG 19255 = NCTC 10437 = NRRL B-4037.
*Mycolicibacterium austroafricanum* comb. nov. (aus.tro.a.fri.ca´num. L. adj. *australis*, southern; L. adj. *africanus*, pertaining to Africa; N.L. neut. adj. *austroafricanum*, of or pertaining to South Africa, the source of the isolates)	Basonym: *Mycobacterium austroafricanum* Tsukamura et al. 1983 The description of this taxon is as given by Tsukamura et al. (1983c). The type strain is E9789-SA12441 = ATCC 33464 = CCUG 37667 = CIP 105395 = DSM 44191 = HAMBI 2271 = JCM 6369.
*Mycolicibacterium bacteremicum* comb. nov. (bac.ter.e´mi.cum. N.L. n. *bacteremia*, bacteremia; N.L. neut. suff. -(*i*)*cum*, suffix used with the sense of pertaining to; N.L. neut. adj. *bacteremicum*, pertaining to bacteremia, referring to the organism´s association with bloodstream infections)	Basonym: *Mycobacterium bacteremicum* (Brown-Elliott et al., 2012) The description of this taxon is as given by Brown-Elliott et al. (2010). The type strain is ATCC 25791 = DSM 45578.
*Mycolicibacterium boenickei* comb. nov. (boe.ni´cke.i. N.L. gen. masc. n. *boenickei*, of Bönicke, in honor of the contribution of Rudolf Bönicke, a German mycobacteriologist, who first recognized the heterogeneity within the *Mycobacterium fortuitum* complex)	Basonym: *Mycobacterium boenickei* Schinsky et al. 2004 The description of this taxon is as given by Schinsky et al. (2004). The type strain is W5998 = ATCC 49935 = DSM 44677 = JCM 15653.
*Mycolicibacterium brisbanense* comb. nov. (bris.ban.en´se. N.L. neut. adj. *brisbanense*, of or pertaining to Brisbane, Queensland, Australia, the source of the type strain)	Basonym: *Mycobacterium brisbanense* Schinsky et al. 2004 The description of this taxon is as given by Schinsky et al. (2004). The types strain is W6743 = ATCC 49938 = CCUG 47584 = DSM 44680 = JCM 15654.
*Mycolicibacterium brumae* comb. nov. (bru´mae. L. gen. n. *brumae*, of winter, referring to the time of year at which the first strains were isolated)	Basonym: *Mycobacterium brumae* Luquin et al. 1993 The description of this taxon is as given by Luquin et al. (1993). The type strain is CR-270 = ATCC 51384 = CCUG 37586 = CIP 103465 = DSM 44177 = JCM 12273.
*Mycolicibacterium canariasense* comb. nov. (ca.na.ri.as.en´se. L. neut. adj. *canariasense*, of or belonging to the Canarias (the Spanish name of the Canary Islands), where all strains were isolated)	Basonym: *Mycobacterium canariasense* (Jíménez et al., 2004) The description of this taxon is as given by Jíménez et al. (2004). The type strain is 502329 = CCUG 47953 = CIP 107998 = JCM 15298.
*Mycolicibacterium celeriflavum* comb. nov. (ce.le.ri.fla´vum. L. adj. *celer*, rapid; L. neut. adj. *flavum*, yellow; N.L. neut. adj. *celeriflavum*, referring to rapid growth and yellow pigmentation features of the species)	Basonym: *Mycobacterium celeriflavum* Shahraki et al. 2015 The description of this taxon is as given by Shahraki et al. (2015). The type strain is AFPC-000207 = DSM 46765 = JCM 18439.
*Mycolicibacterium chitae* comb. nov. (chi´tae. N.L. gen. n. *chitae*, of Chita, a place in Japan)	Basonym: *Mycobacterium chitae* Tsukamura 1967 (Approved Lists 1980) (Skerman et al., 1980) The description of this taxon is as given by Tsukamura (1967b). The type strain is ATCC 19627 = CCUG 39504 = CIP 105383 = DSM 44633 = JCM 12403 = NCTC 10485.
*Mycolicibacterium chlorophenolicum* comb. nov. (chlo.ro.phe.no´li.cum. N.L. n. *chlorophenol*, chlorophenol; L. neut. suffix -*icum*, suffix used with the sense of pertaining to; N.L. neut. adj. *chlorophenolicum*, related to chlorophenols)	Basonym: *Mycobacterium chlorophenolicum* (Apajalahti et al., 1986) Brigila et al. 1994 The description of this taxon is as given by Apajalahti et al. (1986). The type strain is PCP-I = ATCC 49826 = CIP 104189 = DSM 43826 = HAMBI 2278 = IEGM 559 = IFO (now NBRC) 15527 = JCM 7439 = NRRL B-16528.
*Mycolicibacterium chubuense* comb. nov. (chu.bu.en´se. N.L. neut. adj. *chubuense*, of or belonging to Chubu, coming from soil of Chubu hospital)	Basonym: *Mycobacterium chubuense* (*ex* Tsukamura et al. 1973) Tsukamura et al. 1981 The description of this taxon is as given by Tsukamura et al. (1981). The type strain is 48013 (previously, strain 5517) = ATCC 27278 = CCUG 37670 = CIP 106810 = DSM 44219 = JCM 6374 = JCM 16420 = NCTC 10819.
*Mycolicibacterium conceptionense* comb. nov. (con.cep.tio.nen´se. N.L. neut. adj. *conceptionense*, of or pertaining to Hôpital de la Conception, the hospital where the first strain was isolated)	Basonym: *Mycobacterium conceptionense* Adékambi et al. 2006 The description of this taxon is as given by Adékambi et al. (2006b,c). The type strain is D16 = CCUG 50187 = CIP 108544 = JCM 15299.
*Mycolicibacterium confluentis* comb. nov. (con.flu.en´tis. M.L. gen. n. *confluentis*, of *Confluentes*, now Koblenz, the source of the strain on which the species description is based)	Basonym: *Mycobacterium confluentis* Kirschner et al. 1992 The description of this taxon is as given by Kirschner et al. (1992). The type strain is 1389/90 = ATCC 49920 = CIP 105510 = DSM 44017 = JCM 13671.
*Mycolicibacterium cosmeticum* comb. nov. (cos.me´ti.cum. N.L. neut. adj. *cosmeticum* (from Gr. adj. *kosmetikos*), referring to cosmetics)	Basonym: *Mycobacterium cosmeticum* Cooksey et al. 2004 The description of this taxon is as given by Cooksey et al. (2004). The type strain is LTA-388 = ATCC BAA-878 = CIP 108170 = JCM 14739.
*Mycolicibacterium crocinum* comb. nov. (cro´ci.num. L. neut. adj. *crocinum*, saffron-colored, pertaining to the colony pigmentation of known strains)	Basonym: *Mycobacterium crocinum* Hennessee et al. 2009 The description of this taxon is as given by Hennessee et al. (2009). The type strain is czh-42 = ATCC BAA-1373 = CIP 109269 = JCM 16369.
*Mycolicibacterium diernhoferi* comb. nov. (diern.ho´fe.ri. N.L. gen. masc. n. *diernhoferi*, of Diernhofer, who originally isolated the organisms)	Basonym: *Mycobacterium diernhoferi* (*ex* Bönicke and Juhasz 1965) Tsukamura et al. 1983 The description of this taxon is as given by Tsukamura et al. (1983c). The type strain is 41001 = ATCC 19340 = CIP 105384 = DSM 43524 = HAMBI 2269 = IFO (now NBRC) 14756 = JCM 6371.
*Mycolicibacterium doricum* comb. nov. (do´ri.cum. L. neut. adj. *doricum*, of or belonging to *Dorica civitas*, the ancient name of the Italian city of Ancona, from where the organism was first isolated)	Basonym: *Mycobacterium doricum* Tortoli et al., 2001 The description of this taxon is as given by Tortoli et al. (2001). The type strain is FI-13295 = CCUG 46352 = CIP 106867 = DSM 44339 = JCM 12405.
*Mycolicibacterium duvalii* comb. nov. (du.va´li.i. N.L. gen. masc. n. *duvalii*, of Duval, named for Professor C.W. Duval who isolated two strains of the organism)	Basonym: *Mycobacterium duvalii* Stanford and Gunthorpe1971 The description of this taxon is as given by Stanford and Gunthorpe (1971). The type strain is ATCC 43910 = CCUG 41352 = CIP 104539 = DSM 44244 = JCM 6396 = NCTC 358.
*Mycolicibacterium elephantis* comb. nov. (e.le.phan´tis. L. gen. n. *elephantis*, of an elephant)	Basonym: *Mycobacterium elephantis* Shojaei et al. 2000 The description of this taxon is as given by Shojaei et al. (2000). The type strain is 484 = CIP 106831 = DSM 44368 = JCM 12406.
*Mycolicibacterium fallax* comb. nov. (fal´lax. L. neut. adj. *fallax*, deceptive, in the sense that the colonies resemble those of *Mycobacterium tuberculosis*)	Basonym: *Mycobacterium fallax* Lévy-Frébault et al. 1983 The description of this taxon is as given by Lévy-Frébault et al. (1983). The type strain is ATCC 35219 = CCUG 37584 = CIP 81.39 = DSM 44179 = JCM 6405.
*Mycolicibacterium farcinogenes* comb. nov. (far.ci.no´ge.nes. Fr. n. *farcin* (from L. n. *farciminum*, a disease in horses and other animals), farcy or glanders; Gr. v. *gennaio*, produce; N.L. part. adj. *farcinogenes*, producing farcy)	Basonym: *Mycobacterium farcinogenes* Chamoiseau 1973 (Approved Lists 1980) (Skerman et al., 1980) The description of this taxon is as given by Chamoiseau (1973). The type strain is IEMVT 75 = ATCC 35753 = CCUG 21047 = DSM 43637 = JCM 15463 = NCTC 10955.
*Mycolicibacterium flavescens* comb. nov. (fla.ves´cens. L. v. *flavesco*, to become golden yellow; L. part. adj. *flavescens*, becoming yellow)	Basonym: *Mycobacterium flavescens* Bojalil et al. 1962 (Approved Lists 1980) (Skerman et al., 1980) The description of this taxon is as given by Bojalil et al. (1962). The type strain is ATCC 14474 = CCUG 29041 = CIP 104533 = DSM 43991 = JCM 12274 = NCTC 10271 = NRRL B-4038.
*Mycolicibacterium fluoranthenivorans* comb. nov. (flu.or.an.the.ni.vo´rans. N.L. n. *fluoranthenum*, fluoranthene; L. pres. part. *vorans*, devouring; N.L. part. adj. *fluoranthenivorans*, digesting fluoranthene)	Basonym: *Mycobacterium fluoranthenivorans* Hormisch et al., 2006 The description of this taxon is as given by Hormisch et al. (2004, 2006). The type strain is FA4 = DSM 44556 = CIP 108203 = JCM 14741.
*Mycolicibacterium frederiksbergense* comb. nov. (fre.de.riks.ber.gen´se. N.L. neut. adj. *frederiksbergense*, of or belonging to Frederiksberg, Denmark, referring to the place of isolation)	Basonym: *Mycobacterium frederiksbergense* Willumsen et al. 2001 The description of this taxon is as given by Willumsen et al. (2001). The type strain is FAn9 = CIP 107205 = DSM 44346 = NRRL B-24126.
*Mycolicibacterium gadium* comb. nov. [ga´di.um. L. gen. pl. n. *gadium*, of *Gades*, the modern Cadiz (a town on the Atlantic coast of Spain)]	Basonym: *Mycobacterium gadium* Casal and Calero 1974 (Approved Lists 1980) (Skerman et al., 1980) The description of this taxon is as given by Casal and Calero (1974). The type strain is ATCC 27726 = CCUG 37515 = CIP 105388 = DSM 44077 = HAMBI 2274 = JCM 12688 = NCTC 10942.
*Mycolicibacterium gilvum* comb. nov. (gil´vum. L. neut. adj. *gilvum*, pale yellow)	Basonym: *Mycobacterium gilvum* Stanford and Gunthorpe 1971 (Approved Lists 1980) (Skerman et al., 1980) The description of this taxon is as given by Stanford and Gunthorpe (1971). The type strain is ATCC 43909 = CIP 106743 = JCM 15464 = NCTC 10742.
*Mycolicibacterium goodii* comb. nov. (good´i.i. N.L. gen. masc. n. *goodii*, of Good, named for Robert Good who made significant contributions to the study of mycobacteria)	Basonym: *Mycobacterium goodii* Brown et al. 1999 The description of this taxon is as given by Brown et al. (1999). The type strain is MO69 = ATCC 700504 = CIP 106349 = DSM 44492 = JCM 12689.
*Mycolicibacterium hassiacum* comb. nov. (has.si.a´cum. M.L. neut. adj. *hassiacum*, of or belonging to *Hassia*, the German province of Hesse, where the organism was first isolated)	Basonym: *Mycobacterium hassiacum* Schröder et al. 1997 The description of this taxon is as given by Schröder et al. (1997). The type strain is 3849 = CCUG 37519 = CIP 105218 = DSM 44199 = JCM 12690.
*Mycolicibacterium helvum* comb. nov. (hel´vum. L. neut. adj. *helvum*, pale yellow, intended to mean pale yellow-pigmented)	Basonym: *Mycobacterium helvum* Tran and Dahl 2016 The description of this taxon is as given by Tran and Dahl (2016). The type strain is DL739 = JCM 30396 = NCCB 100520.
*Mycolicibacterium hippocampi* comb. nov. (hip.po.cam´pi. L. gen. n. *hippocampi*, of the seahorse)	Basonym: *Mycobacterium hippocampi* Balcázar et al. 2014 The description of this taxon is as given by Balcázar et al. (2014a,b). The type strain is BFLP-6 = DSM 45391 = LMG 25372.
*Mycolicibacterium hodleri* comb. nov. (hod´le.ri. N.L. gen. masc. n. *hodleri*, of Hodler, named after Christian Hodler, director of the Ministry of Science and Culture of the State of Lower Saxony, Germany, a strong supporter of natural sciences)	Basonym: *Mycobacterium hodleri* Kleespies et al. 1996 The description of this taxon is as given by Kleespies et al. (1996). The type strain is EMI2 = CIP 104909 = DSM 44183 = JCM 12141 = LMG 19253.
*Mycolicibacterium holsaticum* comb. nov. (hol.sa´ti.cum. M.L. neut. adj. *holsaticum*, of or belonging to *Holsatia*, the German region of Holstein, the location of the institute in which the strains were first analyzed)	Basonym: *Mycobacterium holsaticum* Richter et al. 2002 The description of this taxon is as given by Richter et al. (2002). The type strain is 1406 = CCUG 46266 = DSM 44478 = JCM 12374.
*Mycolicibacterium houstonense* comb. nov. (hous.ton.en´se. N.L. neut. adj. *houstonense*, of or pertaining to Houston, TX, USA, where the first isolate of the *Mycobacterium fortuitum* third biovariant (sorbitol positive) was identified)	Basonym: *Mycobacterium houstonense* Schinsky et al. 2004 The description of this taxon is as given by Schinsky et al. (2004). The type strain is W5198 = ATCC 49403 = DSM 44676 = JCM 15656.
*Mycolicibacterium insubricum* comb. nov. (in.su´bri.cum. L. neut. adj. *insubricum*, pertaining to *Insubria*, the Latin name of part of the Lombardy region of Italy that includes the cities in which four of the first five strains were isolated, including the type strain)	Basonym: *Mycobacterium insubricum* Tortoli et al., 2009 The description of this taxon is as given by Tortoli et al. (2009). The type strain is FI-06250 = CIP 109609 = DSM 45132 = JCM 16366.
*Mycolicibacterium iranicum* comb. nov. (i.ra´ni.cum. N.L. neut. adj. *iranicum*, of or belonging to Iran, isolated in Iran)	Basonym: *Mycobacterium iranicum* Shojaei et al. 2013 The description of this taxon is as given by Shojaei et al. (2013). The type strain is M05 = DSM 45541 = CCUG 62053 = JCM 17461.
*Mycolicibacterium komossense* comb. nov. (ko.mos.sen´se. N.L. neut. adj. *komossense*, of or belonging to Komosse sphagnum bog in south Sweden)	Basonym: *Mycobacterium komossense* Kazda and Müller 1979 (Approved Lists 1980) (Skerman et al., 1980) The description of this taxon is as given by Kazda and Müller (1979). The type strain is Ko 2 = ATCC 33013 = CIP 105293 = DSM 44078 = HAMBI 2279 = HAMBI 2280 = JCM 12408.
*Mycolicibacterium litorale* comb. nov. (li.to.ra´le. L. neut. adj. *litorale*, of or belonging to the seashore)	Basonym: *Mycobacterium litorale* Zhang et al. 2012 The description of this taxon is as given by Zhang et al. (2012). The type strain is F4 = CGMCC 4.5724 = JCM 17423.
*Mycolicibacterium llatzerense* comb. nov. (llat.ze.ren´se. N.L. neut. adj. *llatzerense*, pertaining to Hospital Son Llàtzer, the hospital where the strains were isolated)	Basonym: *Mycobacterium llatzerense* Gomila et al. 2008 The description of this taxon is as given by Gomila et al. (2008). The type strain is MG13 = CCUG 54744 = CECT 7273 = JCM 16229.
*Mycolicibacterium lutetiense* comb. nov. (lu.te.ti.en´se. N.L. neut. adj. *lutetiense*, of or belonging to Lutetia, now Paris, pertaining to the widespread distribution of this species within the Paris water distribution system)	Basonym: *Mycobacterium lutetiense* Konjek et al. 2016 The description of this taxon is as given by Konjek et al. (2016). The type strain is 071 = ParisRGMnew_1 = CIP 110656 = DSM 46713.
*Mycolicibacterium madagascariense* comb. nov. (ma.da.gas.car.i.en´se. N.L. neut. adj. *madagascariense*, of or belonging to the island of Madagascar, the source of the strains)	Basonym: *Mycobacterium madagascariense* Kazda et al., 1992 The description of this taxon is as given by Kazda et al. (1992). The type strain is P2 = ATCC 49865 = CIP 104538 = JCM 13574.
*Mycolicibacterium mageritense* comb. nov. (ma.ge.ri.ten´se. N.L. neut. adj. *mageritense*, of or pertaining to *Magerit*, old (first) Arabic name of Madrid, the source of most of the isolates)	Basonym: *Mycobacterium mageritense* Domenech et al. 1997 The description of this taxon is as given by Domenech et al. (1997). The type strain is 938 = ATCC 700351 = CCUG 37984 = CIP 104973 = DSM 44476 = JCM 12375.
*Mycolicibacterium malmesburyense* comb. nov. (mal.mes.bu.ry.en´se. N.L. neut. adj. *malmesburyense* pertaining to Malmesbury, after a town (Malmesbury) in South Africa, where one of the isolates (the type strain) of this species originated from)	Basonym: *Mycobacterium malmesburyense* Gcebe et al. 2017 The description of this taxon is as given by Gcebe et al. (2017). The type strain is WCM 7299 = ATCC BAA-2759 = CIP 110822.
*Mycolicibacterium monacense* comb. nov. (mo.na.cen´se. M.L. neut. adj. *monacense*, of or belonging to *Monacum*, the Latin name of the German city Munich where the first strain was isolated)	Basonym: *Mycobacterium monacense* Reischl et al. 2006 The description of this taxon is as given by Reischl et al. (2006). The type strain is B9-21-178 = CIP 109237 = DSM 44395 = JCM 15658.
*Mycolicibacterium montmartrense* comb. nov. (mont.mar.tren´se. N.L. neut. adj. *montmartrense*, pertaining to the Parisian quartier of Montmartre where most isolates were recovered ‘Mycobacterium sp. NL-JvlW-016’ (van Ingen *et al*., 2010))	Basonym: *Mycobacterium montmartrense* Konjek et al. 2016 The description of this taxon is as given by Konjek et al. (2016). The type strain is 196 = ParisRGMnew_2 = CIP 110655 = DSM 46714. A RGM isolate putatively belonging to this species on the basis of partial *rpoB* sequence (99% identity across 636 bp) has been reported in the Netherlands under the designation
*Mycolicibacterium moriokaense* comb. nov. (mo.ri.o.ka.en´se. N.L. neut. adj. *moriokaense*, of or belonging to Morioka, the locality where the species was first isolated)	Basonym: *Mycobacterium moriokaense* Tsukamura et al. 1986c The description of this taxon is as given by Tsukamura et al. (1986c). The type strain is NCH E11715 = ATCC 43059 = CCUG 37671 = CIP 105393 = DSM 44221 = JCM 6375 = VKM Ac-1183.
*Mycolicibacterium mucogenicum* comb. nov. (mu.co.ge´ni.cum. L. n. *mucus*, mucus, Gr. v. *gennaio*, to produce; L. neut. suff. -*icum*, suffix used with the sense of pertaining to; N.L. neut. adj. *mucogenicum*, intended to mean producing mucus, referring to the highly mucoid character of most strains on solid agar)	Basonym: *Mycobacterium mucogenicum* Springer et al. 1995 The description of this taxon is as given by Springer et al. (1995). The type strain is MO76 = ATCC 49650.
*Mycolicibacterium murale* comb. nov. (mu.ra´le. L. neut. adj. *murale*, of or belonging to a wall)	Basonym: *Mycobacterium murale* Vuorio et al. 1999 The description of this taxon is as given by Vuorio et al. (1999). The type strain is MA112/96 = CCUG 39728 = CIP 105980 = DSM 44340 = HAMBI 2320 = JCM 13392.
*Mycolicibacterium neoaurum* comb. nov. (ne.o.au´rum. Gr. adj. *neos*, new; L. n. *aurum*, gold; N.L. n. *neoaurum*, a new gold, intended to mean a new gold-pigmented organism)	Basonym: *Mycobacterium neoaurum* Tsukamura 1972 (Approved Lists 1980) (Skerman et al., 1980) The description of this taxon is as given by Tsukamura (1972). The type strain is ATCC 25795 = CCUG 37665 = CIP 105387 = DSM 44074 = HAMBI 2273 = JCM 6365 = NCTC 10818.
*Mycolicibacterium neworleansense* comb. nov. (new.or.le.ans.en´se. N.L. neut. adj. *neworleansense*, of or pertaining to New Orleans, LA, USA, the source of the type strain)	Basonym: *Mycobacterium neworleansense* Schinsky et al. 2004 The description of this taxon is as given by Schinsky et al. (2004). The type strain is W6705 = ATCC 49404 = DSM 44679 = JCM 15659.
*Mycolicibacterium novocastrense* comb. nov. (no.vo.cas.tren´se. L. adj. *novus*, new; L. n. *castrum*, castle; N.L. neut. adj. *novocastrense*, of or pertaining to Newcastle, a city in the northeast of England)	Basonym: *Mycobacterium novocastrense* Shojaei et al. 1997 The description of this taxon is as given by Shojaei et al. (1997). The type strain is 73 = CIP 105546 = DSM 44203 = JCM 18114.
*Mycolicibacterium obuense* comb. nov. (o.bu.en´se. N.L. neut. adj. *obuense*, of or belonging to Obu, Japan)	Basonym: *Mycobacterium obuense* (*ex* Tsukamura and Mizuno 1971) Tsukamura and Mizuno 1981 The description of this taxon is as given by Tsukamura et al. (1981). The type strain is 47001 (previously, strain 4388) = ATCC 27023 = CCUG 37669 = CIP 106803 = DSM 44075 = HAMBI 2272 = JCM 6372 = NCTC 10778.
*Mycolicibacterium oryzae* comb. nov. (o.ry´zae. L. gen. n. *oryzae*, of rice, the origin of the type strain).	Basonym: *Mycobacterium oryzae* Ramaprasad et al. 2016 The description of this taxon is as given by Ramaprasad et al. (2016). The type strain is JC290 = KCTC 39560 = LMG 28809.
*Mycolicibacterium pallens* comb. nov. (pal´lens. L. neut. adj. *pallens*, pale yellow, pertaining to the colony pigmentation of the type strain)	Basonym: *Mycobacterium pallens* Hennessee et al. 2009 The description of this taxon is as given by Hennessee et al. (2009). The type strain is czh-8 = ATCC BAA-1372 = CIP 109268 = JCM 16370.
*Mycolicibacterium parafortuitum* comb. nov. (pa.ra.for.tu´i.tum. Gr. prep. *para*, alongside of or near; L. neut. adj. *fortuitum*, casual, accidental, and also a specific epithet; N.L. neut. adj. *parafortuitum*, alongside of (*Mycobacterium*) *fortuitum*)	Basonym: *Mycobacterium parafortuitum* Tsukamura, 1965 (Approved Lists 1980) (Skerman et al., 1980) The description of this taxon is as given by Tsukamura (1965b). The type strain is ATCC 19686 = CCUG 20999 = CIP 106802 = DSM 43528 = JCM 6367 = NCTC 10411 = NRRL B-4035.
*Mycolicibacterium peregrinum* comb. nov. (pe.re.gri´num. L. neut. adj. *peregrinum*, strange, foreign)	Basonym: *Mycobacterium peregrinum* (*ex* Bojalil et al. 1962) Kusunoki and Ezaki 1992 The description of this taxon is as given by Kusunoki and Ezaki (1992). The type strain is ATCC 14467 = CCUG 27976 = CIP 105382 = DSM 43271 = JCM 12142 = NCTC 10264.
*Mycolicibacterium phlei* comb. nov. (phle´i. N.L. neut. n. *Phleum*, a genus of grass, timothy; N.L. gen. n. *phlei*, of *Phleum*, of timothy)	Basonym: *Mycobacterium phlei* Lehmann and Neumann 1899 (Approved Lists 1980) (Skerman et al., 1980) The description of this taxon is as given by Lehmann and Neumann (1899). The type strain is ATCC 11758 = CCUG 21000 = CIP 105389 = DSM 43239 = JCM 5865 = JCM 6385 = NCTC 8151 = NRRL B-14615 = VKM Ac-1291.
*Mycolicibacterium phocaicum* comb. nov. (pho.ca´i.cum. L. neut. adj. *phocaicum*, Phocœan, referred to Phocaea, a maritime town of Ionia, a colony of the Athenians, whose inhabitants fled, to escape from Persian domination, and founded Massilia (Marseille), which was the source of the type strain)	Basonym: *Mycobacterium phocaicum* Adékambi et al. 2006 The description of this taxon is as given by Adékambi et al. (2006a). The type strain is N4 = CCUG 50185 = CIP 108542 = JCM 15301.
*Mycolicibacterium porcinum* comb. nov. (por.ci.num. L. neut. adj. *porcinum*, pertaining to swine)	Basonym: *Mycobacterium porcinum* Tsukamura et al. 1983 The description of this taxon is as given by Tsukamura et al. (1983b). The type strain is E10241-1 = ATCC 33776 = CCUG 37674 = CIP 105392 = DSM 44242 = JCM 6378.
*Mycolicibacterium poriferae* comb. nov. (po.ri´fe.rae. N.L. gen. *poriferae* of the *Porifera*, the phylum of sponges)	Basonym: *Mycobacterium poriferae* Padgitt and Moshier 1987 The description of this taxon is as given by Padgitt and Moshier (1987). The type strain is 47 = ATCC 35087 = CIP 105394 = JCM 12603.
*Mycolicibacterium psychrotolerans* comb. nov. (psy.chro.to´le.rans. Gr. adj. *psychros*, cold; L. pres. part. *tolerans*, tolerating; N.L. part. adj. *psychrotolerans*, cold-tolerating)	Basonym: *Mycobacterium psychrotolerans* Trujillo et al. 2004 The description of this taxon is as given by Trujillo et al. (2004). The type strain is WA101 = DSM 44697 = JCM 13323 = LMG 21953.
*Mycolicibacterium pulveris* comb. nov. (pul´ve.ris. L. gen. n. *pulveris*, of dust, referring to the source, house dust)	Basonym: *Mycobacterium pulveris* Tsukamura et al. 1983 The description of this taxon is as given by Tsukamura et al. (1983a). The type strain is NCH 33505 = ATCC 35154 = CCUG 37668 = CIP 106804 = DSM 44222 = JCM 6370.
*Mycolicibacterium pyrenivorans* comb. nov. (py.re.ni.vo´rans. N.L. n. *pyrenum*, pyrene; L. pres. part. *vorans*, devouring, destroying; N.L. part. adj. *pyrenivorans*, destroying pyrene)	Basonym: *Mycobacterium pyrenivorans* Derz et al. 2004 The description of this taxon is as given by Derz et al. (2004). The type strain is 17A3 = DSM 44605 = JCM 15927 = NRRL B-24349.
*Mycolicibacterium rhodesiae* comb. nov. (rho.de.si´ae. N.L. gen. n. *rhodesiae*, of/from Rhodesia)	Basonym: *Mycobacterium rhodesiae* (*ex* Tsukamura et al. 1971) Tsukamura et al. 1981 The description of this taxon is as given by Tsukamura et al. (1981). The types strain is 02002 (previously, strain 5295) = ATCC 27024 = CIP 106806 = DSM 44223 = JCM 6363 = NCTC 10779.
*Mycolicibacterium rufum* comb. nov. (ru´fum. L. neut. adj. *rufum* ruddy or red, pertaining to the colony pigmentation of the type strain)	Basonym: *Mycobacterium rufum* Hennessee et al. 2009 The description of this taxon is as given by Hennessee et al. (2009). The type strain is JS14 = ATCC BAA-1377 = CIP 109273 = JCM 16372.
*Mycolicibacterium rutilum* comb. nov. (ru´ti.lum. L. neut. adj. *rutilum*, rust-colored, pertaining to the colony pigmentation of known strains)	Basonym: *Mycobacterium rutilum* Hennessee et al. 2009 The description of this taxon is as given by Hennessee et al. (2009). The type strain is czh-117 = ATCC BAA-1375 = CIP 109271 = JCM 16371.
*Mycolicibacterium sarraceniae* comb. nov. (sar.ra.ce´ni.ae. N.L. fem. gen. n. *sarraceniae*, of *Sarracenia*, for the pitcher plant from where the species was isolated)	Basonym: *Mycobacterium sarraceniae* Tran and Dahl 2016 The description of this taxon is as given by Tran and Dahl (2016). The type strain is DL734 = JCM 30395 = NCCB 100519.
*Mycolicibacterium sediminis* comb. nov. (se.di´mi.nis. L. gen. n. *sediminis*, of a sediment)	Basonym: *Mycobacterium sediminis* Zhang et al. 2013 The description of this taxon is as given by Zhang et al. (2013a). The type strain is YIM M13028 = DSM 45643 = KCTC 19999.
*Mycolicibacterium senegalense* comb. nov. (se.ne.gal.en´se. N.L. neut. adj. *senegalense*, of or belonging to the West African Republic of Senegal)	Basonym: *Mycobacterium senegalense* (Chamoiseau, 1973) Chamoiseau 1979 (Approved Lists 1980) (Skerman et al., 1980) The description of this taxon is as given by Chamoiseau (1973, 1979). The type strain is IEMVT 378 = ATCC 35796 = CCUG 21001 = CIP 104941 = DSM 43656 = JCM 15467 = NCTC 10956.
*Mycolicibacterium septicum* comb. nov. (sep´ti.cum. L. neut. adj. *septicum*, producing a putrefaction, putrefying, septic, referring to the isolation of the organism from blood)	Basonym: *Mycobacterium septicum* Schinsky et al. 2000 The description of this taxon is as given by Schinsky et al. (2000). The type strain is W4964 = ATCC 700731 = CCUG 43574 = CIP 106642 = DSM 44393 = JCM 14743.
*Mycolicibacterium setense* comb. nov. (se.ten´se. N.L. neut. adj. *setense*, pertaining to Sète (France), the city from which the infected patient originated)	Basonym: *Mycobacterium setense* Lamy et al. 2008 The description of this taxon is as given by Lamy et al. (2008). The type strain is ABO-M06 = CIP 109395 = DSM 45070 = JCM 15660.
*Mycolicibacterium smegmatis* comb. nov. (smeg.ma´tis. L. n. *smegma*-*atis*, an unguent (for making the skin smooth), a detergent, a cleansing medicine, and in biology the sebaceous humor; L. gen. n. *smegmatis*, of smegma)	Basonym: *Mycobacterium smegmatis* (Trevisan, 1889) Lehmann and Neumann, 1899 (Approved Lists, 1980) (Lehmann and Neumann, 1899) The description of this taxon is as given by Trevisan (1889); Lehmann and Neumann (1899). The type strain is ATCC 19420 = CCUG 21002 = CCUG 21815 = CIP 104444 = DSM 43756 = JCM 5866 = JCM 6386 = NCTC 8159 = NRRL B-14616 = VKM Ac-1239.
*Mycolicibacterium sphagni* comb. nov. (sphag´ni. N.L. n. *Sphagnum*, generic name of the moss of sphagnum bogs, the habitat of these strains; N.L. gen. n. *sphagni*, of *Sphagnum*)	Basonym: *Mycobacterium sphagni* Kazda 1980 The description of this taxon is as given by Kazda (1980). The type strain is Sph 38 = ATCC 33027 = DSM 44076.
*Mycolicibacterium thermoresistibile* comb. nov. (ther.mo.re.sis.ti´bi.le. Gr. n. *thermê*, heat; L. v. *resisto*, to stand back, remain standing, endure; L. neut. suff. -*ile*, suffix denoting an active quality, able to; N.L. neut. adj. *thermoresistibile*, able to resist to high temperature)	Basonym: *Mycobacterium thermoresistibile* Tsukamura 1966 (Approved Lists 1980) (Skerman et al., 1980) The description of this taxon is as given by Tsukamura (1966). The type strain is ATCC 19527 = CCUG 28008 = CCUG 41353 = CIP 105390 = DSM 44167 = JCM 6362 = NCTC 10409.
*Mycolicibacterium tokaiense* comb. nov. (to.kai.en´se. N.L. neut. adj. *tokaiense*, of or belonging to Tokai district of Japan)	Basonym: *Mycobacterium tokaiense* (*ex* Tsukamura et al. 1973) Tsukamura 1981 The description of this taxon is as given by Tsukamura et al. (1981). The type strain is 47503 (previously, strain 5553) = ATCC 27282 = CIP 106807 = DSM 44635 = JCM 6373 = NCTC 10821.
*Mycolicibacterium tusciae* comb. nov. (tus´ci.ae. L. gen. n. *tusciae*, of Tuscia (the country of the Tuscans), now Tuscany, the Italian region where all the organisms were isolated)	Basonym: *Mycobacterium tusciae* Tortoli et al., 1999 The description of this taxon is as given by Tortoli et al. (1999). The type strain is FI-25796 = CCUG 50996 = CIP 106367 = DSM 44338 = JCM 12692.
*Mycolicibacterium vaccae* comb. nov. (vac´cae. L. gen. n. *vaccae*, of a cow)	Basonym: *Mycobacterium vaccae* Bönicke and Juhasz 1964 (Approved Lists 1980) (Skerman et al., 1980) The description of this taxon is as given by Bonicke and Juhasz (1964). The type strain is ATCC 15483 = CCUG 21003 = CIP 105934 = DSM 43292 = HAMBI 2276 = IFO (now NBRC) 14118 = JCM 6389 = NCTC 10916.
*Mycolicibacterium vanbaalenii* comb. nov. (van.baa.len´i.i. N.L. gen. masc. n. *vanbaalenii*, of Van Baalen, in memory of Chase Van Baalen, late Professor at The University of Texas Marine Science Institute, Port Aransas Marine Laboratory, Port Aransas, Texas, USA)	Basonym: *Mycobacterium vanbaalenii* Khan et al. 2002 The description of this taxon is as given by Khan et al. (2002). The type strain is PYR-1 = DSM 7251 = JCM 13017 = NRRL B-24157.
*Mycolicibacterium vulneris* comb. nov. (vul´ne.ris. L. gen. n. *vulneris*, of a wound, from which the type strain was isolated)	Basonym: *Mycobacterium vulneris* van Ingen et al. 2009 The description of this taxon is as given by van Ingen et al. (2009). The type strain is NLA000700772 = CIP 109859 = DSM 45247 = JCM 18115.
*Mycolicibacterium wolinskyi* comb. nov. (wo.lins´ky.i. N.L. masc. gen. n. *wolinskyi*, of Wolinsky, named for Emanuel Wolinsky for his significant contributions to the study of non-tuberculous mycobacteria)	Basonym: *Mycobacterium wolinskyi* Brown et al. 1999 The description of this taxon is as given by Brown et al. (1999). The type strain is MO739 = ATCC 700010 = CCUG 47168 = CIP 106348 = DSM 44493 = JCM 13393.

In addition to the new name combinations for species which are part of this genus, we also provide below description of two new species that should also be placed in the genus *Mycolicibacterium*.

**Description of *Mycolicibacterium acapulense* sp. nov**. (a.ce.pul.cen´se. N.L. neut. adj. *acapulcense* from Acapulco, a town on the Pacific coast of México).

The description of this taxon is as given by Bojalil et al. (1962) for “*Mycobacterium acapulensis”*. The type strain is AC-103 (= ATCC 14473 = JCM 6402).

**Description of *Mycolicibacterium komanii* sp. nov**. (ko.ma´ni.i. N.L. gen. n. *komanii* named after a town in South Africa where one of the isolates originated from, Komani is the Xhosa name for Queenstown (South Africa)).

The description of this taxon is as given by Gcebe (2015) and Gcebe et al. (2016) for “*Mycobacterium komanii”*. The type strain is GPK 1020.

## Author contributions

RG was responsible for conceiving the idea of this study, carried out phylogenomic and other analyses reported here, supervised and directed the entire project and obtained funds for carrying out these studies. Involved in the writing and finalizing of the manuscript and all presented data. BL and JS were responsible for analysis and organization of the comparative genomic data on identification of described molecular signatures, under the direction of RG. They also helped in the preparation of a draft version of the manuscript.

### Conflict of interest statement

The authors declare that the research was conducted in the absence of any commercial or financial relationships that could be construed as a potential conflict of interest.
