# Corrigendum: Phylogenomics and Comparative Genomic Studies Robustly Support Division of the Genus *Mycobacterium* into an Emended Genus *Mycobacterium* and Four Novel Genera

**DOI:** 10.3389/fmicb.2019.00714

**Published:** 2019-04-09

**Authors:** Radhey S. Gupta, Brian Lo, Jeen Son

**Affiliations:** Department of Biochemistry and Biomedical Sciences, McMaster University, Hamilton, CA, Canada

**Keywords:** *Mycobacterium* classification, slow-growing and fast-growing mycobacteria, conserved signature indels and signature proteins, phylogenomic analysis, fortuitum-vaccae clade, abscessus-chelonae clade, terrae clade, triviale clade

In the original article, there was an error. Based on the branching position of *Mycobacterium vulneris* (van Ingen et al., 2009) in different phylogenomic trees and on multiple identified molecular signatures that this species shared with a clade of rapid growing mycobacteria, we proposed a reclassification of *M. vulneris*, into a new genus, *Mycolicibacterium*, corresponding to a clade of rapid-growing mycobacteria. However, it was noted in our article that the branching of *M. vulneris*, which is a slow-growing species with rapid-growing mycobacteria, was anomalous.

In a Frontiers commentary, Tortoli (2018) indicated that the genome sequence of *M. vulneris*, originally available in the NCBI genome database (accession CCBG00000000; Croce et al., 2014), was mislabeled and very likely corresponded to *Mycobacterium porcinum* (a rapid grower). Tortoli (2018) also reported the sequencing of the type strain of *M. vulneris*, DSM 45247^T^ and this genome sequence (accession NCXM01000000) showed the branching of *M. vulneris* within the slow-growing group of mycobacteria, belonging to the genus *Mycobacterium*.

Our own analysis with this new genome sequence also confirms the branching of *M. vulneris* within the delimited genus *Mycobacterium*, encompassing different slow-growing mycobacteria. As a result, the transfer of *M. vulneris* into the genus *Mycolicibacterium* as proposed in Table 11 of our article was incorrect as a direct result of the mislabeling of the available genome sequence for this species. To correct this error, we propose that the species *Mycolicibacterium vulneris* (Gupta et al., 2018) should be reinstated to its previous basonym *Mycobacterium vulneris* (van Ingen et al., 2009) and as part of the genus *Mycobacterium* (Gupta et al., 2018).

The authors apologize for this error and state that this does not change the scientific conclusions of the article in any way.
