# Supplementary material for: Phylogenomics and Comparative Genomic Studies Robustly Support Division of the Genus Mycobacterium into an Emended Genus Mycobacterium and Four Novel Genera
Source: Front Microbiol. 2018 Feb 13;9:67. doi: 10.3389/fmicb.2018.00067 (PMC5819568; doi:10.3389/fmicb.2018.00067)
Supplement: Supplementary file 1 [file Table1.PDF]

Supplementary Table 1

Genome characteristics of the sequenced members of the genus *Mycobacterium*.

Clade	Organism	Type	BioProject	Size (Mb)	GC%	Proteins	Level
"Abscessus-Chelonae" Clade	*Mycobacterium abscessus* ATCC 19977	Y	PRJNA15691	5.09	64.1	4942	Complete Genome
	Mycobacterium abscessus subsp. *bolletii* MM1513	N	PRJNA260833	4.50	64.5	4297	Complete Genome
	Mycobacterium chelonae ATCC 35752	Y	PRJNA251569	4.90	63.9	4656	Chromosome
	Mycobacterium franklinii DSM 45524	Y	PRJNA354248	5.44	64.1	5235	Contigs (61)
	Mycobacterium immunogenum CCUG 47286	Y	PRJNA284839	5.57	64.3	5333	Complete Genome
	Mycobacterium salmoniphilum D16Q15	N	PRJNA323571	4.85	64.15	4606	Contigs (16)
	Mycobacterium saopaulense CCUG 66554	Y	PRJNA347845	5.20	64.6	5130	Contigs (103)
"Fortuitum-Vaccae" Clade	*Mycobacterium fortuitum* CT6	N	PRJNA280545	6.25	66.2	5855	Complete Genome
	Mycobacterium vaccae 95051	N	PRJNA210723	6.24	68.6	5723	Complete Genome
	“Mycobacterium acapulcensis” CSURP1424	n/a	PRJEB14254	5.29	66.7	4740	Scaffold
	Mycobacterium aromaticivorans JS19b1=JCM16368	Y	PRJNA232438	6.30	66.4	5830	Contigs (6)
	Mycobacterium aurum	N	PRJEB8229	6.02	67.5	5518	Scaffold
	Mycobacterium austroafricanum DSM 44191	Y	PRJEB5747	6.77	67.7	6140	Scaffold
	Mycobacterium bacteremicum DSM 45578	Y	PRJNA354248	5.95	68.1	5603	Contigs (85)
	Mycobacterium brisbanense strain JCM15654	Y	PRJDB4227	7.39	66.6	6913	Scaffold
	Mycobacterium boenickei CIP107829	N	PRJEB19486	6.51	66.8	6012	Contigs (14)
	Mycobacterium canariasense JCM 15298	Y	PRJDB4227	6.73	67.6	6449	Scaffold
	Mycobacterium celeriflavum DSM 46765	Y	PRJNA354248	4.95	66.9	4695	Contigs (129)
	Mycobacterium chlorophenolicum DSM 43826	Y	PRJNA274376	7.38	68.4	6686	Scaffold
	Mycobacterium chubuense NBB4	N	PRJNA53215	6.34	68.3	5740	Complete Genome
	Mycobacterium conceptionense D16	Y	PRJEB5740	6.39	66.3	7093	Scaffold
	Mycobacterium confluentis DSM 44017	Y	PRJNA299467	5.84	67.5	5291	Contigs (57)
	Mycobacterium cosmeticum DSM 44829	N	PRJEB5748	6.46	68.2	6281	Contigs (5)
	Mycobacterium diernhoferi BM1	N	PRJNA338014	5.92	67.8	5,834	Scaffold
	Mycobacterium doricum DSM 44339	Y	PRJNA299467	3.95	67.6	3542	Contigs (74)
	Mycobacterium elephantis Lipa Mele	N	PRJNA282888	5.19	67.8	4865	Contigs (234)
	Mycobacterium fallax DSM 44179	Y	PRJNA299467	4.23	70.3	3814	Contigs (84)
	Mycobacterium farcinogenes DSM 43637	Y	PRJEB5746	6.14	66.6	5545	Scaffold
	Mycobacterium flavescens M6	N	PRJNA338014	5.97	68.5	5584	Scaffold
	Mycobacterium gilvum Spyr1	N	PRJNA28521	5.55	67.9	5077	Complete Genome
	Mycobacterium goodii X7B	N	PRJNA288832	7.11	67.6	6459	Complete Genome
	Mycobacterium hassiacum DSM 44199	Y	PRJNA165409	5.08	69.4	4632	Scaffold
	Mycobacterium holsaticum strain M7	N	PRJNA338014	5.75	67.0	5158	Contigs (353)
	Mycobacterium houstonense ATCC 49403	Y	PRJEB13221	6.38	66.9	5691	Contigs (197)
	Mycobacterium insubricum DSM 45130	N	PRJNA354248	4.55	68.8	4201	Contigs (157)
	Mycobacterium iranicum UM_TJL	N	PRJNA213255	6.14	66.1	5663	Scaffold
	“Mycobacterium komanii” sp. GPK 1020	N	PRJEB9486	5.38	67.3	5140	Contigs (65)
	Mycobacterium litorale strain F4	Y	PRJNA374925	6.1	67.4	5231	Complete Genome
	Mycobacterium llatzerense CLUC14	N	PRJNA273763	6.09	66.5	5407	Contigs (103)
	Mycobacterium mageritense DSM 44476	Y	PRJEB5749	7.97	67.0	7675	Contigs (6)
	Mycobacterium malmesburyense sp. WCM 7299	Y	PRJEB9485	5.47	67.3	5271	Scaffold
	Mycobacterium monacense DSM 44395	Y	PRJNA354248	6.00	68.4	5665	Contigs (106)
	Mycobacterium moriokaense CIP105393	Y	PRJNA354248	6.22	66.0	5973	Contigs (229)
	Mycobacterium mucogenicum CSUR P2099	N	PRJEB10728	6.21	67.2	5857	Contigs (7)
	Mycobacterium neoaurum VKM Ac-1815D	N	PRJNA177066	5.42	66.9	4948	Complete Genome
	Mycobacterium neworleansense ATCC 49404	Y	PRJEB9831	6.29	66.9	5997	Contigs (9)
	Mycobacterium novocastrense JCM 18114	Y	PRJDB4227	6.23	66.8	5815	Scaffold
	Mycobacterium obuense UC1	N	PRJNA279894	6.38	67.6	5809	Scaffold
	Mycobacterium parafortuitum CCUG 20999	Y	PRJNA354248	6.14	68.5	5683	Contigs (88)
	Mycobacterium peregrinum CSUR P2098	N	PRJEB10727	7.11	66.2	6528	Scaffold
	Mycobacterium phlei CCUG 21000	Y	PRJNA311273	5.35	69.4	4950	Complete Genome
	Mycobacterium porcinum HMC1	N	PRJNA338014	6.24	66.8	5866	Scaffold

	Mycobacterium rhodesiae NBB3	N	PRJNA60027	6.42	65.5	6044	Complete Genome
	Mycobacterium rufum JS14	Y	PRJNA247390	6.18	69.3	5443	Contigs (4)
	Mycobacterium rutilum DSM 45405	N	PRJEB15815	5.99	68.4	5729	Chromosome
	Mycobacterium senegalense CK2	N	PRJNA285366	6.86	66.4	6435	Scaffold
	Mycobacterium septicum DSM 44393	Y	PRJEB4006	6.91	66.7	6203	Scaffold
	Mycobacterium setense DSM 45070	Y	PRJNA268140	6.27	66.4	5597	Contigs (21)
	Mycobacterium smegmatis NCTC8159	Y	PRJEB6403	6.98	67.3	6604	Complete Genome
	Mycobacterium thermoresistibile JCM 6362	Y	PRJDB4227	4.89	69.0	4424	Scaffold
	Mycobacterium tusciae JS617	N	PRJNA52639	7.31	65.4	7013	Scaffold
	Mycobacterium vanbaalenii PYR-1	Y	PRJNA15761	6.49	67.8	5963	complete Genome
	Mycobacterium vulneris DSM 45247	Y	PRJEB5750	6.98	66.7	6653	Contigs (4)
	Mycobacterium wolinskyi CDC_01	N	PRJNA296779	7.45	66.5	6901	Contigs (76)
"Tuberculosis-Simiae" Clade	*Mycobacterium tuberculosis* H37Rv	Y	PRJNA224	4.41	65.6	3906	Complete Genome
	Mycobacterium simiae MO323	N	PRJNA276839	5.94	66.3	5326	Chromosome
	Mycobacterium africanum GM041182	N	PRJEA68095	4.39	65.6	3970	Complete Genome
	Mycobacterium alsense DSM 45230	Y	PRJNA354248	5.69	69.3	5206	Contigs (157)
	Mycobacterium angelicum DSM 45057	Y	PRJNA354248	6.66	66.3	5865	Contigs (525)
	Mycobacterium arosiense ATCC BAA-1401	Y	PRJNA354248	5.98	66.8	5579	Contigs (465)
	Mycobacterium asiaticum DSM 44297	Y	PRJEB5743	5.94	66.2	5150	Scaffold
	Mycobacterium avium 104	N	PRJNA88	5.48	69.0	4289	Complete Genome
	“Mycobacterium avium subsp. hominissuis” TH135	N	PRJDB502	4.95	69.3	4476	Complete Genome
	Mycobacterium avium subsp. paratuberculosis K-10	N	PRJNA91	4.83	69.3	4289	Complete Genome
	Mycobacterium avium subsp. silvaticum ATCC 49884	Y	PRJNA219418	4.71	69.2	4259	Contigs (808)
	Mycobacterium bohemicum DSM 44277	Y	PRJNA299467	5.42	69.2	4760	Contigs (52)
	Mycobacterium bouchedurhoenense DSM 45439	Y	PRJNA354248	5.90	68.6	5434	Contigs (230)
	Mycobacterium bovis AF2122 97	N	PRJNA89	4.35	65.6	3918	Complete Genome
	Mycobacterium branderi DSM 44624	Y	PRJNA354248	5.90	66.5	5553	Contigs (63)
	“Mycobacterium canettii” CIPT 140010059	N	PRJEA68135	4.48	65.6	3999	Complete Genome
	Mycobacterium caprae MB2	N	PRJEB7271	4.29	65.5	3768	Scaffold
	Mycobacterium celatum ATCC 51131	Y	PRJDB3515	4.66	66.8	3893	Contigs (1217)
	Mycobacterium chimaera MCIMRL2	N	PRJNA294775	6.09	67.7	5419	Contigs (247)
	Mycobacterium colombiense CECT 3035	Y	PRJNA67689	5.58	68.1	5197	Contigs (17)
	Mycobacterium conspicuum DSM 44136	Y	PRJNA299467	6.20	67.4	5457	Contigs (92)
	Mycobacterium europauem DSM 45397	Y	PRJNA299467	5.63	68.5	5060	Contigs (71)
	Mycobacterium florentinum DSM 44852	Y	PRJNA299467	6.18	66.4	5631	Contigs (34)
	Mycobacterium fragae DSM 45731	Y	PRJNA299467	4.73	66.0	4363	Contigs (35)
	Mycobacterium gastri 'Wayne'	N	PRJNA231757	6.00	66.2	5706	Contigs (635)
	Mycobacterium genavense ATCC 51234	Y	PRJNA223121	4.94	66.9	3558	Contigs (25)
	Mycobacterium gordonae HMC_M15	N	PRJNA338014	7.29	66.6	6109	Scaffold
	Mycobacterium haemophlium DSM 44634	Y	PRJNA171821	4.24	63.9	3730	Complete Genome
	Mycobacterium heckeshornense RLE	N	PRJNA288073	5.01	65.9	4515	Contigs (191)
	Mycobacterium heidelbergense DMS 44471	Y	PRJNA354248	5.00	68.0	4478	Contigs (110)
	“Mycobacterium indicus pranii” MTCC 9506	n/a	PRJNA33469	5.59	68.0	5020	Complete Genome
	Mycobacterium interjectum ATCC 51457	Y	PRJEB13236	5.85	67.9	5073	Contigs (221)
	Mycobacterium intermedium strain HMC2_M5	N	PRJNA338014	6.86	65.8	5667	Scaffold
	Mycobacterium intracellulare ATCC 13950	Y	PRJNA82157	5.40	68.1	4911	Complete Genome
	Mycobacterium kansasii ATCC 12478	Y	PRJNA30907	6.58	66.2	5696	Complete Genome
	Mycobacterium kubicae strain CIP 106428	Y	PRJNA299467	5.83	66.0	5162	Contigs (116)
	Mycobacterium kyorinense KUM 060204	Y	PRJDB3036	5.30	66.9	4700	Contigs (453)
	Mycobacterium lacus DSM 44577	Y	PRJNA299467	4.91	66.9	4203	Contigs (187)
	Mycobacterium lentiflavum CSUR P1491	N	PRJEB8430	6.82	65.7	6354	Contigs (5)
	Mycobacterium leprae Br4923	n/a	PRJEA31271	3.27	57.8	2251	Complete Genome
	“Mycobacterium lepromatosis” Mx1-22A	n/a	PRJNA254202	3.21	57.9	2181	Scaffold
	“Mycobacterium liflandii” 128FXT	N	PRJNA20227	6.21	65.6	4826	Complete Genome
	Mycobacterium malmoense E614	N	PRJNA305922	5.81	68.8	5171	Contigs (282)

	Mycobacterium mantenii DSM 45255	Y	PRJNA354248	6.12	66.9	5543	Contigs (157)
	Mycobacterium marinum M	N	PRJNA16725	6.66	65.7	5426	Complete Genome
	Mycobacterium marseillense DSM 45437	Y	PRJNA354248	5.46	67.7	5041	Contigs (112)
	Mycobacterium microti strain 12	N	PRJNA270004	4.37	65.6	4321	Complete Genome
	"Mycobacterium mungi" BM22813	N	PRJNA320514	4.35	65.5	3901	Contigs (110)
	Mycobacterium nebraskense AKUC2	N	PRJNA284996	6.54	66.6	5802	Scaffold
	Mycobacterium noviomagense DSM 45145	Y	PRJNA354248	4.74	65.7	4411	Contigs (227)
	"Mycobacterium orygis" 112400015	N	PRJNA193095	4.28	65.6	4001	Contigs (108)
	Mycobacterium palustre DSM 44572	Y	PRJNA299467	6.04	68.5	5344	Contigs (158)
	Mycobacterium paraense IEC26	Y	PRJNA308282	5.62	69.3	5131	Contigs (51)
	Mycobacterium paraffinicum strain M11	N	PRJNA338014	6.48	67.6	5876	Scaffold
	Mycobacterium paraintracellulare MOTT-64	Y	PRJNA82155	5.49	68.1	5078	Complete Genome
	Mycobacterium parascrofulaceum ATCC BAA-614	Y	PRJNA31521	6.56	68.5	5586	Scaffold
	Mycobacterium paraseoulense DSM 45000	Y	PRJNA354248	6.08	67.9	5694	Contigs (267)
	Mycobacterium parmense DSM 44553	Y	PRJNA299467	5.89	68.4	5168	Contigs (91)
	Mycobacterium pseudoshottsii L15 JCM 15466 T	Y	PRJDB4345	5.94	65.7	5719	Scaffold
	Mycobacterium riyadhense DSM 45176	Y	PRJNA299467	6.27	65.3	5168	Contigs (263)
	Mycobacterium saskatchewanense DSM 44616	Y	PRJNA299467	5.93	68.3	5297	Contigs (87)
	Mycobacterium scrofulaceum E3039	N	PRJNA305922	5.54	68.4	4945	Contigs (183)
	Mycobacterium sherrisii strain BC1_M4	N	PRJNA338014	5.69	66.9	5019	Scaffold
	Mycobacterium shimoidei DSM 44152	Y	PRJNA299467	4.71	65.8	4307	Contigs (71)
	Mycobacterium shinjukuense CCUG 53584	Y	PRJNA354248	4.41	67.8	3834	Contigs (180)
	Mycobacterium szulgai ACS1160	N	PRJNA305922	5.62	66.0	5035	Contigs (54)
	Mycobacterium timonense CCUG 56329	Y	PRJNA354248	6.01	68.5	5965	Contigs (1217)
	Mycobacterium triplex DSM 44626	Y	PRJEB5744	6.38	66.6	5768	Scaffold
	Mycobacterium ulcerans Agy99	N	PRJNA16230	5.81	65.5	4160	Complete Genome
	Mycobacterium xenopi RIVM700367	N	PRJNA84345	4.43	66.1	4281	Contigs (117)
	Mycobacterium yongonense 05-1390	Y	PRJNA82265	5.66	67.9	5056	Complete Genome
	Mycobacterium terrae CIP 104321	Y	PRJNA299467	4.52	68.4	4086	Contigs (47)
	Mycobacterium algericum DSM 45454	Y	PRJNA354248	4.62	68.3	4306	Contigs (107)
	Mycobacterium arupense GUC1 Marup	N	PRJNA280139	4.44	67.3	3941	Contigs (173)
	Mycobacterium engbaekii ATCC 27353	Y	PRJNA299467	4.52	68.5	3978	Contigs (79)
	Mycobacterium heraklionense Davo	N	PRJNA285002	5.11	67.9	4578	Scaffold
	Mycobacterium hiberniae ATCC 49874	Y	PRJNA299467	4.34	68.5	3897	Contigs (48)
"Terrae" Clade	"Mycobacterium icosiumassiliensis" 8WA6	Y[1]	PRJEB13234	4.84	66.8	4283	Contigs (79)
	Mycobacterium kumamotonense Roo	N	PRJNA288076	5.33	67.7	4851	Scaffold
	Mycobacterium longobardum DSM 45394	Y	PRJNA299467	4.81	67.9	4435	Contigs (59)
	Mycobacterium minnesotense DSM 45633	Y	PRJNA354248	4.19	67.1	3778	Contigs (60)
	Mycobacterium nonchromogenicum DSM 44164	Y	PRJNA299467	4.47	67.8	3988	Contigs (91)
	Mycobacterium senuense DSM 44999	Y	PRJNA299467	4.53	68.7	4106	Contigs (54)
	"Mycobacterium sinense" JDM601	Y	PRJNA51513	4.64	68.4	4251	Complete Genome
"Triviale" Clade	*Mycobacterium koreense* KCTC 19819	Y	PRJNA354248	4.08	69.4	3862	Contigs (129)
	Mycobacterium triviale DSM 44153	Y	PRJNA299467	3.59	70.3	3290	Contigs (57)

[1] PMID: 27154465
