# Supplementary material for: Phylogenomics and Comparative Genomic Studies Robustly Support Division of the Genus Mycobacterium into an Emended Genus Mycobacterium and Four Novel Genera
Source: Front Microbiol. 2018 Feb 13;9:67. doi: 10.3389/fmicb.2018.00067 (PMC5819568; doi:10.3389/fmicb.2018.00067)
Supplement: Supplementary file 2 [file Table2.PDF]

Supplementary Table 2

Protein families used in the housekeeping genes based phylogenetic tree (Supplementary Figure 2).

**Protein Family Name**	**UniProtKB ID**
DNA-directed RNA polymerase subunit A (*rpoA*)	P9WGZ1
DNA-directed RNA polymerase subunit beta (*rpoB*)	P9WGY9
DNA-directed RNA polymerase subunit beta' (*rpoC*)	P9WGY7
60 KDa chaperonin 2 (*hsp65*)	P9WPE7
DNA gyrase subunit A (*gyrA*)	P9WG47
DNA gyrase subunit B (*gyrB*)	P9WG45
Protein RecA (*recA*)	P9WHJ3
Elongation factor Tu (*tufA*)	P9WNN1

Protein families used in the phyloeco markers based phylogenetic tree (Figure 1B).

**Protein Family Name**	**UniProtKB ID**
ATP synthase F1 subcomplex gamma subunit (*atpG)*	P9WPU9
Uridylate kinase (*pyrH)*	P9WHK5
Hypoxanthine phosphoribosyltransferase (*hpt*)	P9WHQ9
RecG-like helicase (*recG*)	P9WMQ7
Signal recognition particle subunit SRP54 (*ffh*)	P9WGD7
LSU ribosomal protein L1 (*rplA*)	P9WHC7
DNA replication and repair protein *(RecF)*	P9WHI9
ATP synthase F1 subcomplex alpha subunit (*atpA*)	P9WPU7
FMN adenylyltransferase/riboflavin kinase (*ribF*)	I6X5C9
2C-methyl-D-erythritol 2 4-cyclodiphosphate synthase (*ispG*)	P9WKG3
Holliday junction DNA helicase subunit (*ruvA*)	P9WGW3
Conserved hypothetical protein TIGR01033 (*Rv2603c*)	P9WGA5
UDP-N-acetylmuramate--alanine ligase (*murC*)	P9WJL7
DNA polymerase III subunits gamma and tau (*dnaX*)	P9WNT9
Translation initiation factor IF-3 (*infC*)	P9WKJ9
tRNA pseudouridine synthase A (*truA*)	P9WHP9
ribosomal protein L3 (*rplC*)	P9WH87
Exodeoxyribonuclease VII (*xseAB*)	P9WF31/ P9WF29
Recombination protein (*RecR*)	P9WHI3
Transcription-repair coupling factor (*mfd*)	P9WMQ5
Ribosomal protein S20 (*rpsT*)	P9WH41
Ribosomal protein S15 (*rpsO*)	P9WH55
16S rRNA processing protein *(RimM*)	P9WH19
Ribosomal protein L23 (*rplW*)	P9WHB9
Ribosomal protein S12 (*rpsL*)	P9WH63
Ribosomal protein L5 (*rplE*)	P9WH83
cell division (*FtsK*/*SpoIIIE*)	P9WNA3
ribonuclease III (*rnc*)	P9WH03

chromosomal replication initiator protein (*dnaA*)	P9WNW3
DNA-directed RNA polymerase beta' subunit (*rpoC*)	P9WGY7
ribosomal protein S16 (*rpsP*)	P9WH53
Leucyl-tRNA synthetase (*leuS*)	P9WFV1
ribosomal protein L1 (*rplA*)	P9WHC7
ribosomal protein S6 (*rpsF*)	P9WH31
Phosphoribosylformylglycinamidine synthase (*purQ*)	P9WHL5
ParB-like partition protein (*parB*)	P9WIJ9
30S ribosomal protein S8 (*rpsH*)	P9WH27
UDP-N-acetylglucosamine pyrophosphorylase (*glmU*)	P9WMN3
F0F1 ATP synthase subunit beta (*atpD*)	P9WPU5
deoxyuridine 5-triphosphate nucleotidohydrolase (*dut*)	P9WNS5
ribosome recycling factor (*frr*)	P9WGY1
50S ribosomal protein L19 (*rplS*)	P9WHC9
Excinuclease ABC subunit C (*uvrC*)	P9WFC5
50S ribosomal protein L27 (*rpmA*)	P9WHB3
Cell division protein(*ftsX*)	P9WG19
phosphomannomutase (*glmM*)	P9WN41
Holliday junction resolvase (*ruvC*)	P9WGV9
DNA repair protein (*recN*)	P9WHI7
30S ribosomal protein S13 (*rpsM*)	P9WH61
guanylate kinase (*gmk*)	P9WKE9
tyrosyl-tRNA synthetase (*tyrS*)	P9WFT1
Transcriptional repressor (*nrdR*)	P9WIZ1
Phosphoribosylamine--glycine ligase (*purD*)	P9WHM9
S-adenosylmethionine-dependent methyltransferase (*mraW*)	P9WJP1
phenylalanyl-tRNA synthetase subunit alpha (*pheS*)	P9WFU3
Holliday junction DNA helicase B (*ruvB*)	P9WGW1
priA primosome assembly protein (*priA*)	P9WMQ9
sporulation transcription regulator (*whiA*)	P9WF45
tRNA pseudouridine synthase B (*truB*)	P9WHP7
ribosomal protein S10 (*rpsJ*)	P9WH67
30S ribosomal protein S19 (*rpsS*)	P9WH45
50S ribosomal protein L6 (*rplF*)	P9WH81
50S ribosomal protein L13 (*rplM*)	P9WHE1
polynucleotide phosphorylase polyadenylase (*pnp*)	P9WI57
elongation factor P (*efp*)	P9WNM3
putative iron-regulated ABC-type transporter (*sufB*)	P9WFP7
50S ribosomal protein L10 (*rplJ*)	P9WHE7
Holliday junction resolvase (*yqgF*)	P9WGV7
30S ribosomal protein S3 (*rpsC*)	P9WH37
transcription elongation factor (*nusA*)	P9WIV3
phosphoribosylaminoimidazole synthetase (*purM*)	I6Y4V6
amidophosphoribosyltransferase (*purF*)	P9WHQ7
dimethyladenosine transferase (*ksgA*)	P9WH07

Phosphatidate cytidylyltransferase (*cdsA*)	P9WPF7
tRNA delta 2 -isopentenylpyrophosphate transferase (*miaA*)	P9WJW1
3-5 exonuclease	L0TBY7
GTPase Obg (*obg)*	P9WMT1
50S ribosomal protein L21 (*rplU*)	P9WHC3
preprotein translocase subunit (*secY*)	P9WGN3
histidyl-tRNA synthetase (*hisS*)	P9WFV5
30S ribosomal protein S2 (*rpsB*)	P9WH39
SsrA-binding protein (*smpB*)	P9WGD3
DNA-directed RNA polymerase alpha subunit (*rpoA*)	P9WGZ1
DNA repair protein (*RecO*)	P9WHI5
Endoribonuclease UPF0054 (*ybeY*)	P9WGX9
ribosomal protein L18 (*rplR*)	P9WHD1
isoleucyl-tRNA synthetase (*ileS*)	P9WFV3
RecA recombinase (*recA*)	P9WHJ3
10 kDa chaperonin (*groES*)	P9WPE5
excinuclease ABC B subunit (*uvrB*)	P9WFC7
cell division protein (*ftsZ*)	P9WN95
putative translation elongation factor Ts (*tsf*)	P9WNM1
30S ribosomal protein S9 (*rpsI*)	P9WH25
50S ribosomal protein L17 (*rplQ)*	P9WHD3
50S ribosomal protein L2 (*rplB*)	P9WHA5
DNA polymerase III delta subunit	A0A089QT28
CTP synthetase (*pyrG*)	P9WHK7
bifunctional cytidylate kinase GTP-binding protein (*cmk*)	P9WPA9
50S ribosomal protein L24 (*rplX*)	P9WHB7
translation-associated GTPase (*ychF*)	O53459
Conserved hypothetical protein 95	I6XFS7
DNA primase (*dnaG*)	P9WNW1
30S ribosomal protein S1 (*rpsA*)	P9WH43
phosphoglycerate kinase (*pgk*)	P9WID1
50S ribosomal protein L15 (*rplO*)	P9WHD7
30S ribosomal protein S7 (*rpsG*)	P9WH29
50S ribosomal protein L22 (*rplV*)	P9WHC1
50S ribosomal protein L16 (*rplP*)	P9WHD5
50S ribosomal protein L35 (*rpmI*)	P9WH91
GTP-binding protein (*lepA*)	P9WK97
DNA topoisomerase I (*topA*)	P9WG49
transcription antitermination protein (*nusG*)	P9WIU9
50S ribosomal protein L7/L12 (*rplL*)	P9WHE3
50S ribosomal protein L9 (*rplI*)	P9WH79
phosphoribosylaminoimidazole carboxylase catalytic subunit (*purE*)	P9WHM1
aspartyl glutamyl-tRNA amidotransferase subunit B (*gatB*)	P9WN61
30S ribosomal protein S17 (*rpsQ*)	P9WH51
1-deoxy-D-xylulose 5-phosphate reductoisomerase (*dxr*)	P9WNS1

LSU ribosomal protein L20P (*rplT*)	P9WHC5
chorismate synthase (*aroC*)	P9WPY1
LSU ribosomal protein L4P (*rplD*)	P9WH85
homoserine dehydrogenase (*hom*)	P9WPX1
preprotein translocase SecG subunit (*secG*)	P9WGN5
dephospho-CoA kinase unknown domain fusion protein (coaE)	P9WPA3
tRNA guanine-N 1 - -methyltransferase (*trmD*)	P9WFY7
tRNA N6-adenosine threonylcarbamoyltransferase	P9WHT7
30S ribosomal protein S11 (*rpsK*)	P9WH65
F0F1 ATP synthase subunit A (*atpB*)	P9WPU5
50S ribosomal protein L14 (*rplN*)	P9WHD9
phosphopantetheine adenylyltransferase (*coaD*)	P9WPA5
metal dependent phosphohydrolase (*pcnA*)	L7N672
ribosomal protein S5 (*rpsE*)	P9WH33
UDP-N-acetylmuramoyl-L-alanyl-D-glutamate synthetase (*murD*)	P9WJL5
DNA-directed RNA polymerase subunit beta (rpoB)	P9WGY9
bifunctional phosphoribosylaminoimidazolecarboxamide formyltransferase IMP cyclohydrolase (*purH*)	P9WHM7
phenylalanyl-tRNA synthetase subunit beta (*pheT*)	P9WFU1

Protein families obtained from (Wang and Wu, 2013).
