# Supplementary material for: Phylogenomics and Comparative Genomic Studies Robustly Support Division of the Genus Mycobacterium into an Emended Genus Mycobacterium and Four Novel Genera
Source: Front Microbiol. 2018 Feb 13;9:67. doi: 10.3389/fmicb.2018.00067 (PMC5819568; doi:10.3389/fmicb.2018.00067)
Supplement: Supplementary file 3 [file Table3.PDF]

Supplementary Table 3

Selected phenotypic characteristics of most members of the genus *Mycobacterium*.

Clade	Organism	Growth Rate	Pathogenicity	Arylsulfatase (3 days)	Arylsulfatase (7 days)	Arylsulfatase (10 days)	Pigmentation	Nitrate Reduction	Iron Uptake	Catalase	Catalase (68°C)	Tween 80 Hydrolysis
"Abscessus-Chelonae" Clade	*Mycobacterium abscessus*	< 7 days	Pathogenic	+	+	nd	N	-	-	nd	nd	-
	Mycobacterium abscessus subsp. *abscessus*	< 7 days	Pathogenic	+	+	nd	N	-	-	nd	nd	-
	Mycobacterium abscessus subsp. *bolletii*	2-5 days	Pathogenic	+	+	nd	N	-	-	nd	nd	nd
	Mycobacterium abscessus subsp. *massiliense*	2-4 days	Pathogenic	+	+	nd	N	-	-	+	nd	nd
	Mycobacterium chelonae	3-4 days	Pathogenic	+	+	nd	N	-	-	d	nd	-
	Mycobacterium immunogenum	< 7 days	Pathogenic	+	+	nd	N	-	-	nd	nd	nd
	Mycobacterium salmoniphilum	4-6 days	Pathogenic	+	+	nd	N	-	nd	nd	nd	nd
	Mycobacterium franklinii	3-5 days	Nonpathogenic	nd	nd	nd	N	-	nd	nd	nd	-
	Mycobacterium saopaulense	3-5 days	Pathogenic	nd	nd	nd	N	-	nd	nd	nd	-
"Fortuitum-Vaccae" Clade	*Mycobacterium fortuitum*	2-4 days	Pathogenic	+	+	nd	N	+	+	nd	nd	nd
	Mycobacterium fortuitum subsp. *acetamidolyticum*	5 days	Generally nonpathogenic	+	+	nd	N	+	+	nd	nd	nd
	Mycobacterium fortuitum subsp. *fortuitum*	2-4 days	Pathogenic	+	+	nd	N	+	+	nd	nd	nd
	Mycobacterium agri	< 5 days	Nonpathogenic	d	nd	nd	N	+	-	+	+	+
	Mycobacterium aichiense	3-4 days	Nonpathogenic	+	+	nd	S	-	nd	-	nd	-
	Mycobacterium alvei	5 days	Generally nonpathogenic	+	+	nd	N	+	-	+	+	+

	Mycobacterium aromaticivorans	< 7 days	Nonpathogenic	nd	nd	nd	S	-	nd	+	nd	nd
	Mycobacterium aubagnense	2-5 days	Nonpathogenic	+	+	nd	N	-	-	nd	nd	nd
	Mycobacterium aurum	< 5 days	Generally nonpathogenic	-	nd	nd	S	-	nd	+	nd	+
	Mycobacterium austroafricanum	< 3 days	Generally nonpathogenic	+	+	nd	S	+	nd	nd	nd	d
	Mycobacterium boenickei	< 7 days	Pathogenic	d	nd	nd	N	+	nd	nd	+	nd
	Mycobacterium brisbanense	< 7 days	Pathogenic	+	+	nd	N	+	nd	nd	-	nd
	Mycobacterium brumae	< 5 days	Nonpathogenic	-	nd	nd	N	+	+	+	+	+
	Mycobacterium canariasense	2-3 days	Generally nonpathogenic	+	+	nd	N	-	-	nd	+	+
	Mycobacterium chitae	3-5 days	Nonpathogenic	-	+	nd	N	+	v	+	+	-
	Mycobacterium chlorophenolicum	4-8 days	Generally nonpathogenic	d	nd	nd	S	-	nd	nd	nd	nd
	Mycobacterium chubuense	3 days	Nonpathogenic	-	+	nd	S	+	nd	-	nd	-
	Mycobacterium conceptionense	2-5 days	Pathogenic	+	+	nd	N	+	+	+	nd	nd
	Mycobacterium confluentis	2-4 days	Nonpathogenic	-	-	nd	N	+	-	nd	+	-
	Mycobacterium cosmeticum	3 days	Pathogenic	-	nd	nd	S	+	+	nd	nd	nd
	Mycobacterium diernhoferi	3 days	Nonpathogenic	-	d	nd	N	+	nd	-	-	-
	Mycobacterium doricum	< 14 days	Generally nonpathogenic	-	nd	nd	S	+	nd	nd	nd	-
	Mycobacterium duvalii	< 7 days	Nonpathogenic	-	-	nd	S	+	nd	+	nd	+
	Mycobacterium elephantis	< 7 days	Nonpathogenic	-	-	nd	N	+	nd	+	+	+
	Mycobacterium fallax	< 5 days	Nonpathogenic	-	nd	nd	N	+	-	+	-	+

	Mycobacterium farcinogenes	1-2 days	Pathogenic	nd	nd	nd	N	+	-	-	nd	nd
	Mycobacterium flavescens	7-10 days	Generally nonpathogenic	nd	nd	nd	S	nd	nd	nd	nd	nd
	Mycobacterium fluoranthenivorans	< 7 days	Nonpathogenic	nd	nd	nd	N	-	nd	+	nd	-
	Mycobacterium frederiksbergense	5 days	Generally nonpathogenic	nd	nd	nd	S	+	nd	+	nd	+
	Mycobacterium gadium	3-4 days	Generally nonpathogenic	-	-	nd	S	+	nd	+	nd	-
	Mycobacterium gilvum	< 7 days	Nonpathogenic	+	+	nd	S	+	nd	+	nd	+
	Mycobacterium goodii	2-4 days	Pathogenic	-	nd	nd	N*	+	+	+	-	nd
	Mycobacterium hassiacum	2-3 dqays	Nonpathogenic	nd	nd	nd	S	-	-	+	+	-
	Mycobacterium hodleri	4-5 days	Nonpathogenic	nd	nd	nd	S	-	nd	+	nd	+
	Mycobacterium holsaticum	< 7 days	Nonpathogenic	-	nd	nd	v	+	-	-	-	d
	Mycobacterium houstonense	< 7 days	Pathogenic	+	+	nd	N	+	nd	nd	+	nd
	Mycobacterium iranicum	4 days	Pathogenic	+	nd	nd	S	-	+	nd	+	-
	Mycobacterium komossense	< 7 days	Generally nonpathogenic	-	nd	nd	S	-	+	nd	nd	+
	Mycobacterium llatzerense	3-4 days	Generally nonpathogenic	nd	nd	nd	N	-	nd	-	-	+
	Mycobacterium madagascariense	3-7 days	Nonpathogenic	+	+	nd	S	-	+	nd	nd	+
	Mycobacterium mageritense	2-4 days	Generally nonpathogenic	+	+	nd	N	d	+	nd	-	-
	Mycobacterium monacense	< 7 days	Generally nonpathogenic	-	nd	nd	S	+	nd	nd	+	+
	Mycobacterium montmartrense	3-4 days	Nonpathogenic	+	nd	nd	N	-	-	+	-	+
	Mycobacterium moriokaense	3 days	Nonpathogenic	+	+	nd	N	+	nd	-	nd	nd

	Mycobacterium mucogenicum	2-4 days	Pathogenic	+	+	nd	N	d	-	-	-	+
	Mycobacterium murale	2-4 days	Nonpathogenic	+	+	nd	S	-	nd	+	d	+
	Mycobacterium neoaurum	< 5 days	Pathogenic	-	nd	nd	S	-	nd	nd	nd	+
	Mycobacterium neworleansense	< 7 days	Pathogenic	+	+	nd	N	+	nd	nd	+	nd
	Mycobacterium novocastrense	3-7 days	Nonpathogenic	-	-	nd	P	+	-	+	nd	-
	Mycobacterium obuense	≤ 5 days	Nonpathogenic	+	+	nd	S	-	nd	-	nd	-
	Mycobacterium parafortuitum	3-4 days	Nonpathogenic	-	+	nd	P	d	nd	nd	nd	nd
	Mycobacterium peregrinum	7 days	Generally nonpathogenic	+	+	nd	N	+	+	+	+	v
	Mycobacterium phlei	2-5 days	Generally nonpathogenic	-	nd	nd	S	+	+	nd	nd	nd
	Mycobacterium phocaicum	2-5 days	Pathogenic	+	+	nd	N	+	-	d	nd	nd
	Mycobacterium porcinum	> 3 days	Pathogenic	+	+	nd	N	-	nd	+	nd	-
	Mycobacterium poriferae	4 days	Generally nonpathogenic	-	-	nd	S	-	+	+	+	+
	Mycobacterium psychrotolerans	2 days	Nonpathogenic	nd	nd	nd	S	+	nd	+	-	nd
	Mycobacterium pulveris	5 days	Nonpathogenic	nd	nd	nd	N	+	nd	nd	+	+
	Mycobacterium pyrenivorans	< 7 days	Nonpathogenic	nd	nd	nd	S	d	nd	+	nd	-
	Mycobacterium rhodesiae	< 5 days	Generally nonpathogenic	+	+	nd	S	-	nd	+	nd	+
	Mycobacterium rufum	< 7 days	Nonpathogenic	nd	nd	nd	S	+	nd	+	nd	nd
	Mycobacterium rutilum	< 7 days	Nonpathogenic	nd	nd	nd	P	-	nd	+	nd	nd
	Mycobacterium senegalense	1-2 days	Pathogenic	+	+	nd	N	+	-	+	nd	nd

	Mycobacterium septicum	< 7 dasy	Generally nonpathogenic	-	nd	nd	N	+	nd	nd	nd	nd
	Mycobacterium setense	2-4 days	Pathogenic	+	nd	nd	N	+	+	nd	+	nd
	Mycobacterium smegmatis	2-4 days	Pathogenic	-	nd	nd	N	+	+	nd	nd	nd
	Mycobacterium sphagni	3-7 days	Nonpathogenic	-	nd	nd	S	+	-	nd	nd	+
	Mycobacterium thermoresistible	3-5 days	Generally nonpathogenic	-	nd	nd	N	+	nd	+	nd	nd
	Mycobacterium tokaiense	< 5 days	Generally nonpathogenic	+	+	nd	S	-	nd	-	nd	-
	Mycobacterium tusciae	28 days	Nonpathogenic	v	v	d	S	+	nd	nd	+	+
	Mycobacterium vaccae	< 5 days	Generally nonpathogenic	d	+	nd	P	+	+	nd	nd	-
	Mycobacterium vanbaalenii	1-2 days	Nonpathogenic	+	+	nd	S	+	nd	+	nd	+
	Mycobacterium vulneris	< 28 days	Generally nonpathogenic	-	nd	nd	S	-	nd	nd	+	-
	Mycobacterium wolinskyi	2-4 days	Pathogenic	-	nd	nd	N	+	+	+	+	nd
"Tuberculosis-Simiae" Clade	*Mycobacterium tuberculosis*	< 1 day	Pathogenic	-	-	-	N	+	nd	nd	-	d
	Mycobacterium simiae	14- 21 days	Pathogenic	-	-	-	P	-	nd	+	+	-
	Mycobacterium alsense	> 14 days	Nonpathogenic	-	nd	nd	S	-	nd	nd	+	-
	Mycobacterium angelicum	14 days	Nonpathogenic	nd	nd	nd	N	+	nd	nd	-	-
	Mycobacterium arosiense	> 14 days	Generally nonpathogenic	nd	nd	nd	S	+	nd	nd	+	-
	Mycobacterium asiaticum	15-21 days	Pathogenic	nd	nd	+	P	-	nd	+	+	+
	Mycobacterium avium	> 7 days	Pathogenic	-	-	d	N	-	nd	nd	d	-
	Mycobacterium avium subsp. avium	> 7 days	Pathogenic	-	-	d	N	-	nd	nd	d	-

	Mycobacterium avium subsp. *paratuberculosis*	21-42 days	Pathogenic	-	-	-	N	-	nd	nd	d	-
	Mycobacterium avium subsp. *silvaticum*	≥ 14 days	Pathogenic	-	-	-	N	-	nd	nd	+	-
	Mycobacterium bohemicum	28-42 days	Generally nonpathogenic	-	-	-	S	-	nd	+	+	-
	Mycobacterium bovis	≥ 21 days	Pathogenic	-	-	d	N	-	nd	nd	-	-
	Mycobacterium branderi	14-21 days	Generally nonpathogenic	nd	nd	nd	N	-	nd	nd	nd	-
	Mycobacterium caprae	28-42 days	Pathogenic	-	-	-	N	-	nd	+	-	w
	Mycobacterium celatum	21-35 days	Pathogenic	+	+	+	N	-	nd	nd	+	-
	Mycobacterium chimaera	> 7 days	Pathogenic	-	nd	nd	N	-	nd	nd	+	-
	Mycobacterium colombiense	21 days	Pathogenic	v	v	v	N	v	nd	+	+	-
	Mycobacterium conspicuum	14-21 days	Generally nonpathogenic	+	+	+	S	-	nd	nd	+	+
	Mycobacterium florentinum	14 days	Generally nonpathogenic	-	-	-	N	v	nd	v	nd	-
	Mycobacterium gastri	≥ 7 days	Generally nonpathogenic	nd	nd	+	N	-	nd	nd	-	+
	Mycobacterium genavense	21-84 days	Pathogenic	-	-	-	N	-	nd	nd	+	-
	Mycobacterium gordonae	7 days	Generally nonpathogenic	-	nd	d	S	-	nd	nd	+	+
	Mycobacterium haemophilum	14-28 days	Pathogenic	-	nd	nd	N	-	nd	-	-	-
	Mycobacterium heckeshornense	28 days	Pathogenic	-	-	-	S	-	nd	nd	+	-
	Mycobacterium heidelbergense	21-28 days	Generally nonpathogenic	-	nd	nd	N	-	nd	-	+	+
	Mycobacterium interjectum	21-28 days	Generally nonpathogenic	-	nd	w	S	-	nd	+	+	-
	Mycobacterium intermedium	14-21 days	Generally nonpathogenic	+	+	+	P	-	nd	+	+	+

	Mycobacterium intracellulare	≥ 7 days	Generally nonpathogenic	nd	nd	+	N	-	nd	nd	+	-
	Mycobacterium kansasii	≥ 7 days	Generally nonpathogenic	nd	nd	+	P	+	nd	+	+	+
	Mycobacterium kubicae	21 days	Nonpathogenic	-	nd	nd	S	-	nd	nd	nd	+
	Mycobacterium lacus	14-28 days	Generally nonpathogenic	w	w	w	N	+	nd	nd	-	w
	Mycobacterium lentiflavum	21-28 days	Generally nonpathogenic	-	-	-	S	-	nd	nd	+	-
	Mycobacterium lepraemurium	28-35 days	Pathogenic	-	-	-	N	-	nd	+	-	-
	Mycobacterium malmoense	7-42 days	Pathogenic	-	-	-	N	-	nd	+	d	+
	Mycobacterium marinum	≥ 7 days	Pathogenic	-	nd	+	P	-	nd	nd	-	+
	Mycobacterium microti	28-60 days	Pathogenic	nd	nd	nd	N	d	nd	nd	-	nd
	Mycobacterium nebraskense	21-28 days	Pathogenic	-	nd	nd	S	-	nd	nd	+	v
	Mycobacterium palustre	14-35 days	Nonpathogenic	-	nd	d	S	d	nd	nd	nd	+
	Mycobacterium parascrofulaceum	14-28 days	Generally nonpathogenic	-	nd	nd	S	-	nd	nd	+	-
	Mycobacterium parmense	14 days	Nonpathogenic	-	nd	nd	S	-	nd	nd	+	+
	Mycobacterium pseudoshottsii	28-42 days	Pathogenic	-	nd	nd	P	-	nd	nd	d	-
	Mycobacterium saskatchewanense	14-28 days	Generally nonpathogenic	-	nd	nd	S	-	nd	nd	+	+
	Mycobacterium scrofulaceum	≥ 7 days	Generally nonpathogenic	-	nd	d	S	-	nd	+	+	-
	Mycobacterium shimoidei	14-21 days	Pathogenic	nd	nd	nd	N	-	nd	nd	+	+
	Mycobacterium szulgai	14 days	Generally nonpathogenic	nd	nd	d	S/P	+	nd	nd	+	d
	Mycobacterium triplex	14-21 days	Generally nonpathogenic	-	nd	nd	N	+	nd	nd	+	-

	Mycobacterium ulcerans	28 days	Pathogenic	-	nd	-	N/S	-	nd	nd	+	-
	Mycobacterium xenopi	≥ 14 days	Pathogenic	v	nd	+	S	-	nd	+	+	-
"Terrae" Clade	*Mycobacterium terrae*	≥7 days	Generally nonpathogenic	nd	nd	d	N	d	nd	nd	+	+
	Mycobacterium algericum	7-35 days	Nonpathogenic	nd	nd	nd	N	+	nd	+	nd	nd
	Mycobacterium arupense	5-7 days	Generally nonpathogenic	-	nd	nd	N	-	nd	nd	+	+
	Mycobacterium engbaekii	< 10 days	Nonpathogenic	-	nd	nd	P	-	nd	nd	+	+
	Mycobacterium heraklionense	5-12 days	Generally nonpathogenic	-	nd	nd	N	+	nd	nd	+	+
	Mycobacterium hiberniae	20 days	Nonpathogenic	nd	nd	+	S	+	nd	nd	nd	d
	*"*Mycobacterium icosiumassiliensis*"*	5 days	Nonpathogenic	nd	nd	nd	N	-	nd	+	+	-
	Mycobacterium kumamotonense	7-14 days	Generally nonpathogenic	-	nd	nd	N	+	nd	nd	nd	+
	Mycobacterium longobardum	7-14 days	Generally nonpathogenic	+	nd	nd	N	+	nd	nd	+	-
	Mycobacterium minnesotense	7-10 days	Nonpathogenic	-	nd	nd	P	-	nd	nd	+	+
	Mycobacterium nonchromogenicumn	≥ 7 days	Generally nonpathogenic	nd	nd	+	N	-	nd	nd	+	+
	Mycobacterium paraterrae	≥ 28 days	Generally nonpathogenic	-	nd	nd	S	-	nd	nd	+	-
	Mycobacterium senuense	28-35 days	Nonpathogenic	-	nd	nd	N	+	nd	nd	+	+
	Mycobacterium virginiense	> 7 days	Generally nonpathogenic	-	nd	nd	N	+	nd	nd	nd	+
	Mycobacterium triviale	> 7 days	Nonpathogenic	v	v	d	N	+	nd	nd	+	+
	Mycobacterium koreense	14 days	Nonpathogenic	-	nd	nd	N	-	nd	nd	+	+
	Mycobacterium parakoreense	≥ 28 days	Generally nonpathogenic	-	nd	nd	S	+	nd	nd	+	+

+, 90% or more of strains are positive; −, 90% or more of strains are negative; d, 11–89% of strains are positive; v, variability of reaction of strains within a given species; w, weak reaction; N, non-chromogenic; N*, pigmentation develop after 10-14d; S, scotochromogenic; P, photochromogenic. nd, No data available. Adapted table from (Magee and Ward, 2012) with additional information from selected papers.
