# Supplementary material for: Phylogenomics and Comparative Genomic Studies Robustly Support Division of the Genus Mycobacterium into an Emended Genus Mycobacterium and Four Novel Genera
Source: Front Microbiol. 2018 Feb 13;9:67. doi: 10.3389/fmicb.2018.00067 (PMC5819568; doi:10.3389/fmicb.2018.00067)
Supplement: Supplementary file 4 [file DataSheet1.PDF]

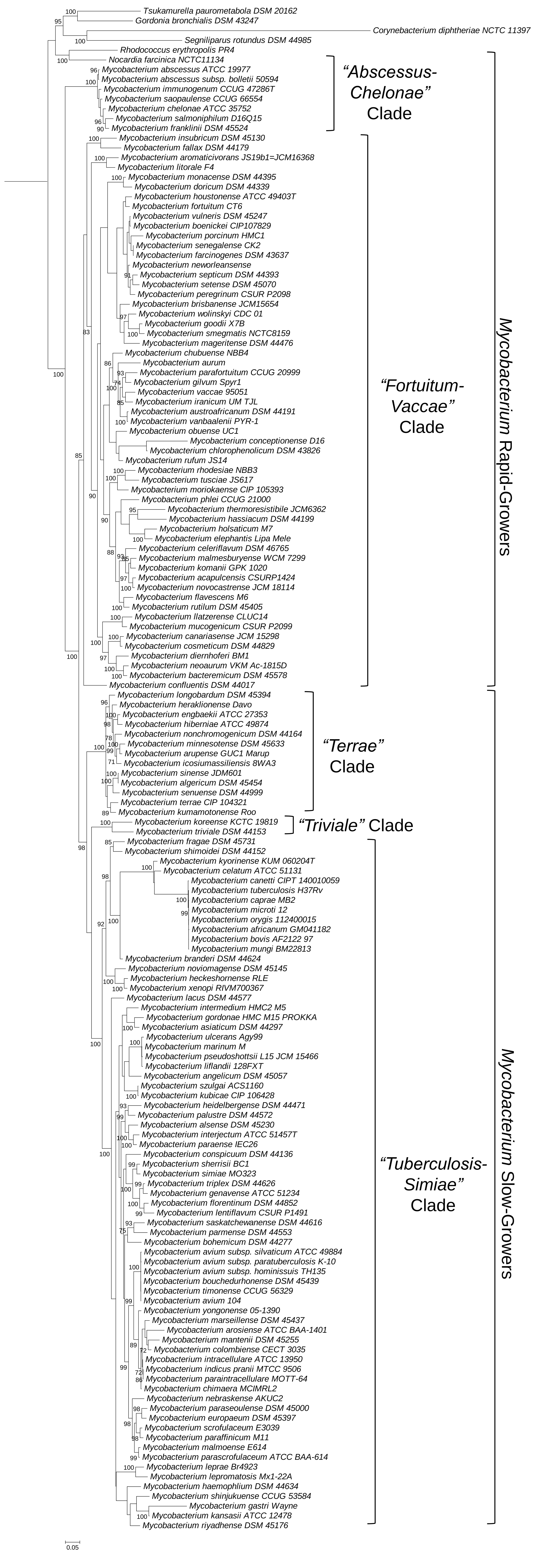

Supplementary Figure 1

A maximum-likelihood tree based on the concatenated amino acid sequences of eight housekeeping genes from the genus *Mycobacterium*. The major clades and subclades are labelled on the diagram. Additional information on the housekeeping genes are present in Supplementary Table 2.

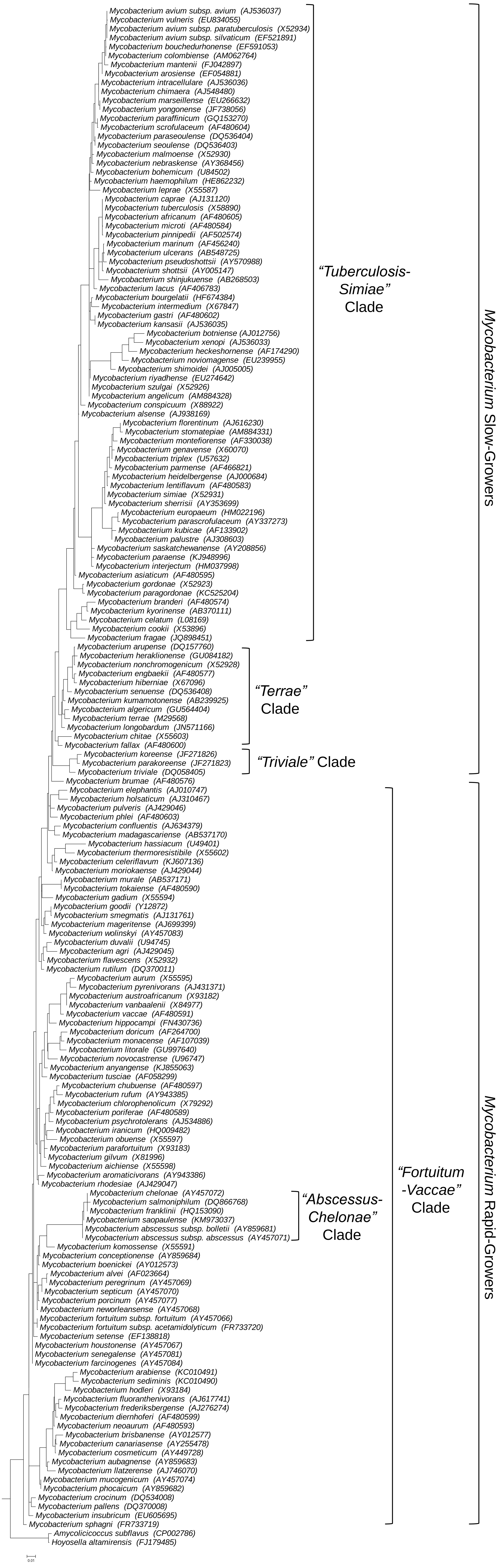

Supplementary Figure 2

A 16s rRNA tree extracted from the SILVA Tree of Life Project 128. Clades identified in the core protein tree are analogously labelled in this tree. The 16s rRNA reference ID are in the brackets.

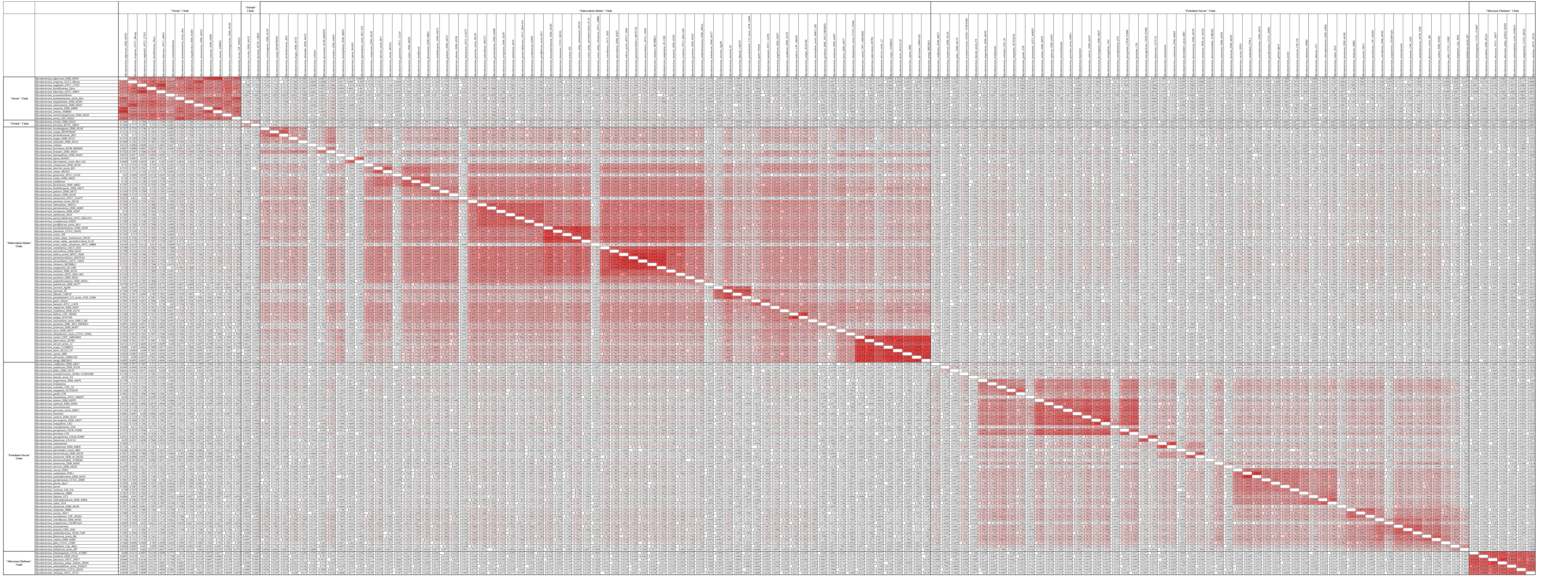

Supplementary Figure 3

A detailed matrix of the average amino acid identity of the 150 Mycobacterium genomes analyzed in this study.

Genus *Mycobacterium* (>100/>100)

Species	Accession	167	(boxed)	220
Mycobacterium abscessus	WP_062878530	VLVPEGPFVLGV	DA	VDEPFSLDNERPAHVVHLKGFRIGTVPVTN
Mycobacterium abscessus subsp. bolletii	EUA73945	------------	--	------------------------------
Mycobacterium africanum	AMC66272	---AG-------	--	A---C-----------DVPA----R-----
Mycobacterium algericum	WP_083038826	----A-E-----	--	TA---A--------R-EVAA----R---S-
Mycobacterium alsense	WP_083141053	--DG-------	--	AS--Y-----------DVPA----R-----
Mycobacterium angelicum	WP_083115545	----G-------	--	AT--Y-----------DVPA----R-----
Mycobacterium aromaticivorans	WP_036341879	----G-------	--	-T--L-----------DVPA----R-----
Mycobacterium arosiense	WP_083063923	----A-E-----	--	AS--Y-----------D-PA----R-----
Mycobacterium arupense	WP_046190308	---AG-E-----	--	A----A--------R-DVAD----R-----
Mycobacterium asiaticum	OBI73671	----G-------	--	AT--Y-----------EVTS----R-----
Mycobacterium aurum	WP_087027967	--I-G---T---	N-	SE--Y---------T--VEA----R-----
Mycobacterium austroafricanum	WP_036375120	-R--A-E-----	--	-T--Y-----------DVPA----R-----
Mycobacterium avium	WP_062886650	----G-E-----	--	A---E----------LD-PA----------
Mycobacterium avium subsp. avium	EUA24987	----G-E-----	-P	AA----------G---D-PA----R-----
Mycobacterium avium subsp. paratuberculosis	ETB47656	----G-E-----	--	A---E------R---LD-PA----------
Mycobacterium bacteremicum	WP_083061501	----A---T---	--	S----A--------T-DVDA----R-----
Mycobacterium boenickei	WP_077738598	--I-G-S-----	-E	LT--H---------S-EVAS-Y--R-----
Mycobacterium bohemicum	WP_085180022	----A-------	--	AT--H-----------D-PA----R-----
Mycobacterium branderi	WP_083134489	----G-------	--	AN--Y---------I-DVPA----R-----
Mycobacterium brisbanense	WP_062831310	--I-G---T---	-E	LN--H-----------DVPS----R-----
Mycobacterium canariasense	WP_062660193	-P--A-A-T---	N-	S---HA--------T-DVEA----R-----
Mycobacterium canettii	WP_015288612	---AG-------	--	A---C-----------DVPA----R-----
Mycobacterium celatum	WP_062541899	-V--G-------	--	AT--Y-----------DVPA----R-----
Mycobacterium celeriflavum	WP_083000355	----A-E-----	--	-T--H-----------DVPA----R-----
Mycobacterium chelonae	WP_070916850	----A-------	--	I----A---------LD-PS----------
Mycobacterium chimaera	WP_089151292	----A-E-----	-P	AA----------G---D-PA----R-----
Mycobacterium chlorophenolicum	WP_048472092	-E--A-T-----	-P	-A-------------ADVPA----R-----
Mycobacterium chubuense	WP_014817991	----G-E-----	--	LT--------------DIPA----R---S-
Mycobacterium colombiense	WP_064880000	----G-E-----	--	AG--Y-----------D-PA----R-----
Mycobacterium conceptionense	WP_064898411	--I-G-S-----	-E	LT--H---------S-DVAP----R-----
Mycobacterium confluentis	WP_085154446	-V--G-------	--	KG--Y-----------DVPA----R-----
Mycobacterium conspicuum	WP_085232661	----G-------	--	GS--Y-----------D-PA----R-----
Mycobacterium cosmeticum	WP_036397745	-P--A---T---	N-	S---YA--------T-EVAA----R-----
Mycobacterium diernhoferi	WP_073859276	--I-A---T---	N-	SE-HY---------T--VE-----R-----
Mycobacterium doricum	WP_085191888	-S--G-R-----	--	-T--H-----------D-P-----R-----
Mycobacterium elephantis	WP_083043802	----G-E-----	--	AT--H-----------DVPS----R-----
Mycobacterium engbaekii	WP_085128689	----G-E-----	--	EH--S---------R-EVAA----R-----
Mycobacterium europaeum	WP_085241311	----A-------	--	IS--Y-----------E-PA----R-----
Mycobacterium fallax	WP_085098299	-T--A-------	--	A---HA----------EVP-----R-----
Mycobacterium farcinogenes	WP_036391218	--I-G-S-----	-E	LT--H---------S-DVAP----R-----
Mycobacterium flavescens	WP_069415361	----G-E-----	--	-T--H-----------DVPA----R-----
Mycobacterium florentinum	WP_085225031	----G-------	--	TG--------------D-PP----R-----
Mycobacterium fortuitum	WP_061265067	--I-G-E-----	-E	LT--H---------T-D-PP-Y--R-----
Mycobacterium fragae	WP_085194088	---AG-------	--	AS--------------DVPA----R-----
Mycobacterium franklinii	WP_070937395	---------M--	--	I---Y---------S---------------
Mycobacterium gastri	WP_036416516	----G-------	--	ET--Y-----------DVPA----R-----
Mycobacterium genavense	WP_084298686	----G-------	--	TS------------I-DVPA----R-----
Mycobacterium gilvum	WP_011892214	---TG-E-----	--	-T------------T-DVPD----R-----
Mycobacterium goodii	WP_049743498	----G-S-----	--	LT--H-----------DVPS----R-----
Mycobacterium gordonae	OBJ80743	----G-------	--	AT--Y---------E-EVPS----R-----
Mycobacterium haemophilum	WP_054880823	-P--A-------	--	AS--Y-----------DVPA----RF--S-
Mycobacterium hassiacum	WP_005630880	--I-G-E-----	--	A---Y-----------DVPA----R-----
Mycobacterium heckeshornense	WP_048891452	----G-------	--	A---------------DVPS----R-----
Mycobacterium heidelbergense	ORA72063	-P--G-------	--	AS--Y-----------DVPT----R-----
Mycobacterium heraklionense	WP_064891129	----G-E-----	--	G-------------YIDVAA----R-----
Mycobacterium hiberniae	WP_085134111	----G-E-----	--	A---S---------R-DVAA----R-----
Mycobacterium holsaticum	WP_069407410	----G-E-----	--	AT--H-----------DIPS----R-----
Mycobacterium houstonense	WP_084459410	I-G---L---	-E	LA--H---------Q-DVAP-Y--R-----
Mycobacterium icosiumassiliensis	WP_067976611	----G-E-M---	--	T-------------R-DVAA----R-----
Mycobacterium immunogenum	WP_064631749	---------Q--	--	-------------------S----R-----
Mycobacterium insubricum	WP_083030804	-V--A---T---	-Q	----Y---------T-DAPA---AV-----
Mycobacterium interjectum	WP_085202297	--I-G-------	--	AS--Y-----------DVPA----R-----
Mycobacterium intermedium	WP_069417956	----G-------	--	AT--Y---------L-DVPA----R-----
Mycobacterium intracellulare	WP_064935034	----G-E-----	-P	AT---A------G-L-D-PA----R-----

Genus *Mycobacterium* (>100/>100)

Mycobacterium iranicum	WP_024447166	----A-E-T---	--	AT------------T-DVPA----R-----
Mycobacterium kansasii	WP_063468648	----G-------	--	EA--Y-----------DVPA----R-----
Mycobacterium komanii	CRL73949	----G-E-----	--	AT------------T-DVPS----R-----
Mycobacterium kubicae	WP_085074709	----G-------	--	AS--Y-----------DVPS----R-----
Mycobacterium kumamotonense	WP_065289552	----A-E-----	--	S----A--------R-EVAA----R-----
Mycobacterium kyorinense	WP_065012857	----A-------	-S	A---------------DVPA----R-----
Mycobacterium lacus	WP_085159009	----A-------	--	AS--Y---------I-DVPAY---R-----
Mycobacterium lentiflavum	CQD22090	----G-------	--	TS--------------D-PA----R-----
Mycobacterium litorale	WP_078021271	----G-------	--	Q---L---------T-DVAS----R-----
Mycobacterium longobardum	WP_085265501	----G-E-----	--	T----A--------R-DVAT----R-----
Mycobacterium mageritense	WP_036431691	----G---T---	--	LA--H---------T-DVPS----R-----
Mycobacterium malmesburyense	CRL72666	----G-E-----	--	AT--Y--------YI-DVPA----R-----
Mycobacterium malmoense	WP_065446360	-P--A-------	--	AS--Y-----------E-PA----R-----
Mycobacterium mantenii	WP_083095551	----A-E-----	--	AS--Y-----------D-PA----R-----
Mycobacterium marinum	WP_020730941	-E--A-------	-G	SA--Y---------P-DVAA----R-----
Mycobacterium marseillense	WP_083016561	----G-E-----	-P	AR----------G---D-PA----R-----
Mycobacterium minnesotense	WP_083025537	----G-E-----	N-	A----A--------C-DVAT----R-----
Mycobacterium moriokaense	WP_083154783	----A-E-I---	--	-T--H------GP---D-PA----------
Mycobacterium mucogenicum	WP_064857720	----G-------	--	A----A----G-----DVPS----R-----
Mycobacterium nebraskense	WP_046184329	----A-------	--	-S--Y-----------E-PA----R-----
Mycobacterium neoaurum	WP_030132771	--I-G---T---	--	SA--Y---------T-EVDA----R-----
Mycobacterium neworleansense	CRZ17720	--I-G-------	-E	LT--H---------E-DVAS----R-----
Mycobacterium nonchromogenicum	WP_085138290	----G-A-----	--	T----A--------T-EVAT----R-----
Mycobacterium noviomagense	WP_083089099	----G-------	E-	AS--------------DIPS----R-----
Mycobacterium novocastrense	WP_084377314	----G-E-----	--	AT--Y-----------DVPA----R-----
Mycobacterium obuense	WP_046364035	-E--A-E-----	-P	AA------------T-DVPA----R-----
Mycobacterium palustre	ORW29770	----G-------	--	AS--H-----------DVPP----R-----
Mycobacterium paraense	WP_085244773	--I-G-------	--	AG--Y-----------DVPA----R-----
Mycobacterium paraffinicum	WP_073871784	----A-------	--	-S--Y-----------DVPA----R-----
Mycobacterium parafortuitum	WP_083142934	----G-E-----	--	--------------R-DVPA----R-----
Mycobacterium paraintracellulare	AFC51868	----G-E-----	-P	AA----------G---D-PA----R-----
Mycobacterium paraseoulense	WP_083173508	-P--A-------	--	AT--Y-----------E-PA----R-----
Mycobacterium parmense	WP_085267120	----A-------	--	AS--S-----------D-PA----R-----
Mycobacterium peregrinum	WP_064878751	--I-G-------	-E	LT----------P-S-EVAS----R-----
Mycobacterium phlei	WP_003888646	----G-E-----	--	-T--H-----------DVPA----R-----
Mycobacterium porcinum	WP_069425639	--I-G-S-----	-E	LT--H---------I-EVAP----R-----
Mycobacterium rhodesiae	WP_083120875	----G-------	--	-S--L-----------DVPA----R-----
Mycobacterium riyadhense	WP_085251751	----G-------	--	AS--Y-----------DVPA----R-----
Mycobacterium rufum	KGI70142	-E--A-------	--	-T----------S-T-DVPA----R-----
Mycobacterium rutilum	WP_083406888	-P--G-E-----	--	-T--H---------I-DVPA----R-----
Mycobacterium salmoniphilum	WP_078324371	------------	--	S-------------T-D--S----R-----
Mycobacterium saopaulense	WP_070912294	------------	-P	--------------T----S----------
Mycobacterium saskatchewanense	WP_085258257	----A-------	--	AT--Y-----------D-PA----R-----
Mycobacterium scrofulaceum	WP_067270900	----A-------	--	AT--Y-----------EVPA----R-----
Mycobacterium senuense	WP_085085561	----A-E-----	--	TA---A--------R-QVAA----R-----
Mycobacterium septicum	WP_044521239	--I-G-------	-E	FT--H-------P-T-E-PA-S--R-----
Mycobacterium setense	WP_064875156	--I-G-------	-E	LT--H---------E-DVAS----R-----
Mycobacterium sherrisii	WP_085167088	----G-------	--	GS--------------DVPS----R-----
Mycobacterium shigaense	BAX94819	----A-------	--	AE--Y-----------DVPA----R-----
Mycobacterium shimoidei	WP_069397216	------------	--	AT---A----------DVPA----R-----
Mycobacterium shinjukuense	WP_083052084	----G-------	--	AS--Y-----------DVPA----R-----
Mycobacterium simiae	WP_061557478	----G-------	N-	SS--------------DVPS----R-----
Mycobacterium sinense	WP_064855727	----A-E-L---	-L	T----A--------R-EVAA----R-----
Mycobacterium smegmatis	WP_003897671	----G-------	--	LT--H-----------DIPS----R-----
Mycobacterium szulgai	WP_085670728	---AG-------	--	AT--Y-----------DVPA----R-----
Mycobacterium terrae	WP_085259933	----A-Q-----	--	A----A--------R-EVGA----R-----
Mycobacterium thermoresistibile	WP_050811957	----G-------	-L	A---YA----------DVPA----R-----
Mycobacterium triplex	WP_084163242	----G-------	--	TS--------------D-PA----R-----
Mycobacterium triviale	WP_085109296	----G-------	-E	GT---A-----S--E-EVPA----R-----
Mycobacterium tuberculosis	WP_070892200	---AG-------	--	A---C-----------DVPA----R-----
Mycobacterium tusciae	WP_083126298	----A-E-----	--	-T--H------C--M-D-PA----------
Mycobacterium ulcerans	WP_011741908	-E--A-------	-G	SA--Y---------P-DVAA----R-----
Mycobacterium vaccae	WP_003931849	----G-E-T---	--	AA--Y---------T-DVPS----R-----
Mycobacterium vanbaalenii	WP_011782609	-R--A-E-----	--	-T--Y-----------DVPA----R-----
Mycobacterium vulneris	WP_065462877	--I-G-------	-E	LT--H---------I-EVAS----R-----

**Genus *Mycobacterium* (>100/>100)**	*Mycobacterium wolinskyi*	WP_067851775	--I-G-------	-E	LT--H-------P---DVAS----R-----
	Mycobacterium xenopi	WP_085196016	----G-------	--	A----A--------I-DVPS----R-----
	Mycobacterium yongonense	WP_065507949	----A-K-----	-P	AA----------G---D-PA----R-----
**Other *Corynebacteriales***	*Hoyosella altamirensis*	WP_064438961	AV--A---EM-T		SSD-WA--------N--VDA-A-ARA----
	Hoyosella subflava	WP_013806907	AV--A---EM-T		SSD-WA--------R--VDA-A-ARA----
	Nocardia acidivorans	WP_067570027	-VI-A-E--M-T		DSD-WA--------P--VP--A-DIA-I--
	Nocardia africana	WP_062964754	-VI-A-E-TM-T		SSD-WA------E-R---PA-A-DAA----
	Nocardia alba	WP_067451616	IVL-A-E-LM-T		SAD-WA--------P--VD--A-DVA----
	Nocardia beijingensis	WP_067795302	-VI-A-E-TM-T		STD-WA--------P--VA--A-DAA----
	Nocardia brasiliensis	WP_014988196	-V--A-E-TM-T		DTD-WA--------RIR-P--A-DAA----
	Nocardia brevicatena	WP_040838137	-I-AG-E--M-S		STD-WA--------P--VP--A-DAA----
	Nocardia caishijiensis	WP_067980174	-I--A-E-TM-T		SAD-WA--------P-YVE--A-DVA----
	Nocardia cerradoensis	WP_039780690	-VI-A-E-TM-T		SSD-WA------E-R--VP--A-DAA----
	Nocardia concava	WP_051179399	-VI-A-E-MM-T		DTD-WA--------P--VG--A-DAA-I--
	Nocardia coubleae	WP_067637928	-V----E-MM-T		SAD-WA--------P-YVE--A-DVA----
	Nocardia crassostreae	WP_067533732	-VI-A-E--M-T		DTD-WA--------P--VP--A-DAA----
	Nocardia cummidelens	WP_063005595	IVI-A-E-LM-T		SAD-WA--------P-QVD--A-DVA----
	Nocardia cyriacigeorgica	WP_014352878	-II-A-Q-TM-T		STD-WA--------LA-VP--A-DAA----
	Nocardia elegans	WP_063023545	-VI-A-E-TM-T		SSD-WA------E-R-Q-P--A-DAA----
	Nocardia farcinica	WP_011211421	-I--G-E-TM-T		SAD-WA--------P--VPA-A-DAA----
	Nocardia fusca	WP_063128264	-VI-A-E--M-T		STD-WA--------P--VPA-A-DAA-IS-
	Nocardia harenae	WP_067653123	-I--A-E--M-T		STD-WA--------P--VP--A-DAA----
	Nocardia higoensis	WP_040798307	-V--G-E-LM-T		SAD-WA--------P--VPA-A-DAA----
	Nocardia ignorata	WP_067485523	-V--A-E-MM-T		SAD-WA--------P-YVE--A-DVA----
	Nocardia inohanensis	WP_067816692	-VI-A-E--M-T		DTD-WA--------P--VP--A-DAA-I--
	Nocardia jejuensis	WP_067697884	-VI-A-E-TM-T		DTD-WA--------P--VP--A-DAA----
	Nocardia jinanensis	WP_058853158	-MI-A-E--M-T		STD-WA--------P--VPA-A-DAA--S-
	Nocardia seriolae	WP_033088773	-VI-A-E-IM-T		DAD-WA--------P--VDA-A-DAA-I--
	Nocardia shimofusensis	WP_067853513	-I--G-E-LM-T		SAD-WA--------P--VPA-A-DAA----
	Nocardia sienata	WP_063061717	-VI-A-E--M-T		STD-WA--------P--VPA-A-DAA--G-
	Nocardia soli	WP_063054395	IVI-A-E-LM-T		SAD-WA--------P-QVD--A-DVA----
	Nocardia takedensis	WP_051029793	-VI-A-E-TM-T		STD-WA--------P--VPE-A-DAA----
	Nocardia tenerifensis	WP_051187223	-V--A-E-TM-T		ST--WA--------R-R-P--A-DAA----
	Nocardia testacea	WP_051165029	-VI-A-E--M-T		STD-WA--------P--VPA-A-DAA--G-
	Nocardia thailandica	WP_043657455	-R--A-E--M-T		SAD-WA--------P--VPA-A-DAA----
	Nocardia yamanashiensis	WP_067710574	-II-A-E--M-T		DTD-WA--------P--VPA-A-DAA-I--
	Rhodococcus corynebacterioides	WP_068147532	-I--G---EM-T		DTD-WA--------T-QVET-A-DA-----
	Rhodococcus equi	WP_064058930	TVI-A-E-TM-T		S-D-WA--------R--VPE-A-D----S-
	Rhodococcus erythropolis	WP_069146383	-TI-A----M-T		D---WA-----T--Q-YVTD-A-DRF----
	Rhodococcus jostii	SEE36435	-II-A-E-TM-T		S-D-WA-----S--S---PA-V-DS-----
	Rhodococcus kroppenstedtii	WP_068365082	-I--G---EM-T		DTD-WA--------T--VET-A-DA-----
	Rhodococcus kunmingensis	WP_068273637	-I--G-E--M-T		ST--WA--------R-SVHA-A-DAA-I--
	Rhodococcus phenolicus	WP_068159940	-TI-G-EVEI-T		TTD-WA--------R--VRA---D------
	Rhodococcus qingshengii	WP_007728907	-TI-A----M-T		D---WA-----T--Q-Y-TD-A-DRF----
	Rhodococcus rhodochrous	WP_033234289	-V--A----M-T		D---WA-----T--Q-Y-GPYA-DRF----
	Rhodococcus triatomae	SDH14337	-V--G-E--M-T		SV--WA--------A-DVPS-G-D-F----
	Rhodococcus tukisamuensis	SDE34070	----A-E-TM-T		STD-WA--------R-V-PA-A-D-A----
	Tsukamurella pulmonis	WP_068537494	IVI-A---TM-T		SS--WA--------R-D-PAYA-DA-----

Supplementary Figure 4

Detailed sequence information for the two amino acid insertion found in ergothioneine biosynthesis protein EgtB, which is shown in Figure 3. This insertion is specific for members of the genus *Mycobacterium* and absent in other *Corynebacteriales* including the two *Hoyosella* species.

Genus *Mycobacterium* (>100/>100)

		159		206
Mycobacterium abscessus	WP_078061976	TLALHLAAAQIDTVAADLV	A	GGYPSDTPVAVVAYASWPSETILRGTL
Mycobacterium abscessus subsp. bolletii	SHY10218	-------------------	-	----------A----------------
Mycobacterium africanum	WP_031667640	--V---------AIVPR-L	D	---RPE-------F----QQRT-----
Mycobacterium alsense	WP_083137874	--V----------IVPQ-L	E	---RPE--T----F----R--V-----
Mycobacterium angelicum	WP_083114891	--V---------AIVPQ-L	-	---RAE--A----F----QQ-V---C-
Mycobacterium aromaticivorans	WP_036337566	--V-----HR--AIVPE-L	D	---RP--------F----K--V-----
Mycobacterium arosiense	WP_083064272	--V---------AIVPQ--	E	---RAE--A----F----Q-AV-----
Mycobacterium asiaticum	WP_065034187	--V---------AIVPQ-L	-	---RPE-------F----QQ-V-----
Mycobacterium aurum	WP_087023314	--V-------AE-IV-E-T	S	---TRE--C---------T-QVV-CA-
Mycobacterium avium	WP_003872141	--V---------SIVGQ-L	D	N-RQ-Q--C----F----QQ-V-----
Mycobacterium boenickei	WP_077741038	--V---------NIVPQ-L	D	---TPE--C----F----Q-IV-----
Mycobacterium bohemicum	WP_085181134	--V----------IVEQ--	N	---RRE--A-A--F----QQAV-----
Mycobacterium branderi	WP_083132010	--V---------AIVPQ-L	-	---RAE--T----F----QQ-V-----
Mycobacterium brisbanense	WP_062830464	--V---------HIVPQ-L	D	---KPE--C----F----Q--V-----
Mycobacterium canariasense	WP_062657249	--V--------E-IV---T	-	---TT---C----F----TQQV--S--
Mycobacterium canettii	WP_015290385	--V---------AIVPR-L	D	---RPE-------F----QQRT-----
Mycobacterium celatum	WP_062539094	--V---------AIVPQ-L	-	---LAE--T----F----QQ-V-----
Mycobacterium chelonae	WP_070916146	---N-V----	-	----PG---------------------
Mycobacterium chimaera	WP_054585327	--V---------AIVPQ-L	D	---RRE-------F----Q-MV-----
Mycobacterium colombiense	WP_064881472	--V---------SIVPQ-L	E	---QPE--A----F----Q-AV-----
Mycobacterium confluentis	WP_085151125	--V-----------V-E-T	-	--VDAA--T----F----EQIV-----
Mycobacterium conspicuum	WP_085232912	--V---------AIVPQ-L	-	---QPE--A----F----EQ-V-----
Mycobacterium cosmeticum	WP_036399906	--V--------E-IV---T	-	---TA---C---------TQQV--S--
Mycobacterium diernhoferi	WP_073855056	--V-------AE-IV-E-T	-	---TRE--C---------T-KVV-CA-
Mycobacterium doricum	WP_085191582	--V-------V-NIVPE-L	-	---RPE--C----F----GQIV-----
Mycobacterium elephantis	WP_046754054	--V-------V--IVPE-L	-	---RPE--C----F----QQQVV-C--
Mycobacterium europaeum	WP_085239843	--V---------AIVPQ-L	E	---RPE--A----F----QQVV-----
Mycobacterium fallax	WP_085099537	--------------V-E-R	-	--L-G-------------T--VV----
Mycobacterium flavescens	WP_069413618	--V----------I--E-A	D	---RPE--C----F----QQQVV-C--
Mycobacterium florentinum	WP_085220588	--V--------V-IVEQ-R	T	N--KPE--T----F----Q-IT-----
Mycobacterium fortuitum	WP_061264656	--V---------NIVPQ-L	D	---QPE--C----F----Q-IV-----
Mycobacterium fragae	WP_085199890	--V---------AIVPQ-L	G	-S-RTE--T----F----QQVV-----
Mycobacterium franklinii	WP_070937106	-----V----	-	----P---------------I------
Mycobacterium gastri	WP_036409318	--V---------AIVPE-L	S	---RAE--T----F----QQAV-----
Mycobacterium genavense	WP_025736158	--V---------AIVPQ-L	R	S--SPE--S----F----E--V-----
Mycobacterium goodii	WP_049746351	--V---------N-VPQ-L	D	---RPE--C----F----Q-VV-----
Mycobacterium gordonae	WP_065049226	--V---------HIVPQ-L	-	---AAQ--T----F----QQIV-----
Mycobacterium haemophilum	WP_047316387	--V---------AIIPQ-L	-	---RPE--A----F----QQ-V-----
Mycobacterium hassiacum	WP_005627975	--V---------NIVPE-L	-	---RP---C----F----QQQ-V-CP-
Mycobacterium heckeshornense	WP_048892134	--V---------AIIPE-L	S	---RPE--T----F----QQVM-----
Mycobacterium heidelbergense	WP_083072570	--V---------AIVPQ-I	-	---RAE--A----F----RQ-V---A-
Mycobacterium holsaticum	WP_069406187	--V-------V-SIVPE-L	-	A--RPE--C----F----Q-QVI-C--
Mycobacterium houstonense	WP_066897996	--V---------NIVPQ--	D	---RPE--C----F----Q-IV-----
Mycobacterium immunogenum	WP_064632612	-----VG---	-	---SP---A-----------IV-----
Mycobacterium indicus pranii MTCC 9506	AFS14298	--V---------AIVPQ-L	D	---RPE----A--F----Q--V-----
Mycobacterium insubricum	WP_083029941	--V-----------V---R	-	--LAG-------------T--VV----
Mycobacterium interjectum	WP_066913257	--V---------AIVPQ-L	E	---GPE--S----F--R-QQ-V-----
Mycobacterium intermedium	WP_069420331	--V---------VIVPQ-T	-	A--RPE--T----F----QQVV-----
Mycobacterium intracellulare	WP_064938959	--V---------AIVPQ-L	D	---RPE-------F----Q--V-----
Mycobacterium kansasii	WP_063466478	--V---------AIVPQ-L	S	---RPE--T----F---SQQCV-----
Mycobacterium komanii	CRL66867	--V---------AIVPE-L	V	---QPE--C----F----QQQVV-C--
Mycobacterium kubicae	WP_085072898	--V---S------IV-Q-Q	-	A--H-E--C----F----QQ-V---V-
Mycobacterium kyorinense	WP_065016487	--V---------AIVQK--	N	A--RPE--T----F----RQSV-----
Mycobacterium lacus	WP_085155774	--V---------HIVGQ--	V	-C-RPE--A----F----QQ-V-----
Mycobacterium lentiflavum	CQD15599	--V----------IVPQ-L	K	A--RPE--T----F----Q-VT-----
Mycobacterium litorale	WP_078019022	--VI----HR--AIVPQ-L	E	---RP---A----F-T--QQVV-----
Mycobacterium llatzerense	WP_071288865	--V---------NIVPQ-L	E	---RPE--C----FV---T-QVV-C--
Mycobacterium mageritense	WP_036439256	--V---------NIVPH-L	D	---RPE--C----F----D-VV-----
Mycobacterium malmesburyense	CRL68876	--V---------AIVPE-L	-	---RPE--C----F----Q-QVV-C--
Mycobacterium malmoense	WP_065443132	--V--------GAIVPQ-L	D	---RPE--A----F----Q-VV-----
Mycobacterium mantenii	WP_083092175	--V---------AIVPQ-L	E	---RPE--A----F----Q-AV-----
Mycobacterium marinum	WP_012394742	--V---------AIVPQ-L	-	---RPE--A----F----QQAV-----
Mycobacterium moriokaense	WP_083151520	--V----------IVPQ-L	-	--RD-E--C----F-T--QQQV--CR-
Mycobacterium mucogenicum	WP_064859094	--V---------NIVPQ-L	E	---RPE--C----FV---T-QVITC--
Mycobacterium nebraskense	WP_046184717	--V---------A-VPQ-L	E	---RPE--A----F----QQVV-----
Mycobacterium neworleansense	CRZ13909	--V---------NIVPQ-I	D	---RPE--C----F----Q-SV-----
Mycobacterium noviomagense	WP_083087417	--V-----T--AAIVPQ-L	-	---RPG--T----F----QQIV-----
Mycobacterium novocastrense	WP_067389528	--V---V------I--E-L	-	---RPE-AC----F----QQQVV-C--
Mycobacterium palustre	WP_085076101	--V---------AIVPR-L	E	---RPE--T----F----RQSV-----
Mycobacterium paraffinicum	WP_073873206	--V---------AIVPQ-L	D	---R-E--A----F----Q-VV-----
Mycobacterium parascrofulaceum	WP_007170351	--V-------V-AIVPQ-L	E	---RPE--A----F----QQVV-----
Mycobacterium paraseoulense	WP_083172687	--V---------AIVPQ-L	D	---RPE--A----F----QQVV-----
Mycobacterium parmense	WP_085268704	--V---------A-VPQ-T	-	---RPE--A----F----QQVV-----

Group	Organism	Accession	Alignment	Boxed position	Alignment (cont.)
Genus *Mycobacterium* (>100/>100)	*Mycobacterium peregrinum*	WP_064887194	--V---------NIVPQ-L	D	---RPE--C----F----Q-IV-----
	Mycobacterium persicum	WP_083156067	--V---------KIVPE-L	S	---RPE--T----F----QQCV-----
	Mycobacterium phlei	WP_061482930	--V---------AIVPQ-L	E	---TPE--C----F----QQQVV-CP-
	Mycobacterium porcinum	WP_075920018	--V---------NIVPQ-L	D	---RPE--C----F----Q-IV-----
	Mycobacterium rhodesiae	WP_083120479	--V-----HR--AIVPQ-L	E	---RA--------F----Q-VV-----
	Mycobacterium riyadhense	WP_085250558	--V----------IVPQ-L	-	A--KPE--T--A-F----Q--V---S-
	Mycobacterium rutilum	WP_083408478	--V----------I-GE-L	T	---RPE--C----F----QQQVV-C--
	Mycobacterium salmoniphilum	WP_078326323	-----V-E--	-	----PQ--A-----------V------
	Mycobacterium saopaulense	OHT86450	-----V----	-	--------------------I------
	Mycobacterium saskatchewanense	WP_085254099	--V---------AIVPQ-L	D	---RHE--A-A--F----QQAV-----
	Mycobacterium scrofulaceum	WP_067272784	--V-------V-AIVPQ-L	E	---RPE--A----F----Q-VA-----
	Mycobacterium septicum	WP_044523953	--V---------NIVPQ-L	D	---GPE--C----F----E-IV-----
	Mycobacterium setense	WP_039321391	--V---------NIVPQ-I	D	---RLE--C----F----Q-IV-----
	Mycobacterium sherrisii	WP_069403061	--V----------IVQQ-I	E	S-CRPE----T--F-T--Q--V-----
	Mycobacterium shigaense	BAX93094	--V---------AIVPQ-L	-	D--RPE--A-T--F----Q--V-----
	Mycobacterium simiae	WP_061559679	--V-------V---V-Q-L	E	N--RPE----T--F-T--Q--L-----
	Mycobacterium smegmatis	CKI05351	--V---------NIVPQ-I	E	---RPE--C----F----Q-VV-----
	Mycobacterium szulgai	WP_085671161	--V---------AIVPQ-L	D	---RAE--T----F----QQ-V---C-
	Mycobacterium thermoresistibile	WP_003923419	--V---------AIVPK-L	E	---RPE--C----F----EQ-V-----
	Mycobacterium triplex	WP_036472612	--V---------AIVPQ-L	-	S-HGPE--S----F----QQ-V-----
	Mycobacterium triviale	WP_085111326	--V---------AIVPQ-L	-	---RP--------F--R-EQ-V---E-
	Mycobacterium tuberculosis	WP_070898912	--V---------AIVPR-L	D	---RPE-------F----QQRT-----
	Mycobacterium tusciae	WP_083123709	--V---------AIVPQ-L	-	---RPE--C----F----QQQ-I-CA-
	Mycobacterium ulcerans str. Harvey	EUA89918	--V---------AIIPQ-L	-	---RPE--A----F----QQAV-----
	Mycobacterium vulneris	WP_065458557	--V---------NIVPQ-L	D	---RPE--C----F----Q-IV-----
	Mycobacterium wolinskyi	WP_085149689	--V---------AIVPQ-I	D	---RPE--C----F----E-IV-----
	Mycobacterium xenopi	WP_003918956	--V---------AIVPE-L	-	---RPE--T----F----QQVV-----
	Mycobacterium yongonense	WP_065503798	--V---------AIVPQ-L	D	---RRE-------F----Q--V-----
Other bacteria	*Hoyosella altamirensis*	WP_064438896	--V----VGH--R-VSE-L		PS-GP-C------F--R-D-K-V----
	Hoyosella subflava	WP_013806825	--V----VGH--R-VSE-L		PS-GP-C------F--R-D-K-V----
	Acetobacter cerevisiae	WP_062141496	---I---IHALGKIVQE-T		PF-GA-C---I--R-T--DQLV-----
	Acidocella facilis	WP_026439939	------SIHRLAEIV-E-T		PF-GA-C----IWR----DQRV-----
	Actibacterium mucosum	WP_035260435	---I--SIH-V---VGA-T		PA-GA-C-----YR----DQQ-V-A--
	Actinomadura chibensis	WP_067900227	-MV----VQR--E-T-E-L		PH-GA-C------R--R-D-L------
	Actinophytocola xanthii	WP_075123954	--V----ITRTRVL-KE-T		-F-GP-C--V---N--Q-A-VV-----
	Actinoplanes rectilineatus	WP_045743006	-MV----VQR--A-V----		-N-GT-C------R--R-D-L-V----
	Aeromicrobium choanae	SKB10401	-M----GITRTREL--E-A		EH-GQ-C--V--SK-TQ-DQLV-----
	Aestuariimicrobium kwangyangen	WP_040437348	S-V----ITRTR-LM-E-E		PH-GA-C--V--YR--Q-G-LVV---V
	Agrobacterium tumefaciens	WP_065659990	---I---IHA-GQ-VEE-T		PL-G--C---I-VR----D-RVI----
	Aidingimonas halophila	SDW91658	---I--SVHKL-Q-VEE--		PF-GG-C---I-WR----D-RVV--Q-
	Albidovulum xiamenense	SDJ92753	---I--GIRALREIVRV-T		PH-GE-C-----YR----DQQ-I----
	Aminobacter aminovorans	WP_067960670	---V--SIHA--R-V-E-T		PH-GP-C-----FR----D-R-V-A--
	Amycolatopsis nigrescens	WP_020666836	-------VNR-EQ--EE-L		PH-GP-C------R--QDA-QV---R-
	Austwickia chelonae	WP_006502647	--V---SIRHVRRL-GE--		RP-GA-C-----SR-EQ-D-LV-----
	Blastococcus endophyticus	SEP27627	--V----VQRLGEL-PE-A		-H-GA-C-A----F--RDD-VV-----
	Bradyrhizobium pachyrhizi	WP_057015899	V--I--SIHLL-K-I-E-T		PH-GA-C---I-WR----DQR-V----
	Citreicella thiooxidans	SDE48302	---I--SIGNLAH-T-E-A		PH-GA-C-----FR----D-R-I----
	Dietzia alimentaria	WP_069390255	--V----ITR-REL-ER--		PTHGA-C------N--R-HRVE-----
	Geodermatophilus siccatus	SDM50945	--V----VQR-AEL-PE-A		EH-GD-G------R--RDD-LV-----
	Gordonia paraffinivorans	WP_006899423	--------HRAEQIVE--T		PY-GA-C-T-T--F--R-NQQVV-SP-
	Haematobacter massiliensis	WP_035710198	------SIHRLAEIS-E-E		SH-GPEC----IWR----E-RVV----
	Halomonas smyrnensis	WP_016854517	---I--SIHNLKQ-V-E-A		PR-GD-C---I-WR----DQKVI--R-
	Janibacter terrae	WP_072624188	--V----IRRAREL-GR-A		EH-GA-C----AYRVEQ-E-IV-----
	Kribbia dieselivorans	WP_068397782	--V----IRRTRELMSEIE		PE-GP-C--V--YR--Q-EQQ-----V
	Loktanella atrilutea	WP_072856304	---I--GIRALRDIVRR--		PF-GA-C-----YR----DQV------
	Luteipulveratus halotolerans	WP_050671693	--V----IRHTRRLTGE--		EH-G--C-----YR--Q-EQIV-A---
	Luteococcus japonicus LSP_Lj1	SJN44469	--V----ITRTRELM-E-E		PTHGP-C--V--SR--Q-E-QV----V
	Magnetovibrio blakemorei	WP_069957557	---V--SVNNLGP-IKQ-S		PY-GA-C-----YR----D-AVVT---
	Mameliella alba	WP_074623222	------SVG-LEK-Q---L		P--GA-C-----YR----DQRVI-C--
	Marinobacter nanhaiticus	WP_004582371	---I--SIHNLAQ-V---T		PH-GA-C-----WR----D-RVI--R-
	Marinovum algicola	WP_074835195	------SIQK-AR-V---T		PA-GA-C-----YR----DQK------
	Marmoricola aequoreus	WP_030484160	-IV----T-RVGEAMRE-A		PHQGE-C--V--HR--Q-G-LV----V
	Meganema perideroedes	WP_018633605	------SIHRLKEI-EE-A		AH-GP-C-S---WR----D-RVV-AP-
	Mesorhizobium erdmanii	WP_027051234	---I---IHA--R-V-E-T		PH-GG-C---I-FR----D-RV-----
	Millisia brevis	WP_066906379	L--V--G-HR-TE-V-EVI		-VLGG-C-A----F--Q-G-RV--AP-
	Modestobacter caceresii	WP_036332873	--V----VQRM-AL-PE-A		AH-GA-C------K--RDD-LV-----
	Monashia flava	WP_076261220	--V----IRHVRRL--E--		DD-G--C--V---N-EQ-DQLV-----
	Natronohydrobacter thiooxidans	WP_071796619	------SIQNLGQIV-E-T		PY-GP-C---I-WR----DQR-I-A--

**Other bacteria**	*Nautella italica*	WP_050672509	---I--SIGNL-H-VES-T	PH-GA-C-----YR----DQQ-H-A--
	Neorhizobium galegae	WP_046668177	---I---IHAL-RIV-E-T	PL-GA-C---I-VR----E-R-I----
	Nereida ignava	WP_048598379	---I---IRNLREIERQ-I	PY-GE-C--V-AYR----DQLFI----
	Nocardia arthritidis	WP_063052481	-------ITRVRAL--E-S	AD-GP-C-----YR--Q-EQL------
	Oceaniovalibus guishaninsula	WP_007425625	---I--SIQNLA--V---T	PH-GG-C---I-WR----D-R-V-A--
	Pacificibacter marinus	SEK90300	-M-I--GVRNLREIERQ--	PY-GA-C-----YRVG--DQMLI----
	Planktomarina temperata	WP_044050703	---I---IRNMREIERV-I	PH-GA-C--V-AYR----DQI-I----
	Rhizobium marinum	WP_029621063	---I---IHA--R-V-E-L	PF-GT-C---I-VR----E-R-I----
	Rhodococcus equi	WP_013416035	-MVV--G-HR-EQIVEE-S	EN-G--C-A----F--R-D-VV-----
	Tetrasphaera japonica	WP_048554257	--V----ITRTREL-VE-A	-H-GPAC-----SRV-Q---LV-----
	Thermobifida fusca	WP_011290762	--V----IRH-RVL-EQ--	PE-GA-C------H-TR-N-LV-----
	Tsukamurella paurometabola	WP_013126811	--------HRGPELT-E--	PH-GA-C------F--R-EQQ-V-CR-
	Williamsia herbipolensis	WP_045822799	--------HR-EQ-Q----	EH-GA-C-C----F--RDD-Q-V-C--
	Yangia pacifica	SDI12102	---I--SIQN-AR-V---S	PA-GP-C-----YR----D-V------
	Yuhushiella deserti	SFO98244	-------VNR-EH-V-E--	PH-GG-C-A----H--Q-GQRV---P-

Supplementary Figure 5

A partial sequence alignment of a conserved region of precorrin-4 C(11)-methyltransferase showing a one amino acid insertion that is specific for members of the genus *Mycobacterium* and absent in most other bacteria including the two *Hoyosella* species.

Genus *Mycobacterium* (93/97)

		9		56
Mycobacterium abscessus	WP_062879231	GGGPAGYEAALVAAAHERS	TTE	VTVIDSDGIGGACVLFDCVPSKTFIA
Mycobacterium acapulcensis	WP_066810436	---------------RGPE	VA-	---------------W----------
Mycobacterium africanum	WP_049958603	--------------TSHPE	--Q	-----C------A--D----------
Mycobacterium aromaticivorans	WP_036340424	--------------GRG-E	VA-	---V----------------------
Mycobacterium arupense	WP_046190694	---------------KGPE	AVS	---V-A---------W----------
Mycobacterium asiaticum	WP_065159567	---------------RGPE	IAQ	---V-A------A--C----------
Mycobacterium aurum	WP_048630233	---------------RG-D	AVQ	--IV----L------Y-------L--
Mycobacterium austroafricanum	WP_036369760	--------------GLG-D	L-Q	--------L------Y----------
Mycobacterium avium subsp. avium	EUA38817	--------------SSHPD	S-H	------E-----A--D----------
Mycobacterium avium subsp. hominissuis	KDP04680	--------------SSHPD	S-H	------E-----A--D----------
Mycobacterium avium subsp. paratuberculosis	AAS05974	--------------SSHPD	S-H	------E-----A--D----------
Mycobacterium avium subsp. silvaticum	ETB15343	--------------SSHPD	--H	------E-----A--D----------
Mycobacterium boenickei	WP_077743424	---------------RGPE	VAH	--IV-C---------W----------
Mycobacterium bohemicum	CPR13461	--------------TSHPD	SAR	------------A--D----------
Mycobacterium bovis	WP_031703943	SHPE	-AQ	-----C-----GA--D----------
Mycobacterium brisbanense	WP_062830253	---------------RGPK	VAH	---V-----------W----------
Mycobacterium canariasense	WP_062659962	--------------SRGAD	VAQ	---V----------------------
Mycobacterium canettii	WP_015303810	--------------TSHPE	--Q	-----C------A--D----------
Mycobacterium caprae	WP_075744592	--------------TSHPE	-AQ	-----C------A--D----------
Mycobacterium chelonae	WP_070947668	-------------------	A--	--------------------------
Mycobacterium chlorophenolicum	WP_048471577	----------------G-D	VAQ	---V----L------Y-------L--
Mycobacterium chubuense	WP_014814513	---------------RG-D	V-Q	---V----L------Y----------
Mycobacterium colombiense	WP_040630951	--------------TSHPD	--R	------E-----A--D----------
Mycobacterium cosmeticum	CDO10008	--------------SRGAD	VAQ	---V----------------------
Mycobacterium diernhoferi	WP_073859587	---------------RGPE	VAQ	---V----------------------
Mycobacterium europaeum	CQD19584	--------------TSHPD	S-H	------------A--D----------
Mycobacterium flavescens	WP_069414645	---------------RGPE	VA-	---V-A---------W----------
Mycobacterium fortuitum	WP_061262766	---------------RGPE	VAH	---V-----------W----------
Mycobacterium franklinii	WP_070938647	-------------------	---	-----------V--------------
Mycobacterium gastri	WP_036418215	----------I---TAHPE	SVQ	------------A--D----------
Mycobacterium gilvum	WP_011895655	--------------GLG-E	L-Q	---V----L------Y----------
Mycobacterium goodii	WP_049747955	---------------RGPE	VAD	---V-C---------W------S---
Mycobacterium gordonae	WP_065046324	--------------NAKPD	DVD	--I-----V---A--D----------
Mycobacterium hassiacum	WP_005625761	---------------RGPD	VAD	---V-----------Y----------
Mycobacterium heraklionense	WP_064996810	---------------KGPD	AVS	---V-----------W----------
Mycobacterium holsaticum	WP_069403791	---------------RGPA	VV-	---V-C---------W----------
Mycobacterium houstonense	WP_066903694	---------------RGPE	VAH	---V-----------W----------
Mycobacterium icosiumassiliens	WP_078058561	---------------KGPE	AVS	---V-A---------W----------
Mycobacterium immunogenum	WP_043076592	-------------------	---	--------------------------
Mycobacterium indicus pranii	WP_014942834	--------------TSHPD	--H	----E-E-----A--D----------
Mycobacterium interjectum	WP_066908017	--------------TSHPD	--H	--------V---A--D----------
Mycobacterium intermedium	WP_069420437	--------------TSHPD	--K	------E-----A--D----------
Mycobacterium intracellulare	WP_064934441	---------------RGPE	VAR	-SIV-A------A--C----------
Mycobacterium iranicum	WP_064283336	---------------RG-D	V-Q	---V-E--L------Y-------L--
Mycobacterium kansasii	WP_063466816	----------I---TAHPE	SVQ	------------A--D----------
Mycobacterium kumamotonense	WP_065289191	---------------KGPE	AVA	---V-----------W----------
Mycobacterium litorale	WP_078020524	--------------GRG-D	VA-	---V-----------Y----------
Mycobacterium malmesburyense	CRL78554	--------------TRGPE	VA-	---V-----------W----------
Mycobacterium malmoense	WP_065442972	--------------TSHPD	S-H	------E-----A--D----------
Mycobacterium marinum	WP_020732082	--------------TSHPE	SAQ	------------A--D----------
Mycobacterium mucogenicum	WP_064858201	--------------GYG-E	A--	---V-R---------W----------
Mycobacterium nebraskense	WP_046184923	--------------TSHPD	--H	------------A--D----------
Mycobacterium neoaurum	CDQ46252	---------------RGPE	AAQ	---V----V-----------------
Mycobacterium neworleansense	CRZ15752	---------------RGPE	VAH	---V-C---------W----------
Mycobacterium paraffinicum	WP_073872583	---------------RGPA	VAR	---V----V---A--C----------
Mycobacterium peregrinum	WP_064879419	---------------RGPE	VAH	--IV-C---------W----------
Mycobacterium phlei	WP_003886443	--------------SRGPE	IAD	---V-----------Y----------
Mycobacterium porcinum	WP_075921722	---------------RGPE	VAH	---V-C---------W----------
Mycobacterium pseudoshottsii L	GAQ31817	--------------TSHPE	SAQ	------------A--D----------
Mycobacterium rhodesiae	WP_014208936	---------------RGPE	VA-	---V-----------W----------
Mycobacterium rufum	KGI67229	----------------G-D	VAQ	---V----L------Y-------L--
Mycobacterium rutilum	SEH91663	---------------RGPE	VA-	---V----L------W----------
Mycobacterium salmoniphilum	WP_078323697	-------------------	A--	--------------------------
Mycobacterium saopaulense	WP_070909510	-------------------	---	--------------------------

Group	Species	Accession	Sequence	Insert	Sequence
Genus *Mycobacterium* (93/97)	*Mycobacterium scrofulaceum*	WP_067312334	--------------TSHPD	--H	------------A--D---------
	Mycobacterium septicum	WP_044516528	---------------RGPE	VAH	--IV-C---------W---------
	Mycobacterium setense	WP_039378347	---------------RGPE	VAH	--IV-----------W---------
	Mycobacterium sherrisii	WP_069399856	--------------TSHPD	--D	------E-----A--D---------
	Mycobacterium shimoidei	WP_069397703	-A-------------RGPD	VA-	---V--------A--C--------V-
	Mycobacterium sinense	WP_064856716	---------------KGPE	AVA	---V-A---------W---------
	Mycobacterium smegmatis	WP_011727859	---------------RGPE	VAD	---V-C---------W------S---
	Mycobacterium szulgai	WP_068033585	----------V---TSHPD	---	--I---E-----A--D---------
	Mycobacterium thermoresistibile	WP_003923610	---------------KGTE	V-	--IV-----------W---------
	Mycobacterium triplex	WP_036467155	--------------TSHPD	--D	------E-----A--D---------
	Mycobacterium triviale	WP_069391259	----------------GSD	AVG	-----A-----S---W---------
	Mycobacterium tuberculosis	WP_070892986	--------------TSHPE	--Q	A----C------A--D---------
	Mycobacterium tusciae	WP_006244294	---------------RGPE	IA-	---V-----------W---------
	Mycobacterium ulcerans	WP_011740603	--------------TSHPE	SAQ	------------G--D---------
	Mycobacterium vaccae	WP_003929482	---------------RG-D	V-Q	-----Q--L------Y-------L--
	Mycobacterium vanbaalenii	WP_041305954	--------------GLG-D	L-Q	--------L------Y---------
	Mycobacterium vulneris	WP_065510109	---------------RGPE	VAH	--IV-C---------W---------
	Mycobacterium wolinskyi	WP_067843149	---------------RGPK	VAQ	---V-----------W---------
	Mycobacterium yongonense	WP_020823438	--------------TSHPD	--H	----E-E-----A--D---------
	Mycobacterium abscessus subsp. bolletii	SIM07438	----G---------TLGAD		--L-ERQ-L--SA--T-V-----L--
	Mycobacterium xenopi	WP_003919164	---------------RDAQ		---V--------A--A---------
	Mycobacterium kyorinense	WP_045381260	---------------RDAQ		---V--------A--A---------
	Mycobacterium heckeshornense	WP_048891858	--------------TRDAQ		---V--------A--A---------
Other bacteria (0/>300)	*Hoyosella altamirensis*	WP_064440622	--------------Q-GAD		-----A-----------------Y--
	Hoyosella subflava	WP_013808635	--------------Q-GAD		-----A-----------------Y--
	Actinocatenispora sera	WP_030445011	--------------QLGAE		--LVER--S------Y---------
	Actinokineospora bangkokensis	WP_075977155	--------------Q-GAE		---VER--L------Y---------
	Actinopolyspora erythraea	WP_043575645	--------------QNGAD		--LVEAE-L--S---Y---------
	Actinosynnema mirum	WP_015805088	--------------Q-GAD		----ERE-L------Y------A---
	Aeromicrobium marinum	WP_007078465	----G---------RLGAE		---VER--L--ST--T-------L--
	Alloactinosynnema album	SDH59143	--------------Q-GAD		---VER--L------Y---------
	Allokutzneria albata	WP_030426894	--------------P-GAD		---VEDE-V--S---Y---------
	Amycolatopsis keratiniphila	WP_063272373	--------------Q-GAD		--IVER--L------Y---------
	Arsenicicoccus bolidensis	WP_034251837	----G---------QLGAT		---V-R--L---A--S------AL-S
	Asanoa ishikariensis	SDZ16546	--------------QLDAD		---VEA--A------S---------
	Blastococcus endophyticus	SE069839	------------G-SLGAE		---VER--V---S--T------A---
	Corynebacterium bovis	WP_043362011	-----------AG-KSGAD		---VEDR-M-----ID------S--S
	Dietzia alimentaria	WP_010542039	-----------QYGAD		--IV-------N---H---------
	Frankia discariae	WP_018501449	----G---------SLGAT		---------------T-------L--
	Haloglycomyces albus	WP_025272839	--------------QLNAE		--LVE-T-A------T---------
	Hamadaea tsunoensis	WP_027342626	--------------QLDAD		--IVEE--A------H---------
	Jiangella alba	WP_069110535	----G---------QLGAE		--LV-R--L--SA--T-------L--
	Kutzneria albida	WP_025361154	--------------P-GAE		---VER--L------Y-------Y--
	Lechevalieria aerocolonigenes	WP_045317840	--------------Q-GAD		---VEN--A------Y---------
	Micromonospora echinospora	SCE81996	--------------QLDAD		---VEAE-A------W---------
	Nakamurella lactea	WP_029138168	-------------VQYGAD		-SLVEDQ-A--S---L---------
	Nocardia cyriacigeorgica	WP_014349307	--------------Q-GAE		--L------------W---------
	Pseudonocardia asaccharolytica	WP_028930428	--------------Q-GSD		---VEP--M------D---------S
	Rhodococcus pyridinivorans	WP_019288536	-------------SQYGAT		-SL-----------------------
	Streptomyces lividans	WP_079021306	----G---------QLGAE		---V-C--L---S--T-------L--
	Tetrasphaera japonica	WP_048550881	----G---------QLGAE		--LVEK--V---A--T------ALV-
	Thermoactinomyces vulgaris	KPC74433	----G---------QLGAE		---V----L---S--T-------L--
	Tsukamurella paurometabola	WP_013125662	-------------V--GGD		---VEA---------Y---------
	Verrucosispora maris	WP_013731548	--------------QLDAD		---VEDE-A------S---------
	Williamsia muralis	WP_062800095	--------S--A--SYGAD		I--V-----------W---------

Supplementary Figure 6

A partial sequence alignment of a conserved region of NAD(P)H-quinone dehydrogenase showing a three amino acid insertion that is specific for most members of the genus *Mycobacterium* and absent in other bacteria including the two *Hoyosella* species.

Genus *Mycobacterium* (>100/>100)

		39	71
Mycobacterium abscessus	WP_062879058	GLARFSDICVAAFAD	VAIVKPQVAFFESYGAAG
Mycobacterium abscessus subsp. bolletii	EHM19575	---------------	-------------H----
Mycobacterium acapulcensis	WP_066809062	--R--CSV--T--S-	F-V---------A-----
Mycobacterium africanum	AMC63453	---A-C----R----	F-V---------------
Mycobacterium algericum	WP_083037395	---A-GET------G	F-----------A--S--
Mycobacterium alsense	WP_083138934	---A-C-T-IE---G	F-V---------A-----
Mycobacterium angelicum	WP_083114003	---A-C-V--E-Y-G	F-V---------A-----
Mycobacterium aromaticivorans	WP_036338022	------E---Q-Y-G	F-----------A--S--
Mycobacterium arosiense	WP_083067183	---A-C---IE--SG	--V---------A-----
Mycobacterium arupense	WP_046188070	-V-A--ET------G	F-V---------A--S--
Mycobacterium asiaticum	WP_065037660	---A-C----Q---G	F-V---------A--S--
Mycobacterium aurum	WP_083442999	-------L--T--EG	F-V---------A--S--
Mycobacterium avium	WP_062890323	---A-C----E---G	F-V---------A-----
Mycobacterium avium subsp. avium 2285	EUA32147	---A-C---IE--SG	F-V---------A-----
Mycobacterium bacteremicum	WP_083060227	--RA------Q----	----------Y-A--S--
Mycobacterium boenickei	WP_077739264	---A------T---G	F-M---------A--S--
Mycobacterium bohemicum	WP_085179917	---A-C----E---G	F-V---------A-----
Mycobacterium bovis	WP_019283631	---A-C----R----	F-V---------------
Mycobacterium branderi	WP_083133489	---A-C-T----YSG	F-A---------A-----
Mycobacterium brisbanense	WP_062831812	--R--C----T----	F-----------A-----
Mycobacterium canariasense	WP_062655572	--R--A----E--GG	F-----------AF--G-
Mycobacterium canettii	WP_014000739	---A-C----R----	F-V---------------
Mycobacterium celatum	WP_062538726	---A-C-T----Y--	F-V---------A-----
Mycobacterium celeriflavum	WP_083001533	--R--C-V--T---G	F-----------A-----
Mycobacterium chelonae	WP_070917543	----------E----	------------T-----
Mycobacterium chimaera	WP_072501364	---A-C---IE--SG	F-V---------A-----
Mycobacterium chlorophenolicum	KMO78082	----------T---G	--V---------A--S--
Mycobacterium chubuense	WP_014815654	------E------G-	F-V---------A-----
Mycobacterium colombiense	WP_064885698	---A-C---IDSLSG	F-V---------A-----
Mycobacterium conceptionense	WP_076212804	----------S---G	F-----------A--S--
Mycobacterium confluentis	WP_085157587	---A--A---E----	F-----------A--S--
Mycobacterium conspicuum	WP_085232539	---A-C-R-IE---G	L-V---------TH--L-
Mycobacterium diernhoferi	WP_073854623	------E--------	F-----------A--S--
Mycobacterium doricum	WP_085190743	--R--C----S---G	L-----------A-----
Mycobacterium elephantis	WP_046750341	--R--C-------EG	F-V---------A-----
Mycobacterium engbaekii	WP_085127823	--EA--QT--S---G	F-V---------A--S--
Mycobacterium europaeum	WP_085238724	---A-C----E--SG	F-V---------P-----
Mycobacterium fallax	WP_085094120	-----A-A--R---G	F-V---------A-----
Mycobacterium flavescens	WP_069412119	--R-------T----	I-V---------R--S--
Mycobacterium florentinum	WP_085221995	-V-K-C----E--SG	F-V------------SV-
Mycobacterium fortuitum	WP_061263936	---A------T---G	F-M---------A--S--
Mycobacterium fragae	WP_085194231	---A-C----Q-Y--	--V---------A-----
Mycobacterium franklinii	WP_070939537	----------E----	------------T-----
Mycobacterium gastri	WP_036415769	---A-C----Q-Y-G	F-V---------A-----
Mycobacterium genavense	WP_025735897	-V-K-C-------SG	F-V------------S--
Mycobacterium gilvum	WP_011894594	--S--A-L--T---G	F-V---------A--S--
Mycobacterium goodii	WP_049746989	--RA-CE--------	F-----------A--S--
Mycobacterium gordonae	WP_065042622	---A-C----D-Y-G	F-M---------A--S--
Mycobacterium haemophilum	WP_054880172	---A-C----E--SG	F-----------A-----
Mycobacterium hassiacum	WP_005627248	--Q--C-V--Q-Y--	F-----------Q-----
Mycobacterium heckeshornense	WP_048891752	---A-C-T--E-Y-G	F-V---------A-----
Mycobacterium heidelbergense	WP_083074300	---A-C-T--D---G	F-V---------A-----
Mycobacterium heraklionense	WP_064996962	---A--ET--T----	F-V---------A--S--
Mycobacterium hiberniae	WP_085134557	--EA--QS--S---G	F-V---------A--S--
Mycobacterium holsaticum	WP_069406338	--R--C-------TG	F-V---------T-----
Mycobacterium houstonense	WP_066897151	---A------T---G	F-M---------A--S--
Mycobacterium immunogenum	WP_064633574	---------------	------------------
Mycobacterium insubricum	WP_083030653	-----A-V--R-YSG	F-----------A-----
Mycobacterium interjectum	WP_085201952	---A-C----E---G	F-V---------T-----
Mycobacterium intermedium	WP_069418531	---A-C----Q-Y-G	F-A---------A-----
Mycobacterium intracellulare	WP_064936583	---A-C---IE--SG	F-V---------A--S--
Mycobacterium iranicum	WP_064285073	-------------GG	F-V---------A--SS-
Mycobacterium kansasii	WP_063470485	---A-C----R-Y-G	F-V---------A-----
Mycobacterium komanii	CRL74455	--R--C-A--T----	F-----------A-----
Mycobacterium kubicae	WP_085072991	--SA-C----E-YVG	F-----------A-----
Mycobacterium kumamotonense	WP_065288489	---A--ER--E--TG	F-V------------S--
Mycobacterium kyorinense	WP_065014003	---A-C-T--Q---Q	F-M---------A-----
Mycobacterium lacus	WP_085159783	---A-C---IE-Y-G	F-V---------------
Mycobacterium lentiflavum	CQD12611	-V-T-C----E---G	F-V------------S--

Group	Species	Accession	Sequence		
Genus *Mycobacterium* (>100/>100)	*Mycobacterium leprae*	WP_010907780	---A-C----E--SG		F----------A-----
	Mycobacterium lepromatosis	WP_045842537	---A-C----E--SG		F----------A-----
	Mycobacterium litorale	WP_078019699	-----C----Q-Y-G		F-V---------A--S--
	Mycobacterium llatzerense	WP_083420442	-V-A------E---G		F----------A--S--
	Mycobacterium longobardum	WP_085263900	---A--ET------G		F-V-----------S--
	Mycobacterium mageritense	WP_036441430	--R--C----E----		F----------A--S--
	Mycobacterium malmesburyense	CRL74313	--R--C-V-AT----		F----------V-----
	Mycobacterium malmoense	WP_065442394	---A-C----E--SG		F-V---------P-----
	Mycobacterium mantenii	WP_083094813	---A-C---IE--SG		F-V---------A-----
	Mycobacterium marinum	WP_020724944	---A-C--------G		F-V---------A-----
	Mycobacterium marseillense	WP_083019532	---A-C---IE--SG		F-V---------A-----
	Mycobacterium minnesotense	WP_083024805	------ET--T---G		F-------------S--
	Mycobacterium moriokaense	WP_083149643	-----CET--E--SG		F----------A-----
	Mycobacterium mucogenicum	OBA78456	-V-K------E---G		F----------A--S--
	Mycobacterium nebraskense	WP_046185657	---A-C-V--E--SG		F-V---------P-----
	Mycobacterium neoaurum	WP_030135263	--RT------Q----		----------Y-A--S--
	Mycobacterium neworleansense	CRZ14649	---A-----------		F-M---------A--S--
	Mycobacterium nonchromogenicum	WP_085138019	---A--ET--T----		F-V---------A--S--
	Mycobacterium noviomagense	WP_083085793	---S-C---IE-YGG		F-V---------A-----
	Mycobacterium novocastrense	WP_067389997	--R--C-V-------		F-V---------A-----
	Mycobacterium obuense	WP_046365761	--------------G		F-V---------A--S--
	Mycobacterium palustre	WP_085077854	---A-C----E---G		F-V---------AH----
	Mycobacterium paraense	WP_085102184	---A-C-T--E---G		F-V---------T-----
	Mycobacterium paraffinicum	WP_073872794	---A-C----E---G		F-V---------------
	Mycobacterium parafortuitum	WP_083145293	----------S--DG		F-V---------A--S--
	Mycobacterium paraseoulense	WP_083173978	---A-C----E---G		F-V---------A-----
	Mycobacterium parmense	WP_085269685	-----C----E--SG		F-V---------A-----
	Mycobacterium peregrinum	WP_064879717	---A----------G		F-M---------A--S--
	Mycobacterium persicum	WP_083155779	---V-C----R-Y-G		F-V-------------E-
	Mycobacterium phlei	WP_003889985	--R-------T---G		F-V---------R-----
	Mycobacterium porcinum	WP_075920908	--TA---A--T---G		F-M---------A--S--
	Mycobacterium pseudoshottsii	WP_086084663	---A-C--------G		F-V---------A-----
	Mycobacterium rhodesiae	WP_083122930	------E-----YTG		F----------A--S--
	Mycobacterium riyadhense	WP_085252078	---T-C----Q---G		F-V---------A--T--
	Mycobacterium rufum	KGI68063	------ET--T---G		F-V---------A--S--
	Mycobacterium rutilum	WP_083409060	--R-------T----		I-V---------R-----
	Mycobacterium salmoniphilum	WP_078327411	----------E----		------------T-----
	Mycobacterium saopaulense	WP_070910365	----------E----		------------------
	Mycobacterium saskatchewanense	WP_085257013	--SA-C-T--E---G		F-V---------A--S--
	Mycobacterium scrofulaceum	WP_067282683	---A-C---IE---G		F-V---------A-----
	Mycobacterium senuense	WP_085086893	---A--EA------G		F-V---------A--S--
	Mycobacterium septicum	WP_044518188	---A------T---G		F-M---------A--S--
	Mycobacterium setense	WP_064876756	---A-C----T---G		F----------T--S--
	Mycobacterium sherrisii	WP_069402169	---K-C----E---G		F-V---------------
	Mycobacterium shigaense	BAX92218	---T-C-T-IE--SG		F-V-----------S--
	Mycobacterium shimoidei	WP_069397294	---A-C----T---G		F-M-----------S--
	Mycobacterium shinjukuense	WP_083048458	---A-C----R---G		F-V---------A-----
	Mycobacterium simiae	WP_061559234	---K-C----E---G		F-V---------------
	Mycobacterium sinense	WP_064853921	---A--ET------G		F-V---------A--S--
	Mycobacterium smegmatis	WP_003894432	--RA-C--------G		F----------A--S--
	Mycobacterium szulgai	WP_068031662	--SA-C----E-YVG		F----------A-----
	Mycobacterium terrae	WP_085259578	---A--ET------G		F-V-----------S--
	Mycobacterium thermoresistibile	WP_003925444	--R-------T---E		L----------A-----
	Mycobacterium triplex	WP_036468577	-V-K-C-------SG		F-V-----------S--
	Mycobacterium triviale	WP_085108128	------E-----Y-G		F-V---------A-----
	Mycobacterium tuberculosis	WP_070892765	---A-C----R----		F-V---------------
	Mycobacterium tusciae	WP_083123466	--S--C----E---G		F----------A-----
	Mycobacterium ulcerans	WP_011739882	---A-C--------G		F-V---------A-----
	Mycobacterium vaccae	WP_081529169	---A--EL-----QE		F-V---------A--S--
	Mycobacterium vulneris	WP_065509368	---A------T---G		F-M---------A--S--
	Mycobacterium wolinskyi	WP_085141858	---A-C---------		F----------A--S--
	Mycobacterium xenopi	WP_085198518	---A-C-T--E-Y-G		F-V---------A-----
Other bacteria	*Hoyosella altamirensis*	WP_074390713	--VE--RR-AD---G	T	A----------A--SP-
	Hoyosella subflava	WP_041450980	--TE--RR-AD---E	T	A----------A--SP-
	Actinoalloteichus hymeniacidon	WP_069848753	--R--AE---E-L--	E	--ML---S-----H-SG-
	Actinokineospora bangkokensis	WP_075976532	--E--ALT--E-L-G	R	--L----S----AH-SR-
	Actinomyces nasicola	WP_073716258	--RA------RELDG	A	--AL--------T-----
	Actinophytocola xinjiangensis	WP_075130679	--E--AMT--E-M-G	E	I-VL---S----A--S--
	Aeromicrobium massiliense	WP_019146543	--D--AST--E---G	K	--FA---A----RH--R-
	Alloactinosynnema album	SDJ10178	--E--ALT--E-L-G	E	ISVL---S----A--SR-

Group	Species	Accession	Sequence
**Other bacteria**	*Amycolatopsis halophila*	WP_034270778	---Q-AQR----I-G E A-V----S----AF-S--
	Arthrobacter halophytocola	WP_060701655	-VRS--LTV-E-MVG A A--L-----LY-RF-S--
	Campylobacter concisus	WP_075466742	--R---E---E---G H A-L---------RF-S--
	Cellulomonas carbonis	WP_043610835	--R--A-TVTT-L-G R --AI---A----RH-S--
	Corynebacterium durum	WP_006063489	--R---R---E--GQ S --L-------Y-AF-SQ-
	Demetria terragena	WP_018155711	--R--C-TV-ESL-G Q --LA---S----RH--G-
	Dermatophilus congolensis	WP_028327961	--EV-AAT--E--GG Y --A----S----RF-S--
	Geodermatophilus soli	SDO71166	--R--T-AV-E-L-G T --VL---S--Y-RH-SR-
	Gordonia jacobaea	WP_049697280	--RQ-A----D-LGP V A-VI--------AF----
	Jatrophihabitans endophyticus	WP_073390650	--G--A-T--E--GG L A-V----S----T--S--
	Knoellia aerolata	WP_035932815	--SA-TAT--E---G H --V----S----AF-SR-
	Kutzneria albida	WP_025356276	--E--ALT--E---G E --V----S----AH-SR-
	Kytococcus sedentarius	WP_015779247	---A-T-R--K--SG R --V-----SL--AF-SD-
	Luteipulveratus mongoliensis	WP_052592350	--R--AE-S-E---G Q -------S----RH-S--
	Microbacterium paraoxydans	WP_060922166	-VRE-GLRT-E-A-G R -GV-----S---R-----
	Millisia brevis	WP_066903776	--E--AETV---T-G Q --V---------PF-S--
	Mobilicoccus pelagius	WP_009483412	---T-ASR--E---G T --C----S----RF-S--
	Nocardia coubleae	WP_067636061	--EA-AE---E--DG R --L---------T--SG-
	Phycicoccus dokdonensis	SDP05052	--R--T-T--E---G Q -GV----S----VF-SR-
	Pseudonocardia thermophila	WP_073454859	---A-AGA--E---G H --V----S----RH-S--
	Rhodococcus coprophilus	WP_072698363	--EK--EL--E--DG V --V---------VF-S--
	Saccharothrix espanaensis	WP_015103669	--E--ALT--E--GG H --V----A----A--SR-
	Sciscionella marina	WP_020501942	--E--ART--E--GS L AGVI---S-------S--
	Skermania piniformis	WP_066468316	---A-A----E---G R --L---------A-----
	Tetrasphaera jenkinsii	WP_048546357	--R--ART--E---G H --L----S----E--S--
	Tomitella biformata	WP_024796458	--GE-A----E--GG T --V---------AH-S--
	Turicella otitidis	WP_004600629	---E-TGR--E---G R --L---------RH--L-
	Williamsia muralis	WP_062798005	-VCA-G----E---G S --VI--------TF-S--

Supplementary Figure 7

A partial sequence alignment of a conserved region of orotidine 5'-phosphate decarboxylase showing a one amino acid deletion that is specific for members of the genus *Mycobacterium* and absent in most other bacteria including the two *Hoyosella* species.

Genus
Mycobacterium
(>100/>100)

		109	149
Mycobacterium africanum	WP_031666830	GFQRWRKALDRL	ETEVPVYLENTAGGDHAMARRFDTIARLW
Mycobacterium abscessus	CPU65096	--DN----I-A-	Q-D-T--I-------FS----L-R-EG--
Mycobacterium algericum	WP_083037205	--V-------Q-	--T--------------------------
Mycobacterium alsense	WP_083136040	--E--V----Y-	--D-Q---------------H----G---
Mycobacterium aromaticivorans	WP_036343453	--E--A---AQ-	--S-----------------H--------
Mycobacterium arosiense	WP_083063445	--E--V---NY-	KSD-Q---------------H----G---
Mycobacterium asiaticum	WP_065035248	--D--V--MKY-	--D-Q---------------Y----G---
Mycobacterium aurum	WP_087031425	--E--V----A-	N-D-----------------H--------
Mycobacterium avium	WP_062907968	--E--V----Y-	--D-Q---------------H----G---
Mycobacterium avium subsp. avium	EUA40617	--E--V----Y-	--D-Q---------------H----G---
Mycobacterium avium subsp. hominissuis	KDO93064	--E--V----Y-	--D-Q---------------H----G---
Mycobacterium avium subsp. silvaticum	ETB04189	--E--V----Y-	--D-Q---------------H----G---
Mycobacterium bacteremicum	WP_083055510	--E--A----Y-	--D-----------------H--------
Mycobacterium bohemicum	WP_085179222	--E--V----Y-	--D-----------------Y----G---
Mycobacterium bovis	WP_024457368	------------	-----------------------------
Mycobacterium branderi	WP_083130178	-CE-----ME--	--------------------H-E------
Mycobacterium brisbanense	GAS88772	--E--V----S-	K-D-----------------H--------
Mycobacterium canariasense	WP_062658106	--E--V----A-	N-D-----------------H--------
Mycobacterium canettii	WP_014000351	------------	-----------------------------
Mycobacterium celatum	WP_062539708	-CE-----ME--	--D-----------------H-E------
Mycobacterium chubuense	WP_014814204	-YE--V----Y-	-SD-----------------H----G---
Mycobacterium colombiense	WP_064883885	--E--V----Y-	--D-Q---------------H----G---
Mycobacterium conceptionense	CQD07569	--E--A----Y-	N-D-----------------H----S---
Mycobacterium conspicuum	WP_085233414	--E--A----Y-	--DMQ---------------Y----G---
Mycobacterium diernhoferi	WP_073856262	--E--V----A-	N-------------------H--------
Mycobacterium doricum	WP_085192435	-----V------	--T-----------V-----H----G---
Mycobacterium engbaekii	WP_085127725	--V---------	--T-----------EY----H-----K--
Mycobacterium europaeum	WP_085240544	--E--V----Y-	--DMQ---------------H----G---
Mycobacterium farcinogenes	CDP85145	--E--A----Y-	N-D-----------------H----S---
Mycobacterium flavescens	WP_069413162	--E--V----Y-	--D-----------------H--------
Mycobacterium florentinum	WP_085222916	--V-------Q-	-ST-----------------H--------
Mycobacterium fortuitum	WP_064895278	--E--A----YV	N-D-----------------H--------
Mycobacterium fragae	WP_085198004	--A--V----Y-	--D-QM--------------H----G---
Mycobacterium gastri	WP_036412186	--E--V----Y-	--D-----------------Y----G---
Mycobacterium genavense	WP_025738348	----------Q-	-ST-----------------H--------
Mycobacterium gilvum	WP_011895916	--E--V---AS-	K-D-----------------H--------
Mycobacterium goodii	WP_049748218	--T--V----A-	N-D-----------------H--------
Mycobacterium gordonae	WP_065046078	--E--V----Y-	--DMQI--------------Y----G---
Mycobacterium haemophilum	WP_047313546	--G-------Q-	Q-D--------------------------
Mycobacterium hassiacum	WP_005625364	--A-------T-	Q-D-----------------H--------
Mycobacterium heckeshornense	WP_048890290	-CE----TME--	--D-----------E-----H-E------
Mycobacterium heidelbergense	WP_083073974	--E--V----Y-	D-D-R---------------H----G---
Mycobacterium heraklionense	WP_064997876	--A---------	--T------------Y----H-----K--
Mycobacterium hiberniae	WP_085134903	--V---------	--T-----------EY----H-----K--
Mycobacterium holsaticum	WP_069403317	--E--V----Y-	--D-----------------H--------
Mycobacterium houstonense	WP_066901513	--E--V----A-	N-D-----------------H--------
Mycobacterium icosiumassiliensis	WP_067967176	--A---------	--T------------Y----H-----K--
Mycobacterium interjectum	WP_066907291	--E--V----Y-	----Q---------------Y----G---
Mycobacterium intermedium	WP_069420826	--E--V---KY-	--D-Q---------------Y----G---
Mycobacterium intracellulare	WP_064938885	--E--V----Y-	-SD-----------------H----G---
Mycobacterium iranicum	WP_064283909	--E--V---AS-	K-D-----------------H--------
Mycobacterium kansasii	WP_063469918	--E--V----Y-	--D-Q---------------Y----G---
Mycobacterium komanii	CRL75050	--E--V----Y-	--------------------H--V-----
Mycobacterium koreense	WP_085301962	--A-------Q-	DSD---L-------ER-------V-----
Mycobacterium kubicae	WP_085073654	--E--V---KY-	K-D-Q---------------H----G---
Mycobacterium kyorinense	WP_065014889	-CE-----M---	--D-----------------H-E------
Mycobacterium lacus	WP_085157534	----------Q-	H-------------------H--------
Mycobacterium lentiflavum	CQD06355	----------Q-	-ST-----------------H--------
Mycobacterium leprae	WP_010908592	----------Q-	Q-D--------------------------
Mycobacterium lepromatosis	WP_045843464	---------EQ-	Q-N--------------------------
Mycobacterium liflandii	WP_015354661	----------Q-	Q-D-----------E-----H--------
Mycobacterium litorale	WP_078017985	--E--A---AQ-	Q-D-----------------H--------
Mycobacterium llatzerense	WP_043984431	--E--V----A-	K-DM----------E-----H--------
Mycobacterium longobardum	WP_085265927	--A---------	--S-----------------H--------

Group	Species	Accession	Alignment	Boxed region	Alignment
Genus *Mycobacterium* (>100/>100)	*Mycobacterium malmesburyense*	CRL68028	--E--V----Y-		--D-----------------H--V-----
	Mycobacterium malmoense	WP_065441035	--E--V----Y-		N-D-----------------H----G---
	Mycobacterium mantenii	WP_083093275	--E--V----Y-		--D-R---------------Y----G---
	Mycobacterium marinum	WP_020731922	----------Q-		Q-D-----------E-----H--------
	Mycobacterium marseillense	WP_083018524	--E--V----Y-		--D-Q---------------H----G---
	Mycobacterium minnesotense	WP_083027775	--A-------S-		-S------------------H--------
	Mycobacterium moriokaense	WP_083156115	--E--V----Y-		--DMQI--------------H--V-G---
	Mycobacterium mucogenicum	WP_064857274	--E--V----A-		N-D-----------E-----Y--------
	Mycobacterium nebraskense	WP_046184737	--E--V----Y-		--D-Q---------------H----G---
	Mycobacterium neoaurum	WP_030134425	--E--A----Y-		--D-----------------H----G---
	Mycobacterium neworleansense	CRZ16173	--E--A----Y-		NSD-----------------H----S---
	Mycobacterium nonchromogenicum	WP_085138214	--A---------		--T------------Y----H-----K--
	Mycobacterium noviomagense	WP_083089173	-CE----TME--		--D-----------------H-E------
	Mycobacterium novocastrense	GAT11058	--E--V----Y-		--D-----------------H--V-----
	Mycobacterium obuense	WP_046361097	--E--V----Y-		--D-----------------H----G---
	Mycobacterium palustre	WP_085081091	--E--A----Y-		--D-Q---------------H----G---
	Mycobacterium paraense	WP_085097131	--E--V----Y-		-SD-Q---------------Y----G---
	Mycobacterium paraffinicum	WP_073878319	-----V----Y-		--D-----------------H----GK--
	Mycobacterium parafortuitum	WP_083145951	--E--V---AS-		N-D-----------------H--------
	Mycobacterium paraseoulense	WP_083171601	--E--V----Y-		--D-Q---------------H----G---
	Mycobacterium parmense	WP_085271915	--E--A--V-Y-		--D-Q---------------Y----G---
	Mycobacterium peregrinum	WP_064886323	--E--A----Y-		N-D-----------------H----S---
	Mycobacterium persicum	WP_083153140	--E--V----Y-		--D-----------------Y----G---
	Mycobacterium phlei	AMO60085	--E--V----Y-		Q-D-----------------H--------
	Mycobacterium porcinum	WP_075921660	--E--V----A-		N-D-----------------H--------
	Mycobacterium rhodesiae	WP_083120562	--E--A---AQ-		--S-----------------H--------
	Mycobacterium riyadhense	WP_085249142	--E--V---KY-		--D-QL--------------Y----G---
	Mycobacterium rufum	KGI66971	--E--A----Y-		--D-----------------H--------
	Mycobacterium rutilum	SEH46002	--E--V----Y-		--D-----------------H--V-----
	Mycobacterium saskatchewanense	WP_085258161	--E--V----Y-		K-D-----------------H----G---
	Mycobacterium scrofulaceum	WP_067274936	-----V----Y-		-SD-----------------Y----G---
	Mycobacterium senuense	WP_085088229	--V---T---Q-		--T--------------------------
	Mycobacterium septicum	WP_044516040	--E--V----A-		N-D-----------------H----S---
	Mycobacterium setense	WP_039315396	--E--V----A-		N-D-----------------H--------
	Mycobacterium sherrisii	WP_069399660	-Y---V----Y-		Q-D-----------------Y----G---
	Mycobacterium shigaense	BAX91021	----------Q-		-ST---F-------------H--------
	Mycobacterium shimoidei	WP_069395687	-CE-----ME--		Q-D-----------------H--------
	Mycobacterium shinjukuense	WP_083045959	----------Q-		H----------------------------
	Mycobacterium simiae	WP_061558312	--E--V----Y-		D-D-----------------Y----G---
	Mycobacterium sinense	WP_064855598	--V---------		--T-----------EY----------K--
	Mycobacterium smegmatis	WP_003892771	--T--V----A-		D-D-----------------H--------
	Mycobacterium szulgai	WP_068155966	--E--V---KY-		K-D-Q---------------H----G---
	Mycobacterium terrae	WP_085258900	--I---------		--T-----------E-----------E--
	Mycobacterium thermoresistibile	WP_003926019	-----V----Y-		--D-----------N-----H--------
	Mycobacterium triplex	CDO86986	----------Q-		-ST-----------------H--------
	Mycobacterium triviale	WP_085109910	--T-------Q-		-SD----V------QN----------G--
	Mycobacterium tuberculosis	WP_070893656	------------		-----------------------------
	Mycobacterium tusciae	WP_083124520	--E--V----Y-		--DI----------E-----H--V-G---
	Mycobacterium ulcerans	WP_011739021	----------Q-		Q-D-----------E-----H--------
	Mycobacterium vaccae	WP_003929614	--E--V---AS-		K-D-----------------H--------
	Mycobacterium vanbaalenii PYR-1	ABM12095	--E--V---AS-		KSD-----------------H--------
	Mycobacterium vulneris	WP_085291263	--E--V----Y-		--D-Q---------------Y----GK--
	Mycobacterium wolinskyi	WP_085146481	--V--V----A-		N-D-----------------H--------
	Mycobacterium xenopi	WP_003923339	-CE----TME--		--D-----------E-----H-E------
Other bacteria	*Hoyosella altamirensis*	WP_064440007	--TN---LFE-Q	DDEG	GFA--ILI-------S--S-Y--S-----
	Hoyosella subflava	WP_013809312	--TN---LFE-Q	DDEG	GFA--ILI-------S--S-Y--S-----
	Actinoalloteichus cyanogriseus	WP_026417887	-IEN---LFG-Q	HAEG	GFP---LV-------G-L-----VL----
	Actinokineospora bangkokensis	WP_075977687	-IDN---FFE-Q	DAEG	GFA--ILI-------G-------VL----
	Actinoplanes derwentensis	SDT73785	--TN----FEYA	ESEG	GLPL-ILI-------N-C-----DL----
	Actinopolyspora mzabensis	SDJ85483	-VAN---LFE-Q	AAEG	GF----LI------AN----G--DL----
	Amycolatopsis alba	WP_020632564	-LVN---LFE-E	QEKG	GFA--ILI-------S--T-DL-------
	Blastococcus endophyticus	SEP12122	-VDN---TFS-Q	AEAG	GFP--LLV------GN----DL-AL----
	Candidatus Blastococcus massil	WP_051515947	-VDN----FAYQ	QKDG	GFPL-LLI------GN----DL-ALD--F
	Couchioplanes caeruleus	WP_071810085	--DN---TFAYA	EKEG	GLPL--LI-------N-------NL----
	Cryptosporangium arvum	WP_035859664	--EN----FAQA	AESG	GFPL-ILV-------N-------RL-M--

**Other bacteria**	*Dactylosporangium aurantiacum*	WP_033359941	--DN---TFKFA ADEG GFAT--LI-------N-C-----AL----
	Geodermatophilus poikilotrophu	SET71270	-VDN---TFA-Q AEDG GFPL-LLI------GN----DL-AL----
	Gordonia hydrophobica	WP_066163809	--TN---LFE-Q EDKG GFG--ILI------------TLES-E---
	Hamadaea tsunoensis	WP_027342869	--DN---TFAYA QEQG GFA---LI----S--L-C--S-EAL----
	Kibdelosporangium phytohabitan	WP_054296252	-IGN---FFE-Q AKDG GFPL-ILI-------N-------VL----
	Kutzneria albida	WP_025361890	-LAN---LFE-Q ADSG GFA---LI-------N----K-EML----
	Lechevalieria aerocolonigenes	WP_030465687	--AN---FFE-Q VAEG GFA--ILI-------N----S--SLKL--
	Lentzea waywayandensis	SFR17552	--AN---FFE-Q VADG GFA---LI-------G----T--SLKL--
	Longispora albida	WP_018349018	--DN----FAYA ESEG GFPL-ILI-------N-C-----ALG---
	Micromonospora narathiwatensis	SBT53217	--DN---TFAYA AESG GFPL--LI-------N-C---L-AL----
	Millisia brevis	WP_066912325	-VEN---LFE-Q AEEG GFA--IWV-------R-------A-----
	Modestobacter caceresii	WP_036333083	-VDN---TFA-Q ADEG GFGL--LI-------N----EL-AV----
	Nakamurella multipartita	WP_041369525	--DN---VFE-Q AQEG GFP---LI-------K----TLEE-----
	Nocardia concava	WP_040811967	--VN---LFE-Q ADKG GFP--ILI------N-----Y--D-G---
	Pseudonocardia dioxanivorans	WP_013678626	--EN---FVE-Q QDEG GFA---FI-------N----S--AL----
	Rhodococcus kyotonensis	WP_068430944	--EN---LFE-Q AKDG GFG--IFI-------F-------D-----
	Saccharomonospora paurometabol	WP_028662471	-LTN---LFE-Q AEHG GFG--ILI------EG----DL-VLE---
	Saccharopolyspora rectivirgula	WP_029721405	-VAN---LFE-Q AEQG GF---ILI------AN----K--AL----
	Saccharothrix syringae	WP_033428230	-VAN---MFE-Q AEKG GFAT--LI-------N----T--ML----
	Salinispora pacifica	WP_028192827	--DN----FAYA AESG GFG---LI------TN-C---L-AL----
	Sciscionella marina	WP_020496730	-LGN---LFE-Q AKEG GFPTR-LI-------N----G--ML----
	Skermania piniformis	WP_066470289	-IDN---LFQ-Q EDKG GFG--ILI---------------------
	Smaragdicoccus niigatensis	WP_018159685	-IEN---LFE-Q EEKG GFA---LI-------F----Y--S-----
	Stackebrandtia nassauensis	WP_013021600	--AN----FEFG ADQG GFGL-ILI-------K-C---LEN-E---
	Streptoalloteichus hindustanus	WP_073479717	-VAN---LFE-Q ADAG GFP--ILI-------N-------ML----
	Tsukamurella paurometabola	WP_013125119	-IDN---LFT-Q EEKG GFG--ILI-------G-I--QLE--D---
	Verrucosispora sediminis	SFD48116	--DN-----SYA AESG GFGL--LI------EN-C---L-AL----
	Williamsia herbipolensis	WP_045821328	--AN---LFE-Q ADKG GFA--IFI------------TLGS-E---
	Xiangella phaseoli	SEJ74491	--DN-----TYA AEAG GFGL--LI------E--C---L-AL----
	Yuhushiella deserti	SFP84215	-IAN---LFE-Q AEQG GFA--LLI----S--N----SL-ML----

Supplementary Figure 8

A partial sequence alignment of a conserved region of deoxyribonuclease IV showing a four amino acid deletion that is specific for members of the genus *Mycobacterium* and absent in most other bacteria including the two *Hoyosella* species.

Genus *Mycobacterium* (>100/>100)

		122		166
Mycobacterium abscessus	WP_062879883	YLREAVLEPLGMSSTTLPG	GAD	TAGYGASSTVADLVAFVGDLLRP
Mycobacterium africanum	WP_061846059	--T---C-----VT-R-D-	-PA	A--F--T------TV-A------
Mycobacterium algericum	WP_083036220	--T---F------ASR-D-	--A	A--F-VT------A--A----A-
Mycobacterium alsense	WP_083137515	--A---C----LTA-R-D-	--A	A--F-MT------AV-AA-----
Mycobacterium angelicum	WP_083113555	--S---F-----RT-R-E-	--A	A--F------D--AV-AA-----
Mycobacterium arosiense	WP_083067008	--T---C-----AA-H-H-	--L	A--F--R------A--A------
Mycobacterium arupense	WP_046188342	--A---F-----TASR-D-	--A	R--F-GT--LT-----A----A-
Mycobacterium asiaticum	WP_065037501	--T---CA----TD-R-E-	--A	A--F--T------AV-A------
Mycobacterium aurum	WP_087024704	--G---F-----TA---R-	--A	A-----Y-S-T--AR-AA-----
Mycobacterium avium	WP_063967046	--H---CQ----TA-R-D-	--V	A--F--T------A--A------
Mycobacterium avium subsp. avium	ETB17155	--H---CQ----TA-R-D-	--V	A--F--T------A--A------
Mycobacterium avium subsp. hominissuis	ETB30335	--H---CQ----TA-R-D-	--V	A--F--T------A--A------
Mycobacterium bacteremicum	WP_083055791	------F-----A-SM-AE	--Q	A--F--E-S----TR-AA-----
Mycobacterium bohemicum	WP_085181505	------C-----AA-R-D-	--E	A-----T---------A------
Mycobacterium bovis	WP_014390302	--T---C-----VT-R-D-	-PA	A--F--T------AV-A------
Mycobacterium brisbanense	WP_062831364	--T-S-F------AS--T-	--E	A----GT------AK-ARE----
Mycobacterium canariasense	WP_062658009	------F-----T-SD-V-	---	A----GR------S--A-E----
Mycobacterium canettii	WP_015290533	--T---C-----VT-R-D-	-PA	A--F---------AV-A------
Mycobacterium chelonae	WP_070916267	------F-----GN-V---	---	-----GA---V-----A-E----
Mycobacterium chimaera	WP_069953797	--A---C-----AA-R-E-	--V	A--F--T------A--A------
Mycobacterium chlorophenolicum	WP_048471766	--A-S-CA-----D---E-	--E	A----VT---------A----A-
Mycobacterium chubuense	WP_048419182	--A-S-CA-----D---E-	-TE	A----VT---------A----A-
Mycobacterium colombiense	WP_064880513	--T---C-----AA-R-H-	--V	A--F--R------A--A------
Mycobacterium confluentis	WP_085150670	--T--IFQ--A--DSA---	--A	E--F--V------AS-A----A-
Mycobacterium conspicuum	WP_085231105	--A---C---R-T--R-D-	--E	A--F--T------A--A------
Mycobacterium cosmeticum	CD006555	------F-----ADSD-V-	---	A----GRA-----S--A-E----
Mycobacterium diernhoferi	WP_073853933	--S---F-----A-S--R-	--P	A--H-GYAS-T--A--A------
Mycobacterium doricum	WP_085191383	--A---F------DS--D-	--E	A-----Y-----VA--A------
Mycobacterium elephantis	WP_046754061	--T---F------A-A-T-	--S	A--F--T------T--AV-----
Mycobacterium engbaekii	WP_085128621	--S---F------ASR-D-	--A	A----GT------A--A------
Mycobacterium europaeum	WP_085242263	--A---C-----AA-R-D-	--P	A--F--T---------A------
Mycobacterium flavescens	WP_069412845	--T---F---A--DS--D-	--E	A-----T------A--A------
Mycobacterium fortuitum	WP_061264866	--T---F---Q-AKS--V-	--E	---F-GV------A--AAE----
Mycobacterium fragae	WP_085199734	--A---------AA-R-D-	-TE	A--F-VT--L---A--A------
Mycobacterium franklinii	WP_070935261	------------GN-----	---	------T---L-----A------
Mycobacterium gastri	WP_036412542	--TQ--F-----AT-R-H-	--A	A--F--T---T--AL-A------
Mycobacterium genavense	WP_025735044	--A---C---H-VA-R-A-	--A	E-----T------A--A------
Mycobacterium gilvum	WP_011893649	--A-S-FA--NLGD-D-D-	--E	A----VT---E-----A----T-
Mycobacterium goodii	WP_083453166	--T---F------ASM---	--E	A--F-VT------AV-A-E----
Mycobacterium gordonae	WP_065043654	--T---C-----T--R-D-	---	------D---D--AR-A------
Mycobacterium haemophilum	WP_054879789	--T---F---A-AA-K-ED	--W	A--F--T------A--AT-----
Mycobacterium hassiacum	WP_005628376	--T---FD----AD---D-	--A	A-----V------AR-A------
Mycobacterium heckeshornense	WP_048890595	--T---F---R-TASR-G-	-TW	A--F--T------TG-AA-----
Mycobacterium heraklionense	WP_064887533	--T---F-----TDSR-D-	--P	A--F-GT------A--A----S-
Mycobacterium hiberniae	WP_085135875	--S---F-----TASR-D-	--A	A----GT------A--A------
Mycobacterium holsaticum	WP_069404895	--T---F-A----A-S-T-	--S	A--F--T---T--A--AV-----
Mycobacterium houstonense	WP_066897767	--TQ--F---R---SV-S-	--Q	A--F--V------AV-A-E----
Mycobacterium icosiumassiliensis	WP_067974464	--T---F-----AASR-D-	--S	A--F-GT------A--AT-----
Mycobacterium immunogenum	WP_064632286	------------TG-----	---	-----GR---V-----A------
Mycobacterium indicus pranii	WP_043954755	--A---C-----AA-R-E-	--V	A--F--T------T--A------
Mycobacterium insubricum	WP_083028899	--A---FA----ADSS---	--E	-----GE------SR-AA---S-
Mycobacterium interjectum	WP_066913560	--A---C----LAA-R-T-	---	A--F--T------AV-A------
Mycobacterium intermedium	WP_069421222	--T---F-----TA-K-E-	-TA	A--F-V-------A--A------
Mycobacterium intracellulare	WP_064937161	--A---C-----AA-R-E-	--V	A--F--T------A--A------
Mycobacterium iranicum	WP_024445352	--T-S-F-----TA-D-E-	-N-	A----VT---D-----A----A-
Mycobacterium kansasii	WP_063466593	--T---F-----AA-R-D-	-TG	A--F--T------AL-A------
Mycobacterium komanii	CRL72845	--T---F-----TDSA-D-	--A	A-----T------AV-A------
Mycobacterium koreense	WP_085302077	--H--ICV-----A-R-D-	--A	A-----T------A--AT-----
Mycobacterium kubicae	WP_085074576	--N---C-----TT-R-D-	--P	A--F--T------A--AR-----
Mycobacterium kyorinense	WP_065014648	--T---F-----TT-A-V-	--Q	A--F-VR--LS--T--AA-----
Mycobacterium lacus	WP_085161815	-VA---FG----AA-R-D-	-TW	A--F--T------AV-A------
Mycobacterium lentiflavum	CQD16443	--T---C---S-RA-R-D-	--A	E-----T--I---A--A------
Mycobacterium leprae	WP_010908532	--T-------A-TA-K-ED	-TW	A--F--T------A--AN-----
Mycobacterium lepromatosis	WP_045842608	--T---F---A--A-K-KD	--W	A--F--T------A--AS-----
Mycobacterium liflandii	WP_015356267	--A---F------TSK-E-	--A	A--F--T---G--AL-A------
Mycobacterium litorale	WP_078021925	--A--IF-------SR-D-	--A	AS-F--V------S--A----A-
Mycobacterium llatzerense	WP_071286880	------F------DSD---	-S-	------W---T--A--A------
Mycobacterium longobardum	WP_085263949	--S---F------ASR-D-	--A	A--F-GT------A--A----V-
Mycobacterium malmesburyense	CRL71451	--T---F-----TDSA-D-	--A	------T--T---AV-A------
Mycobacterium malmoense	WP_071511991	--A---C---A-A--R-D-	--A	A--F--T--A---AV-A------
Mycobacterium mantenii	WP_083099802	--T---C-----AA-S-D-	--V	A--F--R------A--A------
Mycobacterium marseillense	WP_083020439	--A---C-----TT-R-E-	--V	A--F--T---T--A--A------
Mycobacterium minnesotense	WP_083022507	--T---F-----TASR-D-	--A	A--F-GI--L------A----A-

**Genus *Mycobacterium* (>100/>100)**	*Mycobacterium moriokaense*	WP_083157650	--A--LF-----ADSA-Q-	--E	V----VV---------A------
	Mycobacterium mucogenicum	WP_064983531	------F------DSD---	A-E	------W---T--A--A------
	Mycobacterium nebraskense	WP_046184109	--A---CQ----AA-R-D-	--P	A--F--T------AV-A------
	Mycobacterium neoaurum	CDQ43143	------F-----TGSS-A-	---	A--H-GQ-C-D---R-A------
	Mycobacterium nonchromogenicum	WP_085136999	--T---F-----TASR-D-	--S	A--F-GT------A--A----V-
	Mycobacterium noviomagense	WP_083089024	--S---F---T-AA-R-D-	--E	V--F--T------A--AV-----
	Mycobacterium obuense	WP_046363412	--T-S-FD--Q--D---D-	--P	A-----T------T--A-E--T-
	Mycobacterium palustre	WP_085078176	--A---C-----AA-R-A-	--A	A--F--T------A--A------
	Mycobacterium paraffinicum	WP_073875470	--A---F-----A--R-D-	--A	A--F--V------AK-A------
	Mycobacterium parafortuitum	WP_083146632	--A---FT----TA-HFD-	-TE	A-----T---T--I--AA---A-
	Mycobacterium paraintracellulare	AFC53589	--A---C-----AA-R-E-	--V	A--F--T------A--A------
	Mycobacterium paraseoulense	WP_083172785	--A---C-----AA-R-D-	--A	A--F--T------A--AR-----
	Mycobacterium parmense	WP_085271306	--A---C-----TT-R-D-	--A	E-----T------A--A------
	Mycobacterium peregrinum	WP_064878042	--T---F---Q-TKS--L-	-TQ	A--F-GV---G--A--ARE----
	Mycobacterium phlei	AMO62207	--T---F-----ADSV-E-	--A	A----VT-------R-A------
	Mycobacterium rhodesiae	WP_083121454	--A---F-----A-SR-D-	--Q	V--F-GL---------AA---A-
	Mycobacterium riyadhense	WP_085250046	-FI---C-----AT-H-E-	--A	---F--T--I---A--A------
	Mycobacterium rufum	KGI68846	--A-S-FA-----D---D-	-TE	A-----T---D-----A----A-
	Mycobacterium rutilum	WP_083408303	--T---F---A-TDS--D-	--A	A----VT------A--A------
	Mycobacterium salmoniphilum	WP_078327537	------F-----GD-V---	---	-----GA---V--A---------
	Mycobacterium saopaulense	WP_070911056	------F-----GH-V---	---	-----G----V------------
	Mycobacterium saskatchewanense	WP_085254598	--T---C---A-VA-R-N-	-TE	A--F--T------A--A------
	Mycobacterium scrofulaceum	WP_067269522	--A---CQ----A--R-E-	--A	A--F--V------AK-A------
	Mycobacterium senuense	WP_085082835	--T---F------ASR-D-	--A	A--F-VT------A--A----A-
	Mycobacterium setense	WP_064875010	--T---F---Q--AS--L-	--E	A----GV------A--AKE----
	Mycobacterium sherrisii	WP_085168208	--A---C-----TA-R-D-	--Q	E--F--------MA--A------
	Mycobacterium shigaense	BAX93320	-VT---C-----A--R-D-	--A	A--F--T---T--A--A------
	Mycobacterium shimoidei	WP_069396230	--A---F-----AA-R-D-	--R	A--F-VT--LG-MA--AV---E-
	Mycobacterium shinjukuense	WP_083046528	--A---C-----AT-R-D-	-PA	V--F--T------A--AA-----
	Mycobacterium simiae	WP_061556117	--C---C-----AA-R-D-	--P	H--F--------MA--A------
	Mycobacterium sinense	WP_064920789	--T---F------ASR-D-	--A	A--F--T------A--A----A-
	Mycobacterium smegmatis	WP_003895732	--T---F------TSK-L-	---	A--F-VT---S--AR-A------
	Mycobacterium szulgai	WP_085670930	------F-----GT-R-E-	--A	---F--T---G--A--AA---S-
	Mycobacterium terrae	WP_085261093	--T---F---A--ASR-D-	--A	A--F--T------A--A----A-
	Mycobacterium thermoresistibile	WP_003924638	-FH---C------D-A---	--A	A--F--G------AL-AV-----
	Mycobacterium triplex	WP_036469964	--A---C---N-GA-R-D-	--S	E-----T------A--A------
	Mycobacterium triviale	WP_069391498	--H--ICV-----A-R-D-	--A	A-----T------A--AT-----
	Mycobacterium tuberculosis	WP_063738383	--T---C-----VT-W-D-	-PA	A--F--T------AV-A------
	Mycobacterium tusciae	WP_083125359	--T---C----L-D---A-	--E	A--F--T---------A------
	Mycobacterium ulcerans	WP_011739476	--A---F------TSK-E-	--A	A--F--T---G--AL-T------
	Mycobacterium vaccae	WP_003933262	--A-------R-GD---D-	-TE	A----VT---D---S-A----A-
	Mycobacterium vulneris	WP_085291726	--T---C------A-E-H-	--V	A--F--R---T--A--A------
	Mycobacterium wolinskyi	WP_067855213	--T---F-----AASK-L-	--E	V--F-GV------A--ASE----
	Mycobacterium xenopi	WP_085196125	--T---F---Q-TASR-D-	-TW	A--F--T------TE-AA-----
	Mycobacterium yongonense	WP_065501022	--A---C-----AA-R-E-	--V	A--F--T------A--A------
**Other bacteria (0/>500)**	*Hoyosella altamirensis*	WP_064439259	---LG-F-----E--A-D-		P--H--V---D--AR-AEE--E-
	Hoyosella subflava	WP_013806021	---LG-F-----E--A-E-		P--H--V---D--AR-AEE--E-
	Actinoalloteichus hymeniacidonis	AOS63826	--A---F------------		SPAS--M--A---AR-AAE-QN-
	Actinokineospora enzanensis	WP_026316223	------F-----GASE-T-		S--A--V--C---TL-AAE-QA-
	Actinomycetospora chiangmaiensis	WP_018330455	------C-----G--S-E-		S--H--T---D---LLARE--T-
	Actinosynnema mirum	WP_012782788	--A----G----G--A-E-		SPAA-GV---------AAEVQS-
	Alloactinosynnema album	SDI53291	--A-G-FA----AASV-V-		S--A--E--CG--AR-AVE-QS-
	Amycolatopsis jejuensis	WP_033291652	-Q---L-A----TA-R-D-		SPAS--V--LD--LR-AAE-QS-
	Arthrobacter crystallopoietes	SDR08107	---DG--L----QQ-S-E-		SPAA--T---T--SR-AAE-QQ-
	Corynebacterium lactis	WP_053412493	------F-----DASA-K-		S--H--R-S-R--M---DEVFS-
	Dietzia cinnamea	WP_043567217	--H---CR----E--A-E-		S--H---ASL----P-ARE-VA-
	Frankia elaeagni	WP_018638971	--A---FV----KAAA-T-		SPAF-VLA-L--VT--AAE--A-
	Gordonia effusa	WP_007318587	--H---CA-----R-E-S-		P--H--R-C----A--A----A-
	Jiangella muralis	WP_053205614	-V-DGL-G----TG-VFE-		SPAS--R------AW--AE-MA-
	Kibdelosporangium aridum	WP_033390269	--A---FA----DD---K-		SPAHAGV--C---SK-AAE--K-
	Lechevalieria fradiae	SDH26015	------FV----K-SA-V-		S--A--E-CA---AL-AAE--S-
	Lentzea waywayandensis	SFR16291	--H---FA----N-SA-V-		S--A--E-SA---AL-ASE--S-
	Leucobacter komagatae	WP_042543328	----GL------TA-S-E-		EPAA-GV--A---SR-AAE-QA-
	Microbacterium oleivorans	WP_060915720	-AH---FA----A--SIGD		S-AA-G--ST---AR-ARE-QE-
	Millisia brevis	WP_066907285	-----IF-----A-SA-V-		P--S--E---R--SR-AAE--A-
	Nocardia concava	WP_040804011	------F-------SA-V-		P--HAGR-S----LR-A-E--E-
	Paeniglutamicibacter antarcticus	WP_068733340	--T----N----D--SVN-		S-AADGV--A---LK-AAE-QN-
	Prauserella rugosa	WP_030531817	--S-G--Q----TN--STD		SP-A-SA---D---R-AAE-QS-
	Pseudonocardia dioxanivorans	WP_041760352	--H---CV---LGG-R-E-		HP-S--V---D--AR-AAE-QA-
	Rhodococcus gordoniae	WP_064064124	---Q--F-----R-SS---		P--H--Q-S----AL-ARE--T-

**Other bacteria (0/>500)**	*Saccharomonospora marina*	WP_009154976	-QQ--L-D--R--R-R-E-	SP-S------D-----AAE-QS-
	Saccharothrix syringae	WP_033433415	--A---FA----T-SS-D-	SPAA--T---S---R-AAE-QA-
	Sanguibacter keddieii	WP_012867114	-AH----V--A-T--DVS-	SPAA---------AR-AAE-QQ-
	Sciscionella marina	WP_026198095	------CV---LVA-E-R-	S--A--V--A-E-AV-A-E--N-
	Skermania piniformis	WP_066468967	--A---F-----RA-V-A-	S---A-R--M---I--AAE-QA-
	Smaragdicoccus niigatensis	WP_018161452	------F-----TR-M-V-	S--H--R------TY-L-E--Q-
	Streptomyces flocculus	WP_055498306	-------S----TA-E-E-	S-AKD-V-SC---AR-AAE-QS-
	Thermocrispum municipale	WP_028849240	-HQ--L-T----T--K-A-	SP-ADGY--A----R-TKE-Q--
	Timonella senegalensis	WP_019147966	-AH---F-----A----GT	SPAA-GL-S-S--SR-AQE--A-
	Tomitella biformata	WP_024793231	--A---F-----A----W-	V--HE-R-S----SR-ALE--N-
	Williamsia herbipolensis	WP_045823152	--H---CA---LGA-A-S-	P--H--E-S-D--AV-AAE--A-

Supplementary Figure 9

A partial sequence alignment of a conserved region of serine hydrolase showing a three amino acid insertion that is specific for members of the genus *Mycobacterium* and absent in other bacteria including the two *Hoyosella* species.

Genus *Mycobacterium* (>100/>100)

		279		327
Mycobacterium abscessus	WP_070410295	HVPIQRMANVSLQASPEERS		TDDLISGVEDGIYIVGDKSWSIDMQRYN
Mycobacterium africanum	WP_003910538	---------I---PGI-DL-		-A---GR-D-------------------
Mycobacterium algericum	WP_083036271	-------------PGA-NV-		-A---AR-D--------R----------
Mycobacterium alsense	WP_083137224	-------------PAA-DI-		--E--GH---------------------
Mycobacterium angelicum	WP_083116295	-------------PGTDNL-		-E---GR-D-------------------
Mycobacterium aromaticivorans	WP_036344501	-------------PGA-DL-		------R-S-------------------
Mycobacterium arosiense	WP_083067118	-------------PAQ-DI-		-----GR---------------------
Mycobacterium arupense	WP_046189582	-------------PG--DV-		-V---GR-D--------R----------
Mycobacterium asiaticum	WP_065034239	-------------PG-QDL-		-A----RID-------------------
Mycobacterium aurum	WP_087024763	-------------PGT-DL-		-E---AR-S-------------------
Mycobacterium austroafricanum	WP_036372126	-------------PG--DL-		-A---AQ-S----V--------------
Mycobacterium avium	WP_065371105	-------------PA--QI-		-----AR---------------------
Mycobacterium avium subsp. paratuberculo	ETB52477	-------------PA--QI-		-----AR---------------------
Mycobacterium bacteremicum	WP_083056061	-------------PG--DL-		-A----R-S-------------------
Mycobacterium boenickei	WP_077741272	-------------PG-DDL-		-Q---AR-S----V--------------
Mycobacterium bohemicum	WP_085182897	-------------PAA-DI-		--A--AR-Q-------------------
Mycobacterium branderi	WP_083133352	-------------P-AD-LT		-A---GR-D-----------------H-
Mycobacterium brisbanense	WP_062827453	-------------PGA-DL-		-A----R-S----V--------------
Mycobacterium canariasense	WP_062655329	-------------PGA-DL-		-A---AR-H-------------------
Mycobacterium canettii	WP_080627439	---------I---PGI-DL-		-A---GR-D-------------------
Mycobacterium celatum	WP_062539876	-------------PGTD-L-		-A---GR------V--------------
Mycobacterium celeriflavum	WP_083001194	-------P----EPAADDV-		-----AR-D-------------------
Mycobacterium chelonae	WP_070916292	----------------D---		----------------------------
Mycobacterium chimaera	WP_089151607	-------------PA--DI-		--E--GR---------------------
Mycobacterium chlorophenolicum	WP_048468407	-------------P--DDV-		-E---AR-S-------------------
Mycobacterium chubuense	WP_041783157	-------------PG-DDV-		--E--AR-C--L----------------
Mycobacterium chubuense NBB4	AFM18069	-------------PG-DDV-		--E--AR-C--L----------------
Mycobacterium colombiense	WP_064878414	-------------PA-DDI-		-----GR---------------------
Mycobacterium conceptionense	WP_064899263	-------------PG--DV-		--------S-------------------
Mycobacterium confluentis	WP_085151297	-------------PAA-DI-		-E----R-S-------------------
Mycobacterium conspicuum	WP_085231339	-------------P---DI-		-A---GR-Q-------------------
Mycobacterium cosmeticum	WP_036396099	-------------PGS-DL-		-A---AR-R-------------------
Mycobacterium diernhoferi	WP_073853834	-------------PGA-DL-		-A---AR-S-------------------
Mycobacterium doricum	WP_085189977	-------------PAL-DVG		-----AR-D--L----------------
Mycobacterium elephantis	WP_083043350	-------------PAA-DV-		-E---AR-D-------------------
Mycobacterium europaeum	WP_085241935	-------------PAR-DV-		-A---GR---------------------
Mycobacterium fallax	WP_085100567	------------APAA-DIA		-E----R-D--L----------------
Mycobacterium farcinogenes	CDP89149	-------------PG--DV-		------R-S-------------------
Mycobacterium flavescens	WP_069413032	------------EPAA-DV-		-E---AR-S--L----------------
Mycobacterium florentinum	WP_085221322	-------------PG-DDL-		-G---AR--N------------------
Mycobacterium fortuitum	WP_064900328	-------------PGR-DL-		--E---R-S-------------------
Mycobacterium fragae	WP_085199698	-------------P---PI-		-A---DR-D-------------------
Mycobacterium franklinii	WP_070936784	----------------D---		----------------------------
Mycobacterium gastri	WP_036415472	-------------PGS-DL-		-A----R-R-------------------
Mycobacterium genavense	WP_025737935	-------------PG-DDL-		-E---GH-DN------------------
Mycobacterium gilvum	WP_011893612	-------------PA--DV-		-EE--GR-S--L----------------
Mycobacterium goodii	WP_049745926	-------------PAA-DL-		------R-S-------------------
Mycobacterium gordonae	WP_065043113	-------------PG--DL-		-E----RID-------------------
Mycobacterium haemophilum	WP_054879747	-------------PG-D-IT		-A---AR-D-------------------
Mycobacterium hassiacum	WP_005628443	-------------PA--DI-		-E---AR-D-------------------
Mycobacterium heckeshornense	WP_048890618	-------------PGA-DL-		-----GR-D-------------------
Mycobacterium heraklionense	WP_064997586	-------------PGT-DL-		-A----R-D--------R----------
Mycobacterium holsaticum	WP_069403201	-------------PAT-DV-		-EE--AR-D-------------------
Mycobacterium houstonense	WP_066898246	-------------PA--DI-		------R-A-------------------
Mycobacterium immunogenum	WP_064632230	----------------D---		-S--------------------------
Mycobacterium indicus pranii	WP_014941698	-------------PA--DI-		--E--GR---------------------
Mycobacterium insubricum	WP_083028871	------------APA--DI-		----V-R-D-------------------
Mycobacterium interjectum	WP_085205285	-------------PG--DV-		-AE--GR---------------------
Mycobacterium intracellulare	WP_064934251	-------------PA--DI-		--E--GR---------------------
Mycobacterium iranicum	WP_064282313	-------------PG-DDI-		-AE---R-S--L----------------
Mycobacterium kansasii	WP_063468229	-------P-----PG--DL-		-A---GR-A-------------------
Mycobacterium komanii	CRL70718	-------------P-A-DV-		-E----R-D-------------------
Mycobacterium koreense	WP_085302127	-------------PG-QDL-		-A----R-D----V---R----------
Mycobacterium kyorinense	WP_065012593	-------------PGRD-L-		-A---AK-D-----I-------------

Group	Species	Accession	Sequence		Sequence
**Genus *Mycobacterium* (>100/>100)**	*Mycobacterium lentiflavum*	CQD17000	-------------PG-DDL-		-----GR-Q-------------------
	Mycobacterium liflandii	WP_015356429	-------------PGA-DL-		-A---GR---------------------
	Mycobacterium litorale	WP_078018779	-------------PGT-DL-		-E---GR-A-------------------
	Mycobacterium llatzerense	WP_071286913	-------------PG-DDL-		-----GR--N------------------
	Mycobacterium longobardum	WP_085263444	-------------PGT-DL-		-A---AR-D--------R----------
	Mycobacterium mageritense	WP_036443423	-------------PGR-DL-		------R-S--L----------------
	Mycobacterium malmesburyense	CRL71544	------------EP-AHDV-		I----AR----L----------------
	Mycobacterium malmoense	WP_065442617	-------------PAR-DV-		-A---GR---------------------
	Mycobacterium mantenii	WP_083099925	-------------PA-D-I-		-----GR---------------------
	Mycobacterium marinum	WP_020725888	-------------PGA-DL-		-A---GR---------------------
	Mycobacterium marseillense	WP_083017801	-------------PA--DIT		--E---R--E------------------
	Mycobacterium moriokaense	WP_083155728	-------------PGTADL-		-----AR---------------------
	Mycobacterium mucogenicum	WP_064860187	-------------PGTDDL-		------R--N------------------
	Mycobacterium nebraskense	WP_046182078	-------------PAR-DV-		-A---GR-------I-------------
	Mycobacterium neoaurum	WP_030133580	-------------PAT-DL-		-A----R-S-------------------
	Mycobacterium neworleansense	CRZ13647	-------------PGL-DL-		-A----R-S----V--------------
	Mycobacterium nonchromogenicum	WP_085137075	-------------PGT-DL-		-A----R-D--------R----------
	Mycobacterium noviomagense	WP_083088979	-------------PG--DV-		-E---GR---------------------
	Mycobacterium novocastrense	WP_067389667	------------EP-AD-V-		-E---AR-D----V--------------
	Mycobacterium obuense	WP_046363596	-------------PAA--V-		-E---AR---------------------
	Mycobacterium palustre	WP_085080063	-------------PA--DIT		-A---GR--N------------------
	Mycobacterium paraense	WP_085095349	-------------PG--DL-		-A---GR---------------------
	Mycobacterium paraffinicum	WP_073880970	-------------PAR-DV-		-A----R-------I-------------
	Mycobacterium parafortuitum	WP_083146810	------------RPGA-DL-		------R-S--L----------------
	Mycobacterium paraintracellulare	WP_014384412	-------------PA--DI-		--E--GR---------------------
	Mycobacterium paraseoulense	WP_083175082	-------------PARD-V-		-A---AR-R-------------------
	Mycobacterium parmense	WP_085269058	------------RPAH-DV-		-A---GR---------------------
	Mycobacterium peregrinum	WP_064877955	-------------PG--DL-		------R-S-------------------
	Mycobacterium phlei	WP_061481797	-------------PAA-DI-		-----AR---------------------
	Mycobacterium porcinum	WP_069426579	-------------PG--DL-		-Q----R-S----V--------------
	Mycobacterium pseudoshottsii	WP_086085485	-------------PGA-DL-		-A---GR---------------------
	Mycobacterium rhodesiae	WP_083121405	-------------PGT-DL-		------R--N------------------
	Mycobacterium rufum	KGI68888	-------------PG--DV-		-E---AR-A-------------------
	Mycobacterium rutilum	WP_083410615	------------EPAA-DV-		-A---AC----L----------------
	Mycobacterium salmoniphilum	WP_078325628	----------------D---		----------------------------
	Mycobacterium saopaulense	WP_070911084	--------------------		------A---------------------
	Mycobacterium saskatchewanense	WP_085257418	-------------PA--DI-		-A---GR---------------------
	Mycobacterium scrofulaceum	WP_067272416	-------------PAR-DV-		-A----R-------I-------------
	Mycobacterium senuense	WP_085083712	-------------PG--DL-		-A---AR-D--------R----------
	Mycobacterium septicum	WP_044523255	-------------PG--DL-		-Q----R-S----V--------------
	Mycobacterium setense	WP_064874943	-------------PG-QDL-		-Q----R-Y----V--------------
	Mycobacterium shigaense	BAX93436	-------------P--DDL-		-----AR-Q-------------------
	Mycobacterium shimoidei	WP_069397039	-------P-----PGRDDL-		-A-----ID-------------------
	Mycobacterium sinense	WP_064922002	-------------PGT-DV-		-A----R-D--------R----------
	Mycobacterium smegmatis	WP_011729853	------------RPAA-DL-		-----AR-S--L----------------
	Mycobacterium szulgai	WP_068029737	-------------PGTDDL-		-A---AR-D-------------------
	Mycobacterium terrae	WP_085261136	-------------PG-ADL-		-A---AR-D--------R----------
	Mycobacterium timonense	WP_083187068	-------------PA--QI-		-----AR---------------------
	Mycobacterium triplex	WP_036470173	-------------PG-DDL-		-E---GR--N------------------
	Mycobacterium triviale	WP_069391529	-------------PG-QDL-		-A----R-D----V---R----------
	Mycobacterium tuberculosis	WP_064792902	---------I---PGI-DL-		-A---GR-D-------------------
	Mycobacterium tusciae	WP_083125326	-------------PGTD-L-		-----AR-Q-------------------
	Mycobacterium ulcerans str. Harvey	EUA91505	-------------PGA-DL-		-A---GR---------------------
	Mycobacterium vaccae	WP_003933300	-------------PAAQDV-		-A---A--S--L----------------
	Mycobacterium vanbaalenii	WP_011780972	-------------PGA-DL-		-A---AQ-S--L-V--------------
	Mycobacterium vulneris	WP_065462253	-------------PG--DL-		-Q----R-S----V--------------
	Mycobacterium wolinskyi	WP_085148872	-------------PG-DDL-		-E----R-S--L----------------
	Mycobacterium xenopi	WP_085194966	-------------PGV-DL-		-----GR-D-------------------
	Mycobacterium yongonense 05-1390	AGP63369	-------------PA--DI-		--E--GR---------------------
**Other *Corynebacteriales***	*Millisia brevis*	WP_066906399	---L---------PD--SDR	T	-----AR--S------------------
	Nocardia arthritidis	WP_063052842	-------------PD-DRDT	S	-EE---R-D--L----------------
	Nocardia asiatica	WP_043726271	-------------PD-DHDT	T	-EE---R-D--L----------------
	Nocardia asteroides NBRC 15531	GAD85803	-------------PD--RDT	S	-AE---R---------------------
	Nocardia beijingensis	WP_067807594	-------------PD-DRDT	S	-EE---R-D--L-----R----------
	Nocardia inohanensis	WP_067822563	-------------PD-DRDT	S	-AE---R--N------------------

Other *Corynebacteriales*	*Nocardia jejuensis*	WP_067686925	-------------PD-DNDT S -AE---R--N-L----------------
	Nocardia jiangxiensis	WP_040823029	-------------PD--HDT S --E---R--N---V--------------
	Nocardia jinanensis	WP_058855627	-------------PD-DVDT S -AE---R-S---F---------------
	Nocardia kruczakiae	WP_063011533	-------------PD-QTDT S -AE---R----------R----------
	Nocardia lijiangensis	WP_067843959	-------------PD-VRDT S -EE---R----L----------------
	Nocardia mexicana	WP_068020331	-------------PD--RDT T -E----R-D-------------------
	Nocardia mikamii	WP_062997058	-------------PD-QTDT S -AE---R------V---R----------
	Nocardia miyunensis	WP_067666508	-------------PD--HDT S -AE---R--N---V--------------
	Nocardia niigatensis	WP_040857950	-------------PD--RDT S -AE---R--N------------------
	Nocardia niwae	WP_063018760	-------------PD-DRDT S -EE---R-D--L----------------
	Nocardia nova	WP_063013590	-------------PD--SDT S -AE---R----------R----------
	Nocardia otitidiscaviarum	WP_039814499	------------RPD--RDT T -AE---R-A--L----------------
	Nocardia paucivorans	WP_040790932	-------------PD-DRDT T -EE---R-DN-------R----------
	Nocardia pneumoniae	WP_040779440	-------------PD-DRDT S -EE---R-D-------------------
	Nocardia pseudobrasiliensis	WP_062512121	-------------PD-RADT S -AE---R---------------------
	Nocardia pseudovaccinii	WP_063042263	-------------PD--NDA S -AE---R--N------------------
	Rhodococcus coprophilus	WP_072698168	-------------PD-VVDR S -A---A-----L-----R----------
	Rhodococcus corynebacterioides	WP_072681530	-------------PD-AVDR T V----AD--H-V-V--------------
	Rhodococcus defluvii	WP_031936972	--------------D-ATD- S -A----R------V--------------
	Rhodococcus enclensis	SCC24409	------------SPD-STDT S -A----R--N---V--------------
	Segniliparus rotundus	WP_013138013	---------I--EPDRQTDR S -QE--GA--K----E-------------
	Segniliparus rugosus	WP_007468002	---------I--EPDRQTDR S -S---GA-------E-------------
	Smaragdicoccus niigatensis	WP_018162172	-------------PD--RDT T -AE---R----L-----R----------

Supplementary Figure 10

A partial sequence alignment of a conserved region of peptidase C69 showing a one amino acid deletion that is specific for members of the genus *Mycobacterium* and absent in most other *Corynebacteriales*.

Genus *Mycobacterium* (>100/>100)

		95		143
Mycobacterium tuberculosis	WP_031743956	PTALRELIRYVRPSWLRRWVRDGYAWVQPRLSP		VARAALPPHLTAEYL
Mycobacterium abscessus	WP_074330105	------S--LI--AK--S------S-L------		I--V----K--V---
Mycobacterium acapulcensis	WP_066808347	-------------PL---------C--------		----------S----
Mycobacterium alsense	WP_083136726	-------------P-----A------L------		---S------S----
Mycobacterium angelicum	WP_083114302	-------------P----------G--------		I--P----R------
Mycobacterium aromaticivorans	WP_036344414	------------------------G----K---		M--P-------V---
Mycobacterium arosiense	WP_083062921	-------------P----------G-L------		---S------S-D--
Mycobacterium arupense	WP_046187600	------------------------G-L---F--		M----------VS--
Mycobacterium asiaticum	WP_065036781	-------------P----------G-L------		I--P-----------
Mycobacterium aurum	WP_048633211	-------------PRV--------G-----F--		I----------V---
Mycobacterium avium	WP_062890843	-------------PR---------G-L------		---S------SVD--
Mycobacterium avium subsp. avium	EUA41267	-------------PR---------G-L------		---S------SVD--
Mycobacterium avium subsp. hominissuis	KDP00718	-------------PR---------G-L------		---S------SVD--
Mycobacterium bacteremicum	WP_083055948	----------I--PV----A----G-L-----A		---P------SV---
Mycobacterium boenickei	WP_077741352	-------------A-----A----G--------		I--S-------V---
Mycobacterium bohemicum	WP_085182982	---V---------P-----A----G-L------		---S------SV---
Mycobacterium branderi	WP_083131559	-------------P----------G-L------		F---------SV---
Mycobacterium brisbanense	WP_062827232	-------------A-----A----G-L------		I--S-----V-V---
Mycobacterium canariasense	WP_062657454	------------------------G--------		---P------SV---
Mycobacterium canettii	WP_015290658	---------------------------------		---------------
Mycobacterium celatum	WP_062540381	-------------P----------G-L------		F--P------S----
Mycobacterium celeriflavum	WP_083001279	---A---------P----------C--------		----------S----
Mycobacterium chelonae	WP_070942613	------S--LI--PR--S------G-L------		I--V----K--V---
Mycobacterium chimaera	WP_089151509	-------------P----------G-L------		---P------S-D--
Mycobacterium chlorophenolicum	WP_048468330	-------------P-V----------A------		I----------V---
Mycobacterium chubuense	WP_014816627	-------------P-V--------G--------		-----------V---
Mycobacterium colombiense	WP_064878339	-------------P-----A----G-L------		---S------S-D--
Mycobacterium conceptionense	WP_076214269	-------------A-----A----G-I------		I--S-------V---
Mycobacterium confluentis	WP_085153211	---A--A------AT---------G-L------		-S-S-----------
Mycobacterium conspicuum	WP_085235066	-------------P-----A----G-L------		I--P-----------
Mycobacterium diernhoferi	WP_073853608	-------------PF---------G-L-----R		---P-------V---
Mycobacterium doricum	WP_085189831	-------------P----------G-I------		----------S----
Mycobacterium engbaekii	WP_085129765	--------------G-------F-G-L---F--		F----------VS--
Mycobacterium europaeum	WP_085240259	-------------PR---------G-L------		---S---A--SVD--
Mycobacterium fallax	WP_085100409	---V---------P----------G-A---F--		L--P-----------
Mycobacterium flavescens	WP_069412955	-------------PF---------G-L------		-S-------------
Mycobacterium florentinum	WP_085220699	-------------P----------G-L------		------------D--
Mycobacterium fortuitum	WP_064900394	-------------A----------G--------		I--------V-V---
Mycobacterium fragae	WP_085199570	-------------P----------G-L------		I--S----Q-S----
Mycobacterium franklinii	WP_070935513	------S--LI--PK--S------G-L------		I--V----K--V---
Mycobacterium gastri	WP_036409473	-------------P-----A----------F--		---S-------V---
Mycobacterium genavense	WP_025736085	-------------P----------G-L------		---P-----------
Mycobacterium gilvum	WP_011893536	-------------PR----A----G-L------		I--P-------V---
Mycobacterium goodii	WP_049745829	-------------PR-------A-G--------		I--------V-V---
Mycobacterium gordonae	WP_065043294	---M---------P-------E--G-L------		---S----Q---Y--
Mycobacterium haemophilum	WP_054879697	-------------P-------G--G--------		--K-----S--VK--
Mycobacterium hassiacum	WP_026213345	----------I--P----R---L-G-L------		-S-N-----------
Mycobacterium heckeshornense	WP_048892737	-------------P----------G-L-R----		---P----S--V---
Mycobacterium heidelbergense	WP_083072047	-------------P-----A------L------		---P------S----
Mycobacterium heraklionense	WP_064998375	-------------A----------G-L------		---S-------V---
Mycobacterium hiberniae	WP_085134760	--------------G-------F-G-L---F--		F----------VS--
Mycobacterium holsaticum	WP_069407694	-------------P----------G-L------		-S-P---GR-S----
Mycobacterium houstonense	WP_066898289	-------------PR---------G-L------		I--S-----V-V---
Mycobacterium icosiumassiliensis	WP_067974551	---V---------A----------G-L------		-----------V---
Mycobacterium immunogenum	WP_043075933	------S--LI--AT--G------G-L------		I--V----K--V---
Mycobacterium insubricum	WP_083028810	---A---------PR---------G-L------		L--P---AK---D--
Mycobacterium interjectum	WP_085200647	---V---------P-----A----G-L------		---P-----------
Mycobacterium intermedium	WP_069417409	-------------PR---------G-L------		Y--P-----------
Mycobacterium intracellulare	WP_064943309	-------------P----------G-L------		---P------S-D--
Mycobacterium iranicum	WP_064283380	-------------PRV---A----G-L------		I--P-------V---
Mycobacterium kansasii	WP_063471287	------F------P-----A----------F--		---S-------V---
Mycobacterium komanii	CRL67155	------------------------G-I------		----------S----
Mycobacterium kubicae	WP_085073361	-------------P-------E--G--------		---S------SV---
Mycobacterium kumamotonense	WP_019735179	-------------PR----I----G-L------		L-----------D--

Group	Species	Accession	Alignment		
**Genus *Mycobacterium* (>100/>100)**	*Mycobacterium kyorinense*	WP_065015577	-------------P----------G-L------		M---------SV---
	Mycobacterium lacus	WP_085162923	-------------P-------------------		---S------S----
	Mycobacterium lentiflavum	CQD17361	-------------P----------G--------		---P--------D--
	Mycobacterium leprae	WP_041322801	-------------P-----I-G--G--------		--K-----Y---Q--
	Mycobacterium lepromatosis	WP_045843164	-------------P-----I-G--G--------		--KP----Y---K--
	Mycobacterium litorale	WP_078018709	------S---I--P----VI----G-I------		---P----K-S-D--
	Mycobacterium llatzerense	WP_043985252	-------------P----I-----G--------		I--P---A-------
	Mycobacterium longobardum	WP_085263534	-------------A----------G-L---F--		-----------VD--
	Mycobacterium mageritense	WP_036442660	-------------A----------G--------		I--S----RV-V---
	Mycobacterium malmesburyense	CRL79056	-------------P-----A----G-I------		----------S----
	Mycobacterium malmoense	WP_065442951	-------------PR---------G-L------		---P------SVD--
	Mycobacterium mantenii	WP_083097007	-------------P----------G-L------		---S------S-D--
	Mycobacterium minnesotense	WP_083022294	------------------------G-L---F--		M----------VS--
	Mycobacterium moriokaense	WP_083150127	----------I--A----------G-L------		-S-S-----V-V---
	Mycobacterium mucogenicum	WP_064860899	-------------P----I-----G-L------		---P---A---V---
	Mycobacterium nebraskense	WP_046182123	---V---------PR---------G-L------		---S------S-D--
	Mycobacterium neoaurum	WP_030133673	-------------PL----A----G-L------		---P------SV---
	Mycobacterium neworleansense	CRZ13539	----------I--AR----A----G--------		I--S-------V---
	Mycobacterium nonchromogenicum	WP_085139632	-------------A----------G-L------		---S-------V---
	Mycobacterium noviomagense	WP_083084401	---V---------P----------G-L------		L--P-------V---
	Mycobacterium novocastrense	WP_067386811	-------------P----------G--------		---P------S----
	Mycobacterium obuense	WP_046366202	----------A--PQ---------S-L------		I--S-------V---
	Mycobacterium palustre	WP_085078226	-------------P----------G-L------		---P----R-S----
	Mycobacterium paraense	WP_085094460	---M---------P-----A----G-L------		---S-----------
	Mycobacterium paraffinicum	WP_073880125	-------------PR---------G-L------		---S------SV---
	Mycobacterium parafortuitum	WP_083145152	-------------PR----A----G-L------		I--P-------V---
	Mycobacterium paraseoulense	WP_083168791	-------------PR---------G-L------		---S------S----
	Mycobacterium peregrinum	WP_064882480	-------------A-----A----G-I------		I--------V-V---
	Mycobacterium phlei	WP_003890466	-------------PT-------A-G-L------		---T-----------
	Mycobacterium porcinum	WP_069424831	-------------A-----A----G--------		I--S-------V---
	Mycobacterium rhodesiae	WP_083121345	------------------------G--------		M--P-------V---
	Mycobacterium riyadhense	WP_085250730	-------------P-------------------		M--S------S----
	Mycobacterium rufum	KGI68987	-------------PRV----------A---F--		I----------V---
	Mycobacterium rutilum	WP_083408188	-------------PF---------G--------		-S-------------
	Mycobacterium salmoniphilum	WP_078325461	------S--LI--PR--S-I----G-L------		I--V----K--V---
	Mycobacterium saopaulense	WP_070911170	------S--LI--AK--S------G-L------		I--N----K--V---
	Mycobacterium saskatchewanense	WP_085257983	-------------P----------G-L------		---S------S----
	Mycobacterium scrofulaceum	WP_067283034	-------------PR---------G-L---F--		---P------SV---
	Mycobacterium senuense	WP_085083548	------F------PR---------G-L------		R-------Q---D--
	Mycobacterium septicum	WP_044522912	------S---I--A-----A----G-L------		IS-------V-V---
	Mycobacterium setense	WP_064874839	-------------A-----T----G-L--K---		I--S----G--V---
	Mycobacterium sherrisii	WP_069399039	-------------PR---------G-L------		----------S----
	Mycobacterium shigaense	BAX93554	-------------P----------G-L---F--		L--P-----------
	Mycobacterium shimoidei	WP_069394590	-------------P----------G-L---F--		I--S------S-K--
	Mycobacterium simiae	WP_061556328	-------------P----------G-L-----A		---P------S----
	Mycobacterium sinense	WP_064856652	---V---------P----------G-L------		L-------R---D--
	Mycobacterium smegmatis	WP_011729938	------M------P----------G--------		I--S-----V-V---
	Mycobacterium szulgai	WP_085669676	-------------P----------G--------		I--P----R------
	Mycobacterium terrae	WP_085260287	-------------PR---------G-L------		L-------N-S-D--
	Mycobacterium thermoresistibile	WP_003926898	-------------PR---------G-L------		Y--S-----VSVQ--
	Mycobacterium triplex	WP_036472761	-------------P----------G--------		---------------
	Mycobacterium triviale	WP_085108877	----------I--PR----T----G-L------		---S----Q--VD--
	Mycobacterium tusciae	WP_006241162	-------------A-----A----G-L------		-S-------VSV---
	Mycobacterium ulcerans	WP_011741423	-------------P-------------------		---S---AD-S----
	Mycobacterium vaccae	WP_003928447	-------------PR---------G--------		---P-------V---
	Mycobacterium vulneris	WP_065461858	-------------A-----A----G--------		I--S-------V---
	Mycobacterium wolinskyi	WP_067847117	-------------A----------G-A------		I--S-----V-V---
	Mycobacterium xenopi	WP_085193525	-------------P-V--------G-L-R----		---P-------V---
	Mycobacterium yongonense	WP_065500170	-------------P----------G-L------		---P------S-D--
**Other *Corynebacteriales***	*Hoyosella altamirensis*	WP_064439312	------H------PA---V--GA-RH--SA---	V	GWPM----R--VS--
	Hoyosella subflava	WP_013805942	------H---I--PV---V--GA-RH--SA---	V	GWPM----R--VC--
	Gordonia malaquae	WP_052005467	------Q---L--AR--QV-----S-L------	L	GWPM----RV-V--F
	Gordonia namibiensis	WP_006865743	------Q------NR--QL---A-S-----G-R	L	GWPL----KV-V---
	Gordonia neofelifaecis	WP_009677575	------Q------AK--QA--GA-G-L------	V	GWPM-I--AE--R-F
	Gordonia otitidis	WP_007238992	------Q------GR--QL--SA-Q-----A-R	I	GWPV----AV-V---

Other *Corynebacteriales*

```
Gordonia paraffinivorans            WP_006899821   ------Q-------R--QL--AA-S-----G-R L GWPL----KV-V---
Gordonia polyisoprenivorans         WP_006370609   ------Q---L--PR--QA--T--Q-W---A-R L GWPV----KV-V-H-
Gordonia rhizosphera                WP_051998860   ------Q---L--NR--QV-----Q-L------ L GWPT----RV-V---
Gordonia soli                       WP_040508891   ------Q------AR--QV--SA-E-L------ V GWPM----AV-V---
Gordonia terrae                     WP_051129758   ---F--Q---I--NG--QKI-EA-------GAR L GWPL----RI-I---
Millisia brevis                     WP_066904514   ------GL--L--DP---VA-S--Q-L------ L GRPV----KISVQ--
Nocardia abscessus                  WP_043688838   -------------PA---V--AT-N-L--K--K L GRPV----KVSVD--
Nocardia acidivorans                WP_067568809   --------------FA--Q--TA-N-L--K--- L GWPV----AVSVD--
Nocardia altamirensis               WP_069166728   -------------PV---G--AT-N-L--K--K L GRPV----KVSVD--
Nocardia amamiensis                 WP_067479774   -------------PA---A--AT-N-L--K--K L GRPV----KVSVD--
Nocardia amikacinitolerans          WP_067787481   -------------PG---R--SV-N-L--K--K L GRPV----KVSVD--
Nocardia anaemiae                   WP_062993415   -------------PA---G--GT-N-L--K--K L GRPV----KVSVD--
Nocardia aobensis                   WP_051024443   ------M---L--PV---Q--T--Q-L-----K L GRPV----RVSID--
Rhodococcus aetherivorans           WP_029544384   ------TL-----PR---V--ET-G-L------ L GWPV----RVSVD--
Rhodococcus coprophilus             WP_072699644   ------GL-----PA---VA-NA-G-L------ L GWPV----RVSV---
Rhodococcus corynebacterioides      WP_068146993   ------Q------PA---A--A--G-A-----R L GRPV----A-SVD--
Rhodococcus defluvii                WP_031938305   ------QL--I--PA---V--S--Q-L-----K L GRPV----R-SV---
Rhodococcus enclensis               WP_058227182   ------T------PA---V--E--Q-L----A- L GRPV----K-SV---
Rhodococcus equi                    WP_064078765   ------QL--I--PA---V--T--Q-L-----K L GRPV----R-SV---
Rhodococcus triatomae               WP_072737610   ------Q---L--PQV--V--A--Q-L------ L GRPV----R-SV---
Rhodococcus tukisamuensis           WP_072844974   ------Q------PA---V--S--Q-L----A- L GWPV----K-SV---
Rhodococcus wratislaviensis         WP_037226366   ------QL-----PA---V--A--Q-L------ L GWPV----R-SV---
Rhodococcus yunnanensis             WP_072802475   ------Q------PA---V--A--Q------AH L GWPV----K-SVD--
Rhodococcus zopfii                  WP_072812676   ------QL-----AS---VA-TA-G-L------ L GWPV---ARVSV---
Segniliparus rotundus               WP_013137938   ------SM--I--PR---R--EA-Q-A--K-A- I GWPI---AG--VH-Q
Segniliparus rugosus                WP_021030688   ---V--T---I--PQ---R--EA-Q-A----A- L GWPI---AR--V--Q
Skermania piniformis                WP_066469070   ------Q---L--PSV--A--SA-G-------R V GWPV----R-S-A--
Smaragdicoccus niigatensis          WP_051090822   ------Q------AA---R---A-G-L------ L GWPV-----VSVD--
Tomitella biformata                 WP_024793296   ------Q------ESA--A---A-S-L----A- L GWPL------S----
Tsukamurella paurometabola          WP_013127417   ------Q---L--AS--ARA-----V--RLFA- V GWPM----EV-VD--
Tsukamurella pulmonis               WP_068529834   ---I--Q---L--AS--ARA-----VL-KTFA- V GWPM-----V-VD--
Tsukamurella tyrosinosolvens        WP_068521384   ---I--Q---L--AS--ARA-----TI-RVF-- V GWPM----KV-VD--
Williamsia herbipolensis            WP_045823205   ---V--Q---L------QA--AA-GRL--L--- L GWPV---APV-V---
Williamsia muralis                  WP_062799763   ------Q---I--PA--QQ--NA-G-L------ L GWPL----A--TD--
Williamsia sterculiae               WP_076480027   ------D---I--AR--Q---AA-GRL--L--- V GWPI---ASA-VDH-
```

Supplementary Figure 11

A partial sequence alignment of a conserved region of SGNH/GDSL hydrolase family protein showing a one amino acid deletion that is specific for members of the genus *Mycobacterium* and absent in other *Corynebacteriales* including the two *Hoyosella* species.

Genus *Mycobacterium* (>100/>100)

		27		56
Mycobacterium abscessus	WP_062880084	DRPAALDNPRAPRRGSGM	P	NFEKYAWIFMRLS
Mycobacterium abscessus subsp. bolletii	EHM16914	------------------	-	-------------
Mycobacterium angelicum	WP_083116202	----S-----S---RA-I	-	----F--L---F-
Mycobacterium aromaticivorans	WP_036340468	----G-----T---R---	-	-------L---F-
Mycobacterium arosiense	WP_083066382	----S-----S---RA-I	-	----F--L---F-
Mycobacterium asiaticum	WP_065033737	----S-----S---RA-I	-	----F--L---F-
Mycobacterium aurum	WP_087019921	----S--H------PR-I	-	Y------L---F-
Mycobacterium avium	WP_084247083	----S-----S---RA-I	-	----F--L---F-
Mycobacterium bacteremicum	WP_083058790	----S--H-----KPR-I	-	Y------L---F-
Mycobacterium bohemicum	WP_085182135	----G-----S---R--I	-	----F--L---F-
Mycobacterium bovis	WP_024456379	----S-----S---RA--	-	----F--L---F-
Mycobacterium branderi	WP_083130002	----G---------PG--	-	-------L---F-
Mycobacterium brisbanense	WP_062830213	-------H------PR-I	-	Y------L---F-
Mycobacterium canariasense	WP_062659799	----G---------PG--	-	-------L---F-
Mycobacterium canettii	WP_014001681	----S-----S---RA--	-	----F--L---F-
Mycobacterium celatum	WP_062541225	----G---------R---	-	-------L---F-
Mycobacterium chelonae	WP_046254563	--------------A---	-	-------------
Mycobacterium chlorophenolicum	WP_048471552	----S---------AG--	-	-------L---F-
Mycobacterium chubuense	WP_041781761	----S---------AG--	-	-------L---F-
Mycobacterium colombiense	WP_064878684	----S-----S---RA-I	-	----F--L---F-
Mycobacterium confluentis	WP_085149144	----G---------RA--	-	-------L---F-
Mycobacterium conspicuum	WP_085231539	----S-----S---RA-I	-	----F--L---F-
Mycobacterium diernhoferi	WP_073856577	----S--H------PR-I	-	Y------L---F-
Mycobacterium doricum	WP_085187108	---PS-------K-AT--	-	-----T-L---F-
Mycobacterium engbaekii	WP_085127920	----S-GD------H---	-	-----T-L---F-
Mycobacterium europaeum	WP_085241731	---PS-----S---RA-I	-	----F--L---F-
Mycobacterium fallax	WP_085095314	-------H------PR-V	-	Y------L---F-
Mycobacterium flavescens	WP_069414731	----G--H------PR-I	-	Y------L---F-
Mycobacterium florentinum	WP_085219952	---PS-----S---RA-I	-	----F--L---F-
Mycobacterium fortuitum subsp. fortuitum	EJZ13648	-------H------PR-I	-	Y------L---F-
Mycobacterium fragae	WP_085198543	----G---------RA--	-	-------L---F-
Mycobacterium franklinii	WP_070938629	--------------A---	-	-------------
Mycobacterium gastri	WP_036410069	----S-----S---RA--	-	----F--L---F-
Mycobacterium genavense	WP_025738485	----S-----S---R--I	-	----F--L---F-
Mycobacterium gilvum	WP_085978075	----G---------SG--	-	-----T-L---F-
Mycobacterium goodii	WP_049747998	-------H------PR-I	-	Y------L---F-
Mycobacterium gordonae	WP_06504629	---S-----S---RV-I	-	----F--L---F-
Mycobacterium haemophilum	WP_054880591	----S-----S---RA-I	-	----F--L---F-
Mycobacterium hassiacum	WP_018354366	----G--H------PR-I	-	Y------L---F-
Mycobacterium heidelbergense	WP_083076346	----S-----S---RA-I	-	----F--L---F-
Mycobacterium heraklionense	WP_065041863	----S-GD----Q-H---	-	-----T-L---F-
Mycobacterium hiberniae	WP_085135114	-H--S-GD-----QH---	-	-----T-L---F-
Mycobacterium holsaticum	WP_069407318	----S--H------AR-I	-	Y------L---F-
Mycobacterium icosiumassiliensis	WP_067970457	----S-GD----Q-H---	-	-----T-L---F-
Mycobacterium immunogenum	WP_043076573	------------------	-	-------------
Mycobacterium insubricum	WP_083031093	----S--H-----KPR-I	-	Y------L---F-
Mycobacterium interjectum	WP_066907970	----S-----S---RA-I	-	----F--L---F-
Mycobacterium intermedium	WP_069420420	----S-----S---RG-I	-	----F--L---F-
Mycobacterium iranicum	WP_064283322	---PG---------AG--	-	-------L---F-
Mycobacterium kansasii	WP_063472134	----S-----S---RA--	-	----F--L---F-
Mycobacterium komanii	CRL75541	----S--H------PR-I	-	Y------L---F-
Mycobacterium kyorinense	WP_065014993	----G---------RA--	-	-----T-L---F-
Mycobacterium lacus	WP_085162478	----S-----S---RA--	-	----F--L---F-
Mycobacterium lentiflavum	CQD07608	---PS-----S---R--I	-	----F--L---F-
Mycobacterium leprae	WP_010907881	----S-----S---RA-I	-	----F--L---F-
Mycobacterium lepromatosis	WP_045842652	----S-----S---RA-I	-	----F--L---F-
Mycobacterium litorale	WP_078020539	----G-----T--QRA--	-	-------L---F-
Mycobacterium llatzerense	KIU15599	-------H------PR-I	-	Y------L---F-
Mycobacterium longobardum	WP_085266889	----S-GD------SN--	-	-----T-L---F-
Mycobacterium malmesburyense	CRL78521	----G--H------PR-I	-	Y------L---F-
Mycobacterium malmoense	WP_071513303	----S-----S---RA-I	-	----F--L---F-
Mycobacterium minnesotense	WP_083023655	----S-GD--S-Q-H---	A	-----T-L---F-
Mycobacterium moriokaense	WP_083153867	----S--H------PR-I	-	Y------L---F-
Mycobacterium nebraskense	WP_046186125	----S-----S---RA-I	-	----F--L---F-

	Mycobacterium noviomagense	WP_083087073	----G---------R---	-	-------L---F-
	Mycobacterium novocastrense	GAT12424	----G--H------PR-I	-	Y------L---F-
	Mycobacterium palustre	WP_085080276	----S-----S---RA-I	-	----F--L---F-
	Mycobacterium paraense	WP_085096199	----S-----S---RA-I	-	----F--L---F-
	Mycobacterium paraffinicum	WP_073878083	----S-----S---RA-I	-	----F--L---F-
	Mycobacterium parafortuitum	WP_083145770	----G---------AG--	-	-------L---F-
	Mycobacterium parmense	WP_085270611	----S-----S---RA-I	-	----F--L---F-
	Mycobacterium phlei	WP_003886459	----G--H------PR-I	-	Y------L---F-
	Mycobacterium rhodesiae	WP_083121570	----G-----T---R---	-	-------L---F-
	Mycobacterium riyadhense	WP_085251055	----S-----S---RA-I	-	----F--L---F-
	Mycobacterium rufum	KGI67212	----S---------AG--	-	-------L---F-
	Mycobacterium rutilum	WP_083410040	----G--H------PR-I	-	Y------L---F-
	Mycobacterium salmoniphilum	WP_078323680	------------------	-	-------------
	Mycobacterium saopaulense	WP_083335707	------------------	-	-------------
	Mycobacterium saskatchewanense	WP_085253649	----S-----S---RA-I	-	----F--L---F-
	Mycobacterium scrofulaceum	WP_083179255	----S-----S---RA-I	-	----F--L---F-
	Mycobacterium senuense	WP_085087696	----S-GD----F-HK--	A	-----T-L---F-
	Mycobacterium sherrisii	WP_069399048	---PS-----S---RA-I	-	----F--L---F-
**Genus *Mycobacterium* (>100/>100)**	*Mycobacterium shigaense*	BAX91270	----S-----S---RA-I	-	----F--L---F-
	Mycobacterium shimoidei	WP_069395276	----G---------RT--	-	-------L---F-
	Mycobacterium shinjukuense	WP_083048709	----S-----S---RA-I	-	----F--L---F-
	Mycobacterium simiae	WP_061558461	---PS-----S---RA-I	-	----F--L---F-
	Mycobacterium sinense	WP_064856703	----S-GD--S---HA--	-	-----T-L---F-
	Mycobacterium smegmatis	WP_003893078	-------H-----KPR-I	-	Y------L---F-
	Mycobacterium szulgai	WP_085671177	----S-----S---RA-I	-	----F--L---F-
	Mycobacterium terrae	WP_085259873	----S-GD--S---SN--	-	-----T-L---F-
	Mycobacterium thermoresistibile	WP_050812142	--------------RG--	-	-------L---F-
	Mycobacterium triplex	WP_036467114	----S-----S---R--I	-	----F--L---F-
	Mycobacterium triviale	WP_085109744	-S--SFG---S---HTA-	-	-----T-----F-
	Mycobacterium tuberculosis	WP_070896326	----S-----S---RA--	-	----F--L---F-
	Mycobacterium tusciae	WP_083124935	----S--H------PR-I	-	Y------L---F-
	Mycobacterium ulcerans	WP_071498036	----S-----S---RAS-	-	-------L---F-
	Mycobacterium vaccae	WP_003929450	----G-----S---SG--	-	-----T-L---F-
	Mycobacterium vanbaalenii PYR-1	ABM12417	----G-----S---AG--	-	-------L---F-
	Mycobacterium vulneris	WP_065461470	-------H------PR-I	-	Y------L---F-
	Mycobacterium wolinskyi	WP_085146845	-------H-----KPR-I	-	Y------L---F-
	Mycobacterium xenopi	WP_085197211	----G--KL-----R---	-	-------L---F-
	Multispecies:*Hoyosella*	WP_041451212	----S-SL--T---R--N		---MWS-L---I-
	Dietzia alimentaria	WP_010542052	----S-AQ--S--LR-RG		---RN--M---A-
	Dietzia cinnamea	WP_063974176	----S-AQ--S--LR-RF		---RT------Y-
	Dietzia timorensis	WP_067477923	----S-AQ--S--LRN-G		---RN--L---I-
	Gordonia aichiensis	WP_005174037	----S-----S--KPR-G		----W--L---F-
	Gordonia amarae	WP_005189744	---S-----S--KPK-G		----N--M---F-
	Gordonia amicalis	WP_024497213	----S-----S--KPK-G		----N--M---F-
	Gordonia araii	WP_007320786	---PS-----S--KPR-G		-------L---F-
	Gordonia bronchialis	WP_012833598	----S-----S--KSK-G		----N--M---F-
	Gordonia desulfuricans	WP_059036086	----S--------ARK-G		-------L---F-
	Gordonia effusa	WP_007317948	----S-----S--KPK-G		----N--L---F-
	Gordonia hirsuta	WP_005937375	----S--A-----AV-RS		----W--L---F-
	Gordonia hydrophobica	WP_066165915	----S--A-N---PRNRG		----N--L---F-
**Other *Corynebacteriales***	*Gordonia kroppenstedtii*	WP_018180765	----S---------PR-G		----N--L---F-
	Millisia brevis	WP_066905422	----G---------AG-G		---L-S-L-----
	Nocardia abscessus	WP_043687868	----S--L--S--AR-SN		-------L---F-
	Nocardia acidivorans	WP_067562030	----S--A------QG-N		-------L---F-
	Nocardia africana	WP_062961651	----S--S------S--N		-------L---F-
	Nocardia alba	WP_067447909	----S--S--S--GK-NG		-------L---I-
	Nocardia altamirensis	WP_069166253	----S--S--S--AR-NN		-------L---F-
	Nocardia amamiensis	WP_067476922	----S--L--S--AR-SN		-------L---F-
	Nocardia amikacinitolerans	WP_067787814	----S--L--S--AR-SN		-------L---F-
	Nocardia anaemiae	WP_062982838	----S--L--S--AR-NN		-------L---F-
	Nocardia araoensis	WP_039796191	----S--L--S--AR-NN		-------L---F-
	Nocardia arizonensis	WP_054815690	----S--L--S--AR--G		-------L---F-
	Nocardia arthritidis	WP_063051569	----S--L--S--AR-NN		-------L---F-
	Nocardia asiatica	WP_043720381	----S--L--S--AR-NN		-------L---F-
	Rhodococcus coprophilus	WP_072699515	----S-----S---V-NN		---L---L-----

Group	Species	Accession	Sequence 1	Sequence 2
**Other *Corynebacteriales***	*Rhodococcus defluvii*	WP_031938616	----S-----S--KAAKN	---L---L---F-
	Rhodococcus kroppenstedtii	WP_068363589	----G-----S---KNRG	---L---L---F-
	Rhodococcus kunmingensis	WP_068275047	----S-----S---KAKG	---L---L---F-
	Rhodococcus kyotonensis	WP_068428273	----S-----S---S-RG	---L---L---F-
	Rhodococcus marinonascens	WP_072687252	----S-----S---A-KS	---L---L---F-
	Rhodococcus phenolicus	WP_068154277	----G-----S---QAKN	---L---L-----
	Rhodococcus rhodnii	WP_010839279	----S---------QAKN	---L---L---F-
	Rhodococcus rhodochrous	WP_016692832	----G-----T---AAKN	---L---L-----
	Rhodococcus triatomae	WP_007538220	----S--S--S---Q-KN	---L---L-----
	Rhodococcus tukisamuensis	WP_072843697	----S--S--S---E-KN	---L---L-----
	Rhodococcus wratislaviensis	WP_037227605	--QAST-G	----F--------
	Rhodococcus yunnanensis	WP_072807208	----S-----S---K-KG	---L---L---F-
	Rhodococcus zopfii	WP_072814244	----G-----S---QAKN	---L---L-----
	Segniliparus rugosus	WP_021030245	-PTVPA-A-KK-SGWR	H--RH--F---I-
	Skermania piniformis	WP_066472511	----S-----S--QL-KN	---L---L---F-
	Smaragdicoccus niigatensis	WP_018163408	----S--A---A--VNKS	---L---------
	Tomitella biformata	WP_024793456	----S-----S---PRRG	---MM--V-----
	Tsukamurella paurometabola	WP_013125639	----S-----S-H-PT-G	-------L---F-
	Tsukamurella pseudospumae	WP_068570800	----S--A--S-H-PA-N	----W--L---F-
	Tsukamurella pulmonis	WP_068528819	----S-----S-H-PAAG	-------L---F-
	Tsukamurella tyrosinosolvens	WP_068522412	----S-----S-H-PASG	-------L---F-
	Williamsia sterculiae	WP_076477042	----S---------A-TS	----N--L---F-

Supplementary Figure 12

A partial sequence alignment of a conserved region of succinate dehydrogenase showing a one amino acid insertion that is specific for members of the genus *Mycobacterium* and absent in other *Corynebacteriales* including the *Hoyosella* species.

Genus *Mycobacterium* (90/90)

		109	153
Mycobacterium tuberculosis	SGA93253	GESRAYASWMSSVGYRPVT	TRHVNEGQGDLLMVGERVLAGYGFRT
Mycobacterium abscessus	WP_074249060	---D-H-A--RQH-L--MH	S-YT------F-LA-DIM---T----
Mycobacterium abscessus subsp. bolletii	SHT62009	---D-H-A--RQH-L--MH	S-YT------F-LA-DIM---T----
Mycobacterium africanum	WP_003910540	-------------------	--------------------------
Mycobacterium aromaticivorans	WP_051660121	---V---A--GQH-HD--A	------------V--SMI---T----
Mycobacterium asiaticum	WP_065139599	D--KV-SK----L-----S	--Y---------QI-DM---------
Mycobacterium aurum	WP_087031507	A-AD---A--TAA-F--ER	---R--------VA-ST---------
Mycobacterium austroafricanum	WP_036370151	---V---G--DGR-H---S	------------V--SMI---T----
Mycobacterium avium	WP_062888090	-------E--R-L----LL	------------K--DI----W----
Mycobacterium avium subsp. paratuberculosis	ETB40501	-------E--R-L-----L	------------K--DI----W----
Mycobacterium bacteremicum	WP_083055529	P-AD---A--TRA-LQ-HR	---H--------VA-PM---------
Mycobacterium boenickei	WP_077742230	--AD---E---AA-----R	-E-I--------L--SNL--------
Mycobacterium bohemicum	WP_085182904	---Q---E----L-H----	-------------I--T----H----
Mycobacterium bovis	WP_080712386	-------------------	--------------------------
Mycobacterium brisbanense	WP_084388370	P-AD---D--AAR-FTLAR	------------V--STL---W----
Mycobacterium canariasense	WP_062658088	D-AA---A--TDR-HH--Q	------------V--SKI--------
Mycobacterium canettii	WP_015290585	-------------------	--------------------------
Mycobacterium chelonae	WP_070915821	---A-H-E--RVH-L-AMH	--YT------F-LA-DVM---T----
Mycobacterium chlorophenolicum	WP_082168991	A-AV---A--ADH-FAATE	------------LA-SMI---H----
Mycobacterium chubuense	WP_014814223	---V---A---RN-FHLAD	---A--------LL-ATI---H----
Mycobacterium colombiense	WP_064878136	-------R----L----LC	------------K--DI----W----
Mycobacterium conceptionense	WP_076215813	A-AD---E--TAA-----R	-E----------L--SKL--------
Mycobacterium confluentis	WP_085149685	D-A----D--TAR--P-I-	-Q-T--------V--ATI---T----
Mycobacterium conspicuum	WP_085231322	-------A--A-R------	------------LA-QT---------
Mycobacterium diernhoferi	WP_073856285	A-AD---A--TAA-WH-ER	---H--------VA-ST----H----
Mycobacterium europaeum	WP_085241942	-------Q----L-----S	----------------T---------
Mycobacterium fallax	WP_085096358	D-AL-HRA--TAA-H--IP	-TG---------T--SMI---T----
Mycobacterium farcinogenes	CDP85104	A-AD---E--TAA-----R	-E----------L--SKL--------
Mycobacterium flavescens	WP_069413145	A-AH---A--TAR-L---E	------------V--AT---------
Mycobacterium florentinum	WP_085221316	--AA--GE----M-FQ--S	--FI-------------M--------
Mycobacterium fortuitum	OBB02222	--AD---E---AT---T-F	-D----------LA-STL--------
Mycobacterium fragae	WP_085197290	R--I---H--RRR--T--S	------------P---MI---T----
Mycobacterium franklinii	OHU22852	---D-H-E--RQHELH-MH	SQYT------F-L--DIM---T----
Mycobacterium gilvum	WP_011895894	R-AV---D--TRN--DAFE	------------T--GV----F----
Mycobacterium gordonae	WP_065043126	---K---R----------S	------------LI-DM---------
Mycobacterium haemophilum	WP_047315121	---K---E-----------	----------V-L---ML--------
Mycobacterium heidelbergense	WP_083077014	-------E----L-----S	----------V-----TA--------
Mycobacterium houstonense	WP_066901477	A-AD---E--D-R---T-R	-E-L--------L--RTL--------
Mycobacterium immunogenum	OAT71092	---D-H-A--RQH-LQ-RH	SHYT------F-LA-DVM---T----
Mycobacterium interjectum	WP_085205268	--AA---E----L-----S	---------------DT---------
Mycobacterium intermedium	WP_069420760	---K--SR----A-----S	------------LI--T---------
Mycobacterium iranicum	WP_064283906	--AV---Q--ARN--E-FE	----------F-A--TVI---F----
Mycobacterium komanii	CRL75103	A-AA---E--AQH--Q--Q	A--T--------IA-SIL--------
Mycobacterium lacus	WP_085161528	---K---G----------H	-------------I--K----F----
Mycobacterium lentiflavum	CQD17017	A-AV--GE----M--Q---	---I--------L---M----F----
Mycobacterium litorale	AQT78778	---A---A--GLR-HQ--A	-H-I--------V--AMI---T----
Mycobacterium llatzerense	KIU18503	---V---G-LLDH--C-LY	-E-T--------I--SKI--------
Mycobacterium mageritense	CDO20513	--AE---D--AAQ-FTT--	-E----------L--RTL---W----
Mycobacterium malmesburyense	CRL67984	--AAV--E-LARH--Q--Q	--YT--------VA-SIL--------
Mycobacterium malmoense	WP_065442610	-------D----L----LS	--Y---------L---T---------
Mycobacterium mantenii	WP_083099913	-------R----L-----R	------------K--NI----W----
Mycobacterium marinum	WP_012395247	---K---R---AM-----S	------------R---M---------
Mycobacterium monacense	WP_083045318	A-ED---E--TRH--D--R	-GYA------V-VA-PIL--------
Mycobacterium moriokaense	ORB19451	--AA---E--RRH--DVAE	---T--------V--SII---H----
Mycobacterium mucogenicum	OBJ45361	---V---G--MDH--C-LY	-E-T--------II-SKI--------
Mycobacterium nebraskense	WP_046182066	------TE----L-----S	---I------------T---------
Mycobacterium neoaurum	CDQ46888	A-AD--SA---AA-LQ-RR	---R--------VA-TM---------
Mycobacterium neworleansense	CRZ16149	--AD---E--AAA-----R	-D----------L--STL---H----
Mycobacterium obuense	WP_082133063	--AP-H-A--LDH-FVS-Q	---T--------L--ATI--------
Mycobacterium palustre	WP_085080055	-------E----------S	-------------A-DV---------
Mycobacterium paraense	WP_085095335	---A---E--A-L-----S	------------L--DM---------
Mycobacterium paraffinicum	WP_073880978	-------D----I-----S	---I---------I-QT---------
Mycobacterium parafortuitum	ORB27627	--AV---D--RRN--E-FE	----------F-T--DVL--------
Mycobacterium paraseoulense	WP_083175095	-------E---AL-----S	-------------I------------

Genus *Mycobacterium* (90/90)	*Mycobacterium parmense*	WP_085269066	-------A----L-----S		---I-----------QTL--------
	Mycobacterium peregrinum	OBB28747	--AD---E--TAA---T-F		-D----------L--STL--------
	Mycobacterium phlei	WP_061489841	A-AD---D--RRH-F---R		------------V--P-I--------
	Mycobacterium porcinum	ODR21483	--AD---E--AAA-----R		-E----------L--SNL--------
	Mycobacterium rhodesiae	WP_083120575	---V---A--GQH-HD-IA		------------I--SMI---T----
	Mycobacterium riyadhense	WP_085250469	--AK---K----------C		----------------M--G------
	Mycobacterium rufum	KGI70661	A-AV---A--ADN-FHAAE		------------LA-SMI---H----
	Mycobacterium rutilum	WP_083405422	A-AD---A--AAR----AQ		------------V--SM----F----
	Mycobacterium salmoniphilum	WP_078324076	---A-H-G--RAH-L--MH		S-YT------F-LA-DIM---T----
	Mycobacterium saopaulense	OHT88729	S-AD-H-E--RGH-L-AMH		S-YT------F-LA--IM---T----
	Mycobacterium saskatchewanense	WP_085257412	---A---R----------S		------------L--ST---------
	Mycobacterium scrofulaceum	WP_067271968	-------D----I-----S		---T-----------QT---------
	Mycobacterium setense	WP_064871478	--AD---A--TAA-F---Y		-E----------L--SL---------
	Mycobacterium sherrisii	WP_069402706	--A----D----I--T-L-		--Y-------------M----H----
	Mycobacterium shigaense	BAX93442	---KG--D-------Q---		----------------K---------
	Mycobacterium simiae	WP_061556238	--A----D----M--T-LV		--Y-------------M----H----
	Mycobacterium smegmatis	WP_080627992	P-AD---E-AAAH-LD--F		-Q----------L--PNL---W----
	Mycobacterium triplex	WP_036470186	A-AA---E--T-M------		--Y---------L---M---------
	Mycobacterium tusciae	ORB67146	---A---E--TRH--S-AD		---T--------V--SIM--------
	Mycobacterium ulcerans str. Harvey	EUA91539	---K---R---AM-----S		------------R---M---------
	Mycobacterium vaccae	WP_003929594	--AI---E---RN--H-NE		----------F-A--STI--------
	Mycobacterium vanbaalenii	WP_041307611	--AV---E---RN--L--E		-T--------F-A--ATL---H----
	Mycobacterium vulneris	OCB14179	--AD---E--AAA-----R		-E----------L--SNL--------
	Mycobacterium wolinskyi	WP_084356356	A-AD---E--ARS-FE-L-		-Q----------L--STL---W----
Other *Corynebacteriales*	*Nocardia abscessus*	WP_043692909	A-GP--HA-FAGY-VPNLV	A	AAEC---E--F-L--D-L---M---S
	Nocardia altamirensis	WP_069162572	A-GP--HQ-FAQH-LAGLV	D	AKEL---E--F-I--D-M---M---S
	Nocardia anaemiae	WP_062990901	D-GP--HR-FAQQ-LVELV	G	AQEI---E--F-L----M--AT---S
	Nocardia araoensis	WP_039798386	A-GP--HA-FARC-LANLV	A	AAEC---E--F-L--D-L---T---S
	Nocardia arthritidis	WP_063052817	A-GP--HA-FAGY-LPNLV	A	AAEC---E--F-L--D-L---T---S
	Nocardia asiatica	WP_043726333	A-GP--HA-FAAC-LPNLV	A	AGEW---E--F-L--D-L---T---S
	Nocardia beijingensis	WP_067807681	A-GP--HA-FAGA-LPNLV	A	AEEC---E--F-L--D-L---T---S
	Nocardia brasiliensis	WP_042259892	A-GP--HR-FAQH-LAGLV	D	A-E----E--F-L----L---M---S
	Nocardia exalbida	WP_040867147	A-GP--HA-LAGY-LPNLV	A	AAEC---E--F-L--D-L---T---S
	Nocardia gamkensis	WP_062972842	A-G---HA-FAGY-VPNLV	A	AAEC---E--F-L--D-L---T---S
	Nocardia niwae	WP_063018153	A-GP--HA-FARY-VPNLV	A	AAEC---E--F-L--D-I---T---S
	Nocardia pseudobrasiliensis	WP_062505999	--TAL--N-FRGNHGGQ-V	V	ASEF---E--F-RA--LM---T----
	Nocardia pseudovaccinii	WP_063042362	A-GP--HR-FAQL-LVELV	G	AQET---E--F-L----M--AT---S
	Nocardia seriolae	WP_033086712	Q-GP--HD--AAR-FAA--	A	A-ET---E--FAIA-D-I---T---S
	Nocardia tenerifensis	WP_040735585	A-GP--HN-FAQH-LAGLV	D	AKEI---E--F-L--D-L---M---S
	Nocardia vinacea	WP_051182144	A-GP--HR-FAQH-LVELV	G	AQEI---E--F-L----M--AT---S
	Nocardia vulneris	WP_043678062	A-GP--HR-FAQH-LAGLV	D	AQE----E--F-L----L---M---S
	Gordonia bronchialis DSM 43247	ACY20914	A--DH--A-F-RA-FG--R	R	-DGLG--E----V--D-I---S----
	Gordonia otitidis NBRC 100426	GAB33053	P-GEH-TA-FHTC-VDA-H	E	-VG-Q--E--F-L--R-I---T----
	Gordonia polyisoprenivorans	GAB25568	A-AGH--R-FRTA-I-Q-H	Q	-TG-Q--E--F-V--D-I---T----
	Gordonia rhizosphera NBRC 1606	GAB89896	P-GDH--E-LHTS-FG--H	R	-DGIQ--E--F-V--DVI---T----
	Gordonia westfalica	SDU54581	A-GDL--E-FARN-FGT-H	R	-AA-Q--E--F-V--D-I---T----
	Rhodococcus imtechensis	WP_063709751	A-AEHVFR-F-DN-LIRP-	L	P-Y----E--F-V--DVI---T---S
	Rhodococcus jostii	SEE47551	A-AEHVFR-FRAN-LIRP-	L	P-Y----E--F-V--DVI---T---S
	Rhodococcus koreensis	SEC05368	A-AEHVFR-FRDN-LIRP-	L	P-Y----E--F-V--DVI---T---S
	Rhodococcus maanshanensis	SEK87798	A-ADHVLR-FRAN-LLRPS	R	PEYI---E----V--DI-C--T----
	Rhodococcus opacus	ANS27163	A-AEHAFR-F-DN-LIRP-	L	P-Y----E--F-V--DVI---T---S
	Rhodococcus wratislaviensis	WP_037245014	A-AEYVFR-FQAN-LIRP-	L	P-Y----E--F-V--DVI--AT---S

Supplementary Figure 13

A partial sequence alignment of a conserved region of N-dimethylarginine dimethylaminohydrolase showing a one amino acid deletion that is specific for members of the genus *Mycobacterium* and absent in other *Corynebacteriales*.

Genus *Mycobacterium* except *"Abscessus-Chelonae"* Clade (>100/>100)

Species	Accession	31		75
Mycobacterium acapulcensis	WP_066808468	LVCGDPSETVDTVTVAVDATADVVAEVPERG		LLLAHHPLLLRGVDTV
Mycobacterium algericum	WP_083036380	------DDV-GS--I------A--D---DG-		----------------
Mycobacterium alsense	WP_083137538	------GDALES--I-----PA--D------		----------------
Mycobacterium angelicum	WP_083113532	------ADLLES--I-----PA--DT---A-		----------------
Mycobacterium aromaticivorans	WP_036347529	------DDV--S---------E-ATT-GP--		----------------
Mycobacterium arosiense	WP_083065725	------DDELRS--------PE--D---DAS		----------------
Mycobacterium asiaticum	WP_065037975	------ADD-ES--I-I-V-PA--D---DG-		----------------
Mycobacterium aurum	WP_087024598	------G-P-TS--IS----ES-L----DG-		----------------
Mycobacterium aurum	WP_048631802	------A-P--S-------------Q---Q-		----------------
Mycobacterium avium	WP_061504195	------D-P-ES--------PA--D---AG-		----------------
Mycobacterium avium subsp. avium	EUA40867	------D-P-ES--------PA--D---AG-		----------------
Mycobacterium avium subsp. paratuberculosis	ETB52299	------D-P-ES--------PA--D---AG-		----------------
Mycobacterium boenickei	WP_077741192	------D-P-ES--I-----EA--D------		----------------
Mycobacterium bohemicum	WP_085182249	------GDAL-S--------PA--D----A-		----------------
Mycobacterium bovis	WP_024457138	------DDV--S--------PA--DQ--QA-		---V------------
Mycobacterium canariasense	WP_062654585	------T-P-TS--I------E--D---DG-		M---------------
Mycobacterium canettii	WP_015290513	------DDV--S--------PA--DQ--QA-		---V------------
Mycobacterium chimaera	WP_072501273	------AD-LES--------PA--D---DA-		----------------
Mycobacterium chlorophenolicum	WP_048468800	--------PAES--------PE---TM----		----------------
Mycobacterium chubuense	WP_014816476	--------P-ES--------PG---Q--Q--		--------------S-
Mycobacterium colombiense	WP_064952110	------DDKLES--------PA--D---QA-		----------------
Mycobacterium conceptionense	CQD21231	------G-P-ES--I-----EA--D------		----------------
Mycobacterium cosmeticum	WP_036396767	--------P-TS---------E--D---DG-		----------------
Mycobacterium diernhoferi	WP_073854002	------A-P-TS--I-----ES-L-----G-		----------------
Mycobacterium doricum	WP_085191309	------D-P-ES--------EA-I-Q-----		----------------
Mycobacterium europaeum	WP_085242286	------DDALES--------PA-------G-		----------------
Mycobacterium fallax	WP_085100171	------A-P-TS--------EQ-I-R--DG-		----------------
Mycobacterium farcinogenes	CDP89252	------G-P-ES--I-----EA--D------		----------------
Mycobacterium flavescens	WP_069412801	------D---ES--------PA-A-------		----------------
Mycobacterium florentinum	WP_085220532	------ADVL-S--I-----PA--D----G-		----------------
Mycobacterium fortuitum	WP_061264855	------D-A-ES--I------A-ID------		----------------
Mycobacterium fragae	WP_085199759	------DDVLGS--I---------E---DG-		----------------
Mycobacterium gastri	WP_036415521	------ADV--S--------PA--D---DG-		----------------
Mycobacterium genavense	WP_025735072	------NDLL-S--I-----PA--DD---A-		----------------
Mycobacterium gilvum	WP_011893769	--------P-ES--I--------L-Q--QG-		----------------
Mycobacterium goodii	WP_049745996	------D-P-ES--I------Q------D--		----------------
Mycobacterium gordonae	WP_065043688	------A-K-ES--I-I----E-AD---DN-		--------------S-
Mycobacterium haemophilum	WP_054879806	------ADVLES--I-----PA-ID---DA-		----------------
Mycobacterium hassiacum	WP_005628329	------D-V-ES------------DD--D--		----------------
Mycobacterium heckeshornense	WP_048892420	------D-P--S--I-I----P-IDD--D--		----------------
Mycobacterium heidelbergense	WP_083074388	------DDAL-S--------PA--D---DA-		--------------S-
Mycobacterium heraklionense	WP_064890687	------DDL-SS--I-----SA--D---DG-		----------------
Mycobacterium houstonense	WP_084459202	------G-P-ES--I-----EA--D----G-		----------------
Mycobacterium icosiumassiliensis	WP_067974100	------DDL-SS--I-----SA--D---DG-		----------------
Mycobacterium indicus pranii	WP_043954764	------DDKLES--------PA--D---DA-		----------------
Mycobacterium interjectum	WP_085202761	------DDALGS--------PA--D----G-		----------------
Mycobacterium intermedium	WP_069420014	------DDVLSS--I------E--D---DG-		----------------
Mycobacterium intracellulare	WP_064937193	------DDKL-S--------PA--D---DA-		----------------
Mycobacterium iranicum	WP_064282384	--------P-ES--I----------Q--HG-		----------------
Mycobacterium komanii	CRL72785	----------E--------------Q--D--		----------------
Mycobacterium koreense	WP_085302065	------D-P-ES--I------A--D--A---		----------------
Mycobacterium kubicae	WP_085074546	------T-VL-S----I---PE--DQ--DG-		----------------
Mycobacterium kumamotonense	WP_065288762	------DDV-GS--I------A--D---DG-		----------------
Mycobacterium kyorinense	WP_065014629	------DDA-ES--I------A--D---DG-		----------------
Mycobacterium lacus	WP_085161834	------EDVL-S--I-----PA--DQ--QA-		----------------
Mycobacterium lentiflavum	CQD16310	------DDVL-S--I-----PE--D----A-		----------------
Mycobacterium liflandii	WP_015356210	------G-P-ES--------PA--D------		----------------
Mycobacterium litorale	WP_078018866	------DDV-ES---------E-A-T-DPG-		----------------
Mycobacterium longobardum	WP_085263968	------DDV-SS--I------A--D---DG-		----------------
Mycobacterium mageritense	WP_036438948	------G-R-ES--IS----EA---------		----------------
Mycobacterium malmesburyense	CRL71387	----------E---------SA--D---D--		----------------
Mycobacterium malmoense	WP_065444388	------DDA-ES--------PA--D---AG-		----------------
Mycobacterium mantenii	WP_083099178	------GDEFES--------PA--D---RA-		----------------
Mycobacterium marinum	WP_012394959	------G-P-ES--------PA--D------		----------------

Group	Species	Accession	Alignment	Boxed region	Alignment (cont.)
**Genus *Mycobacterium* except *"Abscessus-Chelonae"* Clade (>100/>100)**	*Mycobacterium marseillense*	WP_083019937	------DDALES--------PA--D---DA-		----------------
	Mycobacterium minnesotense	WP_083022510	------DDV-SS--I---------D----G-		----------------
	Mycobacterium nebraskense	WP_046186221	------DDALES--------PA--D---QG-		----------------
	Mycobacterium neworleansense	CRZ13723	------G-P-ES--I-----EA-IE------		----------------
	Mycobacterium nonchromogenicum	WP_085139793	------DDL-SS--I------A--D---DG-		----------------
	Mycobacterium noviomagense	WP_083088409	------DDA-ES--I------A--D---DG-		----------------
	Mycobacterium novocastrense	WP_067389064	--------A-E----------E-I----D--		----------------
	Mycobacterium palustre	WP_085077460	------D-A-GS--------PA--E----G-		----------------
	Mycobacterium paraense	WP_085103992	------DDALHS--------PA--D----G-		----------------
	Mycobacterium paraffinicum	WP_073875516	------DDRLES--------PT--E---HG-		----------------
	Mycobacterium parafortuitum	WP_083146611	--------P-ES---------E---Q-----		----------------
	Mycobacterium paraintracellulare	AFC53618	------DDKLES--------PA--D---DA-		----------------
	Mycobacterium parascrofulaceum ATCC BAA-	EFG78091	------DDA-ES--------PA--D---AG-		--------------R-
	Mycobacterium paraseoulense	WP_083172808	------DD-IEA------P-PA--D----G-		----------------
	Mycobacterium parmense	WP_085271328	------DDAL-S--------PA--D---DG-		----------------
	Mycobacterium peregrinum	WP_064881645	------D-P-ES--I-----EA--D------		----------------
	Mycobacterium phlei	WP_003885921	--------E--S---------E---Q-----		----------------
	Mycobacterium porcinum	WP_069424770	------D-P-ES--I-----EA--D------		----------------
	Mycobacterium pseudoshottsii	WP_086085340	------G-P-ES--------PA--D------		----------------
	Mycobacterium rhodesiae	WP_081479092	------DDV--S---------E-A-T-GS--		----------------
	Mycobacterium riyadhense	WP_085250035	------ADVL-S--I-----PA--E---QG-		---------FH-----
	Mycobacterium rufum	KGI68827	--------R--S---------E---T-----		----------------
	Mycobacterium scrofulaceum	WP_067269200	------DDALES--------PA--D-I-DG-		----------------
	Mycobacterium senuense	WP_085082762	------DDV-GS--I------A--D---DG-		----------------
	Mycobacterium septicum	WP_044523508	------D-P-ES--I-----EA--D------		----------------
	Mycobacterium setense	WP_064875037	------G-P-ES--I-----EA-ID------		M---------------
	Mycobacterium sherrisii	WP_069400944	------GDQLES--------PA--D---AA-		----------------
	Mycobacterium shigaense	BAX93274	------DDA--S--I------A--D---DG-		--------------S-
	Mycobacterium shimoidei	WP_069396249	------ADA-HS--I------A--D----G-		-------------H--
	Mycobacterium shinjukuense	WP_083046552	------EQA-HS--------PA--DQ---A-		----------------
	Mycobacterium simiae	WP_061559712	------DDLLES--I-----PA--D----A-		----------------
	Mycobacterium sinense	WP_013828600	------DDV-GS--I------A--D---DG-		----------------
	Mycobacterium smegmatis	WP_011729722	------D-P-ES--I--------I----D--		----------------
	Mycobacterium szulgai	WP_085672103	------ADVLES--I-----PA--DQ---A-		----------------
	Mycobacterium terrae	WP_085261077	------DDV-GS--I------A-ID---DG-		----------------
	Mycobacterium thermoresistibile	WP_085975100	--------P-ES--------PA-A-------		----------------
	Mycobacterium triplex	WP_036469916	------DDLL-S--I-----PA--D----A-		----------------
	Mycobacterium triviale	WP_069391478	------D-P-ES--I------A--D--A---		----------------
	Mycobacterium tuberculosis	WP_070891472	------DDV--S--------PA--DQ--QA-		---V------------
	Mycobacterium ulcerans	WP_011739501	------G-P-ES--------PA--D------		----------------
	Mycobacterium vaccae	WP_040542987	------A-P-TS---------E-A-G--DG-		--------------S-
	Mycobacterium vulneris	WP_065458320	------D-P-ES--I-----EA--D------		----------------
	Mycobacterium wolinskyi	WP_085149098	------D-P--S--------DS-IS------		----------------
	Mycobacterium xenopi	WP_085196085	--------P-ES--I-I----A--DA-----		----------------
	Mycobacterium yongonense	WP_065499397	------DDKL-S--------PA--D---DA-		----------------
***"Abscessus-Chelonae"* Clade (0/3)**	*Mycobacterium chelonae*	WP_070916247	------DQQI-A---------S-LD-MDNT-	SH	----------------
	Mycobacterium immunogenum	WP_064632344	------DQQI-----------A-LD-MDSE-	SH	----------------
	Mycobacterium saopaulense	WP_070911028	------DQRI-G--------TA-LD-MDGAA	SQ	----------------
**Other bacteria (0/>300)**	*Acaricomes phytoseiuli*	WP_018133830	--V-R-GAP-SRILF---P-V-----ALDW-	TD	-II-----F-K--NS-
	Acidipropionibacterium acidipropionici	WP_015069929	------ADS-S--AC-LE--DA--EAAIDA-	AQ	M-VV---------SS-
	Acidothermus cellulolyticus	WP_011719736	----E--QA-RRILF---PVPS-A--AI---	VD	--VT----Y---TSS-
	Actinophytocola xanthii	WP_075129884	------E---RR-L-C--PVEST-D-ALAID	AQ	-VV----------HG-
	Actinosynnema mirum	WP_012783502	-----R--P-SK-LFC--PVEST-D-AI-V-	AQ	--VS---------HG-
	Actinotalea fermentans ATCC 43279	KGM16919	T-----DQP-RR-LL-I---TA--D-AL-W-	AD	--WT-------A-HG-
	Alloactinosynnema album	SDJ46379	------A-E-TGIL-C--P-EST-D-AI-L-	AQ	-IV----------HG-
	Allokutzneria albata	SDN57763	-----RT-P-RS-L-C--PVEST-D-AL-L-	AD	-V-----------HG-
	Allosalinactinospora lopnorensis	WP_046469876	------ARR-ERILF---PV-E--D-AA-W-	AD	-IVT---------TS-
	Amycolatopsis methanolica	WP_017987012	------A-P-TD-LIC--PV-TTID-AI-T-	VQ	-VV----------HG-
	Arcanobacterium haemolyticum	WP_013169953	--A---HAP-SK-GF---PSP-T-E-AIA--	AQ	M-IT----F---TSS-
	Arsenicicoccus bolidensis	WP_029212720	--T---DQP-RS-LL---P-L--ID-AR-L-	SD	--VT---------HS-
	Arthrobacter alpinus	WP_082357881	--V-R-DAD-ERIMF---P-LE-IE-AL-W-	AK	--IT------K--NS-
	Blastococcus saxobsidens	WP_083878151	------A-E-RR-VF---P--A--D--V-T-	AQ	--VT----F-TP-HG-
	Brevibacterium ravenspurgense	KXZ58268	-AV---EAP-TH-HC-L-PSDE-I--AV-L-	AD	F-FT---------TS-
	Catenulispora acidiphila	WP_015794478	------E-P-R--GF---PV-A-ID-AL---	AD	--VT----F----HG-
	Cellulomonas bogoriensis	WP_035056870	T-----DQP-RRILL---P-STT-D-AE-WD	AD	--FT----F--P-HS-

Group	Species	Accession	Sequence
**Other bacteria (0/>300)**	*Chlamydia trachomatis*	CRH89195	-IV----DR-SNIGF---PCEAT-R-AI--- AQ M-IT----Y---TSS-
	Corynebacterium kutscheri	WP_046439081	------DDN-TK-VC-L-C-QE-ADAAVAS- AQ M-VV-----M---TS-
	Geodermatophilus brasiliensis	SDG52353	------A-A-RS-LF---P-DA--D-AI-A- VD -VVT------TP-HG-
	Glutamicibacter mysorens	WP_066138150	--T-RTGQQISKIVM---P-LE-IEDAI--- AD --IT---------TQ-
	Gordonia aichiensis	WP_005180680	-----RA-P-GRALIC--V-DA--DAAI-AD VD -IV-----------S-
	Kibdelosporangium phytohabitans	WP_054288740	------ADE-RRAL-C--P-EST-D-AV-L- AD -VV----------HG-
	Kutzneria albida	WP_030110347	-----RA-P--K-LFC--PVEATIE-AL-I- AQ -VV----------HG-
	Lechevalieria aerocolonigenes	WP_081902695	------R-E-TR-L-C--P-SST-D-AV-V- AQ -V-----------NG-
	Lentzea albidocapillata	SMD22043	------Q-E-TR-L-C--P-SST-D-AV--- AQ -V--------K--NG-
	Micromonospora chersina	SCL70581	--L-E-ANP-RR-AC---VVPET-E-ALAA- AD MIV----------SS-
	Nocardia araoensis	WP_039796906	------A-ELTR-LF------A--E-AIDWR AQ A-VV------------
	Nocardiopsis ganjiahuensis	WP_017588516	------AQ--ERILF---PVDA--D-AVTW- AD -VIT----M----TS-
	Nonomuraea coxensis	WP_026214065	--S---TQE-RR-LL---PV-A-AE-AL-W- AD -IVV----Y---TTS-
	Pseudarthrobacter chlorophenolicus	WP_045731604	--A-H-ASP-TK-MF---P-LE-IE-AV-W- AE --IT------K--TS-
	Pseudarthrobacter equi	SDT36439	--A-H-AAL-TK-MF---P-LE-IE-AV-W- AE --IT------K--TS-
	Pseudonocardia asaccharolytica	WP_028929789	------DDP-ER-L----PVTET-E-AI-T- AG --VT---------HG-
	Rhodococcus opacus	AII09953	------DD--A---I---P--A--D-AIDA- AD ---V------------
	Saccharomonospora cyanea	WP_005453845	------A-P-ER-L-C--PVTAT-E-AV-W- AQ -VV----------HG-
	Saccharothrix espanaensis	WP_015098406	-----RG-D-SR-LFC--PD-S--D-AESV- AQ -VV----------HG-
	Serinicoccus chungangensis	WP_058892550	--A---EQR-HR-LL---P-LE----AVRT- AD -VIT--------IHS-
	Streptomyces aurantiacus	WP_055515372	T-----DQE-SR-LF---PVQEI-D-AVKL- AD --VT----Y---TT--
	Streptosporangium canum	SFL06482	-------SE-RR-LF---PV-A-AD-ALHW- AD -IVT----Y---TTS-
	Thermobifida halotolerans	WP_068692834	------GQP-RR-LF---PV-E--D-AARW- AD -VIT---------TS-
	Thermocrispum municipale	WP_028851398	------EDE-ER-L-C--PVQAT---AE-L- AQ -VV----------SS-
	Trueperella bernardiae	KTF04872	--V---ADE-TK-GF---PCGA--D-AI--- AQ M-IT----Y---TSS-
	Tsukamurella paurometabola	WP_049825995	------AAE-TR-LAC--V-----DAAL-Q- AQ -IV-------------
	Xylanimonas cellulosilytica	WP_012878845	-IA----AP-RR-LL---PV----D-AL-W- AD -VVT----F-K--HS-
	Yuhushiella deserti	SFQ16741	------A-P-TR-L-C--VVGET---AA-L- AQ -IV----------HG-

Supplementary Figure 14

Detailed sequence information for the two amino acid deletion found in Nif3-like dinuclear metal center hexameric protein, which is shown in Figure 4. This deletion is specific for members of the genus *Mycobacterium* except the "*Abscessus-Chelonae"* clade, and absent in other bacteria.

Genus *Mycobacterium* except *“Abscessus-Chelonae”* Clade (130/132)

Species	Accession	106		161
Mycobacterium tuberculosis	CKM81105	VRTANSEIVDSPAHLDAALDRFGPPA	GDPAW	VVKDDRLAAGKGVVVT
Mycobacterium acapulcensis	WP_066810153	----R--V--N-------------DS	-EA--	-----G----------
Mycobacterium africanum	WP_031670259	--------------------------	S----	----------------
Mycobacterium algericum	WP_083037641	----R--------D-----S----D-	-EA--	-----G----------
Mycobacterium alsense	WP_083136508	----T--T-----R------------	-----	----------------
Mycobacterium angelicum	WP_083115989	----K-----N---------------	-E---	-----H----------
Mycobacterium aromaticivorans	WP_036341651	----G--T--N-------------A-	-Q---	-----G----------
Mycobacterium arosiense	WP_083066672	----T-----T--------E-----V	--A--	----------------
Mycobacterium arupense	WP_046190470	----R---I-N--D-----NN---E-	-EA--	-----G----------
Mycobacterium asiaticum	WP_065037890	----S-----N-G-------------	-----	-----S----------
Mycobacterium aurum	WP_087027350	----V-----N-G-------------	-QA--	-----G----------
Mycobacterium austroafricanum	WP_036377353	----V-----N---------------	-QA--	-----G----------
Mycobacterium avium	WP_084024772	----A-----T---------------	-EA--	----------------
Mycobacterium avium subsp. paratuberculosis	ETB48406	----A-----T---------------	-EA--	----------------
Mycobacterium bacteremicum	WP_083059445	----G-----N-G-------------	-QA--	-----G----------
Mycobacterium boenickei	WP_077740358	----S-----N---------------	-QA--	-----G---------S
Mycobacterium bohemicum	WP_085182646	----T--T--T--V------------	-----	----------------
Mycobacterium bovis	WP_044798678	--------------------------	-----	----------------
Mycobacterium brisbanense	WP_062831958	----S-----N-G-------------	-QA--	-----G----------
Mycobacterium canariasense	WP_062655808	----T-----N---------------	-QQ--	-----G----------
Mycobacterium canettii	WP_014000405	--------------------------	-----	----------------
Mycobacterium celatum	WP_062540780	----S------------------QS-	-EA--	----------------
Mycobacterium celeriflavum	WP_083006210	----R--V--N--------------G	-----	-----G----------
Mycobacterium chlorophenolicum	WP_048474005	----T-----N--------------G	--R--	-----G----------
Mycobacterium chubuense	WP_048417159	----T-----N--------------G	--R--	-----G----------
Mycobacterium colombiense	WP_064882305	----T-----T---------------	--A--	----------------
Mycobacterium conceptionense	CQD23795	----S-----N--R------------	-QA--	-----G---------S
Mycobacterium confluentis	WP_085153498	----T-----N-G-----------A-	-QR--	-----G----------
Mycobacterium conspicuum	WP_085234854	----A---I-N--N-----E------	-----	-----Q----------
Mycobacterium cosmeticum	WP_036397513	----T-----N---------------	-QR--	-----G----------
Mycobacterium diernhoferi	WP_073856645	----V-----N---------------	-QA--	-----G----------
Mycobacterium doricum	WP_085188455	-P--A-----N---------------	-EA--	-----G----------
Mycobacterium engbaekii	WP_085129800	----R--------D-----S----D-	-EA--	-----G----------
Mycobacterium europaeum	WP_085243033	----A--T--N--N------------	-----	----------------
Mycobacterium fallax	WP_085092526	----T-----N--R-----N------	-QA--	-----G----------
Mycobacterium farcinogenes	WP_036391841	----S-----N--R------------	-QA--	-----G---------S
Mycobacterium flavescens	WP_069413739	----R--V--N-------------AV	-----	-----G----------
Mycobacterium florentinum	WP_085223529	----S-----N---------------	-----	-----S----------
Mycobacterium fortuitum	WP_061262970	----S-----N---------------	-QA--	-----G---------S
Mycobacterium fragae	WP_085198843	----S-----N--R------------	DEA--	----------------
Mycobacterium gastri	WP_036411040	----A-Q---N-----V---------	-----	-----H----------
Mycobacterium genavense	WP_025736888	----A-----N-------------A-	-----	-----A----------
Mycobacterium gilvum	WP_011892505	----G-----N---------------	-QA--	-----G----------
Mycobacterium goodii	WP_049744818	----T--V--N-G-------------	-QA--	-----G----------
Mycobacterium gordonae	WP_065046934	----A-----N--N-----E------	-----	-----S----------
Mycobacterium haemophilum	WP_054879318	-H--R--T--R---------------	-----	----------------
Mycobacterium heckeshornense	WP_048889703	----A-----N--------A---VA-	-----	----------------
Mycobacterium heidelbergense	WP_083072292	----T--TI----R---T--------	-----	----------------
Mycobacterium heraklionense	WP_064998131	----R-----N--E-----T----D-	-EA--	-----G----------
Mycobacterium hiberniae	WP_085135818	----R--------D-----S----D-	-EA--	-----G----------
Mycobacterium holsaticum	WP_069405321	----R-----N--R-----------G	-EA--	-----G----------
Mycobacterium houstonense	WP_066899745	----S-----N-G-------------	-QA--	-----G---------S
Mycobacterium icosiumassiliensis	WP_067976066	----R--------D-----T----D-	-EA--	-----G----------
Mycobacterium insubricum	WP_083030432	----T--V--N--------N------	-QR--	-----G----------
Mycobacterium interjectum	WP_085203097	-P--T--------R------------	-----	-----A----------
Mycobacterium intermedium	WP_069419733	----R-----N--R--E---------	-----	-----H----------
Mycobacterium intracellulare	WP_064933908	----TT-------L------------	--A--	----------------
Mycobacterium iranicum	WP_064279599	----FG----N--N--T---------	-QA--	-----G----------
Mycobacterium kansasii	WP_063468636	----A---I-N---------------	-----	-----H----------
Mycobacterium komanii	CRL76089	----R-----N--R----------AG	-ES--	-----G----------
Mycobacterium kubicae	WP_085074975	----S-----T---------------	-----	-----H----------
Mycobacterium kumamotonense	WP_065288208	----R--------D-----G----D-	-EA--	-----G----------
Mycobacterium kyorinense	WP_065013725	----S-----N-------------T-	-SQ--	----------------
Mycobacterium lacus	WP_085159162	----K-----N------V-------S	-----	----------------

Genus *Mycobacterium* except "*Abscessus-Chelonae*" Clade (130/132)

Species	Accession			
Mycobacterium lentiflavum	CQD21230	-P--S-----N--------G------	-E---	-----S----------
Mycobacterium leprae	WP_010908779	---SR----N---R-----------V	--LS-	----------------
Mycobacterium lepromatosis	WP_045843684	-C-SR----N---R----------AV	--LS-	----------------
Mycobacterium litorale	WP_078020966	----T--T--N-------------A-	-Q---	-----G----------
Mycobacterium llatzerense	WP_071286479	----T-----N---------------	-QQ--	-----G---------S
Mycobacterium longobardum	WP_085264667	----R--------D-----S----E-	-EA--	-----G----------
Mycobacterium mageritense	WP_019348293	----S-----N--R------------	-QA--	-----G---------S
Mycobacterium malmesburyense	CRL67485	----R--V--N--R-----------G	-ES--	-----G----------
Mycobacterium malmoense	WP_065446400	----A--T------------------	-----	----------------
Mycobacterium mantenii	WP_083093946	-------------L--S--N------	--A--	----------------
Mycobacterium marinum	WP_020730725	----S-----N--R------------	-----	-----H----------
Mycobacterium marseillense	WP_083014667	----AT-------R------------	--A--	----------------
Mycobacterium minnesotense	WP_083026974	----R---I-N--D-----N----D-	--A--	-----G----------
Mycobacterium monacense	WP_083044855	----A-----N--------G------	-EA--	-----G----------
Mycobacterium moriokaense	WP_083153599	----T-----N--N-----------S	---S-	-----G----------
Mycobacterium mucogenicum	WP_064857353	----T-----N---------------	-QQ--	-----G---------S
Mycobacterium nebraskense	WP_046185973	----S--------------------G	-----	----------------
Mycobacterium neoaurum	CDQ45200	----T-----N--K------------	-QA--	-----G----------
Mycobacterium neworleansense	CRZ18927	----G-----N---------------	-QA--	-----G---------S
Mycobacterium nonchromogenicum	WP_085138432	----R--------E-----A----D-	-EA--	-----G----------
Mycobacterium noviomagense	WP_083085323	----A-----N--R-----G---VA-	-----	----------------
Mycobacterium novocastrense	WP_067395465	----R--V--N-------------DG	-EA--	-----G----------
Mycobacterium obuense	WP_046362206	----T-----N---------------	-EQ--	-----G----------
Mycobacterium orygis	WP_003403930	--------------------------	-----	------------A---
Mycobacterium palustre	WP_085078551	----F-----N---------------	-----	----------------
Mycobacterium paraense	WP_085095665	----A--------R------------	-----	-----A----------
Mycobacterium paraffinicum	WP_073871103	----V--T--------T---------	-----	----------------
Mycobacterium parafortuitum	WP_083141845	----G-----N---------------	-QR--	-----G----------
Mycobacterium paraintracellulare	WP_014383789	----AT-------L------------	--A--	----------------
Mycobacterium parascrofulaceum	WP_007166387	----A--T------------------	-----	----------------
Mycobacterium paraseoulense	WP_083168003	----A--T-----------------V	-----	----------------
Mycobacterium parmense	WP_085268373	-----T-------L-----------S	-----	----------------
Mycobacterium peregrinum	WP_064884712	----T-----N---------------	-QA--	-----G---------S
Mycobacterium phlei	AMO59503	----R--V--N---------------	-EQ--	-----G----------
Mycobacterium porcinum	WP_069425387	----S-----N---------------	-QA--	-----G---------S
Mycobacterium pseudoshottsii	WP_086084731	----S-----N--R------------	-----	-----H----------
Mycobacterium rhodesiae	WP_083118654	----G--T--N-------------A-	-Q---	-----G----------
Mycobacterium riyadhense	WP_085249598	-P--RG----N-----T---------	-----	-----H----------
Mycobacterium rufum	KGI69896	----S-----N-G------------G	--R--	-----G----------
Mycobacterium rutilum	SEH62830	----R-----N--------------G	-----	-----G----------
Mycobacterium saskatchewanense	WP_085255707	-S--V-----N--L-----------N	-----	----------------
Mycobacterium scrofulaceum	WP_067269404	----T--T------------------	-----	----------------
Mycobacterium senuense	WP_085084369	----R--------D-----S----D-	-EA--	-----G----------
Mycobacterium septicum	WP_044520531	----S-----N---------------	-QA--	-----G---------S
Mycobacterium setense	WP_064875699	----S-----N-------------S-	-QA--	-----G---------S
Mycobacterium sherrisii	WP_085166452	----S---I-N-G------E-----G	-----	-----S----------
Mycobacterium shigaense	BAX94540	----R--------K------------	-----	-----G----------
Mycobacterium shimoidei	WP_069394903	----A---I-N------------KSV	-E---	----------------
Mycobacterium shinjukuense	WP_083050832	----A-----N---------------	-----	----------------
Mycobacterium simiae	WP_061557240	----S---I-N-G------E------	-----	-----S----------
Mycobacterium sinense	WP_064854240	----R--------D-----S----D-	-EA--	-----G----------
Mycobacterium smegmatis	WP_011730846	----T--V--N-G-------------	-QA--	-----G----------
Mycobacterium szulgai	WP_085670792	----K-----N-------------A-	-E---	-----H----------
Mycobacterium terrae	WP_085261588	----R--------D-----S----E-	-EA--	-----G----------
Mycobacterium thermoresistibile	WP_040547168	----G--LI-N---------------	-----	-----G----------
Mycobacterium timonense	WP_083187302	----A-----T---------------	-EA--	----------------
Mycobacterium triplex	WP_036471659	----S--L--N--------------V	-E---	-----A----------
Mycobacterium triviale	WP_085110352	----L--V--N---------------	-NR--	-----G----------
Mycobacterium tusciae	WP_006241633	----T-----N--N-----------G	-Q-S-	-----G----------
Mycobacterium ulcerans	WP_011738808	----S-----N--R------------	-----	-----H----------
Mycobacterium vaccae	WP_003930461	----S-----N-G-------------	-QA--	-----G----------
Mycobacterium vanbaalenii	WP_041307124	----V-----N---------------	-QA--	-----G----------
Mycobacterium vulneris	WP_065457693	----S-----N---------------	-QA--	-----G---------S
Mycobacterium wolinskyi	WP_085142379	----G-----N---------------	-QA--	-----G----------
Mycobacterium xenopi	WP_085194745	----A---I-N--------A---VA-	-----	----------------

Group	Organism	Accession	Sequence	Insert	Sequence
**Genus *Mycobacterium* except “*Abscessus-Chelonae*” Clade (130/132)**	*Mycobacterium yongonense*	WP_065499680	----AT-------L------------	--A--	----------------
	Mycobacterium branderi	WP_083130961	----S-----N-----------AGEP	--	----------------
	Mycobacterium kyorinense	WP_045375736	----S--M--N-----------SGQ-	--	----------------
**“*Abscessus-Chelonae*” Clade (6/6)**	*Mycobacterium abscessus*	AMU23549	----R-----N-----------ASEP	--	-----G----------
	Mycobacterium chelonae	WP_070916695	----R-----N-----------ASEP	--	-----G----------
	Mycobacterium franklinii	WP_070936732	----R-----N-----------ASEP	--	-----G----------
	Mycobacterium immunogenum	WP_064631952	----R-----N-----------AGEP	--	-----G----------
	Mycobacterium salmoniphilum	WP_078324737	----R-----N-----------ASEP	--	-----G----------
	Mycobacterium saopaulense	WP_088413147	----R-----N-----------AGEP	--	-----G----------
**Other bacteria**	*Actinoalloteichus cyanogriseus*	WP_026419711	-P--H-----N--------A----TW		-----G----------
	Actinokineospora inagensis	WP_026423443	-P--H-----N--R--S--A----TW		-----G----------
	Actinomyces timonensis	WP_017177529	-P--EAAVCET--E-E-------A-H		---E-G----------
	Actinomycetospora chiangmaiens	WP_018330559	-P--RA-V--T--R-----A--E--W		-----G-------L--
	Alloactinosynnema album	SDI61911	-P--H-----N--R-----S----NW		-----G----------
	Allokutzneria albata	WP_030429978	-P--R--V--N--R-----G----TW		-----G----------
	Amycolatopsis nigrescens	WP_026360586	-P--H-----N--------A----TW		-----G----------
	Corynebacterium lubricantis	WP_026196297	-K--QA-RITDVSEI-GV--N---NY		-----G--G-------
	Dietzia cinnamea	WP_061230201	----RA-------D--E-------TW		-----G----------
	Gordonia amarae	WP_005185257	----HA-V--N--K----------TW		-----G----------
	Hoyosella altamirensis	WP_064441254	----HA----T--N----------TW		--------G------S
	Jiangella gansuensis	WP_026877698	-P--MAVVCETR-EVE---------Y		-----G----------
	Kutzneria albida	WP_025361726	-A--R--T--T--R-----R----TW		-----G----------
	Lechevalieria xinjiangensis	SEP76539	-P--H--V--N--R-----G----TW		-----G----------
	Millisia brevis	WP_066909645	----H-----N--L--E-------TW		-----G----------
	Nocardia crassostreae	WP_067531248	----H-----H-GE----------TW		-----G----------
	Pseudonocardia dioxanivorans	WP_041759199	-A--SC-------L---T----T--Y		-----G----------
	Rhodococcus kyotonensis	WP_068425022	----H--V-----K--E-------NW		-----G----------
	Saccharomonospora marina XMU15	EHR53715	-P--R--V--N--------G----TW		-----G----------
	Saccharothrix syringae	WP_033428343	-P--T--V--N--R-----TH---TW		-----G----------
	Sciscionella marina	WP_020499559	-P--R--T--N--R--K--AG---TY		-----G----------
	Segniliparus rotundus	WP_013137392	----R--T---T-S----------IW		----------------
	Skermania piniformis	WP_066474305	----HA-V--T--R----------TW		-----G----------
	Smaragdicoccus niigatensis	WP_018160407	----H--V--N--K----------VW		-----G----------
	Streptomyces endus	WP_067077927	-P--RAYVCTT--EI-E---A----Y		-----G----------
	Thermocrispum agreste	WP_038044680	-P--H--V--N---I---------IW		-----G----------
	Tomitella biformata	WP_024793577	----HA----N--E----------TW		-----G----------
	Tsukamurella paurometabola	WP_013128200	----R-----N---I---------TW		-I---G----------
	Williamsia muralis	WP_062795289	----H--------R----------TW		-----G----------
	Yuhushiella deserti	SFP77359	-P--H--V--N--------G----TW		-----G----------

Supplementary Figure 15

A partial sequence alignment of a conserved region of phosphoribosylamine-glycine ligase showing a five amino acid insertion that is specific for most members of the genus *Mycobacterium* except the *“Abscessus-Chelonae”* clade, and a two amino acid insertion specific for the members of the “*Abscessus-Chelonae”* clade that are both absent in most other bacteria.

Genus *Mycobacterium* except *“Abscesssus-Chelonae”* Clade (>100/>100)

Species	Accession	418	449
Mycobacterium algericum	WP_083039002	RVLTFAFISNDA	GPTGRTSIDALAAVLRTCGC
Mycobacterium alsense	WP_083138432	-------L--A-	--N--NAM----TK-WF---
Mycobacterium angelicum	WP_083115802	----------E-	--N--NA-----TK-WF---
Mycobacterium aromaticivorans	WP_081845060	-------L----	--M--VAL----S-------
Mycobacterium arosiense	WP_083066603	-------L----	--N--NVM----SR-WS---
Mycobacterium arupense	WP_046191112	------------	------A-------------
Mycobacterium asiaticum	WP_065035958	-------V----	--N--NAL----SR-WF---
Mycobacterium aurum	WP_048635521	------LL----	-------L--F--T--S---
Mycobacterium austroafricanum	WP_036375241	------LL----	------AL--F--T--S---
Mycobacterium avium	WP_062888222	------------	--N--NAL----TR-WF---
Mycobacterium avium subsp. avium 2285	EUA25080	------------	--N--NVM----TR-WF---
Mycobacterium bacteremicum	WP_083058566	------LL--N-	-------L-----K--L---
Mycobacterium bohemicum	WP_085182357	-------L----	--N--NAM----TR-WS---
Mycobacterium bovis BCG str. ATCC 35743	AHM09405	----------E-	--N--NAM----TK-WF---
Mycobacterium branderi	WP_083131180	------L-----	-----IA--NV--A-WS---
Mycobacterium brisbanense	WP_062828441	------LL--N-	-A----A---V-----S---
Mycobacterium canariasense	WP_062655982	------LM--N-	-------L--------S---
Mycobacterium canettii	WP_015291597	----------E-	--N--NAM----TK-WF---
Mycobacterium celatum	WP_085167493	------L-----	-----IA--NV--A-WS---
Mycobacterium celeriflavum	WP_083004620	------L-----	-----IAL-N---T--R---
Mycobacterium chlorophenolicum	WP_048468697	------LL--Q-	--S---AL--F--T------
Mycobacterium chubuense	WP_014817899	------LL--E-	--S---AL--V--T--S---
Mycobacterium colombiense	WP_007772293	----------N-	--N--NVM----TR-WS---
Mycobacterium conceptionense	WP_064898643	------L---N-	------A--E------S---
Mycobacterium confluentis	WP_085152357	-----S-L--N-	-----LA------T------
Mycobacterium conspicuum	WP_085235334	----------A-	--N--NA-----SK-W----
Mycobacterium cosmeticum	CDO07577	------LM--N-	-------L--------S---
Mycobacterium diernhoferi	WP_073856876	------L---N-	-------L-----K--S---
Mycobacterium doricum	WP_085188351	------LM----	--S---AL-----T--A---
Mycobacterium elephantis	WP_083043438	------------	--M-LVAL-T---T--S---
Mycobacterium engbaekii	WP_085128737	------------	------A-------------
Mycobacterium europaeum	WP_085241428	----------A-	--N--NAM----TK-WS---
Mycobacterium fallax	WP_085094877	-----S-L--G-	------A--N----------
Mycobacterium farcinogenes	WP_036391438	------L---N-	------A--E------S---
Mycobacterium flavescens	WP_069415296	------L---N-	-----IAL-N------S---
Mycobacterium florentinum	WP_085223238	----------A-	--N--NAM----TR-W----
Mycobacterium fortuitum	WP_064850944	------L---N-	------A--E------S---
Mycobacterium fragae	WP_085199010	------L-----	------A---V-ST-WS---
Mycobacterium gastri	WP_036418184	-------L----	-LN--NAM----TK-WF---
Mycobacterium genavense	WP_036467606	----------A-	--N--NAM-T--TR-W----
Mycobacterium gilvum	WP_011892306	------LL----	-------L--F--T--S---
Mycobacterium goodii	AKS32173	------L---N-	------A-----S-------
Mycobacterium gordonae	WP_065043411	------------	--N--NAM----TK-WF---
Mycobacterium haemophilum	WP_054880847	------L-A-G-	--H--AAL-TV--A--SYD-
Mycobacterium hassiacum	WP_005630740	------L-----	--M--VAL-NV-----S---
Mycobacterium heckeshornense	WP_048893629	------------	-----LA------R------
Mycobacterium heidelbergense	WP_083073751	------------	-----NAM----TR-WS---
Mycobacterium heraklionense	WP_064888675	------------	------A-------------
Mycobacterium hiberniae	WP_085134064	------------	------A-------------
Mycobacterium holsaticum	WP_069404819	------------	--M--VAL-H---T--S---
Mycobacterium houstonense	WP_084459416	------L---N-	------A---------S---
Mycobacterium icosiumassiliensis	WP_067976504	------------	------A-------------
Mycobacterium insubricum	WP_083033680	-------L--GG	-----VAL-N----------
Mycobacterium interjectum	WP_066916566	-------L----	--N--NAM----TK-WF---
Mycobacterium intermedium	WP_069419413	-------V----	--N--NAM----TK-WF---
Mycobacterium intracellulare	WP_064940287	------------	--N--NVM----TR-WF---
Mycobacterium iranicum	WP_064279789	------LL----	-------L--F--T--S---
Mycobacterium kansasii	KZS69356	-------L----	--N--NAM----TK-WF---
Mycobacterium komanii	CRL74165	------L---N-	-----IAL-N---T--A---
Mycobacterium koreense	WP_085303733	-----TL-----	------A--TV--T--S---
Mycobacterium kubicae	WP_085074722	------------	--N--NAM----TK-WF---
Mycobacterium kumamotonense	WP_065287559	------------	-----IA-------------
Mycobacterium kyorinense	ORW09086	------L-----	-----IA--NV--A-WS---
Mycobacterium lacus	WP_085157153	------------	--N--NAM----TK-WF---

Group	Species	Accession	Sequence	Boxed position	Sequence
Genus *Mycobacterium* except *"Abscesssus-Chelonae"* Clade (>100/>100)	*Mycobacterium lentiflavum*	CQD21819	----------A-		--N--NAM----TR-W----
	Mycobacterium leprae	WP_010907601	------------		-----AAM-E--TR-WM---
	Mycobacterium lepromatosis	WP_082082285	------------		-----AAM-E--TR-WA---
	Mycobacterium liflandii	WP_051045892	------------		--N--NAM----TT-WS---
	Mycobacterium longobardum	WP_085266486	------------		------A-------------
	Mycobacterium mageritense	WP_036432023	------L---N-		----------------S---
	Mycobacterium malmesburyense	CRL66705	------L---N-		-----IAL-N---T--A---
	Mycobacterium malmoense	WP_071513044	----------A-		--N--NAM----TR-WF---
	Mycobacterium mantenii	WP_083099024	-------L--N-		--N--NVM----TR-WS---
	Mycobacterium marinum	WP_012396646	------------		--N--NAM----TT-WS---
	Mycobacterium marseillense	ORA90434	------------		-----NVM----TK-WF---
	Mycobacterium minnesotense	WP_083026039	------------		------A-------------
	Mycobacterium moriokaense	WP_083155172	------------		----LIAL-NF---------
	Mycobacterium mucogenicum	WP_064860966	------L---N-		--E---AL-----AF-F---
	Mycobacterium nebraskense	WP_047322497	----------A-		--N--NVM----TK-WS---
	Mycobacterium neworleansense	CRZ17874	------L---N-		------A--E------S---
	Mycobacterium nonchromogenicum	WP_085138894	------------		------A-------------
	Mycobacterium noviomagense	WP_083088584	------------		-----LA------R------
	Mycobacterium novocastrense	WP_067394118	------L---N-		-----IAL-NV--T--A---
	Mycobacterium obuense	WP_046364492	------LL--Q-		--S---AL--F--T------
	Mycobacterium palustre	WP_085079439	----------A-		--N--NAM----SR-W----
	Mycobacterium paraense	WP_085103352	-------L----		--N--NAM----TK-WF---
	Mycobacterium paraffinicum	WP_084187707	----------A-		--N--NVV----TK-WS---
	Mycobacterium parafortuitum	WP_083142035	------LM----		-------L--F--T--S---
	Mycobacterium paraintracellulare	WP_014383735	------------		--N--NVM----TR-WF---
	Mycobacterium parascrofulaceum	WP_085981508	----------A-		--N--NVM----TK-WS---
	Mycobacterium paraseoulense	WP_083175966	----------A-		--N--NVM----TK-WS---
	Mycobacterium parmense	WP_085267353	-------L--A-		--N--NAM-----R-WS---
	Mycobacterium peregrinum	WP_064883965	------L---N-		------A--E------S---
	Mycobacterium phlei	WP_040634291	------L---N-		-----IAL-NV-----S---
	Mycobacterium porcinum	WP_069426330	------L---N-		------A--E------S---
	Mycobacterium pseudoshottsii L15	GAQ32269	------------		--N--NAM----TT-WS---
	Mycobacterium rhodesiae	WP_083120941	------------		--M--VAL--V-S-------
	Mycobacterium riyadhense	WP_085250820	----------E-		--N--NAM----TK-WF---
	Mycobacterium rufum	KGI70083	------LL--Q-		--S---AL--F--T--S---
	Mycobacterium rutilum	WP_083406959	------L---N-		-----IAL-N------S---
	Mycobacterium saskatchewanense	WP_085255259	-------L--A-		--N--NAM-----R-WS---
	Mycobacterium scrofulaceum	WP_067270625	----------A-		--N--NVM----TK-WS---
	Mycobacterium senuense	WP_085085665	------L-----		--------------------
	Mycobacterium setense	WP_039325721	------L---N-		------A--E------S---
	Mycobacterium sherrisii	WP_069400784	----------A-		--N--NAM--V--R-W----
	Mycobacterium shigaense	BAX94736	----------A-		--N--NAV-----R-W----
	Mycobacterium shimoidei	WP_069394925	------L-----		-----IA--N---A-WS---
	Mycobacterium shinjukuense	ORB63826	----------E-		--N--NA-----TK-WF---
	Mycobacterium simiae	WP_044508762	----------A-		--N--NAM--V--R-W----
	Mycobacterium sinense	WP_064856049	------------		------A-------------
	Mycobacterium smegmatis	WP_080628222	------L---N-		------A-----S---S---
	Mycobacterium szulgai	WP_085670126	----------E-		--N--NA-----TK-WF---
	Mycobacterium terrae	WP_085261415	------------		-----M--------------
	Mycobacterium thermoresistibile	WP_040546611	-----TL-----		--M---AL--V-----S---
	Mycobacterium timonense	WP_083186926	------------		--N--NAM----TR-WF---
	Mycobacterium triplex	CDO91150	----------A-		--N--NAM----TR-W----
	Mycobacterium triviale	WP_085110444	------L-----		------A---V--T--S---
	Mycobacterium tuberculosis	WP_061456217	----------E-		--N--NAM----TK-WF---
	Mycobacterium tusciae	WP_083126224	------------		-----VAL-T---AM-K---
	Mycobacterium ulcerans	WP_011741840	------------		--N--NAM----TT-WS---
	Mycobacterium vaccae	WP_040542062	------LL----		------AL--F--T------
	Mycobacterium vanbaalenii	WP_011782519	------LL----		------AL--F--T--S---
	Mycobacterium vulneris	WP_065459082	------L---N-		------A--E------S---
	Mycobacterium wolinskyi	WP_085143294	------L---N-		------A-------------
	Mycobacterium xenopi	WP_085193061	------------		-----LA------R------
	Mycobacterium yongonense	WP_008263448	------------		--N--NVM----TR-WF---
"Abscessus-Chelonae" Clade (0/6)	*Mycobacterium abscessus*	WP_052624961	-----SLMQ-H-	T	A-TA---V-NT-AV-RS---
	Mycobacterium abscessus subsp. bolletii	EHM21774	-----SLMQ-H-	T	A-TA---V-NT-AV-RS---
	Mycobacterium chelonae	WP_070940539	-----TLMQ-Q-	T	A-TA---V-NT-AV-RS---

```
"Abscessus-          ┌ Mycobacterium franklinii               WP_070939058    -----SLMQ-H- T A-TA---V-NT-AV-RS---
Chelonae" Clade     ─┤ Mycobacterium immunogenum              WP_064630627    -----SLMQ-H- T A-TA---V-N--AV-RS---
(0/6)                └ Mycobacterium saopaulense              WP_070912409    ------LMQ-Q- T A-TA---V-NT-AV-RS---
                     ┌ Actinokineospora enzanensis            WP_084477214    ---V--LM--GS N PADA-P----V-AA-RQ---
                     │ Actinomycetospora chiangmaiensis       WP_084681905    -L-V---M--GP D PVGA-PR-----TA-RG---
                     │ Amycolatopsis decaplanina              WP_039921337    -M-V--LM-SGS D QNK--A---VV-AT-HK---
                     │ Amycolatopsis halophila                WP_084036853    -L-V--LM-SGT G PNSA-P----V--A-RG---
                     │ Gordonia desulfuricans                 WP_059039318    ---S--LM-GGT S PADA-P----IPGD-RE---
                     │ Gordonia effusa                        WP_050949906    ---S--LM--GT S PADA-P----V-GA-RE---
                     │ Kutzneria albida                       WP_025354015    ---V--LM--GS N IDS--A---V--AT-RG---
                     │ Nocardia arizonensis                   WP_054816777    ------LM---R P PEVS-P----I-GT-RN---
                     │ Nocardia cyriacigeorgica               WP_048833850    ------LM-S-R P PEVS-P----I-AT-RN---
                     │ Nocardia elegans                       WP_063026257    --M---LM---R P PEAS-P----V-TT-RN---
Other bacteria      ─┤ Pseudonocardia acaciae                 WP_028925454    -L----LM--GT N PAES-PR---I-AA-RA---
                     │ Rhodococcus defluvii                   WP_031939458    ------LM--NS P PEVS-P----V-GT-RL---
                     │ Rhodococcus equi                       WP_022593940    ------LM---R P PEVS-P----V-GA-RL---
                     │ Rhodococcus erythropolis               WP_069147292    ------LM---R P PEVS-P----I-GA-RN---
                     │ Saccharopolyspora flava                SFS57194        -L-V--MM--GT S SSSA-P------DA-RG---
                     │ Saccharopolyspora shandongensis        SDY12614        -L-A--L---GT S SAAA-P---K---A-RT---
                     │ Streptoalloteichus hindustanus         WP_073479991    -L-V--L---GS Q QDP--R---SV--A-RG---
                     │ Tsukamurella paurometabola             WP_013125235     II--TLM-SGT P VDVA-P-M--VTAA-RG---
                     │ Tsukamurella pseudospumae              WP_068574066     ----TLM-SGT P VDVA-P-M--VTAA-RG---
                     │ Tsukamurella pulmonis                  WP_068531315     -F--TLM-SGT P VDVA-P-M--VTAA-RG---
                     └ Tsukamurella tyrosinosolvens           WP_068523644     ----TLM-SGT P VDVA-P-M--VTAA-RG---
```

Supplementary Figure 16

A partial sequence alignment of a conserved region of D-alanyl-D-alanine carboxypeptidase/D-alanyl-D-alanine-endopeptidase showing a one amino acid deletion that is specific for members of the genus *Mycobacterium* except the "*Abscessus-Chelonae"* clade, and absent in most other bacteria.

Genus *Mycobacterium* except *“Abscessus-Chelonae”* Clade (84/84)

		193		228
Mycobacterium africanum	WP_031668340	AASVAVADLASQLGGA	GG	LGDAVGRAATVLLESLVE
Mycobacterium angelicum	WP_083114416	---------G---RV	--	-------S----------
Mycobacterium aromaticivorans	WP_036344462	----------AR-D-S	--	-------S----------
Mycobacterium arosiense	WP_083062973	----------G--RSP	D-	-------S----------
Mycobacterium asiaticum	WP_065036895	----------GG-HKG	D-	-------S----------
Mycobacterium aurum	WP_087032364	-----------A-D-H	--	------------V-T---
Mycobacterium avium	WP_062906375	----------E--RSP	D-	-------S----------
Mycobacterium bacteremicum	WP_083055852	---I------GA-D-H	--	------------V-T---
Mycobacterium bohemicum	WP_085182928	----------G--RSP	D-	-------S----------
Mycobacterium bovis	WP_011799260	----------------	--	------------------
Mycobacterium brisbanense	WP_062827294	------S------N-S	--	-A-----S----V-T---
Mycobacterium canariasense	WP_062655367	T---------GAFD-H	--	----I-------V-T---
Mycobacterium canettii	WP_015290620	----------------	--	------------------
Mycobacterium celeriflavum	WP_083001237	------S------N-R	--	-A-----S----V-T---
Mycobacterium chimaera	WP_089151592	----------G--SSP	E-	-------S----------
Mycobacterium chubuense	WP_014816577	------S----HIN-Q	--	-A-----S----V-----
Mycobacterium colombiense	WP_007770255	----------G--RSP	D-	-------S----------
Mycobacterium confluentis	WP_085151265	----------A--D-R	--	------------------
Mycobacterium conspicuum	WP_085235397	----------E--H--	-S	--------------T---
Mycobacterium cosmeticum	WP_036396170	T----------AFD-H	--	----I-------V-T---
Mycobacterium diernhoferi	WP_073853761	-----------G-H-H	-R	------------V-T---
Mycobacterium europaeum	WP_085240317	----------G--RAP	D-	-------S----------
Mycobacterium farcinogenes	CDP89102	---I--S----H-N-Q	-A	-A----------V-T---
Mycobacterium flavescens	WP_069412910	------S----H-N-N	--	-A-----S----V-T---
Mycobacterium florentinum	WP_085220638	----------Q-----	--	----I--S----------
Mycobacterium fragae	WP_085199658	-HS	-S	-SN----S----------
Mycobacterium gastri	WP_036408441	---I------G--R--	--	-------S----------
Mycobacterium genavense	WP_025735542	----------Q-----	--	----I--S----------
Mycobacterium gilvum	WP_011893581	T--I--S----H---Q	-R	-AN----S----V-----
Mycobacterium goodii	WP_049745897	--I--S----H-N-T	DR	-A----------V-T---
Mycobacterium gordonae	WP_065042552	----------GG-H-G	D-	-------S----------
Mycobacterium haemophilum	WP_047315087	-FR-T	--	-AN-I--S----------
Mycobacterium hassiacum	WP_005628529	------S-----IQ-D	VR	-AN---------V-T---
Mycobacterium heckeshornense	WP_048890751	H-D--	--	------------V-----
Mycobacterium holsaticum	WP_069408135	--D-T	--	-S--I-------V-T---
Mycobacterium houstonense	WP_066898249	---I--S---TH-N-Q	-A	-A----------V-T---
Mycobacterium intermedium	WP_069420563	----------A--RAG	D-	-------S----------
Mycobacterium intracellulare	WP_064933124	----------GR-SSP	E-	-------S----------
Mycobacterium iranicum	WP_064282279	---I--S----H-N-Q	--	-A-----S----V-T---
Mycobacterium kansasii	WP_063471782	---I------G--S--	--	-------S----------
Mycobacterium komanii	CRL70531	---I---------D-H	--	-A--I--S----V-T---
Mycobacterium lacus	WP_085161020	----------G--N--	--	F------S----------
Mycobacterium lentiflavum	CQD17214	---------------H	--	----I--S----------
Mycobacterium leprae	WP_010907841	---A-----VGRFR-T	--	-AN----S-N--------
Mycobacterium lepromatosis	WP_045842613	---A-----VG-FR-T	--	-AN--V-S-S--------
Mycobacterium liflandii	WP_015356478	----------G--S--	--	-------S----------
Mycobacterium litorale	WP_078021913	----------AR-D-S	--	-------S----------
Mycobacterium malmesburyense	CRL74192	---I---------D-H	--	-A-----S----V-T---
Mycobacterium malmoense	WP_065443784	---I------G--RAP	D-	-------S----------
Mycobacterium marinum	WP_012395300	----------G--S--	--	-------S----------
Mycobacterium moriokaense	WP_083155459	---I--S----G-NDN	TR	-A-----S----V-T---
Mycobacterium mucogenicum	WP_061009708	---I--------MD-H	--	------------V-T---
Mycobacterium nebraskense	WP_046185707	---I------G--RAP	D-	-------S----------
Mycobacterium neworleansense	CRZ13602	---I--S----H-N-H	-A	-A-----S----V-T---
Mycobacterium paraffinicum	WP_073873799	---I------G--RAP	D-	-------S----------
Mycobacterium parafortuitum	WP_083145217	T-----S---TH-N-Q	-T	-A-----S----V-T---
Mycobacterium parascrofulaceum	WP_007169606	---I------G--RAP	D-	-------S----------
Mycobacterium paraseoulense	WP_083169167	---I------G--RAP	D-	-------S----------
Mycobacterium parmense	WP_085267530	----------G--R-G	D-	-SH----C------T---
Mycobacterium peregrinum	WP_064877965	---I--S---TH-N-H	-A	-A-----S----V-T---
Mycobacterium phlei	WP_040635619	---I--S------N--	-K	-A----------V-T---
Mycobacterium porcinum	WP_075921339	---I--S----H-N-Q	-A	-A-----S----V-T---
Mycobacterium rhodesiae	WP_083121461	----------AR-D-S	--	-------S----------
Mycobacterium riyadhense	WP_085252546	E-R--	--	-------S----------

Group	Species	Accession	Sequence	Insertion	Sequence
Genus *Mycobacterium* except *“Abscessus-Chelonae”* Clade (84/84)	*Mycobacterium rufum*	KGI68917	---I--S----H-N-Q	--	-A----------V-T---
	Mycobacterium rutilum	WP_083408235	------S----H-N-N	-R	-A-----S----V-T---
	Mycobacterium saskatchewanense	WP_085254734	---I------G--RSP	D-	-------S----------
	Mycobacterium scrofulaceum	WP_083174230	---I------G--RAP	D-	-------S----------
	Mycobacterium septicum	WP_044523103	---I--S---TH-N-H	-A	-A-----S----V-M---
	Mycobacterium setense	WP_039380680	---I--S---TH-N-Q	-T	-A-----S----V-T---
	Mycobacterium sherrisii	WP_069398906	------SE--A----T	--	-------S----------
	Mycobacterium shigaense	BAX93501	----------G--D--	--	-------S----------
	Mycobacterium shinjukuense	WP_083046356	----------GK-R--	--	-SN---------------
	Mycobacterium simiae	WP_044506959	------S---K----T	--	-------S----------
	Mycobacterium smegmatis	WP_003895873	--I--S----H-N-S	DR	-------S----V-T---
	Mycobacterium szulgai	WP_085669717	----------G---R-	--	-------S----------
	Mycobacterium thermoresistibile	WP_003926856	---A--S--VA----Q	-R	FS------S---V-T---
	Mycobacterium triplex	WP_036470260	----------Q-----	--	----I--S----------
	Mycobacterium tuberculosis	WP_070898250	----------------	--	------------------
	Mycobacterium tusciae	WP_083125294	---I---------N-N	--	-A-----S--M-V-T---
	Mycobacterium ulcerans	WP_011741366	--------F-G--S--	--	-------S----------
	Mycobacterium vaccae	WP_003928535	------S----H-N-Q	--	-AN----S----V-T---
	Mycobacterium wolinskyi	WP_067852040	------S---TH-N-S	D-	-A-----S----V-T---
	Mycobacterium xenopi	WP_085193584	H-D--	--	------------V-----
“Abscessus-Chelonae” Clade (0/6)	*Mycobacterium abscessus*	WP_016888444	-------E--E-SDDD		-R-PIT------V-T---
	Mycobacterium abscessus subsp. bolletii	EHM20306	-------E--E-SDDD		-R-PIT------V-T---
	Mycobacterium chelonae	WP_046253229	-------E--E-SDDD		-RNPLT------V-T---
	Mycobacterium saopaulense	WP_070911137	-------E--E-SDDD		-R-PLT------V-T---
	Mycobacterium franklinii	WP_070935446	-------E--D-SDDD		-R-PLT------V-T---
	Mycobacterium immunogenum	WP_043075899	E------E--E-SSDD		-RN-LT------V-T---
Other bacteria (0/>200)	*Corynebacterium ammoniagenes*	WP_003849314	E-TAHLNV-EEHVPLD		IRPHLRK-----V-T---
	Corynebacterium argentoratense	WP_021011948	D---SI-E--EEAPSD		IR-V-I-C----I-T---
	Corynebacterium casei	WP_006822345	--TAHLSE-EESAPLD		IRPHLRN-----V-T---
	Corynebacterium durum	WP_006064060	D---SL-----NAPED		IR---I-SS---I-T---
	Corynebacterium lactis	WP_053412527	D--I-I----VNAPRN		-R-VII-CS---V---I-
	Corynebacterium lubricantis	WP_018296058	D--TS--E--ASAPPE		IA-H-M--------T--D
	Corynebacterium maris	WP_020935356	E--TSL-E--D-VPPE		-HEHML------R-T--D
	Corynebacterium ulceribovis	WP_018024493	D-AAGL----ANGPSE		-R-TIT-C----I-E-L-
	Corynebacterium vitaeruminis	WP_048759610	D--I-IS---ANAPRD		-R-VII-CS---V---I-
	Gordonia kroppenstedtii	WP_018177412	---H---E--NHAP-D		-RQTII-V----V-T---
	Gordonia malaquae	WP_008380311	D------E--THAPRE		-QEP-VTV----V-T---
	Gordonia polyisoprenivorans	WP_006370641	E--AS--E--NNAPAD		-R--TVNV----V-T---
	Gordonia rhizosphera	WP_006332623	---A---E--NEAPDD		IR---INV----V-T---
	Nocardia arthritidis	WP_063049205	S--A---E-PEHAPVR		-R-VLV-VS---V-T---
	Nocardia asiatica	WP_043721898	---A---E-PEHAPLR		-R-VLV-VS---V-T---
	Nocardia beijingensis	WP_067801239	---A---E-PEHAPLR		-R-VLV-VS---V-T---
	Nocardia brevicatena	WP_040834256	T--A-----PE-APLR		-R-VLI-VS-M-V-T---
	Nocardia crassostreae	WP_067541313	I--A---E-PEHAPAR		-R-VLV-VS---V-T---
	Nocardia cyriacigeorgica	WP_014349754	N--A---Q-PE-APPR		-R-VLV-V----V-T---
	Pseudonocardia ammonioxydans	SFN32268	E--A---E-PETGPDD		-RVLLTQI----I-T---
	Pseudonocardia autotrophica	WP_073577998	E--A---E-PDTAPED		-RTPLLHL----I-T---
	Rhodococcus aetherivorans	WP_050035407	---I--SE--EDAPDE		-R---I-C----V-T---
	Rhodococcus corynebacterioides	WP_068146922	Q--I--SE--DDAPDD		-R---I-S----V-T---
	Rhodococcus defluvii	WP_031938275	---I---E--EDAPEN		-R---V-S----V-T---
	Rhodococcus equi 103S	CBH48951	---I--SE--EDAPEN		-R---I-S----V-T---
	Rhodococcus fascians	WP_032379171	-------E--ENAPAG		-R--MI-S----V-----
	Rhodococcus jostii	SEC79455	---I--SE--DEAPED		-R---I-S----V-T---
	Tsukamurella paurometabola	WP_013127378	D--M------PLAPRD		MR---I-IS---V-T---
	Tsukamurella pseudospumae	WP_068572504	D--MG-----PLAPRD		-R---I-VS---V-T---
	Tsukamurella pulmonis	WP_068529936	D--MG-----PLAPRD		-R---I-IS---V-T---
	Tsukamurella tyrosinosolvens	WP_068521293	D--MG-----PLAPRD		-R---I-VS---V-T---

Supplementary Figure 17

A partial sequence alignment of a conserved region of heat-inducible transcriptional repressor HrcA showing a two amino acid insertion that is specific for members of the genus *Mycobacterium* except the “*Abscessus-Chelonae”* clade, and absent in other bacteria.

Group	Species	Accession	161		199
***"Abscessus-Chelonae"* Clade (6/6)**	*Mycobacterium abscessus*	WP_052618900	APQGVTAVQECAE	RFGPST	SVRLFTATIDEGLNDAAYIV
	Mycobacterium abscessus subsp. bolletii	EIU81318	--------R----	------	--------------------
	Mycobacterium chelonae	WP_070915252	-----E----GVA	-Y----	-A------------------
	Mycobacterium franklinii	WP_070938642	-----A------T	------	--------------EV----
	Mycobacterium immunogenum	WP_064627902	--------R----	------	--------------AE----
	Mycobacterium saopaulense	WP_070909505	-----A------A	-Y-RTA	--------------ED----
**Other *Mycobacterium* (0/>100)**	*Mycobacterium algericum*	WP_083037894	--E-IA-LKNT-A		D--M---A--D---EN----
	Mycobacterium alsense	WP_083139288	--E-IA-LDKA-P		DA----VAV-D---EI----
	Mycobacterium angelicum	WP_083111192	--E-LA-LE-A-P		NA-V--VAV-----ES----
	Mycobacterium aromaticivorans	WP_036340448	--E-IA--AKV-P		-A--------D----V----
	Mycobacterium arosiense	WP_083067050	--E-LA--EKA-P		DA-V---A--K---KS----
	Mycobacterium asiaticum	WP_065037377	--E-IA--EKV-P		-A-----AV-D---EI----
	Mycobacterium aurum	WP_048630223	--E-LA-LEQM-P		EL--I-----S----I----
	Mycobacterium avium	WP_080772843	--E-LA--G-T-P		DA-V---AV-K---K-----
	Mycobacterium avium subsp. paratuberculosis	OUZ02257	--E-LA--G-T-P		DA-V---AV-K---K-----
	Mycobacterium bacteremicum	WP_083059235	--E-IA-LEQV-P		GI--V-----D---EI----
	Mycobacterium boenickei	WP_077743411	----IE-IEKV-P		DM--V---------EI----
	Mycobacterium bohemicum	WP_085182125	--E--A-LEKT-P		DA-----AV-DR--EI----
	Mycobacterium bovis	WP_003900012	--E-IA-L-KA-P		N------A------EV----
	Mycobacterium branderi	WP_083129993	--E-IA-LEKT-P		Y--V---AV-D---EN----
	Mycobacterium brisbanense	WP_062830231	--E-IA-LEKI-P		GI--I-----D---EI----
	Mycobacterium canariasense	WP_062659791	--E-IA-LERA-P		GI--V---V-----EI----
	Mycobacterium canettii	WP_044097309	--E-IA-L-KA-P		N------A------EV----
	Mycobacterium celatum	WP_062541135	--E-LA-LEKA-T		F--V---AV--R--EN----
	Mycobacterium celeriflavum	WP_082999773	--E-LA--EKA-P		NI--I------C---I----
	Mycobacterium chimaera	WP_089152138	--E-LA---KA-P		DA-V---AV-K---KS----
	Mycobacterium chlorophenolicum	WP_048471569	--E--A-LEKA-P		EL-----S--S---EI----
	Mycobacterium chubuense	WP_014814504	--E-LATLEKM-P		EL-----AV-T---------
	Mycobacterium colombiense	WP_075233674	--E-IA--RKA-P		DA-V---AV-K---K-----
	Mycobacterium conceptionense	WP_064894751	----IA-IEKV-P		DM--I---------EI----
	Mycobacterium confluentis	WP_085149124	-----S-IG-A-P		NA------------EI----
	Mycobacterium conspicuum	WP_085231528	--E-LA-LEKT-P		N--V---AV-D---EIF---
	Mycobacterium diernhoferi	WP_073859576	--E-IA-LEKA-P		DM--I----------I----
	Mycobacterium doricum	WP_085187089	--E-IATL-RA-P		DM--------DC-DE-----
	Mycobacterium farcinogenes	WP_036388831	----IA-IEKV-P		GM--I---------EI----
	Mycobacterium florentinum	WP_085219965	--E-LA--EKI-P		DA-V---AV-K---K--F--
	Mycobacterium fortuitum	WP_061262772	----IQ-LE-A-P		DI--I---------EI----
	Mycobacterium fragae	WP_085198559	--E-IA-LEKA-P		N--V---AV-D----V---M
	Mycobacterium gastri	WP_036410341	--E-IA-LEKV-P		NA-----AV-----E-----
	Mycobacterium gilvum	WP_013472606	--E-LAL-EKT-P		EL--Y--A--S----I----
	Mycobacterium goodii	WP_049747975	----IA-LEAV-P		NM--I---V-----EI----
	Mycobacterium gordonae	WP_065046308	--E-IA--EKV-P		NA--Y--A------ET-F--
	Mycobacterium haemophilum	WP_047313379	--E-IA--EKT-P		NA-----AV-----E-K---
	Mycobacterium hassiacum	WP_005631381	----LA--EKV-P		GI--------Q---EI----
	Mycobacterium heckeshornense	WP_048891852	--E-LA-LEKA-P		N---Y--A---R--EI----
	Mycobacterium heidelbergense	WP_083076380	-E-IA-LEKA-P		NA-----A--D---EIT---
	Mycobacterium heraklionense	WP_064890878	--E--A-LAQA-P		G------AV-D---ED----
	Mycobacterium holsaticum	WP_069403798	--E-LA-LEKV-P		NL---------C---I----
	Mycobacterium houstonense	WP_066903741	----IA-LE-V-P		EM--I---------EI----
	Mycobacterium icosiumassiliensis	WP_067970469	--E--A-LEQA-P		A----A-S--D---ED----
	Mycobacterium interjectum	WP_085203323	--E--A-LEKA-P		EA----VAV-D---EI----
	Mycobacterium intermedium	WP_069420430	--E-IA-LEKV-P		NL-----A------EI----
	Mycobacterium intracellulare	WP_064939522	--E-LA---KA-P		DA-V---AV-K---KS----
	Mycobacterium iranicum	WP_024448940	--E-LA-LEKA-P		EL-----S--S---EI----
	Mycobacterium kansasii	WP_063469399	--E-IA-LEKV-P		NA-----V------E--F--
	Mycobacterium komanii	CRL75563	--E-LA-LAKA-P		DI--V---------EV----
	Mycobacterium kubicae	WP_085072770	--E-IA--EQVVP		NA-----AV-G------F--
	Mycobacterium kyorinense	WP_045381475	--E-LA-LEKT-P		H--V---AV-D--DEN----
	Mycobacterium lacus	WP_085159419	--E-LA-LEKV-P		NI----V-V-D---E-----
	Mycobacterium lentiflavum	CQD07660	--E-LA--E-I-P		DA-V---AV-K---KS----
	Mycobacterium liflandii	WP_015354800	-----S-LEKA-P		NA----VA--D---EE----
	Mycobacterium malmesburyense	CRL78543	--E-LA-LEKA-P		N---V----------V----
	Mycobacterium malmoense	WP_065442482	--E--A-LEKT-P		AA----VAV-D--D-N----
	Mycobacterium mantenii	WP_083098791	--K-LA--EKA-P		NA-V---AV-K---KS----

Other *Mycobacterium* (0/>100)	*Mycobacterium marinum*	WP_012393096	-----A-LEKA-P	NA----VA--D---EE----
	Mycobacterium marseillense	WP_083017467	--E-LA---KA-P	DA-V---AV-K---KS----
	Mycobacterium moriokaense	WP_083153853	--E-LA-LEAV-P	-T--Y---------EV----
	Mycobacterium mucogenicum	WP_064858277	--E-IAL-EKAVP	-LK-------AE---D----
	Mycobacterium neworleansense	CRZ15766	----IA-IEKV-P	DM--V---------EI----
	Mycobacterium nonchromogenicum	CRL72562	--E--A-LEKA-P	G-------V-D----D----
	Mycobacterium noviomagense	WP_083087086	--E-LA-LENA-P	N---Y--A---R--EI----
	Mycobacterium novocastrense	WP_067396047	--E-LA-LEKA-P	NI--V------C---I----
	Mycobacterium obuense	WP_046366798	--E--A-LEKA-P	QIK----S--A----V----
	Mycobacterium palustre	WP_085080268	--E-IA-LGKA-P	DA----VAV-D---EI----
	Mycobacterium paraense	WP_085096177	--E-IA-LEKA-P	GA----VAV-D---EI----
	Mycobacterium parafortuitum	WP_083146795	--E-LRTLEKV-P	EL--Y--A--S----I----
	Mycobacterium parascrofulaceum	EFG76139	--E--A-LEKT-P	AA----VAV-D--D-N----
	Mycobacterium peregrinum	WP_064879401	----IA-LEKV-P	EI--I---------EI----
	Mycobacterium phlei	WP_003886451	--E-IA-LEKA-P	D---Y---V-Y---ES----
	Mycobacterium porcinum	WP_069427230	----IE-IEKV-P	DI--V---------EI----
	Mycobacterium pseudoshottsii	WP_086084680	-----S-LEKA-P	NAW---VA--D---EE----
	Mycobacterium rhodesiae	WP_083121556	--E-IA--AKV-P	-A--------D---EV----
	Mycobacterium riyadhense	WP_085251045	--E-LA-LEKA-P	TA-V--VAV-----Q-----
	Mycobacterium rufum	KGI67221	--E-IA-LEKA-P	ELK-I--S--S----I----
	Mycobacterium rutilum	WP_083410032	--E-LA-LEQA-P	DI--I-----DH--E-----
	Mycobacterium saskatchewanense	WP_085253640	--E-IA-LGDS-P	NA-----AV------I----
	Mycobacterium scrofulaceum	WP_083179245	--E-IA-LEKT-P	GA----VAV-D--D-N----
	Mycobacterium senuense	WP_085087668	--E--A-LKNT-V	D------A--D---QN----
	Mycobacterium septicum	WP_044516517	----IA-IEKV-P	EM--I---------E-----
	Mycobacterium setense	WP_039317346	----IA-IEKI-P	EM--V---------EI----
	Mycobacterium sherrisii	WP_084229256	--E-IA--E-I-P	NA-V---AV-N---ES-F--
	Mycobacterium shigaense	BAX91284	--E-LA--EKI-P	N--V---A--K---T--F--
	Mycobacterium shinjukuense	WP_083048693	--E-IA-LEQA-P	N---V--A--D---EI----
	Mycobacterium sinense	WP_064856711	--E-IA-LGNADP	G-----VAV----DEN----
	Mycobacterium smegmatis	WP_003893093	----IA-LEAV-P	DM--I---V-----EI----
	Mycobacterium szulgai	WP_068033600	--E-IA--EQVVP	NA-----AV-G------F--
	Mycobacterium thermoresistibile	WP_003923603	--E-LA--EQVRP	DL-----AV--R--EI-F--
	Mycobacterium triplex	WP_084163328	--E-LA--E-I-P	DA-V---AV-K---KS----
	Mycobacterium tuberculosis	WP_069334357	--E-IA-L-KA-P	N------A------EV----
	Mycobacterium tusciae	WP_083124945	--E-LA-LESV-P	DL---------R---I----
	Mycobacterium ulcerans Agy99	ABL04996	-----S-LEKA-P	NA----VA--D---EE----
	Mycobacterium vaccae	WP_003929459	--E-LRTLEKV-P	RLK-------S----I----
	Mycobacterium vulneris	WP_085290258	--E-IA--RKA-P	DT-V-A-A--K---K-----
	Mycobacterium wolinskyi	WP_067843104	--E-IA-LEAV-P	DM--I---V-----EI----
	Mycobacterium xenopi	WP_085197203	--E-LA-LEKA-H	N---Y--A---R--EI----
	Mycobacterium yongonense	WP_065500922	--E-LA---KA-P	DA-V---AV-K---RS----
Other bacteria	*Actinoalloteichus cyanogriseus*	WP_026419383	--E--EHLRRSGL	PL-VV--S--QR---SG---
	Cryptosporangium aurantiacum	SHN45425	--E-LRRLE-ADL	PI-VV--S---R---S----
	Frankia discariae	WP_018501457	--E-IA-LEGSGL	DISVV--VV--K---T----
	Geodermatophilus nigrescens	WP_073421961	--A-LERLEQSGL	PL-V---SV------Q----
	Glycomyces tenuis	WP_026929877	--E-IERLSASGI	TM-MT--AV-P---ES----
	Gordonia otitidis	WP_007240761	--E----LENSGH	P---VV-SV----D------
	Jatrophihabitans endophyticus	SHF99936	--E-HA-LGGSGL	PI-----S---R--EN----
	Kibdelosporangium aridum	SMC86515	--E-IQ-LADSGL	P---V--S---R---SGF--
	Lentzea albida	SEQ73824	--E-IN-LEDSKL	PC--V--SV--R---SGF--
	Micromonospora carbonacea	WP_074476743	--V-IRRLE-SGL	PL--V--S-------KMF--
	Nakamurella multipartita	WP_052308145	--E-LATLAAANL	P--VV--SV--R--EIG---
	Nocardia asiatica	WP_043720423	--E-IA-LTDSGL	P---V--VV-AE--EH----
	Pseudonocardia autotrophica	WP_073576390	--E--ERLRASGL	P--MV--S---R---S-F--
	Rhodococcus ruber	WP_003935717	--E----LS-SGH	P---V--S--D---EDK---
	Tsukamurella paurometabola	WP_013125653	--E--R-LTD-GH	P---V---V-R---QD----

Supplementary Figure 18

Detailed sequence information for the six amino acid insertion found in uracil phosphoribosyltransferase, which is shown in Figure 5A. This insertion is specific for members of the "*Abscessus-Chelonae*" clade and absent in other bacteria.

			94		130
“Abscessus-Chelonae” Clade (6/6)	*Mycobacterium abscessus*	WP_052620488	SEKTRMLLDALDPNT		FIPFDVDSGVLRAAGDALVAEY
	Mycobacterium abscessus subsp. bolletii	EHM21958	---------------		----------------------
	Mycobacterium chelonae	WP_070916846	-----------A-K-		----------------------
	Mycobacterium franklinii	WP_070937393	-----------N---		-------------------T--
	Mycobacterium immunogenum	WP_064631754	-----------N-G-		----------------------
	Mycobacterium saopaulense	WP_070912296	-----------G-S-		----------------------
Other *Mycobacterium* (0/>100)	*Mycobacterium acapulcensis*	WP_066810255	-----------RDRG	SLRR	-------AS--A---S--Q---
	Mycobacterium africanum	WP_003909980	-----------RHRG	SLRR	-V-----AS--S-TAT-IQR--
	Mycobacterium algericum	WP_083038822	-A---------QNTG	ALRR	-V-----A-I-ET--E-IARD-
	Mycobacterium alsense	WP_083141055	-----R-----RGRG	SLRG	-V-----A---S-TAA-VQH--
	Mycobacterium angelicum	WP_083115547	-----------RDRG	SLAR	-V-----AS--S-TAS-IQR--
	Mycobacterium aromaticivorans	WP_036341878	-----L--S-MRG-G	SLRR	-V------S--EM--A--EH--
	Mycobacterium arosiense	WP_083063925	-----L-----RARG	SLRR	-V-----ASI-ST-AA-IAR--
	Mycobacterium asiaticum	WP_065033936	-----L--N--RDSG	ALRR	-V-----AN--S--AE-IQR--
	Mycobacterium aurum	WP_087027963	-----L----MRDSG	ALKR	-------PS--HD-AA--RG--
	Mycobacterium austroafricanum	WP_036375127	-----A----MRDRG	TLRR	-------AS--KD--ASIE---
	Mycobacterium avium	WP_084026326	-----V-----RERG	ALRR	YV-----A-I-S-SAA-IQR--
	Mycobacterium avium subsp. hominissuis	KDP02737	-----V-----RERG	ALRR	YV-----A-I-S-SAA-IQR--
	Mycobacterium avium subsp. paratuberculosis	AAS02620	-----V-----RERG	ALRR	YV-----A-I-S-SAA-IQR--
	Mycobacterium avium subsp. silvaticum	ETB06252	-----V-----RERG	ALRR	YV-----A-I-S-SAT-IQRD-
	Mycobacterium bacteremicum	WP_083061503	-----L----FRDSG	ALRR	-------PS--D--AT--RS--
	Mycobacterium boenickei	WP_077738600	----------MRDGE	QLRR	-------A---------IGQ--
	Mycobacterium bohemicum	WP_085180024	-----------RDRG	SLRR	-V-----ATI-ST-AT-IQR--
	Mycobacterium branderi	WP_083134491	-----------RAGG	TLRR	-V-----ANM-SS-AA-IQR--
	Mycobacterium canariasense	WP_062660195	-----L--S-WRARG	LLRR	-------P---QD-AG--R---
	Mycobacterium canettii	WP_014001898	-----------RHRG	SLRR	-V-----AS--S-TAT-IQR--
	Mycobacterium celatum	WP_062541790	-----------RDGG	SLRR	-V-----ANM-SS-AA-IQR--
	Mycobacterium celeriflavum	WP_083000351	-----------RDSG	SLRR	-------V---T---SE-SI--
	Mycobacterium chimaera	WP_089151294	-----L-----RARG	SLRR	-V-----ASI-ST-AA-IAQ--
	Mycobacterium chlorophenolicum	W8472090	-----R----MAGRG	ALRR	-------AT--
	Mycobacterium chubuense	WP_014817989	-----R-----SDRG	ALVR	-------AS--AD--A-IQR--
	Mycobacterium colombiense	WP_064879996	-----V-----RARG	SLRR	-V-----ASI-ST-AA-IQR--
	Mycobacterium conceptionense	WP_064898415	----------MRDGG	QLRR	-------A-------A-IGQ--
	Mycobacterium confluentis	WP_085154450	-----R-----RAQG	TLRR	-V-----AS--AQ--AE-QRD-
	Mycobacterium conspicuum	WP_085232663	-----L-----RDRG	SLCG	-V-----A-M-S-TAT-IQG--
	Mycobacterium cosmeticum	WP_036397743	-----L--S-WRTRG	LLRR	-------P----D-AG--R---
	Mycobacterium diernhoferi	WP_073859278	-----L----MRD-G	SLKR	-------PS---D-AT--H---
	Mycobacterium doricum	WP_085191884	--------S--RDHG	SLRR	-V-----PT---SV-A--GI--
	Mycobacterium elephantis	WP_046754731	-----------RD-G	TLRR	-------A---N---A-IQK--
	Mycobacterium engbaekii	WP_085128691	-----------RDTG	TLQR	-V-----A---ET--E-IARD-
	Mycobacterium europaeum	WP_085241314	-----L-----RQRG	ALRR	-V-----A-I-ET-AA-IQR--
	Mycobacterium fallax	WP_085098293	-A-----I---RERG	TLDR	-V-V---AT--AQ-QRR-G-G-
	Mycobacterium farcinogenes	WP_036391222	----------MRDGG	QLRR	-------A-------A-IGQ--
	Mycobacterium flavescens	WP_069415360	-----------REHG	SLRR	-------AS--S---A-IA---
	Mycobacterium florentinum	WP_085224981	-----L-----HGRG	SLRR	-V-----ASI-ST-AA-IQE--
	Mycobacterium fortuitum	WP_061265065	----------MRDSG	QLRR	-------A---------IGQ--
	Mycobacterium fragae	WP_085194092	-----I--N--RDGG	SLRR	-V-----ASM-S--AT-IQR--
	Mycobacterium gastri	WP_036416518	--------N--RDRR	SLRR	-V-----A---S--AN-IKS--
	Mycobacterium genavense	WP_025736378	-----L-----HNRG	SLRR	-V-----ASI-ST-AA-IQH--
	Mycobacterium gilvum	WP_011892216	-----------RDRG	SLRR	--------S--T---A-IET--
	Mycobacterium goodii	WP_049748490	----------MRDAD	LLRR	-------A----S--A-IG---
	Mycobacterium gordonae	WP_065043849	-----------RERG	SLRR	-V-----AS--LETAA-IQH--
	Mycobacterium haemophilum	WP_047316212	-----L-----RDRG	SLRR	-V-----ASM-S--AT-IQH--
	Mycobacterium hassiacum	WP_005630877	-----------RDAG	TLRR	-------A-------A-IQT--
	Mycobacterium heckeshornense	WP_048891453	-A---------CERG	SLRR	-V-----ASM--S-AV-VQ---
	Mycobacterium heidelbergense	WP_083074862	-----R-----RDRG	SLRG	-V-----A-M-S-TAT-IQR--
	Mycobacterium heraklionense	WP_064891125	-----------QDAG	TLRR	-------A---ET--E-ISGD-
	Mycobacterium hiberniae	WP_085134109	-----------RD-R	TLQR	-V-----A---EK--E-IARD-
	Mycobacterium holsaticum	WP_069407412	-----------RD-G	TLRR	-------A---S---T-IQH--
	Mycobacterium houstonense	WP_066902270	----------MRDTG	QLRR	-------A-------A-IGQ--
	Mycobacterium icosiumassiliensis	WP_067976608	-----------QDAG	LLRR	-------A---ET--E-IARD-
	Mycobacterium insubricum	WP_083030801	A----L-----RDRG	SLHR	YV-----AAM-ADV-A-VA---
	Mycobacterium interjectum	WP_066916720	-----R-----RDRG	SLRG	-V-----AS--S-TAA-IQR--
	Mycobacterium intermedium	WP_069417958	-----Q-----RDRE	SLRK	-V-----ANM-SESAA-IQR--

Group	Species	Accession	Sequence
**Other *Mycobacterium* (0/>100)**	*Mycobacterium intracellulare*	WP_064935036	-----L-----RARG SLRR -V-----ASI-ST-AA-IAR--
	Mycobacterium iranicum	WP_064283230	-----------RDGG SLRR --------S------A-IQN--
	Mycobacterium kansasii	WP_063468646	--------N--RDRG SLQR -V-----A---S--AN-IKS--
	Mycobacterium komanii	CRL73953	-----L-----HD-G ALRR -------AS--T---A--QD--
	Mycobacterium koreense	WP_085305342	-----L--A--HRSG RLRR -V-----A---QS-AAGIE--F
	Mycobacterium kubicae	WP_085074707	-----------RGQG SLRR -V-----AN--S--AN-IQR--
	Mycobacterium kumamotonense	WP_065289550	-A---------QATG SLRR -V-----A---ET--E-IARD-
	Mycobacterium kyorinense	WP_065012855	-----------RDRG ALRR YV-----ANM-SV-AS-VQR--
	Mycobacterium lacus	WP_085159007	-----------RDRG SLRR -V-----A-M-S-TAS-VQR--
	Mycobacterium lentiflavum	CQD22085	-----L-----HGCG SLRR -V------TI-ST-AA-IQQ--
	Mycobacterium liflandii	WP_015357532	-----L--N--RDRG LLQR -V-----ES--S--AS-IQS--
	Mycobacterium litorale	WP_078021270	-----L--T-MRD-G SLRR -----------ES--A--STD-
	Mycobacterium llatzerense	WP_071286503	-----I----FRDAA ALRR -VA----EN--S--LTQ-SD--
	Mycobacterium longobardum	WP_085265499	-----------QDTG SLRR -------A---EM--E-IARD-
	Mycobacterium mageritense	WP_036431695	----------MRDGG QLRR -------A---Q---K-IGR--
	Mycobacterium malmesburyense	RL72670	-----------RDRG SLRR -------
	Mycobacterium malmoense	WP_065446358	-----L-----RDRG ALRR -V-----A-I-ST-AA-IQR--
	Mycobacterium mantenii	P_O83095549	-----L-----RARG SLRR -V-----
	Mycobacterium marinum	WP_012396724	-----L--N--RDRG LLQR -V-----ES--S--AS-IQS--
	Mycobacterium marseillense	WP_083016554	-----L-----RASG SLRR -V-----ASI-ST-AA-IQQ--
	Mycobacterium minnesotense	WP_083025541	-----------RDAG ALRR -------A---ET--E-IARD-
	Mycobacterium moriokaense	WP_083154996	-----------RDSG SLRR -------AS------A-IEQ--
	Mycobacterium mucogenicum	WP_064857718	-----I----FRDAA ALRR -VA----EN--S--LTQ-SD--
	Mycobacterium nebraskense	WP_046184327	-----L-----RERG SLRR -V-----A-I-ST-AA-IQR--
	Mycobacterium neoaurum	WP_030132773	-----L----FRARG VLRR -------PS--G--AT--HT--
	Mycobacterium neworleansense	CRZ17722	----------MRDGG QLRR -------A-------A-IGQ--
	Mycobacterium nonchromogenicum	WP_085138292	-----------QEVG ALRR -------A---ET--E-IARA-
	Mycobacterium noviomagense	WP_083089101	--------A--REHG SLRR -V-----AS--Q--ATGIQHD-
	Mycobacterium novocastrense	P_O67388753	-----------RDHG SLRR -------
	Mycobacterium obuense	WP_048422569	-----R----MRERG ALRR -------AT--E---A-ISD--
	Mycobacterium palustre	WP_085076711	-----R-----RERG SLRG -V-----A---S-TAA-VQR--
	Mycobacterium paraense	WP_085094952	-----R-----RDRG SLRG -V-----AS--S-TAT-IQR--
	Mycobacterium paraffinicum	WP_073871663	-----L-----RERG SLRR -V-----A-I-ST-AA-IQR--
	Mycobacterium parafortuitum	WP_083142936	-----------SERG SLRR -V------S--Q---A-IEQ--
	Mycobacterium paraseoulense	WP_083173506	-----L-----RNRG SLRR -V-----A-I-ST-AA-IQR--
	Mycobacterium parmense	WP_085267122	-----L-----RDRG SLRR -V-----ASI-SS-AT-IQQ--
	Mycobacterium peregrinum	WP_064878749	----------MRDGG QLRR -------AS---S----IGQ--
	Mycobacterium persicum	WP_083156010	--------N--RDRG SLRR -V---------S--AN-IKG--
	Mycobacterium phlei	WP_061481379	--------A--HDRG ALRR -------AT--T---S-IA---
	Mycobacterium porcinum	WP_069428094	----------MRDGG QLRR -------A---------IGQ--
	Mycobacterium rhodesiae	WP_083120873	-----L--S-MRD-G SLRR -V------S--EL--A--EQ--
	Mycobacterium riyadhense	WP_085251753	-----------RDRG SLRR -V-----AS--S-TAG-IQR--
	Mycobacterium rufum	KGI71186	-----R----MAGRG ALRR -------A---E---A-IES--
	Mycobacterium rutilum	WP_083406890	-----------HEHG SLRR -------A---S---T-IAD--
	Mycobacterium saskatchewanense	WP_085258255	-----------RDRG RLRR -V-----AT--ST-AT-IQR--
	Mycobacterium scrofulaceum	WP_067270891	-----L-----RERG QLRR -V-----A-I-SS-AA-IQR--
	Mycobacterium senuense	WP_085085565	-A---------RATG SLRR -V-----A---ET--E-IARD-
	Mycobacterium septicum	WP_044521237	----------MRDGG QLRR -------AS--------IGQ--
	Mycobacterium setense	WP_064875154	----------MRDGG QLRR -------AS------A-IGR--
	Mycobacterium sherrisii	WP_069401940	-----L-----HQRA ALRR -V-----A-M-AT-AT-IQQ--
	Mycobacterium shigaense	BAX94817	-----L---S-HTRG SLRR -V-----ETM-S---A-IQH--
	Mycobacterium shimoidei	WP_069397218	-----L--E--QAAG SLRR -V-----ASM-SS-AT-IKRD-
	Mycobacterium shinjukuense	WP_083052086	--------N--RDRG SLCG -V-----AN--S-TAA-IQ---
	Mycobacterium simiae	WP_061557476	-----L-----HRRA ALRR -V-----A-I-AT-AA-IQQ--
	Mycobacterium sinense	WP_064855725	-----------HA-G SLRR -------A---ET--A-IAGD-
	Mycobacterium smegmatis	WP_003897669	----------MRDAE LLRR -------A----S--A-IG---
	Mycobacterium szulgai	WP_068034372	-----------RGQG SLRR -V-----AN--S--AN-IQR--
	Mycobacterium terrae	WP_085259931	-V---------QATG ALRR -V-----A---ET--E-IARD-
	Mycobacterium thermoresistibile	WP_003925247	-----L-----HTRG VLRR -V-----A----SS-A-IA---
	Mycobacterium triplex	WP_036465202	-----L-----HNRG SLRR -V-----ASI-ST-AA-IQH--
	Mycobacterium triviale	WP_069391689	-----L--A--HRSG RLRR -V-----A---QS-AAGIE--F
	Mycobacterium tuberculosis	WP_070897718	-----------RHRG SLRR -V-----AS--S-TAT-IQR--
	Mycobacterium tusciae	WP_083126296	-----------RD-G SLRR -------AS--K---S-IEE--
	Mycobacterium ulcerans	WP_011741906	-----L--N--RDRG LLQR -V-----ES--S--VS-IQS--
	Mycobacterium vaccae	WP_003931851	-----T-----RDRG SLRR -V-----PS--QD--A-IQ---

Group	Organism	Accession	Sequence
**Other *Mycobacterium* (0/>100)**	*Mycobacterium vanbaalenii*	WP_011782607	-----A----MRDRG TLRR -------AS--KD--ASIE---
	Mycobacterium vulneris	WP_065462875	---------GMRDGG QLRR -------A---------IGQ--
	Mycobacterium wolinskyi	WP_085143091	----------MRAGG SLRR -------A---------IGR--
	Mycobacterium xenopi	WP_003921580	-A---------CEQG SLRR -V-----ASM-QS-AT-VQ---
**Other bacteria**	*Actinoalloteichus cyanogriseus*	WP_026418763	---L----MTALG DLRR YV-V-ISMSA--QSSTEIA-D-
	Actinoplanes rectilineatus	WP_045743149	---L-----TARG TLGG -V-L--SADA-GD-VTV-GES-
	Amycolatopsis balhimycina	WP_020638329	---L-----TGHG TLEA -V-L--SESA-AD-AE-ISRD-
	Geodermatophilus poikilotrophus	SET90336	---L-----RGAG TLRR -V-C---PS--Q---A-IT---
	Kitasatospora mediocidica	WP_035796272	---F---G-HALG TLES YV-V--SESA-QE--EQ--K--
	Lentzea guizhouensis	WP_065917564	---L--S--REHG TLTQ -V-Q--SVSA-TE-AH-IM-D-
	Micromonospora coriariae	SCF11030	---L----FTREG NLGT -V-L--SVSA--GSTAEIA-D-
	Myxococcus stipitatus	WP_015353632	---L----MEEAG QLSR -V----SEVF--R-AAS-AR--
	Nocardia arthritidis	WP_063052577	---L--T--SARG PLKT YV-Q--S-AA----A-EVA--F
	Pseudonocardia ammonioxydans	SFN63176	---L----FTAAG TLRR YV-Q--SESA--G-M-E-HEA-
	Saccharomonospora saliphila	WP_019812602	---A---G-REHA TLRQ -V-L--SASA-SE-AETIARD-
	Streptacidiphilus anmyonensis	WP_042420914	---L-----TEAG TLSC YA-L--SPSA-EQ--S---RD-
	Streptomyces aureofaciens	WP_033348364	---L-----RSLD TLED YV-V--SESA-TS--A--A---
	Xiangella phaseoli	SEJ43738	---L----FTQHG DLGT -V-L--SVSA-EGSTA--A-D-

Supplementary Figure 19

Detailed sequence information for the four amino acid deletion found in L-histidine N(alpha)-methyltransferase, which is shown in Figure 5B. This deletion is specific for members of the "*Abscessus-Chelonae"* clade and absent in other bacteria.

			364		407
**"Abscessus-Chelonae" Clade (7/7)**	*Mycobacterium abscessus*	WP_074245867	LLVAAVSDPRVEDMVTP	ANATGPGNIR	SLDAEQVYDAAAAERTR
	Mycobacterium abscessus subsp. bolletii	SKL48271	---------------M-	E-S-------	---V-------------
	Mycobacterium chelonae	WP_070916831	---------------M-	E-S-------	T--V-------------
	Mycobacterium franklinii	WP_078334786	--------------AM-	P-S-------	---V-------------
	Mycobacterium immunogenum	WP_064631781	------------E-AM-	E-S-------	---V-------------
	Mycobacterium salmoniphilum	WP_078330499	-----------D---M-	E---A-----	-----------------
	Mycobacterium saopaulense	WP_083015045	------------M-	EKS-------	---V-------------
**Other *Mycobacterium* (0/85)**	*Mycobacterium algericum*	WP_083040572	VIL---A-----RLARG		RT--AA----G----A-
	Mycobacterium alsense	WP_083141095	VM-G--A----DQ-AAG		RS--AA---------S-
	Mycobacterium angelicum	WP_083115555	V-----G----DQLAAG		RS--AA---------S-
	Mycobacterium aromaticivorans	WP_036341860	VI---------DELAAG		RA--A----------A-
	Mycobacterium arosiense	WP_083063933	--L--------DQ-AAG		RS--AA---------S-
	Mycobacterium austroafricanum	WP_036375155	VI----A----DTLAAG		RA--VE---------A-
	Mycobacterium avium	WP_062906431	------A----DQ-AAG		RS--AA---------S-
	Mycobacterium avium subsp. avium 2285 (R)	EUA41931	------A----DQ-AAG		RS--AA---------S-
	Mycobacterium avium subsp. paratuberculosis	ETB35959	------A----DQ-AAG		RS--AA----V----S-
	Mycobacterium bohemicum	WP_085180031	------G----DQ-AAG		RS--AA--------KS-
	Mycobacterium bovis	WP_080655150	V-----A----DQLAAG		RS--AA---------A-
	Mycobacterium branderi	WP_083134504	VIL---A----DQLAIG		RV--PA---------A-
	Mycobacterium brisbanense	WP_062828382	V-L---A-----RLAAG		RD--A----------A-
	Mycobacterium canettii	WP_014001891	V-----A----DQLAAG		RS--AA---------A-
	Mycobacterium celatum	WP_085168538	VIL---A----DQLAIG		RA--PA---------A-
	Mycobacterium celeriflavum	WP_083000336	VIL---A-----VLAAG		RA--P----------A-
	Mycobacterium chimaera	WP_072501142	--L--------DQ-AAG		RS--AA---------S-
	Mycobacterium chlorophenolicum	WP_048472072	V-L--------DALAAG		RA--VE---------A-
	Mycobacterium chubuense	WP_014817970	V----------DRLA-G		RT--VA---------A-
	Mycobacterium colombiense	WP_064879978	--L--------DQ-AAG		RS--AA---------S-
	Mycobacterium confluentis	WP_085150316	VI----------ELAEG		RS--A-I--------S-
	Mycobacterium conspicuum	WP_085232680	VMI---A----DQLAAA		RS-PAA---------A-
	Mycobacterium europaeum	WP_085241330	-M----A----DH-AAG		RS-PAA---------S-
	Mycobacterium flavescens	WP_069415350	V-I---A----A-LAAG		RA--P---N-G----S-
	Mycobacterium florentinum	WP_085224999	-I---------DQ-AAG		RS--AA---------S-
	Mycobacterium fortuitum	WP_064848743	V-L--------DQLAAG		RS--P----------A-
	Mycobacterium fragae	WP_085194123	VIL---A----DQ-AVG		RA--AA---------S-
	Mycobacterium gastri	WP_036413622	VV----A----DQLAIG		RS--AA---------A-
	Mycobacterium genavense	WP_025736369	------A----DQ-AAG		RA--AA---------S-
	Mycobacterium gilvum	WP_011892234	VI----A-----KLAAG		RA--VE---------A-
	Mycobacterium gordonae	WP_065045787	V----------DELAAG		RS--AA---------S-
	Mycobacterium haemophilum	WP_054879185	VMI---T----DH-AAG		RS--AA---------A-
	Mycobacterium hassiacum	WP_005630860	V-L---A----DQLAAG		RA--P------------
	Mycobacterium heidelbergense	WP_083077169	------A----DH-AAG		RT--AA---------S-
	Mycobacterium hiberniae	WP_085134102	VIL---A-----RLAHG		RG--AA----G----A-
	Mycobacterium icosiumassiliensis	WP_067976589	VIL---A-----RLADG		RG--AA----G----A-
	Mycobacterium insubricum	WP_083033466	VI----A-----TLAAG		RG--A----------S-
	Mycobacterium interjectum	WP_066916704	VMI---G----DQLAAG		LS--AA---------S-
	Mycobacterium intermedium	WP_083148552	V-L---A----DELAVG		RS--AA---------A-
	Mycobacterium intracellulare	WP_064892783	--L--------DQ-AAG		RS--AA---------S-
	Mycobacterium iranicum	WP_024446978	VI--S-A----DKLAAG		RA--VE---------S-
	Mycobacterium kansasii	WP_063472998	VV----A----DELAIG		RS--AA---------A-
	Mycobacterium komanii	CRL73993	V-----A----DTLAAG		RA--P----------A-
	Mycobacterium kumamotonense	WP_065289626	VIL---A-----RLARG		RG--AA----G----S-
	Mycobacterium lacus	WP_085158969	V-----A----DQLAAG		RS--AA---------A-
	Mycobacterium lentiflavum	CQD22066	-----------DQ-A-G		RS--AA---------S-
	Mycobacterium litorale	WP_078021257	VI----A----DELAAG		RA-SA----------A-
	Mycobacterium longobardum	WP_085265494	VIL---A-----RLARG		RA--AA----G----S-
	Mycobacterium mageritense	WP_036431773	V-----A----DALAAG		RA--AR------S--A-
	Mycobacterium malmesburyense	CRL72692	V--S--A---IDTLAAG		RA--P----------A-
	Mycobacterium malmoense	WP_065444484	-M----A----DHLASG		RA-PAA---------S-
	Mycobacterium mantenii	WP_083095541	--L--------DQ-AAG		RS--AA---------S-
	Mycobacterium marseillense	WP_083016538	--L--------DQ-AAG		RS--AA---------S-
	Mycobacterium neworleansense	CRZ17776	V-L--------DQLAAG		RS--A----------A-
	Mycobacterium obuense	WP_048422589	V-L--------DALAAG		RA--VE---------A-
	Mycobacterium palustre	WP_085076703	M-----A----DE-AAG		RS--AA---------S-
	Mycobacterium paraense	WP_085094930	VMI---G----DQ-AAG		RS--AA---------S-

Other *Mycobacterium* (0/85)	*Mycobacterium paraffinicum*	WP_073871638	------A----DQ-AAG	RS-PAA---------S-
	Mycobacterium parafortuitum	WP_083142952	V---------DTLAAG	RA--VE---------A-
	Mycobacterium persicum	WP_083155111	VV----A----DELAIG	RS--AA---------A-
	Mycobacterium porcinum	WP_075924435	V-L--------DQLAAG	RS--A----------A-
	Mycobacterium rhodesiae	WP_083120857	VI---------DELAAG	RA--A----------A-
	Mycobacterium riyadhense	WP_085251763	V-----A----DQLAIG	RS--AA---------A-
	Mycobacterium rufum	KGI70125	V-L--------DTLAAG	RA--VE---------A-
	Mycobacterium rutilum	WP_083406907	V-I---A----AALAAG	RA--A-----G------
	Mycobacterium saskatchewanense	WP_085258245	------A----DQ-AAG	RS--AA--------KS-
	Mycobacterium scrofulaceum	WP_067270867	-M----A----DQ-AAG	RS-PAA---------S-
	Mycobacterium sherrisii	WP_085167093	--I--A-----DQ-A-G	RS--AA----G---KS-
	Mycobacterium shigaense	BAX94809	VML---A----DQ-AAG	RS--AA---------S-
	Mycobacterium shimoidei	WP_069397225	VII---A----DQLA-G	RS--PA---------S-
	Mycobacterium shinjukuense	WP_083048384	V-----A----DQLAAG	RS--AA---------A-
	Mycobacterium simiae	WP_061557469	--I--A-----DQ-A-G	RS--AA----G---KS-
	Mycobacterium sinense	WP_013830893	VIL---A-----RLARG	RT--AA----G----A-
	Mycobacterium szulgai	WP_068156425	V-----A----DELALG	RS--AA---------A-
	Mycobacterium thermoresistibile	WP_003925238	V-L---A----DQLAAG	RA--A------S---S-
	Mycobacterium triplex	WP_036465190	------A----DQ-AAG	RA--AA---------S-
	Mycobacterium triviale	WP_085111432	VIL---A----ARLAAG	RT--AA---------S-
	Mycobacterium tuberculosis	WP_070895792	V-----A----DQLAAG	RS--AA---------A-
	Mycobacterium ulcerans str. Harvey	EUA86602	VV----A----DQLAIG	RS--AA---------A-
	Mycobacterium vaccae	WP_003931894	VI---------DKLAAG	RA-PVE---------A-
	Mycobacterium vanbaalenii	WP_011782581	VI----A----DTLAAG	RA--VE---------A-
	Mycobacterium vulneris	WP_085290716	-----------DQ-AAG	RS--AA---------S-
	Mycobacterium wolinskyi	WP_067850143	V-L--------DQLAAG	RS--A----------S-
	Mycobacterium xenopi	WP_003921589	VI----A----DQLASG	RS--AA---------A-
	Mycobacterium yongonense	WP_020821434	--L--------DQ-AAG	RS--AA---------S-
Other bacteria	*Actinomadura chibensis*	WP_067905717	VMI--------GE-AAG	RG-LAS---------A-
	Amycolatopsis balhimycina	WP_020643641	-I--S-A----AE-LAG	RE---A-----S-S--V
	Brachybacterium massiliense	WP_087483494	-V--S-A--AL-QLAAG	RG-T-A--Q-----QA-
	Cellulomonas bogoriensis	WP_035060000	VVL-S-A--E-AELRAR	RG-VAE-F--------E
	Frankia elaeagni	WP_018635354	V----LR---LDELA-G	HD-VG---A-----Q-L
	Gordonia aichiensis	WP_005178254	VVIGS----LLDE-AQR	RD--REI------S--L
	Herbidospora cretacea	WP_061299375	V-L---A----AALAAG	RDGP-E-F-------QG
	Hoyosella subflava	WP_013807470	VIF-S-A--S-QV-SRA	RGSSDD--T-------I
	Kitasatospora mediocidica	WP_035793381	VV----A--HL-TLAAG	RGTLPA--G-----Q--
	Lentzea albida	SES38765	--I-S-A--A-HA-AAG	RG-L-A--S-------L
	Nocardiopsis gilva	WP_017618748	------G----TE-AAA	RG--RG--E-------L
	Planomonospora sphaerica	WP_068894570	V-L--------AA-ARG	RDTL----------HL-
	Segniliparus rotundus	WP_013138912	V-L---A---IAELAAG	RA------G-----LM-
	Streptomyces aureofaciens	WP_033350791	VVL-S-A--HLDVLAAG	RGTV-A--S--------
	Streptosporangium roseum	WP_012888146	V-L------Q-AV-AAG	RGTP-L--N-----HQN
	Tetrasphaera japonica	WP_048551988	VV--S-A-SA-AR-RGD	RSGVA-----------T
	Thermobifida halotolerans	WP_068692121	V-----A----TE-AAE	RG-TRA--N----Q--I
	Thermomonospora chromogena	SDQ89496	V-L-S-A----AA-ARG	RGTP-R--------HLN
	Yuhushiella deserti	SFQ17127	------A----ASLAHA	RG---A--S-------L

Supplementary Figure 20

A partial sequence alignment of a conserved region of DUF58 domain-containing protein showing a 10 amino acid insertion that is specific for members of the *"Abscessus-Chelonae"* clade and absent in other bacteria.

Group	Species	Accession	724		762
**"Abscessus-Chelonae" Clade (5/5)**	*Mycobacterium abscessus*	WP_062878914	TANEIGAAEGESVTV	RSLS	ESIRGAITLPLRITEMPDRV
	Mycobacterium chelonae	WP_070916166	--S-----------I	----	---Q-S--------------
	Mycobacterium franklinii	WP_070937060	-------T---L---	--M-	---P-S-----L--------
	Mycobacterium immunogenum	WP_064632555	---------------	----	---G-S-------A------
	Mycobacterium saopaulense	WP_070910881	---------------	--M-	---P----------------
**Other *Mycobacterium* (0/81)**	*Mycobacterium aromaticivorans*	WP_036339931	--AG---VD--V-S-		STG--D-A---A----A-G-
	Mycobacterium arupense	WP_046188477	--A------AD----		STA--R-----S--D---N-
	Mycobacterium asiaticum	WP_065036467	--A----GH-DP---		STD--S-----SV-D-----
	Mycobacterium aurum	WP_048634293	--A----GD-DLL--		STD-------VEV-D-G---
	Mycobacterium avium	WP_062895015	--A-----D-DA---		STP--S-S---V--D-----
	Mycobacterium avium subsp. avium 10-9275	ETB20634	--A-----D-DA---		STP--S-S---V--D-----
	Mycobacterium avium subsp. hominissuis	KDO94172	--A-----D-DA---		STP--S-S---V--D-----
	Mycobacterium avium subsp. paratuberculosis	AGL35540	--A-----D-DA---		STP--S-S---V--D-----
	Mycobacterium avium subsp. silvaticum	ETB14382	--A-----D-DA---		STP--S-S---V--D-----
	Mycobacterium bohemicum DSM 44277	CPR13304	--A----GD-DA---		STD--S-----CV-D-----
	Mycobacterium bovis	WP_049950214	--A-----D--A---		STS--S----CSV-D-----
	Mycobacterium brisbanense	WP_062827114	--A-----Q-DP---		RTD---V----AV-A-A---
	Mycobacterium canariasense	WP_062657303	--A-T-CSDTVR-ST		D--------E--D-----
	Mycobacterium canettii	WP_014001595	--A-----D--A---		STS--S----CSV-D-----
	Mycobacterium caprae	WP_054938676	--A-----D--A---		STS--S----CSV-D-----
	Mycobacterium celatum	WP_062539375	--A-------DP---		STA--------AV-D---H-
	Mycobacterium chlorophenolicum	WP_048472775	--A--D--A-DP---		STE--------AV-D-A---
	Mycobacterium chubuense	WP_014814808	--AG--VGD--M-S-		RTD--V-----DV---D-G-
	Mycobacterium colombiense	WP_007772887	--A-----D-DAI--		STSH-S-S---M--D-----
	Mycobacterium conceptionense	CQD04312	--A----TP-DL-D-		STD--TV----AV---A---
	Mycobacterium cosmeticum	CDO05840	--R---VG---H-SI		STVK----A-VH----T---
	Mycobacterium elephantis	KKW65728	--A---VG--DL---		RTE--------A--D-D---
	Mycobacterium europaeum	CQD20284	--A-----D-DA---		STP--S-S---V--D-----
	Mycobacterium farcinogenes	WP_036388640	--A----TP-DL-D-		STD--TV----AV---A---
	Mycobacterium fortuitum	WP_061264116	--A-L---A-DP---		STES-------SVA------
	Mycobacterium gastri	WP_036411977	--A-----D-DD---		STS--S--V--CV-D-----
	Mycobacterium genavense	WP_025735672	--T----S--DT---		STS--S-S---N--D-----
	Mycobacterium gilvum	WP_011895330	--AD---RG-DV---		STD--------TV-D-A-G-
	Mycobacterium goodii	WP_049747699	--AD--TRD-AP---		STE--------AV-D-----
	Mycobacterium gordonae	WP_065043453	--A-----D------		STD--S-----NV-D-----
	Mycobacterium haemophilum	WP_054880440	S-A-----D-DA---		TTS--S-----T--D-----
	Mycobacterium hassiacum DSM 44199	EKF24642	--AG----D-DP---		RTG--------K--D---GT
	Mycobacterium heckeshornense	WP_048891318	S-A-----D-DL---		STE--------TV-D---H-
	Mycobacterium heraklionense	WP_064888086	--AD--V----P---		STEH-S-----V------N-
	Mycobacterium holsaticum	WP_069405786	--A----G--DP---		RTD--------A--D-A---
	Mycobacterium interjectum	WP_066908709	--A--A--D-DN---		STP--S-S---N--D-----
	Mycobacterium intermedium	WP_069419220	--A--N--D-DE---		STQ--S-----K--DL----
	Mycobacterium intracellulare	WP_064936970	--A-------DA---		STP--S-S---LV-D-----
	Mycobacterium iranicum	WP_024444727	--A---V-A--L-S-		STD--------EV-D-D---
	Mycobacterium kansasii	KZS58870	--A-----D-DD---		STS--S--V--CV-D-----
	Mycobacterium kyorinense	WP_065016514	--A-----D-DP---		STP--SVS---AV-D-----
	Mycobacterium lentiflavum	CQD22953	--A--N--A-DL-SI		STG--T-----ALADL-H--
	Mycobacterium liflandii	WP_015354937	--A-----D-DA---		S-S--S-----N--D--E--
	Mycobacterium llatzerense	WP_043987722	--AS---G-TLT-ST		D--S-----E--D--TG-
	Mycobacterium mageritense	WP_036434128	--T----GP-DA---		RTE--------VV-D-V-G-
	Mycobacterium malmoense	WP_065443959	--A----T--DA---		STP--S-S---VV-D-----
	Mycobacterium marinum	WP_012393321	--A-----D-DA---		S-S--S-----N--D--E--
	Mycobacterium microti	AMC60900	--A-----D--A---		STS--S----CSV-D-----
	Mycobacterium mungi	WP_064319767	--A-----D--A---		STS--S----CSV-D-----
	Mycobacterium nebraskense	WP_046186667	--A-----D-DA--I		STP--S-S---I--D-----
	Mycobacterium neoaurum	WP_030135954	--A---C---DP-RI		STD--TVD--VA--------
	Mycobacterium neworleansense	CRZ15496	--A-LDV-P-D----		STD--TV----EMA------
	Mycobacterium obuense	KKF00617	--A--N-EP-DP---		STE--S-----AV---A---
	Mycobacterium orygis	WP_003416441	--A-----D--A---		STS--S----CSV-D-----
	Mycobacterium parascrofulaceum ATCC BAA-	EFG75829	--A----T--DA---		STP--S-S---VV-D-----
	Mycobacterium peregrinum	WP_064878920	--A---V-A-DP---		STD--SV----SV-------
	Mycobacterium phlei	WP_003888733	--A-----DRDL---		RTP--TV----AV-D-A---
	Mycobacterium porcinum	WP_069427500	--A-L-V-SDDP---		STD---V----AV-------
	Mycobacterium pseudoshottsii L15	GAQ39683	--A-----D-DA---		S-S--S-----N--D--E--

**Other *Mycobacterium* (0/81)**	*Mycobacterium rhodesiae*	WP_014208714	--GQ----DDDL---	RTE--EV----L--D---GT
	Mycobacterium rufum	KGI67482	--A----GA-DP---	STD--------AV-D-A---
	Mycobacterium rutilum	SEH89748	--A-------DL---	RTP--S-----V--DIG---
	Mycobacterium scrofulaceum	WP_067271421	--A-----D-DA---	STP--S-S---V--D-----
	Mycobacterium septicum	WP_044516950	--T-L-VTP-DP---	STD--SV----S-A------
	Mycobacterium setense	WP_064871766	--T----TA--P---	STE--SV----AV---A---
	Mycobacterium sherrisii	WP_069400203	--A----V--DM---	STP--S-R---N--D-----
	Mycobacterium shimoidei	WP_069396513	--S-----D-DA---	STS--------AV-D-----
	Mycobacterium sinense	WP_064854762	--T-----P-DP---	STEQ-D-----V--------
	Mycobacterium smegmatis	WP_011728121	--AG---SD-AP---	STE---V----AV-D---G-
	Mycobacterium szulgai	WP_068026433	--A-----D-DE---	STS--S-----EV-DL----
	Mycobacterium thermoresistibile	WP_003927346	--AD----D--P-A-	GTD--E-V---AV---A---
	Mycobacterium triplex	WP_036467520	--T----S--DT---	STS--S-S---N--D-----
	Mycobacterium triviale	WP_069393741	--A-----D--Q---	ATE--------CV-A--P--
	Mycobacterium tuberculosis	WP_070890035	--A-----D--A---	STS--S----CSV-D-----
	Mycobacterium tusciae	WP_006242848	--C--S-TP-DL---	STD----S---A--------
	Mycobacterium ulcerans Agy99	ABL04813	--A-----D-DA---	S-S--S-----N--D--E--
	Mycobacterium vaccae	WP_003928982	--A-V-V-D--LL--	STD----S---QV-D-D---
	Mycobacterium vanbaalenii	WP_011779118	--A-V-V-G--PL--	STD---V----ELAD-D---
	Mycobacterium vulneris	WP_065461164	--A-L-VTSDDP---	STD---V----AV-------
	Mycobacterium wolinskyi	WP_067856624	--A-----D-DP---	STD--------A--D-A---
	Mycobacterium xenopi	WP_050947710	S-A-------DL---	STE--------TV-D---H-
**Other bacteria**	*Actinoalloteichus cyanogriseus*	WP_026419564	-GERL-L-V-DP---	STD--SV---AELADL--D-
	Amycolatopsis rubida	SFP38659	--D-F-IQP-DP---	STD-------TTTADL----
	Geodermatophilus nigrescens	SHG79275	A-ARL-L-D-DPL--	TGAT-SV---VLV-----GI
	Gordonia amarae	WP_005184257	--G----LPAGR---	STLY---S---ELDDL----
	Micromonospora avicenniae	WP_076469142	--EAL-V-D-DA---	GTD---V---AA------G-
	Nocardia crassostreae	WP_067538593	--A-----T-DP---	SNDH-Q-----V-----Q--
	Pseudonocardia ammonioxydans	SFM56914	--ERL-LV---DAA-	GTD--T----VALADL--G-
	Rhodococcus jostii	SEE41578	S-A-T--GD-DP---	STD---V----V--D-----
	Salinispora pacifica	WP_029130328	--ES--V-DSDA---	GTE--GV---VVV-----G-
	Streptomyces celluloflavus	WP_052856194	--R-T-VKD-DLLA-	AGPA--VQ---Q--P-----
	Tetrasphaera japonica T1-X7	CCH80327	--EAL-V-D-DA---	STDA-S--A-VAV-D-V-H-
	Verrucosispora sediminis	SFC80123	S-EAL-V-D-DP---	GTD---V---AAT-----G-
	Xiangella phaseoli	SEJ67096	--EAL-V-D-DP---	GTD---L---AA------G-
	Xylanimonas cellulosilytica	WP_012877258	--AAVDVFD-DQ---	STE--SL---VVV---V-H-

Supplementary Figure 21

A partial sequence alignment of a conserved region of NADH-quinone oxidoreductase subunit G showing a four amino acid insertion that is specific for members of the "*Abscessus-Chelonae"* clade and absent in other bacteria.

			169		208
**"*Abscessus-Chelonae*" Clade (5/5)**	*Mycobacterium abscessus*	WP_052624897	DEMLDLGFLPDIERILALAP	SAD	AGRQSMLFSATMPDPII
	Mycobacterium chelonae	WP_070920767	--------------------	--E	S-------------A--
	Mycobacterium franklinii	WP_070938733	--------------------	--E	S-------------A--
	Mycobacterium immunogenum	WP_064627986	--------------------	---	-----------------
	Mycobacterium saopaulense	WP_070909589	--------------------	---	-----------------
**Other *Mycobacterium* (0/>100)**	*Mycobacterium africanum*	WP_031668570	----------------RQI-		-D---------------
	Mycobacterium algericum	WP_083038590	----QM--AE-V-----DT-		EYK-VA-------PA-
	Mycobacterium alsense	WP_083138608	----------------RQI-		-D-----------G---
	Mycobacterium angelicum	WP_083111091	----------------RQI-		-D---------------
	Mycobacterium aromaticivorans	WP_036340128	----------------KQI-		TE--A------------
	Mycobacterium arosiense	WP_083065563	----------------RQI-		TD---------------
	Mycobacterium arupense	WP_046686367	----------------SRI-		DD-----------G---
	Mycobacterium asiaticum	WP_065035329	----------------RQI-		-D---------------
	Mycobacterium aurum	WP_048630372	----------------KQI-		-K--A------------
	Mycobacterium austroafricanum	WP_036369449	----------------KQI-		-K--A------------
	Mycobacterium avium	WP_073578897	----------------RQI-		DD---------------
	Mycobacterium avium subsp. avium	EUA38782	----------------RQI-		DD---------------
	Mycobacterium avium subsp. hominissuis	KDP03616	----------------RQI-		DD---------------
	Mycobacterium avium subsp. paratuberculosis	ELP45005	----------------RQI-		DD---------------
	Mycobacterium bacteremicum	WP_083057322	----------------R-T-		DD-----------G---
	Mycobacterium boenickei	WP_077743098	----TM--AEEV-----DT-		EYK-VA-------PA-
	Mycobacterium bohemicum	WP_085182017	----------------RQI-		ED-----------G---
	Mycobacterium bovis	AMC56664	----------------RQI-		-D---------------
	Mycobacterium branderi	WP_083129881	----------------RQI-		DD---------------
	Mycobacterium canariasense	WP_062654506	----------------R-T-		DS--A------------
	Mycobacterium canettii	WP_015291283	----------------RQI-		TD---------------
	Mycobacterium caprae	WP_075744585	----------------RQI-		-D---------------
	Mycobacterium celatum	WP_085167763	----------------RQI-		DD--A------------
	Mycobacterium chimaera	WP_074020982	----------------RQI-		VD---------------
	Mycobacterium chlorophenolicum	WP_048471362	----------------KQI-		EQ--A------------
	Mycobacterium chubuense	WP_014814688	----------------KQI-		-Q--A------------
	Mycobacterium colombiense	WP_064877099	----------------RQI-		TD---------------
	Mycobacterium conceptionense	CQD04712	----------------R-T-		DD---------------
	Mycobacterium confluentis	WP_085148698	----------------R-T-		DS---------------
	Mycobacterium conspicuum	WP_085231400	----------------RQI-		-D---------------
	Mycobacterium cosmeticum	WP_036402235	----------------R-T-		DS--A------------
	Mycobacterium diernhoferi	WP_073857781	----------------R-T-		DD-----------G---
	Mycobacterium doricum	WP_085190070	----------------RQT-		DT--A------------
	Mycobacterium elephantis	WP_083042970	----QM--AE-V-----DT-		EYK-VA-------PA-
	Mycobacterium engbaekii	WP_085129329	----------------SRI-		DD-----------G---
	Mycobacterium europaeum	WP_085241621	----------------RQI-		SD-----------G---
	Mycobacterium fallax	WP_085095152	----------------R-T-		DT---------------
	Mycobacterium farcinogenes	WP_036394843	----TM--AEEV-----DT-		EYK-VA-------PA-
	Mycobacterium flavescens	WP_069415950	----------------RQI-		DN---------------
	Mycobacterium florentinum	WP_085220083	----------------RQI-		-D---------------
	Mycobacterium fortuitum	WP_061263410	----TM--AEEV-----DT-		EYK-VA-------PA-
	Mycobacterium fragae	WP_085195870	----------------RQI-		DV---------------
	Mycobacterium gastri	WP_036416013	----------------RQI-		-E---------------
	Mycobacterium genavense	WP_025735737	----------------RQI-		-D---------------
	Mycobacterium gilvum	WP_013472458	----------------KQI-		-K--A------------
	Mycobacterium goodii	WP_049747787	----------------Q-T-		DS--A------------
	Mycobacterium gordonae	WP_065047256	----------------RQI-		-D---------------
	Mycobacterium haemophilum	WP_047313282	----------------RQI-		TD---------------
	Mycobacterium hassiacum	WP_005625924	----------------R-I-		ER--A------------
	Mycobacterium heckeshornense	WP_048891237	-----------V----RQI-		DD-----------T---
	Mycobacterium heidelbergense	WP_083073142	----------------RQI-		-D-----------G---
	Mycobacterium heraklionense	WP_064999860	----------------SRI-		DD-----------G---
	Mycobacterium hiberniae	WP_085133956	----------------SRI-		DD-----------G---
	Mycobacterium holsaticum	WP_069403976	----------------KQI-		DK--A------------
	Mycobacterium houstonense	WP_066898750	----TM--AEEV-----DT-		EYK-VA-------PA-
	Mycobacterium icosiumassiliensis	WP_067970785	----------------SRI-		DD-----------G---
	Mycobacterium insubricum	WP_083030726	----------------R-T-		DT---------------
	Mycobacterium interjectum	WP_085201873	----------------RQI-		-E---------------
	Mycobacterium intermedium	WP_069419033	----------------RQI-		-D---------------

	Mycobacterium intracellulare	WP_064938516	----------------RQI-	VD--------------
	Mycobacterium iranicum	WP_064283177	----------------KQI-	EK--A-----------
	Mycobacterium kansasii	WP_063467941	----------------RQI-	-E--------------
	Mycobacterium komanii	CRL78181	----------------RQI-	DS--------------
	Mycobacterium kubicae	WP_085075282	----------------RQI-	-D-----------Q--
	Mycobacterium kumamotonense	WP_065287470	-----------------RI-	DD----------G---
	Mycobacterium kyorinense	WP_045383576	----------------RQI-	ED--A-----------
	Mycobacterium lacus	WP_085161137	---------------VRQI-	-D--------------
	Mycobacterium lentiflavum	CQD08387	----------------RQI-	-D--------------
	Mycobacterium leprae	WP_010907942	--------F-----L-GQI-	TD--------------
	Mycobacterium lepromatosis	WP_045842704	--------F-------RQI-	TD--------------
	Mycobacterium liflandii	WP_015354891	----------------RQI-	-D--------------
	Mycobacterium litorale	WP_078020436	----------------K-T-	DN----------G---
	Mycobacterium llatzerense	WP_071286853	----------------R-T-	DS--A-----------
	Mycobacterium longobardum	WP_085264325	-----------------RI-	DD----------G---
	Mycobacterium mageritense	WP_036428323	----------------R-I-	ED--------------
	Mycobacterium malmesburyense	CRL77337	----------------RQI-	DK--------------
	Mycobacterium malmoense	WP_065445002	----------------RQI-	DE----------G---
	Mycobacterium mantenii	WP_083096160	----------------RQI-	TD--------------
	Mycobacterium marinum	WP_012393213	----------------RQI-	-D--------------
	Mycobacterium microti	AMC60972	----------------RQI-	-D--------------
	Mycobacterium minnesotense	WP_083027550	----------------SRI-	DD----------G---
	Mycobacterium moriokaense	WP_083154196	----------------RQI-	DE--A-----------
	Mycobacterium mucogenicum	WP_064860827	----------------R-T-	DS--A-----------
	Mycobacterium nebraskense	WP_085165080	----------------RQI-	ED----------G---
	Mycobacterium neoaurum	WP_030134886	----------------R-T-	DD----------G---
	Mycobacterium neworleansense	CRZ15571	----------------R-T-	DD--------------
	Mycobacterium nonchromogenicum	WP_085137930	----------------SRI-	DD----------G---
	Mycobacterium noviomagense	WP_083086304	----------------SQI-	DD--A-------N---
	Mycobacterium novocastrense	WP_067395806	----------------RQI-	DS--------------
	Mycobacterium obuense	WP_046676626	----------------KQL-	-Q--A-----------
	Mycobacterium palustre	WP_085080122	----------------RQI-	-E--A-----------
**Other *Mycobacterium* (0/>100)**	*Mycobacterium paraense*	WP_085103864	----------------RQI-	-E--------------
	Mycobacterium paraffinicum	WP_073876057	----------------RQI-	VE--A-------G---
	Mycobacterium parafortuitum	WP_083143074	----------------KQI-	-K--A-----------
	Mycobacterium parascrofulaceum	WP_007168147	----------------RQI-	EE----------G---
	Mycobacterium paraseoulense	WP_083170015	----------------RQI-	SD----------G---
	Mycobacterium parmense	WP_085267686	----------------RQI-	ED----------G---
	Mycobacterium peregrinum	WP_064878169	----------------R-T-	DD--------------
	Mycobacterium phlei	WP_061481533	--------------V-RQI-	DD--------------
	Mycobacterium porcinum	WP_075921418	----------------R-T-	DD--------------
	Mycobacterium pseudoshottsii L15	GAQ34159	----TM--AE-V----SET-	EYK-VA-------PA-
	Mycobacterium rhodesiae	WP_083121086	----------------KQI-	TE--A-----------
	Mycobacterium riyadhense	WP_085252020	----------------RQI-	-D--------------
	Mycobacterium rufum	KGI67384	----------------KQI-	EQ--A-----------
	Mycobacterium rutilum	WP_083409867	----------------RQI-	DS--------------
	Mycobacterium saskatchewanense	WP_085254195	----------------RQI-	TD--A-------G---
	Mycobacterium scrofulaceum	WP_067268407	----------------RQI-	VE--A-----------
	Mycobacterium senuense	WP_085081769	-----------------RI-	DD----------G---
	Mycobacterium septicum	WP_044516772	----------------R-T-	DD--------------
	Mycobacterium setense	WP_064871726	----------------R-T-	DD--------------
	Mycobacterium sherrisii	WP_069402489	----------------RQI-	-D--------------
	Mycobacterium shigaense	BAX91421	----------------RQI-	-D----------G---
	Mycobacterium shimoidei	WP_069396567	----------------R-I-	DE--------------
	Mycobacterium shinjukuense	WP_083047320	----------------RQI-	TD--------------
	Mycobacterium simiae	WP_061558576	----------------RQI-	-D--------------
	Mycobacterium sinense	WP_064855427	------------G----RI-	DD----------G---
	Mycobacterium smegmatis	WP_003893311	----------------Q-T-	DS--A-----------
	Mycobacterium szulgai	WP_068029223	----------------RQI-	-D-----------Q--
	Mycobacterium terrae	WP_085262246	-----------------RI-	DD----------G---
	Mycobacterium thermoresistibile	WP_040548608	----------------RQI-	EK--A-----------
	Mycobacterium triplex	WP_036467364	----------------RQI-	-D--------------
	Mycobacterium triviale	WP_085110752	-----------------QI-	DQ--A----------V
	Mycobacterium tuberculosis	WP_070894055	----------------RQI-	-D--------------
	Mycobacterium tusciae	WP_006245874	----------------RQI-	TD--A-----------

**Other *Mycobacterium* (0/>100)**	*Mycobacterium ulcerans*	WP_011740491	----------------RQI-	-D---------------
	Mycobacterium vaccae	WP_003929160	----------------KQI-	-K--A------------
	Mycobacterium vanbaalenii	WP_011779031	----------------KQI-	-K--A------------
	Mycobacterium vulneris	WP_065461046	----------------R-T-	DD---------------
	Mycobacterium wolinskyi	WP_067854457	----------------R-T-	DD---------------
	Mycobacterium xenopi	WP_085196655	-----------V----RQI-	DD-----------N---
	Mycobacterium yongonense	WP_065503851	----------------RQI-	FD---------------
**Other bacteria**	*Actinoalloteichus cyanogriseus*	WP_035291288	-----------V----SMV-	GE--T--------G--V
	Actinomycetospora chiangmaiensis	WP_018335200	-----------V-----ML-	TQ--T--------G---
	Actinophytocola xanthii	WP_075126540	-R--------------RMV-	DQ-HT--------G---
	Amycolatopsis australiensis	SFW92558	----------------RMV-	DE--T--------G---
	Corynebacterium amycolatum	WP_005511163	-------------K---AV-	DE--T--------G--L
	Geodermatophilus obscurus DSM 43160	ADB76816	-----------------MV-	DK--T--------G--V
	Gordonia jacobaea	WP_049698967	---------------MSAL-	EQK-T--------G--V
	Kitasatospora griseola	WP_043915922	-----------V-K-ITML-	-K--TL-------GQV-
	Lentzea flaviverrucosa	SER27622	----------------NMV-	DE--T--------G---
	Nocardia beijingensis	WP_067810611	----------------GMV-	DK--T--------G---
	Pseudonocardia autotrophica	WP_051737104	-----------V---MRML-	DE-HT--------G---
	Rhodococcus jostii	SEE52062	----------------GMV-	DK--T--------G---
	Streptomyces anulatus	WP_030580419	-----------V---ITML-	PK--T--------GAV-
	Tsukamurella paurometabola	WP_041944752	----------------TMV-	TD--T--------G---
	Yuhushiella deserti	SFQ18237	----------------RMV-	EQ--T--------G---

Supplementary Figure 22

A partial sequence alignment of a conserved region of ATP-dependent helicase showing a three amino acid insertion that is specific for members of the "*Abscessus-Chelonae"* clade and absent in other bacteria.

Group	Species	Accession	13		52
**“Abscessus-Chelonae” Clade (6/6)**	*Mycobacterium abscessus*	WP_005056099	PNTGNAIRMVAGTGCELHLVRPL		FDLSEAKVRRAGLDYHD
	Mycobacterium abscessus subsp. bolletii	EHM16642	-----------------------		-----------------
	Mycobacterium chelonae	WP_070915807	--------------------Q--		-----------------
	Mycobacterium franklinii	WP_070939304	--------------------D--		-----------------
	Mycobacterium immunogenum	WP_064627879	-----------------------		-----------------
	Mycobacterium saopaulense	WP_070909471	--------------------Q--		-----------------
**Other *Mycobacterium* (1/>100)**	*Mycobacterium thermoresistibile*	WP_003928097	-----------A--A-----D--		-----P-L---------
	Mycobacterium alsense	WP_083139323	--------TA-A--A-----E--	G	-----P-L---------
	Mycobacterium angelicum	WP_083115639	--------TA-A--------E--	G	-----P-L---------
	Mycobacterium aromaticivorans	WP_036346078	-----------A--------E--	G	-----P-L---------
	Mycobacterium arosiense	WP_083066302	--------TA-T--------E-M	G	-----P-L---------
	Mycobacterium arupense	WP_046189778	-----------A--A-----E--	G	-----P-L---------
	Mycobacterium asiaticum	WP_065036046	--------TA-A--------E--	G	-----P-L---------
	Mycobacterium aurum	WP_087019836	-----------A--------E--	G	-----P-L---------
	Mycobacterium avium	WP_003879015	--------TA-A--------E-I	G	-----P-L---------
	Mycobacterium avium subsp. avium	EUA37500	--------TA-A--------E-M	G	-----P-L---------
	Mycobacterium avium subsp. paratuberculosis	EGO38687	--------TA-A--------E-I	G	-----P-L---------
	Mycobacterium bacteremicum	WP_083058912	-----------A--AH----E--	G	-----P-L---------
	Mycobacterium boenickei	WP_077743364	---------------A----E--	G	-----P-L---------
	Mycobacterium bohemicum	WP_085180146	--------TA-A--------E--	G	-----PQL---------
	Mycobacterium bovis	CEJ36703	--------TC-A--------E--	G	-----P-L---------
	Mycobacterium branderi	WP_083130031	--------TA-A--A-----E--	G	-----P-L---------
	Mycobacterium brisbanense	GAS89884	---A--------E--	G	-----P-L---------
	Mycobacterium canariasense	WP_062659849	-----------A--------E--	G	-----P-L---------
	Mycobacterium canettii	WP_015291396	--------TC-A--------E--	G	-----P-L---------
	Mycobacterium celatum	WP_062541731	--------TA-A--------E--	G	-----P-L---------
	Mycobacterium celeriflavum	WP_083000876	-----------A--------E--	G	-----P-L---------
	Mycobacterium chimaera	ASL11265	--------TA-A--------E-M	G	-----P-L---------
	Mycobacterium chlorophenolicum	KMO71944	--------------------E--	G	-----P-L---------
	Mycobacterium chubuense	WP_041781758	--------------A-----E--	G	-----P-L---------
	Mycobacterium colombiense	WP_064880824	--------T--A--------E-M	A	-----P-L---------
	Mycobacterium conceptionense	WP_065062012	--------------------E--	G	-----P-L---------
	Mycobacterium confluentis	WP_085149228	-----------A--A-----E--	G	-----P-L---------
	Mycobacterium conspicuum	WP_085234189	--------TA-A--------E--	G	-----P-L---------
	Mycobacterium cosmeticum	WP_036401883	-----------A--------E--	G	-----P-L---------
	Mycobacterium diernhoferi	WP_073856494	-----------A--------E--	G	-----P-L---------
	Mycobacterium doricum	WP_085187555	------------S----------	G	-----P-L---------
	Mycobacterium engbaekii	WP_085126606	-----------A--A-----E-M	G	-----P-L---------
	Mycobacterium europaeum	WP_085242214	--------TA-A--------E--	G	-----PQL---------
	Mycobacterium fallax	WP_085095342	--------------A-----E--	G	-----P-L---------
	Mycobacterium farcinogenes	WP_036388877	--------------------E--	G	-----P-L---------
	Mycobacterium flavescens	WP_069414723	--------------------E--	G	-----P-L---------
	Mycobacterium florentinum	WP_085219915	--------TA-A--A-----E-M	G	-----P-L---------
	Mycobacterium fortuitum	WP_064850535	--------------------E--	G	-----P-L---------
	Mycobacterium fragae	WP_085198482	--------TA-A--S-----E--	G	-----P-L---------
	Mycobacterium gastri	WP_036418197	--------TA-A--------E--	G	-----P-L---------
	Mycobacterium genavense	WP_025737438	--------TA-A--T-----E-M	G	-----P-----------
	Mycobacterium gilvum	WP_011895722	---------------A----E--	G	-----P-L---------
	Mycobacterium goodii	WP_049749155	-----------A--------H--	G	-----P-L---------
	Mycobacterium gordonae	WP_065043889	--------TA-A--------E--	G	-----P-L---------
	Mycobacterium haemophilum	WP_054880604	-------TA-V--------E-M	G	-----P-L---------
	Mycobacterium hassiacum	WP_005625690	-----------A--------E--	G	-E-TDS-L---------
	Mycobacterium heckeshornense	WP_048893273	-----------A--A-----E--	G	-----P-L---------
	Mycobacterium heidelbergense	WP_083077361	--------TA-A--A-----E--	G	-----P-L---------
	Mycobacterium heraklionense	WP_065039977	-----------A--A-----E-M	G	-----P-L---------
	Mycobacterium hiberniae	WP_085135261	-----------A--A-----E-M	G	-----P-L---------
	Mycobacterium holsaticum	WP_069406234	----------SA--A-----E--	G	-----P-L---------
	Mycobacterium houstonense	WP_066903853	--------------------E--	G	-----P-L---------
	Mycobacterium icosiumassiliensis	WP_067970234	-----------A--AQ----E--	G	-----P-L---------
	Mycobacterium insubricum	WP_083031127	-----------A--TH----E--	G	-----P-L---------
	Mycobacterium interjectum	WP_066907863	--------TA-A--A-----E--	G	-----P-L---------
	Mycobacterium intermedium	WP_069422140	--------TA-A--------E--	G	-----P-L---------
	Mycobacterium iranicum	WP_064284899	--------------------E--	G	-----P-L---------
	Mycobacterium kansasii	WP_063471958	--------TA-A--------E--	G	-----P-L---------

**Other *Mycobacterium* (1/>100)**	*Mycobacterium komanii*	CRL75435	-----------A--------E-M G -----P-L---------
	Mycobacterium kubicae	WP_085073751	--------TA-A--------E-- G -----P-L---------
	Mycobacterium kyorinense	WP_065015026	--------TA-A--------E-- G -----P-L---------
	Mycobacterium lacus	WP_085162086	--------TA-A--------E-- G -----P-L---------
	Mycobacterium lentiflavum	CQD07443	--------TA-A--A-----E-M G -----S-----------
	Mycobacterium leprae	CAB09926	--------TA-V---A----E-I G ----NP-L---------
	Mycobacterium lepromatosis	WP_045842466	-------TA-A---A----E-I G ----D--L----V----
	Mycobacterium litorale	WP_078020570	-----------A--------Q-- G -----P-L---------
	Mycobacterium longobardum	WP_085262976	-----------A--A-----E-M G -----P-L---------
	Mycobacterium malmesburyense	CRL78481	--------------------E-M G -----P-L---------
	Mycobacterium malmoense	WP_065441444	--------TA-A--------E-- G -----PQL---------
	Mycobacterium mantenii	WP_083100023	--------T--A--------E-M G -----P-L---------
	Mycobacterium marseillense	WP_083020504	--------TA-A--------E-M G -----P-L---------
	Mycobacterium minnesotense	WP_083023567	-----------A--A-----E-- G -----P-L---------
	Mycobacterium moriokaense	WP_083154001	-----------A--------E-- G -----P-L---------
	Mycobacterium mucogenicum	WP_082981826	-----------A--A-----E-- G -----P-L---------
	Mycobacterium nebraskense	WP_046187152	--------TA-A--------E-M G -----P-L---------
	Mycobacterium neoaurum	WP_030134692	-----------A--------E-- G -----P-L---------
	Mycobacterium neworleansense	CRZ15836	--------------------E-- G -----P-L---------
	Mycobacterium noviomagense	WP_083087836	-----------A--A-----E-- G -----P-L---------
	Mycobacterium novocastrense	WP_067396130	-----------A--------E-M G -----P-L---------
	Mycobacterium obuense	WP_046361727	--------------------E-- G -----P-L---------
	Mycobacterium palustre	WP_085079350	--------TA-A--V-----E-- G -----P-L---------
	Mycobacterium paraense	WP_085096478	--------TA-A--A-----E-- G -----P-L---------
	Mycobacterium paraffinicum	WP_073880236	--------TA-A--------E-M G -----P-L---------
	Mycobacterium parafortuitum	WP_083145811	------------S-------E-- G -----P-L---------
	Mycobacterium paraseoulense	WP_083169695	--------TA-A--------E-- G -----PQL---------
	Mycobacterium parmense	WP_085270590	--------T--A--------E-M G -----P-L---------
	Mycobacterium peregrinum	WP_064880198	--------------------E-- G -----P-L---------
	Mycobacterium phlei	WP_003886498	-----------A--A-----E-- G -----P-L---------
	Mycobacterium porcinum	WP_075921788	--------------------E-- G -----P-L---------
	Mycobacterium rhodesiae	WP_083122412	-----------A--------E-- G -----P-L---------
	Mycobacterium riyadhense	WP_085252926	--------TA-A--------E-- G -----P-L---------
	Mycobacterium rufum	KGI67161	------------S-------E-- G -----P-L---------
	Mycobacterium rutilum	WP_083410080	-----------A--A-----E-- G -----P-L---------
	Mycobacterium saskatchewanense	WP_085255607	--------TA-A--------E-- G -----PQL---------
	Mycobacterium scrofulaceum	WP_067280573	--------TA-A--------E-M G -----P-L---------
	Mycobacterium senuense	WP_085083247	-----------A--A-----E-- G -----P-L---------
	Mycobacterium septicum	WP_044516427	--------------------E-- G -----P-L---------
	Mycobacterium setense	WP_039317246	--------------------E-- G -----P-L---------
	Mycobacterium sherrisii	WP_069399076	--------TA-A--A-----E-- G -----P-L---------
	Mycobacterium shigaense	BAX91224	--------TA-A--A-----E-- G -----P-L---------
	Mycobacterium shimoidei	WP_069395318	--------TA-A--A-----E-- G -----P-L---------
	Mycobacterium shinjukuense	WP_083052287	--------TC-A--------E-- G -----P-L---------
	Mycobacterium simiae	WP_061558435	--------TA-A--A-----E-- G -----P-L---------
	Mycobacterium sinense	WP_065026135	-----------A--A-----E-- G -----P-L---------
	Mycobacterium smegmatis	WP_011727792	-----------A--------H-- G -----P-L---------
	Mycobacterium szulgai	WP_085671310	--------TA-A--------E-- G -----P-L---------
	Mycobacterium terrae	WP_085260983	-----------A--A-----E-- G -----P-L---------
	Mycobacterium triplex	WP_036467034	--------TA-A--T-----E-M G -----P-----------
	Mycobacterium triviale	WP_085111391	--------------A-----Q-- G -----P-L---------
	Mycobacterium tuberculosis	SGD49918	-----I--LC-N--FR---IH-- G -TWDDKRL-------SE
	Mycobacterium tusciae	WP_083124881	-----------A--------E-- G -----P-L---------
	Mycobacterium ulcerans	WP_011740751	--------TA-A--------E-- G -----P-L---------
	Mycobacterium vaccae	WP_003929284	--------------------E-- G -----P-L---------
	Mycobacterium vulneris	WP_085292090	--------TA-A--------E-M G -----P-L---------
	Mycobacterium wolinskyi	WP_067842905	-----------A--------Q-- G -----P-L---------
	Mycobacterium xenopi	WP_085197248	-----------A--A-----E-- G -----P-L---------
**Other bacteria**	*Acidothermus cellulolyticus*	WP_011719306	--------LA-A--------G-- A --M-D-RL--------E
	Amycolatopsis balhimycina	WP_020638314	--------LA-N--------E-- G -T-EDKQL---------
	Arthrobacter enclensis	SCB82311	-------LA-I--A-----E-- G --F-D--L---------
	Brevibacterium album	WP_029089180	-------LA-V--AH----E-- G ----D--L---------
	Cellulomonas fimi	WP_013772418	--------A----AT----E-- G ------RL---------
	Corynebacterium glucuronolyticum	WP_005394981	---------C----AH---I--- G ---EDRHL---------
	Gordonia amicalis	WP_006434487	--------LA-N-------IE-- G -SM-D-Q-K-------E

**Other bacteria**	*Halomonas meridiana*	WP_074211253	-----IM-L--NN--R---IE-- G ---E-K-L-------R-
	Nocardia alba	WP_067447973	--------LA-----A---IE-- G -----S-L---------
	Rhodococcus defluvii	WP_031938634	--------------------G-- G -----P-LK--------
	Saccharomonospora glauca	WP_040919926	-------LA-N-------IE-- G -SMEDRYL---------
	Tsukamurella pulmonis	WP_068528760	--------IA-V--------E-- G -----P-L---------
	Vibrio coralliilyticus	WP_065741066	-----I--LC-NC-AN---IE-- G ---E-K-----------
	Yuhushiella deserti	SFQ56360	--------LA-N----------- G ---DARN----------

Supplementary Figure 23

A partial sequence alignment of a conserved region of tRNA (cytidine(34)-2'-O)-methyltransferase showing a one amino acid deletion that is specific for members of the "*Abscessus-Chelonae"* clade and absent in most other bacteria.

			49		81
	Mycobacterium abscessus	WP_052618678	AGRLANLESAIAES		GESFAATTGMGHTRWATHG
	Mycobacterium abscessus subsp. bolletii	EUA66320	--------------		-------------------
***"Abscessus-Chelonae"* Clade (6/6)**	*Mycobacterium chelonae*	WP_070919449	--------------		----G--------------
	Mycobacterium franklinii	WP_070938584	--------------		-------------------
	Mycobacterium immunogenum	WP_043076525	--------A-----		-------------------
	Mycobacterium saopaulense	WP_070909443	--------A-----		-------------------
	Mycobacterium angelicum	WP_083112990	--------A-V-DM	T	QAEL-G---L---------
	Mycobacterium aromaticivorans	WP_036340734	--------E-LT-T	G	-DAL-G-A-----------
	Mycobacterium arosiense	WP_083065467	--------H-V-QM	P	PL-LTG---L---------
	Mycobacterium asiaticum	WP_065142826	--Q-----A----M	T	PTEL-G-A-L---------
	Mycobacterium avium	WP_084023132	--------E-V-QL	P	PSALTG---L---------
	Mycobacterium avium subsp. paratuberculosis	EGO39574	--------E-V-QL	P	PSALTG---L---------
	Mycobacterium boenickei	WP_077743951	--------ATL--T	D	EGVLTGS--L---------
	Mycobacterium bohemicum	WP_085180106	--------E-V-GM	A	P--KTG---L---------
	Mycobacterium bovis	WP_079293635	--------E-V--M	P	STALSG---L---------
	Mycobacterium branderi	WP_083130073	--------Q-LE-A	D	PAAL-GG--L---------
	Mycobacterium brisbanense	WP_062830061	----V---A-L--T	D	DDLLTGS--L---------
	Mycobacterium canettii	WP_014001742	--------E-V--M	P	STALSG---L---------
	Mycobacterium celatum	WP_085168010	--------H-LE-A	D	PAAL-GG--L---------
	Mycobacterium celeriflavum	WP_083000950	--------A-LS-T	G	AAGLTGS--L---------
	Mycobacterium chlorophenolicum	WP_048471870	--------A-LT-A	G	ED-LVGS--L---------
	Mycobacterium chubuense	WP_014814372	--------A-LG-A	E	DGTLVGA--L---------
	Mycobacterium colombiense	WP_007773444	--------E-V--M	P	AS-LTG---L---------
	Mycobacterium conceptionense	CQD06302	--------T-L--T	D	DNILTGS--L---------
	Mycobacterium confluentis	WP_085149355	--------E-L--T	D	AADL-G-S-----------
	Mycobacterium cosmeticum	WP_036401782	--------E-VT-T	D	AALLTG-------------
	Mycobacterium elephantis	WP_083043862	--------AELSAT	G	AA-L-G---L---------
	Mycobacterium europaeum	WP_085242169	--------D-V-HM	P	P--L-G---L---------
	Mycobacterium fallax	WP_085092586	--------EEL-GT	D	PAEL-G---V---------
	Mycobacterium flavescens	WP_069416858	----------LTDT	D	AAGLVGS--L---------
	Mycobacterium florentinum	WP_085224622	----E--DK-V--M	P	PDDLR----L---------
	Mycobacterium fortuitum	WP_061262602	--------T-L--T	D	DGILTG---L---------
	Mycobacterium gastri	WP_036410038	--------A-V-DM	T	PSELTG---L---------
	Mycobacterium gordonae	WP_065133264	--Q-----A-L--M	T	SDELSG---L---------
**Other *Mycobacterium* (0/91)**	*Mycobacterium haemophilum*	WP_047315550	----S-----L--M	V	PA-L-G-V-L---------
	Mycobacterium hassiacum	WP_018354617	------------DT	D	PALLCGS--L---------
	Mycobacterium heidelbergense	WP_083075048	--------E-V--M	A	PAAT-G---L---------
	Mycobacterium holsaticum	WP_069404767	--------NELSAT	D	AQAL-GS--L---------
	Mycobacterium houstonense	WP_066901119	--------ATL--T	D	DGVLTG---L---------
	Mycobacterium insubricum	WP_083031995	--------E-L-DT	D	PSELTG---V---------
	Mycobacterium interjectum	WP_066907647	-------DE-V-GM	A	PA-K-G---L---------
	Mycobacterium intermedium	WP_069417521	--------A-T--M	S	PAEL-GC--L---------
	Mycobacterium intracellulare	ETZ31864	--------E-V-QM	P	RH-LTG---L---------
	Mycobacterium iranicum	WP_085172857	--------A-L---	G	A--P-GA--L---------
	Mycobacterium kansasii	WP_063469999	--------A-V-DT	A	PTELTG---L---------
	Mycobacterium komanii	CRL75336	--------A-LT-T	D	AS-LGGS--L---------
	Mycobacterium koreense	WP_085304911	--Q-----A-L--M	D	PALLTGS--L---------
	Mycobacterium kubicae	WP_085073715	--------DT-G-M	T	PNALSG---L---------
	Mycobacterium kyorinense	WP_065013419	--------ESLRDT	D	SAALSG---L---------
	Mycobacterium lacus	WP_085160725	--------E-M--M	P	PAAL-GS--L---------
	Mycobacterium lentiflavum	CQD07064	----E--GK-V-DM	P	L-DLG----L---------
	Mycobacterium leprae	AAA17307	----S----VL--M	V	PA-L-GNV-L--I------
	Mycobacterium lepromatosis	WP_045842428	----S-----L--M	V	PA-L-GNV-L---------
	Mycobacterium liflandii	WP_041298859	--------T-V--M	P	A--LTG---L---------
	Mycobacterium litorale	WP_078020628	--------Q-LE-T	D	AAALTG-A-----------
	Mycobacterium longobardum	WP_085263018	--------A-L-DT	D	PALLVGG--L---------
	Mycobacterium malmesburyense	CRL78430	--------A-LT-T	D	AS-LVGS--L---------
	Mycobacterium malmoense	WP_065441518	--------E-V-QM	P	P--LGG---L---------
	Mycobacterium mantenii	WP_083093470	--------E-V--M	P	PS-LTG---L---------
	Mycobacterium marinum	WP_012393001	--------T-V--M	S	A--LTG---L---------
	Mycobacterium marseillense	WP_083019851	--------E-V-QM	P	PS-LTG---L---------
	Mycobacterium moriokaense	WP_083154084	----D--TA-L--T	D	AATL-GS--L---------
	Mycobacterium mucogenicum	WP_064859827	--------E-L--T	D	AAQLEG-------------
	Mycobacterium mungi	WP_064319709	--------E-V--M	P	STALSG---L---------

Other *Mycobacterium* (0/91)	*Mycobacterium nebraskense*	WP_046185363	--------E-V-QM P P--LGG---L---------
	Mycobacterium neoaurum	WP_030134565	--------E-LG-A G -DALVGS-----------
	Mycobacterium neworleansense	CRZ15963	--------T-L--T D DGILTG---L---------
	Mycobacterium noviomagense	WP_083087878	--------K-LEDT D AAALTGS--L---------
	Mycobacterium novocastrense	WP_067396219	--------A-L-DT E AS-L-GN--L---------
	Mycobacterium obuense	WP_046363959	----------L--Q S S-DLVGS--L---------
	Mycobacterium paraense	WP_085096388	--------E-V--M A PA-K-G---L---------
	Mycobacterium paraffinicum	WP_073880309	--------E-V-QM P P--LGG---L---------
	Mycobacterium parafortuitum	WP_083146882	--------A-L--- G A--P-GA--L---------
	Mycobacterium parascrofulaceum	WP_040622065	--------E-V-QM P P--LGG---L---------
	Mycobacterium paraseoulense	WP_083175382	--------E-V-QM P P--LGG---L---------
	Mycobacterium parmense	WP_085272011	--------E-V--M P P--L-GA--L---------
	Mycobacterium peregrinum	WP_064888051	--------T-L--T D ENILTGS--L---------
	Mycobacterium persicum	WP_083153099	--------A-V-DM A PSELTG---L---------
	Mycobacterium porcinum	WP_069427464	--------ATL--T D DGVLTGS--L---------
	Mycobacterium rhodesiae	WP_083121697	--------E-L--T E ADAL-GAA-----------
	Mycobacterium riyadhense	WP_085249891	----S---A-V-DM T PSEL-GG--L---------
	Mycobacterium rufum	KGI67101	--------T-LT-A G ADNLIGS--L---------
	Mycobacterium rutilum	WP_083406416	--------M-LD-T V -DVL-GS--L------S--
	Mycobacterium saskatchewanense	WP_085255558	--------D-V-QM P P--L-G---L---------
	Mycobacterium scrofulaceum	WP_067280368	--------D-V-QM P P--LGG---L---------
	Mycobacterium septicum	WP_044516315	--------T-L--T D DGVLTG---L---------
	Mycobacterium setense	WP_064876084	--------TTL--T D DGVLSGS--L---------
	Mycobacterium sherrisii	WP_085166231	----E--DK-V--M A PAELGG---L---------
	Mycobacterium shinjukuense	WP_083045871	--------A-V-DM P AA-L-GS--L---------
	Mycobacterium simiae	WP_061558392	----E--DK-V--M A P-DLGG---L---------
	Mycobacterium szulgai	WP_085672412	--------A-V-DM T QAEL-G---L---------
	Mycobacterium terrae	WP_085261324	--------ADL-RT D PSLLVGG--L---------
	Mycobacterium tuberculosis	WP_069334376	--------E-V--M P STALSG---L---------
	Mycobacterium tusciae	WP_083124801	--------T-L--- E AGTL-GS--L---------
	Mycobacterium ulcerans	WP_011739129	--------T-V--M P A--LTG---L---------
	Mycobacterium vaccae	WP_003929235	--------A-L--- G PDHLVGA--L---------
	Mycobacterium vulneris	WP_065459531	--------ATL--T D DGVLTGS--L---------
	Mycobacterium wolinskyi	WP_067842590	--------A-L--T D DSTLTGS--L---------
	Mycobacterium yongonense	WP_065499535	--------E-V-QM P RH-LTG---L---------
Other bacteria	*Actinopolyspora mzabensis*	SDJ77474	--A-S---QRV--D G R-H-VG-G-----------
	Amycolatopsis azurea	WP_039919713	--------TQLDTV G RDA-YG-A-----------
	Corynebacterium capitovis	WP_018017545	--K-----DK---V G S--LNG--AI---------
	Gordonia aichiensis	WP_040518235	----E--DKQ--AV G R-ALTG-------------
	Kutzneria albida	WP_025361333	--------ARLD-V G RDHVSG-------------
	Lechevalieria aerocolonigenes	WP_030472004	----Q---TRLD-V G RDN--G-V-----------
	Nocardia acidivorans	WP_067561793	--------AEF-AT G IDR-TGA------------
	Prauserella marina	SDC76949	--------A-LDTA G RDA--G-A-----------
	Rhodococcus kroppenstedtii	WP_068360446	--K-S--DAELDDL G RDT-VG-A-----------
	Saccharopolyspora flava	SFS43505	--A-S---KKL--G D -TG-EGNS-----------
	Thermocrispum agreste	WP_028848758	----G---QQLDAV G RDA--GSA-----------
	Yuhushiella deserti	SFQ25821	--Q-----AELDAV G RDG--G-A-----------

Supplementary Figure 24

A partial sequence alignment of a conserved region of glutamine-fructose-6-phosphate transaminase (isomerizing) showing a one amino acid deletion that is specific for members of the "*Abscessus-Chelonae*" clade and absent in other bacteria.

Group	Species	Accession	674		715
**"*Abscessus-Chelonae*" Clade (6/6)**	*Mycobacterium abscessus*	WP_052620523	GPIQGGSVHPYIRRYNG	I	DTNWEYEHPSMERALKKTLGVPLF
	Mycobacterium abscessus subsp. bolletii	EHM16643	-----------------	-	------------------------
	Mycobacterium chelonae	WP_070919441	----------------K	-	-KD--HD----AA--D--------
	Mycobacterium franklinii	WP_070938613	----------------K	-	-KD-QHD----AA--D--------
	Mycobacterium immunogenum	WP_064627878	----------------K	-	-KD-QHD----AA--D--------
	Mycobacterium saopaulense	WP_070909470	----------------K	-	-KD-KHD----AA--D--------
**Other *Mycobacterium* (0/>100)**	*Mycobacterium acapulcensis*	WP_066810469	------------K-R--		EEPVT-D-----P--H--------
	Mycobacterium africanum	WP_049958458	--------------R--		VDPVI------AP--R--------
	Mycobacterium alsense	WP_083141122	--------------R--		LDPVV-D-----P--R--------
	Mycobacterium angelicum	WP_083115636	--------------R--		IDPVV-------S--R--------
	Mycobacterium aromaticivorans	WP_036340629	------------K-R--		LEEVT-D-----P--R--------
	Mycobacterium arosiense	WP_083066300	--------------R--		EAPVV-D-----P--R-----A--
	Mycobacterium arupense	WP_046686430	--------------R--		IDPVT----C-----E----I---
	Mycobacterium asiaticum	WP_065036050	-----N------K-R--		IEPVV-D-----P--R--------
	Mycobacterium aurum	WP_087019824	------------K-R--		IEPVV-D-----P--R--------
	Mycobacterium avium	WP_062889850	--------------R--		VDPVL-D-----P--R--------
	Mycobacterium avium subsp. avium	EUA28361	--------------R--		EDPVV-D-----P--R--------
	Mycobacterium avium subsp. hominissuis	ETB36080	--------------R--		VDPVL-D-----P--R--------
	Mycobacterium avium subsp. paratuberculosis	EGO38685	--------------R--		VDPVL-D-----P--R--------
	Mycobacterium bacteremicum	WP_083058923	------------K-R--		EEEVV-D----KP--E--------
	Mycobacterium boenickei	WP_077743360	--------------R--		LEKVA-D-----S-------I---
	Mycobacterium bohemicum	WP_085180143	--------------R--		LDPVV-D-----P--R--------
	Mycobacterium bovis	WP_024456539	--------------R--		VDPVI------AP--R--------
	Mycobacterium branderi	WP_083130033	--------------R-E		IDPVV-D-----T--R--------
	Mycobacterium brisbanense	WP_062830175	--------------R-K		MEKVT-D-D--KP--E----I---
	Mycobacterium canariasense	WP_062659852	--------------R--		QEEVT-D-----P--S--------
	Mycobacterium canettii	WP_015288522	--------------R--		VDPVI------AP--R--------
	Mycobacterium celatum	WP_062541729	--------------R-D		VDPVV-D-----T--R--------
	Mycobacterium celeriflavum	WP_083000891	------------K-R--		EEPVT------AP--H--------
	Mycobacterium chimaera	ASL11267	--------------R--		EDPVV-D-----P--R--------
	Mycobacterium chlorophenolicum	WP_048471498	------------K-R--		EEPVT-D-----S--R--------
	Mycobacterium chubuense	WP_014814423	------------K-R--		EEAIT-------S--R--------
	Mycobacterium colombiense	WP_064880830	--------------R--		LDPVL-D-----P--R--------
	Mycobacterium conceptionense	CQD05943	------------K-R--		LEEVT-------A-----------
	Mycobacterium confluentis	WP_085149236	--------------R--		LDPVV-D-----P--R--------
	Mycobacterium conspicuum	WP_085234185	--------------R--		LDPVV-D-----P--R--------
	Mycobacterium cosmeticum	CDO09932	--------------R--		QEEVT-D-----P--R--------
	Mycobacterium diernhoferi	WP_073856489	------------K-R--		IEPVV-D-----P--R--------
	Mycobacterium doricum	WP_085192447	------------K-R--		EEVVT-D-----N--R--------
	Mycobacterium elephantis	WP_046752491	------------K-R--		VEPVT-D-----P--R--------
	Mycobacterium engbaekii	WP_085126604	--------------R--		IDPVT----C-A---H----I---
	Mycobacterium europaeum	WP_085242212	--------------R--		LDPVT-D-----P--R--------
	Mycobacterium fallax	WP_085095346	--------------R--		LEPPDC-----QN--R--------
	Mycobacterium farcinogenes	CDP84693	------------K-R--		LEEVT-------A-----------
	Mycobacterium flavescens	WP_069414591	------------K-R--		EEAVT-------P--H--------
	Mycobacterium florentinum	WP_085219912	--------------R--		IDPVV-D-----S--R--------
	Mycobacterium fortuitum	WP_061262631	------------K-R--		LEKVT-D-----A-----------
	Mycobacterium fragae	WP_085198471	--------------R--		VDPVV-D-----S--R--------
	Mycobacterium gastri	WP_036412365	--------------R--		IDPVV------AP--R--------
	Mycobacterium genavense	WP_025737443	--------------R--		LDPVV-D-----H--R--------
	Mycobacterium gilvum	WP_013472635	------------K-R--		QEPVT----------R--------
	Mycobacterium goodii	WP_049748029	------------K-R--		LEPVT-D-----P-----------
	Mycobacterium gordonae	WP_065044067	--------------R--		LDPVV-D-----P--R--------
	Mycobacterium hassiacum	WP_005625680	------------K-R--		EEPVT-------P--R--------
	Mycobacterium heckeshornense	WP_048893269	--------------R--		IDPVV-D-----S--R--------
	Mycobacterium heidelbergense	WP_083077367	--------------R-K		LDKV-C------P--L--------
	Mycobacterium heraklionense	WP_064889008	--------------R--		IDPVT----C-A---E--Y-----
	Mycobacterium hiberniae	ORV70643	--------------R--		IDPVT----C-A---H----I---
	Mycobacterium indicus pranii	WP_014942850	--------------R--		EDPVV-D-----P--R--------
	Mycobacterium insubricum	WP_083031132	--------------R-E		LEPIH-D-----P--R--------
	Mycobacterium interjectum	WP_066907857	--------------R--		VDPVV-D-----S--R--------
	Mycobacterium intermedium	WP_079220282	--------------R--		IDPVT-------S--R--------
	Mycobacterium intracellulare	WP_064893686	--------------R--		EDPVV-D-----P--R--------
	Mycobacterium iranicum	WP_064284902	------------K-R--		QEEVT-D-----S--R--------

Other *Mycobacterium* (0/>100)

Mycobacterium kansasii	WP_063467729	--------------R--	IDPVV------AP--R--------
Mycobacterium komanii	CRL75427	------------K-R--	EEPVT-D-----P--H--------
Mycobacterium kubicae	WP_085073748	--------------R--	LDPVL-------P--R--------
Mycobacterium kumamotonense	WP_065287307	--------------R--	IDPVT----------R----I---
Mycobacterium kyorinense	WP_065015028	--------------R--	VDPTV-D-----S--R--------
Mycobacterium lacus	WP_085162089	--------------R--	IDPVG-D-----P--R--------
Mycobacterium lentiflavum	CQD07422	--------------R--	IDPVV-D-----P--R--------
Mycobacterium liflandii	WP_015354759	--------------R--	IDPVV-------S--R--------
Mycobacterium llatzerense	WP_071287193	--------------H--	LEDATC--Q---A--TR-------
Mycobacterium mageritense	WP_036428854	------------K-R--	LE-VT-D-----P-----------
Mycobacterium malmesburyense	CRL78477	------------K-R--	EEPVT-D-----P--H--------
Mycobacterium malmoense	WP_065441450	--------------R--	LDPVV-D----AP--R--------
Mycobacterium mantenii	WP_083100020	--------------R--	LDPVV-D-----P--R--------
Mycobacterium marinum	WP_081435777	--------------R--	IDPVV-------S--R--------
Mycobacterium marseillense	WP_083020505	--------------R--	LDPVV-D-----S--R--------
Mycobacterium minnesotense	WP_083023565	--------------R--	IDPVT----C-A--------I---
Mycobacterium moriokaense	WP_083154006	------------K-R--	EEPVT-D-----P--R--------
Mycobacterium mucogenicum	WP_064860882	--------------H--	LEDATC--K---A--TR-------
Mycobacterium nebraskense	WP_085165017	--------------R--	LDPVV-D-----P--R--------
Mycobacterium neoaurum	WP_030134689	------------K-R--	EEEVT-D----KP--E--------
Mycobacterium neworleansense	CRZ15882	------------K-R--	LEKVT-D-----P-----------
Mycobacterium nonchromogenicum	WP_085139817	--------------R--	IDPVT----C-V---E----I---
Mycobacterium noviomagense	WP_083087885	--------------R--	VDPVV-D-----Q--R--------
Mycobacterium novocastrense	WP_067396136	------------K-R--	EEPVT-D-----P--H--------
Mycobacterium obuense	WP_046361729	------------K-R--	EEEVV-D-----S--R--------
Mycobacterium palustre	WP_085079376	--------------R--	IDPVV-D-----P--R--------
Mycobacterium paraense	WP_085096291	--------------R--	VDPVI-D-----P--R--------
Mycobacterium paraffinicum	WP_073880226	--------------R--	LDPVV-D-----P--R--------
Mycobacterium parafortuitum	WP_083145813	------------K-R--	QEPVT-D--------R--------
Mycobacterium parascrofulaceum ATCC BAA-	EFG76163	--------------R--	LDPVV-D-----P--R--------
Mycobacterium paraseoulense	WP_083169689	--------------R--	LDPVV-D-----P--R--------
Mycobacterium parmense	WP_085270592	--------------R--	LDPVV-D-----P--R--------
Mycobacterium peregrinum	WP_064880190	--------------R--	LEPVT-D-----S--R----I---
Mycobacterium persicum	WP_083156309	--------------R--	VDPVV------AP--R--------
Mycobacterium phlei	AMO60292	------------K-R--	EEPVT-D-----P--H--------
Mycobacterium porcinum	WP_069425015	--------------R--	LEKVT-D-----S-------I---
Mycobacterium pseudoshottsii	WP_086085245	--------------R--	IDPVV-------S--R--------
Mycobacterium rhodesiae	WP_083122262	------------K-R--	LEEVT-D-----P--R--------
Mycobacterium riyadhense	WP_085252924	--------------R--	LDPVV------AP--R--------
Mycobacterium rufum	KGI67159	------------K-R--	EEAVT-D-----S--R--------
Mycobacterium rutilum	WP_083410084	------------K-R--	EETIT-D-----P--H--------
Mycobacterium saskatchewanense	WP_085255605	--------------R--	LDPVV-D-----P--R--------
Mycobacterium scrofulaceum	WP_067280566	--------------R--	LDPVV-D-----P--R--------
Mycobacterium senuense	WP_085083241	--------------R--	VDPVA----C-Q---R----I---
Mycobacterium septicum	WP_044516421	--------------R--	LEEVT-D-----S-------I---
Mycobacterium setense	WP_064876516	------------K-R--	LEKVT-------P-----------
Mycobacterium sherrisii	WP_069399079	--------------R--	LDPVV-D-----P--R--------
Mycobacterium shigaense	BAX91220	--------------R--	VDPVV-D----AS--R--------
Mycobacterium shimoidei	WP_069395339	--------------R-K	IDPVS-D--------R--------
Mycobacterium shinjukuense	WP_083052309	--------------R--	VDPVV------AP--R--------
Mycobacterium simiae	WP_061558431	--------------R--	IDPVC-D----AP--R--------
Mycobacterium sinense	WP_064853526	--------------R--	IDPVA----------R--------
Mycobacterium smegmatis	WP_003893039	------------K-R--	LEPVT-D-----P-----------
Mycobacterium szulgai	WP_085671307	--------------R--	IDPVV-D-----S--R--------
Mycobacterium terrae	WP_085260980	--------------R--	IDPVT----------R----I---
Mycobacterium thermoresistibile	WP_003928094	------------K-R--	IEPVT-------P--R--------
Mycobacterium triplex	CDO87218	--------------R--	IDPVV-D-----S--R--------
Mycobacterium triviale	WP_085111389	--------------R--	LDPVV-D----IP--R--------
Mycobacterium tuberculosis	WP_061140135	--------------R--	VDPVI------AP--R--------
Mycobacterium tusciae	WP_083124873	------------K-R--	EERVT-------A--R--------
Mycobacterium ulcerans	OIN34661	--------------RS-	IDPVV-------S--R--------
Mycobacterium vaccae	WP_003929282	------------K-R--	QEAVT-D----AP--R--------
Mycobacterium vulneris	WP_065458707	--------------R--	LEKVT-D-----S-------I---
Mycobacterium wolinskyi	WP_085146753	------------K-R--	LERVT-------P-----------
Mycobacterium xenopi	ORX14119	--------------R--	VDPVV-D-----Q--R--------

Other *Mycobacterium* (0/>100)	*Mycobacterium yongonense*	WP_065501743	--------------R--	EDPVV-D-----P--R--------
Other bacteria	*Acidimicrobium ferrooxidans*	WP_015799012	-----------L--RA-	EEPVV-P--LA----A--------
	Acidipropionibacterium jensenii	WP_028702933	------A-------RT-	VDPVS-P--ML-PV-RR---I---
	Actinokineospora enzanensis	WP_018682714	--------------K--	KEPIT-D--LL-K--H--K-----
	Actinoplanes awajinensis	WP_067704928	--------------K--	LEKPSVP--L--N--A--------
	Amycolatopsis benzoatilytica	WP_020662677	--------------RQ-	REQ--HD--LLKK--D--------
	Brachybacterium squillarum	WP_010534587	-----------L--RT-	EEPVTFI--LL-KS-AR-------
	Brevibacterium mcbrellneri	WP_040348481	-----E-----L--RQ-	VEPVT-A--ALKNS-G--------
	Cellulomonas fimi	WP_013770896	-----N------E-AH-	RQAVT-L--LL-KS-A--K-----
	Corynebacterium ciconiae	WP_026161506	--------------R--	LEPV-FD--CL-N--G--------
	Gordonia effusa	WP_007319083	--------------R--	-EKANCD-----N--GR-------
	Kitasatospora azatica	WP_051969618	-----------L--RA-	QEAADCP--L-K---D--------
	Nocardia gamkensis	WP_062973261	--------------R--	VEDVV-D--ALKN--ER-------
	Rhodococcus imtechensis	WP_007296649	--------------R-K	LEPVT-D---L-K---R-------
	Saccharopolyspora flava	SFS42319	--------------R-H	EEE-D----L-AG--E--------

Supplementary Figure 25

A partial sequence alignment of a conserved region of error-prone DNA polymerase showing a one amino acid insertion that is specific for members of the "*Abscessus-Chelonae"* clade and absent in other bacteria.

			39		78
“Abscessus-Chelonae” Clade (6/6)	*Mycobacterium abscessus*	WP_052622963	TAPWGHVPQPDFFNAIVVAD		GREPLEWLAVAHPLEQSAQR
	Mycobacterium abscessus subsp. bolletii	EIU58836	--------------------		------------R-------
	Mycobacterium chelonae	WP_070918916	-----P---S-YL--V-I-Q		----R---E---R-------
	Mycobacterium franklinii	WP_070937275	-----P--------VV-I--		----R-------R-------
	Mycobacterium immunogenum	WP_064630595	-----P---S-------I--		-H--R----L-QH-------
	Mycobacterium saopaulense	WP_070912427	-----P---S--L----I-T		----Q---EQ-QR--R----
Other *Mycobacterium* (0/>100)	*Mycobacterium angelicum*	WP_083116336	-D---G-E-GP-L--VLI-E	DP	ACDEHD--RR-QAA-DA-G-
	Mycobacterium aromaticivorans	WP_036341789	-DA--G-E-GP-L--V----	DP	EADGH---RR-QA--NAND-
	Mycobacterium arosiense	WP_083067254	-D---R-D-AP-L--V-L--	DP	SLDGQG--RR-QEF--A-G-
	Mycobacterium arupense	WP_046191459	-EA--G-E-GP-L--VLIVE	DP	-TDGRG--RR--E---A-E-
	Mycobacterium asiaticum	WP_065035951	-D---G-E-EP-L--VL--E	DP	A-DAHA--RW-QE--RA-G-
	Mycobacterium aurum	WP_087027799	-DA--G-E-GS-L--VLI-E	DP	SCDGYG--RRGQR--AA-D-
	Mycobacterium avium	WP_084037761	-Q---R-D-AP-L--VLI--	DP	ACDGQG--RR-QEF-RA-G-
	Mycobacterium avium subsp. avium	EUA40957	-Q---R-D-AP-L--VLI--	DP	ACDGQG--RR-QEF-RA-G-
	Mycobacterium avium subsp. paratuberculosis	ETB09681	-Q---R-D-AP-L--VLI--	DP	ACDGQG--RR-QEF-RA-G-
	Mycobacterium bacteremicum	WP_083058544	-DA--G-D-GA-L--VLI-A	DP	-YDGHG--RHGQA--AA-E-
	Mycobacterium boenickei	WP_077738724	-DA--G-E-GP-L--VLI--	DP	DLDAHG--RRGQQ---A-E-
	Mycobacterium bohemicum	WP_085182816	-D---R-D-AP-L--VLI--	DP	ACDGQG--RR-QAF-RA-G-
	Mycobacterium bovis BCG str. ATCC 35743	AHM09394	---G-E-GQ-L--VLI--	DP	TC--R---RR-EEF-RA-G-
	Mycobacterium branderi	WP_083131143	--A--G-E-GP-L--VLI-E	DP	ALDGQG--HR-QEM-EA-D-
	Mycobacterium canariasense	WP_062655973	-DA--G-E-GP-L--V-I-E	HP	D-S-AD--RLGQEF-DA-G-
	Mycobacterium canettii	WP_015291580	---G-E-EQ-L--VLI--	DP	SCG-RQ--RR-QEF-HA-G-
	Mycobacterium celatum	WP_062540983	--A--G-E-GP-L--VLI-E	DS	ALDGQG--RR-QEM-EA-D-
	Mycobacterium celeriflavum	WP_083004642	-----G-D-GP-L--V-I-E	DP	ALDGRG--RR--E---T-D-
	Mycobacterium chimaera	WP_089152368	-D---R-D-AP-L--VLI--	DA	ACDGQG---R-QEF-RA-G-
	Mycobacterium chlorophenolicum	WP_048468689	-DA--G-E-GA-L--V-L--	DS	S-D-HG--RR--E--DA---
	Mycobacterium chubuense	WP_014817887	-DA--G-E-AP-L--V-I--	DP	ALDSHG--RR--E--DA-G-
	Mycobacterium colombiense	WP_064885203	-D---R-D-AP-L--VLI--	DP	ACDGQG--RR-QEF--A-G-
	Mycobacterium conceptionense	WP_076216167	-DA--G-E-SP-L--VLI--	DP	DLDCHG--RRGQR---E-D-
	Mycobacterium confluentis	WP_085152346	-----G-D-GP-L----I-E	DS	-LDGPG--Q--QQ--RD-D-
	Mycobacterium conspicuum	WP_085235323	-D---ETE-GP-L--VLI--	DP	S-DARA--RR-QEF-RA-G-
	Mycobacterium cosmeticum	WP_036397669	-DA--G-E-GP-L--V-I-E	HP	D-S-AD--RIGQEF-DASG-
	Mycobacterium diernhoferi	WP_073856865	-DA--G-E-GA-L--VLI--	DP	SCDGHG--RRGQR--AA-D-
	Mycobacterium doricum	WP_085188364	-EA--G-E-GA-L--VLI--	DP	D-DCHG--RR-QQ---A-A-
	Mycobacterium engbaekii	WP_085128759	--A--G---GP-L--VL-V-	DP	AADERH--RR--E---A-E-
	Mycobacterium europaeum	WP_085241972	-D---R-D-AP-L--VLI-N	DP	ACDAQG--RR-QDF-RA-G-
	Mycobacterium farcinogenes	WP_036391454	-DA--G-E-SP-L--VLI--	DP	DLDCHG--RRGQR---E-D-
	Mycobacterium flavescens	WP_069415287	-----G-E-GP-L--V---E	DD	ALDAPG--RR--EF--A-E-
	Mycobacterium florentinum	WP_085223255	-----GLE-GP-L--VLI--	DP	ALDGQG--RR-QEF--A-G-
	Mycobacterium fortuitum	WP_064850949	-DA--G-E-SP-L--VLL--	DP	-LDCHG--RRGQQ---E-D-
	Mycobacterium fragae	WP_085198689	--A--G-E-GP-L--V-I-E	DA	ALDGHG--RC-QEM-RA-D-
	Mycobacterium gastri	WP_036415849	-D---GID-DP-L--VLI--	DP	E-DCHA--CRV-E--RA-G-
	Mycobacterium genavense	WP_025736762	-----R-E-AP-L--VLI--	DP	ALDGQG--RR-QEF-AA-G-
	Mycobacterium gilvum	WP_011892317	-DA--G-E-GP-L----L-E	DP	ALDAHG--RR--E--DACG-
	Mycobacterium goodii	WP_049744621	--A--F-D-GP-L--VLL--	DP	ALDGHG--RR-QD--AA-G-
	Mycobacterium gordonae	WP_065043394	-D---G-E-EP-L--VLI-E	DP	D-DAHA--RR--EI--A-G-
	Mycobacterium haemophilum	WP_047316399	-VA--G-E-GS-L--V-I--	GP	AYDAQA--CR-QQ--RK-G-
	Mycobacterium hassiacum	WP_005630713	-E---G-E-GP-L--VLLV-	DP	AIDAHG--RRGRE--EA-E-
	Mycobacterium heckeshornense	WP_048891072	-D---G-D-GP-L--VLI--	DP	ACDGHG--RR-QA--RA-D-
	Mycobacterium heidelbergense	WP_083073740	-D---G-E--P-L--VLI--	DP	A-DGQA--RR-QEF-RA-G-
	Mycobacterium heraklionense	WP_064889246	-EA--G-E-GA-L--VLIV-	DP	AADGEV--RR--E---A-E-
	Mycobacterium hiberniae	WP_085134050	-EA--G---GP-L--VL-V-	DP	AADERH--RR--E---A-E-
	Mycobacterium holsaticum	WP_069405838	-Q---G-A-DH-L--V-I-E	DA	DLDARG--RR-QD--RA-E-
	Mycobacterium houstonense	WP_066902391	-DA--G-E-SP-L--V-I--	DP	DLDAHG--RRGQQ--RE-D-
	Mycobacterium icosiumassiliensis	WP_067976475	-EA--G-E-GP-L--VLIVE	DP	AADERG--RR--E---A-E-
	Mycobacterium indicus pranii	WP_043955590	-D---R-D-AP-L--VLI--	DP	ACDGQG---R-QEF-RA-G-
	Mycobacterium intermedium	WP_069419428	-E---G-E-GP-L--VLI--	DP	DCDEQG--RR-QA--RA-D-
	Mycobacterium intracellulare	WP_064936294	-D---R-D-AP-L--VLI--	DP	A-DGQG---R-QEF-RA-G-
	Mycobacterium kansasii	WP_063473150	-D---GIE-DP-L--VLI--	DP	S-DCHA--CR--EF-RA-G-
	Mycobacterium komanii	CRL75698	-----G-E-GP-L--VL---	DP	-LDAHG--RR--EF-R--E-
	Mycobacterium koreense	WP_085303723	-D---GLD-GQ-L--VLI-E	DP	D-DGHA--RR--E--DA-H-
	Mycobacterium kumamotonense	WP_065287570	-E---V-D-GR-L--VLIVE	DP	ATDEHG--RR--E---A-D-
	Mycobacterium kyorinense	WP_065015117	--A--G-E-GP-L--VLI--	DP	ALDGQG--RC-QEM--A-E-
	Mycobacterium lacus	WP_085157113	-Q---G-E-GP-L--V-I--	DP	TCDGQG--RR-QEF-RA-G-

Other *Mycobacterium* (0/>100)	*Mycobacterium lentiflavum*	CQD21776	-----R-E-GP-L--VLI-- DP ALDGPG--RR-QEF--A-G-
	Mycobacterium leprae	WP_010907610	-V---A-E-RS-L--V-I-- GP AYDTKA--CR-QE--RN-G-
	Mycobacterium lepromatosis	WP_045842336	-VA--G-E-GS-L--VLI-- CP AYDAKA--CW-QE--RN-G-
	Mycobacterium litorale	WP_078021156	-DA--G-E-GP-L--V---- DP ALDGHA--RL-QA---ANE-
	Mycobacterium llatzerense	WP_071287397	-DA--G-E-GP-L--V-L-- AP DLDGPG--RR--EF-NA-D-
	Mycobacterium longobardum	WP_085266474	-EA--G-E-AP-L--VLIVE DP AADGPR--RR-QQ---A-G-
	Mycobacterium mageritense	WP_036432053	--A--G-E-GP-L--VL--- DP ALDGHG--RRGQE---A-G-
	Mycobacterium malmesburyense	CRL78235	-----G-E-GP-L--VLI-E DP ALDGHG--RR--DF-RA-E-
	Mycobacterium malmoense	WP_065445997	-D---R-D-AP-L--VLI-N DP ACDGQC--RR-QEF-RA-G-
	Mycobacterium mantenii	WP_083099006	-D---R-D-AP-L--VLI-- DP A-DGQG--RR-QEF-RA-G-
	Mycobacterium marseillense	WP_083019044	-D---RLD-AP-L--VLI-- DP ACDGPG---R-QEF-RA-G-
	Mycobacterium minnesotense	WP_083025984	-EA--G-E-GP-L--VLIV- DA ATDGHG--RR--E--RA-E-
	Mycobacterium moriokaense	WP_083155142	-D---G-E-GA-L--V-L-- DP TLDAHG--RR-QEF-RG---
	Mycobacterium mucogenicum	WP_064978571	--A--G-E-GP-L--VLL-- DP -LDGPG--RR--GF-NA-D-
	Mycobacterium nebraskense	WP_085165438	-D---R-D-AP-L--VLI-N DP -CDGQC--RR-QEF-RA-G-
	Mycobacterium neworleansense	CRZ17887	-DA--G-E-GP-L--VLI-- DP DVDCHG--RRGQQ---A-D-
	Mycobacterium nonchromogenicum	WP_085138902	--A--G-E-GP-L--VLIV- DP AADGQV--RR--E---A-E-
	Mycobacterium noviomagense	WP_083088574	-E---G-D-GP-L--VLI-- DP ACDAHA--RR-QGF-RA-D-
	Mycobacterium novocastrense	WP_067394100	-----G-E-GP-L--VL--- DP ALDGHG--RR--EF-R--E-
	Mycobacterium palustre	WP_085079450	-D---R-E-AP-L--VLI-G DP TCDGQG--RR-QRF--A-G-
	Mycobacterium paraense	WP_085103184	-D---R-E-AP-L--VLI-Q DP ACDGQA--RR-QEF-RA-G-
	Mycobacterium paraffinicum	WP_073871411	-D---R-D-AP-L--VLI-N DP ACDGQC--RR-QDF-RA-G-
	Mycobacterium parafortuitum	WP_083142026	-DA--G-E-GP-L--V-L-- DP -LDAHG--RR--E--DA-G-
	Mycobacterium paraintracellulare	AFC51989	-D---R-D-AP-L--VLI-- DP ACDGQG---R-QEF-RA-G-
	Mycobacterium paraseoulense	WP_083176178	-D---R-D-AP-L--VLI-N DP ACDGQG--RR-QEF-RA-G-
	Mycobacterium parmense	WP_085267364	-D---R-D-AP-L--VLI-- DP DCDGQG--RR-QQF--A-G-
	Mycobacterium peregrinum	WP_064883947	-DA--G-E-SP-L--VLL-- DP -LDCHD--QRGQR--DE-G-
	Mycobacterium phlei	WP_003888558	-----G-E-DP-L--V---- DP -ADAAA--RR--DF-RA-E-
	Mycobacterium porcinum	WP_075922918	-DA--G-E-SP-L--VLI-E DP -LDCHG--RRGQQ---A-D-
	Mycobacterium rhodesiae	WP_083120774	-DA--G-E-GP-L--V---- DP -ADGH---RR-QA--NAND-
	Mycobacterium riyadhense	WP_085251862	---D-E-GP-L---LIV- DP ACDAQD--RR-QEA-RA-G-
	Mycobacterium rufum	KGI70074	-DA--GIE-GP-L--V-L-- DS A-D-HG--RR--E--DA---
	Mycobacterium rutilum	WP_083406972	-----G-E-DP-L--V-I-E DD ALDAAG--RR--DF--A-E-
	Mycobacterium saskatchewanense	WP_085255276	-D---RLD-AP-L--VLI-- DP ALDGQA--RR-QEF-RA-G-
	Mycobacterium scrofulaceum	WP_067269724	-D---R-D-GP-L--VLI-N DP ACDGQG--RR-QEF-RA-G-
	Mycobacterium senuense	WP_085085789	--G-D-GR-L--VLIVE DP AVDKHG--HR-QE---A-D-
	Mycobacterium septicum	WP_044520879	-DA--G-E-GP-L--VLI-- DP DLDCHG--RRGQQF--E-D-
	Mycobacterium setense	WP_064876365	-DA--G-E-GP-L--V-M-- DP ALDCHG--RRGRQ---E-D-
	Mycobacterium sherrisii	WP_069400805	-E---R-E-GP-L--VLI-- DP ALDGQG--QR-QRF-RA-G-
	Mycobacterium shigaense	BAX94718	-D---R-E-AP-L--VLI-E DP ACDGQA--RR-QGF--A-G-
	Mycobacterium shimoidei	WP_069394739	--A--G-E-GP-L--VLI-E EA TLDAHG--RR-QEM--A-D-
	Mycobacterium simiae	WP_061559900	-----R-D-GP-L--VLI-- DP TLDGQG--GR-QGF-RA-G-
	Mycobacterium sinense	WP_064856062	-E---VLD-GR-L--VLIVE DP AIDEHG--RR--E--DA-D-
	Mycobacterium terrae	WP_085261829	-E---V-E-GR-L--VLIVE DP ATDEHG--RR--E--HA-D-
	Mycobacterium timonense	WP_083187730	-Q---R-D-AP-L--VLI-- DP ACDGQG--RR-QEF-RA-G-
	Mycobacterium triplex	WP_036471830	-----R-E-AP-L--VLI-- DP ALDGQG--RR-QEF-GA-G-
	Mycobacterium triviale	WP_069393159	-D---GLD-GQ-L--VLI-E DP D-DGHA--RR--E--DA-H-
	Mycobacterium tuberculosis	WP_070890648	---G-E-GQ-L--VLI-- DP TC--R---RR-QEF-RA-G-
	Mycobacterium tusciae	WP_083126217	-D---G-E-GA-L--V-I-- DP ALDARG--RR-QEF-RA-G-
	Mycobacterium vulneris	WP_085290845	-D---R-D-AP-L--VLI-- DP ACDGRG--RR-QEF-RA-G-
	Mycobacterium wolinskyi	WP_067850384	--A--G-E-GP-L--VLL-- DP DLDGHG--RRGQE---A-G-
	Mycobacterium xenopi	WP_003921684	-D---G-D-GP-L--VLI-- DP ACDGHG--RR-QA--RA-D-
	Mycobacterium yongonense	WP_065498839	-D---R-D-AP-L--VLI-- DP ACDGQG---R-QEF-RA-G-
Other bacteria	*Actinomycetospora chiangmaiensis*	WP_018333188	-----G-D-E--L-LV-TVE DP EAG-WV---R-RSA--ARD-
	Cryptosporangium aurantiacum	WP_073254398	-D---P-A-DNYL----I-- DP --DAAG--SFVRAC--A-G-
	Kibdelosporangium aridum	SMD00169	-----VTD-D--L----I-S RE DFGEWD--RL-QD--NA-G-
	Kibdelosporangium phytohabitans	WP_054287867	-----VTD-D--L------S HE DFDEWD--RR--E--NA-G-
	Micromonospora echinospora	SCE99188	-P---DTD--TYL--VLL-E DP SATARD--DR-RAA-L--G-
	Micromonospora halophytica	SCG56521	-P---DAD--AYL--A-L-L DP AASARD--TR-RAA--A-D-
	Micromonospora rosaria	WP_067369687	-P---DED--AYL--VLL-A DP TATARD--DR--AA-RA-G-
	Nakamurella multipartita	WP_015746179	-P---G-----YY-LV-I-E DD -NDAHA-WERCQA---E-G-
	Nocardia acidivorans	WP_067568932	-P---G---Q-YL--VL--Q DP NL--SD--RLGQE---A-D-
	Nocardia africana	WP_062962127	-----G---E-YL--V--VQ DS ELG-RD--ERGQR---RSG-
	Nocardia alba	WP_067454263	-----G-D-D-YL--V-I-E DP AFDDYA--RFGQE---A-D-
	Nocardia altamirensis	WP_069165907	-----G-E-D-YL-----VE DP ELDRAD--RLGQQ---A-D-

Other bacteria	*Nocardia amamiensis*	WP_067476098	-----D---G-YL--A-LVE DP AFGCAD--RRGQE---A-G-
	Nocardia amikacinitolerans	WP_067789988	-----G-D-D--L--V-LV- DP ELDC-G--RRGQEF-RA-H-
	Nocardia anaemiae	WP_062991498	-----G-E-D-YL----LV- DP EFGAHD--RFGQQ---A-G-
	Nocardia arizonensis	WP_054816726	-----G-E-D-YL--V-LVN DP -Y-CRD--RAGQR--RA-E-
	Nocardia asiatica	WP_043718530	-----G---D-YL--V-L-E DP AFGCVD--RRGQD---A-G-
	Nocardia brasiliensis	WP_014981272	-----G-E-D-YL------E DP ELGC-D--RRGQA---A-E-
	Pseudonocardia dioxanivorans	WP_013678115	-----GIE----L--VI-V- DP -TDAWG--RRGQR---A-H-
	Rhodococcus equi	WP_064077718	-----G-E-Q--L-----V- DP -TDAHG--RRGQE--A--D-
	Saccharomonospora xinjiangensis	WP_006238898	-----VSD----L--VC-VE DP A-DHWA--RTGQD---R-G-
	Saccharopolyspora antimicrobica	SFO30961	-----VTD----L--VL-VE SP ELDEWG--RRGQQ---Q-E-
	Saccharopolyspora flava	SFS57149	-----VTD----L----L-E SP DLDEWD--RR-QA---R-E-
	Tsukamurella paurometabola	WP_013125245	-P---G-E-D--I--T-L-- DP S-TAAG--DF-RDQ-AA-D-

Supplementary Figure 26

A partial sequence alignment of a conserved region of 2-amino-4-hydroxy-6-hydroxymethyldihydropteridine diphosphokinase showing a two amino acid deletion that is specific for members of the "*Abscessus-Chelonae*" clade and absent in most other bacteria.

			253		295
"Abscessus-Chelonae" Clade (6/6)	*Mycobacterium abscessus*	WP_052619503	DGSPHGMRTVALWEPPLL		PITGENGAPVRRPVSTETSRLLADL
	Mycobacterium abscessus subsp. bolletii	EIU84465	------------------		-------------------------
	Mycobacterium chelonae	WP_070918860	------------------		-----------------A-------
	Mycobacterium franklinii	WP_070937318	------------------		-------------------------
	Mycobacterium immunogenum	WP_064630665	------------------		-------------------------
	Mycobacterium saopaulense	WP_070912379	----------V-------		-------------------------
Other *Mycobacterium* (0/>100)	*Mycobacterium acapulcensis*	WP_066810233	----Q-A-----------	D	DVV---------SAGA-AASVM---
	Mycobacterium africanum	WP_031669502	----R-A--------A-R	S	DVI--H------SAGA-AA-VM---
	Mycobacterium algericum	WP_083040188	------A--------A-R	G	DM----------AAG--AA-VM---
	Mycobacterium alsense	WP_083138395	----Q-A--------A-R	T	DL----------SAGA-AA-VM---
	Mycobacterium angelicum	ORA13096	----Q-A--------A--	T	DLI---------SAGA-AA-VM---
	Mycobacterium aromaticivorans	WP_036341816	------A--------E-R	T	DL----------SAGA--A-MM---
	Mycobacterium arosiense	WP_083066593	--A-Q-A--------A-R	A	DL----------SAGA-AA-VM---
	Mycobacterium arupense	WP_046686428	------A--------A-R	T	DV---H------AAGS-AA-VM---
	Mycobacterium asiaticum	WP_065035925	----Q-A--------A--	T	EL----------SAGA-AA-VM---
	Mycobacterium aurum	WP_087027852	----Q-G--------A--	P	DLV---------SAGA-A--VM---
	Mycobacterium avium	WP_062889758	----Q-A--------A-R	A	DLV---------SAGA-AA-VM---
	Mycobacterium avium subsp. avium	EUA38322	M-----A-R	A	DLV---------SAGG-AA-VM---
	Mycobacterium bacteremicum	WP_083058651	------G--------A--	D	DLV---------SAGA-SA-VM---
	Mycobacterium bohemicum	WP_085182367	----Q-A--------A-R	T	DLL---------SAGA-AA-VM---
	Mycobacterium bovis	WP_024458578	----R-A--------A-R	S	DVI--H------SAGA-AA-VM---
	Mycobacterium branderi	WP_083131257	----Q-G----------R	D	DL----------SAGA-AA-VM---
	Mycobacterium brisbanense	WP_062828552	-A--Q-A--I-----A--	P	DL----------SAGA-AA-VM---
	Mycobacterium canariasense	GAS94787	----Q-G--------A--	D	DL----------SAGA-AA-VM---
	Mycobacterium canettii	WP_014001862	----R-A--------S-R	S	DVI--H------SAGA-AA-VM---
	Mycobacterium celatum	WP_085168566	-S--Q-G----------R	D	DL----------SAGA-AA-VM---
	Mycobacterium celeriflavum	WP_083000273	----Q-A-----------	E	DAV---------SAGA-AAGVM---
	Mycobacterium chlorophenolicum	KMO83561	----Q-A--------A-I	A	DLV---------SAGA-A--VM---
	Mycobacterium chubuense	WP_014817919	----Q-A--------A-I	D	DLV---------SAGS-AA-VM---
	Mycobacterium colombiense	WP_064952580	----Q-A--------A-R	A	DL----------SAGA-AA-VM---
	Mycobacterium conceptionense	WP_064898695	----Q-A--I-----A--	D	DL----------SAGA-AA-VM---
	Mycobacterium confluentis	WP_085152372	----Q-E----------R	T	DV---H------TAGS-AA-VM---
	Mycobacterium conspicuum	WP_085232729	----Q-A--------A-R	A	EV----------SAGG-AA-VM---
	Mycobacterium cosmeticum	CDO07616	----Q-G--------A--	D	DL----------SAGA-AA-VM---
	Mycobacterium diernhoferi	WP_073856897	----Q-G--------A--	P	ELV---------SAGA-A--VM---
	Mycobacterium doricum	WP_085188305	----Q-A--------A--	T	DLV---------SAGA-AA-VM---
	Mycobacterium elephantis	WP_046754022	----Q-A-----------	L	DLV---------SAGA-AAQVM-R-
	Mycobacterium engbaekii	WP_085128723	------A--------A-R	T	DL----------AA---AA-VM---
	Mycobacterium europaeum	WP_085241465	----Q-A--------A-R	T	DLL---------SAGA-AA-VM---
	Mycobacterium fallax	WP_085094970	------T----------R	T	DV---Q------SAGA-AATVM-G-
	Mycobacterium farcinogenes	WP_036391363	----Q-A--I-----A--	D	DL-------A--SAGA-AA-VM---
	Mycobacterium flavescens	WP_069415311	----Q-A-----------	D	DLV---------SAGA-AASVM---
	Mycobacterium florentinum	WP_085223179	----Q-E--------A-R	T	DV---H------SAGV-AA-VM---
	Mycobacterium fortuitum	WP_061265018	----Q-A--I-----A--	E	DL----------SAGA-AA-VM---
	Mycobacterium fragae	WP_085200165	----Q-A----------R	A	DL----------SAGA-AA-VM---
	Mycobacterium gastri	WP_036411211	----Q-A--------A-R	T	DL----D-----SAGA-AA--M---
	Mycobacterium genavense	WP_036467609	----Q-E--------A-R	T	DV---H------SAGV-AA-VM---
	Mycobacterium gilvum	WP_011892289	----Q-A--I-----A-I	P	DLL---------SAGA-AA-VM---
	Mycobacterium goodii	WP_049743430	----Q-A--I-----A--	P	DLA---------SAGS-AG-VM---
	Mycobacterium gordonae	WP_065043626	----Q-A--------A--	T	DLI---------SAGA-A--VM---
	Mycobacterium haemophilum	WP_047316419	----Q-A--------A-R	A	DLA---------SAGA-AA-VM---
	Mycobacterium hassiacum	WP_005630779	------A--------A--	D	DLI---------SAGA-AA-VMG--
	Mycobacterium heckeshornense	WP_071700317	----R-A--------A-R	P	EV----------SAGA-AA--M---
	Mycobacterium heidelbergense	WP_083073760	----Q-A--------A-R	A	DA----------SAGA-AA-VM---
	Mycobacterium heraklionense	OBI05843	------A--------A-R	T	DV----------AAGS-AA-VM---
	Mycobacterium hiberniae	WP_085134077	------A--------A-R	T	DL----------AAGS-AA-VM---
	Mycobacterium holsaticum	WP_069404804	----Q-A-----------	S	DLV---------SAGA-AAQVM-N-
	Mycobacterium houstonense	WP_066902358	----Q-A--I-----A--	D	DL----------SAGA-AA-VM---
	Mycobacterium icosiumassiliensis	WP_067976540	------A--------A-R	T	DM----------AAG--AA-VM---
	Mycobacterium indicus pranii	WP_014941196	----Q-A--------A-R	T	DLV---------SAGA-AA-VM---
	Mycobacterium insubricum	ORA73401	------A----------R	T	D--------L---AG--AAAMM-E-
	Mycobacterium interjectum	WP_085203350	----Q-A--------A-R	A	DL----------SAGA-AA-VM---
	Mycobacterium intermedium	WP_069419405	----Q-A--------A-R	D	DLV---------SAGA-AA-IM---
	Mycobacterium intracellulare	WP_064938669	----Q-A--------A-R	A	DLV---------SAGA-AA-VM---

Other *Mycobacterium* (0/>100)

Mycobacterium iranicum	WP_064279803	----Q-G--------A-- P DLL---------SAGA-AA-VM---
Mycobacterium kansasii	WP_063468662	----Q-A--------A-R T DL----------SAGA-AA--M---
Mycobacterium komanii	CRL74116	----Q-A----------- D DLV---------SAGA-AASVM---
Mycobacterium koreense	WP_085303742	----Q-A--------A-S A EPA---------SAGA-AA-VM---
Mycobacterium kubicae	WP_085074663	------A--------A-R T DLL---------SAGA-AA-VM---
Mycobacterium kumamotonense	WP_065289400	------A--------A-R T DL----------AAG--AA-VM---
Mycobacterium kyorinense	WP_065012743	----Q-A--------A-R A DLI---------SAGA-AA--M---
Mycobacterium lacus	WP_085158513	----Q-A--------A-R A DL----------SAGA-AA-VM---
Mycobacterium lentiflavum	CQD21870	----Q-E--------A-R T DV---H------SAGV-AA-VM---
Mycobacterium llatzerense	WP_071287413	------G--------A-- T DLV---------SAG--A--VM---
Mycobacterium longobardum	WP_085265468	------A--------A-R T DV----------AAGA-AA-VM---
Mycobacterium mageritense	WP_036431925	----Q-A--I-----A-- P ELS---------SAGA-AA-VM---
Mycobacterium malmesburyense	CRL79198	----Q-A----------- D DLV---------SAGA-AASVM---
Mycobacterium malmoense	WP_065445877	----Q-A--I-----A-R A DLL---------SAGA-AA-VM---
Mycobacterium mantenii	WP_083099018	----Q-A--------A-R A DL----------SAGA-AA-VM---
Mycobacterium marinum	WP_012396657	----Q-A--------A-R T DL----------SAG--AA-VM---
Mycobacterium marseillense	WP_083019101	----Q-A--------A-R A DLV---------SAGA-AA-VM---
Mycobacterium minnesotense	WP_083026080	------A--------A-R T DV---H------AAG--AA-VM---
Mycobacterium monacense	WP_083044869	----Q-A--------A-- T DLV---D-----SAGA-AA-VM---
Mycobacterium moriokaense	WP_083155244	----Q-A--------A-- D DLV---------SAGADAA-VMG--
Mycobacterium mucogenicum	WP_064857692	------G--------A-- T DLI---------AAG--A--VM---
Mycobacterium mungi	WP_064319869	----R-A--------A-R S DVI--H------SAGA-AA-VM---
Mycobacterium nebraskense	WP_047322472	----Q-A--------A-R A DLL---------SAGA-AA-VM---
Mycobacterium neoaurum	WP_042510092	------G--------A-- E DLV---------SAGA-SA-VM---
Mycobacterium neworleansense	CRZ17828	----Q-A--I-----A-- D DL----------SAGA-AA-VM---
Mycobacterium nonchromogenicum	WP_085138880	------A--------A-R T DV----------AAGS-AA-VM---
Mycobacterium noviomagense	ORB12884	----Q-A--------A-R P DL----------SAGA-AA--M---
Mycobacterium novocastrense	WP_067394144	----Q-A----------- D DLV---------SAGA-AASVM---
Mycobacterium obuense	WP_046364512	----Q-A--------A-- T DLV---------SAGA-A--VM---
Mycobacterium palustre	WP_085079482	----Q-S--------A-- A DLV---------SAGA-AA-VM---
Mycobacterium paraense	WP_085103371	----Q-A--------A-R A DL----------SAGA-AA-VM---
Mycobacterium paraffinicum	WP_073871754	----Q-A--------A-R P DLL---------SAGA-AA-VM---
Mycobacterium parafortuitum	WP_083142048	----Q-G--I-----A-I P DLH---------SAGA-A--VM---
Mycobacterium paraintracellulare	WP_014383731	----Q-A--------A-R T DLV---------SAGA-AA-VM---
Mycobacterium paraseoulense	WP_083175960	----Q-A--------A-R A DLL---------SAGA-AA-VM---
Mycobacterium parmense	WP_085267341	----Q-S--------A-R T DLI--------SSAGA-AA-VM---
Mycobacterium peregrinum	WP_064884006	----Q-A--I-----A-- D DL----------SAGA-AA-VM---
Mycobacterium phlei	WP_003888586	----Q-A----------- D ELV---------SAGA-AAGVM---
Mycobacterium porcinum	WP_069424684	----Q-A--I-----A-- D DLA---------SAGA-AA-VM---
Mycobacterium pseudoshottsii	WP_086084797	----Q-A--------A-R T DL----------SAG--AA-VM---
Mycobacterium rhodesiae	WP_014210567	----Q-A--I-----A-- D DLV---------SAGADAA-VMGN-
Mycobacterium riyadhense	WP_085252092	----Q-A--------A-R A DL----------SAGA-AA-VM---
Mycobacterium rufum	KGI71169	----Q-A--------A-I D DLV---------SAGA-A--VM---
Mycobacterium rutilum	WP_083406942	----Q-A----------- E DLV---------SAGA-AASVM---
Mycobacterium saskatchewanense	WP_085255399	----Q-A--------A-R A DL----------SAGA-AA-VM---
Mycobacterium scrofulaceum	WP_067270667	----Q-A--------A-R P DLL---------SAGA-AA-VM---
Mycobacterium senuense	WP_085085640	------A--I-----A-R T DV----------AAG--AA-VM---
Mycobacterium septicum	WP_044520967	----Q-A--I-----A-- D DL----------SAGA-AA-VM---
Mycobacterium setense	WP_064872165	----Q-A--I-----A-- T DLI---------SAGA-AA-VM---
Mycobacterium sherrisii	WP_069400776	----Q-A--------A-R T DV---H------SAGG-AA-VM---
Mycobacterium shigaense	BAX94751	----Q-A--------A-R A DL----------SAGA-AA-VM---
Mycobacterium shimoidei	WP_069394707	----Q-G--------A-R A DLI---------SAGS-AA-VM---
Mycobacterium shinjukuense	WP_083051292	----Q-A--------A-R A DL----------SAGA-AA-VM---
Mycobacterium simiae	WP_061559910	----Q-A--------A-R T DV---H------SAGG-AA-VM---
Mycobacterium sinense	WP_064856032	----R-A--------A-R T D-----------AAG--AA-VM---
Mycobacterium smegmatis	WP_003897559	----Q-A--I-----A-- P DLA---------SAGS-AG-VM---
Mycobacterium szulgai	WP_068023072	------A--------A-R T DLL---------SAGA-AA-VM---
Mycobacterium terrae	WP_085260313	------A--------A-R T DL----------AAG--AA-VM---
Mycobacterium thermoresistibile	WP_040546641	-A----A--I-------- P DLV----------AGA-AA-VM---
Mycobacterium timonense	WP_083186932	----Q-A--------A-R A DLV---------SAGG-AA-VM---
Mycobacterium triplex	WP_036471857	----Q-E--------A-R T DV---H------SAGV-AA-VM---
Mycobacterium triviale	WP_069393103	----Q-A--------A-S A EPA---------SAGA-AA-VM---
Mycobacterium tuberculosis	WP_070891510	----R-A--------A-R S DVI--H------SAGA-AA-VM---
Mycobacterium tusciae	WP_083126243	----Q-A--------A-- D D-V---------SAGADAAQVMGN-
Mycobacterium ulcerans subsp. shinshuens	BAV39686	----Q-A--------A-R T DL----------SAG--AA-VM---

Group	Species	Accession	Sequence
**Other *Mycobacterium* (0/>100)**	*Mycobacterium vaccae*	WP_003931739	----Q-A--------A-M A DLV---------SAGA-AA-VM---
	Mycobacterium vulneris	WP_065462807	----Q-A--I-----A-- D DL----------SAGA-AA-VM---
	Mycobacterium wolinskyi	WP_085143432	----Q-A--I-----A-- A DL----------SAGA-AA-VM---
	Mycobacterium xenopi	WP_085193079	----Q-A--------A-R R DL----------SAGA-AA--M---
	Mycobacterium yongonense	WP_065500419	----Q-A--------A-R T DLV---------SAGA-AA-VM---
**Other *Corynebacteriales***	*Corynebacterium halotolerans*	WP_015399784	--A-T-A--------GF- E GVE---------AAT--AAEIM-T-
	Corynebacterium jeikeium	WP_049193090	--A-V-E--I-----G-- P DLQ----------AP--AADIM---
	Gordonia neofelifaecis	WP_009679430	-A----E----M---GF- D EV----D-----SAGS-AA-----F
	Gordonia otitidis	WP_039993691	----R-E--------DF- P GV----N-----SAGA-SA-----F
	Nocardia asiatica	WP_043718477	----Q-P----------- T AV----------AAT--AA-IM---
	Nocardia beijingensis	WP_067811190	----Q-P--I-------- T AM-----------ATA-AA-IM---
	Rhodococcus fascians	WP_052047340	------A----------- K E----------KSAGA-A--IM---
	Rhodococcus gordoniae	WP_064062801	------P----M------ R EV---H------AAG--AA------
	Tsukamurella paurometabola	WP_049826019	-S----P---V------I P DLE---------QAT--AA-MM---
	Tsukamurella pseudospumae	WP_068744180	-S----P---V------- P DLE----------AT--AA-M----

Supplementary Figure 27

A partial sequence alignment of a conserved region of DEAD/DEAH box helicase showing a one amino acid deletion that is specific for members of the "*Abscessus-Chelonae"* clade and absent in other *Corynebacteriales*.

Group	Species	Accession	51–70	Box	71–90
			51		90
"Abscessus-Chelonae" Clade (6/6)	*Mycobacterium abscessus*	WP_052620306	RLAQSLGIEALGNTPQQVQI		EGLKGELYAMMLDMRRTFDE
	Mycobacterium abscessus subsp. bolletii	EHM22982	--------------------		--------------------
	Mycobacterium chelonae	WP_070930228	------------------H-		--I-----------------
	Mycobacterium franklinii	WP_070937349	---------D--------N-		--------------------
	Mycobacterium immunogenum	WP_043078488	--------------------		-------F------------
	Mycobacterium saopaulense	WP_070912333	-------------V-----V		D------F------------
Other *Mycobacterium* (0/>100)	*Mycobacterium africanum*	WP_013989025	----A---ND------R-PL	A	PEVP---H------------
	Mycobacterium aromaticivorans	WP_036341845	----A----E------R-PL	A	PEVT----------------
	Mycobacterium asiaticum	WP_065034127	----A--VND------R-PL	A	PEVP---H------------
	Mycobacterium aurum	WP_048633972	----A---RD------R-PL	P	PEVT---H------------
	Mycobacterium avium	WP_062894517	----A--VND------R-PL	A	AEVP---H------------
	Mycobacterium bohemicum DSM 44277	CPR11049	----A--VND------R-PL	A	PEVP---H------------
	Mycobacterium brisbanense	WP_062828408	----A---KD------R-PL	A	PEVT---H------------
	Mycobacterium canettii	WP_014001883	----A---ND------R-PL	A	PEVP---H------------
	Mycobacterium celatum	WP_062539461	----A---ND---S--R-PL	A	PEVP-Q--------------
	Mycobacterium chlorophenolicum	WP_048471471	----A---KT------R-PL	A	PEVP---H------------
	Mycobacterium chubuense	WP_014817958	----A---RD---S--R-PL	A	PEVT---H------------
	Mycobacterium colombiense	OBJ31014	----A--VSD------R-PL	A	AEVP---H------------
	Mycobacterium cosmeticum	WP_036397715	----A----S------R-PL	A	PEVP---H------------
	Mycobacterium europaeum	CQD05135	----A--VND------R-PL	A	PEVP---H------------
	Mycobacterium flavescens	WP_069415334	----A---KD------R-PL	A	PEVS---H------------
	Mycobacterium gastri	WP_036413776	----A---DD------R-PL	A	PEVP---HG-----------
	Mycobacterium gilvum	WP_011892265	----A----S------R-PL	A	PEVT---H------------
	Mycobacterium goodii	WP_049743455	----A---KD------R-PL	A	PEVT---H------------
	Mycobacterium gordonae	WP_065046076	----A--VND------R-PL	A	PEVP---H------------
	Mycobacterium haemophilum	WP_047316660	----A--VND------R-PL	A	PEVP---H------------
	Mycobacterium hassiacum	WP_005630825	----A---ND------R-PL	A	PEVP---H------------
	Mycobacterium heckeshornense	WP_048891473	----A---ND---S--R-PL	A	AEVP----------------
	Mycobacterium heraklionense	WP_064997560	----A----D---Y--R-PL	P	AEVP-R-H------------
	Mycobacterium holsaticum	WP_069405273	----A---AD------R-PL	A	PEVR---H------------
	Mycobacterium houstonense	WP_066902344	----A---KD------R-PL	A	PEVT---H------------
	Mycobacterium interjectum	WP_066916637	----A--VND------R-PL	A	PEVS---H------------
	Mycobacterium intermedium	WP_069418002	----A--VND------R-PL	A	PEVP---H------------
	Mycobacterium intracellulare	WP_064938646	----A--VND------R-PL	A	AEVS---H------------
	Mycobacterium iranicum	WP_064279910	----A---RS------R-PL	A	PEVT---H------------
	Mycobacterium kansasii	KZS62795	----A---DD------R-PL	A	PEVP---HG-----------
	Mycobacterium kumamotonense	WP_065289544	----A---DD---A--R-PL	A	PEVP---H------------
	Mycobacterium kyorinense	WP_065015377	----A---ND---S--R-PL	A	PEVP-Q--------------
	Mycobacterium lentiflavum	CQD21966	----A--VND------R-PL	A	PEVT---H------------
	Mycobacterium leprae	WP_010908821	----A--VND------R-PL	A	PEVP---H------------
	Mycobacterium lepromatosis	WP_045843733	----A--VND-D----R-PL	A	PEVP---H------------
	Mycobacterium mageritense	WP_036431834	----A---KD------R-PL	A	PEVS---H------------
	Mycobacterium malmoense	WP_065443475	----A--VND------R-PL	A	PEVR---H------------
	Mycobacterium marinum	WP_036456740	----A--VND---V--R-PL	A	PEVS---H------------
	Mycobacterium mucogenicum	WP_064982879	----A---KD------R-PL	A	PEVP----------------
	Mycobacterium nebraskense	WP_046182776	----A--VND------R-PL	A	SEVP---H------------
	Mycobacterium neoaurum	WP_030132808	----A----S---N--R-PL	A	PEVT---H------------
	Mycobacterium neworleansense	CRZ17796	----A---KD------R-PL	A	PEVT---H------------
	Mycobacterium novocastrense	GAT11557	----A---KT------R-PL	A	PEVS---H------------
	Mycobacterium obuense	WP_046365146	----A---KD---V--R-PL	A	PEVS---H------------
	Mycobacterium parascrofulaceum	WP_007166635	----A--VND------R-PL	A	PEVR---H------------
	Mycobacterium peregrinum	WP_064881196	----A---KD------R-PL	A	PEVT---H------------
	Mycobacterium phlei	WP_003888611	----A---KD------R-PL	A	PEVP---H------------
	Mycobacterium rhodesiae	WP_005138617	----A---KE------R-PL	A	PEVT---H------------
	Mycobacterium rufum	KGI70115	----A---KD------R-PL	A	PEVS---H------------
	Mycobacterium rutilum	SEH60280	----A---KD---V--R-PL	A	PEVS---H------------
	Mycobacterium scrofulaceum	WP_067270749	----A--VND------R-PL	A	PEVP---H------------
	Mycobacterium septicum	WP_044521044	----A---KD------R-PL	A	PEVT---H------------
	Mycobacterium setense	WP_039325842	----A---KD------R-PL	A	PEVT---H------------
	Mycobacterium sherrisii	WP_069400754	----A--VND------R-PL	A	PEVS---H------------
	Mycobacterium shimoidei	WP_069394684	----A---ND------R-PL	A	PEVT-Q-F------------
	Mycobacterium simiae	WP_061557441	----A--VND------R-PL	A	PEVS---H------------
	Mycobacterium sinense	WP_013830880	----A---DD---S--R-PL	A	PEVP----------------
	Mycobacterium smegmatis	WP_003897589	----A---KD------R-PL	A	PEVT---H------------

Group	Species	Accession	Sequence
**Other *Mycobacterium* (0/>100)**	*Mycobacterium szulgai*	WP_068034477	----A--VND------R-PL A PEVT---H------------
	Mycobacterium thermoresistibile	WP_003925222	----A---DD------R-PL A PEVP---H------------
	Mycobacterium triplex	WP_036465145	----A--VND------R-PL A PEVS---H------------
	Mycobacterium triviale	WP_069393121	----A----D------R-PL P DEVP---H------------
	Mycobacterium tuberculosis	WP_063738144	----A---ND---A--R-PL A PEVP---H------------
	Mycobacterium tusciae	WP_006243875	----A---KD------R-PL A PEVT----------------
	Mycobacterium ulcerans Agy99	ABL06283	----A--VND---V--R-PL A PEVS---H------------
	Mycobacterium vaccae	WP_003931713	----A---RD---N--R-PL A PEVT---H------------
	Mycobacterium vanbaalenii PYR-1	ABM16210	----A---RD------R-PL A PEVT---H------------
	Mycobacterium vulneris	WP_065514556	----A---KD------R-PL A PEVT---H------------
	Mycobacterium wolinskyi	WP_067850354	----A---KD------R-PL A PEVT---H------------
	Mycobacterium xenopi	WP_003921609	----A---ND---S--R-PL A PEVP----------------
	Mycobacterium yongonense	WP_020821442	----A--VND------R-PL A AEVE---H------------

Supplementary Figure 28

A partial sequence alignment of a conserved region of an anion transporter showing a one amino acid deletion that is specific for members of the "*Abscessus-Chelonae"* clade and absent in other *Mycobacterium*.

Clade	Species	Accession	331		379
**"*Abscessus-Chelonae*" Clade (6/6)**	*Mycobacterium abscessus*	WP_005081027	ATRDAYGLTDDVVTYRDYSGT	ASSPAG	SQQLAKQVAADRSTIANIRVLD
	Mycobacterium abscessus subsp. bolletii	EUA67093	---------------------	------	----------------------
	Mycobacterium immunogenum	WP_064628010	-----------I---------	--A---	----------------------
	Mycobacterium franklinii	WP_070938762	---------------------	--A-T-	-E--------------------
	Mycobacterium chelonae	WP_070927239	---------------------	--A-T-	-E--------------------
	Mycobacterium saopaulense	WP_070909629	---------------------	--A-T-	-E--------------------
**Other *Mycobacterium* (0/>100)**	*Mycobacterium acapulcensis*	WP_083997650	---Q---------S----P-N		APAT-A------A-TS------
	Mycobacterium africanum	WP_013988703	---E--RIGG-W-Q--S-P-I		GTKQPRD-PV-VT---KV-L--
	Mycobacterium algericum	WP_083035837	---H-----E------N---D		ARAT-A--G--AA-TS---L--
	Mycobacterium alsense	WP_083140568	---Q-----S---S--N-T-D		G-AT-Q------A-TS---L--
	Mycobacterium angelicum	WP_083111069	---Q-----S------N-T-D		G-AT-Q------A-TS---L--
	Mycobacterium aromaticivorans	WP_036340069	---Q-----S-H---------		APTT-Q---S--A-TS------
	Mycobacterium arosiense	WP_083065534	---Q-----S-Q----N-T-D		A-AT-Q---D--A-TS---L--
	Mycobacterium arupense	WP_046188452	---Q-----E--------R-D		ARAT-A-IG--AA-TS---L--
	Mycobacterium asiaticum	WP_065035308	---Q-----P------N-T-D		G-AT-Q------A-TS---L--
	Mycobacterium aurum	WP_087020426	---H-----------------D		AAAT-Q------A-TS---L--
	Mycobacterium avium	WP_062887272	---Q-----S------N-T-D		A-AT-Q---D--A-TS---L--
	Mycobacterium avium subsp. avium	EUA28116	---Q-----A------N-T-D		A-AT-Q---D--A-TS---L--
	Mycobacterium avium subsp. hominissuis	ETB32463	---Q-----S------N-T-D		A-AT-Q---D--A-TS---L--
	Mycobacterium avium subsp. paratuberculosis	ETB52791	---Q-----S------N-T-D		A-AT-Q---D--A-TS---L--
	Mycobacterium avium subsp. silvaticum	ETB14850	---Q-----S------N-T-D		A-AT-Q---D--A-TS---L--
	Mycobacterium bacteremicum	WP_083057290	----------QT-E--N-G-D		ANAT-Q--SS-TA-TS---L--
	Mycobacterium boenickei	WP_077743615	---Q-------T----N-ESN		G-TT-A------A-TS---L--
	Mycobacterium bohemicum	WP_085181997	---Q------------N-T-D		APAN-Q---T--A-TS---L--
	Mycobacterium bovis	WP_086449511	---E--RIGG-W-Q--S-P-I		GTKQPRD-PV-VT---KV-L--
	Mycobacterium branderi	WP_083129858	---Q---------S--N---D		GRAT-E---S--A-TS---L--
	Mycobacterium brisbanense	WP_062831658	---E-----S-T----N-ESN		G-TT-A------A-TS---L--
	Mycobacterium canariasense	GAS93131	---Q------QT--------N		--AT-Q------A-TS---L--
	Mycobacterium canettii	WP_014000016	---E--RIGG-W-Q--S-P-I		GTKQPRD-PV-VT---KV-L--
	Mycobacterium caprae	WP_054938513	---E--RIGG-W-Q--S-P-I		GTKQPRD-PV-VT---KV-L--
	Mycobacterium celatum	WP_085167690	---Q-----E---S--N---D		GRAT-E---S--A-TS---L--
	Mycobacterium celeriflavum	WP_083006383	---Q------------N---D		-PAT-Q------A-TS---L--
	Mycobacterium chlorophenolicum	KMO75863	---Q-----EQQ-S----A-N		APAS-Q------A-TS------
	Mycobacterium chubuense	WP_041781791	---Q-----EKE-S----A-N		APAS-Q------A-TS------
	Mycobacterium colombiense	WP_064877082	---Q-----S-Q----N-T-D		A-AT-Q---D--A-TS---L--
	Mycobacterium conceptionense	CQD04555	---Q-----S-T----N-ESS		G-TT-A------A-TS---L--
	Mycobacterium confluentis	WP_085148630	---Q------------N---D		APTS-Q-I-S--A--S---L--
	Mycobacterium conspicuum	WP_085231379	---Q-----E---S--N-N-D		APAT-Q---T--A-TS---L--
	Mycobacterium cosmeticum	CDO10255	---Q------AT--------N		--AT-Q------A-TS---L--
	Mycobacterium diernhoferi	WP_073857814	---H---------S------D		AAAT-Q------A-TS---L--
	Mycobacterium doricum	WP_085190019	---Q--------------P-D		APAT-Q------A-TS------
	Mycobacterium elephantis	WP_083042562	----------E-----S---D		A-AT-Q------A-TS---L--
	Mycobacterium engbaekii	WP_085129314	---Q-----E--------R-D		ARAT-A--G--AA-TS---L--
	Mycobacterium europaeum	WP_085241601	---Q-----S------T-A-D		T-AT-Q---D--A-TS---L--
	Mycobacterium fallax	WP_085092481	---------EEH--------D		TPTT--EI----A-VS------
	Mycobacterium farcinogenes	CDP84269	---Q-----S-T----N-ESS		G-TT-A------A-TS---L--
	Mycobacterium flavescens	WP_069416781	---Q------E--S----P-N		AEAT-A------A-TS------
	Mycobacterium florentinum	WP_085223062	---Q-----S-Q----N---D		G-AT-Q------A-TS---L--
	Mycobacterium fortuitum	WP_061262691	---Q-----N-T----N-ESN		G-TT-A------A-TS---L--
	Mycobacterium fragae	WP_085195828	---Q---------S--N---D		--AT-A------A-TS---L--
	Mycobacterium gastri	WP_085104988	---Q-----S---A--N-T-E		G-AT-Q------A-TS---L--
	Mycobacterium genavense	WP_025735717	---Q-----S-Q----N---D		--AT-Q------A-TS---L--
	Mycobacterium gilvum	WP_011895490	---Q-----EES------P-N		ASAT-Q------A-TS------
	Mycobacterium goodii	WP_049747760	---N------ET----N-EN-		G-TT-A------A-TS---L--
	Mycobacterium gordonae	WP_065047223	---Q-----P------N-T-D		G-AT-Q------A-TS---L--
	Mycobacterium haemophilum	WP_047313259	---Q-----E------N-T-D		APAI-Q-IVT--A-TS---L--
	Mycobacterium hassiacum	WP_036447587	---Q---I--------S-P-N		APAS-Q------A-TS------
	Mycobacterium heckeshornense	WP_048891255	---H---------S--N---D		--AT-Q------A-TS---L--
	Mycobacterium heidelbergense	WP_083073123	---H-----S------N-T-D		G-AT-Q------A-TS---L--
	Mycobacterium heraklionense	WP_064998289	---Q--------------R-D		ARAT-A--G--AA-TS---L--
	Mycobacterium hiberniae	WP_085133936	---Q-----E--------R-D		ARAT-A--G--AA-TS---L--
	Mycobacterium holsaticum	WP_069403956	---Q------E-----N---D		A-AT-Q------A-TS---L--
	Mycobacterium icosiumassiliensis	WP_067970901	---Q-----EN-------R-D		ARAT-A--G--AA-TS---L--
	Mycobacterium insubricum	WP_083030763	---Q-----PQH-E--E-G-G		PSVT-E-IGR-SA-VS------

**Other *Mycobacterium* (0/>100)**	*Mycobacterium interjectum*	WP_085201614	---Q-----S-Q-S--N---D	G-AT-Q------A-TS---L--
	Mycobacterium intermedium	WP_069419056	---Q-----P------N-V-D	G-AT-Q---S--A-TS---L--
	Mycobacterium intracellulare	WP_064938499	---Q-----A------N-T-D	A-AT-Q---D--A-TS---L--
	Mycobacterium iranicum	WP_064283197	---Q-----EES------P-N	ATAT-Q------A-TS------
	Mycobacterium kansasii	WP_063467948	---Q-----S------N-T-E	G-AT-Q------A-TS---L--
	Mycobacterium komanii	CRL78140	---N----------------D	AAAT-Q------A-TS---L--
	Mycobacterium koreense	WP_085302486	---N-----S-----E--P-N	APAT-------QA-TS---L--
	Mycobacterium kumamotonense	WP_065287459	---Q-----E----------D	ARAT-A--G--VA-TS---L--
	Mycobacterium kyorinense	WP_065016588	---H---------S--N---D	ARAT-E---S--A-TS---L--
	Mycobacterium lacus	WP_085157001	---Q-----A------N-T-D	--AT-Q------A-TS---L--
	Mycobacterium lentiflavum	CQD08526	---Q-----S-Q----N---D	--AT-Q------A-TS---L--
	Mycobacterium leprae	WP_010907856	---H-----E------N-I-D	APAI-Q-I-T-HA-TS---L--
	Mycobacterium lepromatosis	WP_045842624	---H-----E------N-T-D	ASAI-Q-I-T--A-TS---L--
	Mycobacterium liflandii	WP_015354907	---Q-----SN-----N-T-D	GEAT-Q------A-TS---L--
	Mycobacterium litorale	WP_078020415	---Q-----N-H---------	APTT-Q------A-TS------
	Mycobacterium llatzerense	WP_071286862	---------G-S--------N	APAT-E------A-TS---L--
	Mycobacterium longobardum	WP_085264345	---Q-----E------N---D	ARAT-A--G--AA-TS---L--
	Mycobacterium mageritense	WP_036428202	------R-GS-R-E----P-I	GTKNPRD----AT----A-L--
	Mycobacterium malmesburyense	CRL77529	---N------------N---D	APAT-Q------A-TS---L--
	Mycobacterium malmoense	WP_065445036	---Q-----S------T-A-D	T-AT-Q---D--A-TS---L--
	Mycobacterium mantenii	WP_083096129	---Q-----S-Q----N-T-D	A-AT-Q---D--A-TS---L--
	Mycobacterium marinum	WP_012393237	---Q-----SN-----N-T-D	GEAT-Q------A-TS---L--
	Mycobacterium marseillense	WP_083020076	---Q-----A------N-T-D	A-AT-Q---D--A-TS---L--
	Mycobacterium microti	AMC57501	---E--RIGG-W-Q--S-P-I	GTKQPRD-PV-VT---KV-L--
	Mycobacterium minnesotense	WP_083027533	---Q-----E--------R-D	ARAT-A-IG--AA-TS---L--
	Mycobacterium moriokaense	WP_083154147	---Q------ET--------D	A-TT-Q---D--A-TS---L--
	Mycobacterium mucogenicum	WP_064860812	---------G-S--------N	APAT-E------A-TS---L--
	Mycobacterium mungi	WP_064319850	---E--RIGG-W-Q--S-P-I	GTKQPRD-PV-VT---KV-L--
	Mycobacterium nebraskense	WP_085165081	---Q-----S------T-A-D	T-AT-Q---D--A-T----L--
	Mycobacterium neoaurum	CDQ46083	----------QT-E--N-G-D	ANAT-Q--SS-TA-TS---L--
	Mycobacterium neworleansense	CRZ15539	---Q------ET----N-ESN	G-TT-A------A-TS---L--
	Mycobacterium nonchromogenicum	WP_085137944	---Q--------------R-D	ARAT-A--G--AA-TS---L--
	Mycobacterium noviomagense	WP_083086255	---Q---------S--N---D	--AT-Q------A-TS---L--
	Mycobacterium novocastrense	WP_067395782	---Q---------S----P-N	AAAT-A------A-TS------
	Mycobacterium obuense	KMO80355	---Q-----ENE------P-N	APAT-Q------A-TS------
	Mycobacterium orygis	WP_003400572	---E--RIGG-W-Q--S-P-I	GTKQPRD-PV-VT---KV-L--
	Mycobacterium palustre	WP_085077998	---Q-----A------N-T-D	G-AT-L------A-TS---L-
	Mycobacterium paraense	WP_085103820	---Q-----S-Q-S--N-T-D	G-AT-Q------A-TS---L--
	Mycobacterium paraffinicum	WP_073876024	---Q-----S------T-A-D	T-AT-Q---D--A-TS---L--
	Mycobacterium parafortuitum	WP_083143055	---Q---I--E--N----P-N	ATAT-Q------A-TS------
	Mycobacterium parascrofulaceum	WP_007168175	---Q-----S------T-A-D	T-AT-Q---D--A-TS---L--
	Mycobacterium paraseoulense	WP_083170110	---Q-----S------T-A-D	T-AT-Q---D--A-TS---L--
	Mycobacterium parmense	WP_085267731	---Q-----S------P-T-D	A-AT-Q---S--A-TS---L--
	Mycobacterium peregrinum	WP_064878842	---Q-------T----N-ESN	G-TT-A------A-TS---L--
	Mycobacterium persicum	WP_083153879	---Q-----S------N-T-E	G-AT-Q------A-TS---L--
	Mycobacterium phlei	AMO60535	---H----------------D	ATAT-Q------A-TS---L--
	Mycobacterium porcinum	WP_075921444	---Q-------T----N-ESN	G-TT-A------A-TS---L--
	Mycobacterium pseudoshottsii L15	GAQ36875	---Q-----SN-----N-T-D	GEAT-Q------A-TS---L--
	Mycobacterium rhodesiae	WP_083121063	---Q-----N-H---------	APTT-Q---S--A-TS------
	Mycobacterium riyadhense	WP_085249736	---Q-----SE-----N-T-D	G-AT-Q------A-TS---L--
	Mycobacterium rufum	KGI67404	---Q-----EEQ-S----P-N	APAS-Q------A-TS------
	Mycobacterium rutilum	SEH90102	---Q------E---------D	A-AT-Q------A-TS---L--
	Mycobacterium saskatchewanense	WP_085257921	---Q------------N-T-D	-PAN-Q---T--A-TS---L--
	Mycobacterium scrofulaceum	WP_067277590	---Q-----S------T-A-D	T-AT-Q---D--A-TS---L--
	Mycobacterium senuense	WP_085081726	---H-----E----------D	ARAT-A--G--VA-TS---L--
	Mycobacterium septicum	WP_044516826	---Q-----N-T----N-ESN	G-TT-A------A-TS---L--
	Mycobacterium setense	WP_064871743	---Q-------T----N-ESN	G-TT-A------A-TS---L--
	Mycobacterium sherrisii	WP_069402474	---Q-----S------N---D	--AT-Q------A-TS---L--
	Mycobacterium shigaense	BAX91445	---Q-----A-Q----N---D	-PAN-Q------A-TS---L--
	Mycobacterium shimoidei	WP_069396543	---Q------------N---D	-RAT-E------A-TS---L--
	Mycobacterium shinjukuense	WP_083049847	---Q-----SE-----N-T-D	A-AT-Q------A-TS---L--
	Mycobacterium simiae	WP_061558588	---Q-----S------N---D	--AT-Q------A-TS---L--
	Mycobacterium sinense	WP_041318415	---H-----E------N---D	ARAT-A--G--AA-TS---L--
	Mycobacterium smegmatis	WP_011728043	---H------ET----N-EN-	G-TT-A------A-TS---L--
	Mycobacterium szulgai	WP_085674308	---Q-----S------N-T-D	G-AT-Q------A-TS---L--

Other *Mycobacterium* (0/>100)

Mycobacterium terrae	WP_085261981	---Q-----E----------D	ARAT-A--G--VA-TS---L--
Mycobacterium thermoresistibile	WP_003927921	---H-----E------S-P-N	APAT-A-----AA-TS------
Mycobacterium triplex	WP_036467404	---Q-----S-Q----N---D	--AT-Q------A-TS---L--
Mycobacterium triviale	WP_069393366	---N-----S-----E--P-N	APAT-------QA-TS---L--
Mycobacterium tuberculosis	WP_064314163	---Q-----S------N---D	-PAT-Q------A-TS---L--
Mycobacterium tusciae	WP_040538682	---Q------ET--------D	A-TT-Q---S--A-TS---L--
Mycobacterium ulcerans	WP_011740463	---Q-----SN-----N-T-D	GEAT-Q------A-TS---L--
Mycobacterium vaccae	WP_003929184	---Q------EA-E----P-N	TAAT-Q------A-TS------
Mycobacterium vanbaalenii PYR-1	A1T636	---Q------EA-E----P-N	ATAT-Q------A-TS------
Mycobacterium vulneris	WP_085292559	---Q-----S-Q----N-T-D	A-AT-Q---D--A-TS---L--
Mycobacterium wolinskyi	WP_085144909	---Q------ET----N-ESS	G-TT-A------A-TS---L--
Mycobacterium xenopi	WP_085196643	---H---------S--N---D	--AT-Q------A-TS---L--
Mycobacterium yongonense	WP_065498511	---Q-----A------N-T-D	A-AT-Q---D--A-TS---L--

Supplementary Figure 29

A partial sequence alignment of a conserved region of a membrane protein showing a six amino acid insertion that is specific for members of the "*Abscessus-Chelonae"* clade and absent in other *Mycobacterium*.

			124		163
**"Abscessus-Chelonae" Clade (5/5)**	*Mycobacterium abscessus*	WP_005074554	GVSRPGYELSSDHIAHA		ELPPDGLSLVEVPALAISSTDCR
	Mycobacterium abscessus subsp. bolletii	BAP96419	-----------------		-----------------------
	Mycobacterium chelonae	WP_046253193	-----------------		-----------------------
	Mycobacterium franklinii	WP_070935519	-----------------		-------T---------------
	Mycobacterium immunogenum	WP_043075936	--N--------------		---S-------------------
**Other *Mycobacterium* (0/>100)**	*Mycobacterium alsense*	WP_083136723	---------RRE--TGV	LG	--TE-A-T---I-----------
	Mycobacterium angelicum	WP_083114305	---------RR---TG-	LG	--AESA-T---I-----------
	Mycobacterium aromaticivorans	WP_036344411	---------NGQ--VA-	FD	Q--EGA----S------------
	Mycobacterium arosiense	WP_083062918	---------RRE-VTGV	LG	---E-A-T---I-----------
	Mycobacterium arupense	KKC01369	---------NG--L-D-	LE	S---HA-T---------------
	Mycobacterium asiaticum	OBI87068	---------RN---IE-	IG	D-DEGT-T-Y-I-----------
	Mycobacterium aurum	WP_087024911	---------DGE---A-	ME	---A-A-T-----------S---
	Mycobacterium austroafricanum	WP_084182307	---------DGK--SA-	MA	---D-A-H-I-------------
	Mycobacterium avium	WP_010949532	---------CRE--TGV	LG	---D-A-T---I-----------
	Mycobacterium avium subsp. avium	A0QDI7	---------RRE--TGV	LG	---D-A-T---I-----------
	Mycobacterium avium subsp. hominissuis	KDP00715	---------RRE--TGV	LG	---D-A-T---I-----------
	Mycobacterium avium subsp. paratuberculosis	EGO37201	---------RRE--TGV	LG	---D-A-T---I-----------
	Mycobacterium bacteremicum	WP_083055954	---------DGE---A-	MK	---AEA-----I-------S---
	Mycobacterium boenickei	WP_077741354	---------DGE--EA-	LR	-----T-T-----------S---
	Mycobacterium bohemicum	ORU95997	---------RRE--TGV	LG	---DEV-T---I-----------
	Mycobacterium branderi	WP_083131556	---------GRE-L-DV	LK	G--D-A-T-L-I-----------
	Mycobacterium brisbanense	GAS86064	---------DGK--SA-	RQ	-----A-Q-----------S---
	Mycobacterium canariasense	WP_062657457	---------DGE---S-	MK	---D-A-------------S---
	Mycobacterium canettii	WP_014001267	---------RNE--TSL	LG	Q-AK-A-T---I-----------
	Mycobacterium celatum	WP_084707034	---------GRE-L-DV	LE	G--D-A-T-L-I-----------
	Mycobacterium celeriflavum	WP_083001283	---------DGK---A-	IK	---D-A-Q---------------
	Mycobacterium chlorophenolicum	WP_082168607	---------DGK--SA-	MA	---A-A-H---------------
	Mycobacterium chubuense	WP_081495085	---------DGK--SA-	MA	---A-A-H---------------
	Mycobacterium colombiense	OBB86965	---------RRE--TGV	LG	---E-A-T---I-----------
	Mycobacterium confluentis	WP_085153205	------F--DGK--SA-	MA	---A-A-----I-----------
	Mycobacterium conspicuum	ORV37556	---------RDE--TSV	LG	D-AE-A-T---I-----------
	Mycobacterium diernhoferi	OJZ68328	---------DGE---A-	MK	---E-A-T-----------S---
	Mycobacterium doricum	WP_085189825	---------DGK--SA-	MR	---D-A-------------S---
	Mycobacterium elephantis	KKW65295	---------DGK-VTA-	GK	Q--R-A-T---------------
	Mycobacterium engbaekii	ORV42805	---------NG--L-E-	LG	S--EHA-T---------------
	Mycobacterium europaeum	WP_085240256	---------RRE--TGV	LG	--DD-V-T---I-----------
	Mycobacterium fallax	ORU96656	------F---GK--IE-	MA	---A-A-T---------------
	Mycobacterium farcinogenes	CDP89037	---------DGE--EA-	LR	--S--A-T-----------S---
	Mycobacterium flavescens	ODQ91223	---------DGK-V-A-	IK	---E-A-Q---------------
	Mycobacterium florentinum	WP_085221214	---------RHE--TEV	IG	--AK-A-T---------------
	Mycobacterium fortuitum	OBB25947	---------DGE--EA-	LR	-----T-T-----------S---
	Mycobacterium fragae	WP_085199567	---------GDE-LGDV	LE	K--H-A-T---I-----------
	Mycobacterium gastri	WP_084293308	-------D-RH--VTA-	LA	G-SE-A-T---I-----------
	Mycobacterium genavense	WP_084298691	---------RHE--TEV	LC	--AK-A-T---I-----------
	Mycobacterium gilvum	WP_081437653	---------DGK--SA-	MA	---A-A-H---------------
	Mycobacterium goodii	WP_083452902	---------DGK--LE-	MR	-----A-------------S---
	Mycobacterium gordonae	OBJ89077	---------RH---TEV	LG	---QEA-T---I-----------
	Mycobacterium haemophilum	KLO30255	---------GHE--TGV	LG	--AA-A-T---I-----------
	Mycobacterium hassiacum	WP_081586661	---------DGA-L-D-	LR	---D-A-T---------------
	Mycobacterium heckeshornense	WP_071700272	-------D-GRE-L-EV	LD	G--D-A-T---------------
	Mycobacterium heidelbergense	WP_083072044	---------RRE--TGV	LG	--AD-A-T---------------
	Mycobacterium heraklionense	OBG37102	---------NG--L-D-	LG	S---HA-T---------------
	Mycobacterium hiberniae	ORV71299	---------NG--L-E-	LG	S--EHA-T---------------
	Mycobacterium holsaticum	ODQ85059	---------DGE-V-A-	GR	R--D-A-T---------------
	Mycobacterium houstonense	WP_074133031	---------DGE--EG-	LR	----EA-T-----------S---
	Mycobacterium icosiumassiliensis	WP_078058736	---------NG--L-E-	LG	S---HA-T---------------
	Mycobacterium insubricum	WP_083028807	------F--HGK---E-	IT	---A-A-T---I-----------
	Mycobacterium interjectum	WP_084454254	---------RRE--TGV	LG	--TE-A-T---I-----------
	Mycobacterium intermedium	WP_079220301	--N-------H---TTV	LT	N-AE-AVT-L-I-----------
	Mycobacterium intracellulare	OBH73865	----------H---TGV	LG	--AD-A-T---I-----------
	Mycobacterium iranicum	OAN36166	---------DGK--SA-	MA	---A-A-H---------------
	Mycobacterium kansasii	KZS57012	-------D-RH--VTA-	LA	G-SA-A-T---I-----------
	Mycobacterium komanii	CRL67161	---------DGK---A-	IK	---D-A-K---------------
	Mycobacterium kubicae	ORW02378	---------RRE-VTGV	LV	D--ENA-T---I-----------
	Mycobacterium kumamotonense	OBY29718	------F--RH---TG-	LG	--EEHA-T---I-----------
	Mycobacterium kyorinense	OBI43586	---------GRE-L-DV	LD	G--D-A-T---I-----------
	Mycobacterium lacus	WP_085162926	---------RH---TD-	LG	---K-A-T-I-I--------E--
	Mycobacterium lentiflavum	CQD17367	---------RHE--TEV	LG	--AK-A-T---------------
	Mycobacterium leprae	WP_081439343	---------GQE--TGV	MG	---A-A-T---I-----------
	Mycobacterium lepromatosis	KJX75122	---------GHE--TGV	LG	---AEA-T-I-I-----------

Other *Mycobacterium* (0/>100)

Mycobacterium liflandii	WP_083866478	---------RH--VTA- LD G-AE-A-----I-----------
Mycobacterium litorale	WP_078018706	---------NGQ--VA- FD Q--EEA-----------------
Mycobacterium llatzerense	WP_082067889	---------DGK--ED- QK -----A------------S---
Mycobacterium longobardum	WP_085263537	---------NG--L-D- LG S--AHA-T---------------
Mycobacterium mageritense DSM 44476	CDO26897	---------DGE--SG- IR ---A-T-N-----------S---
Mycobacterium malmesburyense	CRL79062	---------DGK---A- IK ---D-A-K---------------
Mycobacterium malmoense	OCB30353	---------RRE--TGV LG --AE-V-T---I-----------
Mycobacterium mantenii	WP_083097000	---------RRE-VTGV LG ---E-A-T---I-----------
Mycobacterium marinum M	B2HME5	---------RH--VTA- LD G-AE-A-----I-----------
Mycobacterium minnesotense	WP_083022287	---------NG--L-D- LE S---HA-T---------------
Mycobacterium moriokaense	WP_083150130	---------DGQ--KT- LT ---N-A-T---------------
Mycobacterium mucogenicum	OBA76875	---------DGK--EE- QK ---A-A-T-----------S---
Mycobacterium mungi	WP_064319596	---------RNE--TSL LG Q-AK-A-T---I-----------
Mycobacterium nebraskense	WP_082122841	---------RRE--TGV LG --AE-V-T---I-----------
Mycobacterium neoaurum	CDQ46776	---------DGE---A- MK ---D-A-----I-------S---
Mycobacterium neworleansense	CRZ13536	---------DGE--EA- LR -----A-T-----------S---
Mycobacterium noviomagense	WP_083084393	-------DFRRE-LGDI LD G--D-A-T---------------
Mycobacterium novocastrense	WP_084377017	---------DGK---A- IK ---D-A-R---------------
Mycobacterium obuense	KKE98823	---------DGK--SA- MA ---D-A-H---------------
Mycobacterium palustre	ORW25503	---------RH---TGV LG --AEGA-T---I-----------
Mycobacterium paraense	ORW27421	---------RRE--TGV LG --TE-A-T---I-----------
Mycobacterium paraffinicum	OJZ66310	---------RRE--TGV LG --AE-V-T---I-----------
Mycobacterium parafortuitum	WP_083145149	---------DGK--TT- MA ---T-A-H---------------
Mycobacterium paraintracellulare	AFC53080	----------H---TGV LG --AD-A-T---I-----------
Mycobacterium paraseoulense	WP_083168782	---------RRE-VTGV LG --AD-V-T---I-----------
Mycobacterium parmense	ORW62919	---------RRE--TGV LG ---EEV-T---I-----------
Mycobacterium peregrinum	OBB32608	---------DGE--EA- LR -----A-T-----------S---
Mycobacterium persicum	WP_083153222	-------D-RH--VTA- VA G-SE-A-T---I-----------
Mycobacterium phlei	WP_003890469	---------DAS---A- TQ Q--A-A-T---------------
Mycobacterium porcinum	WP_075922021	---------DGE--EA- LR -----T-T-----------S---
Mycobacterium rhodesiae	WP_083121342	---------NGQ--VA- FD Q--EEA----S------------
Mycobacterium riyadhense	WP_085250733	---------HH---TGV LG --GENA-T---I-----------
Mycobacterium rufum	KGI68989	---------DGK--SA- MA ---A-A-H---------------
Mycobacterium rutilum	SEH73498	---------DGK---A- IK ---D-A-Q---------------
Mycobacterium saskatchewanense	ORW65908	---------RRE--TGV LG ---D-V-T---I-----------
Mycobacterium scrofulaceum	OBH74621	---------RRE-VTGV LG --AE-V-T---I-----------
Mycobacterium senuense	ORW67541	---------RH---TGV LG --EEHA-T---I-----------
Mycobacterium septicum	WP_084622227	---------DG---EA- LR -----A-T-----------S---
Mycobacterium setense	WP_082017617	---------DGE--EA- LR -----A-T-----------S---
Mycobacterium sherrisii	ODR09723	-------D-RRE--TD- LG --AQ-A-A---I-----------
Mycobacterium shigaense	BAX93557	---------HHE--TE- VG --AK-A-T---I-----------
Mycobacterium shimoidei	ODR15086	---------DRE-L-GV LE G--E-A-T-I-I-----------
Mycobacterium shinjukuense	WP_083046292	---------RR---TGV LG --AENA-T---I-----------
Mycobacterium simiae	AMP22330	-------D-RRE--TD- LG --AQ-A-T---I-----------
Mycobacterium sinense	OBG01529	------F--RH---TGV LG --EENA-T------------E--
Mycobacterium smegmatis	WP_011729940	---------DGK--LD- MR -----A------------S---
Mycobacterium szulgai	OBF16093	---------RRE-VTGV LV D--ENA-T---I-----------
Mycobacterium terrae	WP_085260284	------F--RH---TSV LG K-EENA-T---I-----------
Mycobacterium thermoresistibile	WP_081475549	---------DGE--LG- LK -K-A-A-T-----------S---
Mycobacterium triplex	WP_084163466	---------RHE--TEV LG --AK-A-T---I-----------
Mycobacterium triviale	WP_085108874	-------D-ND--LGDV LD D--G-A-T-I-------------
Mycobacterium tuberculosis	SGO91974	---------RNE--TSL LG Q-AK-A-T---I-----------
Mycobacterium tusciae	WP_006241165	---------DGQ--TA- MA ----EA-----------------
Mycobacterium ulcerans Agy99	ABL05820	---------RH--VTA- LD G-AENA-----I-----------
Mycobacterium vaccae	WP_081528947	---------DGK--SA- MA ---D-A-N-I-------------
Mycobacterium vanbaalenii	WP_011781083	---------DGK--SA- MA ---D-A-H-I-------------
Mycobacterium vulneris	WP_085291539	---------RRE--TGV LG ---E-A-T---I-----------
Mycobacterium wolinskyi	WP_084356534	---------DGK--SA- LQ -----A-K-----------S---
Mycobacterium xenopi	WP_081485340	-------D-GRE-L-QV LD G--D-V-T---------------

Supplementary Figure 30

A partial sequence alignment of a conserved region of nicotinate-nucleotide adenylyltransferase showing a two amino acid deletion that is specific for members of the "*Abscessus-Chelonae"* clade and absent in other *Mycobacterium*.

Group	Species	Accession	172		207
**"Abscessus-Chelonae" Clade (5/5)**	*Mycobacterium abscessus*	WP_052529870	ASRAAHIPGPIDGPTR		DPAELPEAVSLTRRMGMTGK
	Mycobacterium abscessus subsp. bolletii	EUA67690	----------------		--------------------
	Mycobacterium chelonae	WP_070915993	------L---------		E----A-G-A----------
	Mycobacterium franklinii	WP_070937818	----------------		-----A-G-A----------
	Mycobacterium immunogenum	OAT69022	------L---------		---D--QG-A-A--------
**Other *Mycobacterium* (0/76)**	*Mycobacterium alsense*	WP_083136911	-AK--NL-SA-----I	GS	NALK-I--TAVSVEF-----
	Mycobacterium arosiense	WP_083065578	-AK--SL-SA-----I	GS	NALK-I--TAVSVEF-----
	Mycobacterium aurum	WP_048631542	-----GL--------L	RD	QARH-TRETEVAKAA----R
	Mycobacterium austroafricanum	WP_011779604	-AK---L-SA-----V	GS	SALR-S--TAVSAEF-----
	Mycobacterium avium	WP_003874873	--K--DL-SA-----I	GS	N-LK-I--TAVSTQF-----
	Mycobacterium bacteremicum	WP_083060509	-----QL-A------L	RD	QSRH-ARETEVAKAA----R
	Mycobacterium boenickei	WP_077739036	-AK--GL--A-----V	GS	SALR-S--TAVSAEF-----
	Mycobacterium bohemicum	WP_085181891	-AK--NL-SA-----I	GS	NALK-I--TAVSVEF-----
	Mycobacterium brisbanense	WP_062831119	-AK---L-SA-----V	GS	SALR-S--TAVSAEF-----
	Mycobacterium canariasense	WP_062656495	-AK--QL-SA-----V	GS	SARK-S--TAVSAEF-----
	Mycobacterium celeriflavum	WP_082999911	-AK---L-SA-----V	GS	SALK-S--TAVSAEF-----
	Mycobacterium chlorophenolicum	WP_048470272	-AK---L-SA-----V	GS	SALR-S--TAVSAEF-----
	Mycobacterium chubuense	WP_014815342	-AK---L-SA-----V	GS	SALR-S--TAVSAEF-----
	Mycobacterium colombiense	WP_064877307	--K--NL-SA-----I	GS	N-LK-I--TAVSTQF-----
	Mycobacterium conceptionense	WP_064894448	-AK--GL--A-----V	GS	SALR-S--TAVSAEF-----
	Mycobacterium confluentis	WP_085152710	--T---L-S------V	GS	SALK-S--TAVSTEF-----
	Mycobacterium conspicuum	WP_085232443	-AK--NL-SA-----I	GS	NALK-I--TAVSVEF-----
	Mycobacterium cosmeticum	CDO09063	-----QL-A------L	RT	AANR-ARETAVAKAA--S-R
	Mycobacterium doricum	WP_085192299	-AK---L-SA-----I	GS	S-LR-I--TAVSAEF-----
	Mycobacterium europaeum	WP_085243488	-AK--SL-SA-----I	GS	NALK-I--TAVSVEF-----
	Mycobacterium fallax	WP_085094402	-A----L-SA-----V	SS	SALK-S--AAVSAEF-----
	Mycobacterium farcinogenes	WP_036388042	-AK--GL--A-----V	GS	SALR-S--TAVSAEF-----
	Mycobacterium flavescens	WP_069416135	-AK---L-SA-----V	GS	SARK-S--TAVSAEF-----
	Mycobacterium fortuitum	WP_064914788	-AK--GL--A-----V	GS	SALR-S--TAVSAEF-----
	Mycobacterium fragae	WP_085193366	-AK--NL-SA-----I	GS	N-LK-I--TAVSAEF-----
	Mycobacterium gastri	WP_036416319	-AK--NL-SA-----I	GT	N-LK-I--TAVSVEF-----
	Mycobacterium gilvum	WP_011894859	-AK---L-SA-----V	GS	SALK-S--TAVSAEF-----
	Mycobacterium goodii	WP_049747257	-AK---L-SA-----V	GS	S-LK-A--TAVSAEF-----
	Mycobacterium haemophilum	WP_047313447	-AK--NL-SA-----I	GS	NALK-I--TAVSAEF-----
	Mycobacterium hassiacum	WP_005626765	-AK---L-SA-----V	GT	NALK-S--TAVSAEF-----
	Mycobacterium heckeshornense	WP_048890876	-AK--NL-SA-----I	GS	N-LK-I--TAVSAEF-----
	Mycobacterium heidelbergense	WP_083077317	-AK--NL-SA-----I	GS	NALK-I--TAVSVEF-----
	Mycobacterium holsaticum	WP_069405233	-AK---L-SA-----V	GH	SALV-S--TAVSAEF-----
	Mycobacterium houstonense	WP_066896646	-AK--GL--A-----V	GS	SALR-S--TAVSAEF-----
	Mycobacterium insubricum	WP_083029594	-A----L-SA-----V	GS	SALK-S--AAVSAEF-----
	Mycobacterium interjectum	WP_066909011	-AK--NL-SA-----I	GS	NALK-I--TAVSVEF-----
	Mycobacterium intracellulare	WP_064936856	--K--DL-SA-----I	GS	N-LK-I--TAVSTQF-----
	Mycobacterium kansasii	WP_063466372	-AK--NL-SA-----I	GT	N-LK-I--TAVSVEF-----
	Mycobacterium komanii	CRL67616	-AK---L-SA-----V	GS	SALK-S--TAVSVEF-----
	Mycobacterium litorale	WP_078019219	-AK---L--AV----V	GS	SALK-S--TAVSAEF-----
	Mycobacterium malmesburyense	CRL75475	-AK---L-SA-----V	GS	SALK-S--TAVSVEF-----
	Mycobacterium malmoense	WP_065444121	-AK--SL-SA-----I	GS	NALK-I--TAVSVEF-----
	Mycobacterium mantenii	WP_083092885	--K--DL-SA-----I	GS	N-LK-I--TAVSTQF-----
	Mycobacterium marseillense	WP_083019612	--K--DL-SA-----I	GS	N-LK-I--TAVSTQF-----
	Mycobacterium moriokaense	WP_083149803	-AK---L-SA-----V	GS	SALK-S--TAVSAEF-----
	Mycobacterium mucogenicum	WP_064859426	-AK---L--A-----I	GS	SSRK-S--TAVSTEF-----
	Mycobacterium nebraskense	WP_046186948	-AK--SL-SA-----I	GS	N-LK-I--TAVSVEF-----
	Mycobacterium neworleansense	CRZ14950	-AK--GL--A-----V	GS	SALR-S--TAVSAEF-----
	Mycobacterium noviomagense	WP_083090000	-AK--NL-SA-----I	GS	N-LK-I--TAVSAEF-----
	Mycobacterium obuense	WP_082163888	-----GL-A------L	RI	NA----RDTEVAKAA----R
	Mycobacterium palustre	WP_085081424	-AK--NL-SA-----I	GS	NALK-I--TAVSIEF-----
	Mycobacterium paraense	WP_085103461	-AK--NL-SA-----I	GS	NALK-I--TAVSVEF-----
	Mycobacterium paraffinicum	WP_073872090	-AK--SL-SA-----I	GS	NALK-I--TAVSVEF-----
	Mycobacterium paraintracellulare	AFC55444	--K--DL-SA-----I	GS	N-LK-I--TAVSTQF-----
	Mycobacterium paraseoulense	WP_083172532	-AK--SL-SA-----I	GS	NALK-I--TAVSVEF-----
	Mycobacterium parmense	WP_085267861	--K--SL-SA-----I	GS	N-LK-I--SAVSVEF-----
	Mycobacterium peregrinum	WP_064884334	-AK--GL--A-----V	GS	SALR-S--TAVSAEF-----
	Mycobacterium persicum	WP_089025301	-AK--NL-SA-----I	GT	N-LK-I--TAVSVEF-----
	Mycobacterium phlei	WP_003887521	-AK---L--A-----V	GT	SALK-S--TAVSAEF-----

Group	Species	Accession	Sequence
**Other *Mycobacterium* (0/76)**	*Mycobacterium porcinum*	WP_075923468	-AK--GL--A-----V GS SALR-S--TAVSAEF-----
	Mycobacterium rhodesiae	WP_041304115	-AK---L-SA-----V GS SALK-S--TAVSAEF-----
	Mycobacterium rufum	KGI67841	-AK---L-SA-----V GS SALR-S--TAVSAEF-----
	Mycobacterium rutilum	WP_083409305	-AK---L-SA-----V GS SARK-S--TAVSAEF-----
	Mycobacterium scrofulaceum	WP_067271233	-AK--SL-SA-----I GS NALK-I--TAVSVEF-----
	Mycobacterium septicum	WP_044517737	-AK--GL--A-----V GS SALR-S--TAVSAEF-----
	Mycobacterium setense	WP_039319206	-AK--GL--A-----V GS SALR-S--TAVSAEF-----
	Mycobacterium shinjukuense	WP_083046131	-----NL-SA-----I GS NALK-I--TAVSLEF-----
	Mycobacterium smegmatis	WP_003894050	-AK---L-SA-----V GS S-LK-A--TAVSAEF-----
	Mycobacterium tuberculosis	CNI16729	--TLGGL--------L GA YG-D-SK-CGYANDH-----
	Mycobacterium tusciae	WP_083127636	-AK---L-SA-----V GS SALK-S--TAVSAEF-----
	Mycobacterium vaccae	WP_040543122	-AK---L-SA-----V GS SALR-S--TAVSAEF-----
	Mycobacterium vanbaalenii	WP_086008496	-AK---L-SA-----V GS SALR-S--TAVSAEF-----
	Mycobacterium vulneris	WP_085290165	--K--DL-SA-----I GS N-LK-I--TAVSTQF-----
	Mycobacterium wolinskyi	WP_067851046	-AK---L-SA-----V GS SALK-S--TAVSAEF-----
	Mycobacterium xenopi	WP_003922226	-AK--NL-SA-----I GS N-LK-I--TAVSAEF-----
	Mycobacterium yongonense	WP_065503591	--K--DL-SA-----I GS N-LK-I--TAVSTQF-----

Supplementary Figure 31

A partial sequence alignment of a conserved region of CoA ester lyase showing a two amino acid deletion that is specific for members of the "*Abscessus-Chelonae"* clade and absent in other *Mycobacterium*.

Group	Species	Accession	342		382
**"Abscessus-*Chelonae*" Clade (6/6)**	*Mycobacterium abscessus*	WP_052613689	VVTYRDYSGTASSPAGS	QQLAKQ	VAADRSTIANIRVLDPNI
	Mycobacterium abscessus subsp. bolletii	EHM16461	-----------------	------	------------------
	Mycobacterium chelonae	WP_070919384	------------A-T--	E-----	------------------
	Mycobacterium franklinii	WP_070938762	------------A-T--	E-----	------------------
	Mycobacterium immunogenum	WP_064628010	I-----------A----	------	------------------
	Mycobacterium saopaulense	WP_070909629	------------A-T--	E-----	------------------
**Other *Mycobacterium* (0/95)**	*Mycobacterium acapulcensis*	WP_083997650	--S----P-N-PAT-AQ		-----A-TS---------
	Mycobacterium africanum	WP_031701648	-----N---DSPAT-QQ		-----A-TS---L---T-
	Mycobacterium alsense	WP_083140568	--S--N-T-DGQAT-QQ		-----A-TS---L---T-
	Mycobacterium angelicum	WP_083111069	-----N-T-DGQAT-QQ		-----A-TS---L---T-
	Mycobacterium aromaticivorans	WP_036340069	----------PTT-QQ		--S--A-TS-------T-
	Mycobacterium asiaticum	WP_065035308	-----N-T-DGQAT-QQ		-----A-TS---L---T-
	Mycobacterium aurum	WP_087020426	---------D-AAT-QQ		-----A-TS---L---T-
	Mycobacterium boenickei	WP_077743615	----N-ESNGQTT-AQ		-----A-TS---L---T-
	Mycobacterium bohemicum	WP_085181997	-----N-T-D-PAN-QQ		--T--A-TS---L---T-
	Mycobacterium bovis	WP_019283731	-----N---DSPAT-EQ		-----A-TS---L---T-
	Mycobacterium branderi	WP_083129858	--S--N---DGRAT-EQ		--S--A-TS---L---T-
	Mycobacterium brisbanense	WP_062831658	----N-ESNGQTT-AQ		-----A-TS---L---T-
	Mycobacterium canariasense	GAS93131	--------NSQAT-QQ		-----A-TS---L---T-
	Mycobacterium canettii	WP_015291267	-----N---DSPAT-QQ		-----A-TS---L---T-
	Mycobacterium celatum	WP_085167690	--S--N---DGRAT-EQ		--S--A-TS---L---T-
	Mycobacterium celeriflavum	WP_083006383	-----N---DSPAT-QQ		-----A-TS---L---T-
	Mycobacterium chlorophenolicum	KMO75863	-S----A-N-PAS-QQ		-----A-TS---------
	Mycobacterium chubuense	WP_041781791	-S----A-N-PAS-QQ		-----A-TS---------
	Mycobacterium conceptionense	WP_085140998	----N-ESSGQTT-AQ		-----A-TS---L---T-
	Mycobacterium confluentis	WP_085148630	-----N---D-PTS-QQ		I-S--A--S---L---T-
	Mycobacterium conspicuum	WP_085231379	--S--N-N-D-PAT-QQ		--T--A-TS---L---T-
	Mycobacterium cosmeticum	CDO10255	--------NSQAT-QQ		-----A-TS---L---T-
	Mycobacterium diernhoferi	WP_079244263	--S------D-AAT-QQ		-----A-TS---L---T-
	Mycobacterium doricum	WP_085190019	-------P-D-PAT-QQ		-----A-TS---------
	Mycobacterium elephantis	WP_083042562	-----S---D-QAT-QQ		-----A-TS---L---T-
	Mycobacterium fallax	WP_085092481	--------DTPTT-KE		I----A-VS-------A-
	Mycobacterium farcinogenes	CDP84269	----N-ESSGQTT-AQ		-----A-TS---L---T-
	Mycobacterium flavescens	WP_069416781	--S----P-N-EAT-AQ		-----A-TS---------
	Mycobacterium florentinum	WP_085223062	----N---DGQAT-QQ		-----A-TS---L---T-
	Mycobacterium fortuitum	WP_061262691	----N-ESNGQTT-AQ		-----A-TS---L---T-
	Mycobacterium fragae	WP_085195828	--S--N---DSQAT-AQ		-----A-TS---L---T-
	Mycobacterium gastri	WP_085104988	--A--N-T-EGQAT-QQ		-----A-TS---L---T-
	Mycobacterium genavense	WP_025735717	----N---DSQAT-QQ		-----A-TS---L---T-
	Mycobacterium gilvum	WP_011895490	------P-N--AT-QQ		-----A-TS---------
	Mycobacterium goodii	WP_049747760	----N-EN-GQTT-AQ		-----A-TS---L---T-
	Mycobacterium gordonae	WP_065047223	-----N-T-DGQAT-QQ		-----A-TS---L---T-
	Mycobacterium hassiacum	WP_036447587	-----S-P-N-PAS-QQ		-----A-TS-------T-
	Mycobacterium heckeshornense	WP_048891255	--S--N---DSQAT-QQ		-----A-TS---L---T-
	Mycobacterium heidelbergense	WP_083073123	-----N-T-DGQAT-QQ		-----A-TS---L---T-
	Mycobacterium holsaticum	WP_069403956	-----N---D-QAT-QQ		-----A-TS---L---T-
	Mycobacterium insubricum	WP_083030763	-E--E-G-GP-VT-EQ		IGR-SA-VS-------T-
	Mycobacterium interjectum	WP_085201614	-S--N---DGQAT-QQ		-----A-TS---L---T-
	Mycobacterium intermedium	WP_069419056	-----N-V-DGQAT-QQ		--S--A-TS---L---T-
	Mycobacterium iranicum	WP_064283197	------P-N-TAT-QQ		-----A-TS---------
	Mycobacterium kansasii	WP_063467948	-----N-T-EGQAT-QQ		-----A-TS---L---T-
	Mycobacterium komanii	CRL78140	---------D-AAT-QQ		-----A-TS---L---T-
	Mycobacterium koreense	WP_085302486	----E--P-N-PAT-KQ		----QA-TS---L---T-
	Mycobacterium kyorinense	WP_065016588	--S--N---D-RAT-EQ		--S--A-TS---L---T-
	Mycobacterium lacus	WP_085157001	-----N-T-DSQAT-QQ		-----A-TS---L---T-
	Mycobacterium lentiflavum	CQD08526	----N---DSQAT-QQ		-----A-TS---L---T-
	Mycobacterium lepromatosis	WP_045842624	-----N-T-D--AI-QQ		I-T--A-TS---L---T-
	Mycobacterium liflandii	WP_015354907	-----N-T-DGEAT-QQ		-----A-TS---L---T-
	Mycobacterium litorale	WP_078020415	----------PTT-QQ		-----A-TS-------T-
	Mycobacterium llatzerense	WP_071286862	--------N-PAT-EQ		-----A-TS---L---T-
	Mycobacterium mageritense	WP_036428202	-E----P-IGTKNPRD		----AT----A-L---KV
	Mycobacterium malmesburyense	CRL77529	-----N---D-PAT-QQ		-----A-TS---L---T-
	Mycobacterium malmoense	WP_071512482	-----N-T-DGQAT-QQ		-----A-TS---L---T-
	Mycobacterium marinum	WP_012393237	-----N-T-DGEAT-QQ		-----A-TS---L---T-

Group	Species	Accession	Sequence	Sequence
**Other *Mycobacterium* (0/95)**	*Mycobacterium mucogenicum*	WP_064860812	--------N-PAT-EQ	-----A-TS---L---T-
	Mycobacterium neworleansense	CRZ15539	----N-ESNGQTT-AQ	-----A-TS---L---T-
	Mycobacterium noviomagense	WP_083086255	--S--N---DSQAT-QQ	-----A-TS---L---T-
	Mycobacterium novocastrense	WP_067395782	--S----P-N-AAT-AQ	-----A-TS---------
	Mycobacterium obuense	WP_046365193	------P-N-PAT-QQ	-----A-TS---------
	Mycobacterium paraense	WP_085174259	-S--N-T-DGQAT-QQ	-----A-TS---L---T-
	Mycobacterium parafortuitum	WP_083143055	--N----P-N-TAT-QQ	-----A-TS---------
	Mycobacterium parmense	WP_085267731	-----P-T-D-QAT-QQ	--S--A-TS---L---T-
	Mycobacterium peregrinum	WP_064878842	----N-ESNGQTT-AQ	-----A-TS---L---T-
	Mycobacterium persicum	WP_083153879	-----N-T-EGQAT-QQ	-----A-TS---L---T-
	Mycobacterium phlei	AMO60535	---------D-TAT-QQ	-----A-TS---L---T-
	Mycobacterium porcinum	WP_075921444	----N-ESNGQTT-AQ	-----A-TS---L---T-
	Mycobacterium pseudoshottsii L15	GAQ36875	-----N-T-DGEAT-QQ	-----A-TS---L---T-
	Mycobacterium rhodesiae	WP_083121063	----------PTT-QQ	--S--A-TS-------T-
	Mycobacterium riyadhense	WP_085249736	-----N-T-DGQAT-QQ	-----A-TS---L---T-
	Mycobacterium rufum	KGI67404	-S----P-N-PAS-QQ	-----A-TS---------
	Mycobacterium rutilum	SEH90102	---------D-QAT-QQ	-----A-TS---L---T-
	Mycobacterium saskatchewanense	WP_085257921	-----N-T-DSPAN-QQ	--T--A-TS---L---T-
	Mycobacterium septicum	WP_044516826	----N-ESNGQTT-AQ	-----A-TS---L---T-
	Mycobacterium setense	WP_064871743	----N-ESNGQTT-AQ	-----A-TS---L---T-
	Mycobacterium sherrisii	WP_069402474	-----N---DSQAT-QQ	-----A-TS---L---T-
	Mycobacterium shigaense	BAX91445	----N---DSPAN-QQ	-----A-TS---L---T-
	Mycobacterium shimoidei	WP_069396543	-----N---DSRAT-EQ	-----A-TS---L---T-
	Mycobacterium shinjukuense	WP_083049847	-----N-T-D-QAT-QQ	-----A-TS---L---T-
	Mycobacterium simiae	WP_061558588	-----N---DSQAT-QQ	-----A-TS---L---T-
	Mycobacterium smegmatis	WP_011728043	----N-EN-GQTT-AQ	-----A-TS---L---T-
	Mycobacterium szulgai	WP_085674308	-----N-T-DGQAT-QQ	-----A-TS---L---T-
	Mycobacterium triplex	WP_036467404	----N---DSQAT-QQ	-----A-TS---L---T-
	Mycobacterium triviale	WP_069393366	----E--P-N-PAT-KQ	----QA-TS---L---T-
	Mycobacterium tuberculosis	WP_064314163	-----N---DSPAT-QQ	-----A-TS---L---T-
	Mycobacterium tusciae	WP_040538682	--------D-QTT-QQ	--S--A-TS---L---T-
	Mycobacterium ulcerans	WP_011740463	-----N-T-DGEAT-QQ	-----A-TS---L---T-
	Mycobacterium vaccae	WP_003929184	-E----P-NTAAT-QQ	-----A-TS---------
	Mycobacterium vanbaalenii PYR-1	A1T636	-E----P-N-TAT-QQ	-----A-TS---------
	Mycobacterium vulneris	CDO27584	----N-ESNGQTT-AQ	-----A-TS---L---T-
	Mycobacterium wolinskyi	WP_067856414	----N-ESSGQTT-AQ	-----A-TS---L---T-
	Mycobacterium xenopi	WP_085196643	--S--N---DSQAT-QQ	-----A-TS---L---T-

Supplementary Figure 32

A partial sequence alignment of a conserved region of a hypothetical protein showing a six amino acid insertion that is specific for members of the "*Abscessus-Chelonae"* clade and absent in other *Mycobacterium*.

			808		844
**"Abscessus-Chelonae" Clade (6/6)**	*Mycobacterium abscessus*	WP_052613689	GPKLVNNAITTDNQVSSHVGII		KNQNILKWGNLLTLP
	Mycobacterium abscessus subsp. bolletii	EHM16461	----------------------		---------------
	Mycobacterium chelonae	WP_070919384	----------------------		------R--------
	Mycobacterium franklinii	WP_070938762	----------------------		------R--------
	Mycobacterium immunogenum	WP_064628010	-------------L--------		---------------
	Mycobacterium saopaulense	WP_070909629	----------------------		---------------
**Other *Mycobacterium* (0/>100)**	*Mycobacterium acapulcensis*	WP_083997650	----AF---S--TA--QDL-V-	GR	D---RIR--------
	Mycobacterium africanum	WP_031701648	----A-------PA--QDL-V-	GR	D---RIR--------
	Mycobacterium algericum	WP_083035837	----A-------TA--QDL-V-	GR	D---RIR--------
	Mycobacterium alsense	WP_083140568	----A-------PA--QDL-V-	GR	D---RIR--------
	Mycobacterium angelicum	WP_083111069	----A-------PA--QDL-V-	GR	D---RI---------
	Mycobacterium aromaticivorans	WP_036340069	----AF---S--TA--QDL-V-	GR	D---RIR--------
	Mycobacterium arosiense	WP_083065534	----A-------PA--QDL-V-	GR	D---RIR--------
	Mycobacterium arupense	WP_046188452	----A-------TT--QDL-V-	GR	D---RIR--------
	Mycobacterium asiaticum	WP_065035308	----A-------PA--QDL-V-	GR	D---RIR--------
	Mycobacterium aurum	WP_087020426	----AF---S--TA--TEL-Q-	GR	DG--RIR--------
	Mycobacterium avium	WP_062887272	----A-------PA--QDL-V-	GR	D---RIR--------
	Mycobacterium avium subsp. avium	EUA28116	----A-------PA--QDL-V-	GR	D---RIR--------
	Mycobacterium avium subsp. hominissuis	ETB32463	----A-------PA--QDL-V-	GR	D---RIR--------
	Mycobacterium avium subsp. paratuberculosis	ETB52791	----A-------PA--QDL-V-	GR	D---RIR--------
	Mycobacterium avium subsp. silvaticum	ETB14850	----A-------PA--QDL-V-	GR	D---RIR--------
	Mycobacterium bacteremicum	WP_083057290	----AF---S--TA--TEL-Q-	GR	DG--RIR--------
	Mycobacterium boenickei	WP_077743615	---QAF---S--TA-TQDL-V-	GR	D-L-RIR--------
	Mycobacterium bohemicum	WP_085181997	----A-------PA--QDL-V-	GR	D---RIR--------
	Mycobacterium bovis	WP_019283731	----A-------PA--QDL-V-	GR	D---RIR--------
	Mycobacterium branderi	WP_083129858	----A-------TA--QDL-V-	GR	D---RIR--------
	Mycobacterium brisbanense	WP_062831658	----AF---S--TA--QDL-V-	GR	D---RIR--------
	Mycobacterium canariasense	GAS93131	----AF---S--TA--QDL-V-	GR	D---RIR--------
	Mycobacterium canettii	WP_015291267	----A-------PA--QDL-V-	GR	D---RIR--------
	Mycobacterium celatum	WP_085167690	----A-------TA--QDL-V-	GR	D---RIR--------
	Mycobacterium celeriflavum	WP_083006383	----AF---S--TA--QDL-V-	GR	D---RIR--------
	Mycobacterium chlorophenolicum	KMO75863	----AF---S--TA--QDL-V-	GR	D---RIR--------
	Mycobacterium chubuense	WP_041781791	----AF---S--TA--QDL-V-	GR	D---RIR--------
	Mycobacterium colombiense	WP_064877082	----A-------PA--QDL-V-	GR	D---RIR--------
	Mycobacterium conceptionense	WP_085140998	---QAF---S--TA-TQDL-V-	GR	D-L-RIR--------
	Mycobacterium confluentis	WP_085148630	----AFT--S--TA-TQDL-V-	GR	D---RIR--------
	Mycobacterium conspicuum	WP_085231379	----A-------PD--TDL-Q-	GR	D---RI---------
	Mycobacterium cosmeticum	CDO10255	----AF---S--TA--QDL-V-	GR	D---RIR--------
	Mycobacterium diernhoferi	WP_073857814	----AF---S--TA--TEL-Q-	GR	DG--RIR--------
	Mycobacterium doricum	WP_085190019	----AF---S--TA--QDL-V-	GR	D---RIR--------
	Mycobacterium elephantis	WP_083042562	----AF---S--TA--QDL-V-	GR	D---RIR--------
	Mycobacterium engbaekii	WP_085129314	----A-------TT--QDL-V-	GR	D---RIR--------
	Mycobacterium europaeum	WP_085241601	----A-------PA--QDL-V-	GR	D---RIR--------
	Mycobacterium fallax	WP_085092481	----AF--MS--TT--QDL-M-	GR	D---RIR--------
	Mycobacterium farcinogenes	CDP84269	---QAF---S--TA-TQDL-V-	GR	D-L-RIR--------
	Mycobacterium flavescens	WP_069416781	----AF---S--TA--QDL-V-	GR	D---RIR--------
	Mycobacterium florentinum	WP_085223062	----A-------PA--QDL-V-	GR	D---RIR--------
	Mycobacterium fortuitum	WP_061262691	---QAF---S--TA-TQDL-V-	GR	D-L-RIR--------
	Mycobacterium fragae	WP_085195828	----A-------TA--QDL-V-	GR	D---RI---------
	Mycobacterium gastri	WP_085104988	----A-------PA--QDL-V-	GR	D---RIR--------
	Mycobacterium genavense	WP_025735717	----A-------PA--QDL-V-	GR	D---RIR--------
	Mycobacterium gilvum	WP_011895490	----AF---S--TAI-TEL-Q-	GR	DG--RIR--------
	Mycobacterium goodii	WP_049747760	----AF---S--TA--QDL-V-	GR	D---RIR--------
	Mycobacterium gordonae	WP_065047223	----A-------PA--QDL-V-	GR	D---RIR--------
	Mycobacterium haemophilum	WP_047313259	----A-------PA--QDL-V-	GR	D---RIR--------
	Mycobacterium hassiacum	WP_036447587	----AF---S--TA--QDL-V-	GR	D---RIR--------
	Mycobacterium heckeshornense	WP_048891255	----A-------TA--QDL-V-	GR	D---RIR--------
	Mycobacterium heidelbergense	WP_083073123	----A-------PA--QDL-V-	GR	D---RIR--------
	Mycobacterium heraklionense	WP_064998289	----A-------TT--QDL-V-	GR	D---RIR--------
	Mycobacterium hiberniae	WP_085133936	----A-------TT--QDL-V-	GR	D---RIR--------
	Mycobacterium holsaticum	WP_069403956	----AF---S--TA--QDL-V-	GR	D---RIR--------
	Mycobacterium icosiumassiliensis	WP_067970901	----A-------TT--QDL-V-	GR	D---RIR--------
	Mycobacterium insubricum	WP_083030763	----AF---N--PT-TA-L-L-	GR	D---RI-F-------
	Mycobacterium interjectum	WP_085201614	----A-------PA--QDL-V-	GR	D---RIR--------

Other *Mycobacterium* (0/>100)

Mycobacterium intermedium	WP_069419056	----A-------PA--QDL-V- GR D---RI---------
Mycobacterium intracellulare	WP_064938499	----A-------PA--QDL-V- GR D---RIR--------
Mycobacterium iranicum	WP_064283197	----AF---S--TA--QDL-V- GR D---RIR--------
Mycobacterium kansasii	WP_063467948	----A-------PA--QDL-V- GR D---RIR--------
Mycobacterium komanii	CRL78140	----AF---S--TA--QDL-V- GR D---RIR--------
Mycobacterium koreense	WP_085302486	----A-------TA--QDL-V- GR D---RIR--------
Mycobacterium kumamotonense	WP_065287459	----A-------TA--QDL-V- GR D---RIR--------
Mycobacterium kyorinense	WP_045384864	----A-------TA--QDL-V- GR D---RIR--------
Mycobacterium lacus	WP_085157001	----A-------PA--QDL-V- GR D---RIR--------
Mycobacterium lentiflavum	CQD08526	----A-------PA--QDL-V- GR D---RIR--------
Mycobacterium leprae	WP_010907856	----A-------PA--QDL-V- GR D---RIR--------
Mycobacterium lepromatosis	WP_045842624	----A-------PA--QDL-V- GR DD--RIR--------
Mycobacterium liflandii	WP_015354907	----A-------PA--QDL-V- GR D---RIR--------
Mycobacterium litorale	WP_078020415	----AF---S--TA--QDL-V- GR D---RIR--------
Mycobacterium llatzerense	WP_071286862	----AF---S--TA--QDL-V- GR D---RIR--------
Mycobacterium longobardum	WP_085264345	----A-------TT--QEL-L- GR D---RIR--------
Mycobacterium mageritense	WP_043367677	---QAF---S--TA-TQDL-V- GR D-L-RIR--------
Mycobacterium malmesburyense	CRL77529	----AF---S--TA--QDL-V- GR D---RIR--------
Mycobacterium malmoense	WP_065445036	----A-------PA--QDL-V- GR D---RIR--------
Mycobacterium mantenii	WP_083096129	----A-------PA--QDL-V- GR D---RIR--------
Mycobacterium marinum	WP_012393237	----A-------PA--QDL-V- GR D---RIR--------
Mycobacterium marseillense	WP_083020076	----A-------PA--QDL-V- GR D---RIR--------
Mycobacterium minnesotense	WP_083027533	----A-------TT--QDL-V- GR D---RIR--------
Mycobacterium moriokaense	WP_083154147	----AF---S--TA--QDL-V- GR D---RIR--------
Mycobacterium mucogenicum	WP_064860812	----AF---S--TA--QDL-V- GR D---RIR--------
Mycobacterium nebraskense	WP_085165081	----A-------PA--QDL-V- GR D---RIR--------
Mycobacterium neoaurum	CDQ46083	----AF---S--TA--TEL-Q- GR DG--RIR--------
Mycobacterium neworleansense	CRZ15539	---QAF---S--TA-TQDL-V- GR D-L-RIR--------
Mycobacterium nonchromogenicum	WP_085137944	----A-------TT--QDL-V- GR D---RIR--------
Mycobacterium noviomagense	WP_083086255	----A-------TA--QDL-V- GR D---RIR--------
Mycobacterium novocastrense	WP_067395782	----AF---S--TA--QDL-V- GR D---RIR--------
Mycobacterium obuense	KMO80355	----AF---S--TA--QDL-V- GR D---RIR--------
Mycobacterium palustre	WP_085077998	----A-------PA--QDL-V- GR D---RIR--------
Mycobacterium paraense	WP_085103820	----A-------PA--QDL-V- GR D---RIR--------
Mycobacterium paraffinicum	WP_073876024	----A-------PA--QDL-V- GR D---RIR--------
Mycobacterium parafortuitum	WP_083143055	----AF---S--TA--TEL-Q- GR DG--RIR--------
Mycobacterium parascrofulaceum	WP_007168175	----A-------PA--QDL-V- GR D---RIR--------
Mycobacterium paraseoulense	WP_083170110	----A-------PA--QDL-V- GR D---RIR--------
Mycobacterium parmense	WP_085267731	----A-------PA--QDL-V- GR D---RIR--------
Mycobacterium peregrinum	WP_064878842	---QAF---S--TA-TQDL-V- GR D-L-RIR--------
Mycobacterium persicum	WP_083153879	----A-------PA--QDL-V- GR D---RIR--------
Mycobacterium phlei	AMO60535	----AF---S--TA--QDL-V- GR D---RIR--------
Mycobacterium porcinum	WP_075921444	---QAF---S--TA-TQDL-V- GR D-L-RIR--------
Mycobacterium pseudoshottsii L15	GAQ36875	----A-------PA--QDL-V- GR D---RIR--------
Mycobacterium rhodesiae	WP_083121063	----AF---S--TA--QDL-V- GR D---RIR--------
Mycobacterium riyadhense	WP_085249736	----A-------PA--QDL-V- GR D---RI---------
Mycobacterium rufum	KGI67404	----AF---S--TA--QDL-V- GR D---RIR--------
Mycobacterium rutilum	SEH90102	----AF---S--TA--QDL-V- GR D---RIR--------
Mycobacterium saskatchewanense	WP_085257921	----A-------PA--QDL-V- GR D---RIR--------
Mycobacterium scrofulaceum	WP_067277590	----A-------PA--QDL-V- GR D---RIR--------
Mycobacterium senuense	WP_085081726	----A-------TA--QDL-V- GR D---RIR--------
Mycobacterium septicum	WP_044516826	---QAF---S--TA-TQDL-V- GR D-L-RIR--------
Mycobacterium setense	WP_064871743	---QAF---S--TA-TQDL-V- GR D-L-RIR--------
Mycobacterium sherrisii	WP_069402474	----A-------PA--QDL-V- GR D---RIR--------
Mycobacterium shigaense	BAX91445	----A-------PA--QDL-V- GR D---RIR--------
Mycobacterium shimoidei	WP_069396543	----A-------TA--QDL-V- GR D---RIR--------
Mycobacterium shinjukuense	WP_083049847	----A-------PA--QDL-V- GR D---RIR--------
Mycobacterium simiae	WP_061558588	----A-------PA--QDL-V- GR D---RIR--------
Mycobacterium sinense	WP_041318415	----A-------TA--QDL-V- GR D---RIR--------
Mycobacterium smegmatis	WP_011728043	----AF---S--TA--QDL-V- GR D---RIR--------
Mycobacterium szulgai	WP_085674308	----A-------PA--QDL-V- GR D---RI---------
Mycobacterium terrae	WP_085261981	----A-------TA--QDL-V- GR D---RIR--------
Mycobacterium thermoresistibile	WP_003927921	----A-------TA--QDL-V- GR DG--RIQF-------
Mycobacterium triplex	WP_036467404	----A-------PA--QDL-V- GR D---RIR--------
Mycobacterium triviale	WP_069393366	----A-------TA--QDL-V- GR D---RIR--------

**Other *Mycobacterium* (0/>100)**	*Mycobacterium tuberculosis*	WP_064314163	----A-------PA--QDL-V- GR D---RIR--------
	Mycobacterium tusciae	WP_083125071	----AF---S--TA--QDL-V- GR D---RIR--------
	Mycobacterium ulcerans	WP_011740463	----A-------PA--QDL-V- GR D---RIR--------
	Mycobacterium vaccae	WP_003929184	----AF---S--TA--QDL-V- GR D---RIR--------
	Mycobacterium vanbaalenii PYR-1	A1T636	----AF---S--TA--QDL-V- GR D---RIR--------
	Mycobacterium vulneris	WP_085292559	----A-------PA--QDL-V- GR D---RIR--------
	Mycobacterium wolinskyi	WP_085144909	----AF---S--TA--QDL-V- GR D---RIR--------
	Mycobacterium xenopi	WP_085196643	----A-------TA--QDL-V- GR D---RIR--------
	Mycobacterium yongonense	WP_065498511	----A-------PA--QDL-V- GR D---RIR--------

Supplementary Figure 33

A partial sequence alignment of a conserved region of a hypothetical protein showing a two amino acid deletion that is specific for members of the "*Abscessus-Chelonae"* clade and absent in other *Mycobacterium.*

			359		399
**"Abscessus-Chelonae" Clade (5/5)**	*Mycobacterium abscessus*	WP_057138049	DVFNMTTGEFRKSIVVQRNTA	DE	KSPVISAVVGNTLVEQRG
	Mycobacterium chelonae	WP_070915156	--------------A-H-DP-	--	-G----------------
	Mycobacterium franklinii	WP_070938732	--------------A---DP-	--	------------V-----
	Mycobacterium immunogenum	WP_064627985	------------N-A---DP-	--	----S-------V-----
	Mycobacterium saopaulense	WP_070909588	----T-------F-A---DP-	--	------------V-----
**Other *Mycobacterium* (0/52)**	Mycobacterium acapulcensis	WP_066810347	---DPA--QGERH-P-T-PPS		AA--VP--A-SMVI----
	Mycobacterium asiaticum	WP_065035330	G-YDPVS--SSRF-P---QPN		S----P--A-SRVI----
	Mycobacterium aurum	WP_048630371	---DPD--TGD-H-PL--PPV		EG--VP--A-S--L----
	Mycobacterium austroafricanum	WP_036369453	---DPM--TGD-H-P---PQV		AG--VP--A-S-VL----
	Mycobacterium avium	WP_043336654	G---QR--APERV-P-S-PPG		V-A-FP--S-P-VL----
	Mycobacterium bohemicum DSM 44277	CPR12092	G-YDPI--ANERD-P-D-APG		T-A-VP--A-SKVF----
	Mycobacterium bovis BCG	AMC52345	G-YDPVS-ANNRY-P-T-PPS		T-A--P--S-SRVI----
	Mycobacterium brisbanense	WP_062831646	---DPG--AGERH-ALT-QPS		AE--VP--A-S--L----
	Mycobacterium canariasense	WP_062654505	----PE--AGVRH-D-K-PPS		QD---P--A-S--L----
	Mycobacterium canettii	WP_015291284	G-YDPVS-ANNRY-P-T-PPS		T-A--P--S-SRVI----
	Mycobacterium celatum	WP_062540615	G-YDPA--AHERD-P-D-PPG		TA---P--S-SQIL----
	Mycobacterium chlorophenolicum	WP_048471363	---DPA--AGERH-PL--PA-		-G---P--A-T--L----
	Mycobacterium chubuense	WP_014814687	-L-DPN--APDRH-PLE-PPV		RG--VP--A-SAVL-K--
	Mycobacterium cosmeticum	WP_036402232	----PE--AGVRH-DLK-PPS		RD---P--A-S--L----
	Mycobacterium flavescens	WP_069415949	---DAQ--RGERH-ALD-PD-		ST--VP--A-A-VL----
	Mycobacterium fortuitum	WP_064849267	-I-DQ---AGQRH-ALP-TPS		VA--VP--A-D-VL----
	Mycobacterium genavense	WP_025735738	G-YDPVS-ANERY-P-D-APS		NRA-VP--S-SRVF----
	Mycobacterium gilvum	WP_041788062	---DPE--TGE-H-P---PPV		DG--VP--A-T-VL-L--
	Mycobacterium goodii	WP_049747788	---DPA--TGDRH-SLP-QPS		V---VP--A-SIVL----
	Mycobacterium hassiacum	WP_005625923	---DAES-AGERH-P-P-DAT		AG--VP--A-ELI-----
	Mycobacterium heckeshornense	WP_048891236	G-YDPL--ANERYLP-N-PAG		T-A-MP--C-SQVI----
	Mycobacterium heraklionense	WP_064999861	G--DQK--APERI-P-S-PPG		V-S-FP--S-P-VL----
	Mycobacterium holsaticum	WP_069406047	-L-DPA--QGDRH-A-S-PPH		SA--VP--A-SL-I----
	Mycobacterium houstonense	WP_066903418	---DPES-TGQRH-ALP-TPS		V---VP--A-D--L----
	Mycobacterium iranicum	WP_064283176	---DPA--AGD-H-PL--PAG		DG--VP--A-A--L----
	Mycobacterium kumamotonense	WP_065287471	G---QR--APERV-P-S-PPG		V-A-FP--S-P-VL---D
	Mycobacterium kyorinense	WP_065016612	G-YDPA--AGDRVLP-N-TPI		P----P--L-SQI-----
	Mycobacterium lentiflavum	CQD08382	G-YDPM--ANERY-P-E-APS		TRA-VP--S-SLVF----
	Mycobacterium mageritense	WP_036428324	---DPA--KGERH-PLS-PPS		V---VPTPA-S--L----
	Mycobacterium neoaurum	CDQ46110	---DPE--TGIRH-DLA-DP-		DTA-VP-TS-S-II----
	Mycobacterium neworleansense	CRZ15572	---DQQS-TGQRH-ALP-TPS		V---VP--A-DI-L----
	Mycobacterium novocastrense	WP_067395809	---DPA--RGERH-PLT-PPS		AA--VP--A-SMVL----
	Mycobacterium obuense	WP_046364809	G--DPVS-AGERH-AL--PPV		RG--VP--A-AM------
	Mycobacterium peregrinum	WP_055112090	---DQE--AGQRH-ALP-TPS		V---VPVAA-D-VL----
	Mycobacterium phlei	WP_061481531	---DPD--KGERH-PLA-PAV		DG--VPG-A-SV-L----
	Mycobacterium rhodesiae	WP_014208815	---DPL--KGDRH-PL--PPS		SDA-VP--A-S-VL----
	Mycobacterium rufum	KGI67383	---DPA--AGDRH-AL--PAV		DG--VP--A-SM-L----
	Mycobacterium rutilum	SEH90366	---DAQ--RGERH-ALD-AGS		PT--VP--A-T-V-----
	Mycobacterium septicum	WP_044516770	---DQQ--TGQRH-ALP-TPS		V---VPV-A-D-VL----
	Mycobacterium shimoidei	WP_069396568	G-YDPI--ANERYLP-K-EPY		NG--TL--S-SQIL----
	Mycobacterium simiae	WP_061558575	G-YDPV--ANERY-P-H-TPS		DA--VP--S-SRVF----
	Mycobacterium sinense	WP_064855428	G--DQY--A-ERV-P-S-PPG		VAA-FP--S-P-VL----
	Mycobacterium smegmatis	WP_011728019	---DPE--TGDRH-SLP-TPS		V---VP--A-SIVL----
	Mycobacterium szulgai	WP_068029221	G-YDPVS-ADLRY-P-E-PPS		SA--VP--S-SKVI----
	Mycobacterium thermoresistibile	WP_003927944	---DPLS-AGERH-PLT-PQQ		Q-A-FPL-A-T-------
	Mycobacterium tuberculosis	WP_069984969	G-YDPVS-ANNRY-P-T-PPS		T-A--P--S-SRVI----
	Mycobacterium tusciae	WP_006245873	---DPDS-KGDRH-P-P-PPS		AD--VP--A-S-V-----
	Mycobacterium vaccae	WP_003929159	---DPM--TGDRHVPL--PNL		DG--VP--A-S--L----
	Mycobacterium vanbaalenii	WP_011779030	---DPM--TGD-H-P---PQV		AG--VP--A-S-VL----
	Mycobacterium vulneris	WP_065516746	-L-DPQ--TGQRH-ALP-TPS		V---VPVAA-D-VL----
	Mycobacterium wolinskyi	WP_067854460	---DPA--TGERH-A-A-EPS		V---VP--A-S-VL----
	Mycobacterium xenopi	WP_003920834	G-YDPL--ANERYLP-N-APG		T-A-MP--S-SHVI----

Supplementary Figure 34

A partial sequence alignment of a conserved region of a hypothetical protein showing a two amino acid insertion that is specific for members of the "*Abscessus-Chelonae"* clade and absent in other *Mycobacterium*.

Clade	Species	Accession	199 … 235
"*Abscessus-Chelonae*" Clade (6/6)	*Mycobacterium abscessus*	WP_052618664	TELVYKLLSGPRPELAG A VRNHLN GLRLRGPVTRVDG
	Mycobacterium abscessus subsp. bolletii	EHM16826	----------------- - ------ -------------
	Mycobacterium chelonae	WP_070920778	---------------V- - -----S -------L-----
	Mycobacterium franklinii	WP_070938706	--------A------G- - -----S -------------
	Mycobacterium immunogenum	WP_064627961	---------------S- - -----S ----------A--
	Mycobacterium saopaulense	WP_070909565	----------------- - ------ -------------
"*Terrae*" Clade (12/12)	*Mycobacterium algericum*	WP_083035895	----S--IA-----M-- ---L-A A P---------A--
	Mycobacterium arupense	WP_046190402	----S--IA-----M-- ---L-A P PV--------A--
	Mycobacterium engbaekii	WP_085128020	----S--IA-----M-- ---L-S A PV--------A--
	Mycobacterium heraklionense	WP_064888065	----S--IA-----M-- ---L-S P PV--------A--
	Mycobacterium hiberniae	WP_085134274	----S--IA-----M-- ---L-S A PV--------A--
	Mycobacterium icosiumassiliensis	WP_067971119	----S--IA-----M-- ---L-A P P---------A--
	Mycobacterium longobardum	ORW07034	----S--IA-----M-- ---L-G P P---------A--
	Mycobacterium minnesotense	WP_083023996	----S--IA-----M-- ---L-A P P---------A--
	Mycobacterium nonchromogenicum	WP_085137876	----S--IA-----M-- ---L-A P PV--------A--
	Mycobacterium senuense	WP_085085030	----S--IA-----M-- ---L-A A P---------A--
	Mycobacterium sinense	WP_064856517	----S--IA-----M-- ---L-A T P---------A--
	Mycobacterium terrae	WP_085259085	----S--IA-----M-- ---L-A A P---------A--
Other *Mycobacterium* (1/>100)	*Mycobacterium aromaticivorans*	WP_036340239	---LT--IA-----M-R T ---L-G PVKMM-----A--
	Mycobacterium koreense	WP_085302544	----S-MIA-------R S ---L-G A P---N-----A--
	Mycobacterium triviale	WP_085110721	----S--VA-----MER S ---L-A S P---N-----A--
	Mycobacterium africanum	WP_061846093	----S---A-----M-R T ---L-A P P---------A--
	Mycobacterium alsense	WP_083138669	---IS-M-A-----M-H T ---L-A P P---------A--
	Mycobacterium arosiense	WP_083067416	---IS--IA-----M-H T ---L-A P P---------A--
	Mycobacterium asiaticum	WP_065122898	---IS---A-----M-H T ---L-A P P---------A--
	Mycobacterium aurum	WP_087020256	----T--IA-----M-R T ---V-A A P---------A--
	Mycobacterium austroafricanum	WP_036369505	----S--VA-----MDK T ---L-G S P---------A--
	Mycobacterium avium	WP_084021992	----S--IA-----M-H T ---L-A P P---------A--
	Mycobacterium avium subsp. paratuberculosis	Q93CB6	----S--IA-----M-H T ---L-A P P---------A--
	Mycobacterium bacteremicum	WP_083057376	---MS--IT-----M-H T ---L-A A PM--------A--
	Mycobacterium bohemicum	ORU97565	---IS--VA-----M-H T ---L-A P P---------A--
	Mycobacterium bovis	WP_031702833	----S---A-----M-R T ---L-A P P---------A--
	Mycobacterium branderi	WP_083130470	----S--IA-----IGK - -H-M-G P PV--------A--
	Mycobacterium brisbanense	WP_062831587	----T--IA-----M-K S -H-L-G A Q-K-------A--
	Mycobacterium canariasense	WP_062654475	----T--IT-----M-R T ---L-A A P---------A--
	Mycobacterium canettii	WP_015291309	----S---A-----M-R T ---L-A P P---------A--
	Mycobacterium celatum	WP_085168669	----S--IA-----IGK - -H-M-G P PV--------A--
	Mycobacterium celeriflavum	WP_083004888	----S--IA-----M-K T ---L-E P P-K-------A--
	Mycobacterium chlorophenolicum	WP_048472010	----S--IV-----MEK T ---L-G A P---------A--
	Mycobacterium chubuense	WP_014814654	---IS--IA-----M-K T ---L-G P P---------A--
	Mycobacterium colombiense	WP_064877161	---IS--IA-----M-H T ---L-A P P---------A--
	Mycobacterium conceptionense	WP_064895261	---IS--VA-----M-R S ---L-D A P--M------A--
	Mycobacterium confluentis	WP_085148781	----S--IT-------N S ---M-G P PV--------A--
	Mycobacterium conspicuum	WP_085231465	----S---A-----M-H T ---L-A P P---------A--
	Mycobacterium diernhoferi	WP_073857724	----T--IA-----M-R T ---L-A A P---------A--
	Mycobacterium doricum	WP_085190113	----S--I------M-N T ---L-G S P-A-------A--
	Mycobacterium elephantis	KKW64027	----S--IA-----M-N T ---L-G P P---------A--
	Mycobacterium europaeum	WP_085241652	----S--IG-----M-H T ---L-A P P---------A--
	Mycobacterium fallax	WP_085095209	----S--IA------EH S ---LAG A P-K-S-----A--
	Mycobacterium flavescens	WP_069415920	----S--IA-----MVN T ---L-G P P-K-------A--
	Mycobacterium florentinum	WP_085220030	---IS---A-----M-H T ---L-A P P---------A--
	Mycobacterium fortuitum	WP_064925845	---IS--VA-----M-R T ---L-D A P---------A--
	Mycobacterium fragae	WP_085195973	----S--IA-----M-K S -H-M-G P P---------A--
	Mycobacterium gastri	WP_085104990	----S---------MTH T ---L-A P P---------A--
	Mycobacterium genavense	WP_036468412	----S---A-----M-H T ---L-A P P---------A--
	Mycobacterium gilvum	WP_011895545	----S--IT-----MDK T ---L-G S P---------A--
	Mycobacterium goodii	WP_049747824	----S--I------MER S ---L-D P P-K-------A--
	Mycobacterium gordonae	WP_065048901	---IS---A-----M-H T ---L-A P P---------A--
	Mycobacterium haemophilum	WP_047313324	----S---A-----M-H T ---L-A P P---------A--
	Mycobacterium hassiacum	WP_005625854	----S--IT-----M-N - ---L-G P P-K-------A--
	Mycobacterium heckeshornense	WP_071700338	----S--IA-----MSR - ---L-A P P---------A--
	Mycobacterium heidelbergense	WP_083073171	----S---A-----M-H T ---L-A P P---------A--
	Mycobacterium holsaticum	WP_084222991	----S--IA-----M-N T ---L-G P P-K-------A--
	Mycobacterium insubricum	WP_083032663	----S--IA------SH - ---MAG A P-K-------A--
	Mycobacterium interjectum	WP_066908218	----S-M-A-----M-H T ---L-A P P---------A--
	Mycobacterium intermedium	WP_069418984	----S---A-Q---M-H T ---L-S P P---------A--
	Mycobacterium intracellulare	WP_036397692	----S-VIA-----M-H T ---L-A P P---------A--
	Mycobacterium iranicum	WP_064282102	----S--IA-----MDK T ---L-G A P---------A--
	Mycobacterium kansasii	WP_063468342	----S---------MTH T ---L-A P P---------A--
	Mycobacterium komanii	CRL78251	----S--IA-----MVK T ---L-D P P-K-------A--

Other *Mycobacterium* (1/>100)

Mycobacterium kubicae	WP_085072806	----S-V-A-----MVH S ---L-S P P---------A--
Mycobacterium kyorinense	WP_065016640	---MS--IA-----MSK S -H-M-G P PV--------A--
Mycobacterium lacus	WP_085161185	---IS---A-A---M-H - ---L-A P P---------A--
Mycobacterium lentiflavum	CQD08080	---IS---A-----M-H T ---L-A P P---------A--
Mycobacterium leprae	WP_010907923	----S---A-----M-H - ---L-A P P---------A--
Mycobacterium lepromatosis	KJX75535	----S---A-----M-H - ---L-A P P---------A--
Mycobacterium liflandii	WP_041300087	----S---A-----M-H S ---L-A P P---------A--
Mycobacterium litorale	WP_078020460	---IT--IA-----M-R T ---LMG A P-KMV-----A--
Mycobacterium mageritense	WP_036428425	----S--IA-----MDK S ---M-G A P-K-------A--
Mycobacterium malmesburyense	CRL72449	----S--IA-----MVK T ---L-D P P-K-------A--
Mycobacterium malmoense	WP_065444960	----S--IA-----M-H T ---L-A P P---------A--
Mycobacterium mantenii	WP_083097854	---IS-VIA-----M-H T ---L-A P P---------A--
Mycobacterium marinum	WP_036451453	----S---A-----M-H S ---L-A P P---------A--
Mycobacterium microti	AMC61007	----S---A-----M-R T ---L-A P P---------A--
Mycobacterium moriokaense	WP_083154429	----S--IA-------K S ---L-G P P-K-------A--
Mycobacterium mucogenicum	WP_064858410	----T---------M-S S ---LMA A P---------A--
Mycobacterium nebraskense	WP_046186942	----S--IA-----M-H T ---L-A P P---------A--
Mycobacterium neoaurum	WP_030134851	---MS-MIT-----M-H T ---L-A A P---------A--
Mycobacterium neworleansense	CRZ15614	---IS--VA-------R S ---L-D A P---------A--
Mycobacterium noviomagense	WP_083086371	----S-VIA-----M-R - ---L-S P P---------A--
Mycobacterium obuense	WP_082163913	----S--IA-----MDK T ---L-G A P---------A--
Mycobacterium palustre	WP_085080170	----S-M-A-----M-H T ---L-A P P---------A--
Mycobacterium paraense	WP_085096021	----S-M-A-----M-H T ---L-A P P---------A--
Mycobacterium paraffinicum	WP_073876115	----S--IA-----M-H T ---L-A P P---------A--
Mycobacterium parafortuitum	WP_083143104	----S--IA-----MDK T ---L-G P P---------A--
Mycobacterium parascrofulaceum	WP_007168099	----S--IA-----M-H T ---L-A P P---------A--
Mycobacterium paraseoulense	WP_083169927	----S--IA-----M-H T ---L-A P P-------A-A--
Mycobacterium parmense	WP_085267626	---IS-MIA-----M-H T ---L-A P P---------A--
Mycobacterium peregrinum	WP_064886427	---IS--V------I-R - ---L-D A P---------A--
Mycobacterium phlei	WP_003886318	----S--IA-----M-H T ---L-G P P-K-------A--
Mycobacterium porcinum	WP_075921198	---IS--VA-----M-R S ---L-D A P--M------A--
Mycobacterium pseudoshottsii	WP_086085455	----S---T-----M-H S ---L-A P P---------A--
Mycobacterium rhodesiae	WP_014208856	----S--IA-------N S ---L-G P P-K-------A--
Mycobacterium riyadhense	WP_085249842	----S---A-----M-H T ---L-A P P---------A--
Mycobacterium rufum	KGI67344	----S--IA-----MEK T ---L-G P P----N----A--
Mycobacterium rutilum	WP_083409900	----S--IA-----M-N T ---L-G P P-K-------A--
Mycobacterium saskatchewanense	WP_085254229	---IS--IA-----M-H T ---L-A P P---------A--
Mycobacterium scrofulaceum	WP_067268517	----S--IA-----M-H T ---L-A P P---------A--
Mycobacterium septicum	WP_044516702	---IS--VA-----MSR - ---L-E A P-Q-------A--
Mycobacterium setense	WP_039317593	---IS--VA-----M-R S ---L-D A P--M------A--
Mycobacterium sherrisii	WP_085166421	---IS---A-----M-H T ---L-A P P---------A--
Mycobacterium shigaense	BAX91357	----S-M-A-----M-H T ---L-A P P---------A--
Mycobacterium shinjukuense	WP_083047375	----S---A-----M-H - ---L-A P P---------A--
Mycobacterium simiae	AMP26314	---IS---A-----M-H T ---L-A P P---------A--
Mycobacterium smegmatis	WP_011727973	----S--IA-----MER S ---L-D P P-K-------A--
Mycobacterium szulgai	WP_068029072	----S---A-----MVH S ---L-S P P---------A--
Mycobacterium thermoresistibile	WP_003927972	----S--IV-----M-- T ---L-E P P---------A--
Mycobacterium triplex	CDO87357	----S---A-----M-H T ---L-A P P---------A--
Mycobacterium tuberculosis	WP_070898580	----S---A-----M-R T ---L-A P P---------A--
Mycobacterium tusciae	WP_083125016	----S--IA-------K S ---L-G P P-K-------A--
Mycobacterium ulcerans	WP_011740533	----S---A-----M-H S ---L-A P P---------A--
Mycobacterium vaccae	WP_040540368	----S--IA-----MDR T ---L-G P PM--------A--
Mycobacterium vanbaalenii	WP_011778997	----S--VA-----MDK T ---L-G S P---------A--
Mycobacterium vulneris	WP_085290337	---IS--IA-----M-H T ---L-A P P-------S-A--
Mycobacterium wolinskyi	WP_067854613	---IS--VA-----M-K S ---L-D A P-KM------A--
Mycobacterium xenopi	WP_085196697	----S--IA-----MSR S ---L-A P P---------A--
Mycobacterium yongonense	WP_065508395	----S--IA-----M-H T ---L-A P P---------A--

Supplementary Figure 35

A partial sequence alignment of a conserved region of a hypothetical protein showing a one amino acid deletion that is specific for members of the "*Abscessus-Chelonae"* clade and a one amino acid deletion that is specific for members of the "*Terrae"* clade that are both absent in most other *Mycobacterium*.

			318		354
**“Abscessus-Chelonae” Clade (5/5)**	*Mycobacterium abscessus*	WP_052543860	PVLVDADALTMLAEHP	DLA	DLVASRPAATVLTPHAGE
	Mycobacterium chelonae	WP_070915319	----------I-----	E--	----A-D-P-L-------
	Mycobacterium franklinii	WP_070938585	----------I----H	--T	E-----A-----------
	Mycobacterium immunogenum	WP_064627854	----------I-----	--T	E-----T-----------
	Mycobacterium saopaulense	WP_070909445	----------I-----	--T	----T-D-P---------
**Other *Mycobacterium* (2/>100)**	*Mycobacterium haemophilum*	WP_047315614	-------G-----A--	--V	TKR-E-GVP---------
	Mycobacterium parafortuitum	WP_083146458	---D--QWR-L-SNLD	R--	GI--A-GLTA--H--V-
	Mycobacterium acapulcensis	WP_066810503	--V----G--I--A--		---DG-E-----------
	Mycobacterium algericum	WP_083040233	--I-------I--A--		S---D-A-P---------
	Mycobacterium alsense	WP_083137304	--I-------I--A--		E-LE--A-P---------
	Mycobacterium angelicum	WP_083112989	--I----G-----A--		----N-D-P---------
	Mycobacterium aromaticivorans	WP_036340729	--V-------I-STQ-		----G-T-P---------
	Mycobacterium arosiense	WP_083065464	--I----G--I--A--		E---N-T-P---------
	Mycobacterium arupense	WP_083070994	--I-------I--A--		A---D-S-P---------
	Mycobacterium asiaticum	WP_065034629	-------G-----A--		E---D-T-P---------
	Mycobacterium aurum	WP_087019696	--I-------L--AE-		A---D-Q-P---------
	Mycobacterium avium	WP_065370826	--I----G-----A--		E---N-A-P---------
	Mycobacterium avium subsp. avium	EUA28414	--I----G--I--A--		E---E-G-P---------
	Mycobacterium bacteremicum	WP_083059229	--I-------L--RA-		Q---D-G-P---------
	Mycobacterium boenickei	WP_077743942	--I-------L--AD-		G-L-G-R-P---------
	Mycobacterium bohemicum	WP_085180173	--I----G--I--A--		E---N-V-P---------
	Mycobacterium bovis	WP_069523319	-------G-----D--		----G-N-P---------
	Mycobacterium branderi	WP_083130065	--I----G--I--A--		E---N-E-P---------
	Mycobacterium brisbanense	WP_062830084	-------G--L-SQD-		G---G-N-----------
	Mycobacterium canariasense	WP_062659917	--I-------L--GQ-		E--ER-S-P---------
	Mycobacterium canettii	WP_014001739	-------G-----D--		----G-N-P---------
	Mycobacterium celatum	WP_085168006	-------G--I--A--		----N-A-P------V--
	Mycobacterium celeriflavum	WP_083000946	--V----G--I--A--		---DG-A-P---------
	Mycobacterium chlorophenolicum	KMO71466	-------G--I--A--		----G-S-P---------
	Mycobacterium chubuense	WP_014814377	--I-------I-SA--		----N-R-P---------
	Mycobacterium colombiense	WP_064883705	--I----G--I--A--		E-----T-P---------
	Mycobacterium conceptionense	CQD06262	--I----G--L--AD-		R-L-G-S-P---------
	Mycobacterium confluentis	WP_085149352	--IA------I--RN-		-W-RG-ANP---------
	Mycobacterium conspicuum	WP_085233534	--I-------IV-A--		EVLVD-A-P---------
	Mycobacterium cosmeticum	WP_036401790	--I-------L--A--		E--EN-S-P---------
	Mycobacterium diernhoferi	WP_073856445	--I-------L--TE-		A--TD-R-P---------
	Mycobacterium doricum	WP_085192453	--I----G--L--A--		---VD-G-P---------
	Mycobacterium elephantis	WP_083043864	--I-------I--A--		----E-A-P---------
	Mycobacterium engbaekii	WP_085130085	--I-------I--A--		----D-S-P---------
	Mycobacterium europaeum	WP_085242172	--I----G--I--A--		E---N-K-P---------
	Mycobacterium fallax	WP_085092554	----------V--A--		E---D-A-P-L-------
	Mycobacterium farcinogenes	WP_036388967	--I----G--L--AD-		R-L-G-S-P---------
	Mycobacterium flavescens	WP_069416853	--I----G--I--A--		----G-N-P---------
	Mycobacterium florentinum	WP_085224618	-------G--I--A--		E---N-R-P-L-------
	Mycobacterium fortuitum	WP_061262607	--I----G--L--AE-		G-L-G-T-P---------
	Mycobacterium fragae	WP_085198366	--M-------I--A--		-M-E--R-P---------
	Mycobacterium gastri	WP_036410036	-------G---V-A--		E--VN-R-P---------
	Mycobacterium genavense	WP_025737516	-------G--I--A--		E-----H-P---------
	Mycobacterium gilvum	WP_013472651	--I----G--I--A--		EI-QG-T-P---------
	Mycobacterium gordonae	WP_065046531	-------G-----A--		----D-T-P---------
	Mycobacterium hassiacum	WP_005625564	--I----G--L--AD-		N---D-D-P---------
	Mycobacterium heckeshornense	WP_048890199	--I--G-G--L--A--		---TR-T-P-------R-
	Mycobacterium heidelbergense	WP_083075117	--I----G--I--A--		E--VN-D-P---------
	Mycobacterium heraklionense	WP_064887657	--I-------I--A--		A---E-N-P---------
	Mycobacterium hiberniae	WP_085135192	--I-------I--A--		A---D-S-P---------
	Mycobacterium holsaticum	WP_069404795	--I-------I--A--		---TD-A-P---------
	Mycobacterium icosiumassiliensis	WP_067970123	--I-------I--A--		S---D-N-P---------
	Mycobacterium insubricum	WP_083031992	----------L--A--		E--VD-S-P-L-------
	Mycobacterium interjectum	WP_085202397	--I----G--I--A--		E--VN-S-P---------
	Mycobacterium intermedium	WP_069417634	-------G-----A--		E---N-Q-P---------
	Mycobacterium intracellulare	WP_064893701	--I----G--I--A--		E---E-G-P---------
	Mycobacterium iranicum	WP_064283894	--I-------I-SA--		E---G-Q-P---------
	Mycobacterium kansasii	WP_063467687	-------G-----A--		E--VD-R-P---------
	Mycobacterium komanii	CRL75338	--V----G--I--A--		---DG-E-P---------
	Mycobacterium koreense	WP_085304758	--I-------L--A--		---TE-T-----------
	Mycobacterium kubicae	WP_085073768	---I---G--I--A--		-M--N-A-P---------
	Mycobacterium kumamotonense	WP_065287258	--I-------I--A--		A---G-S-P---------
	Mycobacterium kyorinense	WP_065013446	--I-------I--A--		E--SA-R-P---------
	Mycobacterium lacus	WP_085160729	-------G-----A--		E--TD-N-P---------
	Mycobacterium lentiflavum	CQD07107	-------G--I--A--		E---N-A-P-L-------

Other *Mycobacterium* (2/>100)

Mycobacterium leprae	AAA17298	-------G-----A--	---IN-N-P-------S-
Mycobacterium lepromatosis	WP_045842463	-------G-----A--	---VN-K-P---------
Mycobacterium liflandii	WP_041300061	--I----G-----A--	E--VN-S-P---------
Mycobacterium litorale	WP_078022223	--V----------A--	----N-A-P---------
Mycobacterium longobardum	WP_085263012	--I-------I--A--	S---D-T-P---------
Mycobacterium mageritense	WP_019347589	--I----G--L--AD-	R-L-G-S-P---------
Mycobacterium malmesburyense	CRL78433	--V-------I--A--	---DG-D-P---------
Mycobacterium malmoense	WP_065441534	--I----G--I--A--	E---N-N-P---------
Mycobacterium mantenii	WP_083093478	--I----G--I--A--	----N-T-P---------
Mycobacterium marinum	WP_020727372	--I----G-----A--	E--VN-S-P---------
Mycobacterium marseillense	WP_083019853	--I----G--I--A--	----G-S-P---------
Mycobacterium minnesotense	WP_083026717	--I-------IV-A--	A---D-A-P---------
Mycobacterium moriokaense	WP_083154081	--I-------I--A--	----N-S-P---------
Mycobacterium mucogenicum	WP_064859889	--I-------L--VDL	----D-T-P---------
Mycobacterium nebraskense	WP_085164135	--I----G--I--A--	E---N-T-P---------
Mycobacterium neworleansense	CRZ15953	--I----G--L--ADL	G-LTG-S-P---------
Mycobacterium noviomagense	WP_083087891	--I----G--L--A--	----G-D-P---------
Mycobacterium novocastrense	WP_067396212	--V----G--I--A--	---D--N-P---------
Mycobacterium obuense	WP_046676502	-------G--I--A--	----G-R-P---------
Mycobacterium palustre	WP_085079368	--I----G--I--A--	E---N-T-P---------
Mycobacterium paraense	WP_085096498	--I-------I--A--	E--VD-S-P---------
Mycobacterium paraffinicum	WP_073880326	--I----G--I--A--	E---N-T-P---------
Mycobacterium parascrofulaceum	WP_040622068	--I----G--I--A--	E---N-N-P---------
Mycobacterium paraseoulense	WP_083175426	--I----G--I--A--	E---N-T-P---------
Mycobacterium parmense	WP_085272088	--I----G--I--A--	E---N-T-P---------
Mycobacterium peregrinum	WP_064888066	--I----G--L--AE-	G-L-G-T-P---------
Mycobacterium persicum	WP_083153167	-------G-----A--	E-AVN-R-P---------
Mycobacterium phlei	WP_061481147	--I-------I--A--	--L-N-S-P---------
Mycobacterium porcinum	WP_075924173	--I----G--L--AD-	G-LSG-G-----------
Mycobacterium pseudoshottsii	WP_086084788	--I----G-----A--	E--VN-S-P---------
Mycobacterium rhodesiae	WP_083121700	--V-------I-STQ-	----A-S-P---------
Mycobacterium riyadhense	WP_085250272	-------G-----A--	G---N-D-P---------
Mycobacterium rutilum	WP_083410123	--I----G--IV-A--	---TG-D-P---------
Mycobacterium saskatchewanense	WP_085255615	--V----G--I--A--	E--VN-A-P---------
Mycobacterium scrofulaceum	WP_067280612	--V----G--I--A--	E---N-T-P---------
Mycobacterium septicum	WP_044519250	--I-------L--AD-	G-LSG-N-P---------
Mycobacterium setense	WP_064876179	--I----G-----AD-	GPLTG-S-P---------
Mycobacterium sherrisii	WP_069399192	-------G--I--A--	N---N-K-P-L-------
Mycobacterium shigaense	BAX91169	-------G--I--A--	E--VN-T-P---------
Mycobacterium shimoidei	WP_069395370	----------I--A--	--L-E-R-P---------
Mycobacterium shinjukuense	WP_083045868	-------G-----A--	---VN-T-P---------
Mycobacterium simiae	WP_061560066	-------G--I--A--	----N-K-P-L-------
Mycobacterium sinense	WP_064921394	--I-------I--A--	S---D-Q-P---------
Mycobacterium smegmatis	WP_011727745	--I----------AD-	AP-TG-S-P---------
Mycobacterium szulgai	WP_085672414	--I----G-----A--	----N-E-P---------
Mycobacterium terrae	WP_085261315	--I-------I--A--	S---D-K-P---------
Mycobacterium thermoresistibile	WP_003928047	-------G--L--AR-	E--RG-S-P---------
Mycobacterium timonense	WP_083187392	--I----G-----A--	E---N-A-P---------
Mycobacterium triplex	WP_036472120	-------G--I--A--	E-----R-P---------
Mycobacterium triviale	WP_069391095	--I-------L-SA--	----E-R-P---------
Mycobacterium tuberculosis	WP_069334375	-------G-----D--	----G-N-P---------
Mycobacterium tusciae	WP_083124807	--I----G--I--A--	----D-A-P---------
Mycobacterium ulcerans	WP_011739132	--I----G-----A--	E--VN-S-P---------
Mycobacterium vaccae	WP_003929238	--I----G--I--A--	E---D-A-P---------
Mycobacterium vulneris	WP_085292051	--I----G--I--A--	E---N-T-P---------
Mycobacterium wolinskyi	WP_067842619	--IA------L--ADS	GP-TD-S-P---------
Mycobacterium xenopi	WP_085197115	--I--G-G--L--A--	----R-E-P-------R-
Mycobacterium yongonense	WP_065499536	-AI----G--I--A--	E---E-S-P---------

Supplementary Figure 36

A partial sequence alignment of a conserved region of bifunctional ADP-dependent (S)-NAD(P)H-hydrate dehydratase/NAD(P)H-hydrate epimerase showing a three amino acid insertion that is specific for members of the "*Abscessus-Chelonae"* clade and absent in most other *Mycobacterium*.

			181		208
"Abscessus-Chelonae" Clade (7/7)	*Mycobacterium abscessus*	WP_052621243	LTGAAPTTSYTLYAVAGSL		LFPLGSAAL
	Mycobacterium abscessus subsp. bolletii	EUA81534	-------------------		---------
	Mycobacterium chelonae	WP_070917027	-------------------		----SA---
	Mycobacterium franklinii	WP_070937505	-------------S-----		-----A---
	Mycobacterium immunogenum	WP_043077370	-------------S-----		----CA---
	Mycobacterium salmoniphilum	WP_078330328	-------------------		----SA---
	Mycobacterium saopaulense	WP_070912186	-------------S-----		-----A---
Other *Mycobacterium* (0/>100)	*Mycobacterium africanum*	AMC66366	--------G---SSL-A-V	W	---VSA-V-
	Mycobacterium algericum	WP_083037999	--------G---AGL-A-V	W	---ISA-L-
	Mycobacterium alsense	WP_083138488	VA------G--IN-L-A-V	W	---S-A-T-
	Mycobacterium angelicum	WP_083114062	-----A--G---TSL-AAI	W	---VSA-V-
	Mycobacterium aromaticivorans	WP_036341979	--------A---SSL-AAV	W	----SA-L-
	Mycobacterium arosiense	WP_083063108	--------G---SS--A-V	W	---TSA-M-
	Mycobacterium arupense	WP_046190530	--------A---SGL-AAV	W	----SA-
	Mycobacterium asiaticum	WP_065034019	-----A--G---TS--AAI	W	---VSA-V-
	Mycobacterium avium	WP_003872601	--------G---SS--A-V	W	---TSA-L-
	Mycobacterium avium subsp. avium	EUA29706	--------G---SS--A-V	W	---T-A-M-
	Mycobacterium avium subsp. hominissuis	KDP11600	--------G---SS--A-V	W	---TSA-L-
	Mycobacterium boenickei	WP_077740649	I----A--A--VSS-TAAV	W	---VSA-V-
	Mycobacterium bohemicum	WP_085183379	--------G---SS--A-V	W	---SSA-M-
	Mycobacterium bovis	WP_047709705	--------G---SSL-A-V	W	---VSA-V-
	Mycobacterium branderi	WP_083133559	---V-A--G---SSL-AAI	W	---ISA-V-
	Mycobacterium brisbanense	WP_062830882	I----A--A--IGT--A-V	W	----SA-M-
	Mycobacterium canariasense	WP_062660100	VS-S-A--A--INS-VAAI	W	---VSA-V-
	Mycobacterium canettii	WP_014001948	--------G---SSL-A-V	W	---VSA-V-
	Mycobacterium celatum	WP_085168554	---V-A--G---SSL-AAI	W	---VSA-V-
	Mycobacterium chlorophenolicum	KMO70104	VS----A-A--VSS--V-S	W	---VSA-V-
	Mycobacterium chubuense	WP_014818122	-S----A-A--VSS--AAS	W	---A-A-I-
	Mycobacterium colombiense	WP_064880227	--------G---SS--A-V	W	---ASA-M-
	Mycobacterium conceptionense	WP_064894030	-----A--A--VSS-TAAV	W	---VSA-M-
	Mycobacterium conspicuum	WP_085233824	--------G--VTS--A-V	W	---SSA-V-
	Mycobacterium cosmeticum	WP_036397816	VS-S-A--A--VNSLVAAI	W	---VSA-V-
	Mycobacterium engbaekii	WP_085126445	-S------G---AGL-A-V	W	---VSA-L-
	Mycobacterium europaeum	WP_085242506	--------G---GS--V-V	W	---TSA-M-
	Mycobacterium fallax	WP_085095592	--------A--IS-L-AAV	W	---A-A-T-
	Mycobacterium farcinogenes	CDP87791	-----A--A--VSS-TAAV	W	---VSA-M-
	Mycobacterium florentinum	WP_085222029	--------G---SS--A-I	W	---TSA-M-
	Mycobacterium fortuitum	WP_061264247	-----A--A--VSS-TAAV	W	---VSA-V-
	Mycobacterium fragae	WP_085196747	--------G---SSL-A-V	W	---ASA-M-
	Mycobacterium gastri	WP_036411649	--------G---NSL-AAI	W	---VSA-V-
	Mycobacterium genavense	WP_025737783	--------G---SS--A-V	W	---TSA-M-
	Mycobacterium goodii	WP_049743615	--------A--VSS-TAAV	W	---ASA-I-
	Mycobacterium gordonae	WP_065046584	-----AA-G---NSL-VAI	W	---VSA-V-
	Mycobacterium haemophilum	WP_054879114	--------G--VS-L-A-V	W	---VSA-I-
	Mycobacterium heckeshornense	WP_048892026	--------A---SSL-AAI	W	---VSA-L-
	Mycobacterium heidelbergense	WP_083076151	--------G---NS--VAV	W	---SSA-T-
	Mycobacterium heraklionense	WP_064888650	--------G---AGL-A-V	W	---VSA-L-
	Mycobacterium hiberniae	WP_085136207	-------AG---AGL-A-V	W	---VSA-L-
	Mycobacterium icosiumassiliensis	WP_067977039	--------G---ASL-A-V	W	---VSA-L-
	Mycobacterium indicus pranii MTCC 9506	AFS12393	--------G---SS--A-V	W	---T-A-M-
	Mycobacterium insubricum	WP_083032491	--------A---S-L-AAV	W	---ASA-V-
	Mycobacterium interjectum	WP_066916957	--------G---TSL-A-V	W	---SSA-T-
	Mycobacterium intermedium	WP_069420302	--------A---SSL-AAT	W	---SSA---
	Mycobacterium intracellulare	WP_064893596	--------G---SS--A-V	W	---T-A-M-
	Mycobacterium kansasii	WP_063466798	-----A--G---NSL-AAI	W	---VSA-V-
	Mycobacterium kubicae	WP_085074913	-----A--G---NS--AAI	W	---VSA-V-
	Mycobacterium kumamotonense	WP_083083071	--------G---AGL-A-V	W	---ISA-L-
	Mycobacterium kyorinense	WP_065015763	--------G---SSL-AAI	W	---VSA-L-
	Mycobacterium lacus	WP_085162451	--------G---SSL-AAV	W	---VSA-V-
	Mycobacterium lentiflavum	CQD02177	--------G---NS--A-I	W	---TSA-M-
	Mycobacterium leprae	WP_010907537	--------G--VSSL-VAV	W	---VSA-T-
	Mycobacterium lepromatosis	WP_045842256	--------G--VTSL-AAV	W	---VSA-T-
	Mycobacterium liflandii	WP_015357611	--------G---NSL-AAI	W	---VSA-T-
	Mycobacterium litorale	WP_078021366	------A-A---S-L-AAV	W	----SA-L-

**Other *Mycobacterium* (0/>100)**	*Mycobacterium llatzerense*	WP_071286335	------A-A--VSSL-A-V W ---VSA-S-
	Mycobacterium longobardum	WP_085266298	--------A---AGL-A-V W ---VSA-L-
	Mycobacterium mageritense	WP_036431270	--------A--VSS-TAAV W ----SA-L-
	Mycobacterium malmoense	WP_065440893	-------SG---CS--A-V W ---TSA-M-
	Mycobacterium mantenii	WP_083097284	---V----G---SS--AAV W ---TSA-M-
	Mycobacterium marinum	WP_012396838	--------G---NSL-AAI W ---VSA-T-
	Mycobacterium marseillense	WP_083016731	--------G---NS--A-V W ---T-A-L-
	Mycobacterium minnesotense	WP_083025570	--------A---AGL-A-V W ---VSA-
	Mycobacterium mucogenicum	OBA75166	--------A---HSL-A-I W ---VSA-S-
	Mycobacterium nebraskense	WP_085165157	--------G---SS--A-V W ---TSA-M-
	Mycobacterium neworleansense	CRZ17581	-----A--A--VSS-TAAV W ---VSA-V-
	Mycobacterium nonchromogenicum	WP_085139970	--------A---SSL-A-I W ---VSA-L-
	Mycobacterium obuense	WP_046363495	VA----A-A--VFS--V-C W ---ASA-M-
	Mycobacterium palustre	WP_085079078	--D-----G---C-L-A-V W ---SSA-T-
	Mycobacterium paraense	WP_085095168	--------G--VTSL-A-V W ---SSA-T-
	Mycobacterium paraffinicum	WP_073874941	--------G---SS--A-V W ---TSA-M-
	Mycobacterium paraintracellulare	AFC51758	--------G---SS--A-V W ---T-A-M-
	Mycobacterium paraseoulense	WP_083171786	--------G---TS--AAV W ---TSA-M-
	Mycobacterium parmense	WP_085270848	--------G---NS--AAV W -Y-SSA-M-
	Mycobacterium peregrinum	WP_064885882	-----A--A--VSS-VAAV W ---VSA-V-
	Mycobacterium persicum	WP_083154696	-----A--G---NSL-AAI W ---VSA-V-
	Mycobacterium porcinum	WP_069427652	I----A--A--VSS-TAAV W ---VSA-V-
	Mycobacterium rhodesiae	WP_083121827	--------A---SSLSAAV W ----SAGL-
	Mycobacterium riyadhense	WP_085252424	--------G---SSL-VAV W ---VSA-V-
	Mycobacterium rufum	KGI70260	-S----A-A--VSS--V-S W ---VSA-V-
	Mycobacterium saskatchewanense	WP_085258345	--------G---TSL-A-V W ---SSA---
	Mycobacterium scrofulaceum	WP_067268664	--------G---SS--A-V W ---TSA-M-
	Mycobacterium senuense	WP_085085396	--------G---AGL-A-V W ---ISA-L-
	Mycobacterium septicum	WP_044513805	-----A--A--VSS-TAAV W ---VSA-V-
	Mycobacterium setense	WP_064876796	-----A--A--VSS-TAAV W ---VSA-V-
	Mycobacterium sherrisii	WP_069402657	--------A---N-----V W ---TSA-M-
	Mycobacterium shigaense	BAX94949	--------G--VSS--A-V W ---TSA-M-
	Mycobacterium shimoidei	WP_069397322	--------G---S-L-AAI W ---VSA-V-
	Mycobacterium shinjukuense	WP_083046717	--A-----G---TSL-AAV W ---VSA-V-
	Mycobacterium simiae	WP_061557585	--------A---NS--A-V W ---TSA-M-
	Mycobacterium sinense	WP_064853984	--------GC--AGL-A-V W ---VSA-L-
	Mycobacterium szulgai	WP_068034160	-----A--G---NS--AAI W ---VSA-V-
	Mycobacterium terrae	WP_085260001	--------GF--AGL-A-V W ---ISA-L-
	Mycobacterium triplex	WP_036465401	--------G---GS--A-I W ---TSA-M-
	Mycobacterium tuberculosis	WP_070890921	--------G---SSL-A-V W ---VSA-V-
	Mycobacterium ulcerans	WP_011738486	--------G---NSL-AAI W ---VSA-T-
	Mycobacterium vulneris	WP_065459598	I----A--A--VSS-TAAV W ---VSA-V-
	Mycobacterium wolinskyi	WP_067851494	--------A--VSS-TAAV W ---SSA-I-
	Mycobacterium xenopi	WP_085195649	--------A---SSL-AAI W ---VSA-L-
	Mycobacterium yongonense	ARR75922	--------G---SS--A-V W ---T-A-M-

Supplementary Figure 37

A partial sequence alignment of a conserved region of a hypothetical protein showing an one amino acid deletion that is specific for members of the *"Abscessus-Chelonae"* clade and absent in other *Mycobacterium*.

Group	Species	Accession	157		194
***"Abscessus-Chelonae"* Clade (6/6)**	*Mycobacterium abscessus*	WP_057138073	CGPETRYQPTVAMLEAL	AQAD	GRVPAGLIVASPANPTG
	Mycobacterium abscessus subsp. bolletii	EHM23222	----------------V	----	-----------------
	Mycobacterium chelonae	WP_070916692	---Q------------F	L-EE	--P--------------
	Mycobacterium franklinii	WP_070936922	---A------------V	S-S-	--A----V---------
	Mycobacterium immunogenum	WP_064632176	----------------V	S---	--A--------------
	Mycobacterium saopaulense	WP_070913175	--LQ------------V	L---	--A--------------
**Other *Mycobacterium* (0/>100)**	*Mycobacterium alsense*	WP_083139027	---Q--F---AQ--AE-		DPP-R-VV---------
	Mycobacterium angelicum	WP_083115127	---D--F---AQ--AE-		DPPVQ-V----------
	Mycobacterium aromaticivorans	WP_036341753	---Q--F----Q--AE-		DPPVQ-V----------
	Mycobacterium arosiense	WP_083064421	---Q--F---AQ--AE-		DPPVQ-V----------
	Mycobacterium arupense	WP_046190270	------F---A---AEI		EGPL--V----------
	Mycobacterium asiaticum	WP_065034316	------F---AQL-AE-		DPPIQ-VV---------
	Mycobacterium aurum	WP_087032674	-EAD--F----Q--D--		DPPVK---I--------
	Mycobacterium austroafricanum	WP_036375342	------F---LQ--TE-		DPPVS-V--------S-
	Mycobacterium avium	WP_065370973	---Q--F---A---AE-		DPPVQ-V----------
	Mycobacterium avium subsp. avium	ABZ81450	---Q--F---A---AE-		DPPVQ-V----------
	Mycobacterium avium subsp. hominissuis	ABZ81451	---Q--F---A---AE-		DPPVQ-V----------
	Mycobacterium avium subsp. paratuberculosis	ABZ81454	---Q--F---A---AE-		DPPVQ-V----------
	Mycobacterium avium subsp. silvaticum	AJT39812	---Q--F---A---AE-		DPPVQ-V----------
	Mycobacterium bacteremicum	WP_083058436	---Q--F---T------		DPPVR---I--------
	Mycobacterium boenickei	WP_077738787	------F---------I		DPPV--V----------
	Mycobacterium bohemicum	WP_085182759	---Q--F---AR--AE-		DPPVR-V----------
	Mycobacterium bovis BCG	AMC52808	---Q--F---AQ--AEI		DPPLR-VV---------
	Mycobacterium branderi	WP_083131103	---D--F---AQ--ADI		EPPVQ-VV---------
	Mycobacterium brisbanense	WP_062828503	------F---LS---E-		DPPV--V----------
	Mycobacterium canariasense	WP_062655928	------F----------		DPPVK---I--------
	Mycobacterium canettii	WP_014001809	---Q--F---AQ--AEI		DPPLH-VV---------
	Mycobacterium celatum	WP_062538903	------F---AQ--AE-		DPPV--VV---------
	Mycobacterium celeriflavum	ORA45847	------F----Q--A--		DPPVR-V----------
	Mycobacterium chlorophenolicum	WP_048468641	---D--F---L---TE-		DPPV--VV---------
	Mycobacterium chubuense	WP_014817833	---D--F---LQ--TE-		DPPVK-V----------
	Mycobacterium colombiense	WP_064882097	------F---AQ--AE-		D-PVQ-V----------
	Mycobacterium conceptionense	WP_064897666	------F----E-----		DPPV--V----------
	Mycobacterium conspicuum	WP_085234837	---Q--F---AQ--AE-		DPPVR-VV---------
	Mycobacterium cosmeticum	WP_036397627	------F----------		DPPAK---I--------
	Mycobacterium diernhoferi	WP_073856792	-EAD--F----Q-----		DPPVK---I--------
	Mycobacterium doricum	WP_085188596	--A---F---A---AEI		DPPVQ-V----------
	Mycobacterium elephantis	WP_083043479	------F---AQ--AE-		DPPVQ-V----------
	Mycobacterium engbaekii	WP_085128796	---A--F---A---SEI		PGPL--V----------
	Mycobacterium europaeum	WP_085239739	---Q--FH---Q--AE-		DPP-H-V----------
	Mycobacterium fallax	WP_085096807	---D--F-------DE-		DPR-D-V----------
	Mycobacterium farcinogenes	WP_036391626	------F----E-----		DPPV--V----------
	Mycobacterium flavescens	WP_069416558	--LQ--F----Q-----		DPPVQ-V----------
	Mycobacterium florentinum	WP_085223925	------F---TR--AE-		DPPVR-V----------
	Mycobacterium fortuitum	WP_061264751	------F----------		DPPV--V----------
	Mycobacterium fragae	WP_085198731	------F---AR--AE-		DPPVQ-VV---------
	Mycobacterium gastri	WP_036412493	---G--F----Q--AE-		DPPVQ--V---------
	Mycobacterium gilvum	WP_011892373	---D--F---LQ--TE-		DPPVS-V----------
	Mycobacterium goodii	WP_049748678	--------------D--		DPPVQ-V----------
	Mycobacterium gordonae	WP_065047504	------F---AQL-AE-		DPPVQ-V----------
	Mycobacterium haemophilum	WP_054879240	------F---AQ--DE-		DPPVQ-V----------
	Mycobacterium hassiacum	WP_051007475	---Q--F----Q--AE-		DPPVQ-V----------
	Mycobacterium heckeshornense	WP_048891202	---D--F---AH--A--		DPQ-R-VV---------
	Mycobacterium heidelbergense	WP_083073694	------F---AQL-AE-		DPPVQ-V----------
	Mycobacterium heraklionense	WP_064889273	------F---A---AEI		PGPL--V----------
	Mycobacterium hiberniae	WP_085134015	------F---AQ--AEI		PGPL--V----------
	Mycobacterium holsaticum	WP_069407662	---Q--F---AQ--DE-		DPPVQ-V----------
	Mycobacterium houstonense	WP_066900024	------F-------D--		DPRV--V----------
	Mycobacterium icosiumassiliensis	WP_067976421	------F---A---AEI		DGPL--V----------
	Mycobacterium indicus pranii	WP_014941230	---Q--F---AR--AE-		DPPVQ-V----------
	Mycobacterium interjectum	WP_066916432	------F---AR--AE-		DPPIK-V----------
	Mycobacterium intermedium	WP_069419481	------F---AQ--AEI		DPPVQ-V----------
	Mycobacterium intracellulare	WP_064935873	---Q--F---AR--AE-		DPPVQ-V----------
	Mycobacterium iranicum	WP_064279719	---Q--F---LD--TE-		DPPVS-V----------
	Mycobacterium kansasii	WP_063467891	---A--F-------AE-		DPPVQ--V---------
	Mycobacterium komanii	CRL75820	------F----Q--QS-		DPPVK-V----------
	Mycobacterium kubicae	WP_085075579	---Q--F---AQL-AE-		DPPVQ-V----------
	Mycobacterium kumamotonense	WP_065287591	------F---A-A-AEI		PGPL--V----------
	Mycobacterium kyorinense	WP_065012913	--AD--F---AQ--AEI		APPL--V----------
	Mycobacterium lacus	WP_085162390	------F---AR--AE-		DPPVR-V----------

**Other *Mycobacterium* (0/>100)**	*Mycobacterium lentiflavum*	CQD21597	------F---AQ--AE-	DPPVR-V----------
	Mycobacterium llatzerense	WP_071285698	------FH--IS---Q-	DPPVK---I---N----
	Mycobacterium mageritense	WP_085980462	------F----E-----	DPPV--V----------
	Mycobacterium malmesburyense	CRL69162	--S---F----Q---N-	DPPV--V----------
	Mycobacterium malmoense	WP_065480998	---D--F----Q--A-I	DPPIQ-V----------
	Mycobacterium mantenii	WP_083095434	---Q--F---AQ--AE-	DPPVQ-V----------
	Mycobacterium marinum	WP_085979927	------F---AD--AQI	DPPVQ-V----------
	Mycobacterium minnesotense	WP_083027503	------F---A---AEI	EGPL--V----------
	Mycobacterium moriokaense	WP_083156719	---Q--F----E--AEI	DPPVQ-VV---------
	Mycobacterium mucogenicum	WP_064859699	------FH--IS---Q-	DPPVK---I---N----
	Mycobacterium nebraskense	WP_046186395	---Q--F----Q--AE-	DPP-H-V----------
	Mycobacterium neworleansense	CRZ19084	------F-------D--	DPPV--V----------
	Mycobacterium nonchromogenicum	WP_085138922	------F---A---AEI	PGPL--V-I--------
	Mycobacterium noviomagense	WP_083085513	---D--F----D--A--	NPR-R-VV---------
	Mycobacterium novocastrense	WP_067393971	--AA--F----Q---E-	DPPLH-V----------
	Mycobacterium obuense	WP_046362815	---D--F---LD--T--	DPPI--V----------
	Mycobacterium palustre	WP_085076165	--A---F---AQ--AE-	DPPVR-V----------
	Mycobacterium paraense	WP_085095871	------F---AR--AE-	DPPIQ-V----------
	Mycobacterium paraffinicum	WP_073871312	---Q--F----Q---G-	DPP-H-V----------
	Mycobacterium parafortuitum	WP_083141971	------F---LQ--TE-	DPPV--V----------
	Mycobacterium paraintracellulare	WP_014383760	---Q--F---AR--AE-	DPPVQ-V----------
	Mycobacterium paraseoulense	WP_083175745	---Q--F----G--DE-	DPPVQ-V----------
	Mycobacterium parmense	WP_085268226	---A--F---AQL-AE-	DPPVQ-V----------
	Mycobacterium peregrinum	WP_064883769	------F----------	DPPV--V----------
	Mycobacterium persicum	WP_083153653	---A--F----Q--AE-	DPPVQ--V---------
	Mycobacterium phlei	WP_003888494	------F---AQ--AE-	DPPVQ-V----------
	Mycobacterium porcinum	WP_075920670	------F----------	DPPV--V----------
	Mycobacterium pseudoshottsii L15	GAQ34787	------F---AD--AQI	DPPVQ-V----------
	Mycobacterium rhodesiae	WP_083118775	------F----Q--AE-	DPPVQ-V----------
	Mycobacterium riyadhense	WP_085251160	------F----R--AE-	DPPVQ-V----------
	Mycobacterium rufum	KGI70006	--AD--F----D--T--	DPPV--V----------
	Mycobacterium rutilum	SEH61565	------F----Q-----	DPPVQ-V----------
	Mycobacterium saskatchewanense	WP_085255331	---Q--F---AR--AE-	DPPVR-V----------
	Mycobacterium scrofulaceum	WP_067279090	---Q--F----Q---E-	DPP-R-V----------
	Mycobacterium septicum	WP_044520754	------F----------	DPPV--V----------
	Mycobacterium setense	WP_064876443	------F-------D--	DPPV--V----------
	Mycobacterium sherrisii	WP_069402346	--EQ--F---AQ--AE-	DPPVR-VV---------
	Mycobacterium shigaense	BAX94666	---N--F---AQ--AE-	DPPVR-V----------
	Mycobacterium shimoidei	WP_069394775	---Q--F---A---AQ-	DPPVQ-VV---------
	Mycobacterium shinjukuense	WP_083047251	---Q--F----Q--AEI	HPPLH-V----------
	Mycobacterium simiae	WP_044508664	--EQ--F---AQL-AE-	DQPVR-VV---------
	Mycobacterium sinense	WP_064856152	------F---A-A-AEI	PGPL--V----------
	Mycobacterium smegmatis	WP_080628215	------F---A------	DPPVQ-V----------
	Mycobacterium szulgai	WP_068023273	---Q--F---AQL-AE-	DPPVQ-V----------
	Mycobacterium terrae	WP_085261462	---A--F---A-A-AEI	PGPL--V----------
	Mycobacterium thermoresistibile	WP_003925036	---Q--F---AR--AE-	DPPVR-V----------
	Mycobacterium triviale	WP_085110560	---D------A---A--	DPPV--V----------
	Mycobacterium tuberculosis	WP_070889906	---Q--F---AQ--AEI	DPPLR-VV-------A-
	Mycobacterium tusciae	WP_083127293	---Q--F---TQ--AE-	DPPVQ-V----------
	Mycobacterium ulcerans	WP_011741775	------F---AD--AQI	DPPVQ-V----------
	Mycobacterium vaccae	WP_003930627	------F---LQ--Q--	DPPVS-V----------
	Mycobacterium vanbaalenii	WP_011782431	---K--F---LQ--TE-	DPPVS-V--------S-
	Mycobacterium vulneris	WP_065460848	------F----------	DPPV--V----------
	Mycobacterium wolinskyi	WP_067858597	------F----T---E-	DPPVQ-V----------
	Mycobacterium xenopi	WP_050947746	---D--F---AQL-A--	DPP-R-VV---------

Supplementary Figure 38

A partial sequence alignment of a conserved region of pyridoxal phosphate-dependent aminotransferase showing a four amino acid insertion that is specific for members of the "*Abscessus-Chelonae"* clade and absent in other *Mycobacterium.*

			221		259
**"Abscessus-Chelonae" Clade (7/7)**	*Mycobacterium abscessus*	WP_062880095	VVFYDLPVTLDMSMISQ	S	VPVPRLLRKPAQIVMNSLIGK
	Mycobacterium abscessus subsp. bolletii	EUA68227	-----------------	-	---------------------
	Mycobacterium chelonae	WP_070915422	-----------TA--TK	-	--M-PI------L--Q-----
	Mycobacterium franklinii	WP_078333161	-----------------	-	---------------------
	Mycobacterium immunogenum	WP_064628257	--L--------TN--TK	-	--M-PI------L--Q-----
	Mycobacterium salmoniphilum	WP_078328001	-----------T---TK	-	--M-AI------L--Q-----
	Mycobacterium saopaulense	WP_070909350	-----------------	-	---------------------
**Other *Mycobacterium* (0/86)**	*Mycobacterium acapulcensis*	WP_066808113	---------F-AG-AVE		MT---G--FVSRL-LSA---R
	Mycobacterium africanum	WP_061846034	--I------F-PMQVVP		AS---W-QR--RL-IQ-VL-R
	Mycobacterium algericum	WP_083037202	--V------F-PIQVLP		MNL--W-SA--RL-L---V--
	Mycobacterium alsense	WP_083136044	--I------F-PVQVMP		AN---W-DL--RL--Q--V-R
	Mycobacterium angelicum	WP_083112868	--I------F-PVQLLP		-Q---W-GLAG-L--R--V-R
	Mycobacterium arosiense	WP_083063439	--L------F-AVQVMP		TN---R-SL--RL--Q----R
	Mycobacterium arupense	WP_046191309	---------F-TRRVAE		GAA--G--L-VRLI-SAV--R
	Mycobacterium asiaticum	WP_065142055	--I------F-PGQVMP		-N---R-SA--RL-LG-IL-R
	Mycobacterium aurum	WP_048635334	---------F-PRRAVA		MS---A-QL--RLLLSA---R
	Mycobacterium austroafricanum	WP_036370813	---------F-SRQAAT		IG---W-QL--RL-LSA---R
	Mycobacterium avium	WP_062908008	---------F-AAVGAA		MTA--G--PI-RLMLSA---R
	Mycobacterium avium subsp. avium	EUA37264	--I------F-PVQVMP		AD---W-SA--RL-VQ--L-R
	Mycobacterium avium subsp. hominissuis	BAN29558	---------F-AAVGAA		MTA--G--PI-RLMLSA---R
	Mycobacterium avium subsp. paratuberculosis	AJK77215	--I------F-PVQVMP		AD---W-SA--RL-VQ--L-R
	Mycobacterium bohemicum	WP_085179232	--I------FEAAQVLP		ANL--W-DL--RL-AQ----R
	Mycobacterium bovis	WP_023349454	--I------F-PMQVVP		AS---W-QR--RL-IQ-VL-R
	Mycobacterium canettii	WP_014000339	--I------F-PMQVVP		AS---W--R--RL-IQ-VL-R
	Mycobacterium celeriflavum	WP_083004131	---------F-AR-AVE		MT---G--LVSRL-LSA---R
	Mycobacterium chlorophenolicum	WP_048473594	-IL------F-VHQAVE		MT---A--L--RLMLSA---R
	Mycobacterium chubuense	WP_048416880	-IL------F-VHQAVE		MT---G--L--RLMLSA---R
	Mycobacterium colombiense	WP_064950202	--I------F-PVQVMP		AN---W-GS--RL--Q----R
	Mycobacterium confluentis	WP_085149730	--I------F-PGEVVP		-T--KW--A--RM-VSA-A-R
	Mycobacterium engbaekii	WP_085127761	--V------F-SADVLP		-RM--W-KA--RM-LS-----
	Mycobacterium europaeum	CQD10977	--I------F-PGQVLP		-TM--W-SM--RL--Q----R
	Mycobacterium florentinum	WP_085222911	--I------F-PAQVVP		AN---W-SL--RL--E-VL-R
	Mycobacterium fragae	WP_085197319	--L------F-PMQALP		TN---W-SL--RL--Q----R
	Mycobacterium gastri	WP_085104927	--I------F-A-QVLP		AKL--W-SL---L--Q-V--H
	Mycobacterium gilvum	WP_011892999	-I-------F-TRRAVE		MT---G--L--RL-LSA---R
	Mycobacterium gordonae	WP_065044466	--I------F-PGQVLP		-KI--WMSV---L--Q--V-R
	Mycobacterium haemophilum	WP_047313550	--I------F-PVPVLR		AA--SW-QL--RL--Q-V--R
	Mycobacterium hassiacum	WP_005623941	--I------A-VRQMAE		MA---P--L--RL-LSA---R
	Mycobacterium heckeshornense	WP_048890373	--I------F-PVQVLP		-N---W-GL--RL-LQ----R
	Mycobacterium heidelbergense	WP_083073978	--I------F-PVQALP		AN---W-SL--RL--Q----R
	Mycobacterium heraklionense	WP_064888172	--V------F-SAQVLP		-SM--W--A--RM-LS--V-R
	Mycobacterium hiberniae	WP_085135069	--V------F-SADVLP		-RM--W-KA--RM-LS-----
	Mycobacterium icosiumassiliens	WP_067968326	---------F-TRQVA-		GAA-PG--L-VRLI-SA---R
	Mycobacterium interjectum	WP_066907245	--I------F-AAQALP		-TM--W-SA--RL--Q--V-R
	Mycobacterium intermedium	WP_069420831	--I------F-PIQ--P		-K---W-NA--RLALQ-VL-R
	Mycobacterium intracellulare	WP_064938881	--I------F-PVQVMP		TN---W-GL--RL--Q----R
	Mycobacterium kansasii	WP_063468622	--V------F-S-QVLP		AKM--W-SL---L--QA---H
	Mycobacterium komanii	CRL68516	---------F-AE-AV-		MT---G--LVSRL-LSA---R
	Mycobacterium koreense	WP_085304698	---------FNAQQVVE		AT---A--W--RL--SA---R
	Mycobacterium kumamotonense	WP_065288180	--V------F-SAQVLP		-SM--W-KA--RM-LS--V-R
	Mycobacterium lacus	WP_085157515	--I-----RF-SRQVQL		-K---W-SLA-RLT-Q----R
	Mycobacterium lentiflavum	CQD06329	--I------F-PMQ-LP		AS---W-SS--RL-LQ--L-R
	Mycobacterium liflandii	WP_041298833	--I------F-SAQVMP		-KM-SW-GV--RL--A----N
	Mycobacterium llatzerense	WP_043986440	-I-------F-ARIVAE		MA--KA--V--RLLLS----R
	Mycobacterium longobardum	WP_085265745	---------F-ARQVAE		GAA-PG--L-VRLILSA---R
	Mycobacterium malmesburyense	CRL69277	---------F-AG-AV-		MT---G--FVSRL-LSAV--R
	Mycobacterium malmoense	WP_065473331	--I------F-PAQVLP		-TA--W-GL--RLALQA---R
	Mycobacterium marinum	WP_012392904	--I------F-SAQVMP		-KM-SW-GV--RL--A----N
	Mycobacterium marseillense	WP_083018529	--I------F-AVQVMP		TT---W-SL--RL--Q----R
	Mycobacterium minnesotense	WP_083022543	---------F-TRRVAA		GAA--G--L-VRLI-SAV--R
	Mycobacterium mungi	WP_064319949	--I------F-PMQVVP		AS---W-QR--RL-IQ-VL-R
	Mycobacterium nebraskense	WP_046186729	--I------F-PMQVLP		-TM--W-GL--RLALQ----R
	Mycobacterium nonchromogenicum	WP_085139974	---------F-SRQVA-		GAA-PG--L-VRLI-SA---R
	Mycobacterium noviomagense	ORB13478	--I------F-PVQALP		AN---W-GL--RL-LQ----R

Other *Mycobacterium* (0/86)	*Mycobacterium novocastrense*	WP_067386389	---------F-TG-AV-	MT---G--SVSRL-LSA---R
	Mycobacterium obuense	WP_046365774	---------F-VEQAVE	MT--KA--L--RLM-SA-L-R
	Mycobacterium palustre	WP_085081083	--L------F-PVQVVP	SS--GW-GA--RL-LQ----R
	Mycobacterium paraense	WP_085097143	--I------F-AVQALP	ANM--W-SA--RLA-Q--V-R
	Mycobacterium parafortuitum	WP_083145491	--I------F-VRRAVE	TS---G--M--RL-LSA-L-R
	Mycobacterium paraseoulense	WP_083171605	--V------F-PMQVLP	-SI--W-GL--RLA-Q----R
	Mycobacterium parmense	WP_085271907	--I------F-PMQALP	TRM-PWS-L--RLA-Q----R
	Mycobacterium pseudoshottsii	WP_086085066	--I------F-SAQVM-	-KM-SW-GV--RL--A----N
	Mycobacterium riyadhense	WP_085249147	--I------F-PVQVLP	-KM--W-GL--RL--Q----R
	Mycobacterium rufum	KGI69460	-IL------F-VAQAVE	MT---A--L--RLMLSA---R
	Mycobacterium rutilum	WP_083407905	-----------ANQMV-	MG--KA--L--RLMLSA---R
	Mycobacterium saskatchewanense	WP_085258110	--I------F-PAQVLP	-KT--W-GL--RL--Q----R
	Mycobacterium scrofulaceum	WP_067274946	--I------F-PAQALP	QAM--W-GL--RLA-Q----R
	Mycobacterium senuense	WP_085088439	--V------F-PIQVLP	-NL--R-QA--RL-LG--L--
	Mycobacterium sherrisii	WP_069399565	--V------F-PIQVAP	AN---W-SA--RL-VE--L-R
	Mycobacterium shigaense	BAX91012	--I------F-AARVAS	AT---W-DL--RL-TQ--L-R
	Mycobacterium shinjukuense	WP_083045963	--I------F-PAQVLP	ATM--W--L---L--Q----R
	Mycobacterium sinense	WP_064855563	---------F-TRQVAE	GSA-PG--L-VRLIL-A---R
	Mycobacterium szulgai	WP_085670010	--I------F-PVQLLP	-K---W-GLAG-LA-Q--V-R
	Mycobacterium terrae	ORW98335	--V------F-SAQVLP	-NM--W-KT--RL-LS--V--
	Mycobacterium thermoresistibile	WP_003926387	I-L------F-ARQAVS	MT---G--L--RL-LSA---R
	Mycobacterium triviale	WP_069390685	---------FNAQQVVE	AT---A--W--RL--SA---R
	Mycobacterium tuberculosis	WP_070892335	--I------F-PMQVVP	AS---W-QR--RL-IQ-VL-R
	Mycobacterium ulcerans subsp. shinshuense	BAV43031	--I------F-SAQVMP	-KM-SW-GV--RL--A----N
	Mycobacterium vaccae	WP_003931132	---------F-ARRAAA	-G---W-QL--RL-LSA---R
	Mycobacterium vanbaalenii	WP_011781747	---------F-SRQAAT	IG---W-QL--RL-LSA---R
	Mycobacterium vulneris	WP_085291267	--I------F-PVQVMP	TN---W-GL--RL--Q----R
	Mycobacterium xenopi	ORX10463	--V------F-PVQVLP	-K---W-GL--RL-LQ----R
	Mycobacterium yongonense	WP_065500726	--I------F-PVQVMP	AN---W-SL--RL--Q----R

Supplementary Figure 39

A partial sequence alignment of a conserved region of carotenoid oxygenase showing a one amino acid insertion that is specific for members of the "*Abscessus-Chelonae"* clade and absent in other *Mycobacterium*.

			139		168
***"Abscessus-Chelonae"* Clade (7/7)**	*Mycobacterium abscessus*	WP_062879314	GEATVDDTLLFDGTAL		VDVEPTESMPGLRA
	Mycobacterium abscessus subsp. bolletii	EHM17548	----------------		--------------
	Mycobacterium chelonae	WP_070915725	-----------E-I-A		--I--I-AL-----
	Mycobacterium franklinii	WP_070938178	----------------		--------------
	Mycobacterium immunogenum	WP_064627322	-------------A--		--------------
	Mycobacterium salmoniphilum	WP_078324014	-----------E-I-A		-----I--L-----
	Mycobacterium saopaulense	WP_070909023	-------------A--		--------V-----
**Other *Mycobacterium* (0/>100)**	*Mycobacterium angelicum*	WP_083114281	---V----V----DVT	G	-SI---AAL-----
	Mycobacterium aromaticivorans	WP_036346871	---V---QV----EVT	A	-R---MAV------
	Mycobacterium arosiense	WP_083064259	---V----T----DVA	G	-RI---PA------
	Mycobacterium asiaticum	WP_065035883	---V----V----DVT	A	IWI---LAL-----
	Mycobacterium aurum	WP_087033019	---V---EV----E-T	A	-RI--IGT------
	Mycobacterium austroafricanum	WP_036369079	---V---EP----EVT	G	-RI---AA------
	Mycobacterium avium	WP_065370866	---V----T----DVT	A	-WI---AA------
	Mycobacterium avium subsp. avium	EUA37430	---V----T----DVT	A	-WI---AA------
	Mycobacterium avium subsp. paratuberculosis	EGO39293	---V----T----DVT	A	-WI---AA------
	Mycobacterium bacteremicum	WP_083062108	---V----V----EVT	A	-R---IPDQ-----
	Mycobacterium boenickei	WP_077740213	---I---IV----E-P	G	-R----L-------
	Mycobacterium bohemicum	WP_085179461	---V----V----DVG	A	-Y----PVP-----
	Mycobacterium bovis	WP_019283982	---V----V----DVA	G	-CI---LTL-----
	Mycobacterium branderi	WP_083133889	---V----T----DVT	A	-RI---AAL-----
	Mycobacterium brisbanense	WP_062829580	---V---H-----DVP	G	-R----L-------
	Mycobacterium canariasense	WP_062658655	---V----V------T	A	-RI--IAAQ-----
	Mycobacterium canettii	WP_015288980	---V----V----DVA	G	-CI---VTL-----
	Mycobacterium celatum	WP_085168485	---V----V----DVA	A	-RL---AAA-----
	Mycobacterium chlorophenolicum	KMO76623	---V----V----EVP	A	-RI---AQL-----
	Mycobacterium chubuense	KMO79404	---V----V----QVP	A	-RI---AQL-----
	Mycobacterium colombiense	WP_064880803	---V----T----DVE	R	-WI---P-A-----
	Mycobacterium conceptionense	WP_064895968	---I---VV----Q-P	G	-R----LV------
	Mycobacterium confluentis	WP_085151647	---I----V----H-P	A	LR---INA------
	Mycobacterium conspicuum	WP_085236320	---V---SV----DVS	G	-RI---L-L-----
	Mycobacterium cosmeticum	WP_036401019	---V----V------T	A	-RI--IATQ-----
	Mycobacterium doricum	WP_085189509	---V----T--S-DVA	G	-RI---AE------
	Mycobacterium elephantis	WP_046751725	---V----V----DVP	G	-R----LL------
	Mycobacterium europaeum	WP_085240708	---V----T----D-P	A	--I---LAL-----
	Mycobacterium fallax	WP_085095987	---V----V----H-P	G	LR-Q-LDRV-----
	Mycobacterium farcinogenes	WP_036389461	---I---VV----E-P	G	-R----LV------
	Mycobacterium flavescens	WP_069412528	---V---EV-----VT	G	-RI---STL-----
	Mycobacterium fortuitum	WP_064901017	---I---VV----E-C	G	-R----T-------
	Mycobacterium fragae	WP_085197872	---I----V--N-DVD	G	-RI---AAL-----
	Mycobacterium gastri	WP_036410514	---V----V----EVA	G	-CI---LQL-----
	Mycobacterium genavense	WP_025736939	---V----Q----EVT	G	-YI---LAV-----
	Mycobacterium gilvum	WP_011891231	---I---ER----DVT	G	-RI---TA------
	Mycobacterium goodii	WP_049748446	---I----V----A-H	G	-R---LAV------
	Mycobacterium gordonae	WP_065045139	---V----V----D-A	G	-WI---PAL-----
	Mycobacterium haemophilum	WP_054878838	---V---IP----DVA	G	-RIA--LA------
	Mycobacterium hassiacum	WP_005624296	-------------RVT	G	-RI---DTL-----
	Mycobacterium heckeshornense	WP_048889652	---V----T----DVA	G	-R----AVL-----
	Mycobacterium heidelbergense	WP_0830741	---V----A----DVA	A	-F----LA----
	Mycobacterium holsaticum	WP_069405467	---V----V----ETP	G	-R----SAL-----
	Mycobacterium houstonense	WP_066900033	---I---HV----E-P	G	-R----PD------
	Mycobacterium indicus pranii	WP_043955442	---V----M----D-A	G	-L----LA------
	Mycobacterium interjectum	WP_085200388	---V----T----EVA	A	-RI---LG------
	Mycobacterium intermedium	WP_06942102	---V----V----D-A	G	A-I---L-L----
	Mycobacterium intracellulare	WP_064935214	---V----T----D-A	G	-F----LA------
	Mycobacterium iranicum	WP_036464602	---I---ER----E-T	A	-RI--IAT------
	Mycobacterium kansasii	WP_063467548	---V----V----DVA	G	-FI---PEL-----
	Mycobacterium komanii	CRL69171	---V---EV----AVT	G	-RI---TA------
	Mycobacterium kubicae	WP_085072710	---V----S----DVS	G	-RI---PAV-----
	Mycobacterium kyorinense	WP_045374110	---V----T----DVA	A	-RI---AAL-----
	Mycobacterium lacus	WP_085160050	---V----V----DVA	G	-CI---AT------
	Mycobacterium lentiflavum	CQD04923	---V----Q----NVT	G	A-I---LAV-----
	Mycobacterium lepromatosis	WP_045842380	---V--NVR----DVA	G	-HIA--LA------
	Mycobacterium litorale	WP_078021749	---V---EV----EVT	G	-RI---RT------

Group	Species	Accession	Sequence
Other *Mycobacterium* (0/>100)	*Mycobacterium llatzerense*	WP_082067831	---V----V----EST G -VI--LPG------
	Mycobacterium mageritense	WP_036433518	---V----V----EVP G -RI---S-L-----
	Mycobacterium malmesburyense	CRL70280	---V---EV----AVT G -RI---PT------
	Mycobacterium malmoense	WP_065477807	---V----T----D-P G --I---PAV-----
	Mycobacterium marseillense	WP_083019126	---V----T----D-A A -FI---LA------
	Mycobacterium moriokaense	WP_083156866	---V----V--H-E-T G -LI---P-------
	Mycobacterium mucogenicum	OBA87916	---V----V-----ST G -VI--LPG------
	Mycobacterium nebraskense	WP_047322824	---V----T----H-P A --I---LAV-----
	Mycobacterium neoaurum	CDQ43333	---V----V----EVA G -R---IADQ-----
	Mycobacterium neworleansense	CRZ16561	---I---VV----E-P G -R----LN------
	Mycobacterium novocastrense	WP_067392131	---V---EV----A-T G -RI---PT------
	Mycobacterium obuense	WP_046364756	---V----V----EVT G -R----RQL-----
	Mycobacterium palustre	WP_085078819	---V----P----EVE Q -W----LA------
	Mycobacterium paraense	WP_085093034	---V----T----EVA A LR----LDL-----
	Mycobacterium paraffinicum	WP_073874462	---V---AV----A-P A ------L-V-----
	Mycobacterium parafortuitum	WP_083142271	---V---EW----EIT G -RI---A-------
	Mycobacterium paraseoulense	WP_083173326	---V---AV----A-R A -HI---LAV-----
	Mycobacterium parmense	WP_085271693	---V----V----EVA A -L----LAP-----
	Mycobacterium peregrinum	WP_064881689	---I---IV----E-P S -R----T-------
	Mycobacterium phlei	WP_040633915	---V---EV----EVT G -RI---GT------
	Mycobacterium porcinum	WP_075922445	---I---IV----E-P G -R----L-------
	Mycobacterium rhodesiae	WP_083118364	---V---QV----EVT A -R---ITA------
	Mycobacterium riyadhense	WP_085251385	---V----V----EVA G -CI---LAL-----
	Mycobacterium rufum	KGI66549	--GV----V----EVP A -R----GTP-----
	Mycobacterium rutilum	WP_083405990	---V----V----EVP G -RI---TA------
	Mycobacterium saskatchewanense	WP_085257863	---V---AV----DVA A -SI---WA------
	Mycobacterium scrofulaceum	WP_067268729	---V---AV----DVP G -H----LAV-----
	Mycobacterium septicum	WP_044515413	---V---VV----E-P G -R----LN------
	Mycobacterium setense	WP_039313632	---I---CV----E-P G -R----LA------
	Mycobacterium sherrisii	WP_069401267	---VA---V----DVT G A-----L-------
	Mycobacterium shigaense	BAX90747	---V----V----D-A G -RI---LAV----
	Mycobacterium shimoidei	WP_069395959	---V----V----DVA A -R----AAL-----
	Mycobacterium shinjukuense	WP_083050930	---V----V----DVA G -C----LAL-----
	Mycobacterium simiae	WP_061558132	---V----V----DVT G A-----LA------
	Mycobacterium smegmatis	WP_003892183	---I----V----Q-H G -RI--LDAV-----
	Mycobacterium szulgai	WP_085672514	---V----V----DVT G -SI---AAL-----
	Mycobacterium triplex	WP_036466366	---V----Q----DVP G --I---LAV-----
	Mycobacterium triviale	WP_085109033	---V----V----RVR G -R---LPEL-----
	Mycobacterium tuberculosis	WP_070891367	---V----V----DVA G -CI---LTL-----
	Mycobacterium tusciae	WP_051469015	-------IA--G-EVT G -RI----T----K-
	Mycobacterium ulcerans str. Harvey	EUA92151	---V---NT----DIA G -YIQ--LEL-----
	Mycobacterium vanbaalenii	WP_011777951	---V---ER----EVT G -R----AA------
	Mycobacterium vulneris	WP_065512618	---I---IV----V-P G -RI---L-------
	Mycobacterium wolinskyi	WP_084356833	---V---AV----D-P G -RI---L-Q-----
	Mycobacterium xenopi	WP_085193261	---V----T----DVA G -R----AAL-----
	Mycobacterium yongonense	WP_065500848	---V----T----D-A G -L----LAV-----
Other *Corynebacteriales*	*Gordonia desulfuricans*	WP_059037965	--SY---HR--S--VS G IRI---REL-----
	Gordonia lacunae	WP_086533913	--TY--NDR----EVR S ILI---LTA-----
	Gordonia soli	WP_051989690	--TI---QR--L-E-A G -EI---GEL-----
	Nocardia abscessus	WP_043699504	---Y--E----S-KVT A ML-S-SLEP--V--
	Nocardia concava	WP_040813940	---Y----R--T-KVA A LH-S-MLE----H-
	Nocardia ignorata	WP_067484363	---Y-------T--VT T MLIS--MA---V--
	Nocardia inohanensis	WP_067830472	---Y----R--T-K-G A LH-S--LR----H-
	Nocardia transvalensis	WP_040751384	---Y----R--T-KVG A LH-S--LE---V--
	Rhodococcus coprophilus	WP_072702854	---Y----R--S--VP G IRI---PA------
	Rhodococcus defluvii	WP_081880676	---Y----R--S--VP G MLIV--PEH-----
	Rhodococcus rhodochrous	WP_080968426	---Y----R--S--VP G IR----PA------
	Rhodococcus triatomae	SDI91510	---Y----R--S-V-P R LN-A-VPAA-----

Supplementary Figure 40

A partial sequence alignment of a conserved region of a hypothetical protein showing a one amino acid deletion that is specific for members of the "*Abscessus-Chelonae"* clade and absent in other *Corynebacteriales*.

Group	Species	Accession	340		367
**"Abscessus-Chelonae" Clade (5/5)**	*Mycobacterium abscessus*	WP_062879407	GSPKGNCDTTYTLCDAA	GGV	GRALPSWL
	Mycobacterium abscessus subsp. bolletii	EUA66722	-----------------	---	--------
	Mycobacterium chelonae	WP_070948438	-----------------	---	--------
	Mycobacterium salmoniphilum	WP_078325861	-----------------	---	--------
	Mycobacterium saopaulense	WP_088415073	--------SA-------	---	--------
**Other *Mycobacterium* (0/>100)**	*Mycobacterium africanum*	WP_031668662	-----S---RQV---SH		E-------
	Mycobacterium alsense	WP_083140675	---R-S---RQV---SH		E-------
	Mycobacterium aromaticivorans	KDF00094	-V---S---MAV---SR		E----A--
	Mycobacterium arosiense	WP_083066234	---R-S---RQV---SH		E-------
	Mycobacterium arupense	WP_046189185	-V-R-T---AQV----H		E-------
	Mycobacterium asiaticum	WP_065034636	-----S---RQV---SR		E-------
	Mycobacterium aurum	WP_087029189	-V---S---YQV---SR		E-------
	Mycobacterium austroafricanum	WP_051558294	---R-S---MAV---SR		E--Q-A--
	Mycobacterium avium	WP_062894682	---R-S---RQV---SH		E-------
	Mycobacterium avium subsp. avium	EUA29154	---R-S---RQV---SH		E-------
	Mycobacterium avium subsp. hominissuis	KDO92739	---R-S---RQV---SH		E-------
	Mycobacterium avium subsp. paratuberculosis	ETB14937	---R-S---RQV---SH		E-S-----
	Mycobacterium bacteremicum	WP_083060996	-V---S---YQV---SK		E-------
	Mycobacterium boenickei	WP_077739818	---R-----RQV---SR		E-------
	Mycobacterium bohemicum	WP_085179527	---R-S---RQV---SH		E-------
	Mycobacterium bovis BCG	AKO23128	-----S---RQV---SH		E-------
	Mycobacterium branderi	WP_083133186	-V---S---RQV---SH		E-------
	Mycobacterium brisbanense	GAS86024	---R-----RAV---SR		E-------
	Mycobacterium canariasense	WP_084395572	-V---S---YQV---SR		E----A--
	Mycobacterium canettii	WP_014000095	-----S---RQV---SH		E-------
	Mycobacterium celatum	WP_062539054	-V---S---RQV---SH		E-------
	Mycobacterium celeriflavum	ORA51146	-V-R-S---RQV---SR		E-------
	Mycobacterium chimaera	WP_087139788	---R-S---RQV---SH		E-------
	Mycobacterium chlorophenolicum	WP_082169109	---Q-S---MAV---SH		E--Q-A--
	Mycobacterium chubuense	WP_048420544	---Q-S---MAV---SH		E--Q-A--
	Mycobacterium colombiense	WP_064877620	---R-S---RQV---SH		E-------
	Mycobacterium conceptionense	OBB03805	---R-----RQV---SR		E-------
	Mycobacterium confluentis	WP_085157327	-V---S---FQV---SR		E-------
	Mycobacterium conspicuum	WP_085232005	---R-S---REV---SH		E-G-----
	Mycobacterium cosmeticum	CDO08452	-V---S---YQV---SR		E----A--
	Mycobacterium diernhoferi	WP_073855365	-V---S---FEV---SR		E-------
	Mycobacterium elephantis	WP_083042733	-V-R-S---RQV---SR		E-------
	Mycobacterium europaeum	WP_085243597	---R-S---RQV---SH		E-------
	Mycobacterium fallax	ORV00015	-V---S---YQV---SR		E-------
	Mycobacterium farcinogenes	WP_036392478	---R-----RQV---SR		E-------
	Mycobacterium flavescens	ODQ88503	-V-R-S---RQV---SR		E-------
	Mycobacterium florentinum	WP_085222440	---R-S---RQV---SH		E-------
	Mycobacterium fortuitum	WP_064850407	---R-----RQV---SR		E-------
	Mycobacterium fragae	WP_085199398	-V---S---AQV---SR		E-------
	Mycobacterium gastri	WP_036412128	-----S---REV---SH		E-------
	Mycobacterium genavense	WP_036468854	---R-S---RQV---SH		E-------
	Mycobacterium goodii	WP_049748594	---R-----RQV---SR		E-------
	Mycobacterium gordonae	WP_065048361	-A---S---RQV---SH		E-------
	Mycobacterium haemophilum	WP_054878938	-----S---RQV---SH		E-------
	Mycobacterium hassiacum	WP_005631910	---R-S---WQV---SR		E-------
	Mycobacterium heckeshornense	WP_048890434	---R-----RQV---SH		E-------
	Mycobacterium heidelbergense	WP_083075822	---R-S---REV---SR		E-------
	Mycobacterium heraklionense	OBK87030	-V-R-S---AQV----H		E-G-----
	Mycobacterium houstonense	WP_066900940	---R-----RQV---SR		E-------
	Mycobacterium icosiumassiliensis	WP_067968930	-V-R-S---AQV----H		E-G-----
	Mycobacterium insubricum	WP_083033027	-V---S---VQV---SR		E-------
	Mycobacterium interjectum	WP_066917379	---R-S---RQV---SH		E-------
	Mycobacterium intermedium	WP_069421434	-----S---RQV---SH		E-------
	Mycobacterium intracellulare	WP_064938329	---R-S---RQV---SH		E-------
	Mycobacterium iranicum	WP_064283459	---R-S---MAV---SR		E--Q-A--
	Mycobacterium kansasii	WP_063470609	-----S---REV---SH		E-------
	Mycobacterium koreense	WP_085303990	-V---S---REV---SK		E-------
	Mycobacterium kyorinense	OBI45981	-V---S---RQV---SR		E-------
	Mycobacterium lacus	WP_085162769	-----S---RQV---SH		E-------

Other *Mycobacterium* (0/>100)

Mycobacterium lentiflavum	CQD03965	---R-S---RQV---SH	E-------
Mycobacterium litorale	WP_078017359	-V---S---MQV---SR	E-------
Mycobacterium llatzerense	WP_083420546	-T-R-S---PAV---SR	E----G--
Mycobacterium longobardum	WP_085267060	-V-R-S---AQV----H	E-------
Mycobacterium mageritense	WP_036435463	---R-S---RQV---SR	E-------
Mycobacterium malmoense	WP_065472960	---R-S---RQV---SH	E-------
Mycobacterium mantenii	WP_083097607	---R-S---RQV---SH	E-------
Mycobacterium marinum	WP_020727828	-A---S---RQV---SH	E-------
Mycobacterium minnesotense	WP_083027952	-V-R-T---AQV----H	E-G-----
Mycobacterium monacense	WP_083044509	---R-S---RQV---SR	E-------
Mycobacterium moriokaense	WP_083153073	-V-D-----RQV---SR	E-------
Mycobacterium mucogenicum	WP_082371126	-T-R-S---PAV---SR	E----G--
Mycobacterium nebraskense	WP_047322374	---R-S---QQV---SH	E-------
Mycobacterium neoaurum	WP_030133981	-V---S---QQV---SK	E-------
Mycobacterium neworleansense	CRZ17073	---R-----RQV---SR	E-------
Mycobacterium noviomagense	WP_083089075	-A-------RQV---SH	E-------
Mycobacterium obuense	WP_048424923	---Q-S---MAV---SH	E--Q-A--
Mycobacterium palustre	WP_085076335	---R-S---RQV---SH	E-------
Mycobacterium paraense	WP_085098551	---R-S---RQV---SH	E-------
Mycobacterium paraffinicum	WP_073877819	---R-S---RQV---SH	E-------
Mycobacterium parafortuitum	WP_083144354	---R-S---MAV---SR	E--Q-A--
Mycobacterium paraseoulense	WP_083175730	---R-S---RQV---SH	E-------
Mycobacterium parmense	WP_085270353	---R-S---RQV---SH	E-------
Mycobacterium peregrinum	WP_064881303	---R-----RQV---SR	E-------
Mycobacterium phlei	WP_081491265	-V-R-S---RQV---SR	E-------
Mycobacterium porcinum	WP_075924300	---R-----RQV---SR	E-------
Mycobacterium rhodesiae	WP_081478992	-V-R-S---MAV---SR	E----A--
Mycobacterium rufum	KGI66265	---Q-S---MAV---SH	E--Q-A--
Mycobacterium rutilum	SEH56621	-V-R-S---RQV---SR	E-------
Mycobacterium saskatchewanense	WP_085254403	---R-S---RQV---SH	E-------
Mycobacterium scrofulaceum	WP_067269972	---R-S---RQV---SH	E-------
Mycobacterium senegalense	KLI06628	---R-----RQV---SR	E-------
Mycobacterium senuense	WP_085081565	-V-R-S---AQV----H	E-------
Mycobacterium septicum	WP_044514525	---R-----RQV---SR	E-------
Mycobacterium setense	WP_039326834	---R-----RQV---SR	E-------
Mycobacterium sherrisii	WP_069401665	---R-S---RQV---SH	E-------
Mycobacterium shigaense	BAX90529	---R-S--IRGV---SH	E-------
Mycobacterium shimoidei	WP_069394236	-V---S---RQV---SH	E-------
Mycobacterium shinjukuense	WP_083045799	-----S---RQV---SH	E-------
Mycobacterium simiae	WP_061557958	---R-S---RQV---SH	E-------
Mycobacterium sinense	WP_065023698	-V-R-S---AQV----H	E-------
Mycobacterium smegmatis	WP_081319323	---R-----RQV---SR	E-------
Mycobacterium szulgai	WP_068160133	-----S---RQV---SH	E-G-----
Mycobacterium terrae	WP_085259157	-V-R-S---AQV----H	E-------
Mycobacterium thermoresistibile	WP_003924532	-A-R-----RQV--NSR	E-------
Mycobacterium triplex	WP_036466038	---R-S---RQV---SH	E-------
Mycobacterium triviale	ODR05076	-V---S---RAV---SK	E-------
Mycobacterium tuberculosis	WP_070894231	-----S---RQV---SH	E-------
Mycobacterium tusciae	WP_051469046	-V-D-----RQV---SR	E----A--
Mycobacterium ulcerans str. Harvey	EUA91811	-A---S---RQV---SH	E-------
Mycobacterium vaccae	WP_040541420	---R-S---MAV---SR	E--Q-A--
Mycobacterium vanbaalenii	WP_086008464	---R-S---MAV---SR	E--Q-A--
Mycobacterium vulneris	WP_065459110	---R-----RQV---SR	E-------
Mycobacterium wolinskyi	WP_085150304	---R-----RQV---SG	E-------
Mycobacterium yongonense	ARR85596	---R-S---RQV---SH	E-------

Supplementary Figure 41

A partial sequence alignment of a conserved region of a hypothetical protein showing a three amino acid insertion that is specific for members of the "*Abscessus-Chelonae"* clade and absent in other *Mycobacterium*.

Group	Species	Accession	92		128
**"Abscessus-Chelonae" Clade (6/6)**	*Mycobacterium abscessus*	WP_062879423	GITLSKNQSEYSRKRLAQV	AA	ETGRSAEIRMQGWEEF
	Mycobacterium chelonae	WP_070920981	-------------T---K-	--	----T-----------
	Mycobacterium franklinii	WP_070938308	-------------------	--	----T-----------
	Mycobacterium immunogenum	WP_043076665	-------------A---K-	-E	----T-----------
	Mycobacterium salmoniphilum	WP_078323248	-------------T---K-	-E	----T-D---------
	Mycobacterium saopaulense	WP_083014881	-------------T--EK-	-E	----T-----------
**Other *Mycobacterium* (0/75)**	*Mycobacterium alsense*	WP_083136201	-----R--F---KAK--GL		P-Q--V-V-L------
	Mycobacterium aromaticivorans	WP_036343168	----------FA-----KL		D-N--I-V-L------
	Mycobacterium arosiense	WP_083066114	-----R--FD--KHK--GL		P-S-NI-V-L------
	Mycobacterium asiaticum	OBJ61363	-----R--F---KNK--GL		P-S-NI-V-L------
	Mycobacterium aurum	WP_087030698	------A----A-Q---KL		D-D-TID--L------
	Mycobacterium avium	WP_062888089	-----R--F---K-L--GF		P-D-NI-V-L------
	Mycobacterium avium subsp. avium	ETB17275	-----R--F---K-L--GF		P-D-NI-V-L------
	Mycobacterium avium subsp. hominissuis	ETB25142	-----R--F---K-L--GF		P-D-NI-V-L------
	Mycobacterium avium subsp. paratuberculosis	ETA96464	-----R--F---K-L--GF		P-D-NI-V-L------
	Mycobacterium bacteremicum	WP_083060342	------A----A-E---KI		D-N--ID--L------
	Mycobacterium boenickei	WP_077742280	-L---GE-R--AIDK-SK-		P-Q-NV-V-L------
	Mycobacterium bovis	WP_044082231	-----R--F---KAK--KI		P-E--VQV-L---D--
	Mycobacterium branderi	WP_083130392	-----R--C---KA---KI		P-K-NV-V-L------
	Mycobacterium canariasense	WP_062658540	------A--D-A-E---KI		D-K-NV---L------
	Mycobacterium canettii	WP_015289067	-----R--F---KAK--KI		P-E--VQV-L------
	Mycobacterium colombiense	OBB83872	-----R--F---KNM--GL		P-N-NI-V-L------
	Mycobacterium confluentis	WP_085156007	--------------L-GGL		D-K--V----------
	Mycobacterium conspicuum	WP_085235621	-----R--F---KAK--KI		P-E-TV-V-L------
	Mycobacterium diernhoferi	WP_073857162	------A----A-E---KL		--E-TVD--L------
	Mycobacterium engbaekii	WP_085127762	-L---GE-RQ-AID---K-		--N-NV-V-L------
	Mycobacterium europaeum	WP_085240805	-----R--FA--KAK--AI		P-E--V-V-L------
	Mycobacterium fallax	WP_085096114	---------A----L-SGL		D-Q-TV-V--------
	Mycobacterium florentinum	WP_085222764	-----R--F---KA-M-K-		P-E--V---L------
	Mycobacterium fortuitum	WP_061265496	------E--D-A-----KI		D-N-NVD--L------
	Mycobacterium fragae	WP_085198085	-----R--YQ--KAK--SI		P-N-T--A-L------
	Mycobacterium genavense	WP_025737023	-----R--F---K----K-		P-E-TV---L------
	Mycobacterium goodii	WP_049743036	------A----A-A---KL		D-N--V-V-L------
	Mycobacterium gordonae	OBJ87042	-----R--F---K-M--GL		P-D-NI-V-L------
	Mycobacterium haemophilum	WP_054880743	-----R--Y---KAK--AI		P-E-TV-V-L------
	Mycobacterium heidelbergense	WP_083074481	-----R--F---KAK--GM		P-Q-NV---L------
	Mycobacterium houstonense	WP_066901554	-L---GE-R--AIEK--KI		P-E-NV-V-L------
	Mycobacterium insubricum	WP_083031589	---------A----L--GI		P-Q-TV-V--------
	Mycobacterium interjectum	WP_066917691	-----R--F---KA---GI		P-E-NV-V-L------
	Mycobacterium intracellulare	OBH35116	-----R--F---K-L--GL		P-D-NI-V-L------
	Mycobacterium kumamotonense	WP_083080596	-L---ER-R--AINK-SKA		DSN-NF-V-L------
	Mycobacterium kyorinense	WP_045375891	-----R--Y---KAK--EI		P-E-T--A-L------
	Mycobacterium lentiflavum	CQD05482	-----R--F------M-KI		P-D-TV---L------
	Mycobacterium liflandii	WP_015354448	-----R--F---KAKM-KI		P-E-TV-V-L------
	Mycobacterium litorale	WP_078021761	-----------A-R---RL		D-E--I-V-L------
	Mycobacterium mageritense	WP_036429641	------A---WA-E---KI		D-N-TVD--L------
	Mycobacterium malmoense	OCB22823	-----R--F---KNK--GL		R-N-NV-V-L------
	Mycobacterium marinum	WP_012392738	-----R--F---KAKM-KI		P-E-TV-V-L------
	Mycobacterium marseillense	WP_083019436	-----R--F---KNK--GL		P-D-NI-V-L------
	Mycobacterium mucogenicum	WP_064858659	------A----A-A---KI		D-N--I----------
	Mycobacterium mungi	WP_064319828	-----R--F---KAK--KI		P-E--VQV-L---D--
	Mycobacterium nebraskense	WP_046182972	-----R--F---KSK--GL		R-N-NV-L-L------
	Mycobacterium neoaurum	WP_030134233	------A----A-E---KI		D-K-NV---L------
	Mycobacterium neworleansense	CRZ16344	------E--D-A-----KI		D-N-NV---L------
	Mycobacterium orygis	WP_003402320	-----R--F---KAK--KI		P-E---QV-L---D--
	Mycobacterium paraense	WP_085097655	-----R--F---KA---SI		P-E-TV-V-L------
	Mycobacterium paraffinicum	WP_073874665	-----R--FA--KAKM-AI		P-E--V-V-L------
	Mycobacterium paraseoulense	WP_083172395	-----R--F----AK--TI		P-N-NV-V-L------
	Mycobacterium parmense	WP_085271777	-----R--F---KAM--RI		P---NV-V-L------
	Mycobacterium peregrinum	WP_064886342	-L---GE-R--AIEK--KI		P-E-NV-V-L------
	Mycobacterium porcinum	WP_069426150	------E--D-A-----KI		D-N-NV---L------
	Mycobacterium pseudoshottsii	WP_086085600	-----R--F---KAKM-KI		P-E-TV-V-L------
	Mycobacterium rhodesiae	WP_083118158	----------FA-----KL		D-N--I-V-L------
	Mycobacterium saskatchewanense	WP_085258728	-----R--F---KAK--TI		P-E-HV-V-L------

Group	Species	Accession	Sequence	
Other *Mycobacterium* (0/75)	*Mycobacterium scrofulaceum*	WP_083177268	-----R--FD--KSK-SGL	R-N-NV-V-L------
	Mycobacterium septicum	WP_044515630	------E----A-----K-	D-N-NI---L------
	Mycobacterium setense	WP_039377534	------A--D-A-E---KI	D-E-NI---L------
	Mycobacterium sherrisii	WP_069400699	-----R--F---K-K-SAI	P-N--V-V-L------
	Mycobacterium shigaense	BAX90852	-----R--F---KAK--KI	P-D-TV-V-L------
	Mycobacterium simiae	WP_061558205	-----R--F---K-K-STI	P-D-TV-V-L------
	Mycobacterium sinense	WP_065025310	-L---GE-R--AIDK-SK-	D-N--V-V-L------
	Mycobacterium smegmatis	WP_003892339	------A--D-A-E---KI	--N--V-V-L------
	Mycobacterium terrae	WP_085259816	-L---GE-R--AVEK--K-	K-N-NV-V-L------
	Mycobacterium thermoresistibile	WP_003926578	-----R--Y---KA----I	D-A-T--V-L----Q-
	Mycobacterium triplex	WP_036466507	-----R--F---KA-M-K-	P-E-TV---L------
	Mycobacterium tuberculosis	WP_065022395	-----R--F---KAK--KI	P-E--VQV-L---D--
	Mycobacterium ulcerans	WP_011742093	-----R--FD--KAKM-KI	P-E-TV-V-L------
	Mycobacterium vaccae	WP_003932775	-----R--F---KALM-GL	D-K-T--V-L------
	Mycobacterium vulneris	WP_065509452	-L---GE-R--AIEK-SK-	P-N-NV-V-L------
	Mycobacterium wolinskyi	WP_067856260	-L---GE-R--AIDK--K-	P-N-NV-V-L------
	Mycobacterium yongonense	OCB19383	-----R--F---K-M--GL	P-D-NI-V-L------

Supplementary Figure 42

A partial sequence alignment of a conserved region of SAM-dependent methyltransferase showing a two amino acid insertion that is specific for members of the "*Abscessus-Chelonae"* clade and absent in other *Mycobacterium*.

Group	Species	Accession	12		47
***"Abscessus-Chelonae"* Clade (6/6)**	*Mycobacterium abscessus*	WP_062879423	QSIYDISDEFYGLFLDE	E	TMGYTCAYFERDDLTLAE
	Mycobacterium chelonae	WP_070920981	-----------------	-	------------------
	Mycobacterium franklinii	WP_070938308	-----------------	-	------------------
	Mycobacterium immunogenum	WP_043076665	-----------------	-	------------------
	Mycobacterium salmoniphilum	WP_078323248	-----------------	-	------------------
	Mycobacterium saopaulense	WP_083014881	-----------------	-	------------------
**Other *Mycobacterium* (0/94)**	*Mycobacterium alsense*	WP_083136201	-----V-N--FA----P		-------------M--E-
	Mycobacterium aromaticivorans	WP_036343168	-----V--D-FA---GP		------G-Y----M--D-
	Mycobacterium arosiense	WP_083066114	-----V-N--FA----A		-------------M--E-
	Mycobacterium asiaticum	WP_065035458	-AH--------A--V-P		-RT-S--------M--E-
	Mycobacterium aurum	WP_087030698	-------N---A---GP		------G-Y--E-MN-E-
	Mycobacterium avium	WP_062888089	-----V----FA----P		--A----F-----M--E-
	Mycobacterium avium subsp. avium	ETB17275	-----V----FA----P		--A----F-----M--E-
	Mycobacterium avium subsp. hominissuis	ETB25142	-----V----FA----P		--A----F-----M--E-
	Mycobacterium avium subsp. paratuberculosis	ETA96464	-----V----FA----P		--A----F-----M--E-
	Mycobacterium boenickei	WP_077742507	-------N---A---GP		------G-Y--E-MN-E-
	Mycobacterium bohemicum	WP_085179372	-----V----FA----S		-------------M--E-
	Mycobacterium bovis	WP_044082231	M--V----FS----P		--A--------E-M--E-
	Mycobacterium branderi	WP_083130392	----------FA----P		-------------M--E-
	Mycobacterium canariasense	WP_062658540	-------N---A---GP		------G-Y--E-M--D-
	Mycobacterium canettii	WP_015289067	-----V----FS----P		--A--------E-M--E-
	Mycobacterium canettii	WP_044109634	--A-----D-FA----P		-WV----------M--E-
	Mycobacterium celatum	WP_062539269	-A---L----FS----P		--T----------M--E-
	Mycobacterium colombiense	WP_065027725	-----V----FA----D		-------------M--E-
	Mycobacterium confluentis	WP_085156007	-----V----FA----P		-------------M--E-
	Mycobacterium conspicuum	WP_085235621	-----V----FA----P		--A----------M--E-
	Mycobacterium diernhoferi	WP_073857162	-------N---A---GP		------G-Y--E-MN-E-
	Mycobacterium engbaekii	WP_085126580	-AA-----D-FS----P		--A----------MS-E-
	Mycobacterium europaeum	WP_085240805	-----V----FA----P		-----------G-M--E-
	Mycobacterium fallax	WP_085096114	-----V----FA----S		-------------MS-E-
	Mycobacterium florentinum	WP_085222764	-----V----FA----P		-------------M--E-
	Mycobacterium fortuitum	WP_061265496	-------ND--A---GP		------G-Y--E-MN-E-
	Mycobacterium fragae	WP_085198085	-----L----FS----P		A-V------A---M----
	Mycobacterium gastri	WP_036410582	-AA-----D-F-----P		-WV----------M--Q-
	Mycobacterium genavense	WP_025737023	-----V----FA----P		------G------M--E-
	Mycobacterium goodii	WP_049743036	-------N--FA---GP		------G-Y--E-M--D-
	Mycobacterium gordonae	WP_065044475	-AT-----D-FA----P		N-V----------M--E-
	Mycobacterium haemophilum	WP_047313557	--A-----D-FA----P		-WV----------M--E-
	Mycobacterium heidelbergense	WP_083073988	-AA-----D-F-----P		-WV----------M--E-
	Mycobacterium heraklionense	WP_064889007	-AA-----D-F-----P		--A----------M--E-
	Mycobacterium houstonense	WP_066902761	-------N---A---GP		------G-Y--E-MN-E-
	Mycobacterium icosiumassiliens	WP_067970353	-AA-----D-F-----P		--A----------M--E-
	Mycobacterium insubricum	WP_083031589	-----V--D-FA----P		-------------ME-E-
	Mycobacterium interjectum	WP_066907221	--T-----D-F-----P		N-V----------M--E-
	Mycobacterium intracellulare	OBH66125	-----V----FA----P		-------------M--E-
	Mycobacterium kansasii	WP_063469906	-AA-----D-F-----P		-WV----------M--Q-
	Mycobacterium koreense	WP_085304410	-------N---A---GP		------G-Y----T-TS-
	Mycobacterium kyorinense	WP_045375891	-A---L----FS----P		--T----------M--E-
	Mycobacterium lacus	WP_085157480	--A-----D-FA----P		-WV----------M--Q-
	Mycobacterium lentiflavum	CQD05482	-----V----FA----P		-------------M--E-
	Mycobacterium leprae	WP_010908600	--A-----D-FA----P		-WV----------M--E-
	Mycobacterium lepromatosis	WP_045843474	--T-----D-FA----P		-WV----------M--E-
	Mycobacterium liflandii	WP_015354448	-----V----FA----P		--A----------M--E-
	Mycobacterium litorale	WP_078021761	-----V--D-FA---GQ		------G-Y--E-M--D-
	Mycobacterium longobardum	WP_085262966	-AA-------FS----P		--A----------M--E-
	Mycobacterium mageritense	WP_036429641	-------N--FA---GP		------G-Y--E-M--D-
	Mycobacterium malmoense	OCB24949	-----V-N--FA----P		--A----------M--E-
	Mycobacterium mantenii	WP_083093251	-AT-----D-FA----P		N-V----------M--E-
	Mycobacterium marinum	WP_012392738	-----V----FA----P		--A----------M--E-
	Mycobacterium marseillense	WP_083019436	-----V----FA----P		-------------M--E-
	Mycobacterium mucogenicum	WP_064858659	-------N--FA-W-GP		------G-Y--E-M--E-
	Mycobacterium mungi	WP_064319828	-----V----FS----P		--A--------E-M--E-
	Mycobacterium nebraskense	WP_046182972	-----V-N--FA----P		--A------A---M--E-
	Mycobacterium neoaurum	WP_030134233	-------ND-FA---GP		------G-Y--E-M--D-

Group	Species	Accession	Sequence (region 1)	Sequence (region 2)
**Other *Mycobacterium* (0/94)**	*Mycobacterium neworleansense*	CRZ16344	-------N---A---GP	------G-Y--E-MN-E-
	Mycobacterium orygis	WP_003402320	-----V----FS----L	--A--------E-M--E-
	Mycobacterium palustre	WP_085081074	-AA-------F-----P	-WV----------M--Q-
	Mycobacterium paraense	WP_085097172	-AA-----D-F-----P	-WV----------M--E-
	Mycobacterium paraffinicum	WP_073874665	-----V----FA----P	-----------E-M--E-
	Mycobacterium paraseoulense	WP_083172395	----------FA----P	-------F--Q--M--E-
	Mycobacterium parmense	WP_085271777	----------F-----P	-------------M--E-
	Mycobacterium peregrinum	WP_055110489	-------N---A---GP	------G-Y--E-MN-E-
	Mycobacterium porcinum	WP_069426150	-------N---A---GP	------G-Y--E-MN-E-
	Mycobacterium pseudoshottsii	WP_086085600	-----V----FA----P	--A----------M--E-
	Mycobacterium rhodesiae	WP_083118158	-----V--D-FA---GP	------G-Y--E-M--D-
	Mycobacterium riyadhense	WP_085249157	--A-----D-F-----P	-WV----------M--E-
	Mycobacterium rutilum	SEH77865	--H--L-----Q----P	-QT-S--------M--E-
	Mycobacterium saskatchewanense	WP_085258119	-AA-----D-F-----P	-WV----------M--E-
	Mycobacterium scrofulaceum	WP_083177268	-----V-N--FA----P	--A----------M--
	Mycobacterium senuense	WP_085083843	-AA-------FS----P	S-A----------MS-E-
	Mycobacterium septicum	WP_044515630	-------N---A---GP	------G-Y--E-MN-E-
	Mycobacterium setense	WP_039377534	-------N--FA-W-GP	------G-Y--E-M--D-
	Mycobacterium sherrisii	WP_069399661	-AT-----D-FA----P	N-V----------M--E-
	Mycobacterium shigaense	BAX91002	-AT-----D-FA----P	N-V----------M--E-
	Mycobacterium shimoidei	WP_069397450	-A-----N--FS----P	--T----------M--E-
	Mycobacterium shinjukuense	WP_083052153	-AA-------F-----P	-WV----------M--E-
	Mycobacterium simiae	WP_061558205	-----V----FA----P	-------------M--E-
	Mycobacterium sinense	WP_064855508	-AA-------FS----P	S-A----------MS-Q-
	Mycobacterium smegmatis	WP_003892339	-------N--FA---GP	------G-Y--E-M--D-
	Mycobacterium thermoresistibile	WP_003926578	-----V--D-FA---GP	-------------M--D-
	Mycobacterium triplex	WP_036466507	-----V----FA----P	-------------M--E-
	Mycobacterium triviale	WP_069392520	-------N---A---GP	------G-Y----T-TS-
	Mycobacterium tuberculosis	WP_065022409	-----V----FS----P	--A--------E-M--E-
	Mycobacterium ulcerans	WP_011742093	-----V----FA----P	--A----------M--E-
	Mycobacterium vaccae	WP_003932361	--H--L-----R----P	-QT-S--------M--E-
	Mycobacterium vanbaalenii	WP_011778077	-----V-N--FA---GP	------S----E-M--D-
	Mycobacterium vulneris	WP_085288105	-----V----FA----D	-------------M--E-
	Mycobacterium wolinskyi	WP_067849249	-------ND-FA---GP	------G-Y----M--D-
	Mycobacterium xenopi 3993	EUA20661	-AH--L----FA----P	-RT-S--------M--E-
	Mycobacterium yongonense	WP_065508267	-AT--L--D-FS----R	--A----------MG-E-
**Other bacteria**	*Achromobacter denitrificans*	WP_062682161	H--L--D--A-W--P	RRV-S---YR-P-M---Q
	Acidovorax wautersii	SFF21693	-FH--L--D--A-W--P	RRV-S---YS-A-M---Q
	Bordetella petrii	WP_085970219	H--L--D--A-W--P	RRV-S---YRTP-MS--Q
	Candidimonas nitroreducens	WP_088604427	-FH--L--D--A-W--P	RRV-S----REP-M-V-Q
	Gordonia soli	WP_007621030	--H--L--D-FA----P	SRT-S--------Y--GQ
	Lactobacillus buchneri	WP_013728563	-KH---GND--RMW--K	S-T-S-----H--D--E-
	Lampropedia cohaerens	WP_046741577	-FH---G-D----W--P	RRV-S----D-P-A---Q
	Laribacter hongkongensis	WP_081666695	H--LGND--R-W--D	S-S-S----A-P-M----
	Lechevalieria aerocolonigenes	WP_030466687	H--L-ND--R-V--P	S-A-S----TS-EDS-VQ
	Lentzea albidocapillata	SMD12454	H--L-ND--R-V--P	S-A-S----TSE-EP--Q
	Mesorhizobium erdmanii	WP_027053303	-RH--L-G-L-R-----	D-Q-S-----QP-M--D-
	Nocardia miyunensis	WP_084530897	-AH--V--D--R----P	S-T-S----V-EGM--EQ
	Paraburkholderia terrae	WP_086908515	-HH-----D--A-W--P	LRV-S----SAP-MS---
	Rhodococcus gordoniae	WP_064062994	-TH--L--D-FE----P	-RT-S-------GMS-EQ
	Segniliparus rugosus	WP_007468872	--H--L--D--A----P	-RT-S------PSMS-E-
	Streptococcus sobrinus	WP_019770744	--H---GND--R-W--K	-AT-S-----HE-DS-E-
	Tsukamurella tyrosinosolvens	KXP01245	-AH--L----FA----P	-RT-S-------GM--EQ
	Variovorax paradoxus	WP_026346321	H--V--D--A-W--P	RRV-S----RTP-----Q
	Williamsia muralis	WP_062795895	--H--L----FA----P	-RT-S--------M--E-

Supplementary Figure 43

A partial sequence alignment of a conserved region of SAM-dependent methyltransferase showing a one amino acid insertion that is specific for members of the "*Abscessus-Chelonae*" clade and absent in other bacteria.

Group	Species	Accession	189		231
"Fortuitum-Vaccae" Clade (46/46)	*Mycobacterium aromaticivorans*	WP_036341761	GHHEIGLLTMRLGNERRV	GDQAA	GLVSADRLRDSRFHVQTERI
	Mycobacterium aurum	WP_087027717	--R----------R-WPH	EHTKP	MVADPQ--DSPH----R--V
	Mycobacterium bacteremicum	WP_083058472	--R----------R-WPL	ETTTP	MVADPE--NSPH----C--V
	Mycobacterium brisbanense	WP_062828483	--RD---------RDWPN	PSGKP	AVAQP--VQSPH----R---
	Mycobacterium canariasense	WP_062655941	--R----------RDWPH	DHPTP	AVAPPE--NSPH----R--
	Mycobacterium celeriflavum	WP_083004729	--R----------RD-PR	SGPGP	T-ADP---QTPH----R---
	Mycobacterium chlorophenolicum	KMO83493	--R----------RDWPH	-GPGP	A-ADPE-V-TPD----S---
	Mycobacterium chubuense	WP_014817850	--R----------R-LPH	-GPRP	A-ADPE-VATPH----S---
	Mycobacterium conceptionense	WP_076216119	--RD---------RDWPH	E-TKP	A-ADPG-VLSPH----R---
	Mycobacterium cosmeticum	WP_036397638	--R----------RDWPH	EHPSP	AVAQPE--NSPH----R--
	Mycobacterium diernhoferi	WP_073856817	--R---V------R-WPH	ESTKP	MVADPE--NSPH----R--V
	Mycobacterium doricum	WP_085188425	--R----------L--PD	-SRQP	AVADPEA--TP-----R---
	Mycobacterium elephantis	WP_046752947	--T--A------DRDWPH	PGSRP	TVADPE--GMPH----R---
	Mycobacterium flavescens	WP_069416537	--R----------RD-PA	-EVG-	AVADPQ--QTPH----R---
	Mycobacterium fortuitum	WP_064850989	--RD---------RDWPH	ESTKP	A-ADP--V-SPH----R---
	Mycobacterium gilvum	WP_011892354	--R----------RDWPH	- PGP	A-ADPE-V-APN----C---
	Mycobacterium goodii	WP_049744668	--RD---------RDWPH	--PRP	A-ADP--VLSPH----R---
	Mycobacterium hassiacum	WP_005630626	--R----------RDWPH	PKPRP	AVADPQ-VESPH----R---
	Mycobacterium holsaticum	WP_069405966	--T--A------DRDWPH	PGSQP	TVADPQ--GMPH----R---
	Mycobacterium houstonense	WP_066902456	--RD---------RDWPH	DGTKP	A-ADP--VLSPH----R---
	Mycobacterium iranicum	WP_064279742	--R----------RDWPH	-GPRP	A-ADP--V-TPH----S---
	Mycobacterium komanii	CRL75788	--R--AV------RD-PD	-GPGS	TVADPE--QTPH----R---
	Mycobacterium litorale	WP_078021116	--------A----TG-A-	-G-T-	-VATDE-----Q----S---
	Mycobacterium llatzerense	WP_071288441	--R----------RHWPH	--AQS	AVADPE--TSQH----C---
	Mycobacterium mageritense	WP_036432161	--RD---------RDWPH	-NPKP	A-ADP---LTPH----R---
	Mycobacterium malmesburyense	CRL69197	--R-V--------RD-PE	-GPGP	TVADPE--QTPH----R---
	Mycobacterium moriokaense	WP_083155054	--R--A-------RDWPH	-GPVP	A-ADPE-VQTPH----R---
	Mycobacterium mucogenicum	WP_064860405	--R----------RQWPH	--AQS	AVADPE--TAQH--A-C---
	Mycobacterium neoaurum	WP_030137084	--R----------R-WPL	ETT-P	MVADPE--NSPH----C--V
	Mycobacterium neworleansense	CRZ19116	--RD---------RDWPH	-GTKP	A-ADP--VLSPH----R---
	Mycobacterium novocastrense	WP_067394019	--R----------RD-PQ	-GAGP	TVADPQ--QTPH----R---
	Mycobacterium obuense	WP_046362873	--R----------RDWPH	SGRGP	A-ADP--V-NPQ----S---
	Mycobacterium parafortuitum	WP_083141994	--R----------RDWPH	-PRP-	-ADP--V-TPH----S---
	Mycobacterium peregrinum	WP_064883819	--QH---------RDWPH	ESMKP	A-ADP--VLSPH----R---
	Mycobacterium phlei	WP_061481250	--T--A-------R-HPS	SGTGP	TVADPE--KSPH----R---
	Mycobacterium porcinum	WP_069427935	--RD---------RDWPH	DGTKP	A-ADPG-VLSPH----R---
	Mycobacterium rhodesiae	WP_083118787	--R---------------	----S	-V--P---S-----------
	Mycobacterium rufum	KGI70031	--R----------RDWPH	-GPRP	A-ADP--V-TPD----S---
	Mycobacterium rutilum	WP_083407016	--R----------RD-PA	--VG-	AVADPE--QTPH----R---
	Mycobacterium septicum	WP_044520796	--RD---------RDWPH	ESTKP	A-ADP--VLSPH----R---
	Mycobacterium setense	WP_039325624	--RD---------RDWPH	AGTKS	A-ADP--VLSPH----R---
	Mycobacterium smegmatis	WP_003897443	--RD---------RDWPH	--PRP	A-ADPE-VLSPH----R---
	Mycobacterium tusciae	WP_006243774	--R----------RDWPH	- AKP	A-ADPE-VKTPH----R---
	Mycobacterium vaccae	WP_003930652	--R----------RDWPH	-GV-P	A-ADPE---TPH-Q--G---
	Mycobacterium vulneris	WP_065514398	--RD---------RDWPH	DGTKP	A-ADP--VLSPH----R---
	Mycobacterium wolinskyi	WP_067850494	--RD---------RDWPH	-SPKP	A-ADPE-VLSPH----R---
Other *Mycobacterium* (1/59)	*Mycobacterium abscessus subsp. abscessus*	SHW90938	--R----------RQWPH	--AQS	AVADPE--TSQH----C---
	Mycobacterium africanum	AMC66132	--R-L--------RD--Q		D--D-E---SPT-D--R---
	Mycobacterium alsense	WP_083139956	--R----------RD--Q		---D-----SPA-D--R---
	Mycobacterium angelicum	WP_083115163	--R----------RD--Q		D--D-E--KSPT-D--R---
	Mycobacterium arosiense	WP_083066170	--R----------RD--Q		---D-E---SPA-D--R---
	Mycobacterium asiaticum	WP_065034552	--R----------DD--M		---D-E---SLT-D--R---
	Mycobacterium avium	WP_087614316	--R----------RD--Q		---D-E---SPA-D--R---
	Mycobacterium avium subsp. avium 2285	EUA25141	--R----------RD--H		---D-E---SPA-D--R---
	Mycobacterium avium subsp. hominissuis	ETB26830	--R----------RD--Q		---D-E---SPA-D--R---
	Mycobacterium avium subsp. paratuberculosis	ETB36530	--R----------RD--Q		---D-E---SPA-D--R---
	Mycobacterium bohemicum	WP_085182776	--R----------RD--Q		--AD-E--QSPA-D--R---
	Mycobacterium bovis	WP_046026302	--R-V--------RD--Q		D--D-E---SPT-D--R---
	Mycobacterium branderi	WP_083131115	--RQ--------RRD--E		-V-NSG---SAT-Q--A---
	Mycobacterium canettii	WP_015291558	--R-L--------RD--Q		D--D-E---SPT-D--R---
	Mycobacterium celatum	WP_062540359	--RQ----S---RRD--E		-V-NVG---SAT-Q--A---
	Mycobacterium colombiense	WP_064882073	--R----------RD--Q		---D-----SPA-D--R---
	Mycobacterium conspicuum	WP_085230983	--R----------RD--H		D--D-E--KSPI-D--R---
	Mycobacterium europaeum	WP_085239753	--R----------RD--Q		---DV----SPA-D--R---

**Other *Mycobacterium* (1/59)**	*Mycobacterium florentinum*	WP_085223921	--R----------R---Q	A-AD-E---SQT-D--R---
	Mycobacterium fragae	WP_085198718	--R---V-----RRD--Q	---DRK--AS-T-Q--S--V
	Mycobacterium gastri 'Wayne'	ETW26080	--RH---------HD--Q	---D-E--KSPA-D--R---
	Mycobacterium gordonae	WP_065047466	--R----------RD--Q	---DPE--NSPT-D--R---
	Mycobacterium haemophilum	WP_047316714	--R----------RD--Q	D--D----QSPT-D--R---
	Mycobacterium heidelbergense	WP_083073704	--R----------RD--Q	---D-----SPA-D--R--V
	Mycobacterium interjectum	WP_066916456	--R----------RD--Q	---D-----SPA-D--R---
	Mycobacterium intermedium	WP_069419459	--R----------RD--Q	D--D-E--QSPT-D--R---
	Mycobacterium intracellulare	WP_064938444	--R-L--------RD--H	---D-E---SPA-D--R---
	Mycobacterium kansasii	WP_063467912	--RD---------HD--Q	---D-E--QSPA-D--R---
	Mycobacterium kubicae	WP_085074718	--R----------SD--Q	---D-E---SPA-D--R---
	Mycobacterium kyorinense	WP_065012904	--R---V-----HR---Q	---A-E---SAM-Q--G--V
	Mycobacterium lacus	WP_085162383	--R----------RD--Q	AP-D-E--QSPT-D--R---
	Mycobacterium lentiflavum	CQD21659	--R----------QD--Q	DVAD-E---SLT-D--R---
	Mycobacterium malmoense	WP_065445767	--R----------RD--Q	---D-----SPA-D--R---
	Mycobacterium mantenii	WP_083095424	--R----------RD--Q	---GT----SPT-D--R---
	Mycobacterium marinum	WP_012396592	--RQ---------RD--Q	D--D-E--KSPT-D--R---
	Mycobacterium nebraskense	WP_085165446	--R----------R---Q	---D-----SPA-D--R---
	Mycobacterium noviomagense	WP_083085536	--R---------SR-PHQ	-V-DPQ---SPT----AD--
	Mycobacterium palustre	WP_085077740	--R----------RD--Q	---D-E---SPA-D--R---
	Mycobacterium paraense	WP_085095893	--R----------R-L-Q	---D-----SPA-D--R---
	Mycobacterium paraffinicum	WP_073871729	--R----------RD--Q	---D-E---SPA-D--R---
	Mycobacterium parascrofulaceum	EFG77605	--R----------RD--Q	---D-----SPA-D--R---
	Mycobacterium paraseoulense	WP_083174493	--RD---------RD--Q	---D-A---SPA-D--R---
	Mycobacterium parmense	WP_085268213	--R----------R---Q	--AD-----SPA-D--R---
	Mycobacterium pseudoshottsii	WP_086085161	--RQ---------RD--Q	D--D-E--KSPT-D--R---
	Mycobacterium riyadhense	WP_085251182	--R----------RD--Q	D--DSE--KSPT-D--R---
	Mycobacterium saskatchewanense	WP_085255317	--R----------RD--Q	--AD-----SPA-D--R---
	Mycobacterium scrofulaceum	WP_067279040	--R----------RD--Q	---DGE---SPA-D--R---
	Mycobacterium sherrisii	WP_085166426	--T----------QD--Q	-VAD-----SLM-S--R---
	Mycobacterium shigaense	BAX94683	--R----------RD--Q	A-AD-----SLK-D--R---
	Mycobacterium shimoidei	WP_069394764	--R---------RPDG-N	-P-D-Q---SAT-Q--SD-V
	Mycobacterium shinjukuense	WP_083047295	--RQL--------RD--Q	---D-E--KSPT-D--R---
	Mycobacterium simiae	WP_061559896	--R-V--------RD--Q	DVAD-E---SLM-S--R---
	Mycobacterium szulgai	WP_085670177	--R----------RD--Q	D--D-E--KSPT-D--R---
	Mycobacterium triplex	WP_036471787	--R----------QD--Q	-VADS----SLT-D--R---
	Mycobacterium tuberculosis	WP_070893018	--R-L--------RD--Q	D--D-E---SPT-D--R---
	Mycobacterium ulcerans	WP_011741790	--RQ---------RD--Q	D--D-E--KSPT-D--R---
	Mycobacterium vulneris	WP_085292191	--R----------RD--Q	---D-G---SPA-D--R---
	Mycobacterium yongonense	WP_065501623	--R----------RD--H	---D-E---SPA-D--R---
**Other bacteria**	*Amycolatopsis benzoatilytica*	WP_027928391	--RQV-I-C----R--ND	DF---E-QSGAH----RT-L
	Amycolatopsis saalfeldensis	SEP25100	--RQV-V-C----R--ND	DF-NPQ-QSSAH----RT-L
	Corynebacterium terpenotabidum	WP_020440452	--TR--I-CI--DRDPNN	-P---A--AGAHL---RD-V
	Corynebacterium variabile	WP_014009065	--TR--I-CI--DRVPNN	-P---E--AGAHL---RD-V
	Lechevalieria aerocolonigenes	WP_030471030	--RR--VVC---ARD-ND	-F-T---QHAAH----RN-L
	Pseudonocardia autotrophica	WP_037045280	--RK--V-C----PQ-HD	-PADLV-QENTSYP--R---
	Rhodococcus defluvii	WP_031939517	--K---V-C----RD-SD	-V-GSG--HS-N----R---
	Rhodococcus equi ATCC 33707	EGD24243	--V---V-C----RD-AD	-V-QGE--HAPN----R---
	Rhodococcus fascians	WP_032380155	--EHLAV-C----RD--D	-V-PPG--A--H----R---
	Sciscionella marina	WP_026196996	--KHV-V-C----RD-ND	-F--LA-QHSA-Y---RA-L

Supplementary Figure 44

Detailed sequence information for the five amino acid insertion found in LacI family transcriptional regulator, which is shown in Figure 6. This insertion is specific for members of the *"Fortuitum-Vaccae"* clade and absent in most other bacteria.

Group	Species	Accession	243		280
“Fortuitum-Vaccae” Clade (42/47)	*Mycobacterium acapulcensis*	WP_066808156	WLHDHDVAAVAADNLMVE	DP	DPANGVEGTFLPMHMVCL
	Mycobacterium aurum	WP_087032439	-----E------------	--	--V------------L--
	Mycobacterium bacteremicum	WP_083057936	-----Q-----S------	--	---------------I--
	Mycobacterium boenickei	WP_077741563	-----Q------------	--	---------------L--
	Mycobacterium celeriflavum	WP_083003924	------------------	--	---------------I--
	Mycobacterium chlorophenolicum	WP_048473305	-----EA-----------	--	---------------L--
	Mycobacterium chubuense	WP_085980897	---ERE------------	--	---Q-----------L--
	Mycobacterium conceptionense	WP_085140460	-----Q------------	--	---------------L--
	Mycobacterium confluentis	WP_085152779	-----E------------	--	---------------L--
	Mycobacterium cosmeticum	CDO06183	-----Q-----S------	--	---------------L--
	Mycobacterium diernhoferi	WP_073854282	-----E------------	--	---------------L--
	Mycobacterium elephantis	WP_083042686	-----E------------	--	---------------L--
	Mycobacterium fallax	WP_085093862	-----R-----S------	--	---A--A--------L--
	Mycobacterium farcinogenes	WP_036393177	-----Q------------	--	---------------L--
	Mycobacterium flavescens	WP_069413997	------------------	--	---------------I--
	Mycobacterium fortuitum	WP_064850766	-----Q------------	--	---------------L--
	Mycobacterium gilvum	WP_013471213	---R-E-----S------	--	N--------------L--
	Mycobacterium goodii	WP_049745631	-----E------------	--	---S--D--------L--
	Mycobacterium hassiacum	WP_085977467	-F----------------	--	---------------L--
	Mycobacterium holsaticum	WP_069407970	----NE------------	--	---------------L--
	Mycobacterium houstonense	WP_066898554	----LQ------------	--	--------------LL--
	Mycobacterium insubricum	WP_083033293	-----R------------	--	--ES--A--------L--
	Mycobacterium iranicum	WP_064281720	---R-E------------	--	--S------------L--
	Mycobacterium komanii	CRL68347	------------------	--	------------------
	Mycobacterium mageritense	WP_019344197	-----Q------------	--	---------------L--
	Mycobacterium malmesburyense	CRL69469	-----E------------	--	---------------I--
	Mycobacterium neoaurum	CDQ46549	-----E-----S------	--	---------------I--
	Mycobacterium neworleansense	CRZ13248	-----Q------------	--	---------------L--
	Mycobacterium novocastrense	WP_067386496	------------------	--	---------------I--
	Mycobacterium parafortuitum	WP_083145423	---HRE-----S------	--	--G------------L--
	Mycobacterium peregrinum	WP_064885410	-----E------------	--	-----I---------L--
	Mycobacterium phlei	WP_003889670	------------------	--	---------------I--
	Mycobacterium porcinum	WP_069425243	-----Q------------	--	---------------L--
	Mycobacterium rufum	KGI69208	---Q-E------------	--	--SH-I---------L--
	Mycobacterium rutilum	WP_083407981	------------------	--	---------------I--
	Mycobacterium septicum	WP_044522233	-----Q------------	--	---------------L--
	Mycobacterium setense	WP_064871335	-----Q------------	--	---------------L--
	Mycobacterium smegmatis	WP_081319722	-----E------------	--	---S-----------L--
	Mycobacterium thermoresistibile	WP_040547600	---E-E------------	--	---------------L--
	Mycobacterium vaccae	WP_003928172	---H-E------------	--	--GK-----------L--
	Mycobacterium vulneris	WP_065460155	-----Q------------	--	---------------L--
	Mycobacterium wolinskyi	WP_085143791	-----E------------	--	---------------L--
	Mycobacterium aromaticivorans	WP_081845176	-MA--Q------------		N--S--D-AI-----L--
	Mycobacterium brisbanense	WP_062832106	-----E------------		N--S--D-A------L--
	Mycobacterium moriokaense	WP_083154621	-M---EI--I--------		N-VS--DEA------L--
	Mycobacterium rhodesiae	WP_083122755	-----Q------------		N--S--D--I-----L--
	Mycobacterium tusciae	WP_083125619	-----E------------		N-VP-ID-C------L--
Other *Mycobacterium* (0/64)	*Mycobacterium algericum*	WP_083037620	---E--I-----------		N-VS-----------L--
	Mycobacterium alsense	WP_083136603	-----EL--------Q--		-LVS----ITF-L-LL--
	Mycobacterium angelicum	WP_083111804	-----EI--I-----Q--		--VS-ID-L---F-LL--
	Mycobacterium arosiense	WP_083063603	-----EI----S---Q--		--VS----V---F-LLT-
	Mycobacterium asiaticum	WP_065036716	-----------S---Q--		--VS----L---F-LL--
	Mycobacterium avium	WP_062887314	-----E-----S---Q--		--VS--D-V---F-LLT-
	Mycobacterium avium subsp. avium	EUA41240	-----E-----S---Q--		--VS--D-V---F-LLT-
	Mycobacterium avium subsp. paratuberculosis	ETB08561	-----E-----S---Q--		--VS--D-V---F-LLT-
	Mycobacterium bohemicum	WP_085182564	----YE---------Q--		-LVS--D-VTF-L-LL--
	Mycobacterium branderi	ORA40171	---ER------S--IA--		TS-TEFP-VI--F--LA-
	Mycobacterium celatum	WP_062539986	-F----------------		--VS-----------L--
	Mycobacterium colombiense	WP_064882483	-----EI----S---Q--		--VS--D-V---F-LLT-
	Mycobacterium conspicuum	WP_085234634	-----EI--------Q--		-LVS----ITF-L-LL--
	Mycobacterium engbaekii	WP_085129924	---E----------V---		--VS-----Y-----L--
	Mycobacterium europaeum	WP_085242431	-----EI--------Q--		-LVS-ID-ITF-L-LL--
	Mycobacterium florentinum	WP_085223711	-----E---------Q--		-LVS----V---L-LL--
	Mycobacterium fragae	WP_085198956	-----EI-----------		--IS----------LL--
	Mycobacterium gastri	WP_036416150	-----EI----S---Q--		--VS--D-L---F-LL--
	Mycobacterium genavense	WP_025737048	-----EI--------Q--		-LVS--D-ITF-L-LL--

**Other *Mycobacterium* (0/64)**	*Mycobacterium gordonae*	WP_065045260	---H-E-----S---Q--	--VS----L---F-LL--
	Mycobacterium heckeshornense	WP_048890134	--Y---I-----------	--LS-----------L--
	Mycobacterium heidelbergense	WP_083072186	-----E-----S---Q--	-QVSD---V---F-LL--
	Mycobacterium heraklionense	WP_064889932	---A--------------	--VS-----Y--L--L--
	Mycobacterium hiberniae	WP_085135696	---E----------V---	N-VS-----Y-----L--
	Mycobacterium interjectum	WP_066915826	-----E---------Q--	-LVS--D-ITF-L-LL--
	Mycobacterium intermedium	WP_069418142	-----EI----S---Q--	--VS-I--
	Mycobacterium intracellulare	WP_064933306	-----E---------Q--	-LVS----V---L-LL--
	Mycobacterium kansasii	WP_063466640	-----E---------Q--	--VS----L---F-LL--
	Mycobacterium kubicae	WP_085075370	-----E-----S---Q--	-LVPD-D-A---L-LL--
	Mycobacterium kumamotonense	WP_065289859	---E--------------	N-VS-----------L--
	Mycobacterium kyorinense	WP_065016257	------I-----------	--VS--K--------L--
	Mycobacterium lacus	WP_085162254	---H-EI--------Q--	--VS----L---F-LL--
	Mycobacterium lentiflavum	CQD20876	-----EI--------Q--	-LVS-ID-VTF-L-LL--
	Mycobacterium liflandii 128FXT	AGC64477	-----E-----S---Q--	--VS--D-
	Mycobacterium longobardum	WP_085263206	---E-N------------	--VS--D--------L--
	Mycobacterium malmoense	WP_065446102	-----E-----S---Q--	-LVSD-D-A---L-LL--
	Mycobacterium mantenii	WP_083093801	-----QI--------Q--	-LVS--D-ITF-L-LL--
	Mycobacterium marinum	WP_012396310	-----E-----S---Q--	--VS--D-
	Mycobacterium marseillense	WP_083014919	-----E---------Q--	-LVS----V---L-LL--
	Mycobacterium nebraskense	WP_046182228	-----EI--------Q--	-LVS-ID-ITF-L-LL--
	Mycobacterium nonchromogenicum	WP_085138500	---ER-------------	--VS-----Y--L--L--
	Mycobacterium noviomagense	WP_083087486	-FY---------------	--VS-----------L--
	Mycobacterium palustre	WP_085076447	-----EI--------Q--	-LVS----ITF-L-LL--
	Mycobacterium paraintracellulare	AFC52267	-----EA--------Q--	-LVS----V---L-LL--
	Mycobacterium paraseoulense	WP_083168384	-----E-----S---Q--	-LVS----V---L-LL--
	Mycobacterium parmense	WP_085270168	-----E-----S---Q--	--VS--D-V---L-LL--
	Mycobacterium pseudoshottsii	WP_086085231	-----E-----S---Q--	--VS--G-
	Mycobacterium riyadhense	WP_085250614	-----E---------Q--	--VS--A-
	Mycobacterium saskatchewanense	WP_085255899	-----E-----S---Q--	-LLPA---A-----LL--
	Mycobacterium scrofulaceum	WP_067273896	-----EI--------Q--	-LVS-ID-ITF-L-LL--
	Mycobacterium senuense	WP_085084097	---Q--------------	N-VS-----------L--
	Mycobacterium sherrisii	WP_069398297	-----EI--------Q--	-LVS--D-ITF-L-LL--
	Mycobacterium shigaense	BAX94431	-----EI--------Q--	-LVS-IA-VTF-L-LL--
	Mycobacterium shimoidei	WP_069395861	------I-----------	--VS-----V-----L--
	Mycobacterium sinense	WP_064854371	---Q--------------	--VS-----------L--
	Mycobacterium szulgai	WP_068031211	-----E-----S---Q--	-LVPD---A---L-LL--
	Mycobacterium triplex	WP_036471547	-----E---------Q--	-LVS--D-ITF-L-LL--
	Mycobacterium tuberculosis TKK	KBZ59518	-----EI----S---Q--	--VS--D-V---F-LLT-
	Mycobacterium ulcerans Agy99	ABL03067	-----E-----S---Q--	--VS--D-
	Mycobacterium xenopi	WP_085197600	--Y---I-----------	--LS-----------L--
	Mycobacterium yongonense 05-13	AGP62283	-----E---------Q--	-LVS----V---L-LL--
**Other bacteria**	*Blastococcus endophyticus*	SEO49684	---G----T--S--WA--	AMPSP-P-SM--L-C-LI
	Nocardia abscessus	WP_086006762	---EREI-----------	--VS--D--------L--
	Nocardia mexicana	WP_068019259	---ERE------------	-VVS--D--------L--
	Nocardia miyunensis	WP_067668546	---ERE------------	-VVS-----------L--
	Nocardia testacea	WP_039823072	---E-EI-----------	N-VS--D-----L--L--
	Nocardia transvalensis	WP_040752655	---ERE------------	-VVS-----------L--
	Nocardia vaccinii	WP_067887448	----RE------------	-VVS-----------L--
	Pseudonocardia thermophila	SHL07691	---ERE---I-----AL-	TSTKEFP-VT--F--LA-
	Streptacidiphilus anmyonensis	WP_042412807	-F-AR------N-T-TF-	TFPPE-D-LY--V-ALD-
	Streptacidiphilus melanogenes	WP_042376830	-F-AR------N-T-TF-	TFPPE-D-LY--V-ALD-
	Streptacidiphilus neutrinimicus	WP_042364980	-F-AR------N-T-TF-	TFPPE-D-LY--V-ALD-
	Streptacidiphilus oryzae	WP_037575082	-F-AR------N-T-TF-	VFPPE---L---V-LLD-

Supplementary Figure 45

A partial sequence alignment of a conserved region of a cyclase showing a two amino acid insertion that is specific for most members of the *"Fortuitum-Vaccae"* clade and absent in other bacteria.

Group	Species	Accession	49		75
"Fortuitum-Vaccae" Clade (44/46)	*Mycobacterium aromaticivorans*	WP_036344961	DWADGKIARLV	P	NQSSQLGALLDPLVD
	Mycobacterium aurum	WP_087032227	-----------	A	D---R--E----AA-
	Mycobacterium bacteremicum	WP_083060709	-----------	D	----R--E----A--
	Mycobacterium boenickei	WP_077740887	-----------	D	----R----------
	Mycobacterium canariasense	WP_084395164	-----------	D	----R--E----A--
	Mycobacterium chlorophenolicum	KMO82234	----------F	A	----R-------A--
	Mycobacterium chubuense	WP_048417531	----------F	A	----R-------A--
	Mycobacterium cosmeticum	WP_084172359	-----------	D	----R--E----A--
	Mycobacterium diernhoferi	WP_073858255	-----------	A	D---R--E----AA-
	Mycobacterium doricum	WP_085192849	-----------	D	----R--E----F--
	Mycobacterium elephantis	KKW64434	-----------	D	----R--E----A--
	Mycobacterium farcinogenes	CDP81859	-----------	D	----R--E-------
	Mycobacterium flavescens	WP_069416594	-----------	D	----R--E-------
	Mycobacterium fortuitum	WP_065051825	-----------	D	----R--E-------
	Mycobacterium gilvum	WP_013471689	----------F	A	----R--E----A--
	Mycobacterium goodii	WP_049746507	-----------	A	----R--E-------
	Mycobacterium hassiacum	WP_051007450	-----------	D	----R--E-----A-
	Mycobacterium holsaticum	WP_069407938	-----------	D	----R--E----A--
	Mycobacterium houstonense	WP_066897650	-----------	D	----R--E-------
	Mycobacterium iranicum	WP_064285405	----------Y	D	----R--E----A--
	Mycobacterium komanii	CRL71636	-----------	D	----R--E----A--
	Mycobacterium litorale	WP_078019347	-----------	D	----R--E-------
	Mycobacterium llatzerense	WP_043985694	-----------	-	----R--E-------
	Mycobacterium malmesburyense	CRL72838	-----------	D	----R--E----A--
	Mycobacterium moriokaense	WP_083150730	-----------	D	----R--E----F--
	Mycobacterium mucogenicum	WP_064860707	-----------	-	----R--E-------
	Mycobacterium neoaurum	WP_042509741	-----------	D	----P--E-------
	Mycobacterium neworleansense	CRZ14107	-----------	D	----R--E-------
	Mycobacterium obuense	WP_046365299	----------F	D	----R-------A--
	Mycobacterium parafortuitum	WP_083143766	----------F	A	----R--E----A--
	Mycobacterium peregrinum	WP_064887615	-----------	D	----R--E-------
	Mycobacterium phlei	WP_003891214	-----------	D	----R--E-----A-
	Mycobacterium porcinum	WP_069426691	-----------	D	----R----------
	Mycobacterium rhodesiae	WP_014213035	-----------	D	----R--E----A--
	Mycobacterium rufum	KGI68344	----------F	D	----R----------
	Mycobacterium rutilum	WP_083408747	-----------	D	----R--E-------
	Mycobacterium septicum	WP_044518829	-----------	D	----R--E-------
	Mycobacterium setense	WP_039320988	-----------	D	----R--E-------
	Mycobacterium smegmatis	WP_011729233	-----------	A	----R--E-------
	Mycobacterium thermoresistibile	EHI10905	-----------	D	----R--E-------
	Mycobacterium tusciae	WP_083127246	-----------	D	----R--E----A--
	Mycobacterium vaccae	WP_003932238	----------Y	T	----R--E----A--
	Mycobacterium vulneris	WP_065457352	-----------	D	----R----------
	Mycobacterium wolinskyi	WP_085145538	-----------	A	----R--E-------
	Mycobacterium fallax	WP_085094759	----------M		D-Y-R-------A--
	Mycobacterium insubricum	ORA73820	------V---L		--Y-R--E----A--
Other *Mycobacterium* (0/79)	*Mycobacterium abscessus*	WP_074253969	----------M		----H--T----AA-
	Mycobacterium alsense	WP_083138516	---------TL		----R--V----A--
	Mycobacterium angelicum	WP_083111363	----------L		----R--VF---A--
	Mycobacterium arosiense	WP_083065048	----------L		----R--V----A--
	Mycobacterium arupense	WP_046189078	------V---F		--Y-T-------AI-
	Mycobacterium asiaticum	WP_065034730	----------L		D---R--M----A--
	Mycobacterium avium	WP_003876502	----------L		----R--V----A--
	Mycobacterium avium subsp. avium	ETB16298	----------L		----R--V----A--
	Mycobacterium avium subsp. hominissuis	ETB29166	----------L		----R--V----A--
	Mycobacterium avium subsp. paratuberculosis	AAS03852	----------L		----R--V----A--
	Mycobacterium bohemicum	WP_085182383	----------L		D---R--V----A--
	Mycobacterium bovis	WP_047713274	----------L		----R-------A--
	Mycobacterium branderi	WP_083132129	----------L		D---R--E----A--
	Mycobacterium canettii	WP_015290171	----------L		----R-------A--
	Mycobacterium celatum	WP_085167587	----------L		D---R--E----A--
	Mycobacterium chelonae	WP_070919860	----------M		----H--T----AA-
	Mycobacterium colombiense	WP_007771381	----------L		----K--V----A--
	Mycobacterium confluentis	WP_085150854	---------VM		----R--E----A--

Other *Mycobacterium* (0/79)	*Mycobacterium conspicuum*	WP_085231013	---------SL	----R--V----A--
	Mycobacterium engbaekii	WP_085129238	--V---L---L	----A-------AI-
	Mycobacterium europaeum	CQD16068	----------L	D---R--V----A--
	Mycobacterium florentinum	WP_085224268	---------TL	----R--V----A--
	Mycobacterium fragae	WP_085195548	----------L	----K--E----A--
	Mycobacterium franklinii	WP_078336361	----------M	----H--T----AA-
	Mycobacterium gastri	WP_036412725	----------L	----R--V----A--
	Mycobacterium genavense	WP_025737086	---------TL	----R--V----A--
	Mycobacterium gordonae	WP_065049647	----------L	--Q-R--M----A--
	Mycobacterium haemophilum	WP_047314191	----------L	D---R--V----A--
	Mycobacterium heckeshornense	WP_048892498	----------L	--A-R--E----A--
	Mycobacterium heidelbergense	WP_083075879	---------ML	D---R--V----A--
	Mycobacterium heraklionense	WP_064887474	------L---L	----A-------AI-
	Mycobacterium hiberniae	WP_085136837	--V---L---L	----A-------AI-
	Mycobacterium icosiumassiliensis	WP_067969257	------L---L	----A-------AI-
	Mycobacterium immunogenum	WP_043078987	----------M	----H--T----AA-
	Mycobacterium indicus pranii	WP_014942125	----------L	----R--V----A--
	Mycobacterium interjectum	WP_066912522	---------TL	----R--V----A--
	Mycobacterium interjectum	WP_085203029	---------TL	----R--V----A--
	Mycobacterium intermedium	WP_069418403	----------L	D---K--T----A--
	Mycobacterium intracellulare	WP_064938456	----------L	----R--V----A--
	Mycobacterium kansasii	WP_063469889	----------L	----R--V----A--
	Mycobacterium kyorinense	WP_065012864	----------L	D---K--E----A--
	Mycobacterium lacus	WP_085158728	---------TL	----R--V----A--
	Mycobacterium lentiflavum	CQD14176	---------TL	----R--V----A--
	Mycobacterium leprae	WP_010908707	----------L	D-Y-R--V----A--
	Mycobacterium lepromatosis	WP_045843584	----------L	D-Y-R--M----A--
	Mycobacterium longobardum	WP_085265370	--V---L---L	D---A-------AI-
	Mycobacterium malmoense	WP_065444708	----------L	D---R--V----A--
	Mycobacterium mantenii	WP_083096512	----------L	D---R--V----A--
	Mycobacterium marinum	WP_012394413	----------L	----R--V----A--
	Mycobacterium marseillense	WP_083019650	----------L	----R--V----A--
	Mycobacterium minnesotense	WP_083022946	------V---F	--Y-K-------AI-
	Mycobacterium nebraskense	WP_046184267	----------L	----R--V----A--
	Mycobacterium nonchromogenicum	WP_085139849	------L---L	D---A-------AI-
	Mycobacterium noviomagense	WP_083087322	----------L	--A-R--E----A--
	Mycobacterium palustre	WP_085077118	---------SL	----R--V----A--
	Mycobacterium paraense	WP_085100694	---------TL	----R--V----A--
	Mycobacterium paraffinicum	WP_073874228	----------L	D---R--V----A--
	Mycobacterium paraseoulense	WP_083174331	----------L	----R--V----A--
	Mycobacterium parmense	WP_085267474	---------TL	----R--V----A--
	Mycobacterium riyadhense	WP_085249348	----------L	----R--V----A--
	Mycobacterium salmoniphilum	WP_078327286	----------M	----H-------AA-
	Mycobacterium saopaulense	WP_070912520	----------M	----H--T----AA-
	Mycobacterium saskatchewanense	WP_085256696	----------L	D---R--V----A--
	Mycobacterium scrofulaceum	WP_067272527	----------L	----R--V----A--
	Mycobacterium senuense	WP_085084005	------L---L	D---A-------AI-
	Mycobacterium sherrisii	WP_069401775	---------TL	----R--V----A--
	Mycobacterium shigaense	BAX92732	---------SL	----R--V----A--
	Mycobacterium shimoidei	WP_069397599	----------L	----KF-E----A--
	Mycobacterium shinjukuense	WP_083051005	----------L	----R--V----A--
	Mycobacterium simiae	WP_061555637	---------TL	----R--V----A--
	Mycobacterium sinense	WP_065025421	------L---L	D---A-------AI-
	Mycobacterium szulgai	WP_085669223	----------L	----R--VF---A--
	Mycobacterium terrae	WP_085259460	------L---L	D---A-------AI-
	Mycobacterium triplex	WP_036469123	---------TL	----R--V----A--
	Mycobacterium triviale	WP_085110989	--L---L---L	G-Y-R--E----A--
	Mycobacterium tuberculosis	AOE36186	----------L	----R-------A--
	Mycobacterium ulcerans	WP_011740903	----------L	----R--V----A--
	Mycobacterium vulneris	WP_085289695	----------L	----R--V----A--
Other *Corynebacteriales*	*Mycobacterium xenopi*	WP_003921788	----------L	--A-R--E----A--
	Corynebacterium auriscanis	KGM19408	-QL--FL--KY	QVITDF-K-A--IA-
	Dietzia cinnamea	WP_082904448	--L-------W	-MR-TW-ER---IA-
	Gordonia araii	WP_007321509	--L---L---L	D---SI-------A-
	Gordonia neofelifaecis	WP_009680907	--L---L--WL	G---K-------AA-

**Other *Corynebacteriales***	*Nocardia uniformis*	WP_067523310	-FL---L---L	D-A-R---I------
	Nocardia vaccinii	WP_067894452	-YL---L---L	D-Y-R-------F--
	Segniliparus rotundus	WP_013139174	------L---L	D---K-------I--
	Segniliparus rugosus	WP_007467016	------L---L	D---K-------V--
	Skermania piniformis	WP_066473858	--L---L--WL	D-M-R----------
	Smaragdicoccus niigatensis	WP_040631908	-FL---L---M	----K---V---M--
	Tomitella biformata	WP_024796293	--L---L--WL	--M-RF------IA-
	Tsukamurella paurometabola	WP_013126970	--L---L---L	--QTR--E----AA-
	Tsukamurella tyrosinosolvens	KXP06381	--L---L---L	D-QTR--E----AA-
	Williamsia herbipolensis	WP_045822898	------L---L	----R-------AA-
	Williamsia sterculiae	SIS19239	------L---L	----KI------AA-

Supplementary Figure 46

A partial sequence alignment of a conserved region of CDP-diacylglycerol--glycerol-3-phosphate 3-phosphatidyltransferase showing a one amino acid insertion that is specific for most members of the *"Fortuitum-Vaccae"* clade and absent in other *Corynebacteriales*.

Clade	Species	Accession	112		160
"Fortuitum-Vaccae" Clade (41/42)	*Mycobacterium acapulcensis*	WP_066811333	CIVLRLARFNAMLDVD		KPDYEKKYFVGMPAPAGAIGAIGPLAA
	Mycobacterium aurum	WP_048633130	--------Y----S--		Q--------T-L--------------
	Mycobacterium austroafricanum	WP_036367720	----------------		Q-A------T-----------------
	Mycobacterium brisbanense	WP_062828536	-----------L----		Q-A---E-------------T-V----
	Mycobacterium canariasense	WP_062658575	--------------A-		L-A---E--M--------------I-
	Mycobacterium chubuense	WP_014813836	--------------L-		Q-A-Q----------------------
	Mycobacterium conceptionense	CQD09591	-----------L----		Q-A---E----------------L---
	Mycobacterium confluentis	WP_085155923	--------Y----AG-		Q-E---QF-T-----------------
	Mycobacterium doricum	WP_085189606	-------------TE-		Q-A-T-EF-------------------
	Mycobacterium fallax	WP_085096077	-----------R-HG-		G-A--QQF-T-----------------
	Mycobacterium flavescens	WP_069414969	----------------		---------------------------
	Mycobacterium gilvum	WP_011891156	-------------A--		Q-A------T-----------------
	Mycobacterium goodii	WP_049743090	-----------L----		Q-A---E--------------------
	Mycobacterium hassiacum	WP_005624476	---------------E		R-A---Q--------------------
	Mycobacterium holsaticum	WP_069406309	--------Y----A--		Q-AF-----------------------
	Mycobacterium houstonense	WP_066902674	-----------L----		Q-A---E----------------L---
	Mycobacterium insubricum	ORA60788	-----------R-HG-		--A--QQF-T-----------------
	Mycobacterium iranicum	WP_036464870	--------Y----S--		Q-A------T-----------------
	Mycobacterium komanii	CRL69362	----------------		---------------------------
	Mycobacterium litorale	WP_078017758	--------Y--L--A-		L-A-T-E--------------------
	Mycobacterium mageritense	WP_085980439	-----------L----		Q-A---E----------------L---
	Mycobacterium malmesburyense	CRL66605	----------------		---------------------------
	Mycobacterium moriokaense	WP_083156383	---------------A		--A---E--------------------
	Mycobacterium mucogenicum	WP_064858684	---------------E		G-AF--EF-T-----------------
	Mycobacterium neworleansense	CRZ16396	-----------L----		Q-A---E----------------L---
	Mycobacterium obuense	KMO76169	--------------L-		Q------F-----------V--A----
	Mycobacterium parafortuitum	WP_083142205	-------------S--		Q-A------T-----------------
	Mycobacterium peregrinum	WP_064880630	-----------L----		Q-A---E-----------L--------
	Mycobacterium phlei	WP_003887071	----------------		R-A---Q--------------------
	Mycobacterium porcinum	WP_075923410	-----------L----		Q-A---E----------------L---
	Mycobacterium rhodesiae	WP_050899809	---------------V		-----N---------------------
	Mycobacterium rufum	KGI66629	--------Y-----L-		Q-A----F-------------------
	Mycobacterium rutilum	WP_083405898	----------------		---------------------------
	Mycobacterium septicum	WP_044515571	-----------L----		Q-A---E-----------L--------
	Mycobacterium setense	WP_039314268	-----------L----		Q-A---E----------------L---
	Mycobacterium smegmatis	WP_003892285	-----------L----		Q-A---E--------------------
	Mycobacterium thermoresistibile	WP_003926617	-----------L----		Q-E-A-EF-T-----------------
	Mycobacterium tusciae	WP_083124294	---------------A		--A--S---------------------
	Mycobacterium vaccae	WP_003932830	----------------		Q-A------T-L---------------
	Mycobacterium vanbaalenii	WP_011778024	---------------E		Q-A------T-----------------
	Mycobacterium wolinskyi	WP_067849359	-----------L----		Q-A---E--------------------
	Mycobacterium aromaticivorans	WP_051660406	-----------L--D-	T	R-A-TRE--T-----C--V-V------
Other *Mycobacterium* (0/79)	*Mycobacterium abscessus*	WP_070410678	----------TL--D-	T	A-AFT-E----V-S----LMVLA----
	Mycobacterium abscessus subsp. bolletii	EHM23174	----------TL--D-	T	A-AFT-E----V-S----LMVLA----
	Mycobacterium africanum	WP_003910123	-V------Y--LQ-DG	T	Q-A-AHEF----------VSM--L--
	Mycobacterium alsense	WP_0831379	-V------Y--LQ-DG	T	Q-A-AHEF----------VSM--
	Mycobacterium angelicum	WP_083116487	-V------Y--QQ-DG	T	L-P-A-EF----------VSM--
	Mycobacterium arosiense	WP_083066477	-V---------LQ-DG	S	Q-S-AHEF-----------SM--
	Mycobacterium asiaticum	WP_065034501	-V------Y--QQ-DG	T	L-PFA-E-----------VSM--TI-
	Mycobacterium aurum	WP_087030588	----------SL--DA	T	Q-A-TRQ--T-----C----V------
	Mycobacterium avium	WP_084023384	-V---------L--DG	S	Q-A-THEF----------VSM--
	Mycobacterium bacteremicum	WP_083060306	-----------L--D-	T	--A-TRQ--T-----C--V-V------
	Mycobacterium bohemicum	WP_085179408	-V------Y--LQ-DG	T	Q-S-AHEF----------VSM--
	Mycobacterium bovis BCG	AMC48994	-V------Y--LQ-DG	T	Q-A-AHEF----------VSM--L--
	Mycobacterium branderi	WP_083134246	-----------L--DA	T	L-A-TREF-----------AVL-----
	Mycobacterium canettii	WP_015289037	-V------Y--LQ-DG	T	Q-A-AHEF----------VSM--L--
	Mycobacterium celatum	WP_062540981	-----------L--DA	T	L-A-TREF-----------AVL-----
	Mycobacterium chelonae	WP_070916706	----------TL--D-	S	Q-AFT-E----V-S----LMVLA----
	Mycobacterium colombiense	WP_064881639	-V---------LQ-DG	G	Q-S-AHEF-----------SM--
	Mycobacterium conspicuum	WP_085235662	-V------Y--QH-D-	T	Q-S-A-EF----------VSM--
	Mycobacterium diernhoferi	WP_073859619	-----------L--D-	T	--A-TRQ--T-----C----V------
	Mycobacterium engbaekii	WP_085126326	-V--------TL--DA	T	Q-A-TREF-----------TLM-L---
	Mycobacterium europaeum	WP_085240770	-V------Y--LQADG	S	Q-A-THEF-----------SM--
	Mycobacterium florentinum	WP_085222719	-V---------LQ-DG	S	R-A-AHEF-----------SM--

	Mycobacterium fragae	WP_085197750	----------L--DA T L-S-TREF-----------S-L-----
	Mycobacterium franklinii	WP_070937187	----------TL--D- S A-AFT-E----V-S----LMVLA----
	Mycobacterium gastri	WP_036413940	-V------Y--Q--DG T L-P-A-EF----------VSM--
	Mycobacterium genavense	WP_025736989	-V---------LQ-DG T Q-A-AREF-----T-----SM--
	Mycobacterium gordonae	WP_065044959	-V------Y--QQ-DG T L-P-A-E-----------VSM--TIG
	Mycobacterium haemophilum	WP_047313699	-V---------L--DG T Q-A-AREF----------VSV--L--
	Mycobacterium heckeshornense	OIZ79573	S----------L--DA T L-A-TREF-----------AVL-----
	Mycobacterium heidelbergense	WP_083074228	-V------Y-TLQ-DG T Q-A-AHEF----------VSM--
	Mycobacterium heraklionense	WP_065041503	-V--------TL--DA T L-A-TREF-----------TLM-L---
	Mycobacterium hiberniae	WP_085136469	-V--------TL--DA T Q-A-TREF-----------TLM-L---
	Mycobacterium icosiumassiliensis	WP_067966528	-V--------TL--DA T L-A-TREF-----------TLM-L---
	Mycobacterium immunogenum	WP_043080216	----------TL--D- T A-AFT-E----V-S----LMVLA----
	Mycobacterium interjectum	WP_085202872	-V------Y--LA-DG A Q-A-AHDF----------VSM--
	Mycobacterium intermedium	WP_069421977	-V------Y--A--DG T L-P-A-EF----------VSM--TI-
	Mycobacterium intracellulare	WP_064934932	-V--------SL--DG S Q-A-THEF-----------SM--
	Mycobacterium kansasii	WP_063472040	-V------Y--Q--DG T L-P-A-EF----------VSM--
	Mycobacterium kumamotonense	WP_065289171	-V--------TL--DA T L-A-TREF-----------TLM-L---
	Mycobacterium kyorinense	WP_065014227	-----------L--DA T L-A-TREF-----------ATL-----
	Mycobacterium lacus	WP_085157920	-V------Y--LQ-DG T Q-A-AREF----------VSM--L--
	Mycobacterium lentiflavum	CQD05250	-V---------LQ-DG S --A-AHEF-----------SM--
	Mycobacterium leprae	WP_010907664	-V---------L--DG T Q-A-TREF----------VSV--L--
	Mycobacterium lepromatosis	KJX75738	-V---------L--DG P Q-A-AREF----------VSL--L--
	Mycobacterium liflandii	WP_015354416	-V---------L--DG T Q-A-AHEF----------VSM--L--
	Mycobacterium longobardum	WP_085264823	-V---------L--DA T L-A-TREF-----------TLM-L---
	Mycobacterium malmoense	WP_065445673	-V------Y--L-EDG S Q-A-THEF-----------SM--
	Mycobacterium mantenii	WP_083094980	-V---------LQ-DG S Q-S-AHEF-----------SM--
	Mycobacterium marinum M	ACC39213	-V---------L--DG T Q-A-AHEF----------VSM--L--
	Mycobacterium marseillense	WP_083019470	-V---------LQ-DG S Q-A-THEF-----------SM--
**Other *Mycobacterium* (0/79)**	*Mycobacterium minnesotense*	WP_083022628	-V--------TL--DA T L-A-TREF-----------TLM-L---
	Mycobacterium nebraskense	WP_046187269	-V------Y--LQ-DG S Q-A-THEF----------VSM--
	Mycobacterium neoaurum	CDQ42664	-----------L--DT S --A-TRQ--T-----C--V-V------
	Mycobacterium noviomagense	WP_083087818	----------L--DA T L-A-TREF-----------AVL-----
	Mycobacterium palustre	WP_085077505	-V------Y--LA-DG T Q-G-AHEF----------VSM--
	Mycobacterium paraense	WP_085092901	-V------Y--LA-DG N Q-A-AHEF----------VSM--
	Mycobacterium paraffinicum	WP_073874596	-V------Y--LQ-DG S Q-A-THEF----------VSM--
	Mycobacterium paraseoulense	WP_083175611	-V------Y--LQADG S Q-A-THEF----------VSM--
	Mycobacterium parmense	WP_085271748	-V------Y--L--DG T Q-A-THEF----------VSM--
	Mycobacterium rhodesiae	WP_083118378	-----------L--D- T R-A-TRE--T-----C--V-V------
	Mycobacterium riyadhense	WP_085248235	-VI-----Y--QQ-DL T Q-A-A-EF----------VSM--
	Mycobacterium salmoniphilum	WP_078331242	----------TL--D- S Q-AFT-E----V-S----LMVLA----
	Mycobacterium saopaulense	WP_070913430	----------TL--D- T A-AFT-E----V-S----LMVLA----
	Mycobacterium saskatchewanense	WP_085258761	-V---------LQ-DG R Q-A-THEF----------VSM--
	Mycobacterium scrofulaceum	WP_067282099	-V------Y--LQSDG S Q-A-THEF----------VSM--
	Mycobacterium sherrisii	WP_069400667	-V---------LQ-DG T Q-A-AREF----------VSM--
	Mycobacterium shigaense	BAX90813	-V---------LQ-DG S Q-AWTHEF-A--------VSM--
	Mycobacterium shimoidei	WP_069397423	-----------L--DA T L-P--REF-----------A-L-----
	Mycobacterium shinjukuense	WP_083046593	-V------Y--LQ-DG T Q-A-AREF----------VSM--L--
	Mycobacterium simiae	WP_044509640	-V---------LQ-DG T Q-A-AREF----------VSM--
	Mycobacterium sinense	WP_064854980	-V---------L--DA T L-A-TREF-----------TLM-L---
	Mycobacterium szulgai	WP_085670556	-V------Y--QQ-DG T L-P-A-EF----------VSM--
	Mycobacterium terrae	WP_085260792	-V--------TL--DA T L-S-TREF-----------TLM-L---
	Mycobacterium triplex	WP_036466456	-V---------LQ-DG T R-A-AREF-----------SM--
	Mycobacterium triviale	WP_085109094	-----------L--DT A Q-A-TRE-----------LAV------
	Mycobacterium tuberculosis	WP_070900687	-VA-----Y--LQ-DG T Q-A-AHEF----------VSM--L--
	Mycobacterium ulcerans	WP_011739551	-V---------L--DG T Q-A-AHEF----------VSM--L--
	Mycobacterium vulneris	WP_085288138	-V---------LQ-DG S Q-S-AHEF-----------SM--
	Mycobacterium xenopi	WP_081485468	----------L--DA T L-A-TREF-----------AVL-----
	Gordonia aichiensis	WP_052003932	A---------TL--DT D A-G-TRDF---V----A-LV-LL-IGL
	Gordonia hirsuta	WP_005938273	A---------TL--D- S A-G-TRDF---V----A-VMVLL-IGL
	Gordonia polyisoprenivorans	WP_006372436	A---------TL--D- D A-A-TRDF---V----A--I-LL-VGL
**Other *Corynebacteriales***	*Gordonia sputi NBRC 100414*	GAB39806	A---------TL--DT D A-G-TRDF---V----A-LVVLL-IGL
	Hoyosella altamirensis	WP_083962397	S---------TLI-DT E A-A-H-D----V-----GLI-LA----
	Hoyosella subflava	WP_013805368	S---------TLI-DT E A-A-H-D----V-----GLI-LA----
	Millisia brevis	WP_066908119	----------TLASDS T R-P-AGDF---V----A-LA-LA--V-
	Nocardia abscessus	WP_051169382	S---------TLM-D- T R--WARE----V------LI-MV-V-L

Other *Corynebacteriales*

Nocardia caishijiensis	WP_067979086	SL--------TL--D- S R-NWARE--T-V------LI-LV-I-L
Nocardia jejuensis	WP_067704334	-M--------TLM-D- N V--WQRE----V------LIVLL-I-L
Nocardia soli	WP_071930669	SL--------TL--D- S R-NWARE----V------LI-LV-I-L
Rhodococcus defluvii	WP_031939599	S---------TL--D- T V-A-T-DF---V------LI-LT----
Rhodococcus erythropolis	WP_060939318	S---------TL--D- D V-A-ARE----V------LI-LT----
Rhodococcus fascians	WP_032380268	S---------TL--E- D T-A-ARE--T-V------LI-LA-I--
Rhodococcus ruber	WP_040270751	S---------TL-ED- T V-A-ARE----V-S----LV-LA-V--
Segniliparus rotundus	WP_013137343	-A--------TLT-N- D A-P-A-E----V-T----LT-CA-I--
Segniliparus rugosus	WP_051357265	-A--------TLT-ES D A-A-A-E----V-T----LT-CA-I--
Skermania piniformis	WP_066466868	S----------LM-D- N R-SWARE----V------LI-LL-IVL
Smaragdicoccus niigatensis	WP_018158681	---------TLM-DA N Q-S-T-D----V------LMTLV-VV-
Tomitella biformata	WP_084611741	S---------TL--D- T A-A-T-E--T-V-S----LL-LA-V--
Tsukamurella paurometabola	WP_013128284	-------TLAADP T A-A-T-DF---V------MLVLA----
Tsukamurella pseudospumae	WP_068571494	AM--------TLAADP T A-A-T-DF---V---
Tsukamurella pulmonis	SDQ55873	AM--------TLAADP T A-A-T-DF---V---
Tsukamurella tyrosinosolvens	SED43121	AM--------TLAADP T A-A-T-DF---V---
Williamsia herbipolensis	WP_082079849	AV--------IID-D- D V-E-T-N----
Williamsia muralis	WP_062795204	A---------TLI-E- D T-Q-T-DF---V----A-LI-ML-I-L
Williamsia sterculiae	WP_083709522	A---------ILA-DE D Q-EWS-GF---V----T--I-LA-V-G

Supplementary Figure 47

A partial sequence alignment of a conserved region of CDP-diacylglycerol--serine O-phosphatidyltransferase showing a one amino acid deletion that is specific for most members of the *"Fortuitum-Vaccae"* clade and absent in other *Corynebacteriales*.

Group	Species	Accession	123		155
Mycobacterium Slow-Growers (61/62)	Mycobacterium angelicum	WP_083113621	AAAALALYRTHRG	G	DRPVVAVIYTHSHVDHFGG
	Mycobacterium alsense	WP_083139150	--A---	-	-------------------
	Mycobacterium asiaticum	OBI98597	-R---E-------	-	-------------------
	Mycobacterium avium	WP_062888026	-----T-------	-	-------------------
	Mycobacterium avium subsp. avium	EUA29728	--------HE---	-	-------------------
	Mycobacterium avium subsp. hominissuis	ETB26180	-----T-------	-	-------------------
	Mycobacterium avium subsp. paratuberculosis	ETB47572	-----T-------	-	-------------------
	Mycobacterium bohemicum	WP_085183349	-----S-------	-	E------------------
	Mycobacterium bovis	WP_024458162	-----D---A---	A	-------------------
	Mycobacterium canettii	WP_014001936	-----D---A---	A	-------------------
	Mycobacterium chimaera	KPN55853	---------E---	-	-------------A-----
	Mycobacterium colombiense	WP_064880184	----M--------	-	E------------------
	Mycobacterium conspicuum	WP_085230936	---S-Q-------	-	-------------------
	Mycobacterium florentinum	ORV53115	-----D-------	A	--A----------------
	Mycobacterium gastri	WP_036418839	-----D-------	-	-------------------
	Mycobacterium gordonae	OBK43639	-----E---A---	-	E------------------
	Mycobacterium haemophilum	WP_054879142	-----D-------	-	-------------------
	Mycobacterium heidelbergense	WP_083076182	-----D---A---	-	-------------------
	Mycobacterium indicus pranii	WP_014941144	---------E---	-	A------------------
	Mycobacterium interjectum	WP_066916889	-------------	-	-------------------
	Mycobacterium intermedium	WP_069420132	---------E---	-	-------------------
	Mycobacterium intracellulare	WP_064934137	---------E---	-	-------------------
	Mycobacterium kansasii	WP_063473086	-----D---A---	-	-------------------
	Mycobacterium kubicae	WP_085074945	-------------	-	-------------------
	Mycobacterium lacus	WP_085162468	-----G-------	-	--R----------------
	Mycobacterium lentiflavum	WP_090607835	-----D-------	A	K------------------
	Mycobacterium liflandii	WP_015357569	-----T---A--D	A	-------------------
	Mycobacterium malmoense	WP_065444852	---------E---	-	-------------------
	Mycobacterium mantenii	WP_083097304	-------------	-	--R----------------
	Mycobacterium marinum	WP_012396819	-----T---A--D	A	-------------------
	Mycobacterium marseillense	WP_083016814	---------D---	-	-------------------
	Mycobacterium nebraskense	WP_046185293	--A---	-	--R----------------
	Mycobacterium palustre	WP_085078928	--A---	-	E------------------
	Mycobacterium paraense	WP_085095131	--A---	-	-------------------
	Mycobacterium paraffinicum	WP_073874906	-----G-------	-	-------------------
	Mycobacterium paraseoulense	WP_083171772	--A---	-	-------------------
	Mycobacterium paraintracellulare	WP_014383652	---------E---	-	A------------------
	Mycobacterium parascrofulaceum ATCC BAA-	EFG77352	---------E---	-	-------------------
	Mycobacterium parmense	WP_085270865	-------------	-	E------------------
	Mycobacterium riyadhense	WP_085252513	-----T-------	-	-------------------
	Mycobacterium scrofulaceum	WP_067268624	-G---G-------	-	-------------------
	Mycobacterium sherrisii	WP_069400052	-----G---A---	-	E------------------
	Mycobacterium shigaense	BAX94922	-----D---A---	-	-------------------
	Mycobacterium shinjukuense	WP_083046780	-----E---A---	-	-------------------
	Mycobacterium simiae	WP_061557562	-----D-------	-	-------------------
	Mycobacterium szulgai	WP_085674191	-------------	-	-------------------
	Mycobacterium timonense	WP_083186977	-----T-------	-	-------------------
	Mycobacterium triplex	CDO85894	-----D-------	A	-------------------
	Mycobacterium tuberculosis	SHA51133	-----D---A---	A	-------------------
	Mycobacterium ulcerans	WP_011741988	-----T---A--D	A	-------------------
	Mycobacterium yongonense	OCB23425	---------E---	-	-------------A-----
	Mycobacterium engbaekii	WP_085129383	--A---	-	----T--------I-----
	Mycobacterium europaeum	WP_085242518	--A--T	-	E------------------
	Mycobacterium heraklionense	WP_064889695	-----K---E---	-	----T--------A-----
	Mycobacterium hiberniae	WP_085136228	--A---	-	----T--------I-----
	Mycobacterium icosiumassiliensis	WP_067976736	-----K---E---	-	--Q-T--------A-----
	Mycobacterium kumamotonense	WP_065289259	--R---	-	E---T--------------
	Mycobacterium longobardum	WP_085265552	---------E---	-	----T--------------
	Mycobacterium nonchromogenicum	WP_085139939	-----K---E---	-	----T--------------
	Mycobacterium sinense	WP_065024439	---------E---	-	Q---T--------------
	Mycobacterium terrae	WP_085259979	-----G---A---	-	----T--------------
	Mycobacterium arupense	WP_046189513	-----K---A---		--A-T--------------
Other *Mycobacterium* (0/21)	Mycobacterium boenickei	WP_077740624	----FG---E---		-------------G-----
	Mycobacterium conceptionense	WP_064896052	----FG---E---		-------------G-----

Other *Mycobacterium* (0/21)	Mycobacterium farcinogenes	WP_036391098	----FG---E---	-------------G-----
	Mycobacterium flavescens	WP_069416672	-----G---A---	-------------------
	Mycobacterium fortuitum	WP_061264312	----IG---E---	-------------G-----
	Mycobacterium fragae	WP_085197091	-----T---SR--	-------------------
	Mycobacterium hassiacum	WP_005632288	-----G---A---	----R--------------
	Mycobacterium iranicum	WP_085176429	---------Q---	----R--------------
	Mycobacterium kyorinense	WP_065015741	-----T-------	--R----------------
	Mycobacterium mageritense	WP_036431482	-----E---A---	-------------A-----
	Mycobacterium neworleansense	WP_090517083	-S--FG---E---	-------------G-----
	Mycobacterium obuense	WP_046362977	---------E---	E---T--------------
	Mycobacterium peregrinum	WP_064957319	----FG---E---	-------------G-----
	Mycobacterium phlei	WP_061481018	-----Q-------	----T--------------
	Mycobacterium porcinum	WP_075922404	-----G---E---	-------------G-----
	Mycobacterium rufum	KGI70237	---------Q---	--A-T--------------
	Mycobacterium rutilum	WP_083406817	-----G---A---	-------------------
	Mycobacterium salmoniphilum	WP_078325211	---G-----S---	----TGL------------
	Mycobacterium shimoidei	ODR12089	-S---S-------	Q------------L-----
	Mycobacterium vulneris	WP_065459628	----FG---E---	-------------G-----
	Mycobacterium wolinskyi	WP_067851541	-----K---E---	-------------G-----
Other bacteria	Achromobacter arsenitoxydans	WP_008163479	-K---D--YQ--P	KK--------------Y--
	Bordetella bronchiseptica	WP_003808624	-K---D--YQ--P	RK--------------Y--
	Buttiauxella gaviniae	WP_064516665	-K-G-E--YKN--	KK------F-------Y--
	Corynebacterium variabile	WP_030198805	-----G---E---	-------V-----------
	Desulfovibrio aespoeensis	WP_041271878	-R---E--YK---	R--------S------Y--
	Enterobacter hormaechei	WP_063412955	-K-GMD--VQ---	K---------------Y--
	Escherichia albertii	WP_059217735	-KVGME--YKN--	KK--------------Y--
	Frankia elaeagni	WP_018636153	-R---E---SQ--	K---T--------A-----
	Gordonia neofelifaecis	WP_009677333	-----K---E---	--T--------C-------
	Herbidospora cretacea	WP_061299309	-E---R---G---	----TG----------Y--
	Klebsiella oxytoca	WP_064344273	-RVGMD--YKN--	NK--------------Y--
	Legionella drancourtii	WP_006869866	-K---E--FKY-P	QK-I------------Y--
	Macrophomina phaseolina MS6	EKG18249	-K---E--QA---	V---K-M--S---------
	Microbacterium mangrovi	WP_039396214	----V----E---	----T---F----I-----
	Millisia brevis	WP_066908449	---------Q---	--A--S-------------
	Nocardia puris	WP_067507202	-----D---R---	----TGL------A-----
	Nocardiopsis listeri	WP_067609245	-R-G-E---SQ--	----SG-L-S---------
	Penicillium brasilianum	OOQ84068	-----E---K---	--K-T----S---I-----
	Pseudomonas knackmussii	WP_043257193	-K---D--FK--P	KK--------------Y--
	Pseudonocardia acaciae	WP_028923840	---D---R---	----K--V-----A-----
	Rhodococcus koreensis	WP_072938114	---------KN--	----TG-------I-----
	Rhodopseudomonas palustris	WP_011156543	-R--MQ--AR---	--------F--T-A--W--
	Saccharopolyspora antimicrobica	SFO82802	---G-----A---	----T--------------
	Salmonella enterica subsp. enterica sero	KGF83939	-K-GMD--FKN--	NK----I---------Y--
	Streptomyces pluripotens	WP_043435333	---G-----S---	----T--L-----A-----
	Tetrasphaera japonica	WP_048550684	-E---E---E---	-----------C-------
	Thalassospira profundimaris	WP_064788542	-K-G-E---A---	-K--LG--H----I-----
	Thermoactinospora rubra	WP_084964359	---------E---	----T--------A-----
	Thiolapillus brandeum	WP_041068169	-K---E--YK--P	KK--K--------------
	Trabulsiella odontotermitis	WP_049857287	-KVGMD--FK---	KK--------------Y--
	Williamsia muralis	WP_062795730	---G-S---SQ--	----T--------I-----

Supplementary Figure 48

Detailed sequence information for the one amino acid insertion found in alkyl/aryl-sulfatase, which is shown in Figure 7. This insertion is specific for most members of the *Mycobacterium* Slow-Growers and absent in other bacteria.

Mycobacterium Slow-Growers except *M. koreense* and *M. triviale* (67/69)

		30		68
Mycobacterium alsense	WP_083139296	IARFNPEQPEA	FAKT	GGWQSFRVPCLPSDRVLNLLIYIK
Mycobacterium angelicum	WP_083116204	------DD-D-	--A-	------------T--L--------
Mycobacterium arosiense	WP_083066380	------D--D-	Y---	---------------M--------
Mycobacterium asiaticum	WP_065033735	------DD-D-	--A-	---------------L--------
Mycobacterium avium	WP_062894660	------D--D-	Y-E-	---------------M--------
Mycobacterium avium subsp. avium	EUA28323	------D--D-	Y-E-	---------------M--------
Mycobacterium bohemicum	WP_085182137	-------D-D-	----	---------------L--------
Mycobacterium bovis	WP_012666361	------DE-D-	--A-	---------------L--------
Mycobacterium colombiense	WP_064878680	------D--D-	--E-	---------------M--------
Mycobacterium conspicuum	WP_085231540	------D--D-	Y-E-	---------------L--------
Mycobacterium doricum	WP_085187113	-------D-D-	YSDS	--F---------T--L----H-V-
Mycobacterium europaeum	WP_085241733	------D--D-	--E-	---------------L--------
Mycobacterium florentinum	WP_085219950	------DD-D-	----	---------------L--------
Mycobacterium fragae	WP_085198539	-------N-DQ	Y-DS	---------------L--------
Mycobacterium gastri	WP_036410071	------DD-DR	--E-	---------------L--------
Mycobacterium genavense	WP_025738483	------DD-D-	----	---------------L--------
Mycobacterium gordonae	WP_065046289	------DD-DR	--A-	---------------L----L-V-
Mycobacterium haemophilum	WP_047313390	------DD-D-	--E-	----T----------L--------
Mycobacterium heidelbergense	WP_083076339	------D--D-	--E-	------------------------
Mycobacterium icosiumassiliensis	WP_067971079	---Y---N-D-	Y-A-	D--------A--T--L------V-
Mycobacterium interjectum	WP_066907964	----D-D----	----	---------------M--------
Mycobacterium intermedium	WP_069420419	---Y--DD--R	--A-	------------T--L----L---
Mycobacterium intracellulare	WP_064893736	------D--D-	Y-E-	---------------M--------
Mycobacterium kansasii	WP_063467586	------DD--R	--A-	---------------LI-------
Mycobacterium komanii	CRL75534	-------N-D-	Y-DS	------------T--L----H-V-
Mycobacterium kubicae	WP_085072758	---------D-	-SD-	---------------L--------
Mycobacterium lacus	WP_085162480	---Y--DD-DR	Y-E-	---------------L--------
Mycobacterium lentiflavum	CQD07600	------DD-D-	----	---------------L--------
Mycobacterium leprae	WP_010907879	------DN-D-	--E-	------------T--L--------
Mycobacterium lepromatosis	WP_045842650	-------N-D-	--E-	----N-------T--L--------
Mycobacterium liflandii	WP_015354788	------DA---	--S-	---------------L--------
Mycobacterium malmesburyense	CRL78517	---------D-	Y-DS	---------S--T--L----H-V-
Mycobacterium malmoense	WP_065444826	------D--D-	--E-	---------------L--------
Mycobacterium mantenii	WP_083098808	------D--D-	H-E-	---------------M--------
Mycobacterium marinum	WP_012393080	------DD---	--S-	---------------L--------
Mycobacterium marseillense	WP_083020406	------D--D-	Y-E-	---------------M--------
Mycobacterium nebraskense	WP_046186123	------DN-D-	--E-	---------------L--------
Mycobacterium palustre	WP_085080278	------D--D-	-SE-	---------------M--------
Mycobacterium paraense	WP_085096203	------D----	----	---------------M--------
Mycobacterium paraffinicum	WP_073878079	------DN-D-	--E-	---------------L--------
Mycobacterium paraseoulense	WP_083169758	------DD-D-	----	A--------------L--------
Mycobacterium parmense	WP_085270609	------DD-D-	----	---------------L--------
Mycobacterium riyadhense	WP_085251057	------DD-D-	--D-	---------------L--------
Mycobacterium saskatchewanense	WP_085253651	------D--D-	--Q-	---------------L------V-
Mycobacterium scrofulaceum	WP_067280664	------DN-D-	--E-	---------------L--------
Mycobacterium sherrisii	WP_069399050	------DD-D-	--E-	---------------L--------
Mycobacterium shimoidei	WP_069395278	-------N-DQ	Y-DS	---------------L--------
Mycobacterium shinjukuense	WP_083048715	---Y--DD---	--A-	------W-----T--L--------
Mycobacterium simiae	WP_061558460	------DD-D-	--E-	---------------L--------
Mycobacterium szulgai	WP_068033624	---------D-	-SD-	---------------L--------
Mycobacterium triplex	WP_036467110	------DD-D-	----	---------------L--------
Mycobacterium tuberculosis	WP_070898135	------DD-D-	--A-	---------------L--------
Mycobacterium tusciae	WP_083124933	-------D-DT	--DS	-----Y---------L----H-V-
Mycobacterium ulcerans	WP_071498037	---Y--DD---	--S-	---------------L--------
Mycobacterium vulneris	WP_085290246	------D--D-	--E-	---------------M--------
Mycobacterium noviomagense	WP_083087071	-------N-DQ	Y-DS	---------------L--------
Mycobacterium xenopi	WP_003919183	-------E-DK	Y-DS	----------S-T-------M---
Mycobacterium algericum	WP_083037901	---Y---N-DQ	Y-A-	D--------A--T--L------V-
Mycobacterium engbaekii	WP_085128072	---Y---D-D-	Y-A-	---------A--T--L------V-
Mycobacterium heraklionense	WP_064890884	---Y---N-D-	Y-T-	---------A--T--L------V-
Mycobacterium hiberniae	WP_085135253	---Y---N-D-	Y-A-	---------A--T--L------V-
Mycobacterium kumamotonense	WP_065289186	-------N-DQ	Y-A-	D--------A--T--L------V-
Mycobacterium longobardum	WP_085266928	---Y---N-D-	Y-A-	D--------A--T--L------V-
Mycobacterium minnesotense	WP_083023941	---Y---N-D-	Y-A-	---------A--T--L------V-

Group	Species	Accession			
***Mycobacterium* Slow-Growers except *M. koreense* and *M. triviale* (67/69)**	*Mycobacterium senuense*	WP_085087702	---Y---D-DQ	Y-A-	D--------A--T--L------V-
	Mycobacterium sinense	WP_064856725	---Y---N-D-	Y-A-	D--------A--T--L------V-
	Mycobacterium terrae	WP_085259871	---Y---N-DQ	Y-A-	D--------A--T--L------V-
	Mycobacterium celatum	WP_062541227	-------N-D-		A--------------L------V-
	Mycobacterium kyorinense	WP_045381227	-------N-D-		A--------------L------V-
**Other *Mycobacterium* (3/38)**	*Mycobacterium acapulcensis*	WP_066810455	-------E-D-	Y-DS	D-----------T--L----H-V-
	Mycobacterium hassiacum	WP_018354365	-------N-DQ	Y-D-	------------T--L----H-V-
	Mycobacterium phlei	WP_061490017	-------N-D-	Y-DS	---------------L----H-V-
	Mycobacterium triviale	WP_085109743	-------N-D-		A-----------T---------V-
	Mycobacterium abscessus	WP_074376563	-Q-----D-D-		A---T-------T--L----L-V-
	Mycobacterium aromaticivorans	WP_036340473	------DD-DS		A-Y------------L------V-
	Mycobacterium aurum	WP_048630212	------DD-D-		A--------------L----H-V-
	Mycobacterium bacteremicum	WP_083058797	------DA-DE		A-Y------------L----H-V-
	Mycobacterium branderi	WP_083130004	-------N-D-		A--------------L------V-
	Mycobacterium canariasense	WP_062659801	-------N-D-		A-F------------L----H-V-
	Mycobacterium chelonae	WP_070915267	-Q-----D-D-		A-----------T--L----L-V-
	Mycobacterium chlorophenolicum	WP_048471550	-------A-DD		A--------------L----H-V-
	Mycobacterium chubuense	WP_014814488	-------D-D-		A--------------L----H-V-
	Mycobacterium confluentis	WP_085149149	-------N-D-		A-F------------L------V-
	Mycobacterium diernhoferi	WP_073856523	------DS-DD		A-Y------------L----H-V-
	Mycobacterium flavescens	WP_069414629	-------D-D-		A--------------L----H-V-
	Mycobacterium gilvum	WP_011895680	-------S-DD		A--------------L----H-V-
	Mycobacterium goodii	WP_049748000	-------N-D-		A--------S-----L----H-V-
	Mycobacterium holsaticum	WP_069407316	-------D-D-		A-----------T--L----H-V-
	Mycobacterium houstonense	WP_066903767	----D--N-D-		A--------------L----H-A-
	Mycobacterium insubricum	WP_083031096	-------D-D-		A-FA-Y---------L----L-V-
	Mycobacterium iranicum	WP_064283320	-------S-DD		A--------------L----H-V-
	Mycobacterium litorale	WP_078020541	-------D-DS		A-Y------------L------V-
	Mycobacterium moriokaense	WP_083153872	-------D-DS		Q-----Q-----T--L----H-V-
	Mycobacterium mucogenicum	WP_064858274	-------N-D-		A-F------------L----L-V-
	Mycobacterium neworleansense	CRZ15783	-------N-D-		A--------------L----L-V-
	Mycobacterium parafortuitum	WP_083145772	-------S-DD		A--------------L----H-V-
	Mycobacterium peregrinum	WP_064879385	-------D-D-		A--------------L----L-V-
	Mycobacterium porcinum	WP_069425395	-------N-D-		A--------------L----L-V-
	Mycobacterium rhodesiae	WP_083121572	-------N-DS		A-Y------------L------V-
	Mycobacterium rufum	KGI67211	-------A-DE		A-F------------L----H-V-
	Mycobacterium rutilum	WP_083410042	-------D-D-		A-----------T--L----H-V-
	Mycobacterium saopaulense	WP_070909490	-Q-----D-D-		A---T------ST--L----L-V-
	Mycobacterium septicum	WP_044516500	-------D-D-		A--------------L----L-V-
	Mycobacterium setense	WP_039317321	-------D-D-		A-----------T--L----L-V-
	Mycobacterium thermoresistibile	WP_003928122	-------N-D-		A-F------T--T--L----H-V-
	Mycobacterium tusciae	WP_006244271	-------D-DT		S----Y---S-----L----H-V-
	Mycobacterium vaccae	WP_003929448	-------S-DD		A--------------L----H-V-
	Mycobacterium wolinskyi	WP_067843019	------DN-D-		A--------------L----H-V-
**Other bacteria**	*Amycolatopsis mediterranei*	WP_013222762	-L-----VDSE		PH-E-YD--AQRT--L----F-V-
	Dietzia timorensis	WP_067477929	-M-----DETK		N---E-S--A--T--L----L-T-
	Gordonia amarae	WP_005189737	-Y-----D-D-		Q-F--Y---A--T--L----L-V-
	Hoyosella subflava	WP_013808705	-R-----D-D-		Q-F-E-E--A-----L----H-V-
	Nocardia brevicatena	WP_040833718	V------DGKG		QH-E--Q--A--T--F--I-----
	Rhodococcus rhodnii	WP_010839281	-------VS-E		QH-D--Q--A--T--L----L-V-
	Saccharomonospora saliphila	WP_019816464	-L-----IDDE		PH-E-YD--A--T-------FN--
	Skermania piniformis	WP_066472521	V------DSLG		EH-ET-Q-----T--F--------
	Tsukamurella paurometabola	WP_013125637	VY-----D-D-		A----YK--A-----F----L---
	Williamsia sterculiae	WP_076477037	-M-----N-DE		Q--E-----A--T--M----L-V-

Supplementary Figure 49

A partial sequence alignment of a conserved region of succinate dehydrogenase iron-sulfur subunit showing a four amino acid insertion that is specific for most members of the *Mycobacterium* Slow-Growers except *M. koreense* and *M. triviale,* and absent in most other bacteria.

Group	Species	Accession	51		100
***Mycobacterium* Slow-Growers (24/24)**	*Mycobacterium avium*	WP_009976218	RWWPLDSQRTDALLRAMFDDAAKNI		GPHAAARQLVASLIHEIIGRMLPLV
	Mycobacterium avium subsp. avium	EUA26778	-------------------------		-S---T-------------------
	Mycobacterium indicus pranii M	AFS13919	-------------------------		-------------------------
	Mycobacterium intracellulare	WP_064933097	-----------T--Q-------N--		-------------------------
	Mycobacterium marseillense	WP_083016487	-------R---T----------D--		-H-------A---V-----------
	Mycobacterium paraintracellulare	AFC53364	-------------------------		------------F------------
	Mycobacterium yongonense	WP_065500060	-----------T--Q----------		-------------------------
	Mycobacterium shimoidei	WP_069397580	-----A-AVSGDR-QI---GI-ADM		TVRS---E-ATA-T-TV---VIA--
	Mycobacterium celatum	WP_062540170	-----A-AL-TNR-QM---G--ADM		DVRS---E-ATT-V-TVV--VVA--
	Mycobacterium heckeshornense	WP_048892526	-----A-AL-TDR--V---N--ADM		-RS------ADT-V-TV---VVA--
	Mycobacterium kyorinense	WP_045381159	-----A-AC-TDR-QV---G--TDM		DVRS---D-ATM-V-TV---VVA--
	Mycobacterium noviomagense	WP_083087344	-----A-AL-TNR-QV------AGM		DRR----DVA-T-V-TV---VVA--
	Mycobacterium xenopi	WP_085196226	-----A-AL-TDR-QV---N--ADM		DRR------ADT-V-TV---VVA--
	Mycobacterium algericum	WP_083036315	--C-M--VL-IDR-GSV--ET-TVA		-RRM-GNL-AGR-T--VL--V----
	Mycobacterium arupense	ORA00844	--F----VF-LDR--VR-YET-AKT		D-R---NV-A------VL--L----
	Mycobacterium heraklionense	OBG40265	--F----VF-IDR--IR--ET-ALT		DRR--GNV-A-R----VL--L----
	Mycobacterium icosiumassiliensis	WP_067971687	--F----VF-IDR--VR--ET-AMT		DRR--GNV-A-R----VL--L-H--
	Mycobacterium minnesotense	ORB04459	--F----VF-LDR--VR-YET-AKT		D-R---NV-A------VL--L----
	Mycobacterium sinense	AEF35516	--C-M--VL-IDR-GSV--ET-TVA		-RRM-GNL-AGR-T--VL--V--V-
	Mycobacterium longobardum	WP_085263512	--F----VF-VDR--VRL-ET-ELT		DRR--GNV-A-R-V--VL--L----
	Mycobacterium senuense	WP_085083594	--C-M--VL-IDR-GSV--ET-TVA		-RR--GNL-AGR-T--VL--V----
**Other *Mycobacterium* (0/57)**	*Mycobacterium abscessus*	WP_074302546	-----ARALNTDR-EQ-YAR-VEET	G	SDSV-VH--ADA-V-TVV--LVA--
	Mycobacterium abscessus subsp. bolletii	AIV18401	-----ARALNTDR-EQ-YAR-VEET	G	SDSI-VH--ADA-V-TVV--LVA--
	Mycobacterium aromaticivorans	WP_036344984	-------V -SGR-A---AS--DEL	G	DRR---H-IA-TFT-AVL--VVT-F
	Mycobacterium aurum	WP_048631062	-----AEIL-TDR-NT--AAGMAET	D	SRASV-Q--A-TFA-VVV--VV--L
	Mycobacterium austroafricanum	WP_036372793	-----AEIV-TDR-NT---A-LVET	E	SSV-VTQ--A-TFA-VVV--VTA-L
	Mycobacterium bacteremicum	WP_083060746	-----IEVV-TDR--L---A-VAET	D	SRA-V-Q--A-T-S-VV---VV---
	Mycobacterium boenickei	WP_077740874	-----AEALSADR-QG---A-MAET	G	NRA-V-Q--A-TF--VV---VV--L
	Mycobacterium brisbanense	WP_062828148	-----EEAVGAGR-QG---A-VAET	G	NRA-V-H--A-T-A-VV---VV--L
	Mycobacterium canariasense	WP_062656964	-----AEVL-TDR--T---I-IAET	D	SHA-V-Q--A-T-A-VVV--VV--I
	Mycobacterium celeriflavum	WP_083002579	-----AEAL-TER-QV---L--EET	D	SRA-V-H--A-T-A-VVV--VV--L
	Mycobacterium chelonae	WP_070919867	-----ARALNTDR-ER-YAR-IEET	G	SDAV-VH--ADA-V-TVV--LVA--
	Mycobacterium chlorophenolicum	WP_048469260	-----AELL-TDR-SR-Y-A-LAES	G	--A-VTQ--A-TFA-VVV--VI--L
	Mycobacterium chubuense	WP_014816032	-----AEVL-TDR-HR--QAGVAEA	D	S-T-VT---A-T-A-VVV--V---L
	Mycobacterium confluentis	WP_085150871	-----A-ALSGEKFEQ-YR---LEM	D	SRT---Q--A-T-A-AV---VVA--
	Mycobacterium diernhoferi	WP_073858248	-----VEVM-TDR-Q----AGVAET	D	S-A-V-Q--A-T-A-VV---VV---
	Mycobacterium doricum	WP_085192867	-----TDAL-GGR-Q----R-SAET	D	NRA-V-Q--A-T-A-VVL--VV--L
	Mycobacterium fallax	WP_085095692	-----EEAL-RGR-AE--ELT-AE-	G	N-AV--Q--A---A-AV---VVA--
	Mycobacterium farcinogenes	WP_036387146	-----AEALSADR-QG---A-MAET	G	NRA-V-Q--A-TF--VV---VV--L
	Mycobacterium flavescens	WP_069416611	-----AEAL-GNR-E--Y-L--QET	D	SRA-V-Q--A-T-A-VV---VI---
	Mycobacterium fortuitum	WP_064867454	-----AEALSADR-QG---A-MAET	G	NRA-V-Q--A-TFT-VV---VV--L
	Mycobacterium franklinii	WP_078336352	-----ARALNTDR-EQ-YAR-IDET	G	SDAV-VH--ADA-V-TVV--LVA--
	Mycobacterium gilvum	WP_013471651	-----TEVL-TDR-DR---AGVAET	D	SRT-VTQ--A-TFA-VVV--VI--L
	Mycobacterium goodii	WP_049746525	-----TEAL-ADR-Q----AGVAET	D	NRP-V-Q--ATT-A-VV---VI--L
	Mycobacterium hassiacum	WP_005627741	-----AEAV-GNR-EL--AR--EET	E	SRT-ITQ--A-T-A-VVL--VI--L
	Mycobacterium holsaticum	WP_069406818	-TEAL-TDR-Q----L--EET	D	S-T-V-Q--A-T-A-VVV--VI--L
	Mycobacterium houstonense	WP_066897632	-----AEALSADRMQ----A-MAET	A	NRA-V-Q--A-T-T-VVV--VV--L
	Mycobacterium immunogenum	WP_064630995	-----ARALNTDR-EQ-YAR-VDET	G	SGSI-VH--ADA-V-TVV--LVA--
	Mycobacterium insubricum	WP_083028975	-----AEAL-AGR-AD-H-A-VAD-	G	N-V---Q--A-T-A-SV---VVA--
	Mycobacterium iranicum	WP_064285360	-----AEIL-TDR-DT---AGVAET	D	SRA-V-Q--A-TFA-VV---VI--L
	Mycobacterium komanii	CRL71595	-----ADAL-TER-QT---L--EET	D	SRA-V-H--A-T-A-VVV--VV--I
	Mycobacterium litorale	WP_078019330	---S-E-VT-TGR-DD--ES--TEL	G	NRR-V-Q--A-TFT-AVL--VVT-F
	Mycobacterium llatzerense	WP_071289191	-----AEAV-GDR-Q----T-VLDT	G	SESV--Q-VA-M-A-VV---VV--L
	Mycobacterium mageritense	WP_036439856	-----SDALDADR-Q----V-VAET	D	NRA-V-Q--A-T-A-VV---VV--L
	Mycobacterium malmesburyense	CRL72873	-----ADAL-TDR-K----L--EET	D	SRA-V-H--A-T-A-VVV--VV--I
	Mycobacterium moriokaense	WP_083150750	-----MQAL-TDR-HS--QL-SEET	E	NRA-V-Q--A-T-A-VV---VV--L
	Mycobacterium mucogenicum	WP_064980837	-----AEAV-GDRMQ--Y-T-MGDT	G	SAAV--Q-VA-M-A-VVV--VV--L
	Mycobacterium neworleansense	CRZ14130	-----AEALSADR-QG---A-MAET	G	NRA-V-Q--A-TF--VV---VV--L
	Mycobacterium novocastrense	WP_067392350	-----ADAL-TDR-QT---L--EET	D	SRA-V-H--A-T-A-VVV--VV---
	Mycobacterium obuense	WP_046366702	-----TELM-TDR-SR--AAGLAE-	G	N-ATVTQ--A-TFA-VVV--VI--L
	Mycobacterium parafortuitum	WP_083143816	-----AEIL-TDR-QQ---AGIAET	E	SRA-V-Q--A-TFA-VVV--VT--L
	Mycobacterium peregrinum	WP_064885630	-----AEALSADR-QS---A-MEET	G	NRA-V-Q--A-TF--VV---VV--L
	Mycobacterium phlei	WP_003891194	-----AEAL-TDR--V---Q-VAET	D	NRA-VTQ--A-T-A-VVV--VI--L
	Mycobacterium porcinum	WP_069425408	-----AEALSADR-QG---A-LAET	G	NRA-V-Q--A-TFT-VV---VI--L

Group	Species	Accession	Sequence
Other *Mycobacterium* (0/57)	*Mycobacterium rhodesiae*	WP_083119073	-------A -SGR-T---ES--DEL G DRR---Q--A-TFT-AVL--VVT-F
	Mycobacterium rufum	KGI68392	-----AELVSTDR-PR---A-LAES A --A-V-Q--A-TFA-VVV--VI--L
	Mycobacterium rutilum	WP_083408731	-----AEAL-GNR-E---EL--EET D SRT-V-H--A-T-A-VV---VI---
	Mycobacterium salmoniphilum	WP_078327273	-----ARALNTDR-ER-YAH-IEET G SDAV-VH--ADA-V-TVV--LVA--
	Mycobacterium saopaulense	ALR14091	-----ARALNTDR-EQ-YAR-V-ET G SDSI-VH--ADA-V-TVV--LVA--
	Mycobacterium septicum	WP_044518805	-----AEALSADR-QG---A-MAET G NRA-V-Q--A-TFT-VV---VV--L
	Mycobacterium setense	WP_039320935	-----AEALSADR-QG---A-MAET G NSA-V-Q--A-TF--VV---VV--L
	Mycobacterium smegmatis	WP_003895084	-----AEAL-ADR-QM---AGIAET G NRP-V-Q--ATT-A-VV---VI--L
	Mycobacterium thermoresistibile	WP_040548167	-----AEAL-TDR-AG--AT-VAET D N-A---Q--A-T-A-VVV--VI--L
	Mycobacterium tusciae	WP_083127261	-----TQAL-TDR-HS--KL--EET E SRA-V-Q--A-T-A-VVV--VV--L
	Mycobacterium vaccae	WP_003932304	-----TDLL-TDR-HS--EAGVAET E SRA-V-Q--A-TFA-VVV--VI--L
	Mycobacterium vanbaalenii	WP_011780293	-----AEIV-TDR-NT---A-LVET E SSV-VTQ--A-TFA-VVV--VTA-L
	Mycobacterium vulneris	WP_065457365	-----AEALSADR-QG---A-LAET G NRA-V-Q--A-TFT-VV---VI--L
	Mycobacterium wolinskyi	WP_067851867	-----AEALSGDR-QL---T-IAET D NRA-V-Q--A-T-A-VV---VV--L
Other *Corynebacteriales*	*Hoyosella altamirensis*	WP_064440353	-----AAALSEDR-EQ-HSRMVRDL G K-GL-SA-VANVV--AVVS-VMA-I
	Hoyosella subflava	WP_013807914	-----AAALSEDR-EQ-YARMMRDL G K-DL-SA-VANVV--AVVS-V-A-I
	Millisia brevis	WP_084351553	---A-GGP-ASERIE--I-R-EEDH P DRRV--Q-VAGACV-A-V--VATS-
	Nocardia abscessus	WP_043690259	---Q-SDGVREGRVEL-YRRH-AEM I SADI--EVVATA---A-V--VVA-L
	Nocardia brasiliensis	WP_014984736	---R-ADGVREGRIEL-YRRH-AEM I RADV--EVVATA---AVV--VTA-L
	Nocardia caishijiensis	WP_067981145	---HVPNGLSEGRVEL-YRRH-AEM I NDDI--EVVATA---AVV--VTA-F
	Nocardia carnea	WP_033246208	---A-STGLREGRIDL-YRRH-AEM I SAT---EVVATA---AVV--VAA-W
	Nocardia mikamii	WP_062997360	---R-ADGVREGRIEL-YRRH-AEM V S-GI--EVVATA---AVV--VVA-A
	Nocardia paucivorans	WP_051133071	---R-ADDPREGRIEL-YRRH-AEM I SAR---EVVATA---AVV--VVA-W
	Nocardia salmonicida	WP_062983527	---HVPNGLSEGRVEL-YRRH-AEM I NDEI--EVVATA---AVA--VTA-F
	Nocardia seriolae	WP_033089459	---R-ADGIREGRIEL-YRRH-AEM S N-DT--EVVATA---AVV--V-AM-
	Nocardia shimofusensis	WP_067862152	---K-SEGVREGRIEM-YRRH-AEM R SADI--EVVATA-V-A-V--VAA-W
	Nocardia sienata	WP_063058082	---A-STGLREGRIDL-YRRH-AEM I SAT--SEVVATA---AVL--VAA-W
	Nocardia soli	WP_071928701	---HVPHGLSEGRVEL-YRRH-AEM I NDEV--EVVATA---SVV--STA-F
	Nocardia speluncae	WP_068035899	---S-SAGLREGRIDL-YRRH-AEM A SATV--EVVATA---GVV--VAA-W
	Nocardia vinacea	WP_040689945	---R-ADGLREGRIEL-YRRH-AEM I SADT--EVVATA---AV---VVA-L
	Nocardia violaceofusca	WP_063065272	---R-ADGVREGRIEL-YRRH-AEM V SAEI--EVVATA---AVV--VVA-A
	Nocardia vulneris	WP_043678577	---R-ADGVREGRIEL-YRRH-AEM I RADV--EVVATA---AVV--VTA-L
	Nocardia xishanensis	WP_068055140	---K-SEGLREGRIEL-YRRH-AEM I SADT--EVVATA---AV---VAA-L
	Rhodococcus fascians	ORI20639	---Q-AVGIEQGRVER-ITRSLEDV K T-EV--I-VASAV--A-V--VVA-L
	Rhodococcus imtechensis	WP_007300698	---S-AEDLDGDRVSR-WARSLEDV P D-DV--M-VATA---AVV--VTA--
	Rhodococcus jostii	SEC30590	---S-ADDLDGDRVPR-WARSLEDV P D-DV--M-VATA---AV---VTA--
	Rhodococcus koreensis	SEB82951	---S-AGDLDGDRVPR-WARSLEDV P D-DV--M-VATA---AVV--VTA--
	Rhodococcus opacus	ANS28209	---S-ADDLDGDRVSR-WARSLEDV P D-DV--M-VATA---AVV--VTA--
	Rhodococcus phenolicus	WP_068160431	---R-AGEDR-RRVELLYRRVLEDV E D-RI-VE-VAGA---V-V--V-APL
	Rhodococcus pyridinivorans	WP_060653028	---F-GGGERQQRIE-LY-R-LLDT E D-RI-VE-VAGA---AVV--V-APY
	Rhodococcus qingshengii	WP_064688199	---Q-SEGLCGDRVHQ--ARSLQDL D SAE---I-VAT----AVV--VATSI
	Rhodococcus rhodnii	WP_010839692	---A-ADAPGNDRVGR-YRRVLLDL P D-E---L-VAD----AVV--VAA-I
	Rhodococcus rhodochrous	WP_016694287	---F-GGGERQQRVE-LY-R-LLDT E D-RI-VE-VAGA---AVV--V-APY
	Rhodococcus triatomae	SDI38600	---A-GGAQRASRISL-L-R-VDDL G D-D---L-VAS----AVV--VTA-T
	Rhodococcus tukisamuensis	SDD45021	---A-RQGIRGGR-DR-VER--EDV G TRE--GAAVATA---AVV--VAA--
	Rhodococcus wratislaviensis	WP_037238930	---S-ADDLDGDRVPR-WARSLEDV P D-DV--M-VATA---AVV--VTA--
	Rhodococcus yunnanensis	WP_072801742	---Q-AAGLD-GRTDQ-LARCVEDL R TTE---S-VATA-V-AVV--VAA-L
	Rhodococcus zopfii	WP_072808708	---R-AGEDRGQRVGLLYRRVLEDV V D-RI-VE-VAGA---VVV--V-APF

Supplementary Figure 50

A partial sequence alignment of a conserved region of a hypothetical protein showing a one amino acid deletion that is specific for members of the *Mycobacterium* Slow-Growers and absent in other *Corynebacteriales*.

***“Tuberculosis-Simiae”* Clade (63/63)**

		81	119
Mycobacterium africanum	WP_031701648	LALAYRTRPVFVPDAD	NDPVARYRAVVLARLRLVGIGIP
Mycobacterium alsense	WP_083140568	---G---------SNT	-----------VS------V---
Mycobacterium angelicum	WP_083111069	-------------NN-	---I----SM-MS---M------
Mycobacterium arosiense	WP_083065534	--V----------SN-	---------L--S-----SA---
Mycobacterium asiaticum	WP_065035308	-------------SN-	------------Q----M-F-V-
Mycobacterium avium	WP_062887272	--V----------SN-	---------L--S-----SV-V-
Mycobacterium avium subsp. avium 2285 (R)	EUA36772	--V----------SN-	---------L--S-----S--V-
Mycobacterium avium subsp. hominissuis	ETB44615	--V----------SN-	---------L--S-----SV-V-
*Mycobacterium avium subsp. paratuberculo*sis	ETB35581	--V----------SN-	---------L--S-----SV-V-
Mycobacterium avium subsp. silvaticum	ETB14850	--V----------SN-	---------L--S-----SV-V-
Mycobacterium bohemicum	WP_085181997	-------------SN-	--------T--MS---V--V-V-
Mycobacterium bovis	WP_019283731	----------------	-----------------------
Mycobacterium branderi	WP_083129858	--M----------SNG	--------T---S----I-----
Mycobacterium canettii	WP_015291267	----------------	-----------------------
Mycobacterium colombiense	WP_064877082	--V----------SN-	---------L--S-----SA---
Mycobacterium conspicuum	WP_085231379	M------------SS-	S--------L-MS----L-----
Mycobacterium europaeum	WP_085241601	--V----------SN-	------------S--------V-
Mycobacterium florentinum	WP_085223062	-------------SN-	--------T--MS-----A----
Mycobacterium fragae	WP_085195828	-------------SNG	--------T--MS---EF-----
Mycobacterium gastri	WP_085104988	-------------SN-	---------------------V-
Mycobacterium genavense	WP_025735717	-------------SN-	--------T--MS-----A----
Mycobacterium gordonae	WP_065047223	--V----------SN-	---------I--S----M-----
Mycobacterium haemophilum	WP_047313259	--V----------SH-	---------A--S---F------
Mycobacterium heidelbergense	WP_083073123	-------------SNT	-----------VS----------
Mycobacterium interjectum	WP_085201614	--V----------SNN	-----------VS---A----V-
Mycobacterium intermedium	WP_069419056	-------------SNE	---------A-MG--------V-
Mycobacterium intracellulare	WP_064938499	--V----------SN-	---------L--S-----SA---
Mycobacterium kansasii	WP_063467948	-------------SN-	---------L-----------V-
Mycobacterium lacus	WP_085157001	-------------NN-	-----------QS----------
Mycobacterium lentiflavum	CQD08526	-G-----------SN-	--------T--MS-----AV---
Mycobacterium leprae	WP_010907856	--V----------SH-	---------MA-S----I-V---
Mycobacterium lepromatosis	WP_045842624	--V---A------SH-	---------M--S------V---
Mycobacterium liflandii	WP_015354907	-------------NN-	--------T--------F-----
Mycobacterium malmoense	WP_065445036	--V----------SN-	------------S--------V-
Mycobacterium mantenii	WP_083096129	--V----------SN-	---------L--S------A-V-
Mycobacterium marinum	WP_012393237	-------------NN-	--------T--------F-----
Mycobacterium marseillense	WP_083020076	--V----------SN-	---------L--S-----SA---
Mycobacterium nebraskense	WP_085165081	--V----------SSE	------------S--------V-
Mycobacterium palustre	WP_085077998	-------------NNT	-----------MS----------
Mycobacterium paraense	WP_085103820	-------------SNN	-----------VS---V------
Mycobacterium paraffinicum	WP_073876024	--V----------SN-	-----------VS--------V-
Mycobacterium parascrofulaceum	WP_007168175	--V----------SN-	------------S--------V-
Mycobacterium paraseoulense	WP_083170110	--V----------SS-	------------S--------V-
Mycobacterium parmense	WP_085267731	--T----------SNE	---------L------V----V-
Mycobacterium pseudoshottsii L15	GAQ36875	-------------NN-	--------T--------F-----
Mycobacterium riyadhense	WP_085249736	-------------NN-	------------S----------
Mycobacterium saskatchewanense	WP_085257921	--V----------NN-	--------T--M----
Mycobacterium scrofulaceum	WP_067277590	--V----------SN-	------------S--------V-
Mycobacterium sherrisii	WP_069402474	------S------SN-	-----------MT-----A--V-
Mycobacterium shimoidei	WP_069396543	-------------SNG	--------TM-MS----------
Mycobacterium shinjukuense	WP_083049847	-------------NN-	A-----------G------L---
Mycobacterium simiae	WP_061558588	------S------SN-	-----------MT---V-A--V-
Mycobacterium szulgai	WP_085674308	-------------NN-	---I-------MS---M------
Mycobacterium triplex	WP_036467404	-------------SN-	--------T--MS-----A----
Mycobacterium tuberculosis	WP_064314163	----------------	-----------------------
Mycobacterium ulcerans	WP_011740463	-------------NN-	--------T--------F-----
Mycobacterium vulneris	WP_085292559	--V----------SN-	---------L--S-----SA---
Mycobacterium yongonense	WP_065498511	--V----------SN-	---------L--S-----SA---
Mycobacterium celatum	WP_085167690	-------------SNG	--------T---S----I---V-
Mycobacterium heckeshornense	WP_048891255	-------------SDG	------------S---V--F---
Mycobacterium kyorinense	WP_065016588	--A----------SNG	--------T--MS---VI---L-
Mycobacterium noviomagense	WP_083086255	-------------SDG	-----------MT---VLA----
Mycobacterium xenopi	WP_085196643	-------------SDG	------------S---V--F---

Other *Mycobacterium* (0/76)

Mycobacterium algericum	WP_083035837	--M-F-V------SNG A --------TA--S-----AF---
Mycobacterium arupense	WP_046188452	M-M-F-A------TNG V --------TT--S------F---
Mycobacterium engbaekii	WP_085129314	M-T-F-A------SNG V --------TT--S----F-F---
Mycobacterium heraklionense	WP_064998289	M-T-F-V------SNG V --------TT-------F-F---
Mycobacterium hiberniae	WP_085133936	M-T-F-A------SNG V --------TT--S----F-F---
Mycobacterium icosiumassiliensis	WP_067970901	--M-F-V------SNG V --------TTA-S----F-F---
Mycobacterium koreense	WP_085302486	-V----S------SNG P D---E---TI-MS--H-I---V-
Mycobacterium kumamotonense	WP_065287459	--M-F-V------SNG A --------TA--S----------
Mycobacterium longobardum	WP_085264345	--M-F-V------NNG V --------TT--S----F-F---
Mycobacterium minnesotense	WP_083027533	-----SNG V --------TT--S------F---
Mycobacterium senuense	WP_085081726	--I-F-A------SNG A --------TA--S----FAF---
Mycobacterium sinense	WP_064854792	--M-F--------SNG A --------TA--S------F---
Mycobacterium triviale	WP_069393366	-V----S------SNG P D---E---TI-MS--H-I---V-
Mycobacterium abscessus	WP_062914945	FG----A------AKG P G-AL-Q---LI-S-V--FLVSV-
Mycobacterium abscessus subsp. bolletii 103	EUA79755	FG----A------AKG P G-AL-Q---LI-S-V--FL--V-
Mycobacterium acapulcensis	WP_083997650	-------------TNG P -----A--TA-M-------F-V-
Mycobacterium aromaticivorans	WP_036340069	-------------TVG P --------TA-M-----F-F---
Mycobacterium aurum	WP_048630391	-T-----------TVG P ---I----TA-MT--------V-
Mycobacterium bacteremicum	WP_083057290	-------------T-G P --------TT-MS----F-----
Mycobacterium boenickei	WP_077743615	------S------A-G P --------TT-------F---V-
Mycobacterium brisbanense	WP_062831658	-------------A-G P --------TT-------F---L-
Mycobacterium canariasense	GAS93131	MT-----------TNG P ---I----TT-MS----F-----
Mycobacterium celeriflavum	WP_083006383	-------------TNG P -----S--TA-M-------F-V-
Mycobacterium chelonae	WP_070919384	FG----A------TKG P G-AL-Q---LI-S-V--FV--V-
Mycobacterium chlorophenolicum	WP_048470747	-------------T-G P ---I----TA-M---------V-
Mycobacterium chubuense	WP_041781791	-------------T-G P ---I----TA-MT----I---V-
Mycobacterium conceptionense	WP_085140998	------S------A-G P --------TT-------F---V-
Mycobacterium confluentis	WP_085148630	-------------A-G P --------TT--T----I-L-V-
Mycobacterium cosmeticum	CDO10255	MT-----------TNG P ---I----TT-MS----F-----
Mycobacterium diernhoferi	WP_079244263	MT-----------T-G P --------TT-MS----F-----
Mycobacterium doricum	WP_085190019	-------------TVG P ---I----TT-MS----F-----
Mycobacterium elephantis	WP_083042562	-------------TDG P --------TA-MT--K-----V-
Mycobacterium fallax	WP_085092481	---S-TM------VSG P E-------TAA-M--K-F-F---
Mycobacterium farcinogenes	CDP84269	------S------A-G P --------TT-------F---V-
Mycobacterium flavescens	WP_069416781	M------------TNG P -----T--TA-MT--------V-
Mycobacterium fortuitum	WP_061262691	------S------A-G P --------TT-------F---V-
Mycobacterium franklinii	WP_070938762	FG----A------AKG P G-AL-Q---LI-S-V--FL--V-
Mycobacterium gilvum	WP_011895490	-------------T-G P ---I----TA-M-----I---V-
Mycobacterium goodii	WP_049747760	-------------T-G P --------TT-M-----F-----
Mycobacterium hassiacum	WP_036447587	------S------TNG P D--L----TA-MT----I-----
Mycobacterium holsaticum	WP_069403956	-------------TDG P --------TA-MT--K-----V-
Mycobacterium immunogenum	WP_064628010	FG----A------AKG P G-AL-Q---LI-S-V--FL--V-
Mycobacterium insubricum	WP_083030763	M-----S------VSG P D-------TAA-M--KMF-----
Mycobacterium iranicum	WP_036465789	-T-----------T-G P ---I----TA-M---------V-
Mycobacterium komanii	CRL78140	-------------TNG P -----S--TA-M-------F---
Mycobacterium litorale	WP_078020415	-------------TTG P --------TA-M---K-F---V-
Mycobacterium llatzerense	WP_071286862	M-----S------ASG P ---------T--S----FA--V-
Mycobacterium mageritense	WP_043367677	------S------A-G P --------TT-------F---V-
Mycobacterium malmesburyense	CRL77529	-------------TNG P -----A--TA-M-------F-V-
Mycobacterium moriokaense	WP_083154147	-------------TNG P --------TA-MT----I---V-
Mycobacterium mucogenicum	WP_064860812	M-----S------ASG P ---------T--S--Q-FA--V-
Mycobacterium neoaurum	CDQ46083	-------------TVG P --------TT-MS----F-----
Mycobacterium neworleansense	CRZ15539	------S------A-G P --------TT-------F---V-
Mycobacterium nonchromogenicum	WP_085137944	M-T-F-V------SNG V --------TT-------F-F---
Mycobacterium novocastrense	WP_067395782	-------------TNG P -----A--TA-M-------F-V-
Mycobacterium obuense	WP_046365193	---------T-G P ---I----TA-MS----I-L-V-
Mycobacterium parafortuitum	WP_083143055	---------T-G P ---I----TA-MT----I---V-
Mycobacterium peregrinum	WP_064878842	-------------A-G P --------TT-------F---V-
Mycobacterium phlei	AMO60535	-------------TNG P ---I-Q--TA-MT----I---V-
Mycobacterium porcinum	WP_075921444	------S------A-G P --------TT-------F---V-
Mycobacterium rhodesiae	WP_083121063	-------------TVG P --------TA-M-----F-F---
Mycobacterium rufum	KGI67404	-------------T-G P ---I----TA-M---------V-
Mycobacterium rutilum	SEH90102	M------------TNG P --------TA-MT--------V-
Mycobacterium salmoniphilum	WP_078330912	IG----A------TKG P G-AL-Q---LI-S-V--FV--V-
Mycobacterium saopaulense	WP_070909629	FG----A------AKG P G-AL-Q---LI-S-V--FV--V-

Group	Species	Accession	Sequence
Other *Mycobacterium* (0/76)	*Mycobacterium septicum*	WP_044516826	------S------A-G P --------TT-------F---V-
	Mycobacterium setense	WP_064871743	------N------ATG P --------TT-------F---V-
	Mycobacterium smegmatis	WP_011728043	-------------T-G P --------TT-M-----F---V-
	Mycobacterium terrae	WP_085261981	--M-F-A------SGG A --------TA--S------F---
	Mycobacterium thermoresistibile	WP_003927921	-------------TNG P D-------IA-M----MY-V---
	Mycobacterium tusciae	WP_083125071	-------------TTG P --------TA-MS--------V-
	Mycobacterium vaccae	WP_003929184	---------T-G P ---I----TA-M-------V-V-
	Mycobacterium vanbaalenii PYR-1	A1T636	M------------T-G P ---I----TA-M---------V-
	Mycobacterium vulneris	CDO27584	------S------A-G P --------TT-------F---V-
	Mycobacterium wolinskyi	WP_067856414	-------------T-G P --------TT-M-----F-----
Other bacteria	*Actinoalloteichus cyanogriseus*	WP_081715234	-W----S------ASG P D-------TA-T--IK-FAF---
	Amycolatopsis coloradensis	WP_076168946	-MI---S------ISG N D--L----SAIVG-I--F-----
	Gordonia effusa	WP_007315795	--A---S------ASS G V--L----TT-MTH—WFS
	Lechevalieria aerocolonigenes	WP_045316034	-Y----A------VSG P D-------T--MR-M--FA----
	Lentzea waywayandensis	SFR22058	-Y----A------VSG P D-------T--MR-M--FA----
	Nocardia coubleae	WP_067637519	-L----S------V-G P ---I----TT-M----WF-----
	Prauserella rugosa	WP_030534398	-MI----------VSG E D--L----SI-VG-VK-F-----
	Pseudonocardia acaciae	WP_084211920	--V-F-S------VSG P E--I----T-IIQ---VFA--V-
	Rhodococcus marinonascens	WP_072687860	-L----S------VSG P --------TT-MT----F-LV--
	Saccharomonospora marina	WP_009152748	-WI----------VSG A D--L----SL-V--T--F-----
	Saccharothrix espanaensis	WP_015098463	-I----S------VSG P D-------S--TR-MK-FA--L-
	Saccharothrix syringae	WP_063741304	-II----------VSG P D---------AR-M--FA--L-
	Smaragdicoccus niigatensis	WP_051090901	-VM----------V-G P ---L-----T-VS---SF-VTL-
	Streptoalloteichus hindustanus	SHG37786	--I---S------VTG P D---S---SF-VS----L---V-
	Streptomyces regensis	KMS85025	-MI----------VSG E D--L----SI-VG-VK-F-----
	Thermocrispum municipale	WP_028852384	-FI---N------V-S Q E--L----STIV--V--F-----
	Tomitella biformata	WP_024795479	ILI-----S----VSN P H-------SL-MGKRKTFAL---
	Williamsia herbipolensis	WP_045823554	------S------TSG P G--I-------MT-I-
	Yuhushiella deserti	SFP10542	-VI----------VSG A D--L----SL-VS-V--F-----

Supplementary Figure 51

Detailed sequence information for the one amino acid deletion found in a hypothetical protein, which is shown in Figure 8. This deletion is specific for members of the *"Tuberculosis-Simiae"* clade and absent in most other bacteria.

Clade	Species	Accession	403		450
“Tuberculosis-Simiae” Clade (42/42)	*Mycobacterium africanum*	WP_080699385	ALNNAVPQGLSSSIFTTDLREAEHFLD		QSDCGIANVNIGTSGAEIGGA
	Mycobacterium alsense	WP_083139400	----G-------A----------R---		A--------------------
	Mycobacterium angelicum	WP_083111172	----------------------QR--A		G--------------------
	Mycobacterium arosiense	WP_083064763	----G-------A----------R--A		E--------------------
	Mycobacterium avium	WP_065370811	------------A----------R---		E--------------------
	Mycobacterium avium subsp. avium 2285 (S)	EUA28286	-----------------------R--A		E--------------------
	Mycobacterium avium subsp. paratuberculosis	ETB36054	------------A----------R---		E--------------------
	Mycobacterium branderi	WP_083129974	----G-------A-----M----R-I-		G-----T--------------
	Mycobacterium bovis	WP_081045359	---------------------------		---------------------
	Mycobacterium canettii	WP_015303803	---------------------------		---------------------
	Mycobacterium colombiense	WP_064877558	------------A----------R--A		E--------------------
	Mycobacterium florentinum	WP_085219983	----G-------A----------R---		G--------------------
	Mycobacterium fragae	WP_085198589	------------A-----M----R-I-		G--------------------
	Mycobacterium gastri	WP_036420058	------------A---N-----QR---		E--------------------
	Mycobacterium genavense	WP_036468424	------------A----------R---		G--------------------
	Mycobacterium haemophilum	WP_047313509	------------A----------R---		G--------------------
	Mycobacterium heidelbergense	WP_083073230	----G-------A--------P-R---		G--------------------
	Mycobacterium intracellulare	WP_064939487	-----------------------R--A		E--------------------
	Mycobacterium kansasii	WP_063466821	------L-----A---N---------N		E--------------------
	Mycobacterium lacus	WP_085159383	------------A----------R---		G--------------------
	Mycobacterium liflandii 128FXT	AGC61363	------------A---N------R--A		A--------------------
	Mycobacterium malmoense	WP_071513263	----D-------A----------R---		G--------------------
	Mycobacterium mantenii	WP_083099681	---------------------S-R--A		E--------------------
	Mycobacterium marinum	WP_012393117	------------A---N------R--A		A--------------------
	Mycobacterium marseillense	WP_083017483	-----------------------R--A		E--------------------
	Mycobacterium microti	AMC61060	---------------------------		---------------------
	Mycobacterium mungi	OAQ16701	---------------------------		---------------------
	Mycobacterium nebraskense	WP_047321987	------------A----------R--A		G--------------------
	Mycobacterium riyadhense	WP_085251029	-------------M--------QR---		G--------------------
	Mycobacterium shimoidei	WP_069395251	--------R---A-----V----R-I-		G-----T--------------
	Mycobacterium shinjukuense	WP_083048657	----S-------A----------R---		G---------V----------
	Mycobacterium szulgai	WP_085671660	----------------------QR--A		G--------------------
	Mycobacterium triplex	WP_036467166	S-----------A----------R---		G--------------------
	Mycobacterium tuberculosis	WP_072139271	---------------------------		---------------------
	Mycobacterium ulcerans	WP_011740591	------------AF--N------R--A		A--------------------
	Mycobacterium yongonense	WP_065508338	-----------------------R--A		E--------------------
	Mycobacterium celatum	WP_085167961	----D-------A---------QR-I-		G-----T-I------------
	Mycobacterium heckeshornense	WP_048891868	------------A-----I----R-I-		G--------------------
	Mycobacterium kyorinense	WP_065014964	-V----------------M----R-I-		G-----T--------------
	Mycobacterium noviomagense	WP_083087106	----G-------A---A-VQ---R-I-		E--------------------
	Mycobacterium xenopi	WP_085193125	------------A-----V----R-I-		G--------------------
Other *Mycobacterium* (0/55)	*Mycobacterium abscessus*	WP_074251137	------------A---L-M----R--A	AD	G--------------------
	Mycobacterium abscessus subsp. bolletii	EUA66823	------------A---L-M----R--A	AD	G--------------------
	Mycobacterium aromaticivorans	WP_036340398	-----------A-----V----R--A	AD	G--------------------
	Mycobacterium aurum	WP_048630256	-M----------A---L-V----R--A	AD	G--------------------
	Mycobacterium austroafricanum	WP_036369728	----------------L-M----R--A	AD	G--------------------
	Mycobacterium bacteremicum	WP_083057477	------------A-----I----R-MA	AD	G--------------------
	Mycobacterium boenickei	WP_077743440	------------A-----V----R-MA	AD	G--------------------
	Mycobacterium brisbanense	WP_062831498	----E-------A-----V----R--A	AD	G--------------------
	Mycobacterium canariasense	WP_062659771	-----------A-----I----R-MA	AD	G--------------------
	Mycobacterium chelonae	WP_070915242	------------A---L-M----R--A	AD	G--------------------
	Mycobacterium chlorophenolicum	WP_048469426	-M----------A---L-V----R--A	AD	G--------------------
	Mycobacterium chubuense	WP_014814542	------------A---L------R-CS	AE	G--------------------
	Mycobacterium conceptionense	WP_064895398	------------A-----V----R--A	AD	G--------------------
	Mycobacterium confluentis	WP_085149054	------------A-----I----R-MA	AD	G--------------------
	Mycobacterium cosmeticum	WP_036402029	-----------A-----I----R-MA	AD	G--------------------
	Mycobacterium diernhoferi	WP_073857609	-----------A-----I----R-MA	AD	G--------------------
	Mycobacterium doricum	WP_085190228	------------A---L-V----Q-MA	AD	G--------------------
	Mycobacterium farcinogenes	WP_036388802	------------A-----V----R--A	AN	G--------------------
	Mycobacterium flavescens	WP_069414666	-M--------------L-M----R--A	AD	G--------------------
	Mycobacterium fortuitum	WP_064896752	-M----------A-----V----R-MA	AD	G--------------------
	Mycobacterium franklinii	WP_070938655	------------A---L-M----R--A	AD	G--------------------
	Mycobacterium gilvum	WP_013472579	----------------L-M----R--A	AD	G--------------------
	Mycobacterium goodii	WP_049747931	----G-------A-----V----R-MA	AD	G--------------------

Group	Species	Accession	Sequence
**Other *Mycobacterium* (0/55)**	*Mycobacterium hassiacum*	WP_005632044	------------------I----R-MA AD G-----V--------------
	Mycobacterium holsaticum	WP_069403779	------------A---R-IQ---R--A AD G--------------------
	Mycobacterium houstonense	WP_066903933	------------A-----V----R-MA AD G--------------------
	Mycobacterium immunogenum	WP_064627910	------------A---L-M----R--A AD G--------------------
	Mycobacterium iranicum	WP_064282001	----------------L-V----R--A AD G--------------------
	Mycobacterium komanii	CRL78367	-M--------------L-M----R--A AD G--------------------
	Mycobacterium litorale	WP_078020506	-----------A-----V----R--A AD G--------------------
	Mycobacterium llatzerense	WP_071285909	------------A-----I----R-MA AD G--------------------
	Mycobacterium malmesburyense	CRL78372	M--------------L-M----R--G AD G--------------------
	Mycobacterium monacense	WP_083044695	S-----------A---L-V----Q-MA AD G--------------------
	Mycobacterium moriokaense	WP_083154391	------------------V----R-MS AD G--------------------
	Mycobacterium mucogenicum	WP_064858500	------------A-----I----R-MA AD G--------------------
	Mycobacterium neoaurum	WP_030134779	-M----------A-----I----R-MA AD G--------------------
	Mycobacterium neworleansense	CRZ15724	------------A-----V----R-MA AD G--------------------
	Mycobacterium obuense	WP_046676516	------------A---L-V----R--A AD G--------------------
	Mycobacterium parafortuitum	WP_083143178	----------------L-M----R--A AD G--------------------
	Mycobacterium peregrinum	WP_064886654	------------A-----V----R-MA AD G--------------------
	Mycobacterium phlei	WP_003886401	-M--------------LNM----R--A AD G--------------------
	Mycobacterium porcinum	WP_075921272	------------A-----V----R-MA AD G--------------------
	Mycobacterium rhodesiae	WP_014208922	------------------V----R-MS AD G--------------------
	Mycobacterium rufum	KGI67249	-M----------A---L-V----R--A AD G--------------------
	Mycobacterium rutilum	WP_083409984	-M--------------L-M----R--A AD G--------------------
	Mycobacterium salmoniphilum	WP_078323704	------------A---L-M----R--A SD G--------------------
	Mycobacterium saopaulense	WP_070909517	------------A---L-M----R--A AD G--------------------
	Mycobacterium septicum	WP_044516579	------------A-----V----R-MA AD G--------------------
	Mycobacterium setense	WP_064876489	------------A-----V----R-MA AD G--------------------
	Mycobacterium smegmatis	WP_011727883	------------A-----V----R--A AD G--------------------
	Mycobacterium tusciae	WP_083124966	-M----------------M----R-MA AD G--------------------
	Mycobacterium vaccae	WP_003929351	----------------L-M----R--A AD G--------------------
	Mycobacterium vanbaalenii	WP_011778897	----------------L-M----R--A AD G--------------------
	Mycobacterium vulneris	WP_065462576	------------A-----V----R-MA AD G--------------------
	Mycobacterium wolinskyi	WP_085146967	----E-------A-----V----R-MA AD G--------------------
**Other bacteria**	*Acetobacter nitrogenifigens*	WP_026396710	S-Q-D-------A----------L--S AR G--------------------
	Acidomonas methanolica	WP_042055982	--Q-D-------A---N------R-VS DD G--------------------
	Agrobacterium vitis	WP_070147431	----D-----------N------A--S DR G----------P---------
	Aliiroseovarius crassostreae	SFU36144	--Q------------S--V--T-Y--S AG G----------P---------
	Azospirillum brasilense	OPH17429	--Q---------C-----I----S--S AS G----------P---------
	Bordetella bronchiseptica OSU054	KAK50849	--Q-D-----------N------R-VS AE G----------P---------
	Burkholderia stagnalis	KVN38720	-H---------A---N-I----Q-MS SA G-----V--------------
	Desulfatirhabdium butyrativorans	WP_028326097	--Q---------A---N----S-I--S EI G--------------------
	Euryhalocaulis caribicus	WP_033310678	-----A---------S--M--T-L--S HA G--------------------
	Geodermatophilus amargosae	SFT83982	----D-----------S-QA---R--A AD G--------------------
	Herbaspirillum rhizosphaerae	WP_050478670	------------A---N-M----T-VS AV G----L---------------
	Kaistia adipata	WP_029074077	-H-------------N------T--S AR G----------P---------
	Lacunisphaera limnophila	WP_069963549	--Q---------A----------Q--S AR G--------------------
	Micromonospora rhizosphaerae	SCL35522	-M--------------N-Q----R-IS AD G-----V--------------
	Millisia brevis	WP_066912149	----D--------V----------R--S AS G----------P---------
	Nocardia cyriacigeorgica	WP_014349322	-H----------V----Q----R--A AD G--------------------
	Pseudomonas lundensis	WP_048376797	-----------C-----V----R-MS AT G----------P---------
	Rhodococcus kunmingensis	WP_068276637	--H-E-------------Q----R--A AD G--------------------
	Rhodoferax ferrireducens	OQW88738	----D------------------A-MS AS G--------------------
	Rhodospirillum rubrum	WP_011388038	--Q-------------N------T--S AA G----------P---------
	Segniliparus rugosus	WP_007470811	------------A---N-V----R-VS AA G--------------------
	Smaragdicoccus niigatensis	WP_018161979	--H---------G-----Q----R-MA AD G-----V--------------
	Streptomyces albulus	WP_045787999	--Q-G-----------R-Q----R--A AD G--------------------
	Xanthobacter autotrophicus	WP_012115479	---------A-------------R--S AT G----------P---------

Supplementary Figure 52

A partial sequence alignment of a conserved region of aldehyde dehydrogenase family protein showing a two amino acid deletion that is specific for members of the *"Tuberculosis-Simiae"* clade and absent in other bacteria.

Group	Species	Accession	136		176
"Tuberculosis-Simiae" Clade (65/65)	*Mycobacterium alsense*	WP_083139967	ALQVPPYVYAHPDDLLATAAG		SPPALLVALDNISDPRNLGA
	Mycobacterium angelicum	WP_083115947	-------N-T-----I-A-LK		-S--M---------------
	Mycobacterium arosiense	WP_083066177	-------N----G--V-A-I-		--------------------
	Mycobacterium asiaticum	WP_065034561	-------N-------V---IE		--------------------
	Mycobacterium avium	WP_062890179	-------N--------EA---		--------------------
	Mycobacterium avium subsp. avium	ETB18859	-------I--------EA---		--------------------
	Mycobacterium bohemicum	WP_085182783	-------D---------A-FD		A-------------------
	Mycobacterium bovis	WP_019283817	-------N---------A-LD		Q----------L--------
	Mycobacterium branderi	WP_083131121	-------E---------A-ME		--------------------
	Mycobacterium canettii	WP_014001819	-------N---------A-LD		Q----------L--------
	Mycobacterium chimaera	WP_072501155	-------N-------VEA-T-		--------------------
	Mycobacterium colombiense	WP_065028599	-------N-------V-D-LA		--------------------
	Mycobacterium conspicuum	WP_085230974	-------D-------I-A-TK		--------------------
	Mycobacterium europaeum	WP_085239762	-------N---------A-MS		--------------------
	Mycobacterium florentinum	WP_085223305	-------N--------T--LQ		--------------------
	Mycobacterium fragae	WP_085198713	-------Q---------A-MD		A-------------------
	Mycobacterium gastri	WP_036409490	----L----------V-A-MD		T-------------------
	Mycobacterium genavense	WP_025736788	-------N--------T--LQ		--------------------
	Mycobacterium gordonae	WP_065047442	-------E-------V-G-LE		N-------------------
	Mycobacterium haemophilum	WP_047316721	-------R---------A-TD		--------------------
	Mycobacterium heidelbergense	WP_083073709	-------N---------A-T-		A-------------------
	Mycobacterium interjectum	WP_085203413	-------R---------A-TD		--------------------
	Mycobacterium intermedium	WP_069419449	-------N-T-------A-LD		V-------------------
	Mycobacterium intracellulare	WP_064938434	-------N--------EA-T-		--------------------
	Mycobacterium kansasii	WP_063466725	-------Q---------A-MD		T-------------------
	Mycobacterium lacus	WP_085157041	-------N---------A-LD		--------------------
	Mycobacterium lentiflavum	CQD21684	-------N--------T--LQ		--------------------
	Mycobacterium leprae	WP_010907675	-------H-V-S-----A-TD		--------------------
	Mycobacterium lepromatosis	WP_045842409	-------H---S-----A-TD		--------------------
	Mycobacterium malmoense	OCB29610	-------N-----------L-		---P----------------
	Mycobacterium mantenii	WP_083095415	-------N-------V-A-IA		--------------------
	Mycobacterium marinum	WP_012396601	-------N---------A-LD		T-------------------
	Mycobacterium nebraskense	WP_047321941	-------S---------A-IN		--------------------
	Mycobacterium palustre	WP_085077749	-------N---------A-QE		T-------------------
	Mycobacterium paraense	WP_085103246	-------N---------A-TD		--------------------
	Mycobacterium paraffinicum	WP_073871363	-------D-----------IN		--------------------
	Mycobacterium paraseoulense	WP_083174477	-------N---------A-L-		--------------------
	Mycobacterium parmense	WP_085270385	-------E---------V-T-		--------------------
	Mycobacterium pseudoshottsii L15	GAQ34727	-------N---------A-LD		T-------------------
	Mycobacterium riyadhense	WP_085251189	-------N-T-------A-IN		A-------------------
	Mycobacterium scrofulaceum	WP_067274166	-------N-----------IN		--------------------
	Mycobacterium sherrisii	WP_069403071	-------N--------G--LQ		--------------------
	Mycobacterium shimoidei	WP_069394761	-----A-D--------QA-MD		--------------------
	Mycobacterium shinjukuense	WP_083050586	-------R-----E---A-LD		A-------------------
	Mycobacterium simiae	WP_061557374	-------H--------GS-LQ		T-------------------
	Mycobacterium szulgai	WP_085670161	-------N-T-----I-A-LK		----M---------------
	Mycobacterium triplex	WP_036471799	-------N--------T--LQ		--------------------
	Mycobacterium tuberculosis	WP_070892668	-------N---------A-LD		Q----------L--------
	Mycobacterium ulcerans str. Harvey	EUA86928	-------N---------A-LD		T-------------------
	Mycobacterium celatum	WP_085167471	-------D---------A-ME		-Q------------------
	Mycobacterium heckeshornense	WP_048891097	-------R--------TA-ME		--------------------
	Mycobacterium kyorinense	WP_045382880	-------D---------A-ME		--------------------
	Mycobacterium noviomagense	WP_083085545	-------D---------A-TE		L-------------------
	Mycobacterium xenopi	WP_085195541	-------R---------A-ME		--------------------
Other *Mycobacterium* (1/61)	*Mycobacterium vulneris*	WP_085292200	-------D------VV-A-LA		--------------------
	Mycobacterium arupense	WP_046191190	-------S---------A-RQ	D	AA------------------
	Mycobacterium engbaekii	WP_085128778	-------A---------A-RK	D	AA------------------
	Mycobacterium heraklionense	WP_065039688	-------N-------I-A-RR	D	VA-P----------------
	Mycobacterium hiberniae	WP_085134032	-------A---------A-RQ	D	AA------------------
	Mycobacterium icosiumassiliensis	WP_067976439	--A----S-------I-A-RR	D	VA-P----------------
	Mycobacterium longobardum	WP_085266508	-------Q---------A-RA	D	VA------------------
	Mycobacterium minnesotense	WP_083027519	-------N---------A-RQ	D	AA------------------
	Mycobacterium senuense	WP_085085717	-------D---------A-RA	D	VA------------------
	Mycobacterium sinense	WP_064924364	-------N---------A-RE	D	VA------------------

**Other *Mycobacterium* (1/61)**	*Mycobacterium terrae*	WP_085261445	-------N---------A-RE E VA-P---------------
	Mycobacterium triviale	ORX02373	-------D---------A-DS D AQ---M-------------
	Mycobacterium abscessus	WP_062878569	---I---K---------R-RA E AQ-----------------
	Mycobacterium abscessus subsp. bolletii	EUA73618	---I---K---------R-RA E AQ-----------------
	Mycobacterium aromaticivorans	WP_036341766	-------N---------A-TS D VE-----------------
	Mycobacterium aurum	WP_048634226	-------S---------D-KS D ATAP---------------
	Mycobacterium bacteremicum	WP_083058506	---------T------KS-QR D -Q-----------------
	Mycobacterium boenickei	WP_077738749	-------D--------KV-RQ S GE-P---------------
	Mycobacterium canariasense	WP_062655953	-------A--------K--QS D -M-----------------
	Mycobacterium celeriflavum	WP_083004703	-------S---------S-RA A GS-----------------
	Mycobacterium chelonae	WP_078284351	-------R---------R-RA E AQ-----------------
	Mycobacterium chubuense	WP_014817859	-----------------E-KR D AAQP---------------
	Mycobacterium conceptionense	CQD23531	-------N--------KA-RD S GE---M-------------
	Mycobacterium confluentis	WP_085155691	-------N--------RA--N D HE---V-------------
	Mycobacterium cosmeticum	WP_036397654	-------A--------KS-QS D -L-----------------
	Mycobacterium diernhoferi	WP_073856830	-------S-I------KA-QR D -M-----------------
	Mycobacterium doricum	WP_085188408	G------D-----------KA D GA-----------------
	Mycobacterium fallax	WP_085096722	-------Q---------E-R- D IT-P---------------
	Mycobacterium flavescens	WP_069415261	---I---S--------SAVTA D GS-----------------
	Mycobacterium fortuitum	WP_064850955	-------D--------KAVRD S GE-G---------------
	Mycobacterium franklinii	WP_070937245	-------K---------R-RA E AQ-----------------
	Mycobacterium gilvum	WP_011892344	-------A--------RD-KS D AA-----------------
	Mycobacterium goodii	WP_049744645	G------Q--------GA-TA D AA-----------------
	Mycobacterium hassiacum	WP_026213240	-------S--------EA-TN D TT-----------------
	Mycobacterium holsaticum	WP_069405959	-----------------A-TA D GS-----------------
	Mycobacterium immunogenum	WP_064630228	-------K---------R-RA E AQ-----------------
	Mycobacterium insubricum	WP_083032188	-------A---------D-RA D VR-P---------------
	Mycobacterium iranicum	WP_064279760	-------D----E---RD-KK D GAQP---------------
	Mycobacterium komanii	CRL75774	-------Q--------SAVTA D GSS----------------
	Mycobacterium litorale	WP_078021121	-------D---------S-TS G GE-----------------
	Mycobacterium mageritense	WP_036432111	-----------------A-KA D GA-----------------
	Mycobacterium malmesburyense	CRL69225	-------Q---------SVTA D GSS----------------
	Mycobacterium moriokaense	WP_083155070	-------S--------K--TT D VT-----------------
	Mycobacterium mucogenicum	WP_064984810	-------Q--------KS-TS D -S-----------------
	Mycobacterium neworleansense	CRZ19141	-------E--------KA-RD A GE-----------------
	Mycobacterium obuense	WP_046362863	-----------------Q-KK D VA-P---------------
	Mycobacterium parafortuitum	WP_083142003	-------D--------RD-KS D AA-----------------
	Mycobacterium peregrinum	WP_064883901	-------N--------KA-RD S GE---M-------------
	Mycobacterium phlei	WP_003888530	-------D--------SA-TA D GS-P---------------
	Mycobacterium porcinum	WP_075920720	-------D--------KA-RE S GE----A------------
	Mycobacterium rhodesiae	WP_083118798	-------N---------A-TS D VE-----------------
	Mycobacterium rufum	KGI70038	-----------------E-KK D AA-P---------------
	Mycobacterium rutilum	WP_083406997	---I---A----N---S---A D GS-----------------
	Mycobacterium salmoniphilum	WP_078324637	-------R---------R-RA E AQ-----------------
	Mycobacterium saopaulense	WP_070913509	-------K---------R-RA E AQ-----------------
	Mycobacterium septicum	WP_044520837	-------N--------KA-RE S GE-----------------
	Mycobacterium setense	WP_039325655	-------N--------K--RD S GE-P-M-------------
	Mycobacterium smegmatis	WP_011731016	G------A-----------RA D AA-----------------
	Mycobacterium thermoresistibile	WP_003925059	-------Q--------ER-TT D TE-----------------
	Mycobacterium tusciae	WP_083129165	-------D-------VKS-TS D VT-----------------
	Mycobacterium vaccae	WP_003930660	-------N--------RD-KS D AA-----------------
	Mycobacterium wolinskyi	WP_067850438	G------N----E---SA-EA D AT-----------------
**Other bacteria**	*Actinoalloteichus cyanogriseus*	WP_026417587	------FD--------TA-EE T GR-P------GVT-------
	Amycolatopsis methanolica	WP_017985382	G-----FE-S-------I-KD S GE-P------GVT-------
	Brevibacterium album	WP_029088215	-------E--------RL--D R YETP------G-T-------
	Corynebacterium falsenii	WP_025402061	G--I---Q--EVE--IDKT-E A GT-G-I------T-------
	Dietzia alimentaria	WP_010540180	G----A-E--D-N--IES-MH S -R----------T-------
	Frankia elaeagni	WP_018637401	--T----S---------A-TR G AQ-G-V----GVT-------
	Geodermatophilus poikilotrophus	SET49610	G------Q--------DL-RD S GR-P-V--M-GVT-------
	Gordonia malaquae	WP_008377451	-----E-R-------M-A-QA S -QRP-I------T-------
	Hoyosella altamirensis	WP_064442355	-------Q----EE-VDASRS S DQ-G-V------T-------
	Kibdelosporangium aridum	WP_084427860	---I---E--------EA-KN S GED-------GVT-------
	Millisia brevis	WP_066912572	---I---R--------QR-LD S RS-G----------------
	Nakamurella panacisegetis	SDO42187	-------D---------R-RD S GR-P-I--V-GVT-------
	Nocardia caishijiensis	WP_067977383	-------R----E--MDRVKS T AE----------T-------

Other bacteria	*Pseudonocardia ammonioxydans*	SFM64337	-------E--------EA--D S GR---A----GVT-------
	Rhodococcus marinonascens	WP_072686873	-------R--------TE-RK H QE-P--------T-------
	Saccharomonospora viridis	WP_037309112	G-----FS--T--E--Q--RE S GE-P------GVT-------
	Skermania piniformis	WP_066469544	-------R--Y------R-RS A GE----------T-------
	Streptomyces coelicoflavus	WP_007390279	V------E----E--V-A-HD E GADP-I----GVT-------
	Tomitella biformata	WP_024795144	---I--FR----E-M--E-KQ A YS-P-I------T-------
	Tsukamurella paurometabola	WP_013125286	---I---S----E---RR--D R GEAPMI------T-------
	Williamsia herbipolensis	WP_045821880	-------R-T--S--MQL-VD S GT-P--------T-------

Supplementary Figure 53

A partial sequence alignment of a conserved region of 23S rRNA (guanosine(2251)-2'-O)-methyltransferase RlmB showing a one amino acid deletion that is specific for members of the *"Tuberculosis-Simiae"* clade and absent in most other bacteria.

			1144		1184
"Terrae" and *"Triviale"* Clades (14/14)	*Mycobacterium algericum*	WP_083036071	LDELLASGEVLWAGAGAPAGN	APAA	DGWITFHTADSAPLTL
	Mycobacterium arupense	WP_046188705	------------S-------S	---S	----S-------A---
	Mycobacterium engbaekii	WP_085127937	------------S-------S	G---	----C----E------
	Mycobacterium heraklionense	OBJ29880	------------S-----V-S	---T	----S-----T-S---
	Mycobacterium hiberniae	WP_085134355	------------S-------S	S---	----C----E------
	Mycobacterium icosiumassiliensis	WP_067970489	------------S-------S	---T	----C-----------
	Mycobacterium koreense	WP_085304130	---V--T-----S---PI--G	T-PT	----V-----T----V
	Mycobacterium kumamotonense	WP_083079602	--------------------S	-L--	----------------
	Mycobacterium longobardum	WP_085266738	-----------------V--S	S--T	----S-----------
	Mycobacterium minnesotense	WP_083023688	------------S-------S	---S	----R-------A---
	Mycobacterium senuense	WP_085087603	------------S--------	----	---V-----E------
	Mycobacterium sinense	WP_064856723	---------------------	----	----------------
	Mycobacterium terrae	WP_085259922	------------T--------	G---	----------------
	Mycobacterium triviale	ODR11270	---V--T-----S---PI--G	T-PT	----V-----T----V
Other *Mycobacterium* (0/84)	*Mycobacterium africanum*	WP_031671011	--------D-T-S---SIS-S		----AL-P-----M--
	Mycobacterium angelicum	WP_083111174	--------D-T-S---SIS-S		----AL-P-E------
	Mycobacterium aromaticivorans	WP_036346036	----------T-S---SIS-S		-------H--T-----
	Mycobacterium asiaticum	WP_065037361	------T---T-S---SIS-S		----A--H---VTM--
	Mycobacterium aurum	WP_048630247	------------S----LSSA		---VA--P--T---S-
	Mycobacterium austroafricanum	WP_036369738	------------S-V-SLSTA		---VA--L-ET---S-
	Mycobacterium avium	WP_062887292	------T---T-S---SIS-S		----AL-PSE------
	Mycobacterium avium subsp. paratuberculosis	EGO38555	------T---T-S---SIS-S		----AL-PSE------
	Mycobacterium bacteremicum	WP_083057506	----------T-S---QIG-S		----A--PS---A---
	Mycobacterium boenickei	WP_077743435	----------M-S---QIG-S		---VA--L-ET-----
	Mycobacterium bohemicum	WP_085182098	------T---T-S---SIS-S		----AL-HS-------
	Mycobacterium bovis	WP_079367537	--------D-T-S---SIS-S		----AL-P-----M--
	Mycobacterium branderi	WP_083129979	----------I-S---SIS-S		----A--A-E------
	Mycobacterium canariasense	WP_062659776	----------T-S---QIGAA		---VV--L-E------
	Mycobacterium canettii	WP_014001670	--------D-T-S---SIS-S		----AL-P-----M--
	Mycobacterium celatum	WP_085168087	----------I-S---SIS-S		----A--A--P-----
	Mycobacterium celeriflavum	WP_083007384	----------T-S---QIGSG		----A--P-E------
	Mycobacterium chlorophenolicum	WP_063840642	------------S---SLSSS		---VA--P--T---S-
	Mycobacterium chubuense	WP_014814528	----------V-S---SLSAA		---VA--P--T---S-
	Mycobacterium conceptionense	WP_064895408	----------M-S---QIG-S		---VA--L--T-----
	Mycobacterium confluentis	WP_085150003	------------S----L-TS		---VA-----T-----
	Mycobacterium cosmeticum	WP_036402017	----------T-S---QIGAA		---VA--L-E------
	Mycobacterium diernhoferi	OJZ62019	----------T-S---QIG-S		----A--PS---S---
	Mycobacterium elephantis	WP_083042602	----------I-S---QIGSG		---VA--L-G---M--
	Mycobacterium europaeum	WP_085241696	------T---T-S---SISAG		---VAL-VG-------
	Mycobacterium farcinogenes	WP_036388806	----------M-S---QIG-S		---VA--L--T-----
	Mycobacterium florentinum	WP_085219981	------A---T-S---SIS-S		----AL-AS-------
	Mycobacterium fortuitum	WP_061262760	----------M-S---QIG-S		---VAL-LVET-----
	Mycobacterium fragae	WP_085198585	----------I-S---SIS-A		---VA----------
	Mycobacterium gastri	WP_036420054	----------I-S---SISAS		----SL-L--------
	Mycobacterium gilvum	WP_011895643	------------S---SLSAA		---VS--P--T---S-
	Mycobacterium gordonae	WP_065046348	------T---T-S---SIS-S		----AL-P--------
	Mycobacterium haemophilum	WP_054880567	------T---T-S----IS-S		----VL-A-E------
	Mycobacterium heckeshornense	WP_071700163	----------M-S---SIS-S		----A--S--T-----
	Mycobacterium holsaticum	WP_069403782	----------T-S---QIGTG		---VA--H-E---M--
	Mycobacterium houstonense	WP_066903666	----------M-S---QIG-S		---VAL-L-ET-----
	Mycobacterium insubricum	WP_083032855	------------S----LP--		---VV--L--T--V--
	Mycobacterium interjectum	WP_085200867	------T---T-S---SIS-S		----AL-VS-------
	Mycobacterium intermedium	WP_069420447	------T---T-----SIS-S		-----L-P-EV--F--
	Mycobacterium iranicum	WP_064281991	--------------S--LSAA		---VS--PS-T---S-
	Mycobacterium kansasii	WP_063466820	----------S-S---SISAS		----SL-L--------
	Mycobacterium komanii	CRL78375	----------M-S---QISAG		----A--S-E------
	Mycobacterium kubicae	WP_085072779	------T---T-S---SIS-S		----AL-P-ET-----
	Mycobacterium kyorinense	WP_065014968	----------I-S---SIS-S		----I--A--A-----
	Mycobacterium lacus	WP_085159387	----------T-S---SIS-S		----AL-P-----M--
	Mycobacterium lentiflavum	CQD07795	--Q---A---T-S---SIS-S		----AL-PS-------
	Mycobacterium llatzerense	WP_071285963	----------T-S---QI--S		---VV--R--T---S-
	Mycobacterium mageritense	WP_036428659	----------T-S---QIGAG		---VA--L--------
	Mycobacterium malmesburyense	CRL78380	----------T-S---QIGSG		----A--S-ET-----
	Mycobacterium malmoense	WP_065517097	----------T-Q---TISAS		---VAL-VGE------

Other *Mycobacterium* (0/84)	*Mycobacterium marinum*	WP_012393116	------T---T-----SIS-S	---V-L-L--------
	Mycobacterium mucogenicum	WP_064858502	----------M-S---QIGS-	---VV--R--T---S-
	Mycobacterium mungi	WP_064319914	--------D-T-S---SIS-S	----AL-P-----M--
	Mycobacterium nebraskense	WP_085165614	------T---T-S---SISAS	---VAL-VS-------
	Mycobacterium neworleansense	CRZ15739	----------M-S---QIG-S	---VA--L--T-----
	Mycobacterium noviomagense	WP_083087098	----------M-S---TIS--	----A--AS-T-----
	Mycobacterium obuense	WP_046364769	------------S----LSSA	---VA--P--T---S-
	Mycobacterium orygis	WP_003417202	--------D-T-S---SIS-S	----AL-P-----M--
	Mycobacterium paraense	WP_085096127	------T---T-S---SIS-S	----AL-AS-------
	Mycobacterium paraffinicum	WP_073876210	------T---T-S---SISA-	---VAL-VS-------
	Mycobacterium parafortuitum	WP_083143181	---------L------SLS-A	---VS--P--T---S-
	Mycobacterium parascrofulaceum	WP_007168045	----------T-Q---TISAS	---VAL-VGE------
	Mycobacterium paraseoulense	WP_083169835	------T---T-S---PISAG	---VAL-VS-------
	Mycobacterium peregrinum	WP_064886663	----------M-S---QIGST	---V---L-ET-----
	Mycobacterium porcinum	WP_069425835	----------M-S---QIG-S	---VA--L-ET-----
	Mycobacterium rhodesiae	WP_083121541	----------T-S---SIS-S	-------H--T-----
	Mycobacterium rufum	KGI70690	------------S----LSSA	---VA--P--T---S-
	Mycobacterium saskatchewanense	WP_085255937	------T---T-S-C-SIS-S	---VAL-PS-------
	Mycobacterium scrofulaceum	WP_067270338	------T---T-S---SISAS	----AL-VS-------
	Mycobacterium septicum	WP_044516549	----------M-S---QIGSS	---V---L-ET-----
	Mycobacterium setense	WP_064876235	----------M-S---QIG-S	---VS--L-ET-----
	Mycobacterium sherrisii	WP_085166478	------A---T-S---SIS-S	----AL-PS-------
	Mycobacterium shimoidei	WP_069395255	----------I-S---SISAV	----A-----------
	Mycobacterium shinjukuense	WP_083048662	------T---T-S----IS-S	----AL-P-A------
	Mycobacterium simiae	WP_06155847	------A---T-S---SIS-S	----AL-AS------
	Mycobacterium szulgai	WP_085671658	----------T-S---SIS-S	----AL-P-E------
	Mycobacterium timonense	WP_083186877	------T---T-S---SIS-S	----AL-PSE------
	Mycobacterium triplex	WP_085200684	------T---T-S---SIS-S	----VL-AG-------
	Mycobacterium tuberculosis	WP_063738150	--------D-T-S---SIS-S	----AL-P-----M--
	Mycobacterium vaccae	WP_003929341	------------S---SLSAA	---VA--L-ET---S-
	Mycobacterium vanbaalenii	WP_011778886	------------S-V-SLSTA	---VA--L-ET---S-
	Mycobacterium vulneris	WP_065462585	----------M-S---QIG-S	---VA--L-ET-----
	Mycobacterium xenopi	WP_085196342	----------M-S---SISAS	----A--S--T-----
	Mycobacterium yongonense	WP_020823425	------T---T-S---SIS-S	----AL-AGE------
Other bacteria	*Arthrobacter enclensis*	WP_058268864	----M-T-----S----LP--	----SM-L----E---
	Gordonia malaquae	WP_008379860	----------C-S-H-SIS-S	---VAL-L--A-----
	Hoyosella altamirensis	WP_083962215	----TS-----------LSTR	----SL-L--T--V--
	Nakamurella lactea	WP_084614169	------T-------H----AD	----SL-P----A---
	Neomicrococcus aestuarii	WP_071894185	------------Q-N-SL---	---VS--VREH-----
	Nocardia otitidiscaviarum	WP_029925647	------T-----S-H--ITAK	----AL-P--Q-----
	Nocardioides dokdonensis	WP_084527652	----T-----I---H-TLP-S	---VSL-L----D---
	Pseudarthrobacter equi	SDS84839	----M-A-----S----LP--	----SL-L----E---
	Rhodococcus fascians	WP_032366663	----TST-------H--ISSK	---VSL-M--T-----
	Sinomonas humi	WP_043119207	----M----I--T----LP--	---VSL-V-E------
	Streptomyces rapamycinicus NRRL 5491	AGP54609	----ST----V------LP-K	---V-L-L--T---L-
	Tetrasphaera elongata Lp2	CCH70793	---V--A-----V-H--TG-D	---LS--L----H--M
	Tomitella biformata	WP_024794816	----T-T---R-S---QI-AK	---VAL-L--------
	Verrucosispora sediminis	SFD61662	----C------------IS-G	---VSLAY------L-

Supplementary Figure 54

Detailed sequence information for the four amino acid insertion found in ATP-dependent helicase, which is shown in Figure 9A. This insertion is specific for members of the “*Terrae”* and *“Triviale”* clades and absent in other bacteria.

Group	Species	Accession	290		324
"Terrae" and *"Triviale"* Clades (14/14)	*Mycobacterium algericum*	WP_083035840	AGATVFLVPAENCYEARSDPN		GLQLIKVDSLAQAV
	Mycobacterium engbaekii	WP_085129315	----M-----D------A-NK		--------------
	Mycobacterium heraklionense	WP_064887978	----------D------N-DK		----V---------
	Mycobacterium hiberniae	WP_085133937	----M-----D------A-NK		--------------
	Mycobacterium icosiumassiliensis	WP_067970899	----------D------N-NK		----V---------
	Mycobacterium koreense	WP_085302488	---K-----VD-------E-H		--T-V--ET-D-T-
	Mycobacterium kumamotonense	WP_065287460	----------D--------N-		--------------
	Mycobacterium longobardum	WP_085264344	----------D-----MA-N-		----V---T-----
	Mycobacterium minnesotense	WP_083027534	----------D--------DK		----V---------
	Mycobacterium nonchromogenicum	WP_085137960	----------D------N-DK		----V---------
	Mycobacterium senuense	WP_085081727	--------------------		------I-------
	Mycobacterium sinense	WP_064854793	------------------N-		--------------
	Mycobacterium terrae	WP_085261980	------------------N-		--------------
	Mycobacterium triviale	WP_085111465	---DY----VD-------ETK		--T---IEN-----
Other *Mycobacterium* (0/>100)	*Mycobacterium africanum*	WP_031661079	---M---K-----S--SP	P	--K-V--ET-S---
	Mycobacterium alsense	WP_083140570	-------K-----S-ANE	P	--R-V--ET-QG--
	Mycobacterium angelicum	WP_083111070	-------K-----T--IP	S	--K-V--ET-GS--
	Mycobacterium aromaticivorans	WP_036340072	---S---------D--K-MHD	D	-ME-V---T-SS--
	Mycobacterium arosiense	WP_083065536	----------K-----S--NP	S	--R-V--ET-G---
	Mycobacterium asiaticum	WP_065035339	-------K-----V-QIP	A	--K-V--ET-GS--
	Mycobacterium aurum	WP_048630390	----------D--A---TGDA	E	-IE-L---T-EH-I
	Mycobacterium avium	WP_084020247	----------K-----S--NP	S	--R-V--ET-----
	Mycobacterium avium subsp. hominissuis	BAN32624	----------K-----S--NP	S	--R-V--ET-----
	Mycobacterium avium subsp. paratuberculosis	EGO38478	----------K-----S--NP	S	--R-V--ET-----
	Mycobacterium bacteremicum	WP_083057538	----I-----D--A--LTARE	D	----V---T--T--
	Mycobacterium boenickei	WP_077743880	----------D--T----A-Q	D	-MD-VR-ET-T---
	Mycobacterium bohemicum	WP_085181998	----------K-----N--NP	S	-MR-V--ET-S---
	Mycobacterium bovis	WP_024456357	-------K-----S--SP	P	--K-V--ET-S---
	Mycobacterium branderi	WP_083129859	----------K-----S--KL	P	----V---T-G---
	Mycobacterium canariasense	WP_062654530	----------D--D--LTARD	D	R---V---T-TT--
	Mycobacterium canettii	WP_015291268	-------K-----S--SP	P	--K-V--ET-S---
	Mycobacterium celatum	WP_062540816	----------K-----S--KL	P	----V--EN-G---
	Mycobacterium chelonae	WP_070915105	----------A--A--KT-GG	Q	--TMV--GT--E--
	Mycobacterium chlorophenolicum	KMO75862	---S------D--D--K-ADE	K	DIE-L---T-EH-I
	Mycobacterium chubuense	WP_041782689	---------TD--D--KTADE	K	-IE-L---T-S--I
	Mycobacterium colombiense	WP_064877167	----------K-----S--NP	S	--R-V--ET-G---
	Mycobacterium confluentis	WP_085148633	-------A-----D----GAQ	D	-ME-V--ET--G--
	Mycobacterium conspicuum	WP_085231380	----------K-----T-NRQ	A	--R-V--ET-G---
	Mycobacterium cosmeticum	CDO10254	----------D--D--LTARD	D	R---V---T-TA--
	Mycobacterium diernhoferi	WP_073857812	------M---D--S--LTAHE	D	----V---T-TT--
	Mycobacterium doricum	WP_085190280	---SI-----D--A--K---Q	D	--E-V--GT-TD--
	Mycobacterium europaeum	WP_085241760	----------K-----N--NP	S	--R-V--ET-S---
	Mycobacterium fallax	WP_085092482	---E------G--A--ETAH-	G	-MT-V---T-TS--
	Mycobacterium farcinogenes	CDP84270	----------D--T----A-Q	D	-MD-VR-ET-T---
	Mycobacterium flavescens	WP_069416780	----L-----D--A--LTA-Q	D	--E-V---T--T--
	Mycobacterium florentinum	WP_085223063	----------K-----SA-NP	H	--R----ET-G---
	Mycobacterium fortuitum	WP_061262773	----------D--T----A-Q	D	-ME-V---T-S---
	Mycobacterium fragae	WP_085195830	----------K-----S--KL	P	----V---D-R---
	Mycobacterium franklinii	WP_070938761	----------A--A--KT-GG	Q	--T-V--GT-TE--
	Mycobacterium gastri	WP_036412751	----------K-----AA-TP	R	--R-V--ET-G---
	Mycobacterium genavense	WP_025735718	----------K-----A--NP	H	--R-V--ET-G---
	Mycobacterium gilvum	WP_011895491	----------D--A---TADA	D	-IE-L--ET-E---
	Mycobacterium goodii	WP_049749117	---EE-----D--T----A-Q	D	--G-V--ET-EG--
	Mycobacterium gordonae	WP_065047226	S------K-----M--IP	S	--K-V--ET-GS--
	Mycobacterium haemophilum	WP_054880497	----------K-----S--NR	T	--R-V--E--S---
	Mycobacterium hassiacum	WP_005625958	-------------A--KAADH	D	--E----ET-D---
	Mycobacterium heckeshornense	WP_048891308	----------K-----NA-SV	P	----V---T-D---
	Mycobacterium heidelbergense	WP_083073124	----------K-----N--NP	S	-MR-V--ET-G---
	Mycobacterium holsaticum	WP_069403957	----------D--E--K-V-Q	D	--E-L---T-TT--
	Mycobacterium houstonense	WP_066903377	----------D--T----A-Q	D	-ME-V---T-T---
	Mycobacterium immunogenum	WP_064628009	----------A--A--KT-GG	Q	--T-V--GT-TE--
	Mycobacterium insubricum	WP_083030761	----------D--S--STAER	G	DMT-V---T-TS--
	Mycobacterium interjectum	WP_066908391	----------K-----T--NP	S	--R-V--ET-S---
	Mycobacterium intermedium	WP_069419055	-------K--V--T--IP	D	--K-V--ET-SS--

Other *Mycobacterium* (0/>100)	*Mycobacterium iranicum*	WP_064283212	----A-----D--A--KTADA A -IE-L---T-EH-I
	Mycobacterium kansasii	WP_063468879	-------K-----G--TP Q --R-V--ET-G---
	Mycobacterium komanii	CRL78142	----L-----D--E--KTA-Q D -ME-V--GT-SE--
	Mycobacterium kyorinense	WP_065016589	----------K--------QL P -ME-V---N-G---
	Mycobacterium lacus	WP_085157003	----------K-----S--NP S -MR-V--ET-S---
	Mycobacterium lentiflavum	CQD08520	----------K-----T--NP H --R----ET-G---
	Mycobacterium leprae	WP_010907855	-------K--------NR T --R-V--E--S---
	Mycobacterium lepromatosis	WP_045842623	----------K--D---F-NR T --R-V--EN-S---
	Mycobacterium liflandii 128FXT	AGC61492	S------K-----A--TP S --R-V--ET-G---
	Mycobacterium litorale	WP_078020416	---S------D--D--K-MRD D KME-V---T-TG--
	Mycobacterium llatzerense	WP_071286861	------M------A--V-AHL D --G----EN-TG--
	Mycobacterium mageritense DSM 44476	CDO19876	----------D--T----A-Q D ----VR-ET-T-T-
	Mycobacterium malmesburyense	CRL77526	S---I-----D--E--KTA-Q D -ME-V--GT-SE--
	Mycobacterium malmoense	WP_065517357	----------K-----N--NP S -MR-V--ET-S---
	Mycobacterium mantenii	WP_083096274	----------K--F--S--NP S --R-V--ET-G---
	Mycobacterium marinum	WP_012393236	S------K-----A--TP S --R-V--ET-G---
	Mycobacterium moriokaense	WP_083154150	----I--------E--K-ANE D --E-VR-ET-D---
	Mycobacterium mucogenicum	WP_064860813	------M------A--V-AHL D --G----EN-SG--
	Mycobacterium nebraskense	WP_046186416	----------K-----N--NP S --R-V--ET-G---
	Mycobacterium neoaurum	CDQ46084	------M------A--LTARE D SM--V---T-TT--
	Mycobacterium neworleansense	CRZ15540	----------D--T----ALQ D -MD-VR-ET-T---
	Mycobacterium noviomagense	WP_083086257	---S------K-----NA-NL P ----V---T-G---
	Mycobacterium novocastrense	GAT12248	---I-----D--E--KTA-Q D -ME-VR-ET-P---
	Mycobacterium obuense	KMO80356	---S------D--D--T-ADE K -IE-----T-TH-I
	Mycobacterium palustre	WP_085077997	-------K-----A--NP S --R-V--ET-S---
	Mycobacterium paraense	WP_085103822	-------K-----S--NP S --R-V--ET-S---
	Mycobacterium paraffinicum	WP_073876026	----------K-----N--NP S --R-V--ET-S---
	Mycobacterium parafortuitum	WP_083143198	----A-----D--A--KTADV D -IE-L---T-EH--
	Mycobacterium paraseoulense	WP_083170049	----------K-----N--NP S --R-V--ET-G---
	Mycobacterium parmense	WP_085267730	----------K-----N--NP S -MR-V--ET-S--I
	Mycobacterium peregrinum	WP_064878840	----------D--T----A-Q D -ME-V---T-TH--
	Mycobacterium phlei	WP_003888766	---SL--------E--K-A-Q D --E-VR-ET-E---
	Mycobacterium porcinum	WP_075921506	----A-----D--T----A-Q D -MD-VR-ET-T---
	Mycobacterium pseudoshottsii L15	GAQ36873	S------K-----A--TP S --R-V--ET-G---
	Mycobacterium rhodesiae	WP_083121064	---S---------D--K-MRD D -ME-V---T-NT--
	Mycobacterium riyadhense	WP_085249735	-------K-----S--DP P --R-V--ET-S---
	Mycobacterium rufum	KGI70726	---S------D--D--K-ADE Q -IE-L---T-EH-I
	Mycobacterium rutilum	SEH90113	----M-----D--E--KTA-Q E --E-V---T--T--
	Mycobacterium salmoniphilum	WP_078330911	----------A--A--KT-GG Q --T-V--GT-TE--
	Mycobacterium saopaulense	WP_070909626	----------A--A--KT-GG Q --T-V-AGT-TE--
	Mycobacterium saskatchewanense	WP_085257922	-------K-----N--NP S --K-V--ET-GE--
	Mycobacterium scrofulaceum	WP_067277588	----------K-----N--NP S --R-V--E--G---
	Mycobacterium septicum	WP_044516824	---S------D--T----E-Q D -MD-V---T-T---
	Mycobacterium setense	WP_039318556	---S------D--T----A-Q D -MD-VR-ET-T---
	Mycobacterium sherrisii	WP_069402475	----------K-----A--NP D --R----ET-----
	Mycobacterium shimoidei	WP_069396544	----------K-----N--KL A ----V--ET-G---
	Mycobacterium shinjukuense	WP_083049850	-------K-----S--SP P --K----ET-G---
	Mycobacterium simiae	WP_061558587	----------K-----A--NP H --R-V--ET-S---
	Mycobacterium smegmatis	WP_011728042	---E------D--T----A-R D --E-V--ET-EG--
	Mycobacterium szulgai	WP_085674306	----------K-----T--IP S --K-V--ET-GS--
	Mycobacterium thermoresistibile	WP_003927922	----------V--K-AQR D -ME-VR-ET-DD--
	Mycobacterium timonense	WP_083187475	----------K-----S--NP S --R-V--ET-----
	Mycobacterium triplex	CDO87446	----------K-----A--NP H --R-V--ET-G---
	Mycobacterium tuberculosis	WP_070890986	-------K-----S--SP P --K-V--ET-S---
	Mycobacterium tusciae	WP_083125070	----I-----D--E--KTAHQ D --E-V--EN-DG--
	Mycobacterium ulcerans str. Harvey	EUA89577	S------K-----A--TP S --R-V--ET-G---
	Mycobacterium vaccae	WP_003929185	----------D--A--TTVDD D -IE-L---T-ET-I
	Mycobacterium vanbaalenii PYR-1	ABM12635	----A-----D--A--KTADA D -IE-L---T-EH--
	Mycobacterium vulneris	WP_065516755	----------D--T----A-Q D -MD-VR-ET-T---
	Mycobacterium wolinskyi	WP_067856411	----------D--T----A-Q D --E-V--ET-TG--
	Mycobacterium xenopi	WP_003920807	----------K-----NA-NV A ----V---T-G---
Other bacteria	*Amycolatopsis lurida NRRL 2430*	AJK58677	----D-----H--A--KTAAP E --N----ST-DE-I
	Corynebacterium freneyi	WP_035123914	G--EM-----D--S--LTADA G DMK-VS-GT-GD--
	Dietzia timorensis	WP_082908530	---A-----D--A--MT--P E -ID-V--GT-DD--
	Gordonia hirsuta	WP_024332489	---------D--A--LT-VP D --E-----T-GG--

Other bacteria

Lentzea violacea	SDM86515	----A-----D--AD-KQQAP A D-K----EN-TD--
Micromonospora eburnea	SCL65448	---K------D--A--VNN-Q P --P-L--T--ED-L
Millisia brevis	WP_066913251	---------SD--A--EAAAP D N---V---T-DG--
Nakamurella lactea	WP_029135577	----------P--Q--LTRVP S --E-V--ST-DD-M
Nocardia asiatica	WP_083887703	---ET-----A--N--KQRIP E --R-VR-E--TG--
Pseudonocardia asaccharolytica	WP_028930382	-------------A----TTP D --E-VR--D--D--
Rhodococcus enclensis	KSU80719	---ET-----Q--D--KQNAP D --R-V---N-PG--
Saccharopolyspora hirsuta	SEG77286	----T-----D--A--KAQAP E ----A--GT-QE-T
Segniliparus rotundus	WP_013139042	--------SD--S--ST-IP A -MRVV--AT-HD--
Skermania piniformis	WP_066467605	----T-----D--S---QQ-P A --R----EK-DD--
Streptomyces mirabilis	WP_037710679	--------EDE-AD-K-ELP K --R--P-T--KG--
Thermocrispum agreste	WP_051309847	------------A--AKSAP D --R-V--SK--E--
Tsukamurella pseudospumae	WP_068571738	--S------R--S--KN--P K ----V--EN-DG--
Williamsia muralis	WP_062798765	---EA----SG--A--K--TP D --T-V---T-QN--

Supplementary Figure 55

A partial sequence alignment of a conserved region of PDZ domain-containing protein showing a one amino acid deletion that is specific for members of the "*Terrae*" and "*Triviale*" clades and absent in other bacteria.

Clade	Species	Accession	199	(boxed)	263
"Terrae" and *"Triviale"* Clades (13/13)	*Mycobacterium algericum*	WP_083035874	PGYRLLLRATRTEGRLDLSG	PGS	LDAEVPDWRGRQAWACGP
	Mycobacterium arupense	WP_046189161	--------T--DQ---E---	-EV	--G------V--T-----
	Mycobacterium engbaekii	WP_085129349	--------T--DQ---Q-R-	-EA	--GA-----E--T-----
	Mycobacterium heraklionense	WP_064888029	--------T--DQ---E-R-	-ET	--V------E--T-----
	Mycobacterium hiberniae	WP_085133974	--------S--DQ---Q-R-	-EA	--SA-----E--T-----
	Mycobacterium icosiumassiliensis	WP_067970714	--------T--DQ---E-R-	-ET	---------E--T-----
	Mycobacterium kumamotonense	WP_065287492	------F----SQ-------	-DT	------------T-----
	Mycobacterium longobardum	WP_085266839	--------T--DS---E-R-	-ET	--R------S--------
	Mycobacterium minnesotense	WP_083027581	--------T--DQ---E-R-	-EV	--G------V--T-----
	Mycobacterium senuense	WP_085085001	--------------------	--P	--V---------------
	Mycobacterium sinense	WP_064855456	--------T--AQ-------	-D-	------------T-----
	Mycobacterium terrae	WP_085259037	------F----SA-------	-DA	------------------
	Mycobacterium triviale	WP_085110731	----S--E--VA-----A-	-AA	---A-----Q-ET-----
Other *Mycobacterium* (0/>100)	*Mycobacterium abscessus*	WP_074332872	-S-HYR--T-K-Q----F-S		-A------AR--T-----
	Mycobacterium abscessus subsp. bolletii	SLI09751	-S-HYR--T-K-Q----F-S		-A------AQ--T-----
	Mycobacterium acapulcensis	WP_066810358	---RT-----Q------R		--D------E--T-----
	Mycobacterium alsense	WP_083138628	-----QV-E--SQ-----AA		--R------E--T-----
	Mycobacterium angelicum	WP_083111112	-----AV-E--A----E--Q		IGQL-G---E--T-----
	Mycobacterium aromaticivorans	WP_036340193	----TV-T----------R		--DL-----E--T-----
	Mycobacterium arosiense	WP_083066649	---MR--E---Q------E		--HD-----E-HT-----
	Mycobacterium asiaticum	WP_065034992	-----T---S-AQ------R		--QA-----D--V-----
	Mycobacterium aurum	WP_048630350	----Q------Q-----DS		--ET-----E--T-----
	Mycobacterium avium	WP_009978714	-----Q--E---R------L		--R------E--T-----
	Mycobacterium avium subsp. avium 2285	EUA39186	-----Q--E---R------L		--R------E--T-----
	Mycobacterium avium subsp. hominissuis	ETB32554	-----Q--E---R------L		--R------E--T-----
	Mycobacterium avium subsp. paratuberculosis	AAS05894	-----Q--E---R------L		--R------E--T-----
	Mycobacterium bacteremicum	WP_083057364	----ME--T---Q-----HR		-AEV----QD--------
	Mycobacterium boenickei	WP_077743524	--------S---------AQ		IGDV-----E--------
	Mycobacterium bohemicum	WP_085182047	--------E---R-----ES		--H------E-HT-----
	Mycobacterium bovis	WP_058222295	-----SV-E--AQ-----TR		IGQQ-----E--T-----
	Mycobacterium branderi	WP_083129903	--Q-RV-T--SQ------R		--D--A---D--T-----
	Mycobacterium brisbanense	WP_062831603	-----H--S----------R		-AEV----QD--------
	Mycobacterium canettii	WP_014001633	-----SV-E--AQ-----TR		IGQQ-----E--T-----
	Mycobacterium caprae	WP_075744587	-----SV-E--AQ-----TR		IGQQ-----E--T-----
	Mycobacterium celatum	WP_062540225	--Q-RV-T--SQ------R		--DQ----KE-HT-----
	Mycobacterium celeriflavum	WP_083004910	--E-R--S---Q----VTR		--D------E--T-----
	Mycobacterium chelonae	OHU26712	-S-HYR--T---Q------A		-AS-----AQ--T-----
	Mycobacterium chimaera	WP_069953954	-----E--E---Q------A		---D-----E--T-----
	Mycobacterium chlorophenolicum	WP_048470734	----TV-----Q-----TR		--EV-----N--T-----
	Mycobacterium chubuense	WP_083119160	----H-QL-QED-KVE-TE		--EI-S--QE-S--V---
	Mycobacterium colombiense	WP_064877182	----MR--E---Q------V		--R------E--T-----
	Mycobacterium conceptionense	WP_085140974	------V-S--I-------S		-GDV-A---E--------
	Mycobacterium confluentis	WP_085148757	-S---R------------DR		--D------E--T-----
	Mycobacterium conspicuum	WP_085231443	-----RV-E--S---F--AR		-SQ------D-HT-----
	Mycobacterium diernhoferi	WP_073857731	----MV--T---Q-----DR		-AEV-----E--------
	Mycobacterium doricum	WP_085190101	----H--T---Q---N--R		--EL-----E--T-----
	Mycobacterium europaeum	WP_085241770	-----T--E--SQ-----AL		--R------D-HT-----
	Mycobacterium fallax	WP_085095195	-----RV----SD---N-DR		--D------E--V-----
	Mycobacterium farcinogenes	WP_036388719	------V-S----------S		-GDV-A---E--------
	Mycobacterium flavescens	WP_069415930	-----RI---------S-DR		-AD------E--T-----
	Mycobacterium florentinum	WP_085220046	-----QV-E--AQ--F--TR		--D------E--T-----
	Mycobacterium fortuitum	WP_064925443	------V-S----------Q		IGDV-----E--------
	Mycobacterium fragae	WP_085195913	--Q-R--T--SH-----NR		---------E--T-----
	Mycobacterium franklinii	WP_070938714	-S-HYR--T-K-Q------A		-AT-----AQ--T-----
	Mycobacterium gastri	WP_036410749	----T--K---------AR		--EQ-----D--T-----
	Mycobacterium genavense	WP_025735765	----QV-E---Q-----AR		--D------Q--T-----
	Mycobacterium gilvum	WP_011895534	----E------Q----VGR		--DV-----D--T-----
	Mycobacterium gordonae	WP_065047320	-----SV-E--AA------R		--EQ-----E--T-----
	Mycobacterium haemophilum	WP_054880536	----RV-Q---------AR		-GE-----SE--T-----
	Mycobacterium heckeshornense	WP_048891221	--H-R--T---Q-----AR		--ED-----E-RT-----
	Mycobacterium heidelbergense	WP_083073161	-----R--E---Q-----AS		--EQ-----E--T-----
	Mycobacterium holsaticum	WP_069404405	--S-HV-T--EQ----M-R		-AD-----HE--T-----
	Mycobacterium houstonense	WP_066903473	------V-S----------R		-GDV-A---E--------
	Mycobacterium immunogenum	WP_064627972	-S-HYR----K-Q------S		-VS-----AQ--T-----

Other *Mycobacterium* (0/>100)	*Mycobacterium indicus pranii MTCC 9506*	AFS16112	-----Q--E---Q------A	---D-----E--T-----
	Mycobacterium insubricum	WP_083030692	-S------E---Q---A-DR	--EV-----E--------
	Mycobacterium interjectum	WP_066908254	-----AV-E--RQ-----AT	--H------E--T-----
	Mycobacterium intermedium	WP_069419071	-----TV-E---A------R	-GD------E--T-----
	Mycobacterium intracellulare	WP_064939689	-----Q--E---Q------A	---D-----E--T-----
	Mycobacterium iranicum	OAN42065	--G-H-HL-KDQ-KV-FDR	F-EL-----D-S--V---
	Mycobacterium kansasii	WP_063468206	-----KV-K---------AR	-GD------D--T-----
	Mycobacterium komanii	CRL78234	----RV----SQ--M---R	--D------E--T-----
	Mycobacterium kubicae	WP_085072824	-----S--E--AQ------R	--EA-----D--T-----
	Mycobacterium kyorinense	WP_065016629	----RV-T--DQ------R	--T-----QE--T-----
	Mycobacterium lacus	WP_085161162	----RV-E---------AR	--Q-L----D--T-----
	Mycobacterium lentiflavum	CQD08141	-----QV-E--AQ-----AR	--DQ-----E--T-----
	Mycobacterium liflandii	WP_015354866	-----SV----SQ------R	IGEQ-----E--T-----
	Mycobacterium litorale	WP_078020453	----TV-T-K-Q-----QR	--EA-----Q--------
	Mycobacterium llatzerense	WP_071286867	-S-Q-RV-----D-----AK	--D--A---T-E------
	Mycobacterium mageritense	WP_036428409	-----Q--T---Q------R	-ADV-A---D--T-----
	Mycobacterium malmesburyense	CRL72429	----RV-----A-----A-	--D------E--------
	Mycobacterium malmoense	WP_065445066	-----A--E---Q-----AL	--H------E-HT-----
	Mycobacterium mantenii	WP_083097918	----MR--E---Q------M	--EQ-----E--T-----
	Mycobacterium marinum	WP_012393181	-----SV----SQ------R	IGEQ-----E--T-----
	Mycobacterium moriokaense	WP_083154254	--Q-RV-----D------R	--DV-----E--T-----
	Mycobacterium mucogenicum	WP_064980096	-S-Q-RV------------R	--E--A---T-E------
	Mycobacterium nebraskense	WP_046186261	--------E---Q-----AL	--H------E-HT-----
	Mycobacterium neoaurum VKM Ac-1815D	AHC23381	-D---H-QM-DRM-H---DR	-ADI----TE-PT-----
	Mycobacterium neworleansense	CRZ15605	--------S---------AH	IGDV----QE--------
	Mycobacterium noviomagense	WP_083086342	--Q-R--T---Q-----AH	--E--A---N--T-----
	Mycobacterium novocastrense	WP_067395853	----RT----SQ------R	--D------E--T-----
	Mycobacterium obuense	WP_046364827	-H-V--S--SQ-----AR	--EV-----D--T-----
	Mycobacterium palustre	WP_085080151	-----R--E--KQ-----AS	--H------E--T-----
	Mycobacterium paraense	WP_085095982	-----AV-E--RQ-----AT	--H------E--T-----
	Mycobacterium paraffinicum	WP_073876252	---Q-K--E--AQ-----AL	--H------E-HT-----
	Mycobacterium parafortuitum	WP_083143094	-----Q------------DR	--EV-----E--T-----
	Mycobacterium parascrofulaceum	WP_007168116	-----A--E---Q-----AL	--H------E-HT-----
	Mycobacterium paraseoulense	WP_083170092	--------E---Q-----AR	--H------E-HT-----
	Mycobacterium parmense	WP_085268023	-----Q--E--RQ-----GS	IEQD-----E--T-----
	Mycobacterium phlei	WP_003886309	-T-R--F------F--AK	---------E--V-----
	Mycobacterium porcinum	WP_069426491	--------S---------AQ	IGDV-----E--------
	Mycobacterium pseudoshottsii L15	GAQ37231	-----SV----SQ------R	IGEQ-----E--T-----
	Mycobacterium rhodesiae	WP_014208843	-----RS-----Q------R	--D------E--V-----
	Mycobacterium riyadhense	WP_085250057	-----SV-Q--V-------R	--WA----CE--T-----
	Mycobacterium rufum	KGI67177	----H-QL-QRD-KIE-GE	--RV-E---D-S--V---
	Mycobacterium rutilum	WP_083409888	----QV-----------AR	-ADD-----E--T-----
	Mycobacterium salmoniphilum	WP_078323761	-S-HYR--T-K-Q------S	-AS-----AQ--T-----
	Mycobacterium saopaulense	WP_070909574	-S-HYR----K-Q------S	-G------AQ--T-----
	Mycobacterium saskatchewanense	WP_085254284	--------E---Q-----AS	--QQ-----D--T-----
	Mycobacterium scrofulaceum	WP_067268542	-----K--E--SQ-----AL	--H------E-HT-----
	Mycobacterium septicum	WP_044516717	--------S---------AH	IGDV-----E--T-----
	Mycobacterium setense	WP_039317603	--------S---------AR	IGEV-----E--------
	Mycobacterium sherrisii	WP_069402597	-----Q--E---Q-----GR	--D------A--T-----
	Mycobacterium shimoidei	WP_069396584	--QFR--T--SQ------R	-EG------D--T-----
	Mycobacterium shinjukuense	WP_083047355	-----TV-E--V-------R	-GRL-----A--T-----
	Mycobacterium simiae	WP_061558555	-----Q--E---Q-----GR	---A-----D--T-----
	Mycobacterium smegmatis	WP_011727980	-----RI-S---------ER	IAD------E--V-----
	Mycobacterium szulgai	WP_068029142	-----S--E--AQ------R	-HEA-----D--T-----
	Mycobacterium thermoresistibile	WP_003927964	-S--FR--T----------R	--D--G---S--T-----
	Mycobacterium triplex	WP_036467287	-----QV-E---R-----AR	--D------Q--T-----
	Mycobacterium tuberculosis	WP_070900871	-----SV-E--VQ-----TR	IGQQ-----E--T-----
	Mycobacterium tusciae	WP_083125028	----R-------------R	--H------E--V-----
	Mycobacterium ulcerans	WP_011740523	-----SV----SQ------R	IGEQ-----E--T-----
	Mycobacterium vaccae	WP_003929127	----RV----------IDR	--EV-----E--T-----
	Mycobacterium vulneris	WP_065462499	--------S---------TQ	IGDV-----E--------
	Mycobacterium wolinskyi	WP_085147168	-----Q--R---------HR	-GD-----QD--------
	Mycobacterium xenopi	WP_085196673	--H-R------Q-----AR	--D------E--T-----
	Mycobacterium yongonense	WP_065503915	-----Q--E---Q------A	---D-----E--T-----
Other *Corynebacteriales*	*Corynebacterium glyciniphilum*	WP_038546165	-S---ITWNSSRD---RVEN	VT-A----ET-E-F----

Other *Corynebacteriales*	*Dietzia alimentaria*	WP_010542002	---T-HV-V-AD---INAKQ	ML-L----SE-PT-----
	Gordonia amarae	WP_005192366	--H-R-Q---SD-KF--AA	---H-----E--C-S---
	Hoyosella altamirensis	WP_064440667	--Q-H-QL-TED-KFMPEM	--EHF----E-EV-----
	Hoyosella subflava	WP_041451195	--Q-H-QL-TED-KFMPEM	--EHF----E-EV-----
	Millisia brevis	WP_084352147	---HVQL--ST-QFTPAD	---TF----T-ET-----
	Nocardia abscessus	WP_043696839	-SFLTR-HL-AEQ-KFA-AD	--TAF----E-HT-----
	Nocardia acidivorans	WP_067574215	-SFTAN-HY-GEN-KFA-AD	--EL-----E-DT-----
	Rhodococcus coprophilus	WP_072699435	--CH-QY--EQ-KFA--Q	--EVC----E--T-----
	Rhodococcus corynebacterioides	WP_068146486	VQL--SD-KF--RS	--ERF----E--T-----
	Segniliparus rotundus	WP_013139106	-QI-SAQ----EQR	-SEL-----E--T-----
	Skermania piniformis	WP_066473020	IQH---A-KFA-TE	--GLF----E--T-----
	Smaragdicoccus niigatensis	WP_018163275	-QL--SQ--FS-DS	--QLY----E--T-----
	Tsukamurella paurometabola	WP_013125757	--Y--DA---TPQN	-GEV----QD--T-I---
	Tsukamurella pseudospumae	WP_068570977	--Y--DA---TPAN	IGEV----QE--T-I---
	Williamsia herbipolensis	WP_045822955	--T-D-QL---M-KV--GR	-GDR-A--QT-ST-----
	Williamsia muralis	WP_062799195	IQF--S--KV-IER	--DLC---AS--T-----

Supplementary Figure 56

A partial sequence alignment of a conserved region of ferredoxin reductase showing a three amino acid insertion that is specific for members of the "*Terrae"* and *"Triviale"* clades and absent in other *Corynebacteriales*.

Clade	Species	Accession	37		80
"Terrae" and *"Triviale"* Clades (9/10)	*Mycobacterium algericum*	WP_083036515	LAEPGVGHGVADHSTVLSRPLD		RLRTTMTYIYVTTLGTEEERRA
	Mycobacterium heraklionense	WP_047321481	-----------N-----N----		----------CV----DD----
	Mycobacterium koreense	WP_085082048	M--------------A-R---N		---A--A-V-AV-M--P--Q--
	Mycobacterium longobardum	WP_013827258	-----------N-----N----		----------CV----D-----
	Mycobacterium senuense	ODR09923	----------------------		----------------------
	Mycobacterium sinense	WP_062879344	----------------------		----------------------
	Mycobacterium triviale	SKN28692	M--------------A-R---N		---A--A-V-AV-M--P--Q--
	Mycobacterium icosiumassiliensis	WP_085266253	--R----Y--MESKVESG-VDL	HPIK	-A---F--LV-S-Q-SD-QKA-
Other *Mycobacterium* (0/96)	*Mycobacterium abscessus*	WP_083139698	--R----Y--V-SKVESG-ADL	HPIK	-A---F-FLA-A----P-QKA-
	Mycobacterium abscessus subsp. bolletii	WP_083113875	--R----Y--V-SKVESG-ADL	HPVK	-A---F--LA-A-M-SP-QKA-
	Mycobacterium alsense	WP_036342866	--R----Y--LESRVESG-VDL	HPIK	-A---F--LA-A-A-SDAQKE-
	Mycobacterium angelicum	WP_065035666	-SR-------TESRVESG-IDR	HPIK	-A---F---A-ANA--DAQKA-
	Mycobacterium aromaticivorans	WP_048632578	--L----Y--MESRVESG-ADR	HPIK	-A---F--LA-A-R--D-QKK-
	Mycobacterium asiaticum	WP_036369125	-SR--------ESRVESG-IDR	HPIK	-A---F---A-ANA--DAQKA-
	Mycobacterium aurum	WP_062899462	-SR--------ESKVESGNLMK	HPWK	-A---FQ-LA-AVM-SDDD-A-
	Mycobacterium austroafricanum	BAN33368	--R----Y--LESRVESG-IDR	HPVK	-A---F--LA-S-N--P-QQA-
	Mycobacterium avium	ELP44663	--N----Y--MESRVESG-VDL	HPIK	-A---F--LA-A-S-S-AQKD-
	Mycobacterium avium subsp. hominissuis	WP_077740083	--N----Y--MESRVESG-VDL	HPIK	-A---F--LA-A-S-S-AQKD-
	Mycobacterium avium subsp. paratuberculosis	WP_083131785	--Q----Y--MESRVESG-VDL	HPFK	-A---F--LA-A-N-SDAQKA-
	Mycobacterium boenickei	WP_062829805	--N----Y--VESRVDSG-TDL	HPIK	-A---F--LA-A-R-SDAQKA-
	Mycobacterium branderi	WP_044095809	--R----Y--LESRVESG-VDL	HPIK	-A---F--LA-ASA-SD-QKA-
	Mycobacterium brisbanense	WP_062540877	--R----Y--MESRVESG-VDR	HPIK	-A---F--LA-A-R---QQKA-
	Mycobacterium canettii	WP_083005925	--M----Y--LESRVESG-LDR	HPIK	-A---F--VA-AVA--DDQKA-
	Mycobacterium celatum	WP_070915764	--R----Y--LESRVESG-VDL	HPIK	-A---F--LA-ASA-SD-QKA-
	Mycobacterium celeriflavum	WP_082168669	--R----Y--K-SRVESG-ADR	HPIK	-A---F--LA-ALA-S--QKAV
	Mycobacterium chelonae	WP_082162364	--R----Y--V-SKVESG-ADL	HPVK	-A---F--LA-A-M-SP-QKA-
	Mycobacterium chlorophenolicum	WP_064881068	--R----Y--LESRVHSG-VDL	HPIK	-A---F--LA-S-N--DAQKA-
	Mycobacterium chubuense	WP_065063279	--R----Y--LESRVDSG-VDL	HPIK	-A---F--LA-S-N--DAQKA-
	Mycobacterium colombiense	WP_085151780	--NA--AY--MESKVDSGNLHK	HPFK	-A---F---A-AIA-NAKD--V
	Mycobacterium conceptionense	WP_085231751	--N----Y--VESRVESG-TDL	HPIK	-A---F--LA-A-R-SDAQKA-
	Mycobacterium confluentis	WP_073855592	--Q-A--Y--MESRVESG-VDR	HPVK	-A---F--LA-A-N--D-QKAS
	Mycobacterium conspicuum	WP_083043188	--R----Y---ESRVESG-IDR	HPIK	-A---F--VA-ANA--DAQKE-
	Mycobacterium diernhoferi	WP_085242595	--R----Y--QESKVHSG-VDL	HPIK	-A---F--LA-AGR--D-QKA-
	Mycobacterium elephantis	WP_085096034	--R----Y--MESRVESG-VDR	HPIK	-A---F--LA-A-Q--D-QKA-
	Mycobacterium europaeum	WP_069412598	--R----Y--MESRVESG-VDL	HPIK	-A---F--LA-A-N-SDAQKD-
	Mycobacterium fallax	WP_085222547	--A-P-----LESTVESGQLMR	HPWK	-A---LS--S-AL--SDAD-GS
	Mycobacterium flavescens	WP_064848048	--R----Y--K-SRVESG-ADR	HPIK	-A---F--LA-ALA-S--QKAT
	Mycobacterium florentinum	WP_070938114	--R--------ESRVESG-IDR	HPIK	-A---F---A-ANG--DAQKA-
	Mycobacterium fortuitum	WP_025736514	--N-------VESRVESG-TDL	HPIK	-A---F--LA-A-R-SDAQKA-
	Mycobacterium franklinii	WP_049743338	--R----Y--V-SKVESG-ADV	HPIK	-A---F-FLA-SA--SP-QKA-
	Mycobacterium genavense	WP_055580209	-SR--------ESRVESG-IDR	HPIK	-A---F---A-ANA--A-QQA-
	Mycobacterium goodii	WP_018354477	--N----Y--VESRVDSG-TDL	HPVK	-A---F--LA-A-R---QQKA-
	Mycobacterium gordonae	WP_071700113	-SL--------ESRVESG-IDR	HPIK	-A---F---A-ANA--DTQKA-
	Mycobacterium hassiacum	WP_083076047	--L----Y--MESRVESG-IDR	HPIK	-A---F--LA-A-M----QKA-
	Mycobacterium heckeshornense	WP_047317423	--R----Y--MESRVESG-VDL	HPIK	-A---F--LA-A-A---AQKA-
	Mycobacterium heidelbergense	WP_069407217	--R----Y--LESRVESG-VDL	HPIK	-A---F--LA-A-A-SDAQKE-
	Mycobacterium holsaticum	WP_066900648	--R----Y--MESRVESG-VDR	HPIK	-A---F--LA-A-M-SDQQKA-
	Mycobacterium houstonense	WP_067967925	--N-A--Y--VESRVDSG-TDL	HPIK	-A---F--LA-A-R--DAQKK-
	Mycobacterium immunogenum	WP_064627269	--R----Y--V-SKVESG-ADL	HPIK	-A---F-FLS-A----P-QKA-
	Mycobacterium interjectum	WP_085200809	--R----Y--LESRVESG-VDL	HPIK	-A---F--LA-A-A-SDAQKE-
	Mycobacterium intracellulare	WP_064936470	--Q----Y--MESRVESG-VDL	HPFK	-A---F--LA-A-N-SDAQKA-
	Mycobacterium iranicum	WP_064281569	--R----Y--LESRVESG-IDR	HPVK	-A---F--LA-S-N--A-QKA-
	Mycobacterium kansasii	ORB87601	--R----C--VESRVESG-LDL	HPIK	-T---F---A-AMA-SD-QKA-
	Mycobacterium komanii	CRL71758	--R----Y--K-SRVESG-ADR	HPIK	-A---F--LA-ALA--D-QKAT
	Mycobacterium kubicae	WP_085075629	--R----Y--MESRVESG-VDL	HPIK	-A---F--LA-A-A--DAQKA-
	Mycobacterium kyorinense	WP_045374214	--R----Y--LESRVESG-VDL	HPVK	-A---F--LA-ASA-SD-QKA-
	Mycobacterium lacus	WP_085159074	--R-------MESRVESG-IDR	HPIK	-A---F---A-ANA--DAQKE-
	Mycobacterium lentiflavum	CQD04404	-SI--------ESRVESG-IDR	HPIK	-A---F---A-ANA--D-QKA-
	Mycobacterium liflandii	WP_015354275	--R-------MESRVESG-IDR	HPIK	-A---F--VA-ASA--D-QKA-
	Mycobacterium litorale	WP_078017570	--R----Y--MESRVESG-ADR	HPIK	-A---F--LA-A-R-SD-QKK-
	Mycobacterium mageritense	WP_036435695	--R----Y--MESRVESG-VDR	HPIK	-A---F--LA-A-R-SDQQKA-
	Mycobacterium malmesburyense	CRL73558	--R----Y--K-SRVESG-ADR	HPIK	-A---F--LA-ALA--D-QKAT
	Mycobacterium malmoense	WP_065443811	--R----Y--LESRVESG-VDL	HPIK	-A---F--LA-A-M-SDAQKD-
	Mycobacterium mantenii	WP_083096718	--Q----Y--MESRVESG-VDL	HPIK	-A---F--LA-A-N-SDAQKD-

Other *Mycobacterium* (0/96)

Mycobacterium marinum	WP_012392504	--R-------MESRVESG-IDR HPIK -A---F--VA-ASA--D-QKA-
Mycobacterium marseillense	WP_083019298	--Q----Y--MESRVESG-VDR HPFK -A---F--LA-A-N-SDAQKD-
Mycobacterium moriokaense	WP_083152803	-SR----Y--K-SRVESG-ADR HPIK -A---F--LA-ALS--D-QKK-
Mycobacterium mucogenicum	WP_061003145	--R----Y--VESRVESG-TDL HPIK -A---F--LA-AI--SD-QKA-
Mycobacterium nebraskense	WP_085164193	--R----Y--MESRVESG-VDL HPIK -A---F--LA-A-N-SDAQKE-
Mycobacterium neworleansense	CRZ16696	--D----Y--VESRVESG-TDL HPIK -A---F--LA-A-R-SDAQKA-
Mycobacterium noviomagense	WP_083088803	--R----Y--LESRVESG-VDL HPIK -A---F--LA-A-A---AQKL-
Mycobacterium novocastrense	WP_067392589	-SR----Y--K-SRVESG-ADR HPIK -A---F--LA-ALA--D-QKAT
Mycobacterium obuense	WP_082133533	--R----Y--LESRVDSG-VDL HPIK -A---F--LA-S-S--DAQKD-
Mycobacterium palustre	WP_085078331	--R----Y--LESRVESG-VDL HPIK -A---F--LA-A-A-S-AQKE-
Mycobacterium paraense	WP_085102322	-SR-------VESRVESG-IDR HPIK -A---F---A-ANA---AQKA-
Mycobacterium paraseoulense	WP_083174533	--R----Y--MESRVESG-IDL HPIK -A---F--LA-A-N-SDAQKA-
Mycobacterium parmense	WP_085267374	--R----Y--MQSRVESG-VDL HPIK -A---F--LA-A-R-S-TQKA-
Mycobacterium peregrinum	WP_064886584	--N----Y--VESRVESG-TDL HPIK -A---F--LA-A-R-SDAQKA-
Mycobacterium phlei	WP_003887305	--R----Y--K-SRVESG-ADK HPIK -A---F--LA-ALM--D-QKAT
Mycobacterium porcinum	WP_075923899	--N----Y--VESRVDSG-TDL HPIK -A---F--LA-A-R-SDAQKA-
Mycobacterium pseudoshottsii	GAQ32567	--R-------MESRVESG-IDR HPIK -A---F--VA-ASA--D-QKA-
Mycobacterium rhodesiae	WP_083117346	--L----Y--MESRVESG-ADR HPIK -A---F--LA-A-R--D-QKK-
Mycobacterium rufum	KGI66431	--R----Y--LESRVESG-VDL HPLK -A---F--LA-S-H--DAQKA-
Mycobacterium rutilum	WP_083409880	-SR----Y---ESKVDSGNL-K HPWK -A---FQ-LA-AV---A-D-A-
Mycobacterium salmoniphilum	WP_078326013	--R----Y--V-SKVESG-ADL HPVK -A---F-FLA-SS--SP-QKA-
Mycobacterium saopaulense	WP_070911911	--R----Y--V-SKVESG-ADL HPIK -A---F-FLS-S---NPDQKA-
Mycobacterium scrofulaceum	WP_067280143	--R----Y--MESRVESG-VDL HPIK -A---F--LA-A-N--DAQKN-
Mycobacterium septicum	WP_044519126	--N----Y--VESRVDSG-TDL HPIK -A---F--LA-A-R-SDAQKA-
Mycobacterium setense	WP_064871309	--N----Y--VESRVDSG-TDL HPIK -A---F--LA-A-R--DAQKA-
Mycobacterium sherrisii	WP_069399964	-SL--------ESRVESG-IDR HPIK -A---F---A-ANA--DAQKA-
Mycobacterium shimoidei	WP_069397106	--R----Y--VESRVESG-VDK HPIK -A---F--LA-AMA--DAQKQ-
Mycobacterium simiae	WP_061558049	-SI--------ESRVESG-IDR HPIK -A---F---A-ANA--D-QKA-
Mycobacterium smegmatis	WP_080627956	--N----Y--VESRVDSG-TDL HPVK -A---F--LA-A-R---AQKA-
Mycobacterium szulgai	WP_068033453	--R----Y--MESRVESG-VDL HPIK -A---F--LA-A-A--DAQKA-
Mycobacterium thermoresistibile	EHI13802	--L----Y--LESPVDSGNVYK HPFK -A---G--LAAA-M---AD--L
Mycobacterium triplex	WP_036466214	-SR--------ESRVESG-IDR HPIK -A---F---A-ANA--A-QKA-
Mycobacterium tuberculosis	CNE13996	--RLPI-Y---ESTVESG-VDK HPVK -G---L---A-AM----D--K-
Mycobacterium tusciae	WP_083128110	--R----Y--K-SRVESG-ADR HPIK -A---F--LA-ALS----QKKL
Mycobacterium ulcerans	WP_011739398	--R-------MESRVESG-IDR HPIK -A---F--VA-ASA--D-QKA-
Mycobacterium vaccae	WP_003933741	--R----Y--VESRVESG-IDR HPVK -A---F--LA-S-S--A-QQA-
Mycobacterium vanbaalenii	WP_011777731	--R----Y--LESRVESG-IDR HPVK -A---F--LA-S-N--P-QQA-
Mycobacterium vulneris	WP_065513220	--N----Y--VESRVDSG-TDL HPIK -A---F--LA-A-R-SDAQKA-
Mycobacterium wolinskyi	WP_085146392	--R----Y--LESRVDSG-VDL HPIK -A---F--LA-A-R-SDQQKA-
Mycobacterium xenopi	WP_003922930	--R----Y--LESRVESG-VDL HPIK -A---F--LA-A-A---AQKA-

Supplementary Figure 57

A partial sequence alignment of a conserved region of DUF2236 domain-containing protein showing a four amino acid deletion that is specific for most members of the "*Terrae*" and "*Triviale*" clades and absent in other *Mycobacterium*.

Group	Species	Accession	100		141
**"*Terrae*" and "*Triviale*" Clades (15/15)**	*Mycobacterium algericum*	WP_083040170	GELRIGAHPVRAFAAAAAET	G	HDSVAAGLRGVVARARLGADV
	Mycobacterium arupense	WP_083070934	---------L----V----S	T	-RG-----G--A---------
	Mycobacterium avium	WP_080575773	--------------V-----	-	-GP------A-----------
	Mycobacterium engbaekii	WP_085128720	----V---------VG---A	-	-GG-----G--A---------
	Mycobacterium heraklionense	WP_064889316	----V---------L----S	N	-PA-V---G--A---------
	Mycobacterium hiberniae	WP_085134080	----V---------VG---A	-	-RV-----G--A---------
	Mycobacterium icosiumassiliensis	WP_067976548	----V-----Q---L----S	N	-PA-----G--A---------
	Mycobacterium koreense	WP_085303746	-------D--AGLLV--DDA	E	PGA-RQ---S-A-G-------
	Mycobacterium kumamotonense	WP_065289404	--------------V-----	-	-GP------A-----------
	Mycobacterium longobardum	WP_085265472	----V---------V-----	-	-PA--------A---------
	Mycobacterium minnesotense	WP_083026090	---V----L---TI----S	T	-PGI----G--A---------
	Mycobacterium senuense	WP_085085630	--------------------	-	-GA----------T------
	Mycobacterium sinense	WP_064856028	------------DV-----	-	-RP---A--------------
	Mycobacterium terrae	WP_085260316	--------------V-----	-	-G-------------------
	Mycobacterium triviale	WP_069393152	-------D--AGLLV--DDA	E	PGA-RQ---S-A-G-------
**Other *Mycobacterium* (0/>100)**	*Mycobacterium abscessus*	WP_016341713	----V--------EI----L		NGAAGETFAAIA------MN-
	Mycobacterium abscessus subsp. bolletii	SKL51467	----V-----H--EIT---L		GGT-GEVFAAMA------VKL
	Mycobacterium alsense	WP_083138398	--M-V--------EV----S		AGT-RSS--A-A---------
	Mycobacterium aromaticivorans	WP_036341819	----V---------V----S		GGAIGTA--S-A---G-----
	Mycobacterium arosiense	WP_083063983	----A-S------CV--DDS		AGA--VS--S-A-----
	Mycobacterium asiaticum	WP_065035921	----V-----N--DV--D--		DGA-SGA--T-A----M----
	Mycobacterium aurum	WP_048633955	----V-----A-IST--H-S		D-R--RS-GA-A---L-----
	Mycobacterium avium	WP_062886580	----A-S------DV--G--		AGP--VA--S-A------
	Mycobacterium avium subsp. avium	EUA25058	----A-S------GV--D--		AGA---S--S-A------
	Mycobacterium avium subsp. hominissuis	KDO98431	----A-S------DV--G--		AGP--VA--S-A-------
	Mycobacterium bacteremicum	WP_083058673	----V-----A-L------V		GGR-GPC-DE-S--G-----I
	Mycobacterium bohemicum	WP_085182344	----V---------V--D--		SGT--VS--A-A---------
	Mycobacterium brisbanense	WP_062828418	----V-----A--TV--Q-V		AGP--D-M-A-A---------
	Mycobacterium canariasense	WP_062656013	----V-----T--DV----V		DGP---A-H--A--G------
	Mycobacterium celatum	WP_085168529	----V--------EV-----		VAP--DCC-A-A---------
	Mycobacterium celeriflavum	WP_083000278	----------A--D-----V		DGV--LS--T-A----M----
	Mycobacterium chelonae	WP_070918853	----V-----H--EIT---L		GGT-GEVFAAMA------VKL
	Mycobacterium chimaera	WP_074020893	----A--------GV--D--		AGA---S--S-A------
	Mycobacterium chlorophenolicum	KMO72181	---V-----A-IGV--R-S		DGR--GSFGA-A---M-----
	Mycobacterium chubuense	WP_081495172	---V-----G-IEV----A		DGQ--ES-NA-A---L-----
	Mycobacterium colombiense	WP_065027032	----A-S------GV--DD-		VGA---S--S-A------
	Mycobacterium conceptionense	WP_064898565	----T-V---T---T--G-V		SGP-RD-MSA-A---------
	Mycobacterium confluentis	ORV28587	---V-----Q--MV--Q-S		-GE---AF---A---L-----
	Mycobacterium conspicuum	WP_085232723	----V-S---D--CV--D--		GGT---S--A-A---------
	Mycobacterium cosmeticum	WP_036397707	----V-----T--DV----V		DGP---A-H--A--G------
	Mycobacterium diernhoferi	WP_073856902	----A-----A--G--G--L		GGD-GER-CL-A--G------
	Mycobacterium doricum	WP_085188297	----V-----A--E---G--		GGQ--DN--A-A---------
	Mycobacterium elephantis	WP_083043443	----V----AT--ET--R-V		DGG---S--T-A----M----
	Mycobacterium europaeum	WP_085241408	----V-S------GV-----		AGA--ES--A-A---------
	Mycobacterium farcinogenes	WP_036391354	----T-V---S---T--G-V		SGP-RD-MSA-A---------
	Mycobacterium flavescens	WP_069415317	----------V--TS--G-V		DGA---S--T-S----M----
	Mycobacterium florentinum	WP_085223175	----V----L---GV--D--		VGA--IS----A---------
	Mycobacterium fortuitum	WP_064898287	----T-V---A--GT--G-V		SGP-RQ-MGA-A---------
	Mycobacterium fragae	WP_085200161	----V----A----V--D--		VGA--NSC-A-A---------
	Mycobacterium franklinii	WP_070937327	----V--------ET----V		GGP-GEVFAAIA------VNL
	Mycobacterium gastri	WP_036413722	----V-L------GT--D--		EGA---SM-A-A---------
	Mycobacterium gilvum	WP_011892283	----V-----A-ITV-GH-A		RGRI-ES-NA-A---L-----
	Mycobacterium goodii	WP_049743436	----------D--G---G-S		EGT--E-M-A-A---------
	Mycobacterium gordonae	WP_065045431	----V-----H--DV--D--		AGA----M-A-A---------
	Mycobacterium hassiacum	WP_018354203	---V-----A-L----DD-		GAT-G-A--TMA---------
	Mycobacterium heckeshornense	WP_048891489	----A-----Q--SV-----		GGT--ERFHA-A---------
	Mycobacterium heidelbergense	WP_083073764	----V-S------RV--D--		VGA--VS--T-A---------
	Mycobacterium holsaticum	WP_069405255	----V----AA--GT--K-V		DGD---S--T-A---GM----
	Mycobacterium immunogenum	OAT67863	----V--------EV----L		SGK-GEAFA-TA----V-VNL
	Mycobacterium indicus pranii	WP_014941193	----A-S------GV--D--		PGA---S--S-A------
	Mycobacterium interjectum	WP_085203347	----V-S------HV--G-S		VGA---S--A-G---------
	Mycobacterium intermedium	WP_069418026	----V-----N--DV--K--		IGP-GDC--A-A---------
	Mycobacterium intracellulare	WP_064938663	----A--------G---D--		AGA---S--S-A------
	Mycobacterium iranicum	WP_024446936	----V-----A-ID---Q--		DGRM--S-GA-A---V-----

**Other *Mycobacterium* (0/>100)**	*Mycobacterium kansasii*	WP_063467209	----V-I------GT--D--	DGV---SM-A-A---------
	Mycobacterium komanii	CRL74098	----------T--DT--S--	EGNA--S--T-A----M----
	Mycobacterium kubicae	WP_085074667	----V-----H--SV-----	GGP-GTA----A---------
	Mycobacterium kyorinense	WP_065014398	---QV---------V--D-S	GGA--T-C-S-A---------
	Mycobacterium lacus	WP_085160980	----V--------GV--D--	VGP----M-A-A---L-----
	Mycobacterium lentiflavum	CQD21889	----V-----Q--G---D--	VGA-SVS----A---------
	Mycobacterium liflandii	WP_015357487	----V--------GV--D--	DGP---AM---A-H-------
	Mycobacterium llatzerense	WP_082067876	----A-----A--D---H-V	DGP--TA-GE-A---------
	Mycobacterium mageritense	WP_036431911	----V-----S--ST----V	SGP--D-M-T-A--G------
	Mycobacterium malmesburyense	CRL79205	----------A--DT----V	DGAA-VS--T-A----M----
	Mycobacterium malmoense	WP_065443439	----V--------GV--G--	AGA--ES--A-A---------
	Mycobacterium mantenii	WP_083098958	----A-S------CV--DD-	GGA--VS--S-A-----
	Mycobacterium marinum	WP_012396662	----V--------GV--D--	DGP---AM---A-H-------
	Mycobacterium moriokaense	WP_083155257	----V-----A-LG---VDV	GGV--TA--TIA---------
	Mycobacterium mucogenicum	WP_064857695	----A-----A--D---H-V	GGP-S-A-GE-A---------
	Mycobacterium nebraskense	WP_047322471	----V-SD-----GV--G--	TGAA-ES--V-A---------
	Mycobacterium neoaurum	CDQ47248	----V-----A-LT---G-V	GGQ-GDR--T-S-HG-----I
	Mycobacterium neworleansense	CRZ17823	----T-V---T--GT--G-V	SGP-H--MSA-A---------
	Mycobacterium noviomagense	WP_083089271	----S---------V--N--	DGT--DRF-T-A---------
	Mycobacterium novocastrense	WP_067394162	----------T--DT--D-A	DGA-STA--T-S----M-G--
	Mycobacterium paraense	ORW54128	----V--------HV--G-S	VGA---S--A-A---------
	Mycobacterium paraffinicum	WP_073871473	----V-S------EV--G--	VGA--ESF-A-A---------
	Mycobacterium parafortuitum	WP_083142064	----V-----A-IEV--Q-A	RERI-DS-T--A---L-----
	Mycobacterium paraintracellulare	AFC51932	----A-S------GV--D--	AGA---S--S-A------
	Mycobacterium paraseoulense	WP_083173449	----V-S------GV--G--	GGA--ES--A-A---------
	Mycobacterium parmense	WP_085269149	----V-S------RV--G--	AGA--ES-GA-A---G-----
	Mycobacterium peregrinum	WP_064884017	----T-V---S--GT--G-V	SGA-RQ-MGA-A---------
	Mycobacterium phlei	WP_003888592	----------A--DT--D-V	GGA---A--T-A----M----
	Mycobacterium porcinum	WP_069424680	----T-V---T---T-GG-V	SGP-QR-MSA-A---------
	Mycobacterium rhodesiae	WP_083120819	----V---------V--G-S	GGA-G-A--S-A---G-----
	Mycobacterium rufum	KGI70100	---V-----A-IRV-GR-G	-GR--HSFGA-A---L-----
	Mycobacterium rutilum	WP_083406939	----------V--DT--G-V	GGV---SM-T-S----M----
	Mycobacterium salmoniphilum	WP_078324439	----V--------EVT---L	GGTTGEVFAAIA------VTL
	Mycobacterium saskatchewanense	WP_085255245	----V--------SV--D--	DGP--VS--T-A---------
	Mycobacterium scrofulaceum	WP_067270681	----V--------GV--G--	AGA--ESF-A-A---------
	Mycobacterium septicum	WP_044520977	----T-V---N--GT--C-V	SGP-HH--SA-A---------
	Mycobacterium setense	WP_039382432	----T-V---N---T----V	SGP-HE-MSA-A---------
	Mycobacterium sherrisii	WP_069400771	----V-N-------V--D--	AGP---A----A---------
	Mycobacterium simiae	WP_061557418	----V-S------TV--D--	AGP--VA----A---------
	Mycobacterium smegmatis	WP_080628362	----V-----D--G---G-S	GGP--E-M-A-A---------
	Mycobacterium szulgai	WP_068023060	----V-----H--SV-----	GGP-GTA----A---------
	Mycobacterium thermoresistibile	WP_003925190	----V-----S-VEV--R-V	SGTTGLA--E-A------GE-
	Mycobacterium triplex	WP_036471860	----V-----Q--DV-----	VGA--RS--A-A---------
	Mycobacterium tuberculosis TKK	KBZ58263	----A-S------G---DD-	EGA-G-S--S-A-----
	Mycobacterium tusciae	WP_083126247	----V-----T--DI--DD-	EGE--SA--TIA---------
	Mycobacterium ulcerans Agy99	ABL06260	----V--------GV--D--	DGP---AM---A-H-------
	Mycobacterium vaccae	WP_082762424	----------A-VR---H-S	NQA-SGS-T--A---L-----
	Mycobacterium vulneris	WP_065462815	----T-V---T---T-GG-V	SGP-QR-MSA-A---------
	Mycobacterium wolinskyi	WP_067850312	----------S--GT--G-V	SGP-GE-M-A-A---------
	Mycobacterium xenopi	WP_085193069	----T-----Q--SV-----	GGI--DRFHT-A---------
	Mycobacterium xenopi RIVM700367	EID12247	----T-----Q--SV-----	GGI--DRFHT-A---------
	Mycobacterium yongonense	WP_065500425	----A-S------GV--D--	AGA---S--S-A------

Supplementary Figure 58

A partial sequence alignment of a conserved region of a hypothetical protein showing a one amino acid insertion that is specific for members of the "*Terrae*" and "*Triviale*" clades and absent in other *Mycobacterium*.

			286		324
**"*Terrae*" and "*Triviale*" Clades (13/13)**	*Mycobacterium arupense*	WP_083070918	LGTAPVTTVVQHAEWP	DPP	VPVETLPSPFDNRLAQPYGG
	Mycobacterium engbaekii	WP_085129423	----------------	---	--------------------
	Mycobacterium heraklionense	OBG30933	----------------	E--	--------------------
	Mycobacterium hiberniae	WP_085136269	----------------	---	--------------------
	Mycobacterium icosiumassiliensis	WP_067976628	------------T---	---	------------G-------
	Mycobacterium kumamotonense	WP_065289563	------------T---	---	--------------------
	Mycobacterium longobardum	WP_085265510	----------------	-W-	--A-----------------
	Mycobacterium minnesotense	WP_083025474	----------------	---	M------P------------
	Mycobacterium nonchromogenicum	WP_085138297	----------------	---	---------L----------
	Mycobacterium senuense	WP_085085547	----------------	---	--------------------
	Mycobacterium sinense	WP_064855734	------------T---	-A-	--------------------
	Mycobacterium terrae	WP_085259940	------------T---	---	--------------------
	Mycobacterium triviale	WP_085109310	--S----------D--	AW-	E---S--D-G----------
**Other *Mycobacterium* (0/>100)**	*Mycobacterium abscessus*	AMU23507	--AV------NAGA--		AVADE--ESW--T-------
	Mycobacterium abscessus subsp. bolletii	SIJ68305	--AV------NAGA--		AVADE--ESW--T-------
	Mycobacterium acapulcensis	WP_066810270	--A-------VAS---		D-ADH-GP-EN---------
	Mycobacterium africanum	AMC66278	--A-------R-D---		E-A-S--P-Y----------
	Mycobacterium alsense	WP_083140804	------------D---		D-A-S--P-Y----------
	Mycobacterium angelicum	WP_083115760	--S---------D---		E-A-S--P-Y----------
	Mycobacterium aromaticivorans	WP_036346450	-----------AGD--		D---S--P-G----------
	Mycobacterium arosiense	WP_083063916	--D---------D---		D-A-S--P-Y----------
	Mycobacterium aurum	WP_083443184	----------VA-P--		D---V--P-Q----------
	Mycobacterium austroafricanum	WP_051558554	----------VA-P--		DR--D--P-H----------
	Mycobacterium avium	WP_062899483	--D---------D---		E-A-S--P-Y----------
	Mycobacterium avium subsp. avium 2285	EUA24981	--D---------D---		E-A-S--P-Y----------
	Mycobacterium avium subsp. paratuberculosis	AAS02626	--D---------D---		E-A-S--P-Y----------
	Mycobacterium bacteremicum	ORA01520	--S-------VA-P--		ERP-Q--A-D----------
	Mycobacterium boenickei	WP_077738592	--S-------FAS---		D--DA--P-EV-Q-------
	Mycobacterium bohemicum	WP_085183283	--D---------D---		D-ADS--P------------
	Mycobacterium branderi	WP_083134484	------------D---		D-A-S--P-Y----------
	Mycobacterium canariasense	WP_084395547	-A--------VA-P--		EAP-E--D-D--S-------
	Mycobacterium canettii	WP_080602981	--A-------R-D---		E-A-S--P-Y----------
	Mycobacterium celatum	WP_084707106	------------D---		D-A-S--P-Y----------
	Mycobacterium celeriflavum	WP_083000486	----------VAT---		D--DH-GP-EN---------
	Mycobacterium chelonae	WP_070917250	--AV-A----NAGA--		EVADE--ESW--T-------
	Mycobacterium chimaera	WP_087139637	--D---------D---		E-A-S--P-Y----------
	Mycobacterium chlorophenolicum	WP_082169033	----------VA-P--		DRA-D--P-Q----------
	Mycobacterium chubuense	WP_081495174	--A-------VASP--		ARP-D--P-Q----------
	Mycobacterium colombiense	WP_064880012	--D---------D---		D-A-S--P-Y----------
	Mycobacterium conceptionense	WP_064898405	--S-------FASA--		D--DS--P-EV-Q-------
	Mycobacterium confluentis	ORV24974	--S--------AS---		DRA-D----E----------
	Mycobacterium conspicuum	WP_085232732	--D---------D---		D-A-S--P-Y----------
	Mycobacterium diernhoferi	OJZ62750	--S-------VG-P--		DRPTD--A-Y----------
	Mycobacterium doricum	WP_085191950	--A-------VA-D--		EQP-F--P-EV---------
	Mycobacterium elephantis	WP_083043806	----------VAT---		T---H-GP-EN---------
	Mycobacterium europaeum	WP_085241298	--D---------D---		D-A-S--P-Y----------
	Mycobacterium fallax	ORV08119	--S-------VATD--		D--DA--P-W--G-------
	Mycobacterium farcinogenes	CDP87900	--S-------FASA--		D--DS--P-EV-Q-------
	Mycobacterium flavescens	WP_069415393	--S-------VA----		D---H-GP-EN---------
	Mycobacterium florentinum	WP_085224968	--A---------D---		E-A-S--P-Y----------
	Mycobacterium fortuitum	OBG46509	----------FASA--		D-ADS--P-EV-Q-------
	Mycobacterium franklinii	WP_070939083	--AV------NAGA--		AVADE--ESW--T-------
	Mycobacterium genavense	WP_051465188	--E---------D---		E-A-S--P-Y----------
	Mycobacterium gilvum	WP_011892204	----------VA-P--		D--DV--P-H----------
	Mycobacterium goodii	WP_049743503	--S-------YASD--		D-A----P-EV---------
	Mycobacterium haemophilum	WP_047316207	------------D---		D-A-S--P-Y----------
	Mycobacterium hassiacum	WP_018354222	----------IAS---		ERP-D-GP-EN---------
	Mycobacterium heckeshornense	WP_048891445	------------D---		E-A-S--P-Y----------
	Mycobacterium heidelbergense	WP_083074959	------------D---		E-A-S--P-Y----------
	Mycobacterium holsaticum	WP_069404955	--A-------VATD--		T---N-GP-EN---------
	Mycobacterium houstonense	WP_066902264	--E-------FASA--		D---S--P-EV-Q-------
	Mycobacterium immunogenum	WP_064631711	--AV------NAGA--		AVADE--ESW--T-------
	Mycobacterium insubricum	WP_083034007	----------VA-D--		D-IDA-GA-E--A-------
	Mycobacterium interjectum	WP_066916754	------------D---		D-A-S--P-Y----------
	Mycobacterium intracellulare	OBH71515	--D---------D---		E-A-S--A-Y----------
	Mycobacterium iranicum	WP_064283241	-----M----VAGP--		ERA-D--P------------
	Mycobacterium kansasii	ORB84588	------------D---		D-ADS--P-Y-----E----
	Mycobacterium komanii	CRL73932	--A-------VA----		D-ADH-GP-ES---------
	Mycobacterium kyorinense	OBI53875	------------D---		D-A-S--P-Y----------
	Mycobacterium lacus	WP_085158928	--D---------D---		E-P-S--P-Y----------

Other *Mycobacterium* (0/>100)

Mycobacterium lentiflavum	CQD22106	--E---------D---	E-A-S--P-T----------
Mycobacterium leprae	WP_010908829	---S--------D---	D-A-S--P-Y----------
Mycobacterium lepromatosis	WP_045843769	---S--------D---	D-A-S--P-Y----------
Mycobacterium liflandii	WP_015357541	--D---------D---	E-A-S--P------------
Mycobacterium litorale	AQT82049	--A--------AGD--	A-ADD--P-G--Q-------
Mycobacterium llatzerense	WP_082067999	--A-------VAGG--	EQADE--V-Q--Q-------
Mycobacterium mageritense	WP_085980337	--S-------FASA--	D--DS--P-EV-Q-------
Mycobacterium malmesburyense	CRL78185	--A-------VAT---	D-ADH-GP-EN---------
Mycobacterium malmoense	WP_065445090	--D-------------	E-A-S--P-Y----------
Mycobacterium mantenii	WP_083095557	--D---------D---	E-A-S--P-Y----------
Mycobacterium marinum	WP_012396732	--D---------D---	E-A-S--P------------
Mycobacterium marseillense	ORA94835	--D---------D---	E-A-S--P-Y----------
Mycobacterium monacense	WP_083045317	--D-------VAED--	DQA-F--P-EV---------
Mycobacterium moriokaense	WP_083154998	----------VAT---	D-A-D-GP-EN---------
Mycobacterium mucogenicum	WP_064857739	----------VAGG--	EQADE--A-Q--Q-------
Mycobacterium nebraskense	WP_046185703	--D---------D---	E-A-S--P-Y----------
Mycobacterium neoaurum	CDQ47313	--W-------RA-P--	ERP-E--A-D----------
Mycobacterium neworleansense	CRZ17714	----------FASA--	D--DS--P-EV-Q-------
Mycobacterium noviomagense	WP_083089807	--A---------D---	D-A-S--P-Y----------
Mycobacterium novocastrense	WP_084377320	----------VAS---	D-ADH-GP-ES---------
Mycobacterium obuense	WP_046364045	----------VA-P--	EQADD--A-Q----------
Mycobacterium palustre	WP_085076716	------------D---	D-A-S--P-Y----------
Mycobacterium paraense	ORW31728	------------D---	D-A-S--P-Y----------
Mycobacterium paraffinicum	WP_073871681	--D---------D---	D-A-S--P-D----------
Mycobacterium parafortuitum	ORB30933	----------VA-P--	D-ADV--PAH----------
Mycobacterium paraintracellulare	AFC51863	--D---------D---	E-A-S--P-Y----------
Mycobacterium parascrofulaceum	WP_040620907	--D-------------	E-A-S--P-Y----------
Mycobacterium paraseoulense	WP_083176620	--D---------D---	E-A-S--P-Y----------
Mycobacterium parmense	WP_085271235	--N-------R-DD--	D-A-S--P-Y----------
Mycobacterium peregrinum	OBB97193	--S-------FASA--	D--DS--P-EV-E-------
Mycobacterium phlei	WP_061481419	--A-------VASD--	D--DH-GP-DN---------
Mycobacterium porcinum	WP_069425641	----------FAS---	D--DA--P-EV-Q-------
Mycobacterium rhodesiae	WP_083120956	-----------AGD--	D-EDS--PSD----------
Mycobacterium riyadhense	WP_085248919	--D-------R-D---	D-A-S--P-Y----------
Mycobacterium rufum	KGI71189	----------VA-P--	DRA-D--P-Q----------
Mycobacterium rutilum	SEH59762	----------VA----	D---H-GP-EN---------
Mycobacterium salmoniphilum	WP_078324556	--AV-A----NAGA--	AMADE--ESW--T-------
Mycobacterium saopaulense	OHT83385	--AV-A----NAGA--	AVADE--ESW--T-------
Mycobacterium saskatchewanense	WP_085258262	--D---------D---	D-A-S--P-S----------
Mycobacterium scrofulaceum	WP_067270928	--D---------D---	D-A-S--P-S----------
Mycobacterium septicum	WP_044521256	----------FASA--	D--DS--P-EV-Q-------
Mycobacterium setense	WP_064875264	----------FASA--	D--D---P-EV-Q-------
Mycobacterium sherrisii	WP_085167086	--D---------D---	E-A-S--P-E----------
Mycobacterium shigaense	BAX94826	------------D---	E-A-S--P-T----------
Mycobacterium shimoidei	WP_069397209	------------DD--	D-A-S--P-H----------
Mycobacterium shinjukuense	WP_083052613	--A-------R-D---	E-A-S--P-Y----------
Mycobacterium simiae	AMP26121	--E---------D---	E-A-S--P-E----------
Mycobacterium smegmatis	WP_080628228	----------YASD--	D-A----P-EV---------
Mycobacterium szulgai	OBF15517	------------DD--	D-A-S--A-Q----------
Mycobacterium thermoresistibile	WP_050811958	----------VAS---	D-A-H--P-D----------
Mycobacterium triplex	CDO85753	--E---------D---	E-A-S----Y----------
Mycobacterium tuberculosis	OAK49448	--A-------R-D---	E-A-S--P-Y----------
Mycobacterium tusciae	WP_083126527	----------VAT---	D---H-GP-EN---------
Mycobacterium ulcerans	WP_011741913	--N---------D---	E-A-S--P------------
Mycobacterium vaccae	WP_086006078	----------VA-P--	ER--D--P-Q----------
Mycobacterium vanbaalenii	WP_041307225	----------VA-P--	DR--D--P-H----------
Mycobacterium vulneris	WP_065462882	----------FAS---	D--DA--P-EV-Q-------
Mycobacterium wolinskyi	WP_067851771	----------VAS---	E-A-A--P-EV---------
Mycobacterium xenopi	WP_081485369	----------R-D---	E-A-S--P-Y----------
Mycobacterium yongonense	WP_065498618	--D---------D---	E-A-S--P-Y----------

Supplementary Figure 59

A partial sequence alignment of a conserved region of DUF4185 domain-containing protein showing a three amino acid insertion that is specific for members of the "*Terrae*" and "*Triviale*" clades and absent in other *Mycobacterium*.

Group	Species	Accession	497		532
***"Terrae"* Clade (9/9)**	*Mycobacterium algericum*	WP_083036306	PGVVDAMFGHQLDELHRLA		DPDAWTTPDSPEVPESQ
	Mycobacterium arupense	WP_046188788	---I----A-HIT-VT---		GGQG-VDHAAAAL-DD-
	Mycobacterium heraklionense	WP_047319994	---I----A-H-AD-T---		GG-G-
	Mycobacterium hiberniae	WP_085134798	---I----A-HVAD-T---		GG-G-GQ-APAAL-AE-
	Mycobacterium kumamotonense	WP_065288331	---I----A-HIADVT---		GGEG-DE-APAAL-AE-
	Mycobacterium longobardum	WP_085263433	---A----A----------		--E--K-----D-----
	Mycobacterium minnesotense	WP_083022343	---I----A-HIAD-T---		AGQG-AE-APVAL-AD-
	Mycobacterium senuense	WP_085083611	-------------------		--E--K-----------
	Mycobacterium sinense	WP_013828465	-------------------		-----------------
**Other *Mycobacterium* (0/69)**	*Mycobacterium alsense*	WP_083136823	--------A-HI---L---	S	AD---DA-GP-AL-DA-
	Mycobacterium aromaticivorans	KDF00236	--AI----A-HI--VQ---	R	-D---DSRRG-LL-P--
	Mycobacterium arosiense	WP_083062962	---I-----YHI---L---	S	AD---DA-GP-AL--T-
	Mycobacterium aurum	WP_048633073	---A----A-HIA--E---	D	-EKS-EAG-LTP--AG-
	Mycobacterium avium	WP_062890591	---I----AYHI---L---	S	AD---DA-GPAAL--A-
	Mycobacterium avium subsp. avium	EUA36980	---I----A-HI---L---	S	AD---DA-GPAAL--A-
	Mycobacterium avium subsp. paratuberculosis	OUZ04476	---I----AYHI---L---	S	AD---DA-GPAAL--A-
	Mycobacterium bohemicum	WP_085182938	---I----D-HI---R---	A	TDH--DA-GQAPL-AA-
	Mycobacterium bovis	WP_019283915	---I----T--V---L---	A	GD---DA-SPSAL-AA-
	Mycobacterium brisbanense	WP_062827312	--------ERHIA--R---	T	-DT--EA-AQ-LLTDT-
	Mycobacterium canettii	WP_014001247	---I----T--V---L---	A	GD---DA-SPSAL-AA-
	Mycobacterium caprae	WP_075744546	---I----T--V---L---	A	GD---DA-SPSAL-AA-
	Mycobacterium celeriflavum	ORA49164	---A----AYH-A--S---	T	-DA--DA--P-A---A-
	Mycobacterium chlorophenolicum	WP_048468373	---A----A-H-A--E---	A	-DG--DAV-VSA--PD-
	Mycobacterium chubuense	WP_048419253	---A----A-H-A--E---	A	-DG--DAV-VSA--PD-
	Mycobacterium colombiense	WP_064878224	---I-----YHI---L---	Y	AD-S-DA-GPAAL--A-
	Mycobacterium conceptionense	CQD21803	---AE---AYH-A--R---	T	-DA--DA--P-AIS-E-
	Mycobacterium confluentis	WP_085151257	---A----A-HVA--I---	H	-DS--EAL-TSAA--E-
	Mycobacterium fallax	WP_085100470	S--I----A-HSA--T---	E	-DA--DA--PTA--GG-
	Mycobacterium farcinogenes	WP_036393629	---AE---AYH-A--R---	T	-DA--DA--P-AIS-E-
	Mycobacterium flavescens	WP_069412922	---A----AYH-A--T---	T	-DA--DR--P-A---AS
	Mycobacterium florentinum	WP_085219651	---A----AYHVA--I---	N	-D---EAL-P-AL-PE-
	Mycobacterium fragae	WP_085199647	---I----TDHV---V---	H	-DG--D-TGPSAL-QT-
	Mycobacterium gilvum	WP_013471366	---A----A-HIA--E---	T	-ES--E-I-PTA--AD-
	Mycobacterium goodii	WP_049745892	---I----ARHIA--R---	T	-EL--D--AP-LL-Q--
	Mycobacterium hassiacum	WP_018354101	---PE---DYHIA--A---	G	-DA--DA--P-A--QA-
	Mycobacterium heckeshornense	WP_048890709	---I----AYHR---I---	G	SD---EG-GPSPL--A-
	Mycobacterium holsaticum	ODQ86703	---A----AYHVA--T---	T	-DA--DA--P-A—Q
	Mycobacterium indicus pranii	WP_014941687	A--I----A-HI---L---	S	ADE--EA-GP-AL--V-
	Mycobacterium insubricum	WP_083028836	---I----E-HIA------	G	--G--EAA-P-A--AD-
	Mycobacterium interjectum	WP_085205342	---I----D-HI---L---	A	AD-V-DA-GP-AL-QT-
	Mycobacterium intracellulare	WP_064933105	A--I----A-HI---L---	S	ADE--DAAGP-AL--A-
	Mycobacterium iranicum	WP_085174075	---A----A-HIA--E---	T	-ESS-EAV-P-A-TSE-
	Mycobacterium komanii	CRL73136	---A----AYH-A--T---	T	-DA--DA--P-A-T-A-
	Mycobacterium lentiflavum	CQD07292	---AE---AYHVA--N---	H	-DA--EA--P-AL-DE-
	Mycobacterium mageritense	WP_036442817	---I----ERHIA--R---	T	-DT--EA-SR-LLTDA-
	Mycobacterium malmesburyense	CRL74174	---A----AYH-A--T---	T	-DA--DA--P-A--D--
	Mycobacterium malmoense	WP_065443766	--------A-HI---L---	S	ADED-DA-GPSPL-AA-
	Mycobacterium mantenii	WP_083095705	----E---A-HI---L---	S	AD---DA-GRAAL--
	Mycobacterium marseillense	WP_083016505	A--I----AYHI---L---	S	AA---DA-GP-AL--A-
	Mycobacterium nebraskense	WP_085164144	--------AYHI---L---	S	SD-S-DV-GPSPL--A-
	Mycobacterium neworleansense	CRZ13592	---AE---AYH-A--R---	T	-DA--DA--P-A-T-Q-
	Mycobacterium noviomagense	ORB18870	S--I----A-HI---A---	G	SDE--DA-GPSAL-VE-
	Mycobacterium novocastrense	WP_084377640	---A----AYH-T--T---	T	-DA--DA--P-A---T-
	Mycobacterium orygis	WP_003412281	---I----T--V---L---	A	GD---DA-SPSAL-AA-
	Mycobacterium palustre	WP_085079968	---I----A-HV---L---	S	TD---DA-GPSAL-DA-
	Mycobacterium paraffinicum	WP_073873776	--------AYHI---L---	S	AD-S-EAIGP-PL-D--
	Mycobacterium parafortuitum	WP_083145208	---A----A-HIA--E---	T	-DS--DAA-L-A-TPH-
	Mycobacterium paraintracellulare	WP_014384380	A--I----A-HI---L---	S	ADE--EA-GP-AL--A-
	Mycobacterium paraseoulense	WP_083169154	--------AYHV---L---	S	TD-S-EAIGP-PL-DA-
	Mycobacterium phlei	WP_003890409	---A----AYHIA--T---	T	-DG--DR--P-A---A-
	Mycobacterium rhodesiae	WP_083117366	---I----A-HI--VQ---	R	ED---DSGRGELL-P--
	Mycobacterium rutilum	SEH73906	---A----AYH-A--T---	T	-DA--DR--P-A---AS
	Mycobacterium saskatchewanense	WP_085254746	---I----AAHV---V---	S	AD---DA--ISCL-AG-
	Mycobacterium septicum	WP_044523068	---AE---AYH----R---	T	-DS--DA--P-AAS---

**Other *Mycobacterium* (0/69)**	*Mycobacterium setense*	WP_064874893	---AE---AYH----R--- T -DA--DA--P-AASGQ-
	Mycobacterium shigaense	BAX93514	---I----A--I---R--- S GD
	Mycobacterium shimoidei	WP_069397079	T--I----A-HI---R--- E --G--D-AS-AAL-P--
	Mycobacterium simiae	WP_061556293	---I----A--I---A--- S GDE--E-SGPSAL-AE-
	Mycobacterium smegmatis	WP_003895882	---I----ARHIA--R--- T -DT--DI-SP-LL-Q--
	Mycobacterium timonense	WP_083187372	---I----AYHI---L--- S AD---DA-GPAAL--A-
	Mycobacterium triplex	WP_036466981	---A----AYHVA--I--- N -D---EA--P-AL-PE-
	Mycobacterium tuberculosis	WP_065022386	---I----T--V---L--- A GD---DA-SPSAL-AA-
	Mycobacterium tusciae	WP_006241090	---A----AYH-A--T--- T -DG--DV--P-A--QA
	Mycobacterium vaccae	WP_003928526	---A----A-H-A--E--- N -ES--EAL--AA-TPE-
	Mycobacterium vulneris	WP_085291612	---I-----YHI---LQ-- Y SD-S-DA-GPAAL--A-
	Mycobacterium wolinskyi	WP_067852022	---I----ARHIA--R--- T -DTS-EA-SL-LLS---
	Mycobacterium xenopi	WP_003921109	---I----A-H----L--- G SD---EAAGPSAL-AA-
	Mycobacterium yongonense	WP_065500066	A--I----A-HI---L--- S ADE--EA-GP-AL--A-

Supplementary Figure 60

A partial sequence alignment of a conserved region of a non-ribosomal peptide synthetase showing a one amino acid deletion that is specific for members of the "*Terrae"* clade and absent in other *Mycobacterium*.

Group	Species	Accession	134		174
**"Terrae" Clade (10/10)**	*Mycobacterium engbaekii*	WP_085126548	EPALPTLLRRLVIMGGAFSGE		LSGEAEFNIGFDPEAAEQVF
	Mycobacterium heraklionense	WP_064890219	---------------------		R--W------------AE--
	Mycobacterium hiberniae	WP_085135166	---------------------		R-AG------------A---
	Mycobacterium icosiumassiliens	WP_067970207	--------H------------		R--W------------AE--
	Mycobacterium kumamotonense	WP_083080213	----------------V----		RADR------------AE--
	Mycobacterium longobardum	WP_085262987	-----------------Y---		R-DR------------AE--
	Mycobacterium nonchromogenicum	WP_085139110	---------------------		R--W------------AE--
	Mycobacterium senuense	WP_085083222	---------------------		RA-W------------AE--
	Mycobacterium sinense	WP_064853534	---------------------		RAERS-----------AE--
	Mycobacterium terrae	WP_085261293	---------------------		RA-RP-----------AE-L
**Other *Mycobacterium* (7/99)**	*Mycobacterium alsense*	WP_083137269	------Q---------S-GHG		TNPL--W--RV-----GE--
	Mycobacterium interjectum	WP_085202349	-----RM-----------G--		ANPM--W--RV-----GEI-
	Mycobacterium kubicae	WP_085073771	------------------DEQ		IDSA--W--RV-----DE-L
	Mycobacterium malmoense	WP_065441460	------------------GPD		TNPM--W--RV-----DE-L
	Mycobacterium paraense	WP_085096305	-----RM---------S-G-D		ANPM--W--RV-----AE--
	Mycobacterium saskatchewanense	WP_085255599	------------------G-T		ANPM--W--RV-----SE--
	Mycobacterium szulgai	WP_068033981	------------------DEQ		IDSA--W--RV-----DE-L
	Mycobacterium abscessus	WP_062879237	--E--K--K-------S-DYP	GN	TTPV--W-VVV--G-TAE-Y
	Mycobacterium acapulcensis	WP_066811221	------------------DYR	GN	TTPV--W-VSV-----AE--
	Mycobacterium aromaticivorans	WP_081845016	--N--S----------S-DYR	GN	TTPV--W--SV-----AE--
	Mycobacterium asiaticum	WP_036352966	--E--R--G-------M-GD-	HG	ADQQ--W-VKV----TAE--
	Mycobacterium aurum	WP_048630166	-----R------------DYR	GN	TTPV--W-TSV---S-AE--
	Mycobacterium austroafricanum	WP_036369877	-----R--G---------DYR	GN	TTPV--W-VSV---S-AE--
	Mycobacterium avium	WP_062886659	----------------SYDHR	GN	TTAV--W--SV-----AE-L
	Mycobacterium avium subsp. paratuberculosis	ETB54757	----------------SYDHR	GN	TTAV--W--SV-----AE-L
	Mycobacterium bacteremicum	WP_083059269	-----R------------DYR	GN	TTPV--W-MSV-----AE--
	Mycobacterium boenickei	WP_077743559	--T--K------------DYR	GN	TTPVS-W--SV---S-AE--
	Mycobacterium branderi	WP_083130041	--E--S--G--------YDYR	GN	TNPV--W--HV-----AE--
	Mycobacterium canariasense	WP_084395520	-----R------------DYR	GN	TTAV--W--SV-----AE--
	Mycobacterium celatum	WP_062540300	-----------------YDYR	GN	TNPV--W--HV-----AE--
	Mycobacterium celeriflavum	WP_083000901	------------------DYR	GN	TTPV--W--SV-----AE--
	Mycobacterium chelonae	WP_070919443	--E--K--K-------S-DYP	GN	TTPV--W-VVV--G-TAE-Y
	Mycobacterium chlorophenolicum	WP_048471491	--E--R-I----------DYR	GN	TTPV--W-TSV---S-AE--
	Mycobacterium chubuense	WP_014814419	-----R--A---------DYR	GN	TTPV--W-VSV---S-AE--
	Mycobacterium colombiense	WP_064880844	--E--A--K-------SYDHR	GN	TTAV--W--SV-----AE-L
	Mycobacterium conceptionense	CQD05982	-----K------------DYR	GN	TTPVS-W-VSV---S-AE--
	Mycobacterium cosmeticum	WP_084172754	-----R------------DYR	GN	TTAV--W--SV-----AE--
	Mycobacterium diernhoferi	WP_073856570	-----R------------DYR	GN	TTAV--W-THV-----AE--
	Mycobacterium doricum	WP_085192318	------------------DYR	GN	TTAV--W-VSV-----AE--
	Mycobacterium elephantis	WP_083043547	-----R------------DYR	GN	TTPV--W-VSV-----AE--
	Mycobacterium europaeum	WP_085242229	------------V---SYDHR	GN	TTAV--W--SV-----AE-L
	Mycobacterium fallax	WP_085095358	--E--R------------DYR	GN	TTPAS-W--AV-----AE--
	Mycobacterium farcinogenes	CDP84701	-----K------------DYR	GN	TTPVS-W-VSV---S-AE--
	Mycobacterium flavescens	ODQ89206	------------------DYR	GN	TTPV--W--SV-----AE--
	Mycobacterium florentinum	WP_085219676	----------------SYDHI	GN	TTAV--W--SV-----AE--
	Mycobacterium fortuitum	OBB02028	--T--K------------DYR	GN	TTPVS-W--SV---S-AE--
	Mycobacterium fragae	WP_085198461	--T--------------YDYR	GN	TNPV--W--SV-----AE--
	Mycobacterium franklinii	WP_070938605	--E--K--K-------S-DYP	GN	TTPV--W-VVV--G-TAE-Y
	Mycobacterium genavense	WP_051465275	--E--N-----A----SYDHI	GN	TTAV--W--SV-----AE--
	Mycobacterium gilvum	WP_011895728	-S---R--K---------DYR	GN	TTPV--W-VSV---S-AET-
	Mycobacterium goodii	WP_049748035	-----A-M----------DYK	GN	TTPV--W--SV---S-AE--
	Mycobacterium haemophilum	WP_047316874	----------------V-D-S	GQ	GNVA--W--RV-----SE--
	Mycobacterium hassiacum	WP_005625659	-----R------------DYR	GN	TTPV--W-VSV-----SE--
	Mycobacterium heckeshornense	WP_048893264	-----R-----------YDYR	GN	TNPV--W--KV-----AE--
	Mycobacterium holsaticum	ODQ84890	-----R------------DYR	GN	TTPV--W--SV-----AE--
	Mycobacterium houstonense	WP_066903924	--T--R------------DYR	GN	TTPVS-W-VSV---S-AE--
	Mycobacterium immunogenum	WP_064627871	--E--K--K-------S-DYP	GN	TTPV--W-VVV--G-TAE-Y
	Mycobacterium indicus pranii	WP_014942853	--T--S----------SYDHR	GN	TTTV--W--SV-----AE-L
	Mycobacterium insubricum	WP_083031138	--E--R----------S-EYR	GN	TTPV--W--AV-----AE--
	Mycobacterium intracellulare	WP_064893689	-----S----------SYDHR	GN	TTAV--W--SV-----AE-L
	Mycobacterium iranicum	OAN29691	--G--G--K---------DYR	GN	TTPV--W-THV---S-AE--
	Mycobacterium komanii	CRL75412	------------------DYR	GN	TTPV--W--SV-----AE--
	Mycobacterium kyorinense	WP_065015037	-----------------YDYR	GN	TNPV--W--SV-----AE--
	Mycobacterium lentiflavum	CQD07384	----------------SYDHF	GN	TTAV--W--SV-----AE--
	Mycobacterium litorale	AQT83014	--S--S----------S-DYR	GN	TTPV--W--SV-----AE--
	Mycobacterium llatzerense	KIU16365	--S--R------------DYR	GN	TTPVS-W--SV-----AE--
	Mycobacterium mageritense	WP_036428871	--E--R------------DYR	GN	TTPVS-W-VSV---S-AE--
	Mycobacterium malmesburyense	CRL78471	--T---------------DYR	GN	TTPV--W--SV-----AE--
	Mycobacterium malmoense	WP_083009488	--E--R----------SYDHR	GN	ITPV--W--SV-----AE-L
	Mycobacterium mantenii	WP_083100009	--E-------------SYDHR	GN	TTAV--W--SV-----AE-L

Group	Species	Accession	Sequence
Other *Mycobacterium* (7/99)	*Mycobacterium marseillense*	WP_083020509	--T-------------SYDHR GN TTAV--W--SV-----AE-L
	Mycobacterium moriokaense	WP_083154023	--T-------------S-DYR GN TTPV--W--SV-----AE--
	Mycobacterium mucogenicum	WP_064858421	--S--R------------DYR GN TTPVS-W--SV-----AE--
	Mycobacterium nebraskense	WP_046187162	-------M----V---SYDHR GN TTAV--W--SV-----AE-L
	Mycobacterium neoaurum	WP_081843384	-----R------------DYR GN TTPV--W-THV-----AE--
	Mycobacterium neworleansense	CRZ15892	--T--K------------DYR GN TTPVS-W-VSV---S-AE--
	Mycobacterium noviomagense	WP_083087845	-----------------YDYR GN TNPV--W--KV-----AE--
	Mycobacterium novocastrense	WP_084377810	Q-----------------DYR GN TTPV--W--SV-----AE--
	Mycobacterium obuense	WP_082133021	--E--K----------S-DYR GN TTPV--W-TSV---S-AE--
	Mycobacterium paraffinicum	OJZ66267	------------V---SYDHR GN TTAV--W--SV-----AE-L
	Mycobacterium parafortuitum	WP_083145817	-----R--K---------DYR GN TTPV--W-THV---S-AE--
	Mycobacterium paraintracellulare	WP_014385744	--T--S----------SYDHR GN TTTV--W--SV-----AE-L
	Mycobacterium paraseoulense	WP_083169677	--E--R------V---SYDHR GN TTAV--W--SV-----AE-L
	Mycobacterium parmense	WP_085270598	--------H-------SYDHR GN TTPV--W--SV-----AE-L
	Mycobacterium peregrinum	WP_064878435	--T--K------------DYR GN TTPVS-W--SV---S-AE--
	Mycobacterium phlei	AMO60287	-----R------------DYR GN TTPV--W--SV-----AE--
	Mycobacterium porcinum	WP_083266838	--T--K------------DYR GN TTPVS-W--SV---S-AE--
	Mycobacterium rhodesiae	ORB48518	--N--S----------S-DYR GN TTPV--W--SV-----AE--
	Mycobacterium rufum	KGI67156	-----R------------DYR GN TTPV--W-TSV---S-AE--
	Mycobacterium rutilum	WP_083410087	------------------DYR GN TTPV--W--SV-----AE--
	Mycobacterium salmoniphilum	WP_078323578	--E--K-IK-------S-DYP GN TTPV--W-VVV--G-TAE-Y
	Mycobacterium saopaulense	WP_070909465	--E--R--K-------S-DYP GN TTPV--W-VVV--G-TAE-Y
	Mycobacterium scrofulaceum	WP_067280541	------------V---SYDHR GN TTAV--W--SV-----SE-L
	Mycobacterium septicum	WP_084621852	--T--K------------DYR GN TTPVS-W--SV---S-AE--
	Mycobacterium setense	WP_064876557	--T--K------------DYR GN TTPVS-W--SV---S-AE--
	Mycobacterium sherrisii	WP_069399085	----------------SYDHV GN TTAV--W--SV-----D---
	Mycobacterium shimoidei	WP_069395346	-----------------YDYR GN TNPV--W--HV-----AE--
	Mycobacterium simiae	WP_061558425	-----R----------SYDHV GN TTAV--W--SV-----DR--
	Mycobacterium smegmatis	WP_003893028	-----R-M----------DYK GN TTPV--W--SV---S-AE--
	Mycobacterium thermoresistibile	EHI10439	--E--R-VK---------DYR GN TTPV--W-VSV----WWE-L
	Mycobacterium triplex	WP_036467008	--E--N----------SYDHI GN TTAV--W--SV-----AE--
	Mycobacterium triviale	WP_085111408	D-E-----D---V----YGRP GE PATA--W--SV-----AE-L
	Mycobacterium tusciae	WP_083124863	-----R------------DYR GN TTPV--W--SV-----GE--
	Mycobacterium vaccae	WP_040540516	-----R--A---------DYR GN TTPV--W-VSV---S-AE--
	Mycobacterium vanbaalenii	WP_011778783	-----R--G---------DYR GN TTPV--W-VSV---S-AE--
	Mycobacterium vulneris	OCB12578	-----K------------DYR GN TTPVS-W--SV---S-AE--
	Mycobacterium wolinskyi	WP_084356367	--G--K------------DYR GN TTPVS-W-VSV---S-AE--
	Mycobacterium xenopi	WP_039889495	-----------------YDYR GN TNPV--W--KV-----AE--
	Mycobacterium yongonense	WP_065507259	--T--S----------SYDHR GN TTTV--W--SV-----AE-L
Other bacteria	*Amycolatopsis orientalis*	WP_043829664	--E--R-V--V-S-A---EVM GN I-DC--S-VWH-----A--V
	Gordonia kroppenstedtii	WP_018179354	D----AR---------T-HHP GN TTPTS-W--AV-----QE--
	Hoyosella subflava	WP_041450986	--D--SM-K---V-----NHP GN TTPV--W--SV-----KL--
	Millisia brevis	WP_084350746	--R--K--N--I------DHP GN TTPT--W--SV-----KE--
	Nocardia alba	WP_067445916	--E--R--N---------NHP GN TTPTN-W--HV-----KE--
	Rhodococcus defluvii	WP_031940701	D-E--QR----IV-----QHP GN TTPV--W--SV--D--AE--
	Segniliparus rotundus	WP_013137358	--E--R------V---S-DYP GN TTPV--W--SV-----KE--
	Streptomyces cattleya	WP_014151949	--R--Q-V-HVTV----VHHP GN I-PV--A---H-----AL-L
	Thermomonas fusca	WP_028838660	D-T--QRIK-C-V--A-V-AH GN I-AA-----A------HI--
	Williamsia muralis	WP_062798103	D-----R---------T-HHP GN TTPTS-W--AV-----KE--

Supplemental Figure 61

A partial sequence alignment of a conserved region of nucleoside hydrolase showing a two amino acid deletion that is specific for members of the "*Terrae*" clade and absent in most other bacteria.

Group	Species	Accession	165		203
"Terrae" Clade (10/10)	*Mycobacterium algericum*	WP_083037632	RNVMARNLLMHTCADHERALASGSSA		SISWGEAGSGLID
	Mycobacterium engbaekii	ORV41155	--------------E----V-A-V--		AA--A-S--D---
	Mycobacterium heraklionense	WP_047318275	--------------E--------A--		-A--T----D---
	Mycobacterium hiberniae	WP_085137265	--------------E----V-A-V--		AA--A-S--D---
	Mycobacterium kumamotonense	WP_065287151	---------------W--T--A-GR-		RT--RK-S-H---
	Mycobacterium longobardum	WP_085263279	---------------------A----		-L--A----D---
	Mycobacterium nonchromogenicum	WP_085137607	--------------E--------VPG		-A--T----D---
	Mycobacterium senuense	WP_085084391	-----------------------G--		----A--------
	Mycobacterium sinense	OBH16961	---------------W--T--A-GK-		RTN-QQ-S-N---
	Mycobacterium terrae	WP_085261723	-----------------------A-T		----A--------
Other *Mycobacterium* (1/97)	*Mycobacterium branderi*	WP_083133925	---------------R---M--R-AP		RT--QA-------
	Mycobacterium alsense	WP_083138978	-I--V---------E--G---EHGPR	S	RS--PV-AE----
	Mycobacterium angelicum	WP_083111814	--I------------L-----T-T--	P	RR--RT-AT----
	Mycobacterium aromaticivorans	WP_036344187	-KI-G----I----EY-----E--AL	P	LTN-RT-A---V-
	Mycobacterium arosiense	WP_083063608	--I------------R-----A-AVT	S	HR--RA-A-----
	Mycobacterium asiaticum	WP_065035685	-I------------E--G---EHGPH	A	RSG-PV--EN---
	Mycobacterium aurum	WP_083443219	--I------V-----F---F-D-ADL	P	RT--SAV------
	Mycobacterium avium	WP_062887311	--I------------R-----A-T-T	S	HR--RA-A-----
	Mycobacterium avium subsp. avium	EUA27668	-M--V---------E--G---EHGPR	S	RSA-PV-AE----
	Mycobacterium avium subsp. hominissuis	BAN29802	--I------------R-----A-T-T	S	HR--RA-A-----
	Mycobacterium avium subsp. paratuberculosis	ETB50975	-M--V---------E--G---EHGPR	S	RSA-PV-AE----
	Mycobacterium bacteremicum	ORA05079	--I--------S---R----TADPV-	A	VRT-SA--A----
	Mycobacterium boenickei	WP_077741559	--I--------S---R---M-Q-GNV	P	RA--QG-A-----
	Mycobacterium bohemicum	WP_085181647	-I--V---------E--A---EHGPR	S	RS--PV--E----
	Mycobacterium brisbanense	WP_062832110	--I--------S---R--G--R-A-V	P	RA--SD-A---V-
	Mycobacterium chlorophenolicum	WP_048473302	--I-G------S--ER--L--Q-APV	A	RP--QG-A-----
	Mycobacterium chubuense	WP_041781984	-I------------E--G---QHGPR	A	RSP-PL-AD--T-
	Mycobacterium colombiense	WP_040629903	-M--V---------E--G---EHGPR	S	RSA-PV--E----
	Mycobacterium conceptionense	WP_076213322	--I--------S---R---M-Q-GNV	P	RA--QG-A-----
	Mycobacterium confluentis	WP_085152762	--A---H-IV-MF-EQ-----Q-DPM	A	RPG-DGTAD--V-
	Mycobacterium conspicuum	WP_085234628	--I------I-----L--S--A-A-L	P	RP--RA-AT-I--
	Mycobacterium diernhoferi	OJZ68091	--I--------S---R----TA-AAP	A	GRT-AA--A----
	Mycobacterium elephantis	WP_083042687	--I-V---M------F---F-E-TDM	A	RTN-RSVA-----
	Mycobacterium europaeum	WP_085239002	-M--V---------E--G---EHGPR	S	RSA-PV--E----
	Mycobacterium farcinogenes	CDP88778	--I--------S---R---M-Q-GNV	P	RA--QG-A-----
	Mycobacterium flavescens	ODQ89930	--I-V---M------F---F-E-ADM	P	RTT-SSVA-----
	Mycobacterium florentinum	WP_085223720	--I------------R-----A-AAM	A	QP--RA-AF----
	Mycobacterium fortuitum	WP_061264232	--I--------S---R---M-Q-GNV	P	RA--QG-A-----
	Mycobacterium fragae	WP_085198974	--L------------R-------A-V	P	RS--QA-------
	Mycobacterium gastri	WP_036416139	--L------------L-----T-T-T	P	RP--
	Mycobacterium genavense	WP_025734998	-I--V---------E--G---EHGQR	T	RSA-PV--E----
	Mycobacterium gilvum	WP_041787789	--I-G------S---R--L--Q--PV	P	RA--QA-AT----
	Mycobacterium goodii	WP_049745635	--I--------S---R-----Q-APV	A	RS--QG-A-----
	Mycobacterium gordonae	WP_065045250	--I------------L-----E-T-M	P	RA--RA-AT----
	Mycobacterium hassiacum	WP_018353845	--I------------F---F-E-TA-	P	RT--SSVA-----
	Mycobacterium heckeshornense	WP_048890126	-----V-----F-----T-APV	P	RP--DA-A-----
	Mycobacterium heidelbergense	WP_083072181	--I------------R----SA-P-I	H	QT--RA-A-----
	Mycobacterium holsaticum	WP_069406732	--I-V---M------F---F-E-ADI	R	RSN-SSVA-----
	Mycobacterium houstonense	WP_066898546	--I--------S---R---M-Q-ADV	P	RA--QG-A-----
	Mycobacterium interjectum	WP_066912313	-I--V---------E--G---EHGPR	S	RS--PV--E----
	Mycobacterium intermedium	WP_069418129	--I------------F-----A-TA-	P	HW--RA-AT----
	Mycobacterium intracellulare	WP_064933251	-M--V---------E--G---EHGPR	S	RSA-PV--E----
	Mycobacterium iranicum	OAN38567	--I------V-----F---F-D-ADL	P	RT--SAV------
	Mycobacterium kansasii	WP_082276548	--L------------L-----T-T-T	P	RP--RA-ATA---
	Mycobacterium komanii	CRL68343	--I-----M------F---F-E-ADM	P	RT--DS-A-----
	Mycobacterium kubicae	WP_085075364	--I------------L-----D-T-T	P	RP--RA-AT----
	Mycobacterium kyorinense	WP_045377034	---------------R---M-R-T--	S	RS--QA-------
	Mycobacterium lacus	WP_085156026	--I------------R-----A-T-T	H	QS--RA-A-----
	Mycobacterium lentiflavum	CQD13948	-I--V---------E--G---EHGPR	S	RS--PV--E----
	Mycobacterium liflandii	WP_015357193	--L------------L-----A-T-V	P	RP--R--AT----
	Mycobacterium mageritense	WP_081812663	--I--------S---R--G--Q-ATV	P	RP--NA-A-----
	Mycobacterium malmesburyense	CRL69480	--I-----M------F---F-E-ADM	P	RTT-DS-A-----
	Mycobacterium malmoense	WP_065444635	-M--V---------E--G---EHGPR	A	RSA-PV--E----
	Mycobacterium mantenii	WP_083093794	--I------------R-----A-A-T	F	HR--RA-A-----

Other *Mycobacterium* (1/97)	*Mycobacterium marinum*	WP_012396302	--L-----------L-----A-T-V P RP--R--AT----
	Mycobacterium marseillense	WP_083014937	--I-----------R----VA-ALT S HR--RA-A-----
	Mycobacterium moriokaense	WP_083154633	--I-V---M------F---F-E-TAM P RTT-RSVAT----
	Mycobacterium nebraskense	WP_046183631	-M--V---------E--S---EHGPR A RSA-PV--E----
	Mycobacterium neoaurum	WP_030137328	--I--------S---R----NT--MP V VRT-SA--G----
	Mycobacterium neworleansense	CRZ13252	--I--------S---R---M-Q-GNV P RA--QG-AA----
	Mycobacterium noviomagense	WP_083087477	---------V-----------R-A-V P RP--HA-A-----
	Mycobacterium novocastrense	GAT11960	-I-----------E--G---HHGRL A RSP-KQ-AE--T-
	Mycobacterium palustre	WP_085076442	--I-----------R-----A--TM H QT--RA-A-----
	Mycobacterium paraense	WP_085093176	--I-----------R-----A---M H QS--RA-A-----
	Mycobacterium paraffinicum	WP_073876749	--I-----------R-----A-A-P P HR--RA-A-----
	Mycobacterium parafortuitum	ORB28292	--I------V-----V---F-D-AAL P RTN-AAV------
	Mycobacterium parascrofulaceum	EFG79345	-M--V---------E--S---EHGPR A RSA-PV--E----
	Mycobacterium paraseoulense	WP_083168411	--I-----------R-----A-A-P L QR--RA-A-----
	Mycobacterium parmense	WP_085270172	--I-----------R-----A-AAP P HR--RA-A-----
	Mycobacterium peregrinum	WP_055116005	--I--------SA--R---M-Q-GTV P RA--QG-A-----
	Mycobacterium phlei	WP_081491197	--I-----------F---F-E-ADV P RTT-SSVA-----
	Mycobacterium porcinum	WP_075921106	--I--------S---R---M-Q-GNV P RA--QG-A-----
	Mycobacterium pseudoshottsii L	GAQ35406	--L-----------L-----A-T-V P RP--R--AT----
	Mycobacterium rhodesiae	WP_014212189	--I-V---M------F---F-E-AAM P RTT-SSVA-----
	Mycobacterium riyadhense	WP_085250625	--I-----------L-----A-A-M P RH--RA-AT----
	Mycobacterium rufum	KGI69204	--I-G------S--ER--L--Q-APV A RP--QA-A-----
	Mycobacterium rutilum	WP_083407985	--I-V---M------F---F-E-ADM P RTT-SSVA-----
	Mycobacterium saskatchewanense	WP_085255802	--I-----------R-------A-P A HR--RA-A-----
	Mycobacterium scrofulaceum	WP_067273868	--I-----------R-----A-A-P P HR--RA-A-----
	Mycobacterium septicum	WP_044524107	--I--------S---R---M-Q-GNV P RA--QG-A-----
	Mycobacterium setense	WP_064871340	--I--------S---R---M-Q-GNV P RA--QG-A-----
	Mycobacterium sherrisii	WP_069401713	-I--V---------E--G---EHGPR A RSA-PV--E----
	Mycobacterium simiae	WP_061557122	--I-----------R-----A-AAM V QS--RA-A-----
	Mycobacterium smegmatis	WP_003896256	--I--------S--ER-----Q-APV P RS--QG-A-----
	Mycobacterium szulgai	WP_068031195	--I-----------L-----D-T-T P RP--RA-AT----
	Mycobacterium thermoresistibil	WP_003926125	--I-----------F---F-E-DPV P RA--QAV-N----
	Mycobacterium triplex	CDO88695	-I--V---------E--G---EHGPR S RS--PV--E----
	Mycobacterium tuberculosis TKK	KBZ59513	--I-----------R-----A-AVT S HR--RA-A-----
	Mycobacterium ulcerans	WP_011738686	--L-----------L-----A-T-V P RPF-R--AT-Q--
	Mycobacterium vaccae	WP_040539666	--I-G----------R--L--Q--PV P RT--QA-A-----
	Mycobacterium vulneris	WP_065512794	--I--------S---R---M-Q-GNV P RA--QG-A-----
	Mycobacterium wolinskyi	WP_085143787	--I--------S---R-----Q-A-V P RA--QG-A-----
	Mycobacterium xenopi	WP_085194404	---------V-----Y-----T-TPV P RP--DT-A-----
	Mycobacterium yongonense	WP_065500145	-M--V---------E--G---EHGPR S RSA-PV-AE----
Other bacteria	*Actinoalloteichus cyanogriseus*	WP_030225923	-AD-V-H-IL-V--ER-----E-EVT P RAG-DG-AT--V-
	Amycolatopsis orientalis	WP_037370775	-SD-T-T--V-LT-ER-----ENTPT P RAG-S--AT----
	Microtetraspora malaysiensis	WP_067137185	-GE-TSQ-MV-I--ER-----E-RPT P RS--HD-AT--V-
	Nocardia transvalensis	WP_040749118	-T--T------S--EI-GE--ARGRR G RVN-R-V-ED-T-
	Nocardiopsis halotolerans	WP_040685038	-QD---Q-MV-MI-ER-----E-GRT P RD--HR-AV--T-
	Nonomuraea pusilla	SEK70628	--A--SQ-MV-M--ER-----E-LPT P RP--DD-AT----
	Pseudonocardia autotrophica	WP_037043089	-GD-T-L--V-V--AR-----E-APT P RTT-DATAT--V-
	Rhodococcus yunnanensis	WP_072802544	-SD----MM--M--EK-LEFSQSHPE C PAN-TDF-TD---
	Saccharomonospora saliphila	WP_019818790	-G----H-IV-M--ER-----D-TLT P RA--Q--AN--V-
	Streptoalloteichus hindustanus	WP_073483206	-AA---Q--V-MV-ER-----E-APT P RA--HD-AT--V-
	Streptomonospora alba	WP_052809356	-SE--GH-VV-M-EQR--R--R-TTT A KH--QS-AV--V-
	Streptosporangium canum	SFK59198	-GD---Q-MV-MY-ER-----E-APT P RS--HD-AT----

Supplementary Figure 62

A partial sequence alignment of a conserved region of TetR/AcrR family transcriptional regulator showing a one amino acid deletion that is specific for members of the “*Terrae”* clade and absent in most other bacteria.

Group	Species	Accession	593		639
"Terrae" Clade (12/13)	*Mycobacterium algericum*	WP_083035732	QLLAAIALTVVTVIVIKR	SRRR	SDLKWALVPGIPLLWDLTVTMTASW
	Mycobacterium arupense	WP_046189959	------------------	G-DG	AG-----I-----------------
	Mycobacterium engbaekii	WP_085130341	-----------------K	--GS	FS-----------------------
	Mycobacterium heraklionense	OBI02997	-----------------K	GAGT	-G-----I-----------------
	Mycobacterium hiberniae	WP_085137479	-----------------K	--GS	FG-----------------------
	Mycobacterium icosiumassiliensis	WP_067971062	-----------------K	GNGK	-G-----I-----------------
	Mycobacterium kumamotonense	WP_065288870	------------------	G--T	-G---------------A-------
	Mycobacterium minnesotense	WP_083024464	------------------	G-GG	AG-----I-----------------
	Mycobacterium nonchromogenicum	WP_085138779	-----------------K	GAGT	-G-----I-----------------
	Mycobacterium senuense	WP_085086124	------------------	--H-	-------I-----------------
	Mycobacterium sinense	WP_064854724	---------------V--	G-GT	-N---------------S-------
	Mycobacterium terrae	WP_085262038	------------------	G--T	-G---------------A-------
	Mycobacterium longobardum	WP_085264413	----------------RH		AG-----I-----------------
Other *Mycobacterium* (0/92)	*Mycobacterium abscessus*	WP_062878717	------------T--V-Q		GLY---WI-AL--G---I-------
	Mycobacterium abscessus subsp. bolletii	EIU65159	------------T--V-Q		GLY---WI-AL--G---I-------
	Mycobacterium acapulcensis	WP_066809481	-------------V-V-K		GL----WI-A-------I-------
	Mycobacterium africanum	WP_013988970	----G-----I--V---K		GR----WI---------A--L----
	Mycobacterium angelicum	WP_083111986	--------S-I--V---K		GL----WI--V------V--L----
	Mycobacterium aromaticivorans	WP_036339478	-------------V----		GL----WI-A----------L----
	Mycobacterium arosiense	WP_083064372	----------I----F-K		GL---VW---V------A--L----
	Mycobacterium asiaticum	OBI98281	------------AV----		GLW---WI--L------A--L----
	Mycobacterium aurum	WP_048631937	-------------VI---		GL----WI-----A---V-------
	Mycobacterium avium	WP_062908766	----------I----V-K		GL----WI--A------V--LS---
	Mycobacterium avium subsp. avium	EUA36639	----------I----V-K		GL----WI--A------V--LS---
	Mycobacterium avium subsp. paratuberculosis	ELP45236	----------I----V-K		GL----WI--A------V--LS---
	Mycobacterium bacteremicum	WP_083056907	-------------V---K		GL----WI--L--A---I-------
	Mycobacterium boenickei	WP_077743904	-------------V----		GLV---WI--V--------------
	Mycobacterium bohemicum	WP_085180638	----G--------V---K		GL-R--WI--L------A--L----
	Mycobacterium bovis	WP_080728682	----G--P--I--V---K		GR----WI---------A--L----
	Mycobacterium brisbanense	WP_062829832	-------------V----		GLV---WI--V--M---V-------
	Mycobacterium canariasense	WP_062656248	-------------VI---		GLV---WI--V-------------F
	Mycobacterium canettii	WP_014001563	----G-----I--V---K		GR----WI---------A--L----
	Mycobacterium chlorophenolicum	WP_048470194	-------------V----		GL----WI-----T---I-------
	Mycobacterium chubuense	WP_014815035	-------------VI---		GL-M--WI--V------I-------
	Mycobacterium colombiense	WP_064877464	----------I------K		GL----W---A------V--L----
	Mycobacterium conceptionense	CQD12322	-------------V----		GLV---WI--V--------------
	Mycobacterium confluentis	WP_085148082	-------------V---K		GH----WI--V------I-------
	Mycobacterium conspicuum	WP_085232276	----------I----V-K		RL----WI--V------V--L----
	Mycobacterium diernhoferi	WP_073856177	-------------VI--K		GY----WIS-L--A---V-------
	Mycobacterium elephantis	WP_083043127	-------------V---K		GL----W---V------VI-L----
	Mycobacterium europaeum	WP_085241483	-------------V---K		GL----WI------------L----
	Mycobacterium fallax	WP_085096895	-------------V----		GL-R--WI--L--A---V-------
	Mycobacterium flavescens	WP_069411810	-------------V-V-K		GLI---WI--V------V-------
	Mycobacterium florentinum	WP_085226061	----------I------K		GL-R--WI--V---------L----
	Mycobacterium fortuitum	WP_064850627	-----------S-V----		GLV---WI--L--------------
	Mycobacterium franklinii	OHU30859	------------T--V-Q		RLYT--WI-AL--G---I-------
	Mycobacterium gastri	WP_036420282	------S---I--V---K		GR----WI--V------A--L----
	Mycobacterium gilvum	WP_011895166	-------------VI---		GL----WI--V------I-------
	Mycobacterium goodii	WP_049749096	-------------V----		GLV---WI-AV--------------
	Mycobacterium gordonae	WP_065043009	----G-S--II------K		GH----WI--V------S--L----
	Mycobacterium haemophilum	WP_047313110	----G-S---I--V---K		GL----CI--V---G--A--LS---
	Mycobacterium houstonense	WP_066903152	-------------V----		GLV---WI--L--F-----------
	Mycobacterium insubricum	WP_083029483	----------A--V----		GL-R--WI--L--A-----------
	Mycobacterium interjectum	WP_066909092	----GMS---I-AV---K		GL----WI---------A--L----
	Mycobacterium intermedium	WP_069420982	-----MS---L--V---K		GL----F----------A--L----
	Mycobacterium iranicum	WP_064280749	-------------VI---		GL----WI-----------------
	Mycobacterium kansasii	KZS58773	------S---I--V---K		GH----WI--V------A--L----
	Mycobacterium komanii	CRL66484	-------------V-V-K		GL----WI-AV--------------
	Mycobacterium kubicae	WP_085073475	----G-----I--V-V-L		GR----WI--V------A--L----
	Mycobacterium lacus	WP_085160590	----------I--V---K		GR-N--WI--V------A--L----
	Mycobacterium lentiflavum	CQD09726	----------IA-----K		GL-I--WI--A---------L----
	Mycobacterium liflandii	WP_051045782	------S---I------K		GH----WI--V------I--L----
	Mycobacterium litorale	WP_078020207	-------------V---K		GL----WI--V---------L----
	Mycobacterium mageritense	WP_036427829	-------------V----		GLA---WI-----------------

Group	Species	Accession		
Other *Mycobacterium* (0/92)	*Mycobacterium malmesburyense*	CRL71696	-------------V-V-K	GL----WI-AV--------------
	Mycobacterium malmoense	WP_065442517	-----V----I------K	GL----WI--L------A--L----
	Mycobacterium mantenii	WP_083092814	----------I-A----K	GL-R--WI--V------V--L----
	Mycobacterium marinum	WP_012393449	------S---I------K	GH----WI--V------I--L----
	Mycobacterium moriokaense	WP_083150377	-------------V---K	GLY---W---V---------L----
	Mycobacterium nebraskense	WP_046186791	-----V----I------K	GL----WI---------A--L----
	Mycobacterium neoaurum	WP_030135840	-------------VI---	GL----WI-----A---V-------
	Mycobacterium neworleansense	CRZ15303	-------------V----	GLV---WI--A--------------
	Mycobacterium noviomagense	WP_083088375	----G-----I--V---K	GH----WI--A------A--L----
	Mycobacterium novocastrense	WP_067394996	-------------V-V-K	GL----WI-A---------------
	Mycobacterium obuense	WP_046363893	-------------V----	GLV---WI--V------I-------
	Mycobacterium palustre	WP_085077339	----G-----I--V---K	GL-R--WI--V------A--L----
	Mycobacterium paraense	WP_085093712	-------------V---K	GL-R--WI--A------A--L----
	Mycobacterium paraffinicum	WP_073872014	----------I--V---K	GL-R--WI--L------A--L----
	Mycobacterium parafortuitum	WP_083141453	-------------V----	GL----WI-----------------
	Mycobacterium paraseoulense	WP_083175133	-----------------K	GLV---WI------------L----
	Mycobacterium parmense	WP_085267908	-------------V-V-K	GL-R--WI------------L----
	Mycobacterium peregrinum	WP_064887826	-------------V----	GLV---WI--L--------------
	Mycobacterium porcinum	WP_075919691	-------------V----	GLV---WI-----------------
	Mycobacterium pseudoshottsii L15	GAQ41290	------S---I------K	GH----WI--V------I--L----
	Mycobacterium rhodesiae	WP_083116645	-------------V----	GL----WI-A----------L----
	Mycobacterium riyadhense	WP_085252003	---------II----V-L	GR----WI---------A--LS---
	Mycobacterium rufum	KGI67581	-------------VI---	GQ----WI-----M---I-------
	Mycobacterium salmoniphilum	WP_078325557	-----------LTVTV-Q	RLYT--WI-----A-------A---
	Mycobacterium saskatchewanense	WP_085255015	----------I----V-K	GL-R--WI--V------A--L----
	Mycobacterium scrofulaceum	WP_067274746	----------I--V---K	GL-R--WI--L--V---A--L----
	Mycobacterium septicum	WP_044517293	-------------V----	GLV---WI--V--------------
	Mycobacterium setense	WP_064873158	-------------V----	GLV---WI--L--------------
	Mycobacterium sherrisii	WP_085166942	----------I------K	GL-A--W-S-V--AG--V--L----
	Mycobacterium shinjukuense	ORB66583	------S---I----V-K	GR----WI--V------A--L----
	Mycobacterium simiae	WP_044510451	----------I------K	GL-A--W-S-V--A---V--L----
	Mycobacterium smegmatis	WP_003893630	-------------V----	GLI---WI-AL--M-----------
	Mycobacterium szulgai	WP_085669591	--------S-I--V---K	GL----WI--A------V--L----
	Mycobacterium thermoresistibile	WP_003924902	-------------V---K	GH----WI--V---------L----
	Mycobacterium timonense	ORB81846	----------I----V-K	GL----WI--A------V--LS---
	Mycobacterium triplex	CDO87690	----------I--V---K	GL-R--WI--V------I--L----
	Mycobacterium tuberculosis	WP_070897891	----G-----I--V---K	GR----WI---------A--L----
	Mycobacterium ulcerans str. Harvey	EUA85256	------S---I------K	GH----WI--V------I--L----
	Mycobacterium vaccae	WP_003928779	-------------V----	GL----WI---------V-------
	Mycobacterium vulneris	WP_065462740	-------------V----	GLV---WI-----------------
	Mycobacterium xenopi	WP_085196409	----------T--V---K	GH---SWI--L------A--L----
Other bacteria	*Brevibacterium epidermidis*	WP_062244401	--------A--FA--C-M	GLA---WI-----V---L---A---
	Geodermatophilus ruber	SFK58182	---------LG--LL--H	GKI-Y-W-T-V--V---V------Y
	Gordonia polyisoprenivorans	WP_081484727	-----V--------LF--	GQA---WI-----V--VI-------
	Microtetraspora fusca	WP_066945191	---------LC-TLL--S	GK-R--W-TA------AA--L----
	Nocardia amikacinitolerans	WP_067788360	-----V-----MT-IV-K	GLA---WI-----A---I-------
	Pseudonocardia spinosispora	WP_051342519	-----V--L-C-TVL--S	GRV---W---L--VF--A--L---Y
	Raineyella antarctica	SDB90244	-----------F-V-M-K	GLF---WI--L--A---FI--W---
	Rhodococcus triatomae	WP_007532451	-------------V-M--	GLF--VWI-AV--V---V-------
	Saccharomonospora cyanea	WP_005455942	-----V--A-CVT-LC-T	GRVRY-W------V--VV--L----
	Skermania piniformis	WP_066468778	-----------LT--V-K	GM-R--WI-A---V-----------
	Solirubrobacter soli	WP_037496588	-----V----C--LL--H	GK----W-T----A--A---L----
	Streptomyces albulus	WP_064068600	-----V--A-C-TLLV-S	GR----W-T-V--A--VA--L----
	Tetrasphaera jenkinsii	WP_048544734	------------A-LV-Q	GK----WI-----A---I--LS--Y
	Thermobispora bispora	WP_013131820	-----V----A-TALV-S	GR----W-T-V--A--AA--L----
	Thermocrispum agreste	WP_028847310	----------A---LV-S	GK----W-TAV--A--AA--L----
	Williamsia muralis	WP_062800764	---------I-L---L--	GLF---WI-----A---I---S---

Supplementary Figure 63

A partial sequence alignment of a conserved region of carbon starvation protein A showing a four amino acid insertion that is specific for most members of the "*Terrae"* clade and absent in other bacteria.

Group	Species	Accession	98		124
"Terrae" Clade (9/9)	*Mycobacterium arupense*	WP_046686430	VRFAEAAREL	SSSGL	EIGTVFGAELSL
	Mycobacterium engbaekii	WP_085126604	-------A--	QAA--	------------
	Mycobacterium heraklionense	WP_064889008	-------A--	DAA--	Q-----------
	Mycobacterium hiberniae	ORV70643	-------A--	QAA--	------------
	Mycobacterium kumamotonense	WP_065287307	-------A--	AAA-Q	G-A---------
	Mycobacterium longobardum	WP_085262978	-------K--	GAA--	------------
	Mycobacterium senuense	WP_085083241	-------A--	AAA-Q	--A--Y------
	Mycobacterium sinense	WP_064853526	-------A--	AAA-H	Q-A---------
	Mycobacterium terrae	WP_085260980	-------A--	AAA-H	Q-A---------
Other *Mycobacterium* (0/>100)	*Mycobacterium abscessus*	AMU22417	-------K--		GLP---------
	Mycobacterium abscessus subsp. bolletii	EUA66851	-------K--		GLP---------
	Mycobacterium acapulcensis	WP_066810469	----------		-MS---------
	Mycobacterium africanum	WP_049958458	-------A--		DVR---------
	Mycobacterium alsense	WP_083141122	-------A--		-LR---------
	Mycobacterium angelicum	WP_083115636	-------A--		DVR---------
	Mycobacterium aromaticivorans	WP_036340629	-------K--		DMR---------
	Mycobacterium asiaticum	WP_065036050	-------A--		DVR---------
	Mycobacterium aurum	WP_048630169	----------		GMA---------
	Mycobacterium avium	WP_062889850	-------A--		DVR---------
	Mycobacterium avium subsp. avium	EUA36654	-------A--		DVR---------
	Mycobacterium avium subsp. paratuberculosis	EG038685	-------A--		DVR---------
	Mycobacterium bacteremicum	WP_083058923	----------		DVA---------
	Mycobacterium boenickei	WP_077743360	----------		DVA---------
	Mycobacterium bohemicum	WP_085180143	-------A--		D-R---------
	Mycobacterium bovis	WP_024456539	-------A--		DVR---------
	Mycobacterium branderi	WP_083130033	-------A--		DMA---------
	Mycobacterium brisbanense	WP_062830175	----------		DVA---------
	Mycobacterium canariasense	WP_062659852	----------		DVA---------
	Mycobacterium canettii	WP_015288522	-------A--		DVR---------
	Mycobacterium celeriflavum	WP_083000891	-------K--		-MS---------
	Mycobacterium chelonae	WP_070919441	-------K--		GLP---------
	Mycobacterium chlorophenolicum	WP_048471498	----------		D-A---------
	Mycobacterium chubuense	WP_014814423	----------		DVA---------
	Mycobacterium colombiense	WP_064952082	-------A--		D-R---------
	Mycobacterium conceptionense	CQD05943	----------		DMA---------
	Mycobacterium conspicuum	WP_085234185	-------A--		DVR---------
	Mycobacterium cosmeticum	CD009932	----------		DVA---------
	Mycobacterium diernhoferi	OPE45270	----------		GMS---------
	Mycobacterium doricum	WP_085192447	-------K--		GMR---------
	Mycobacterium elephantis	WP_046752491	-------K--		NMS---------
	Mycobacterium europaeum	WP_085242212	-------A--		-LR---------
	Mycobacterium fallax	WP_085095346	--L-------		-LA---------
	Mycobacterium farcinogenes	CDP84693	----------		DMA---------
	Mycobacterium flavescens	WP_069414591	-------K--		QMS---------
	Mycobacterium fortuitum	WP_061262631	----------		DVA---------
	Mycobacterium franklinii	WP_078333058	-------K--		GLP---------
	Mycobacterium gilvum	WP_013472635	-------K--		DVD---------
	Mycobacterium goodii	WP_049748029	----------		DVA---------
	Mycobacterium hassiacum	WP_005625680	----------		QMA---------
	Mycobacterium heckeshornense	WP_048893269	-------A--		GVA---------
	Mycobacterium immunogenum	KIU41565	-------K--		GLP---------
	Mycobacterium insubricum	WP_083031132	--L-------		-LA---------
	Mycobacterium interjectum	WP_066907857	-------A--		DLS---------
	Mycobacterium intermedium	WP_069422144	-------A--		DVR---------
	Mycobacterium iranicum	WP_024446328	----------		DMA---------
	Mycobacterium kansasii	WP_063471954	-------A--		DMP---------
	Mycobacterium komanii	CRL75427	-------K--		DMS---------
	Mycobacterium kubicae	WP_085073748	-------H--		G-Q---------
	Mycobacterium kyorinense	WP_045374632	-------A--		D-A---------
	Mycobacterium lacus	WP_085162089	-------A--		DVR---------
	Mycobacterium lentiflavum	CQD07422	-------A--		DVR---------
	Mycobacterium liflandii	WP_015354759	-------SG-		GVR---------
	Mycobacterium llatzerense	WP_071287193	-------K--		DMA---------
	Mycobacterium mageritense	WP_036428854	----------		DVA---------

Group	Species	Accession		
Other *Mycobacterium* (0/>100)	*Mycobacterium malmesburyense*	CRL78477	-------K--	DMS---------
	Mycobacterium malmoense	WP_065441450	-------A--	-LR---------
	Mycobacterium marinum	WP_081435777	-------SG-	GVR---------
	Mycobacterium marseillense	WP_083020505	-------A--	D-R---------
	Mycobacterium minnesotense	WP_083023565	----------	-MA---------
	Mycobacterium moriokaense	WP_083154006	----------	DMS---------
	Mycobacterium mucogenicum	WP_064860882	-------K--	DMA---------
	Mycobacterium nebraskense	WP_047323799	-------A--	-LR---------
	Mycobacterium neoaurum	CDQ46318	----------	DVD---------
	Mycobacterium neworleansense	CRZ15882	----------	DVA---------
	Mycobacterium nonchromogenicum	WP_085139817	-------A--	DMA---------
	Mycobacterium noviomagense	WP_083087885	-------A--	DVA---------
	Mycobacterium novocastrense	WP_067396136	-------K--	DMS---------
	Mycobacterium obuense	WP_046361729	----------	DMA---------
	Mycobacterium palustre	WP_085079376	-------A--	GLR---------
	Mycobacterium paraense	WP_085096291	-------A--	-LN---------
	Mycobacterium paraffinicum	WP_073880226	-------A--	-LR---------
	Mycobacterium parafortuitum	WP_083145813	----------	DMA---------
	Mycobacterium parascrofulaceum	EFG76163	-------A--	-LR---------
	Mycobacterium paraseoulense	WP_083169689	-------A--	-LR---------
	Mycobacterium peregrinum	WP_064880190	----------	DVA---------
	Mycobacterium phlei	WP_003886503	----------	DMA---------
	Mycobacterium porcinum	WP_069425015	----------	DVA---------
	Mycobacterium pseudoshottsii L15	GAQ35454	-------SG-	GVR---------
	Mycobacterium rhodesiae	WP_083122262	-------K--	DMR---------
	Mycobacterium riyadhense	WP_085252924	-------A--	DVR---------
	Mycobacterium rufum	KGI67159	----------	D-A---------
	Mycobacterium rutilum	WP_083410084	-------K--	QMS---------
	Mycobacterium salmoniphilum	WP_078323584	-------K--	GLP---------
	Mycobacterium saopaulense	ALR12832	-------K--	GLP---------
	Mycobacterium saskatchewanense	WP_085255605	-------A--	-MR---------
	Mycobacterium scrofulaceum	WP_067280566	-------A--	-LR---------
	Mycobacterium septicum	WP_044516421	----------	DVA---------
	Mycobacterium setense	WP_064876516	----------	DVA---------
	Mycobacterium sherrisii	WP_069399079	-------A--	DVR---------
	Mycobacterium shimoidei	WP_069395339	-------A--	DMA---------
	Mycobacterium shinjukuense	WP_083052309	-------A--	DVR---------
	Mycobacterium simiae	WP_061558431	-------A--	DVP---------
	Mycobacterium smegmatis	WP_003893039	----------	DVA---------
	Mycobacterium szulgai	WP_068162637	-------H--	G-Q---------
	Mycobacterium thermoresistibile	WP_003928094	----------	GVL---------
	Mycobacterium tuberculosis	WP_061140135	-------A--	DVR---------
	Mycobacterium tusciae	WP_083124873	-------K--	DMQ---------
	Mycobacterium ulcerans	OIN34661	-------SG-	GVR---------
	Mycobacterium vaccae	WP_003929282	----------	DVA---------
	Mycobacterium vulneris	WP_065458707	----------	DVA---------
	Mycobacterium wolinskyi	WP_085146753	----------	DVA---------
Other bacteria	*Actinokineospora terrae*	SER80098	---------V	GVR----T----
	Amycolatopsis marina	SFB32458	----------	GVR----T----
	Corynebacterium glucuronolyticum	WP_084036650	------DT	GLE---------
	Couchioplanes caeruleus	WP_071809679	---SQ-----	GLP-I-------
	Frankia alni	WP_011603345	---------V	GLR------I--
	Gordonia namibiensis	WP_006865970	---------F	AMP---------
	Nocardia otitidiscaviarum	WP_029923803	---------W	GMP---------
	Pseudonocardia autotrophica	WP_037046320	----------	GVR---------
	Rhodococcus phenolicus	WP_068154301	----------	KVP-------T-
	Tsukamurella pseudospumae	WP_068570722	I---------	GVP---------

Supplementary Figure 64

A partial sequence alignment of a conserved region of error-prone DNA polymerase showing a five amino acid insertion that is specific for members of the "*Terrae*" clade and absent in other bacteria.

Group	Species	Accession	153		192
"Terrae" Clade (11/11)	*Mycobacterium algericum*	WP_083040593	PATVLTVLMTSASRMLIIE	H	EALGMTLGHAETVEFVERWL
	Mycobacterium avium	WP_019737406	-----M----G--------	Q	--------------------
	Mycobacterium engbaekii	WP_085129938	-P---M-------------	-	--------------------
	Mycobacterium heraklionense	WP_064889954	-P---M-------------	Q	---------P--I-------
	Mycobacterium hiberniae	WP_085137277	-P---M-------------	-	--------------------
	Mycobacterium kumamotonense	WP_065287159	-----M----G--------	Q	--------------------
	Mycobacterium longobardum	WP_085263276	-P---M-------------	Q	-G----------I-------
	Mycobacterium nonchromogenicum	WP_085137594	-PM--M----------T--	Q	---------------A----
	Mycobacterium senuense	WP_085087222	-----M-------------	-	--------------------
	Mycobacterium sinense	WP_064854393	-----M-------------	Q	--------------------
	Mycobacterium terrae	WP_085261729	----M-------------	Q	-----R--------------
Other *Mycobacterium* (0/82)	*Mycobacterium acapulcensis*	WP_066809934	-PVAVSM-IVQIA-S-CN-		D-V-V----D-MRD-MQ--M
	Mycobacterium alsense	WP_083137863	MT--R-VTGV-A--		-T--V-----K-IQ-A--YI
	Mycobacterium angelicum	WP_083111880	-PV-WA---S-L----VL-		Q-V--SA-----L-L--SY-
	Mycobacterium aromaticivorans	WP_036344143	-PV-WSI-L-GL-TVVMLG		----V-A-----M-L---Y-
	Mycobacterium asiaticum	WP_065037412	-PV-F------L--F-VL-		Q---VSG--P--L----SQ-
	Mycobacterium aurum	WP_048632005	--V-W--FA--V-QA-VM-		R----NT-----FA-C-E-I
	Mycobacterium avium	WP_075362213	-PV-W------L--F-VL-		Q-I--SS------QL--SY-
	Mycobacterium avium subsp. paratuberculosis	ELP47484	-PV-W------L--F-VL-		Q-I--SS------QL--SY-
	Mycobacterium bacteremicum	WP_083057822	-PV-WA-IG--L-WA-VM-		Q----SA-----L-------
	Mycobacterium boenickei	WP_077738776	-PV-MSM-L-QSA-S-CN-		--V-V-D----FRA----F-
	Mycobacterium bohemicum	WP_085182499	-PV-W----S-L--F-VL-		Q-I--SG-----RQL--SH-
	Mycobacterium branderi	WP_083131289	-PL-WS--I--V--V-VM-		Q----SA-----------Y-
	Mycobacterium brisbanense	WP_062832182	-PI-W--FA--V--V-VM-		Q----SS--D-VIA-C-G--
	Mycobacterium celatum	WP_062538577	-PL-WS--I--V--V-VM-		Q----SA------A----Y-
	Mycobacterium celeriflavum	WP_083005970	-PV-W--FA--V-QA-VV-		R-----T-----FA-C-K-I
	Mycobacterium chlorophenolicum	WP_048473397	-PV-W--FA--V-QAMVV-		R---IST-----FA-C-Q--
	Mycobacterium chubuense	WP_014816780	--V-W--FA--V-QA-VV-		R----NT-----F--C----
	Mycobacterium colombiense	WP_064882658	-PV-W----S-L--F-VL-		Q-I--SA-------L--NY-
	Mycobacterium conceptionense	WP_064899576	-PI-W-FIAA-V--VMVM-		Q-----A----VLK-C-D--
	Mycobacterium confluentis	WP_085152730	---M----G-TQV-VQ-		-F-------Q--RA-ADK--
	Mycobacterium cosmeticum	WP_051561044	-PV-W--FA--L--V-VM-		QE---SA-----LD------
	Mycobacterium diernhoferi	WP_079244585	-PV-WS-IG--V-WV-VM-		Q----SA-----L----H--
	Mycobacterium fallax	WP_085093565	---I--L-G-TQV-VQ-		-L-D--V--R--H--AD---
	Mycobacterium farcinogenes	WP_084676714	-PV-MSMIVA-LA-I-VL-		QG--ISR---QAQD--R-Y-
	Mycobacterium flavescens	WP_069414096	-PV-W--FA--V-QS-VV-		R----ST----VFA-C-A-I
	Mycobacterium fortuitum	WP_064847867	-PI-W-FIAA-V--VMVM-		Q-----A----VLK-C-D--
	Mycobacterium fragae	WP_085194701	-PL-WS-----V--V-VM-		Q----SA------A----Y-
	Mycobacterium gastri	WP_036414739	-PV-W------I--F-V--		QG---SG-------V---Y-
	Mycobacterium gilvum	WP_011893398	-PV-W--FA--V-QA-VM-		R----DT-----FA-C-Q-I
	Mycobacterium goodii	WP_049745696	-PV-W-F-A--V--VMVM-		Q----SA----VL--C-S--
	Mycobacterium gordonae	WP_065045183	-PM-W--F---L--FMVL-		Q----SD-----TA----H-
	Mycobacterium haemophilum	WP_054879374	-PV-W---IS-L--L-VL-		Q-V--SA-----FAL--NY-
	Mycobacterium hassiacum	WP_018354796	-PV-W--FA--V-QA-AL-		R---I-S----MFAYC-S-I
	Mycobacterium heckeshornense	WP_048890057	-PL-WS-----I--V-VM-		Q--DVS---P---A----Y-
	Mycobacterium holsaticum	WP_069403720	-PV-W-MFA--V-QA-VV-		Q-----T----ILQ-C-E--
	Mycobacterium houstonense	WP_066898471	-PI-W-FIAA-V--VMVM-		Q-----A----VLK-C-D--
	Mycobacterium insubricum	WP_083030316	---I--L-G-TQV-VQ-		-L-D--V-----Q--AH---
	Mycobacterium intermedium	WP_069420155	-PE-VMT--R-VTGV-A--		-T--V-----K----A--YI
	Mycobacterium iranicum	WP_064281827	-PV-W--FA--V-QA-VM-		R---IST-----FA-C-Q-I
	Mycobacterium kansasii	WP_063472445	-PV-W------I--F-V--		QG---SG-------V---Y-
	Mycobacterium komanii	CRL73201	-PV-W--FA--V-QA-VV-		R-----T-----FA-C-K-I
	Mycobacterium kyorinense	WP_065015660	-PL-WS--I--I----VL-		Q----SA------GL---Y-
	Mycobacterium lacus	WP_085156294	-PV-W--I---L--L-VL-		Q-I--SA-----F-L--SY-
	Mycobacterium mageritense	CDO24041	-PV-W--FA--V--VMVM-		Q----SA----VI--C-G--
	Mycobacterium malmesburyense	CRL74639	-PV-W--FA--V-QA-VV-		R-----T-----FA-C-K-I
	Mycobacterium malmoense	WP_083009230	-PV-W------L--F-VL-		Q----SA-----L-L--SY-
	Mycobacterium mantenii	WP_083093629	-W----S-L--F-VL-		Q-I--SA-----L-L--NY-
	Mycobacterium marinum	WP_012396232	-PV-W----A-L--F-VL-		K----SG-----I-L--SY-
	Mycobacterium monacense	WP_083045528	-PV-W--FA--V-QS-VV-		R-----T-----YA-C-Q-I
	Mycobacterium moriokaense	WP_083153268	-PV-W--FA--V-QG-VM-		R-----T-----FAYC-Q-I
	Mycobacterium nebraskense	WP_046183470	--V-W------L--F-VL-		Q-I--SG-------L--SY-
	Mycobacterium neworleansense	CRZ13343	-PI-W-FIAA-V--VMVM-		Q-----A----VLK-C-D--
	Mycobacterium noviomagense	WP_083087603	-PL-WS--I--V--V-VM-		Q----S-------A----Y-

Other *Mycobacterium* (0/82)	*Mycobacterium novocastrense*	WP_084377005	-PVAVSM-IVQIA-S-CN-	D-V-L----D-MRD-MQ--M
	Mycobacterium paraffinicum	WP_073876618	-PV-W------L----VL-	Q-I--SG-----MAL--SH-
	Mycobacterium parafortuitum	WP_083143955	-PV-W--FA--V-QA-VM-	R----DT-----FA-C-S-I
	Mycobacterium paraseoulense	WP_083171734	-PAGIA-IIAAVG----L-	Q---A-T--S-A-AL-N-FV
	Mycobacterium parmense	WP_085270244	-PV-W------L--F-VL-	Q-I--SG-------L--NY-
	Mycobacterium peregrinum	WP_064886998	-PI-W-FIAA-V--VMVM-	Q-----A----VLQ-C-D--
	Mycobacterium phlei	WP_003890584	-PV-W--FA--V-QA-VV-	R-----T-----Y--C-K-I
	Mycobacterium porcinum	WP_075921032	-PI-W-FIAA-V--VMVM-	Q-----A----VLK-C-D--
	Mycobacterium pseudoshottsii	GAQ32996	-PV-W----A-L--F-VL-	K----SG-----I-L--SY-
	Mycobacterium rhodesiae	WP_014212303	-PV-W--FA-GV-QL-VM-	R---T-F-----FAYC-Q-I
	Mycobacterium riyadhense	WP_085250695	-PV-F----S-L----VL-	Q-V--SA-----L-L--SY-
	Mycobacterium rufum	KGI71022	-PV-W--FA--V-QAMVV-	R---IST-----FA-C-Q--
	Mycobacterium rutilum	WP_083408077	-PV-W--FA--V-QS-VV-	R----ST-----FA-C-A-I
	Mycobacterium saskatchewanense	WP_085255863	-PV-W------L----VL-	Q-I--SA-----M-L--SY-
	Mycobacterium scrofulaceum	WP_067273645	--V-W----S-L----VL-	Q-I--SG-------L--KY-
	Mycobacterium senegalense	KLI07176	-PV-MSMIVA-LA-I-VL-	QG--ISR---QAQD--R-Y-
	Mycobacterium septicum	WP_044522392	-PI-W-FIAA-V--VMVM-	Q-----A----VLK-C-D--
	Mycobacterium setense	WP_039322577	-PI-W-FIAA-V--VMVM-	Q-----A----VM--C-G--
	Mycobacterium sherrisii	WP_069398371	-PV-F----S-L--F-VL-	Q-V-ISA-----RAL--SY-
	Mycobacterium shimoidei	WP_069396775	-PL-WS--I--V--V-VM-	Q----SA------Q----Y-
	Mycobacterium smegmatis	WP_003896181	-PI-W-F-A--V--VMVM-	Q----SA----VL--C-G--
	Mycobacterium szulgai	ORX18976	-PV-F----S-L----VL-	Q----SA-----L-L--NY-
	Mycobacterium thermoresistibile	WP_003927047	-PV-WA-FAAAV----VM-	K-I--SV--K-IL--C-D--
	Mycobacterium tusciae	WP_083125750	-PV-W--FA--V-QG-VM-	R-----T-----FAYC-Q-I
	Mycobacterium ulcerans	EUA92957	-PV-W----A-L--F-VL-	K----SG-----I-L--SY-
	Mycobacterium vaccae	WP_003928279	-PV-W--FA--V-QA-VM-	R----DT-----FA-C-G-I
	Mycobacterium vulneris	WP_085292490	-PV-W----S-L--F-VL-	Q-I--SA-------L--NY-
	Mycobacterium wolinskyi	WP_067847646	-PI-W-IFA--V--VMVM-	Q----SA----VL--C-S--
	Mycobacterium xenopi	WP_085195943	-PVAVAF-L-GVQGV-FM-	----ISA--T-V-AL---Y-

Supplementary Figure 65

A partial sequence alignment of a conserved region of TetR/AcrR family transcriptional regulator showing a one amino acid insertion that is specific for members of the "*Terrae"* clade and absent in other *Mycobacterium*.

Group	Species	Accession	157		199
**"Terrae" Clade (10/10)**	*Mycobacterium engbaekii*	WP_085129961	SVHIRGSVVLRRLYEKNRASDA	E	GPSDFEEALVIDPTVTPLLA
	Mycobacterium heraklionense	WP_064889210	----------Q-----------	-	-------------A------
	Mycobacterium hiberniae	WP_085137314	----------------------	-	-------------A------
	Mycobacterium icosiumassiliensis	WP_067969714	---T------Q-----T-L---	R	-T---D--P----VA-----
	Mycobacterium kumamotonense	WP_083082992	----------------------	-	-------------Q------
	Mycobacterium longobardum	WP_085263170	----------------------	-	-------------A------
	Mycobacterium nonchromogenicum	WP_085137574	----------Q--S--------	-	-----Q--V----A------
	Mycobacterium senuense	WP_085087291	-L--------------------	D	-------------EL-----
	Mycobacterium sinense	WP_064853223	----------------------	-	-------------E------
	Mycobacterium terrae	WP_085261757	----------------------	-	-------------E------
**Other *Mycobacterium* (3/>100)**	*Mycobacterium cosmeticum*	WP_051562165	TL-V------Q--A---HQA-G	D	---APDRTF----E-A----
	Mycobacterium heckeshornense	WP_048893560	---V------H--HQ--QEAEN	G	AARTI-D-M----ET-----
	Mycobacterium xenopi	WP_085195463	---V------H--HQ--QEAEN	G	SARTI-D-M----ET-----
	Mycobacterium alsense	WP_083137059	-L-V------H------QTPES		--RAI-D-VA---AT---I-
	Mycobacterium aromaticivorans	WP_036341476	---V------H--------AGE		-AHQL-D-IIGTAES-----
	Mycobacterium arosiense	WP_083062646	---V------Q------QS--I		--RAI-D-VA---EK-----
	Mycobacterium asiaticum	WP_065038563	-L-V------H--N---EL--D		-NRTI-D-VA---GT---I-
	Mycobacterium aurum	WP_048634603	---V---------R---L-N-D		--G-I-DTMA---ETA----
	Mycobacterium avium	WP_031353960	---V------Q------QS--V		--RAI-D-VA---EK-----
	Mycobacterium avium subsp. hominissuis	ETB30902	---V------Q------QS--V		--RAI-D-VA---EK-----
	Mycobacterium avium subsp. paratuberculosis	AAS04711	---V------Q------QS--V		--RAI-D-VA---EK-----
	Mycobacterium bacteremicum	WP_083056956	TL-V------Q--A---QTDE-		--GA-DQT-----D-A----
	Mycobacterium boenickei	WP_077743899	---V------H--------N-N		A-G-Y--TM----EI-----
	Mycobacterium bohemicum	WP_085183273	-L-V------H--S---QSA-S		--RAIDD-VS---AT---IS
	Mycobacterium bovis	WP_050895880	---V------D--SR-SQSAGS		---AI-HPVA---AT-----
	Mycobacterium branderi	WP_083133682	---V------H--HQ--Q-TEN		-GRTI-D-M---AET-----
	Mycobacterium brisbanense	WP_062829034	---V------H---T----D-N		A-G-Y--TM----ES-----
	Mycobacterium canettii	WP_015289790	---V------D--SR-SQPA-S		---AI-HPVA---AT-----
	Mycobacterium celatum	WP_062539798	---V------H--HQ--Q-TEN		-GRTI-D-M---AET-----
	Mycobacterium celeriflavum	WP_083002180	---V------H--R-----TAE		----I--TMT--AEN-----
	Mycobacterium chimaera	WP_054585558	---V------Q------QS--I		--RAI-D-VA---EK----S
	Mycobacterium chubuense	WP_014815005	---V------Q--R---L-GED		-HG-IDDTMT---ETA----
	Mycobacterium colombiense	WP_064884801	---V------Q------QS--V		--RAI-D-VT---EK-----
	Mycobacterium conceptionense	CQD12503	---V------H--------N-N		A-G-Y--TM----E------
	Mycobacterium confluentis	WP_085153462	---V------Q------A-AED		-GVAL-DN--V--EN-----
	Mycobacterium conspicuum	WP_085233187	-L-V------Q------QS--S		--RSI-D-VGV-LQG-----
	Mycobacterium diernhoferi	WP_073855826	TL-V------Q--A---Q-G-E		--GAYDQT-----E-A----
	Mycobacterium europaeum	WP_085239177	---V------Q------QS--V		-ARAI---VA---EK-----
	Mycobacterium farcinogenes	WP_036389955	---V------H--------N-N		A-G-Y--TM----E------
	Mycobacterium flavescens	WP_069411839	---V------H--R----MATE		----I--TMS--ADS-----
	Mycobacterium florentinum	WP_085220877	---V------Q------QS--V		--RAI-D-VA---EK-----
	Mycobacterium fortuitum	WP_065070866	---V------H--------N-N		A-G-Y--TM----E------
	Mycobacterium fragae	WP_085195309	---V------H--QQ--Q--EN		-ARTI-D-M----QTA----
	Mycobacterium gastri	WP_036416637	-L-V------H--N--HQSA-S		--RAL-D-ME---QT---I-
	Mycobacterium genavense	WP_036466829	---V------Q------QS--V		--RTI-D-VA---DK-----
	Mycobacterium gilvum	WP_011895199	---V------Q--R---L-G-D		-HG-IDDTMA---ETA----
	Mycobacterium goodii	WP_083453206	---V------H--------N-N		A--L--D-M--N-ET-----
	Mycobacterium gordonae	WP_065044909	-L-V------H--H---QS-ED		-TRSI---VAV--QT---I-
	Mycobacterium haemophilum	WP_047315805	---V------H------QSP-S		-SRAI-D-VA---AT---I-
	Mycobacterium hassiacum	WP_005626226	-L-V------Q--R---E-ANE		--R-L-DTMN---ET-----
	Mycobacterium heidelbergense	WP_083073443	-M-V------Q-------S--S		-GRAI-D-VA---QT---I-
	Mycobacterium holsaticum	WP_069407672	---V------Q--RD----ANE		----I-DTMS--AES-----
	Mycobacterium houstonense	WP_066903001	---V------H--------N-N		A-G-Y--TM----D------
	Mycobacterium insubricum	WP_083030415	-L-V------H-------EI-G		--G-----WT-E-SRA-V--
	Mycobacterium interjectum	WP_066914500	-L-V------Q---D--QTP-S		--RAI-D-VA---AT---I-
	Mycobacterium intermedium	WP_069418749	---V------H--S---KSA-S		--R-I-D-MA---QT---I-
	Mycobacterium intracellulare	WP_064935747	---V------Q------QS--I		--RAI-D-VA---EK----S
	Mycobacterium iranicum	WP_064280726	---V------Q--RD--L-GED		-HG-IDDTMT---ETA----
	Mycobacterium kansasii	KZS58020	-L-V------H--N--HQSA-S		--RAL-D-ME---QT---I-
	Mycobacterium komanii	CRL66523	---V------H--R-----A-E		----L--SMD--ADS-----
	Mycobacterium kubicae	WP_085074825	---V------H------LSA-S		E-RGI-D-MA---QT---I-
	Mycobacterium kyorinense	WP_065012666	---V------H--H---A-TEN		-ARTI-DSMI--AET-----
	Mycobacterium lacus	WP_085158707	---V------H------QS--N		--RAI-D-VA---QT---I-
	Mycobacterium lentiflavum	CQD18761	---V------Q------QS--V		--RAI-D-VA---EK-----

Other *Mycobacterium* (3/>100)	*Mycobacterium liflandii*	WP_015356647	-I-V------H-----TQSA-S	--RAI-D-MN---LT-----
	Mycobacterium mageritense	WP_036427874	---V------H--------I-N	A-G-Y--TM----ET---I-
	Mycobacterium malmesburyense	CRL70407	---V------H--R-----VNE	----L--SMS--ADS-----
	Mycobacterium malmoense	WP_065471757	---V------Q--R---Q-AAD	----I--TMT-ESEM-----
	Mycobacterium mantenii	WP_083094265	---V------Q------QS--V	--RAI-DVVS---EK-----
	Mycobacterium marinum	WP_012395610	-I-V------H-----TQSA-S	--RAI-D-MN---LT-----
	Mycobacterium moriokaense	WP_083150443	---V------E--R-----ANE	----ID--MA--SES-----
	Mycobacterium nebraskense	WP_046181879	---V------Q------QS--E	-ARAI-D-VA---EK-----
	Mycobacterium neworleansense	CRZ15359	---V------H--------N-N	A-G-Y--TM----ET-----
	Mycobacterium noviomagense	WP_083084549	---V------H--HQ---E-EN	-ARTI-D-M-M--ET-----
	Mycobacterium novocastrense	WP_067395076	---V------H--R-----A-E	----L--TMS--ADS-----
	Mycobacterium orygis	WP_003406969	---V------D--SR-SQSAGS	---AI-HPVA---AT-----
	Mycobacterium palustre	WP_085077054	---V------H------Q---S	-SRAI-A-VA----T---I-
	Mycobacterium paraense	WP_085094136	-L-V------Q---D--QTP-S	--RAI-D-VA---AT---I-
	Mycobacterium paraffinicum	WP_073875182	---V------Q------QS-GE	-ARAI-D-VA---EK-----
	Mycobacterium parafortuitum	WP_083141672	---V------Q--R---L-GED	-HG-IDDTMAF--ETA----
	Mycobacterium paraseoulense	WP_083170282	---V------Q------QS--E	-ARAI-D-VA---EK-----
	Mycobacterium parmense	WP_085268913	---V------Q------QS--V	--RAI-D-VA---EM-----
	Mycobacterium peregrinum	WP_064886212	---V------H--------N-N	A-G-Y--TM----E------
	Mycobacterium phlei	WP_040633749	---V------H--R-----AGE	----LDDTMSV-AES-----
	Mycobacterium porcinum	WP_075919694	---V------H--------N-N	A-G-Y--TMT---E------
	Mycobacterium rhodesiae	WP_083118578	---V------H--------AGE	DAHQL-D-IIGTAES-----
	Mycobacterium riyadhense	WP_085251702	-L-V------Q--F---QSA-G	A-RTI-D-VS---HS---I-
	Mycobacterium rutilum	WP_083409672	---V------H--R-----AGD	----L--TMN--ADS-----
	Mycobacterium saskatchewanense	WP_085256148	-L-V------H--S---QSP-S	ESRT--DTMA---GS-----
	Mycobacterium scrofulaceum	WP_067281029	---V------Q------QST-E	-ARAI-D-VA---EK-----
	Mycobacterium septicum	WP_044519357	---V------H-------SN-N	A-G-Y--TM----E------
	Mycobacterium setense	WP_039318610	---V------H--------N-N	A-G-Y---M----D------
	Mycobacterium sherrisii	WP_069398774	---V------Q------QS---	-SRAI-D-VS---EK-----
	Mycobacterium shimoidei	WP_069394400	---V------H--Q---K-T-N	-ARTI-D-M---SES-----
	Mycobacterium simiae	WP_061559764	---V------Q------QS--V	-SRAI-D-VS---EK-----
	Mycobacterium smegmatis	WP_003893597	---V------H--------N-N	A-G-----M----ET-----
	Mycobacterium szulgai	WP_068032779	---V------H------LSG-S	E-RGI-D-MA---QT---I-
	Mycobacterium triplex	WP_036470612	---V------Q------QS--V	--RAI-D-VA---DK-----
	Mycobacterium tuberculosis	WP_070894336	---V------D--SR-SQSAGS	---AI-HPVA---AT-----
	Mycobacterium tusciae	WP_083126180	---V------Q--R-----A-D	--A-ID--MA--SES-----
	Mycobacterium ulcerans	WP_011741566	-I-V------H-----TQSA-S	--RAI-D-MN---LT-----
	Mycobacterium vaccae	WP_003928813	---V------Q--R---L-GED	SHG-IDDTMT---ATA----
	Mycobacterium vulneris	WP_085289134	---V------Q------QS--V	--RAI-D-VS---EK-----
	Mycobacterium wolinskyi	WP_067857714	---V------H--------N-N	A-G-Y--TMI---E------
	Mycobacterium yongonense	WP_020822026	---V------Q------QS--I	--RAI-D-VA---EK----S
Other bacteria	*Gordonia amarae*	WP_005190031	L-----I--E---QRSK-E-S	-NVAYY-N---T-EA-----
	Nocardia alba	WP_067446933	NL-VQ-----Q--HDR-QELGS	DATAYYDVIHV-AET-----
	Nocardia carnea	WP_081596016	NL--------Q---D--SEQGD	SARAYY-EIQV--EK----S
	Nocardia flavorosea	WP_062978304	NL--------Q---D--SELGD	SARAYY-EIR---EK----S
	Nocardia gamkensis	WP_062970066	NL--------Q---D--KEQ--	EGGAYYDDIL-E-ET-----
	Nocardia jiangxiensis	WP_083895619	NL--------Q---D--QDGEG	SSRAYYDSI-V--QT-----
	Nocardia miyunensis	WP_067676886	LLV------Q---D--KELYN	EARPYY-S-IFE-ET-----
	Rhodococcus kunmingensis	WP_068274025	L-V------T--HD--K-A-P	ESRAYY-N-I-T-EA-----
	Rhodococcus phenolicus	WP_068166779	L-VH-MI--H--Q----Q-E-	PSGAYY-LFP-SSET-----
	Skermania piniformis	WP_083530052	NL-V------Q---D--KEIE-	-A-AYYDDIT-E-ET-----
	Stenotrophomonas maltophilia	WP_081280515	---V------H------QSP-S	-SRAI-D-VA---AT---I-

Supplementary Figure 66

A partial sequence alignment of a conserved region of TetR family transcriptional regulator showing a one amino acid insertion that is specific for members of the "*Terrae*" clade and absent in most other bacteria.

Group	Species	Accession	47		92
**“*Terrae*” Clade (8/8)**	*Mycobacterium algericum*	WP_083037591	MPAGRRHVRLLIAGTDADIIDVGM		RLCAKAFGTEPVPGVVTYVSR
	Mycobacterium engbaekii	ORV41118	------------S-SE--VREL--		----S----T-E------L--
	Mycobacterium heraklionense	WP_064889908	--V-----------AEE-VV-I--		-------D-M-T------L--
	Mycobacterium hiberniae	ORV69642	--V---------T-SES-VREL--		----S--N-TAE------L--
	Mycobacterium kumamotonense	WP_065288535	--V---Q-----T-AETE---T-T		-------D-T-----------
	Mycobacterium longobardum	ORW13940	--V---QA----T-AE--VV-I-I		----N----M-AA-----L--
	Mycobacterium senuense	WP_085084159	--V---Q-----T-P-IVDTGIQL		-------N-AA--------
	Mycobacterium sinense	OBF97410	--V---Q-----S-A-I--V-I-T		----S----A-T---------
	Mycobacterium terrae	WP_085261683	--V---QI----T-AEK---AT-T		Q------N-K-M---L-----
**Other *Mycobacterium* (0/96)**	*Mycobacterium acapulcensis*	WP_066808239	L-PK--E-E---V-D-Q-VLRRLA	V	E--GN----T-------FI--
	Mycobacterium alsense	WP_083136636	-----E-E---PAE-GEAARKVA	I	E---RV--SAA------FI--
	Mycobacterium angelicum	WP_083111848	-ELE---ETD-VESAKRTA	I	E--GR--N-S--A----F---
	Mycobacterium aromaticivorans	WP_036344100	--R---VAE---GN-AV-DLPRIG	V	T--V--------V--------
	Mycobacterium arosiense	WP_083063645	L--S--E-E--V-AE-G-TAKATA	I	G---S---VD--A--I-F---
	Mycobacterium asiaticum	WP_065036416	-R-G-V-EV---DS-VESAKTTA	I	GV-G-----S-AA----F---
	Mycobacterium aurum	WP_048631975	--RN--Q-E-----D---ELRRVS	V	E--T----GFG-QPCAGVLTY
	Mycobacterium austroafricanum	WP_036371652	L-PK--E-EI-M-----AALQSRA	A	I------R----I--L-FI-H
	Mycobacterium avium	WP_023866502	---S--E-E----AD-G-AARDVA	V	T------QST-------FI--
	Mycobacterium avium subsp. avium	EUA39923	---S--E-E----AD-G-AAMGVA	V	T------QST-------FI--
	Mycobacterium avium subsp. hominissuis	KD097706	---S--E-E----AD-G-AARDVA	V	T------QST-------FI--
	Mycobacterium avium subsp. paratuberculosis	ETB02124	--E-E----ADHG-AARDVA	V	T------QST-------FI--
	Mycobacterium bacteremicum	WP_083057769	-EAEI-ME----AVLQERA	L	SM--V----D--V--L-F--H
	Mycobacterium boenickei	WP_077741520	L--H--E-EV----D--PELQAIA	M	E--------T-TA----F---
	Mycobacterium bohemicum	WP_085182529	--V---E-E----RDNV-ALKTLG	V	EI-D----AFGPRP-AGV-TF
	Mycobacterium branderi	WP_083131451	L-VS--E-Q-----S---AVRQTA	V	E--------V-A---------
	Mycobacterium brisbanense	WP_062832158	LS-H--E-EV----D--QLLQQLV	I	E--------D-AA----F---
	Mycobacterium celatum	WP_062540025	L-VS--E-Q----AS---VVKETA	V	E--------T-AA--------
	Mycobacterium celeriflavum	WP_083006850	L--Q--E-E---E-D-P-TLTRTA	L	D---R---AFGTTP-PGV-TF
	Mycobacterium chlorophenolicum	WP_048473266	L-PK--E-EI-MT----AALQATG	I	A---R-------A--L-F--H
	Mycobacterium chubuense	WP_014816812	L-PK--E-EI-MT--EPSALQNAA	V	E--RR--P-Q--I--L-FI-H
	Mycobacterium colombiense	WP_044487313	---S--E-E----AE-GATARDTA	I	G---S---GA-----I-F---
	Mycobacterium conceptionense	WP_064894139	L--H--E-EV----D--PELQTVA	M	E--------S-TA----F---
	Mycobacterium confluentis	WP_085152898	L-PS--E-EF-MT-A-PAELQDVA	N	G--GR-------A--L-F--H
	Mycobacterium conspicuum	WP_085234843	---E-EV---AD---AAKRTA	I	D------S-T-------F---
	Mycobacterium cosmeticum	CDO06120	L-----QIEV-LT-VH-EVLTDEA	V	T--S-----R--A--L-F---
	Mycobacterium diernhoferi	WP_073853208	--SN--E-EV-L--D-GETLRAAV	V	T--G----DDVL---L-F---
	Mycobacterium europaeum	WP_085242392	--L---E-E----AEHG-EAIEAA	I	G--TTV--GS--A----FI--
	Mycobacterium fallax	WP_085093449	--PS--EIE--MT-A-P-QLRDIA	I	GM--T-----------F--H
	Mycobacterium farcinogenes	WP_036393254	L--H--E-EV----D--PELQTVA	M	E--------S-TA----F---
	Mycobacterium flavescens	WP_069414063	L-PS--E-E-----D-PEELQRIA	L	E--GN----T-----L-FI--
	Mycobacterium florentinum	WP_085223791	--IS--E-EV-V-AE-G-TAKETA	I	G--TMV-SVT-----I-F---
	Mycobacterium fortuitum	WP_064847877	L--H--E-EV----D--PELQSIV	M	E--------A-TA----F---
	Mycobacterium fragae	WP_085194641	L--D--E-Q-----S-S-AVKQVA	V	D--------A--A--------
	Mycobacterium gastri	WP_036417634	-S---ELE---TCDGVEAAKQTA	I	D------D-T--A----FI--
	Mycobacterium gilvum	WP_011893366	L-PK--E-EI-MT---PAALQTQA	A	E---R--P----I--L-F--H
	Mycobacterium goodii	WP_049745676	L--H--E-EV----S--QQLQESV	L	Q--------T-TA----F---
	Mycobacterium gordonae	WP_065045214	-PH--E-E-----E-VEVTK-TA	R	D--R----A-A-V----FI--
	Mycobacterium hassiacum	WP_005623666	L-PA--E-E--MT-D--GGLQSTA	L	A--RD------------FI--
	Mycobacterium heckeshornense	WP_048890090	-VN--E-Q---S-N---SVRQTA	I	G--SN----N--A--I-----
	Mycobacterium heidelbergense	WP_083072145	--R--E-E-----D--ESVKSAG	V	D---R----Q--A--I-FI--
	Mycobacterium holsaticum	WP_069407457	L-PK--E-E---D-E-P-ELQRCA	V	D---G----T-----L--I--
	Mycobacterium houstonense	WP_066898505	L--H--E-EV----EN-PELQAIV	M	E--------S-AA----F---
	Mycobacterium insubricum	WP_083032317	--PS--E-E--MT-A---ALQATA	M	AM--NV---D--A--L-F--H
	Mycobacterium interjectum	WP_066915749	------E-E----AE-G-AASNTA	I	E--T-I---G-------FI--
	Mycobacterium intermedium	WP_069418092	-D-E--VT-D-VESAKKTA	I	E---N--N-S--A----F---
	Mycobacterium intracellulare	WP_064933391	--VR--E-E----AE-SAAAQNTA	I	GV-------T-R---I-F---
	Mycobacterium iranicum	WP_064281796	L-PR--E-E--MR-L-PAALQSTA	A	A---R--P----L--L-F--H
	Mycobacterium kansasii	WP_075512053	-S---ELE---TCDGVEAAKQTA	I	D------D-T--A----FI--
	Mycobacterium komanii	CRL71088	---Q--E-E-----D-P-ELRRIA	I	E--GN----T-------FI--
	Mycobacterium kyorinense	WP_065015634	--I---E-Q---G-N-VETLKRTA	V	E--R-----D--A--------
	Mycobacterium lacus	WP_085155937	-D-Q----SD-VEVAKRAA	I	D--S-V-N-G--A-A--FI--
	Mycobacterium lentiflavum	CQD20739	--ES--E-E----AENG-LAKESA	I	E----V-SNT-----I-F---
	Mycobacterium mageritense	WP_036436219	L--S--EIEV----EG-PELQQLV	M	Q--------S-TA----F---
	Mycobacterium malmesburyense	CRL75622	L-PK--E-E-----D-P-VLHRQA	I	E--GN----T-------FI--

Group	Species	Accession	Sequence		
	Mycobacterium malmoense	WP_065446163	------E-E--V-AQ-G-TAKGTA	M	E----V--GT-------FI--
	Mycobacterium mantenii	WP_083093707	---S--E-E----AE-G-TAKGTA	I	G---R--DAT-I---I-F---
	Mycobacterium marinum	WP_020729527	-V---E-E-----D-V-EAKAAA	I	E---S--MVR--V----FI--
	Mycobacterium marseillense	WP_083015030	--VR--E-E----AE-GGAAQTAA	I	DV-------V-R---I-F---
	Mycobacterium moriokaense	WP_083153213	L--H--E-E-----D--AELERTA	I	A--------T-----L-FI--
	Mycobacterium nebraskense	WP_046181756	--VA--EIE---TAE-G-AARNAA	I	E---RV-SAK-------FI--
	Mycobacterium neworleansense	CRZ13301	L--Q--E-EV----D--PELQAIA	M	G--------V-TA----F---
	Mycobacterium noviomagense	WP_083087442	--E-Q-----D--HTLKQTA	V	DM-------A-AA--------
	Mycobacterium palustre	WP_085076407	--PS--A-EV----D---AVKQVG	I	T---Q----A--A----F---
	Mycobacterium paraense	WP_085093255	---A--E-E----AE-G-AASNAA	I	E--------R-------FI--
	Mycobacterium paraffinicum	WP_073876675	------E-E----AE-G-TAKRVA	I	S----V--GT-------FI--
	Mycobacterium parafortuitum	WP_083143928	L-PK--E-EI-MR----AALQAEA	A	GM-----P----I--L-F--H
	Mycobacterium parascrofulaceum	EFG75519	------E-E--V-AQ-G-TAKGTA	M	E----V--GT-------FI--
	Mycobacterium paraseoulense	WP_083168510	--V---E-EV---AEHG-EAKKAA	I	E---NV--GS-------FI--
	Mycobacterium parmense	WP_085270205	------E-E--V-AE-G-AARRTA	I	E--T-V--AG-------FI--
	Mycobacterium peregrinum	WP_064884087	L--H--E-EV----N--PELQAIA	M	E--------S-AA----F---
	Mycobacterium phlei	WP_081491194	L-PQ--E-E-----E-P-ELQRSA	V	E--T----GYG-TP-PGVLTF
	Mycobacterium porcinum	WP_069428229	L--H--E-EV----D--PELQAIA	M	E--------A-TA----F---
	Mycobacterium rhodesiae	WP_083122125	--R---EAE---GNASPADLTTVG	V	T-----------I--------
	Mycobacterium riyadhense	WP_085250663	-D-Q----CD-VAAAKQAA	I	D--S---N-A--A----F---
**Other *Mycobacterium* (0/96)**	*Mycobacterium rufum*	KGI69148	L-PQ--E-EI-MV-D--ADLQ-AA	I	A---R-------A----F--H
	Mycobacterium rutilum	WP_083408046	L-PQ--E-E---T-D-PAELQRTA	M	Q---N----T-----L-FI--
	Mycobacterium saskatchewanense	WP_085255835	--VD--E-E---T-N-V-GLKALG	I	D--NR---PGALA-----I--
	Mycobacterium scrofulaceum	WP_067273755	------E-E---VAE-G-AAKQRA	M	N----V--DIA------FI--
	Mycobacterium septicum	WP_044522334	L--N--E-EV----N--PELQAIA	M	E--------A-TA----F---
	Mycobacterium setense	WP_039322662	L--H--E-EV----N--PELQTIA	M	Q--------S-T-----F---
	Mycobacterium sherrisii	WP_069398343	-VS--E-A---TAE-P-TAKATA	M	S---SV--ID-------F---
	Mycobacterium shimoidei	WP_069398160	---A--E-----N-D---VVRQSA	V	D--------D--A--------
	Mycobacterium simiae	WP_061557090	-VS--E-E---TAE-GGTAEAAA	I	T---SV---N-------F---
	Mycobacterium smegmatis	WP_011730103	L--H--E-EV----S--EQLQQMV	L	P--------S-TA----F---
	Mycobacterium szulgai	ORX19006	-ELE---ETD-VESAKRAA	I	E--G---N-S-LT----F---
	Mycobacterium thermoresistibile	WP_003927075	---R--E-E--MT-S-PEELQRTA	V	T----V---N-----L--I--
	Mycobacterium triplex	WP_036471497	--ES--E-E---TADHS-TAKEAA	I	G--T----VT-----I-F---
	Mycobacterium tusciae	WP_083125720	L-PQ--E-E-----E--AELERIA	V	S--------T-----L-FI--
	Mycobacterium ulcerans	BAV40040	-V---E-E-----D-V-EAKAAA	I	E---G--RVR--V----FI--
	Mycobacterium vaccae	WP_003928248	L-PT--E-EI-MR-S-PQALQDKA	I	S---G--A-D-----L-F--H
	Mycobacterium vanbaalenii	WP_011781323	L-PK--E-EI-M----PTMLQTRA	A	S------R----I--L-FI-H
	Mycobacterium vulneris	WP_065460230	L--H--E-EV----D--PELQAIA	M	E--------A-TA----F---
	Mycobacterium wolinskyi	WP_067847720	L--H--E-EV----D--RQLQTVA	I	D--------T-AA----F---
	Mycobacterium xenopi	WP_003922634	-VS--E-Q---S-NE--SVKQTA	I	D--SN----S-AA--I-----
	Mycobacterium yongonense	WP_065503375	--VR--E-E----AE-SGAAQNTA	I	G--------T-R---I-F---
	Cryptosporangium aurantiacum	WP_073252043	N-PELQF-M--D--AALRETA	A	A---R---V--AA-A---L--
	Microbacterium ketosireducens	KJL44021	DDA	V	SF-QQV-DAPAAL--T-F---
	Nocardia abscessus	WP_043701629	--A--E-Q--V-TD-P-AAVREA	V	-V--A----T-AA----F---
	Nocardia aobensis	WP_036508584	L--R--EIQV--D-D--AASREHA	V	GM--DI--PAVR--A--F---
**Other bacteria**	*Nocardia carnea*	WP_033246801	V-RS--V-QV-LDTA--AADTARA	V	EI--RV------A--------
	Nocardia cerradoensis	WP_039782431	L--R--EIQV--D-D-TAALLEHA	V	GM--DI--SAVR--A--F---
	Nocardia elegans	WP_063031941	L--R--EIQV--D-D-TAALREHA	V	GM--DI--SAVR--A--F---
	Nocardia exalbida	WP_040864066	---G-E----VETD-P-AAMGEA	V	----A----A--A----F---
	Nocardia mikamii	WP_062996099	L--R--EIQ---D-A-TAAMREHA	V	GI--EI--SAVR--P--F---
	Rhodococcus yunnanensis	WP_072801665	L-PRG-EIE-IVSD--LAERAQ-Y	A	L--ET---RPAYL--I--I--

Supplementary Figure 67

A partial sequence alignment of a conserved region of a hypothetical protein showing a one amino acid deletion that is specific for members of the "*Terrae*" clade and absent in other bacteria.

	Species	Accession	4		32
**"*Terrae*" Clade (9/9)**	*Mycobacterium algericum*	WP_083037811	IDCLVNVHFGESE		QPAWMRKTRDEYFKGP
	Mycobacterium engbaekii	WP_085129967	-----------NR		--E--VRA--D-----
	Mycobacterium heraklionense	WP_064888731	-----------N-		--S--VR---D-----
	Mycobacterium hiberniae	WP_085137325	-------------		--G--VRA--D-----
	Mycobacterium kumamotonense	WP_065287179	-----------R-		-----T----D-----
	Mycobacterium longobardum	WP_085263163	-----------N-		-----VR---D-----
	Mycobacterium senuense	WP_085087307	-----------N-		--S--VR---D-----
	Mycobacterium sinense	WP_064853230	-----------R-		-----A----D-----
	Mycobacterium terrae	WP_085261763	-------------		----------------
**Other *Mycobacterium* (0/91)**	*Mycobacterium alsense*	WP_083137065	-----------T-	K	--T--L-V--D-----
	Mycobacterium angelicum	WP_083113471	-----------T-	A	--EF-T-V--D-----
	Mycobacterium aromaticivorans	WP_036341464	----A------T-	N	--TF-K-V--------
	Mycobacterium arosiense	WP_083062652	-----------T-	Q	--T--L-V--------
	Mycobacterium asiaticum	WP_065033596	-----------T-	A	--EF-KRV--D-----
	Mycobacterium aurum	WP_048634607	-----------TA	R	--EF-L-V--D-----
	Mycobacterium austroafricanum	WP_036373730	-----------TA	K	--EF-L-V--D-----
	Mycobacterium avium	WP_065370725	-----------T-	K	--T--L-V--D-----
	Mycobacterium avium subsp. avium	EUA37421	M----T-	K	--T--L-V--D-----
	Mycobacterium avium subsp. paratuberculosis	ELP47312	M----T-	K	--T--L-V--D-----
	Mycobacterium bacteremicum	WP_083056944	----A------TA	Q	--TF-T-V--D-----
	Mycobacterium boenickei	WP_077743777	----A------T-	N	--TF-K-V--D-----
	Mycobacterium bohemicum	WP_085183156	-----------IG	Q	--T--L-V--D-----
	Mycobacterium branderi	WP_083133676	-------------	K	--S--L-V--D-----
	Mycobacterium celatum	WP_062539792	-------------	K	--S--L-V--D-----
	Mycobacterium chubuense	WP_014815012	-----------TA	Q	--EF-L-V--D-----
	Mycobacterium colombiense	WP_064884789	-----------T-	Q	--T--L-V--D-----
	Mycobacterium conceptionense	WP_085141087	----A------T-	N	--TF-K-V--D-----
	Mycobacterium confluentis	WP_085153450	-----------T-	V	--QF-T-V--D-----
	Mycobacterium conspicuum	ORV41815	-----------TQ	V	--EF-TRV-ED-----
	Mycobacterium diernhoferi	WP_073856173	----A------T-	N	--TF-K-V--D-----
	Mycobacterium europaeum	WP_085239171	-----------A-	S	--T--L-V--D-----
	Mycobacterium fallax	WP_085099610	-----------TA	V	--QF-T-V--D-----
	Mycobacterium farcinogenes	WP_036388464	----A------T-	N	--TF-K-V--D-----
	Mycobacterium flavescens	WP_069411833	-----------TA	K	--EF-L-V--D----
	Mycobacterium florentinum	WP_085220873	----I------VD	S	--T--L-V--D-----
	Mycobacterium fortuitum	WP_061264463	----A------T-	N	---F-K-V--D-----
	Mycobacterium fragae	WP_085195317	-----------A-	S	--T--L-V--D-----
	Mycobacterium gastri	WP_036418739	-----------AG	S	--T--L-V--D-----
	Mycobacterium gilvum	WP_011895192	-----------TA	N	--EF-L-V--D-----
	Mycobacterium gordonae	WP_065044919	-----------T-	T	--EF-T-V--D-----
	Mycobacterium hassiacum	WP_005626233	-----------T-	Q	--S--V-V--D-----
	Mycobacterium heckeshornense	WP_048893556	-----------A-	K	--K-LL-V--D-----
	Mycobacterium heidelbergense	WP_083073431	-----------T-	V	--EF-T-V--D-----
	Mycobacterium holsaticum	WP_069407665	-----------TQ	Q	--T--L-V--D-----
	Mycobacterium houstonense	WP_066902989	---------S-T-	N	--TF-T-V--D-----
	Mycobacterium insubricum	WP_083030411	-----------TA	V	--QF-T-V--D-----
	Mycobacterium interjectum	WP_066914494	----I------AD	S	--T--L-V--D-----
	Mycobacterium intermedium	WP_069417252	-----------T-	K	--EF-TRV--D-----
	Mycobacterium intracellulare	WP_064935733	-----------T-	K	--T--L-V--D-----
	Mycobacterium iranicum	WP_064280732	-----------TA	S	--EF-L-V--D-----
	Mycobacterium kansasii	WP_063467270	-----------AA	S	--T--L-V--D---S-
	Mycobacterium komanii	CRL66517	-----------T-	Q	--T--L-V--D-----
	Mycobacterium kubicae	WP_085074887	-----------TD	V	--T----VK-D-----
	Mycobacterium kyorinense	WP_065014362	-----------T-	K	--T--L-V--D-----
	Mycobacterium lacus	WP_085158580	-----------A-	A	--S--L-V--D-----
	Mycobacterium lentiflavum	CQD18737	-----------VD	S	--T--L-V--D-----
	Mycobacterium liflandii	WP_015356642	-----------A-	A	--N--L-V--D-----
	Mycobacterium mageritense	WP_036427862	-----------T-	N	--TF-K-V--D-----
	Mycobacterium malmesburyense	CRL70389	-----------T-	Q	--T--L-V--D-----
	Mycobacterium malmoense	WP_065441643	-----------A-	S	-----LRV--D-----
	Mycobacterium mantenii	WP_083094258	-----------T-	Q	--T--I-V--D-----
	Mycobacterium marinum	WP_012395604	-----------A-	A	--N--L-V--D-----
	Mycobacterium moriokaense	WP_083150354	-----------T-	K	--EF-L-V--D-----
	Mycobacterium nebraskense	WP_046181885	-----------T-	K	--T--L-V--D-----

Other *Mycobacterium* (0/91)

Mycobacterium neworleansense	CRZ15352	----A------T- N --TF-K-V--D-----
Mycobacterium noviomagense	WP_083084103	------------- K --S--L-V--D-----
Mycobacterium novocastrense	WP_067395060	-----------T- Q --T--L-V--D-----
Mycobacterium palustre	WP_085077060	-----------TD S --G--L-V--D-----
Mycobacterium paraense	WP_085094148	----I------AD S --T--L-V--D-----
Mycobacterium paraffinicum	WP_073875194	-----------A- S --T--L-V--D----
Mycobacterium parafortuitum	WP_083141430	-----------TA K --EF-L-V--D-----
Mycobacterium parascrofulaceum	WP_007169336	-----------A- S --T--LRV--D-----
Mycobacterium paraseoulense	WP_083170295	-----------A- S --T--L-V--D-----
Mycobacterium parmense	WP_085268907	-----------TD K --T--L-V--D-----
Mycobacterium peregrinum	WP_064886204	----A------T- N --TF-K-V--D-----
Mycobacterium phlei	WP_003887825	-----------T- Q --S--L-V--D-----
Mycobacterium porcinum	WP_069425773	----A------T- N --TF-K-V--D-----
Mycobacterium pseudoshottsii	GAQ37687	M------A- A --N--L-V--D-----
Mycobacterium rhodesiae	WP_083118573	--G--------I- Q --T--L-V--D-----
Mycobacterium riyadhense	WP_085251695	-----------T- N --EF-T-V--D-----
Mycobacterium rutilum	WP_083409668	-----------T- Q --T--L-V--D-----
Mycobacterium saskatchewanense	WP_085256153	-----------T- S --S--L-V--D--N--
Mycobacterium scrofulaceum	WP_067281002	-----------A- S --T--L-V--D-----
Mycobacterium septicum	WP_044517208	----A------T- N --TF-K-V--D-----
Mycobacterium setense	WP_039318093	----A------T- N --TF-K-V--D-----
Mycobacterium sherrisii	WP_069398781	-----------AD S --G--L-V--D-----
Mycobacterium shimoidei	WP_069394406	-----------T- K --T--L-V--D-----
Mycobacterium simiae	WP_061556467	-----------AD S --S--LRV--D-----
Mycobacterium smegmatis	WP_003893604	----A------TQ N ---F-T-V--D-----
Mycobacterium szulgai	WP_068032942	-----------TD V --T----VK-D-----
Mycobacterium thermoresistibile	WP_003927258	L----------A- M -----E-V--D-----
Mycobacterium triplex	WP_036470601	----I------AD S --T--L-V--D-----
Mycobacterium tuberculosis	KBZ61444	-----------T- Q --T--L-V--D-----
Mycobacterium tusciae	WP_083126071	-----------T- K --EF-L-V--D-----
Mycobacterium ulcerans	WP_011741561	-----------A- A --N--L-V--D-----
Mycobacterium vaccae	WP_003928807	L----------TA R --EF-L-V--D-----
Mycobacterium vanbaalenii	WP_011779230	-----------TA K --EF-L-V--D-----
Mycobacterium vulneris	WP_065462713	----A------T- N --TF-K-V--D-----
Mycobacterium wolinskyi	WP_067857661	----A------TQ N --TF-K-V--D-----
Mycobacterium xenopi	WP_085195471	-----------A- K --S--L-V--D-----

Supplementary Figure 68

A partial sequence alignment of a conserved region of amidohydrolase showing a one amino acid deletion that is specific for members of the "*Terrae"* clade and absent in other *Mycobacterium*.

Group	Species	Accession	1		29
"Terrae" Clade (10/10)	*Mycobacterium algericum*	WP_083037652	MRLAPLSQSQWD	D	EAVQRALAQVLTADRR
	Mycobacterium engbaekii	WP_085130101	---T---E----	E	----------------
	Mycobacterium heraklionense	WP_064996509	---T---E---E	E	----------------
	Mycobacterium hiberniae	WP_085135826	---T---E----	E	----------------
	Mycobacterium kumamotonense	WP_065288576	---T---E----	-	----------------
	Mycobacterium longobardum	WP_085263242	----E--E-H--	-	AD-------M-PEE--
	Mycobacterium nonchromogenicum	WP_085138482	---T---E---E	E	---------M------
	Mycobacterium senuense	WP_085084351	---T---E----	-	----------------
	Mycobacterium sinense	WP_064854323	---T---A----	-	-E--------------
	Mycobacterium terrae	WP_085261672	---T---E----	-	---------M------
Other *Mycobacterium* (0/70)	*Mycobacterium angelicum*	WP_083111833	---P--PADR--		-T---S--GM-PPE--
	Mycobacterium aromaticivorans	WP_036344078	---T--PAE---		DE--H--
	Mycobacterium arosiense	WP_083063541	---T--PPD---		-QARE---S-VS-E-
	Mycobacterium asiaticum	WP_065036435	---P--PADR--		-S-DQ---AM-P-E--
	Mycobacterium avium	WP_023869728	---Q--PAE---		--TRQ---
	Mycobacterium boenickei	WP_077741544	------PAEE--		DD-R---SVM-PEE-
	Mycobacterium bohemicum	WP_085182543	--VR--PAD---		---EQ--SGM-PPE--
	Mycobacterium branderi	WP_083133906	---S--PADE--		D--RH-V-GM-PEE--
	Mycobacterium brisbanense	WP_062832127	---P--PAD---		DE-L---SVM-P-E-
	Mycobacterium celatum	WP_062540894	---S--PADE--		D--RH-V-GM-PEE--
	Mycobacterium chubuense	WP_014816836	---T--PAEE-N		DD-V---SVL
	Mycobacterium colombiense	WP_064877338	L-----PADE--		-SARA---SLIP---
	Mycobacterium conceptionense	WP_064894126	------PAEE--		DD-R---SVM-PEE-
	Mycobacterium conspicuum	WP_085234756	L-----ADEE--		DQART-V-AL-P-G-
	Mycobacterium europaeum	WP_085242408	------PAD---		DSAH---
	Mycobacterium flavescens	WP_069414148	--V---PAE---		---DG--SALSPER-
	Mycobacterium florentinum	WP_085223754	---P--PAD---		--T-Q--
	Mycobacterium fortuitum	WP_061264225	------PAEE--		DD-R---SVM-PEE-
	Mycobacterium fragae	WP_085198995	---S--PADH--		D-ARD-V-GM-PEE--
	Mycobacterium gilvum	WP_011893320	--V---PAD---		DT-DK--
	Mycobacterium goodii	WP_049748792	---T--PAEE--		DE-R---SVM-PEE-
	Mycobacterium gordonae	WP_065045230	---P--PAD---		---DG---VM-PPE--
	Mycobacterium heckeshornense	WP_048890108	---E--PAD---		-T---S--DM-P----
	Mycobacterium heidelbergense	WP_083072162	---R--PAEE--		-----S-SGM-P-E--
	Mycobacterium holsaticum	WP_069407592	--VL--PAD---		D--EH--
	Mycobacterium houstonense	WP_066898528	------PAEE--		DD-R---SVM-PEE-
	Mycobacterium insubricum	WP_083032286	-L----PAEA-G		-E------PM-P-A--
	Mycobacterium interjectum	WP_066915781	---Q--PAD---		D--EQ--SGM-PPE--
	Mycobacterium intermedium	WP_069418108	---P--PAD---		----KS--GM-PPE--
	Mycobacterium intracellulare	WP_064933357	---R--PAD---		--T-Q--
	Mycobacterium iranicum	WP_064281775	--N--PID---		D--LA--SPL-P-E--
	Mycobacterium kansasii	WP_063469007	---P--PAH---		D--R-S-SGM-PP--
	Mycobacterium kubicae	WP_085075352	---P--PAD---		-S--Q--SGM-PEE--
	Mycobacterium kyorinense	WP_065016301	---S--PAD---		D--RH-V-GM-PEE--
	Mycobacterium lacus	WP_085155979	---Q--PAD---		---R-S--DM-PPE--
	Mycobacterium lentiflavum	CQD20797	---S--PAD---		--T-Q--
	Mycobacterium liflandii	WP_015357174	---P--PAD---		-----S-SVI-P-Q-
	Mycobacterium malmoense	WP_071509747	---Q--PADE--		-T---S-SGM-P-E-
	Mycobacterium mantenii	WP_083096103	---G--PAD---		A--D---SDM-PEA--
	Mycobacterium marinum	WP_012396281	---P--PAD---		-----S-SVI-P-Q-
	Mycobacterium marseillense	WP_083014994	---Q--PAD---		--T-Q--
	Mycobacterium moriokaense	WP_083154760	--VS--PAD---		D--D---
	Mycobacterium nebraskense	WP_046186398	---P--PAD---		-VT-K--
	Mycobacterium neworleansense	CRZ13272	------PAEE--		DN-R---SVM-PEE-
	Mycobacterium noviomagense	WP_083087459	--VE--PAD---		-----S--EM-PE---
	Mycobacterium palustre	WP_085076424	---Q--PADE--		-S-DQ--SGM-PPE--
	Mycobacterium paraense	WP_085093213	--VQ--PAD---		DT-EQ--SGM-PPE--
	Mycobacterium parafortuitum	WP_083145449	--V---PAD---		---DK--
	Mycobacterium parmense	WP_085270189	---G--PAD---		-T---S-SVM-PP-
	Mycobacterium peregrinum	WP_064882650	------PAEE--		DD-R---SVM-PEE-
	Mycobacterium porcinum	WP_075921090	------PAEE--		DD-R---SVM-PEE-
	Mycobacterium rhodesiae	WP_041303643	---P--PAD---		D--DG---VM-PKE--
	Mycobacterium riyadhense	WP_085250648	---P--PAD---		-V---S--GM-PVE--
	Mycobacterium rufum	KGI69179	-I---PAED--		D--D----GLMP-E--

Group	Species	Accession		
Other *Mycobacterium* (0/70)	*Mycobacterium rutilum*	WP_083408009	--V---PAD---	---DG--SSMSPER-
	Mycobacterium saskatchewanense	WP_085255819	--VR--PTDE--	---E-S-SGM-PPE--
	Mycobacterium scrofulaceum	WP_067273816	---P--PAD---	--THQ--
	Mycobacterium septicum	WP_044522274	---P--PAEE--	DD-R---SVM-PEE-
	Mycobacterium setense	WP_039322737	--M---PAEE--	DD-R---SVM-P-E-
	Mycobacterium sherrisii	WP_069398325	---R--PAD---	--TR---
	Mycobacterium shimoidei	WP_069395919	---S--PADA--	D--RH-V-AM-PE---
	Mycobacterium simiae	WP_061557106	---R--PAD---	--T----
	Mycobacterium smegmatis	WP_003896237	---T--PADE--	DE-R---SVM-PEE-
	Mycobacterium szulgai	WP_068031134	---P--PAD---	-S--Q---GM-PEE--
	Mycobacterium thermoresistibile	WP_003926147	------PAE---	DE-LS---VM-PEE--
	Mycobacterium triplex	WP_036471517	---P--PAD---	--T-Q--
	Mycobacterium tusciae	WP_027331288	---P--PAD---	DE-DK---VM-PKE--
	Mycobacterium ulcerans	BAV40026	---P--PAD---	-----S-SVI-P-Q-
	Mycobacterium wolinskyi	WP_067847792	-G----PADE--	DD-R---SVM-PEE-
	Mycobacterium xenopi	WP_003922604	---E--PAD---	-T---S--DM-P-E--

Supplementary Figure 69

A partial sequence alignment of a conserved region of carboxymuconolactone decarboxylase family protein showing a one amino acid insertion that is specific for members of the "*Terrae*" clade and absent in other *Mycobacterium*.

Group	Species	Accession	107		152
"Terrae" Clade (7/7)	*Mycobacterium engbaekii*	WP_085128055	NQVTNFVPFAVSSGVAEVDVRRRGSG	SGE	LEFTKMSVILDVTNSEP
	Mycobacterium heraklionense	WP_064998099	--I------------T--G---KA--	---	------------A----
	Mycobacterium hiberniae	WP_085134240	-----------------IE---T---	---	-----------------
	Mycobacterium kumamotonense	WP_083079854	--I------------T--G---G--S	PSG	----M------------
	Mycobacterium minnesotense	WP_083023869	--I------------T--G---G-KS	RSG	--------V--A-----
	Mycobacterium senuense	WP_085085855	--I-D------------IG---G-RN	ARG	-A---------------
	Mycobacterium sinense	WP_065018972	---------------T--G---G--S	PSR	----T------------
Other *Mycobacterium* (3/64)	*Mycobacterium brisbanense*	WP_062830149	---------------TH-G---DI-A	PKG	-Q----E----------
	Mycobacterium intracellulare	WP_064944095	--IA-----------VN-G---G-A-	PLG	----E-D-V--A---
	Mycobacterium wolinskyi	WP_085144611	---------G------N-A---G-NT	PSG	-Q--RLE----------
	Mycobacterium abscessus	WP_078345540	T-I--S---------VT-S-A--DGE		-R--S-HAV--TQE-V-
	Mycobacterium acapulcensis	WP_066808670	--IA------------D-A---GTN-		----E-EMV--T---
	Mycobacterium alsense	WP_083138857	---A------------T-A---GAG-		----E-DIV--T---V-
	Mycobacterium aromaticivorans	WP_036345307	--I--N---------VDAGI--ASD-		-V--S-T-V--AED-V-
	Mycobacterium arosiense	WP_083064658	--IA-----------VN-G---GAN-		-Q--D-E----A---
	Mycobacterium aurum	WP_048633797	-----N---D------D-GI--A-DA		-VL-S-T-V--AED-I-
	Mycobacterium avium	WP_062886193	-HIA---------A-VN-G---GAH-		---SG-D-T--T---
	Mycobacterium avium subsp. avium	ETB16174	--IA---------A-VN-G---GAH-		---SQ-D----T-S-
	Mycobacterium avium subsp. hominissuis	ETB25129	-HIA---------A-VT-G---GAH-		---SE-D-T--T---
	Mycobacterium avium subsp. paratuberculosis	AAS03757	-HIA---------A-VN-G---GAH-		---SE-D-T--T---
	Mycobacterium bacteremicum	WP_083062085	-----N---D-----VD-AI--AEN-		-VLSS-T-V--AED-I-
	Mycobacterium boenickei	WP_077740273	--I--N---------VDAGI--STD-		-V--S-T-V--AED-V-
	Mycobacterium celeriflavum	WP_083000525	--IA--------------A---GA--		----E-EMV--T---
	Mycobacterium chlorophenolicum	WP_048468852	--I--N----------D-GI--T-D-		-V--S-T-M--ADD-I-
	Mycobacterium chubuense	WP_014814856	--I--H---G-----VD-GI--A-D-		-V--S-T-T--ADD-I-
	Mycobacterium colombiense	WP_064880361	--TA------------N-G---GAG-		----E-DIV--T---
	Mycobacterium conceptionense	WP_065064781	--I--N---------V-AGI--SAD-		-V--S-K-V--AED-V-
	Mycobacterium confluentis	WP_085152144	-----N---D-----VTAGI--GEH-		-V--SLT-L--AED-I-
	Mycobacterium diernhoferi	WP_073858726	-----N---D-----VD-GI--S-DA		-VL-S-T-V--AED-I-
	Mycobacterium elephantis	WP_083042776	-----N---D-----VD-GI--SDD-		-V--S-T----AED-I-
	Mycobacterium europaeum	WP_085241983	--IA-----------VN-G---GPD-		----D-D-V--A---
	Mycobacterium flavescens	WP_069413463	--I--N---D-----VD-GI--ADH-		-V--S-R-V--AED-I-
	Mycobacterium fortuitum	WP_061264413	--I--N---------V-AGI--SAD-		-V--S-T-V--AED-I-
	Mycobacterium fragae	WP_085198812	--IA------------D-A---GSN-		----E-HIV--T---
	Mycobacterium gilvum	WP_011895402	-----N---D-----VD-GI--GEG-		-V--S---V--AED-I-
	Mycobacterium gordonae	WP_065044752	---A-----------V--R---TAER		----E-I-V--T---
	Mycobacterium hassiacum	WP_005627024	-----N---D-----VDAGI--A-D-		-VL-SLK-V--AED-I-
	Mycobacterium holsaticum	WP_069408001	-----N---D-----VD-GI--ADG-		-V--S-T----AED-I-
	Mycobacterium houstonense	WP_066896862	--I--N---------VDAGI--SEA-		-V--SLN-V--AED-I-
	Mycobacterium interjectum	WP_066912132	--IA-----------VD-G---GAN-		---AE-N----T---
	Mycobacterium intermedium	WP_069419238	---A-Y---------V--G---AAD-		----E-DAV--T---
	Mycobacterium komanii	CRL66763	-----N---D-----VD-GI--A-D-		-V--S-T----AED-
	Mycobacterium kyorinense	WP_065012751	--IA------------H-A---CAN-		----E-DIV--T-D-V-
	Mycobacterium litorale	WP_078020302	T--A-H---------L-TAI--GDQ-		-----FDL-M-TD--V-
	Mycobacterium malmesburyense	CRL68990	-----N---D-----VD-GI--A-D-		-V--S-T----AED-
	Mycobacterium malmoense	WP_065445155	--IA-----------VN-G---GPN-		----D-D-V--A---
	Mycobacterium mantenii	WP_083095241	--TA------------T-G---GAD-		----E-DIV--T---
	Mycobacterium moriokaense	WP_083150117	-----AL--------VD-GI-PADH-		-V--S-K----AED-V-
	Mycobacterium mucogenicum	WP_064986483	--I--N---G-----VD-GI--A-DA		-V--S-T-V--AE--I-
	Mycobacterium nebraskense	WP_046183899	--IA----------IVN-A---GDN-		-Q----D-V--A---
	Mycobacterium neoaurum	WP_030137226	-----N---D-----VD-AI--AAE-		-K--S-T-V--AED-I-
	Mycobacterium neworleansense	CRZ16489	--I--N---------VDAGI--SAD-		-V--S-K-V--AED-V-
	Mycobacterium novocastrense	WP_067389372	-----N---D-----VD-GI--ARD-		-V--S-T----AED-
	Mycobacterium obuense	WP_046362778	--I--N----------D-GI--E-D-		-V--S-T-M--ADD-I-
	Mycobacterium paraense	WP_085101101	--IA-----------VN-G---GAD-		-Q--E-D-V--A---V-
	Mycobacterium parafortuitum	WP_083143019	-----N---D-----VD-GI--GE--		-V--S---V--AED-I-
	Mycobacterium paraseoulense	WP_083171509	--IA-----------VD-A---GDN-		-Q--E-D-V--A---
	Mycobacterium peregrinum	WP_064878272	--I--N---------VDAGI-QSED-		-V--SLN-V--AED-V-
	Mycobacterium phlei	WP_061482238	-----N---D-----VD-GI--AEG-		-V--S-R-V--AED-I-
	Mycobacterium porcinum	WP_069426104	--I--N---------VDAGI--SED-		-V--SLN-V--AED-V-
	Mycobacterium rhodesiae	WP_014212499	-----N---D-----VD-GI--GDK-		-V--S---V--AED-I-
	Mycobacterium scrofulaceum	WP_067277048	--IA-----------VN-G---GPN-		----D-D-V--A---
	Mycobacterium setense	WP_064875450	--I--N---------VDAGI--AED-		-V--SLN-V--AED-I-
	Mycobacterium shimoidei	WP_069394862	--IA------------N-A---GAK-		----E-DI---T---

**Other *Mycobacterium* (3/64)**	*Mycobacterium smegmatis*	WP_003894239	-----N---D-----VDAGI--TDD-	-V--SLT----AED-I-
	Mycobacterium thermoresistibile	WP_003925879	-----N---D-----VD-GI--S-D-	-V----T-V--AED-I-
	Mycobacterium tuberculosis	KBZ61842	--IA-----------VN-A---GAN-	----D-D-V--A---
	Mycobacterium tusciae	WP_083125238	--I--N---D-----VD-GI--A-D-	-V--S-R-V--AED-I-
	Mycobacterium vaccae	WP_003929046	-----N---D-----VD-GI--A-D-	-QL-S-K----AED-I-
	Mycobacterium vulneris	WP_065457623	--I--N---------VDAGI--SED-	-V--SLN-V--AED-V-
	Mycobacterium yongonense	WP_020822681	--IA-----------VN-G---GTN-	----E-D----A---

Supplementary Figure 70

A partial sequence alignment of a conserved region of polyketide cyclase showing a three amino acid insertion that is specific for members of the "*Terrae*" clade and absent in most other *Mycobacterium.*

			113		242
***"Terrae"* Clade (6/6)**	*Mycobacterium engbaekii*	WP_085128375	LVQTGMYIYSQV		PFGSIFVGQTHQFPFIFE
	Mycobacterium heraklionense	WP_047317189	------------		------M-----------
	Mycobacterium longobardum	WP_085265802	------------		-I--L-A-------L-V-
	Mycobacterium nonchromogenicum	WP_085139399	------------		------M-E---------
	Mycobacterium senuense	WP_085088783	------------		------M-----------
	Mycobacterium sinense	WP_064854173	------------		------------------
**Other *Mycobacterium* (1/80)**	*Mycobacterium branderi*	WP_083134695	------------		-I--------------I-
	Mycobacterium alsense	WP_083140096	--HM--------	I	-W--V-T-T-F---L-W-
	Mycobacterium angelicum	WP_083114851	--R--F------	I	----A-----Y-----W-
	Mycobacterium aromaticivorans	WP_036339207	--R--L-----A	I	---TL-P-S-F---LLW-
	Mycobacterium arosiense	WP_083064869	--R--F------	I	--------TAY---LLW-
	Mycobacterium asiaticum	WP_065037671	-THW--------	I	-W--V-T-T-F---L-W-
	Mycobacterium avium	WP_062886682	--HM--------	I	-W--V-T-T-F---L-W-
	Mycobacterium avium subsp. avium	EUA38192	--HM--------	I	-W--V-T-T-F---L-W-
	Mycobacterium bacteremicum	WP_083054895	--R--L------	I	------T-T-F---LLW-
	Mycobacterium boenickei	WP_077741510	--R--L-----A	I	---TL-P-S-F---L-W-
	Mycobacterium bohemicum	WP_085180464	--HM--------	I	-W--V-T-T-F---L-W-
	Mycobacterium celatum	WP_062539835	--R--F------	V	----A-A-E-Y----VW-
	Mycobacterium celeriflavum	WP_082999806	--R--L-----A	I	---TM-G-T-F---L-W-
	Mycobacterium chubuense	WP_014815205	--R--L-----A	I	---T--A-S-F---LLW-
	Mycobacterium colombiense	WP_064881853	-IHW--------	I	-W--V-T-T-F---L-W-
	Mycobacterium conceptionense	WP_076211509	--R--L-----A	I	---TL-P-S-F---L-W-
	Mycobacterium conspicuum	WP_085231632	MIH--L-M----	I	--------TPV---LLW-
	Mycobacterium elephantis	WP_046752054	--R---------	P	-WA-V-T--P----L-W-
	Mycobacterium europaeum	WP_085240409	--HM--------	I	-W--V-T-T-F---L-W-
	Mycobacterium flavescens	WP_069411654	--R--F------	V	----V---E-Y-----W-
	Mycobacterium florentinum	WP_085221119	--HA--------	I	-W--V-T-T-F---L-W-
	Mycobacterium fortuitum	WP_061264207	--R--L-----A	I	---TL-P-S-F---L-W-
	Mycobacterium fragae	WP_085195274	-IHW--------	I	-W--V-T-T-F---L-W-
	Mycobacterium gastri	WP_036408610	--R--F------	I	----A-A---Y-----W-
	Mycobacterium gilvum	WP_011895001	--R--L-----A	I	---TL-P-T-F---L-W-
	Mycobacterium gordonae	WP_065050118	-IHW--------	I	-W--V-T-T-F---L-W-
	Mycobacterium hassiacum	WP_005631982	--R---------	P	AW----T-EP----L-W-
	Mycobacterium heckeshornense	WP_083569515	F-RM-F-T-T--	V	-GL-V-A--RY---LVL-
	Mycobacterium heidelbergense	WP_083073314	--H---------	I	-W--V-T-T-F---L-W-
	Mycobacterium holsaticum	WP_069406633	--R---------	P	-WA---T-EP----L-W-
	Mycobacterium houstonense	WP_066898495	--R--L-----A	I	---TL-P-S-F---L-W-
	Mycobacterium indicus pranii	AFS13297	--HW--------	I	-W--V-T-T-F---L-W-
	Mycobacterium insubricum	WP_083029029	--R---------	P	-W----A-D-F---L-W-
	Mycobacterium interjectum	WP_066911702	MIH--L-M----	I	--------TPV---LLW-
	Mycobacterium intermedium	WP_069419864	--R--F------	V	----V-T-E-Y-----W-
	Mycobacterium intracellulare	WP_064933729	--HW--------	I	-W--V-T-T-F---L-W-
	Mycobacterium kansasii	WP_063466698	--R--F------	I	----A-A---Y-----W-
	Mycobacterium komanii	CRL77490	IR--L------	I	--------TPF---L-W-
	Mycobacterium kubicae	WP_085073542	-IHW--------	I	-W--V-T-T-F---L-W-
	Mycobacterium kyorinense	WP_045373581	--R--F------	V	----A-T-E-Y-----W-
	Mycobacterium lentiflavum	CQD19638	--HA--------	I	-W--V-T-T-F---L-W-
	Mycobacterium llatzerense	WP_043987742	--R--L------	I	------T-T-F---LLW-
	Mycobacterium longobardum	WP_085265845	--R---------	I	----V-A-T-F---L-W-
	Mycobacterium malmesburyense	CRL66921	--R---------	P	-WAAV-N-EP----L-W-
	Mycobacterium malmoense	OCB30182	M-RA-IFN-T--	V	--------KPW---L-W-
	Mycobacterium mantenii	WP_083095302	--HM--------	I	-W--V-T-T-F---L-W-
	Mycobacterium marseillense	WP_083016964	--HW--------	I	-W--L-T-T-F---L-W-
	Mycobacterium moriokaense	WP_083149980	--R--L-----A	I	---TL-P-S-F---L-W-
	Mycobacterium nebraskense	WP_046182156	--HM--------	I	-W--V-T-T-F---L-W-
	Mycobacterium neworleansense	CRZ13315	--R--L-----A	I	---TL-P-T-F---L-W-
	Mycobacterium noviomagense	WP_083086191	--R--F------	I	----V-T-K-Y-----W-
	Mycobacterium novocastrense	WP_067390815	--R---------	P	-WA-V-T-D-Y---L-W-
	Mycobacterium paraense	WP_085099644	--HM--------	I	-W--V-T-T-F---L-W-
	Mycobacterium paraffinicum	WP_073879225	--HM--------	I	-W--V-T-T-F---L-W-
	Mycobacterium parafortuitum	WP_083141590	--R--L-----A	I	---TL-P-T-F---LLW-
	Mycobacterium paraintracellulare	AFC52691	--HW--------	I	-W--V-T-T-F---L-W-
	Mycobacterium paraseoulense	WP_083172148	--HM--------	I	-W--V-T-T-F---L-W-
	Mycobacterium parmense	WP_085269354	-IHW--------	I	-W--V-T-T-F---L-W-

**Other *Mycobacterium* (1/80)**	*Mycobacterium peregrinum*	WP_064886223	--R--L-----A I -L-TL-P-S-F---L-W-
	Mycobacterium phlei	WP_003886012	--R--------- P AW----T-E-Y---L-W-
	Mycobacterium porcinum	WP_069425181	--R--L-----A I ---TL-P-S-F---L-W-
	Mycobacterium rhodesiae	WP_005140481	--RS-L-----T I ---TL-P-S-F---L-W-
	Mycobacterium riyadhense	WP_085250833	--R--F------ I ----A---E-Y-----W-
	Mycobacterium rutilum	WP_083409470	--R--F---A-- V ----V---E-Y-----W-
	Mycobacterium saskatchewanense	WP_085253935	--HM-------- I -W--V-T-T-F---L-W-
	Mycobacterium scrofulaceum	WP_067268838	-IHW-------- I -W--V-T-T-F---L-W-
	Mycobacterium septicum	WP_044522354	--R--L-----A I ---TL-P-S-F---L-W-
	Mycobacterium setense	WP_064873527	--R--L-----A I ---TL-P-S-F---L-W-
	Mycobacterium shimoidei	WP_069394380	--R--F------ I ------A-KPY-----W-
	Mycobacterium simiae	WP_061556668	--HL-------- I -W--V-T-T-F---L-W-
	Mycobacterium szulgai	WP_068027727	-IHW-------- I -W--V-T-T-F---L-W-
	Mycobacterium timonense	WP_083187287	--HM-------- I -W--V-T-T-F---L-W-
	Mycobacterium triplex	WP_036471141	--HA-------- I -W--V-T-T-F---L-W-
	Mycobacterium tuberculosis	KBZ59101	-IHW-------- I -W--V-T-T-F---L-W-
	Mycobacterium tusciae	WP_083125894	--R--L-----A I ---TL-P-S-F---L-W-
	Mycobacterium vaccae	WP_003928615	--R--L-----A I ---TL-P-T-F---L-W-
	Mycobacterium vulneris	WP_065460246	--R--L-----A I ---TL-P-S-F---L-W-
	Mycobacterium wolinskyi	WP_085143744	IR--------- I --------TPF---L-W-
	Mycobacterium xenopi	WP_003920773	--R--F------ P ----V-A-K-Y-----W-
	Mycobacterium yongonense	WP_065501086	--H--------- I -W--V-T-T-F---L-W-

Supplementary Figure 71

A partial sequence alignment of a conserved region of spirocyclase AveC family protein showing a one amino acid deletion that is specific for members of the "*Terrae"* clade and absent in most other *Mycobacterium*.

Group	Species	Accession	362		404
“Terrae” Clade (12/12)	*Mycobacterium algericum*	WP_083036336	QAGHVPTAMIFLTEGSVRLT		TEDPAVPAATLRHGAFLGVTTLT
	Mycobacterium arupense	WP_083070785	H-DQ-----T---D----V-		GT--DFAG---GQ-----I----
	Mycobacterium engbaekii	WP_085129690	R--Q-----T---D-T----		AA-----DT---Q-G--------
	Mycobacterium heraklionense	WP_064888912	R--Q-----T----------		SA---W-D---------------
	Mycobacterium hiberniae	WP_085134750	RV-Q-----T---D-A----		AA-----DT---R-G--------
	Mycobacterium icosiumassiliensis	WP_067974574	L--Q-----T---D-----S		G----Q-GT--------------
	Mycobacterium kumamotonense	WP_065288309	---------T--------I-		CA-----D---------------
	Mycobacterium longobardum	WP_085263571	K--------T---A------		DD--DA-GT---------I----
	Mycobacterium minnesotense	WP_083022274	H-DQ-----T--------V-		GA--DFAG---GQ-----I----
	Mycobacterium senuense	WP_085083527	RT-Q----------------		-------D---------------
	Mycobacterium sinense	WP_064921272	---Q--A--T--------I-		GT-----DT--------------
	Mycobacterium terrae	WP_085260278	-------T-T--A-----I-		CA-----D---------------
Other *Mycobacterium* (0/65)	*Mycobacterium africanum*	WP_031667883	H--V--MGIT-VIA------	VTT	DDGSV-AI---KK-T---L-A--
	Mycobacterium angelicum	WP_083114312	H--V--AG-T-VVS------	ATT	EDGSVI-L---AK-------A--
	Mycobacterium aromaticivorans	WP_036345272	RP-ET-SG-T-VLA-T-QMN	AIT	EDGSE-V-W-QDE-S---QS---
	Mycobacterium arosiense	WP_083062914	Y--Q---T-T--VA-R-Q--	ATA	DDGSM--IS--VE-S---L-V--
	Mycobacterium asiaticum	WP_065144138	H--R--EK-T--I---I---	AEG	PDGIP-TVG--DE-S-V---A--
	Mycobacterium avium	WP_062887788	H--R--EK-T--LA-G----	ATA	PDGSV-AVGG-DE-S-----A--
	Mycobacterium avium subsp. avium	EUA35928	H--R--EK-T--LA-G----	ATA	PDGSV-AVGG-DE-S-----A--
	Mycobacterium avium subsp. hominissuis	ETB28521	H--R--EK-T--LA-G----	AAA	PDGSV-AVGG-DE-S-----A--
	Mycobacterium avium subsp. paratuberculosis	EGO37197	D--Q--S--T--VS-R-Q--	ATA	EDGTV--VS--AE-E---L-A--
	Mycobacterium avium subsp. silvaticum	ETB08728	H--R--EK-T--LA-G----	AAA	PDGSV-AVGG-DE-S-----A--
	Mycobacterium bohemicum	WP_085182989	R--E--G--T-VVA-R-QM-	ATA	DDGSV--IS--VE-S---L-A--
	Mycobacterium bovis BCG	AMC51430	H--V--MGIT-VIA------	VTT	DDGSV-AI---KK-T---L-A--
	Mycobacterium branderi	ORA35187	---R--AK-T--IA------	AVA	QDESV-VVG--DD-S-----A--
	Mycobacterium canettii	WP_044096874	H--V--MGIT-VIA------	VTT	DDGSV-AI---KK-T---L-A--
	Mycobacterium chelonae	OHU32190	GS-E--NH-L-IVA-R----	AVG	ADGAEISVG--DE-S---QS---
	Mycobacterium colombiense	OBJ18938	H--R--AG-T--I--G----	ATA	-DGSV-AVGG-DE-S-----A--
	Mycobacterium conspicuum	WP_085235074	H--Q--PG-T--VS-R-Q--	ATA	EDGSI--VT--VQ-SY--L----
	Mycobacterium europaeum	WP_085240252	S--E-SRGVT--VA-R----	ATT	D-GSM--VT--DE-S---L-A--
	Mycobacterium florentinum	WP_085220706	Y--Q--VG-T-VIA-R-QMM	ATA	EDGAV--IS--EA-S---L-A--
	Mycobacterium fragae	WP_085199560	Y--E---G-T--IT------	TTG	EDGSV-ALDA-DE-SY--L-A--
	Mycobacterium franklinii	WP_070939981	DS-E---H-L-IVS-R-L--	AVT	ADGAEISV---DE-S---QS---
	Mycobacterium gastri	WP_036409976	C--E--AE-M-VVA-R----	ATA	DDGSV--IT--EE-G---L-A--
	Mycobacterium goodii	WP_049746565	---E--AQ-S-IVA-Q-VVN	VTG	QD-VV--VR--TE-D---Q-A--
	Mycobacterium gordonae	WP_065045594	Y--T-----A-IVA---Q--	VTD	GDGAVL-F---TE-S-I-I-A--
	Mycobacterium haemophilum	WP_047315039	YT-A--K--T--IA------	ATG	DDGTVM-IG--TE----------
	Mycobacterium heckeshornense	WP_048892728	R--E--AG-A--MQ-----S	VTG	QDGSTTEVG--DE-S-V-L-A--
	Mycobacterium heidelbergense	WP_083072040	Y--Q--SG-T--VA-R-QM-	TTA	DDGSV-AIS--VE-S---L-A--
	Mycobacterium immunogenum	ANO04120	GS-E--SH-L-IVS-R-L--	AGT	A-GSEISVG--DE-S---QS---
	Mycobacterium indicus pranii	WP_014942369	H--R--DK-T--LA-G----	ATA	RDGT--AVGG-DE-S-----A--
	Mycobacterium interjectum	WP_066914166	Y--Q--SG-T--VA-R-Q--	ALA	DDGSI--IS--VE-S---L-A--
	Mycobacterium intracellulare	OBH42359	H--R--DK-T--LA-G----	ATA	RDGT--AVGG-DE-S-----A--
	Mycobacterium kansasii	WP_063467696	C--E--AE-M-VAT-R----	ATA	DDGSV--IT--EE-G---L-A--
	Mycobacterium kubicae	WP_085073368	Y--Q--AG-T--IS-R----	TTA	PDGSV--IS--NE-S-----A--
	Mycobacterium kyorinense	WP_045374565	RV-E-----T--IQ-R-QI-	ATA	DDGST-LTN--DE-S---L-A--
	Mycobacterium lacus	WP_085162933	H--V--K--T-IIA------	AKT	EDGSV--IS--SE-------A--
	Mycobacterium lentiflavum	CQD17376	Y--Q--AG-T-VIA-R-QMM	ATA	EDGSV--IS--EA-S---L-A--
	Mycobacterium liflandii	WP_015356528	Y--Q--AG-T--IT-R----	ATA	EDGSV--VT--HE-G---L-A--
	Mycobacterium malmoense	WP_065442956	ST-Q-SSG-T--I--R----	ATD	DDGSVI-VSI-DE-S---L-A--
	Mycobacterium mantenii	WP_083096992	Y--Q---T-T-VVA-R-Q--	TTA	DDGSM--IS--DE-S---L-A--
	Mycobacterium marinum	WP_012395363	Y--Q--AG-T--IT-R----	ATA	EDGSV--VT--HE-G---L-A--
	Mycobacterium nebraskense	WP_046182129	S--Q-SSG-T--VA-R----	ATT	EDGSVI-VSIIDE-S---L-A--
	Mycobacterium noviomagense	WP_083084376	C--E--RG-S--VA--I--S	VTG	EDGSVTDVG--DE-S-V-L-A--
	Mycobacterium orygis	WP_003412555	H--V--MGIT-VIA------	VTT	DDGSV-AI---KK-T---L-A--
	Mycobacterium palustre	WP_085078206	YV-Q--S--T--VA-R-Q-S	APA	DDGSV--IS--VE-S---L-A--
	Mycobacterium paraense	WP_085094446	Y--Q--RG-T--VA-R-Q--	AMA	DDGSI--IS--VE-S---L-A--
	Mycobacterium paraffinicum	WP_073880139	S--Q-SSG-T--VA-R----	ATT	EDGSV---SI-DE-S---L-A--
	Mycobacterium paraseoulense	WP_083168771	S--Q-SPG-T--VA-R----	ATT	DDGSV--VT--DE-S---L-A--
	Mycobacterium parmense	WP_085267321	R--E-S-G-T--IA-R----	ATA	PDGTSI-VS--DE-E---L-A--
	Mycobacterium phlei	WP_061481894	H--E--KR-S-IVT-RA--V	VTT	DVGAL--VR--ER-D-I-Q----
	Mycobacterium pseudoshottsii	GAQ39819	Y--Q--AG-T--IT-R----	ATA	EDGSV--VT--HE-G---L-A--
	Mycobacterium rhodesiae	WP_083119506	RV-EI--G-W-ILA-T-QMN	AIT	GDGSE-V-W-QDE-S---QS---
	Mycobacterium riyadhense	WP_085250742	H--V--SG-T-IVA------	ATI	EDGSV-AL---HK--M----A--

**Other *Mycobacterium* (0/65)**	*Mycobacterium rutilum*	WP_083408703	Y--E--KR-L-VVT-T---V VTT SDGATIAVR--EA-D---Q----
	Mycobacterium salmoniphilum	WP_078327297	GS-E--HH-L-IVA-R-L-- AAT VDG-EIAVD--DE-S---QS---
	Mycobacterium saskatchewanense	WP_085257990	YT-Q---G-T--VV-R-Q-A ATT DDGSI--IS--AE-S---L-A--
	Mycobacterium scrofulaceum	WP_067283057	S--E-SPG-T--VA-R---- ATT EDGSV--VT--DE-S---L-A--
	Mycobacterium setense	WP_064872047	V--Q--KK-S-VVA-T-Q-A VNG -D-LF--VL--EN-D---Q-A--
	Mycobacterium shimoidei	WP_069394584	H--QI-SG-T--VA-R--M- AMT EDGSMIAVG--NE-S---L----
	Mycobacterium smegmatis	WP_003895031	E--EI--R-S-IVS-R-GVS VAG DG-AVI-VR--TEND---Q-A--
	Mycobacterium szulgai	WP_068033529	Y--Q--AG-T--IS-R---- TTA PDGSV--IS--NE-S-----A--
	Mycobacterium tuberculosis	KBZ61290	Y--Q---T-T--VA-R-Q-- ATA DDGAL--IS--VE-S---L-A--
	Mycobacterium ulcerans	EUA87519	Y--Q--AG-T--IT-R---- ATA EDGSI--VT--HE-G---L-A--
	Mycobacterium vulneris	WP_085291535	Y--Q--AT-T--VS-R-Q-- ATA DDGSV--IS--VE-S---L-A--
	Mycobacterium wolinskyi	WP_085150544	V--E---R-S-IVS-R-S-- VLG KDGAVI-VR--TE-D---Q----
	Mycobacterium yongonense	WP_065500510	H--R--DK-T--LA-G---- ATA RDGT--AIGG-DE-S-----A--

Supplementary Figure 72

A partial sequence alignment of a conserved region of a hypothetical protein showing a three amino acid deletion that is specific for members of the "*Terrae*" clade and absent in other *Mycobacterium*.

			37		83
**"Terrae" Clade (12/12)**	*Mycobacterium algericum*	WP_083035918	QVRATAAAVAEGALDTVRGDIRD	FPA	RTLIWVATRGAAATAGSLLAAA
	Mycobacterium engbaekii	WP_085128011	--------AD--V--GL-EQS-S	Y-P	--I--LS---T---------V
	Mycobacterium heraklionense	WP_064888785	-------T-D-----A--SES--	Y-P	--I--LS---T---------T
	Mycobacterium hiberniae	WP_085134284	---------D--V--GL-EQS-S	Y-P	--I--LS---TG---------
	Mycobacterium icosiumassiliensis	WP_067971117	---------D-----G--SES--	Y-P	--I--LS---T---------T
	Mycobacterium kumamotonense	WP_083079692	---------------A--S-S--	Y-P	----------T--------S--
	Mycobacterium longobardum	WP_085266808	---------D-----AL-S-S--	Y-P	-----LS---T----------
	Mycobacterium minnesotense	WP_083023803	---------D-----G--NES--	Y-P	-SV--LS------M---M---V
	Mycobacterium nonchromogenicum	WP_085137856	---------D-----GI-SES--	Y-P	--I--LS---T---------T
	Mycobacterium senuense	WP_085085060	------------------S----	Y--	--V---S--------------
	Mycobacterium sinense	WP_064856493	---------------A--S-S--	Y-P	----------T--S-----S--
	Mycobacterium terrae	WP_085259103	------------------S-S--	Y-P	----------T--------S--
**Other *Mycobacterium* (0/>100)**	*Mycobacterium abscessus*	WP_074292293	----VGT-I-----EPL-SED-S		-AVV--SG--T-----AI--G-
	Mycobacterium africanum	WP_013988986	----I---AD--E--LL--SD-P		-SV----G--T-E---TI--ST
	Mycobacterium alsense	WP_083138649	----I-G-A------P--AED-A		-SVV---G--T-G---AM---T
	Mycobacterium angelicum	WP_083111133	--SL-S-A-P		--V----G--T-E---AI--ST
	Mycobacterium aromaticivorans	WP_036340273	---------D--E-ALLAA-Y-P		--V----A----ES---M---T
	Mycobacterium arosiense	WP_083064804	-A--I-T--D-D---PL-A-D-P		-SV----G--T-E---TM---T
	Mycobacterium asiaticum	WP_065146934	---TV---SD--E--SL--ED-V		-SV----G--T-E-------ST
	Mycobacterium aurum	WP_048630329	------S-FD--D--P--S-SPP		--VV-I-GP-N-EN--TM--TQ
	Mycobacterium austroafricanum	WP_036370366	--------LD--D--A--S-APP		--IV---GH-N-E----M---L
	Mycobacterium avium	WP_003874556	----I-T--E----EPL-VED-P		-SV----G--T-E---VM-S-T
	Mycobacterium avium subsp. avium	ETB23984	----I-T--E----EPL-VED-P		-SV----G--T-E---VM-S-T
	Mycobacterium avium subsp. hominissuis	ETB32605	----I-T--E----EPL-VED-P		-SV----G--T-E---VM-S-T
	Mycobacterium avium subsp. paratuberculosis	ETB14580	----I-T--E----EPL-VED-P		-SV----G--T-E---VM-S-T
	Mycobacterium bacteremicum	WP_083057396	---------D-----A--A-GPP		-------DS-P-
	Mycobacterium boenickei	WP_077743500	--------L---L--PL-A-HPP		--V----G--T-ES--AM----
	Mycobacterium bohemicum	WP_085182066	---SI---AD-----SLASGD-P		-SV----G--T-E---AI---T
	Mycobacterium bovis	WP_047713313	----I---AD--E--LL--SD-P		-SV---TG--T-E---TI--ST
	Mycobacterium branderi	WP_083130473	--------SD-----P-G--TPR		-----SG----E---TM---T
	Mycobacterium canariasense	WP_062659700	D-----P--S-LPP		--------G-P-V---TM-ST-
	Mycobacterium canettii	WP_015291316	----I---AD--E--LL--SD-P		-SV---TG--T-E---TI--ST
	Mycobacterium celatum	WP_085168397	--------SE-----A----TPR		-V----G--T-E---TM---T
	Mycobacterium celeriflavum	WP_083004869	--------LD--E-ESL-A-GPP		-------G--NSE----V----
	Mycobacterium chelonae	WP_070915200	----VGT-I------PLHVED-S		-A-V---G--T-----AV--G-
	Mycobacterium chlorophenolicum	WP_048471998	--------LD--D-ES--S-VPP		--VV---G--T------M--GV
	Mycobacterium chubuense	WP_083119256	--------LD--D-EP--S-VPP		--VV---G--T------M---V
	Mycobacterium colombiense	WP_064877601	----I----D-----PM-A-D-P		-SI---GG--T-E---TM---T
	Mycobacterium conceptionense	WP_064895290	--------L---V--SL-ANHPP		--V----G--T-EN--AM----
	Mycobacterium confluentis	WP_085148826	---------E--V--PL--ERPR		S----SG--T-ES--VM---V
	Mycobacterium conspicuum	WP_085231473	--SV----Q-----SL-I-E-P		-SV----G--N-E---AM---T
	Mycobacterium diernhoferi	WP_073857703	---------D-----PL-A-VPP		-------DG-P-RA-------V
	Mycobacterium doricum	WP_085190138	--------LD--L-ESL-SGQPP		--V----G--T-E-
	Mycobacterium europaeum	WP_085241663	----I----D-----SL-T-D-P		-S-----G----DS--AM---T
	Mycobacterium fallax	WP_085095231	----V---LD----ARLGSEQPR		C-----G--P-----A
	Mycobacterium farcinogenes	WP_036388735	--------L---V--SL-ANHPP		--V----G--T-EN--AM----
	Mycobacterium flavescens	WP_069415889	--------LD--D-EPL-A-GPP		-------G--P-E----M
	Mycobacterium florentinum	WP_085220020	----I----D--E--SL-TGD-P		-SV----G--T-E---TM---T
	Mycobacterium fortuitum	WP_061262729	--------L---L--PL-A-HPP		--V----G----ES--AM-VG-
	Mycobacterium fragae	WP_085195992	--------LD-----S----D-P		--V----G--T-E---AM-S-T
	Mycobacterium franklinii	WP_070938696	----VGT-I-----EPL-SED-S		-AVV--SG--T-----AI--G-
	Mycobacterium gastri	WP_036417839	----I---HD--E-ELL--GD-P		--V----G--P-E---AM-VST
	Mycobacterium genavense	WP_025737688	----I---AD--E--SL-TGD-P		-SV----G--T-E---AM---T
	Mycobacterium gilvum	WP_011895560	--------LT--D--A--S-SPP		--VV---GP-T-EA--MM---I
	Mycobacterium goodii	WP_049747856	--------LD--LF-RL-A-DPP		--V----N--T-EH--AV---T
	Mycobacterium gordonae	WP_065048928	---SV---LS--E--SL--ED-V		-SV----G----E---A
	Mycobacterium haemophilum	WP_047313332	----I----D---CESL-A-D-P		--V----G--T-E---AM---T
	Mycobacterium hassiacum	WP_005625822	--------LE--E--PL-A-GPP		--VV---G--N-E---A--T-V
	Mycobacterium heckeshornense	WP_048892913	--------FD-----SI--GD-P		---V---G--T--S--AM----
	Mycobacterium heidelbergense	WP_083073182	----I---A---E--SL-AHD-P		-SV----G--T-Q---VI---T
	Mycobacterium holsaticum	WP_069406570	--E--AL-A-QPP		-----L-D--NGQ---P-----
	Mycobacterium houstonense	WP_066903505	--------L---V--PL-S-HPP		--V---GG--T-EH--AI--GV
	Mycobacterium immunogenum	WP_064627952	----VGT-------EPL-SED-S		-AVV-ISG--T-----AI--G-
	Mycobacterium indicus pranii	WP_014942811	----I-T--E-----PL-AED-P		-SV---SG--T-E---AI---T
	Mycobacterium interjectum	WP_085200833	----I---AG----ES--A-D-A		-S-----G--P-E---AM---T
	Mycobacterium intermedium	WP_069418973	-DSVVR		SV---SG--T-----V--TST
	Mycobacterium intracellulare	WP_036459592	----I-T--E-----PL-AED-P		-SV---SG--T-E---AI---T
	Mycobacterium iranicum	WP_064282127	--------LN--D--A--S-TPP		--IV---GH-T-E---TM--VQ
	Mycobacterium kansasii	ORB85870	----I---HD--E--LL-AGD-P		--V----G--T-E---AM-VST
	Mycobacterium komanii	CRL78279	--------L---E-ESLGA-GAP		--V----G--N-E-------S-

Group	Species	Accession	Sequence	Sequence
**Other *Mycobacterium* (0/>100)**	*Mycobacterium kubicae*	WP_085072799	D--D--LL-S-E-P	--I---------E---TM---T
	Mycobacterium kyorinense	WP_065013788	--------ID-----P---ED-P	--V----G--T-E--------T
	Mycobacterium lacus	WP_085161201	---TV---HE--E-ESL---APP	-SV----G--T-E---AM-
	Mycobacterium lentiflavum	CQD08018	----I----E--E--SL-TGD-P	-SV----G--T-E---AM---T
	Mycobacterium leprae	WP_010907917	----I---AE----E-L-AHD-P	--V----G--T-E---AM---T
	Mycobacterium lepromatosis	WP_045842683	----I---AE----EAL-A-D-P	--V--M-G--T-E---AM---T
	Mycobacterium liflandii	WP_015354846	----I---SD--E--SLNI-D-P	--V----G--T-Q---DM---T
	Mycobacterium litorale	WP_078020471	---------E--E-ES-T-GG-P	--M----G----ES--AI---T
	Mycobacterium mageritense	WP_036428456	--------L---L--SL-S-HPP	--V----G----HQ--AM---S
	Mycobacterium malmesburyense	CRL72488	--------L---E-ESL-S-GAP	--V----G--N-E-------S-
	Mycobacterium malmoense	WP_065440968	----IT---D-----SL-A-G-P	-S-V---G--T-DN--AI---T
	Mycobacterium mantenii	WP_083097870	----I-T--E-----PL---D-P	-SV---PG--T-E---TI---T
	Mycobacterium marinum	WP_012393156	----I---SD--E--SLNI-D-P	--V----G--T-Q---DM---T
	Mycobacterium marseillense	WP_083017542	---SI-T--E-----PL-AED-P	-SV----G--T-E---AM---T
	Mycobacterium microti	AMC61019	----I---AD--E--LL--SD-P	-SV---TG--T-E---TI--ST
	Mycobacterium monacense	WP_083044676	--------LD--L-ESL-SGQLP	--V----G--T-E-
	Mycobacterium moriokaense	WP_083154315	--------LD--E--QLQS-QPP	--V----G--N-E---------
	Mycobacterium mucogenicum	WP_064858207	-T--V---LT--E--EL-S-Q-P	--VT--CG--MSGAP-AAGTVL
	Mycobacterium nebraskense	WP_047322004	----I----D-----PL-A-D-P	-S----
	Mycobacterium neoaurum	WP_030134840	----M----G-----E--A-GLA	------SDG-P-RA--TM----
	Mycobacterium neworleansense	CRZ15632	--------L---L--PL-A-HPP	--V----G--T-QN--AM----
	Mycobacterium noviomagense	WP_083087144	--------CD-----SI--GE-P	--VV---S--T-----AM----
	Mycobacterium novocastrense	WP_067395926	---S----L---E-ESL-S-GTP	-------G--N-E---A---S-
	Mycobacterium obuense	WP_046364170	------S-LS--D--S--S-APP	--VV---G--T-SV---M---V
	Mycobacterium paraense	WP_085096040	----I---TD-----SL-T-D-A	-SV----G--T-G---AI---T
	Mycobacterium paraffinicum	WP_073876135	----I----D-----PL-S-D-P	-S-----G--T-DN--AMV--T
	Mycobacterium parafortuitum	WP_083143204	--------LD--D--GL-S-APP	--VV---GP-T-EA---I---V
	Mycobacterium paraseoulense	WP_083169907	H---I----D-----AL-TAD-P	-S-----G----DS--AM---T
	Mycobacterium parmense	WP_085270677	----L---AD--E--SI---D-P	-SV----G--T-E---LM
	Mycobacterium peregrinum	WP_064886440	------------V--PLHA-HPP	--V----G----EN--AM---V
	Mycobacterium phlei	WP_040632790	--------LE--E-EPL-A-GPP	--VV------P-E--------V
	Mycobacterium porcinum	WP_069425818	--------L---L--PL-A-HPP	--V----G--T-ES--AM--V-
	Mycobacterium pseudoshottsii L15	GAQ31884	----I---SD--E--SLNI-D-P	--V----G--T-Q---DM---T
	Mycobacterium rhodesiae	WP_083121493	---------D--E-ALLAA-Y-P	--V----AH---ES---M---T
	Mycobacterium riyadhense	WP_085249836	----I---AD--E--SL---D-A	H-V----G--P-E---AV---T
	Mycobacterium rufum	KGI67330	H-----S--D--D-EP--S-VPP	--VV---G--T-----T----V
	Mycobacterium rutilum	WP_083409914	--------LD--E-EPL-A-GPP	-------G--T-E---L-----
	Mycobacterium salmoniphilum	WP_078323742	----VGT-I-----ESLQSED-S	-AVV---G--T-----AV--G-
	Mycobacterium saopaulense	WP_070909555	----VGT-------EPL-WED-S	-AVV--SG--T-----AI--G-
	Mycobacterium saskatchewanense	WP_085254239	----I----E----ESL---G-P	-SV----G--T-E---AI---T
	Mycobacterium scrofulaceum	WP_083179815	----I----D-----SL-A-G-P	-S-----G--T-DN--AI---T
	Mycobacterium septicum	WP_044516677	--------L---L--PL-A-YPP	--V----G--T-EN--AM----
	Mycobacterium setense	WP_064876312	--------L---V---L-A-RPP	--V----G--T-EN--AI-V--
	Mycobacterium sherrisii	WP_069399787	--D-NSL-T-D-P	-SV----G--T-E---TM---T
	Mycobacterium shimoidei	WP_069395205	--------LD-----P-G--E-P	--V----G--P-E----M---T
	Mycobacterium shinjukuense	WP_083047389	D--D--SL---H-P	--V----G--T-E---AM---T
	Mycobacterium simiae	WP_044510061	-NSL-TED-P	-SV----G--T-E---AV---T
	Mycobacterium smegmatis	WP_011727941	--------LD--LF-PL-A-DPA	--V----G--T-EH--AV---T
	Mycobacterium szulgai	WP_068029037	D--D--LL-S-E-P	--I---------E---TM---T
	Mycobacterium thermoresistibile	WP_003927990	---S----LD--V--PL-S-E-P	--V----G----ES--
	Mycobacterium triplex	WP_036467237	----I---AD--E--SL-TGD-P	-SV----G--T-E---AM---T
	Mycobacterium tuberculosis	WP_070892814	----I---AD--E--LL--SD-P	-SV---TG--T-E---TI--ST
	Mycobacterium tusciae	WP_083125004	--------LD--E--PL-STEPP	--V----G--N-EA--------
	Mycobacterium ulcerans	WP_011740547	----I---SD--E--SLNI-D-P	--V---PG--T-Q---DM---T
	Mycobacterium vaccae	WP_060941617	--------LD--D--A--S-SPP	---V---NA-N-E---AM-S-Q
	Mycobacterium vanbaalenii	WP_041307728	--------LD--D--A--S-APP	--IV---GH-N-E----M---L
	Mycobacterium vulneris	WP_065462522	--------L---L--PL-A-HPP	--V----G--T-ES--AM----
	Mycobacterium wolinskyi	WP_085147128	--------FS--T-EPL-S-HPP	--V----G--V-E---AM----
	Mycobacterium xenopi	WP_085196769	--------SD-----SI--GD-P	---V---G--T--S--AM----
	Mycobacterium yongonense	WP_065503904	----IVT--E-----PL-AED-P	-SV---SG--T-E---AI---T

Supplementary Figure 73

A partial sequence alignment of a conserved region of TobH protein showing a three amino acid insertion that is specific for members of the "*Terrae*" clade and absent in other *Mycobacterium.*

			131		177
	Mycobacterium algericum	WP_083038184	SMLVVALQHCGLDPSFAVGGELG	SAGA	EAGTNAHHGSGPLFVAEADE
	Mycobacterium arupense	WP_046188927	--------R--------------	----	--------------------
	Mycobacterium engbaekii	ORV46924	--------R--------------	---S	--------------------
	Mycobacterium heraklionense	WP_047321033	-----------------------	---S	--------------------
	Mycobacterium hiberniae	ORV69767	--------R--------------	---S	--------------------
	Mycobacterium icosiumassiliensis	WP_067973925	-----------------------	---S	--------------------
**"*Terrae*" Clade (13/13)**	*Mycobacterium kumamotonense*	WP_065288361	-----------------------	----	------------F-------
	Mycobacterium longobardum	WP_085264049	-----------------------	----	--------------------
	Mycobacterium minnesotense	WP_083025168	--------R--------------	---S	--------------------
	Mycobacterium nonchromogenicum	WP_085139896	-----------------------	---S	--------------------
	Mycobacterium senuense	WP_085081587	-----------------------	----	------------F-------
	Mycobacterium sinense	WP_064855952	-----------------------	----	------------F-------
	Mycobacterium terrae	WP_085262732	-----------------------	----	------------F-------
	Mycobacterium koreense	WP_085302354	---------S-------------		-S-------T-AI-------
	Mycobacterium triviale	WP_069390996	-----------------------		-S-------T-AI-------
	Mycobacterium abscessus	WP_074337205	---I-------Y----------N		-----------DV-------
	Mycobacterium acapulcensis	WP_083997560	---I-----S-F--------D--		-------N---DV-------
	Mycobacterium africanum	WP_031670187	---I-------------------		-----------DC-------
	Mycobacterium alsense	WP_083140476	---I-------R-----------		-----------DC-------
	Mycobacterium angelicum	WP_083114602	---I-------R-----------		--A--------DY-------
	Mycobacterium aromaticivorans	WP_036344680	---I-----A----------D--		-------N---DY-I-----
	Mycobacterium arosiense	WP_083064958	---I-------R---------M-		-----------DC-------
	Mycobacterium asiaticum	OBK16804	---I-------R-----------		-----------DY-------
	Mycobacterium aurum	WP_048631869	---------S-F-----I--D-A		A----------SA-------
	Mycobacterium austroafricanum	WP_084182373	---I-----S-F--------D--		-----------SS-------
	Mycobacterium avium	WP_003872225	---I-------R---------M-		-----------DC-------
	Mycobacterium avium subsp. avium	ETB16844	---I-------R---------M-		-----------DC-------
	Mycobacterium avium subsp. hominissuis	ETB34241	---I-------R---------M-		-----------DC-------
	Mycobacterium avium subsp. paratuberculosis	ETB39984	---I-------R---------M-		-----------DC-------
	Mycobacterium avium subsp. silvaticum	ETB10309	---I-------R---------M-		-----------DC-------
	Mycobacterium bacteremicum	WP_083055698	---I-----S-F--------D--		A-----------Y-------
	Mycobacterium bohemicum	ORV03595	---I----R--R-----------		-----------DC-------
	Mycobacterium bovis	WP_024457681	---I-------------------		-----------DC-------
	Mycobacterium branderi	WP_083132020	---I-------K---------M-		-----------DY-I-----
	Mycobacterium brisbanense	WP_062830379	---I-----S-F-----------		-----------RC-------
	Mycobacterium canariasense	WP_062654663	---I-----S-F--------D--		A-----------F-------
	Mycobacterium canettii	WP_014001154	---I-------------------		-----------DC-------
	Mycobacterium celatum	WP_084707405	---I-------F-----------		-----------DY-------
**Other *Mycobacterium* (0/>100)**	*Mycobacterium celeriflavum*	WP_083000690	---I-----S-F--------D--		-------N---DC-------
	Mycobacterium chelonae	WP_075907647	---I-------Y----------N		-----------DV-------
	Mycobacterium chimaera	WP_054585458	---I-------R---------M-		-----------DC-------
	Mycobacterium chlorophenolicum	WP_048470822	---I-----S-F--------D--		-P---------AY-------
	Mycobacterium chubuense	WP_014816387	---I-----S-F--------D--		-------R---TC-------
	Mycobacterium colombiense	WP_007770762	---I-------R---------M-		-----------DC-------
	Mycobacterium confluentis	WP_085155605	---I-----A-M--------D--		-----------GC-------
	Mycobacterium conspicuum	WP_085232961	---I-------R-----------		-----------DC-------
	Mycobacterium cosmeticum	CDO06435	---I-----S-F-----------		A-----------Y-------
	Mycobacterium diernhoferi	WP_073854168	---I-------F-----------		A-----------Y-------
	Mycobacterium doricum	WP_085192565	---I-----G-F--------D--		-----------QC-------
	Mycobacterium elephantis	WP_083042851	---I-----S-F--------D--		-------N---DC-------
	Mycobacterium europaeum	WP_085243183	---I-------R---------M-		-----------DC-------
	Mycobacterium fallax	WP_085099298	---I-----A-------------		G----------DI-------
	Mycobacterium flavescens	WP_069416437	---I-----S-F-----------		A----------RY-------
	Mycobacterium florentinum	ORV56132	---I-------G--------D--		-----------DC-------
	Mycobacterium fortuitum	WP_065148747	---I-----S-F-----------		-----------KC-------
	Mycobacterium fragae	WP_085199839	---I-------K-----------		-----------DC-------
	Mycobacterium franklinii	WP_070935036	---I-------Y----------N		-----------DV-------
	Mycobacterium gastri	WP_036414978	---I-------R-----------		-----------DC-------
	Mycobacterium genavense	WP_025735404	--------Q--R--------D--		-----------DC-------
	Mycobacterium gilvum	WP_011893867	--II-------F--------D--		Q----------ST-------
	Mycobacterium goodii	WP_049746061	---I-----S-F-----------		-----------TT-------
	Mycobacterium gordonae	WP_065047790	---I-------R-----------		-----------D--------
	Mycobacterium haemophilum	WP_054880935	---I-------R-----------		-----------DC-------
	Mycobacterium hassiacum	WP_005628190	---I-----A-Y--------D--		-----------DC-------

Other *Mycobacterium* (0/>100)	*Mycobacterium heckeshornense*	WP_048892083	-----------F-----------	-----------DY-------
	Mycobacterium heidelbergense	WP_083072391	---I-------R-----------	-----------DC-------
	Mycobacterium holsaticum	WP_069407944	---I-----S-F--------D--	-------N---RV-------
	Mycobacterium houstonense	WP_066897879	---I-------F-----------	-----------TC-------
	Mycobacterium immunogenum	WP_043077570	---I-------Y----------N	-----------DV-------
	Mycobacterium insubricum	WP_083029758	---I-G---A-Q--------D--	-----------SI-------
	Mycobacterium interjectum	WP_084454199	---I-------R--------D--	-----------DC-------
	Mycobacterium intermedium	WP_069419517	---I-------R-----------	-----------DC-------
	Mycobacterium intracellulare	WP_064937490	---I-------R---------M-	-----------DC-------
	Mycobacterium iranicum	WP_024445462	--II-----S-F--------D--	Q----------SC-------
	Mycobacterium kansasii	WP_063470527	---I-------R-----------	-----------DC-------
	Mycobacterium komanii	CRL67008	---I-----S-F--------D--	-------N---DC-------
	Mycobacterium kubicae	WP_085075452	---I-------R-----------	-----------DS-------
	Mycobacterium kyorinense	WP_065012786	---I-------C---------M-	-----------DC-------
	Mycobacterium lacus	WP_085161898	---I-------R-----------	-----------DC-------
	Mycobacterium lentiflavum	CQD15912	---I----Q--R--------DF-	-----------DC-------
	Mycobacterium leprae	CAA18667	-----------C----V-----A	VV-------L-AC-------
	Mycobacterium lepromatosis	WP_045842790	-L---------C----V-----A	AV---G---L-DC-------
	Mycobacterium liflandii	WP_015356132	--VI-------R-----------	-----------DY-------
	Mycobacterium litorale	WP_078018937	--VI-----S-F--------D--	-------N----Y----G--
	Mycobacterium llatzerense	WP_043987133	--VI-----S-F--------D--	-----------DC-------
	Mycobacterium mageritense	WP_036439097	--II-----S-F-----------	-----------RC-------
	Mycobacterium malmesburyense	CRL74445	---I-----S-F--------D--	-------N---DC-------
	Mycobacterium malmoense	OCB26850	---I-------R---------M-	-----------DC-------
	Mycobacterium mantenii	WP_083098747	---I-------R---------M-	-----------DC-------
	Mycobacterium marinum	WP_012394858	--VI-------R-----------	-----------DY-------
	Mycobacterium marseillense	WP_083018438	---I-------R---------M-	-----------DC-------
	Mycobacterium moriokaense	WP_083152904	---I-S-----F--------D--	-----------DC-------
	Mycobacterium mucogenicum	WP_064858721	--VI-----S-F--------D--	-----------DC-------
	Mycobacterium nebraskense	WP_047323635	---I-------R---------M-	-----------DC-------
	Mycobacterium neoaurum	WP_030136398	---I-----S-F--------D--	A-----------Y-------
	Mycobacterium neworleansense	CRZ13810	---I-----S-F--------D--	-P---------AY-------
	Mycobacterium noviomagense	WP_083088889	---I-------------------	-----------DY-------
	Mycobacterium novocastrense	WP_084377358	---I-----S-F--------D--	-----------AY-------
	Mycobacterium obuense	WP_046366732	---I-------F--------D--	-P---------AY-------
	Mycobacterium palustre	WP_085081207	---I-------R-----------	-----------DC-------
	Mycobacterium paraense	ORW28730	---I-------R---------M-	-----------DC-------
	Mycobacterium paraffinicum	WP_073875657	---I-------R---------M-	-----------DC-------
	Mycobacterium parafortuitum	WP_083146061	--TI-------F--------DI-	QS---------GT-------
	Mycobacterium paraseoulense	WP_083172745	---I-------R---------M-	-----------DC-------
	Mycobacterium parmense	WP_085267177	---I-------R-----------	-----------DC-------
	Mycobacterium peregrinum	WP_064883080	---I-----S-F-----------	-----------KC-------
	Mycobacterium phlei	WP_003888249	---I-----A-F-----------	A-----------Y-------
	Mycobacterium porcinum	WP_069427243	---I-----S-F--------D--	-P---------AY-------
	Mycobacterium rhodesiae	WP_014212789	---I-------F--------D--	-----------DC-------
	Mycobacterium riyadhense	WP_085251650	---I-------R-----------	-----------DC-------
	Mycobacterium rufum	KGI68751	---I-------F--------D--	-P---------AY-------
	Mycobacterium rutilum	WP_083408399	---I-----S-F-----------	A----------DY-------
	Mycobacterium salmoniphilum	WP_078326422	---I------EY----------N	-----------DV-------
	Mycobacterium saopaulense	WP_083013699	---I------EY----------N	-----------NV-------
	Mycobacterium saskatchewanense	WP_085254083	---I-------R-----------	-----------DC-------
	Mycobacterium scrofulaceum	WP_067274465	---I-------R---------M-	-----------DC-------
	Mycobacterium septicum	WP_044523712	---I-----S-F-----------	-----------KC-------
	Mycobacterium setense	WP_064872884	---I-----S-F-----------	-----------NC-------
	Mycobacterium sherrisii	WP_069400480	---I----Q--R--------D--	-----------DC-------
	Mycobacterium shimoidei	WP_069397881	-----------K---------M-	-----------DC-------
	Mycobacterium shinjukuense	WP_083046886	---I-------R-----------	-----------DC-------
	Mycobacterium simiae	AMP22035	---I----Q--R--------D--	-----------DC-------
	Mycobacterium smegmatis	WP_011729655	---I-----S-F-----------	-----------TT-------
	Mycobacterium szulgai	WP_068026658	---I-------R-----------	-----------DC-------
	Mycobacterium thermoresistibile	WP_003927677	---T-----G-F--------D--	-----------TV-------
	Mycobacterium timonense	WP_083187587	---I-------R---------M-	-----------DC-------
	Mycobacterium triplex	WP_036469793	--------Q--R--------D--	-----------DC-------
	Mycobacterium tuberculosis	WP_063741634	---II------------------	-----------DC-------
	Mycobacterium tusciae	WP_083125481	---I-T-----F--------D--	-----------DC-------

**Other *Mycobacterium* (0/>100)**	*Mycobacterium ulcerans*	WP_011741261	--AI-------R-----------	-----------DY-------
	Mycobacterium vaccae	WP_003933041	---I-------F--------D--	-----------SC-------
	Mycobacterium vanbaalenii	WP_011780721	---I-----S-F--------D--	-----------SS-------
	Mycobacterium vulneris	WP_065458633	---I-----S-F--------D--	-P---------AY-------
	Mycobacterium wolinskyi	WP_067855501	---I-----S-F--------D--	-----------RC-------
	Mycobacterium xenopi	WP_003919031	---I-------F-----------	-----------DY-------
**Other bacteria**	*Actinoalloteichus cyanogriseus*	WP_051314227	---T----R-RV-----I--D-N	-S-A-----T-GI-------
	Amycolatopsis azurea	WP_005156464	---T------R------I--D-N	-S-A-----E-G--------
	Corynebacterium ciconiae	WP_040357892	--A---M-QA-M-----I--Q-N	K--------T-TC-------
	Gordonia araii	WP_007322239	--A-A------V----------N	-S---------DV-------
	Hoyosella subflava	WP_013806129	-L--------K-----------N	---------T-EV-------
	Kribbella catacumbae	WP_020387370	---T-------V---Y-I--N-N	-S-S---D---D--------
	Lentzea albida	SER12439	---T------R------I--D-N	-S-A---Q-T-GV-------
	Millisia brevis	WP_084351587	--VI----QA-V----------N	-S---------GV-------
	Nocardia beijingensis	WP_067811169	---I-S-----F----------N	---------T-DI-------
	Pseudonocardia thermophila	SHK85537	---T-------------I--D-T	S--G--E---EI-------
	Rhodococcus coprophilus	WP_072699107	-----------F----------N	-S---------DV-------
	Saccharopolyspora antimicrobica	SF097086	---T------RF-----I--D-N	-S-A-----D-SI-------
	Segniliparus rotundus	WP_013139465	-LT-A---QA-------------	-------Q---NA-------
	Skermania piniformis	WP_066473291	---I-------F----------N	-----------DV-------
	Streptomyces thermoautotrophicus	WP_066888158	---T-------A----SI--Q-N	-S-----D---EI-------
	Tsukamurella pulmonis	KX087957	--A--------AN---------N	-S----F--T-DV-------
	Williamsia sterculiae	WP_076482410	--A------A------------N	-S-------A-GI-------

Supplementary Figure 74

Detailed sequence information for the four amino acid insertion found in UDP-N-acetylmuramate--L-alanine ligase, which is shown in Figure 9B. This insertion is specific for members of the "*Terrae"* clade and absent in other bacteria.

			145		190
"Terrae" Clade (10/10)	*Mycobacterium engbaekii*	WP_085129074	VDVHRIFLGEMDDEHADRHYH	N	EGKAMATMLQVPEDMWPADRAAFD
	Mycobacterium heraklionense	WP_064890616	--I-----------------R	-	------------------------
	Mycobacterium hiberniae	WP_085135587	---------------------	-	------------------------
	Mycobacterium icosiumassiliensis	WP_067967925	--I-----------------R	-	-------------E----------
	Mycobacterium kumamotonense	WP_065286917	---------------------	-	------------------N-----
	Mycobacterium longobardum	WP_085263369	--I------------------	-	------------------------
	Mycobacterium minnesotense	WP_083025839	---------------------	H	------------------------
	Mycobacterium senuense	WP_085082534	---------------------	-	------------------------
	Mycobacterium sinense	WP_064856436	--I------------------	-	-----------S------------
	Mycobacterium terrae	WP_085260894	-------------D-------	-	-----------------T------
Other *Mycobacterium* (0/>100)	*Mycobacterium koreense*	WP_085303440	--LY-M-Y-----DE-E-A-Q		-ARIFG-A---RPE----------
	Mycobacterium triviale	WP_085111193	--LY-M-Y----E-E-E-G-Q		-A-IFG-A---RPE----------
	Mycobacterium abscessus	WP_062879344	---YEA-V-PLTG-M-E-CLQ		--AV-G-T--M-LA---KSC-E--
	Mycobacterium abscessus subsp. bolletii	SKN28692	---YEA-V-P-TPDM-E-CLQ		--AV-G-T--M-LA---KSC-E--
	Mycobacterium africanum	WP_031668172	---Y-T-V------E--H--R		A-M--G-T----PQ---P------
	Mycobacterium alsense	WP_083137533	-EQ-EFLH-PL--AT--AV-A		DARRLG-T-----R---P-----E
	Mycobacterium angelicum	WP_083113875	--IY-T-V--IPE-D---L-R		--MSIG-T----PEL--P------
	Mycobacterium aromaticivorans	WP_036342866	---A-V-I------T-----R		-SA-L--T----AE----------
	Mycobacterium arosiense	WP_083065475	--IY-M-I--LEG-D-----R		--MTL--T----PK----------
	Mycobacterium asiaticum	WP_065035666	I--Y-L-V--I---D---L-Q		Q-M-IG-T----PE----------
	Mycobacterium aurum	WP_048632484	-----L-V------T---L-L		Q-R-L--------T----------
	Mycobacterium austroafricanum	WP_036369125	-----L-V----E-T---L-L		Q-R-L-------------------
	Mycobacterium avium	WP_062899462	L-IY-M-I--L-GDD-----R		--MTL--T----PA---P------
	Mycobacterium avium	WP_019736611	---------------------	-	------------Q-----------
	Mycobacterium avium subsp. avium	EUA39056	L-IY-M-I--L-GDD-----R		--MTL--T----PA---P------
	Mycobacterium avium subsp. hominissuis	BAN33368	L-IY-M-I--L-GDD-----R		--MTL--T----PA---P------
	Mycobacterium avium subsp. paratuberculosis	ELP44663	L-IY-M-I--L-G-D-E---R		--M-L--T----PK----------
	Mycobacterium bacteremicum	ORA06140	--IR---V--I---T--Q--R		D-ITL--T---TPE----------
	Mycobacterium boenickei	WP_077740083	---F-M-V--L---T-----R		-SVT-G-T-----E----------
	Mycobacterium bohemicum	WP_085182175	M-IY-T-I--L--RE---LHR		Q-MSLG-T----PQ----------
	Mycobacterium bovis	WP_080655122	--Q-EFLY-PLE-AT--AV-Q		DA-RLG-T-----G---P--V---
	Mycobacterium branderi	WP_083131785	--IY---V-----DE-E---R		--M-LG-T----PE----------
	Mycobacterium brisbanense	WP_062829805	---Y-M-V------T-----R		--HTLG-T----PE----------
	Mycobacterium canariasense	WP_062654581	--Q-EFLY-PL---S--AV-A		DARRLG-T---RAE---P--S---
	Mycobacterium canettii	WP_080627842	--Q-EFLY-PLE-AT--AV-Q		DA-RLG-T-----G---P--V---
	Mycobacterium celatum	WP_062540877	--IY---V------E-E---R		--M-LG-T----PE----------
	Mycobacterium celeriflavum	WP_083005925	---F---V------S-----R		DAA-LG-T----AE------K---
	Mycobacterium chelonae	WP_070915764	---YEA-V-P-TPDM-E-CLQ		--AV-G-T--M-LA---KSC-E--
	Mycobacterium chlorophenolicum	WP_082168669	-----L-V--L--DT--QLFV		Q-RKLG------------------
	Mycobacterium chubuense	WP_082162364	-----L-V--L---T---LFV		Q-RKLG------------------
	Mycobacterium colombiense	WP_064877199	--Q-EFLH-PL--AT--TV-R		DAAKLG-T-----R---P--T---
	Mycobacterium colombiense	WP_064877737	L-IY-M-I--L-G-D-----R		--MTL--T----PK----------
	Mycobacterium conceptionense	WP_065063279	---F-M-I--L---I--Q--R		DSVT-G-T-----E----------
	Mycobacterium confluentis	WP_085150815	--Q-EFLY-PL--AA--AV-R		DARKLG-T---R--------V--E
	Mycobacterium conspicuum	WP_085231751	---Y-T-I--IPE-D---L-R		--MSIG-T----PE----------
	Mycobacterium cosmeticum	WP_036396773	--Q-EYLY-PL---S--AV-A		DARTLG-T---HAE---P------
	Mycobacterium diernhoferi	WP_073855592	---Y-L-I----EAT-----R		D-ITLG-T----AE----------
	Mycobacterium doricum	WP_085189363	-E-F-L-V-----QT-EE--R		DSMT-G-T----PQ----------
	Mycobacterium elephantis	WP_083043188	---Y-L-V----E-T-E---E		--ITLG-T---------K-G----
	Mycobacterium europaeum	WP_085242281	--Q-EFLH-PL--AS--AV-L		DARRL--T-----R---PN-----
	Mycobacterium flavescens	WP_069412598	---Y---V------S----FA		--A-LG-T----AE------K---
	Mycobacterium flavescens	WP_069412807	T-Q-EFLY-PL---A--AV-Q		DARKLG-T---RAE---P--V---
	Mycobacterium florentinum	WP_085222547	I--Y-T-V--L-EQD---L-R		--MSLG-T----PE----------
	Mycobacterium fortuitum	WP_064848048	---F-M-I--L---T--Q--R		DSVT-G-T-----E----------
	Mycobacterium fragae	WP_085197788	E-TY-LLR---T--L-EHF-R		SAFTLG-T---S--Q--PT-RD--
	Mycobacterium franklinii	ORA58452	T-TTTLLY-PIP-AE--AL-Q		HCARLG-T--L-PA---------A
	Mycobacterium gastri	WP_036410200	A------V--L--AE-E---R		-TM-L--T----PE---P------
	Mycobacterium genavense	WP_025736514	I--Y-T-I--IPE-D---L-R		--MSLG-T----SE----------
	Mycobacterium gilvum	WP_011891331	-----L-V----E-T---V-V		Q-R-L--------S------E---
	Mycobacterium goodii	WP_049743338	---L-M-V------T-----R		DSVT-G-T----AQ----------
	Mycobacterium gordonae	WP_065042865	I-IY-T-V--LT--V---L-Q		--MSIG-T----IE-----V----
	Mycobacterium haemophilum	WP_047315900	--IY---I--LS--D-----R		--MTL--T----SE---P------
	Mycobacterium hassiacum	WP_005625895	E-T-QLLR---TP-Q-EQF-R		SAWTLG-T---T--Q--PN-----
	Mycobacterium heckeshornense	WP_071700113	-------V------E-E---R		--M-LG-T----PE---P--E--Y

**Other *Mycobacterium* (0/>100)**	*Mycobacterium heidelbergense*	WP_083076047	--I--M-I--L-GAD-----R	--MTLV-T----PER---------
	Mycobacterium holsaticum	WP_069404914	--Q-EFLY-PL--SA--AV-R	DARKLG-T---RA----P---S--
	Mycobacterium houstonense	WP_066897797	I-M-EYLY-PL-S-S--AV-A	DARTLG-T---RD----E--Q---
	Mycobacterium immunogenum	WP_064627269	---YEA-V-PLTG-M-E-CLQ	--AV-G-T--M-LA---KSC-E-E
	Mycobacterium insubricum	WP_083029036	---YNM-I-HL---A-EK--R	-SAVLG-T----LE---E------
	Mycobacterium interjectum	WP_066913529	-EQ-EFLH-PL--AT--AV-R	DA-RLG-T-----R---Q------
	Mycobacterium intermedium	WP_069420010	I-Q-EFLH-PL--AT--AV-R	DA-RLG-T-----R---P--V---
	Mycobacterium intracellulare	WP_064936470	L-IY-M-I--L-G-D-E---R	--M-L--T----PK----------
	Mycobacterium iranicum	WP_064281569	-----V-V-D--EQT---L-L	Q-R-L--------S----------
	Mycobacterium kansasii	WP_063467071	A------V--L--AE-----R	-TM-L--T----PE---P------
	Mycobacterium komanii	CRL72795	--Q-EFLY-PL---A--AI--	DARKLG-T---R-----P------
	Mycobacterium kubicae	WP_085075629	--IY-T-V--I---Q-----R	--MT-G-T----PE----------
	Mycobacterium kyorinense	WP_065014462	--FY-T-I--I-EAD-EQ--R	--MVLG-T----PE----------
	Mycobacterium lacus	WP_085159074	I--Y-T-V--I---E---L-R	--M-LG-T----PQ---P------
	Mycobacterium lentiflavum	CQD04404	I-IY-T-I---TEQD---R-R	D-MSLG-T----PE----------
	Mycobacterium liflandii	WP_015354275	--IY-T-V------E-----R	D-MS-G-T----AEL--P------
	Mycobacterium litorale	WP_078017570	--IA-V-I-D---DT-----R	DSM-L--T----A-----------
	Mycobacterium llatzerense	WP_043985873	L--Y-LVV--L---S-EEF-R	Q-MT-G-T---TPE----------
	Mycobacterium mageritense	WP_019345551	--M-EFLY-PL-E-S--AI-A	DARTLG-T---RDE---Q--N---
	Mycobacterium malmesburyense	CRL71400	I-Q-EFLY-PL--DS--AI-Q	DARKLG-T---R-----P------
	Mycobacterium malmoense	WP_065443811	--IY-M-I--L-G-D-----R	--M-L--T----PE----------
	Mycobacterium mantenii	WP_083099191	--Q-EFLH-PL--AT--AV-R	NASRLG-T-----R---P--H---
	Mycobacterium marinum	WP_012392504	--IY-T-V------E-----R	D-MS-G-T----AEL--P------
	Mycobacterium marseillense	WP_083019298	L-IY-M-I--L-G-D-E---R	--MTL--T----PK----------
	Mycobacterium moriokaense	WP_083152803	---Y---V------L--A--R	--A-LG-T----AE------K---
	Mycobacterium mucogenicum	WP_061003145	L--Y-LVV--L---T-EEF-R	Q-MT-G-T---TPE----------
	Mycobacterium nebraskense	WP_085164193	--IY-M-I--L-G-D-----R	--M-L--T----PE----------
	Mycobacterium neoaurum	CDQ43470	---R---V--V-E-T--Q--R	D-M-LG-T---TPE----------
	Mycobacterium neworleansense	CRZ16696	---F-M-I--L---T--Q--R	DSVT-G-T-----S----------
	Mycobacterium noviomagense	ORB13170	--Q-EFLH-PL--AT--AV-R	DA-RLG-T---R-----P--V---
	Mycobacterium novocastrense	WP_067389054	I-Q-EFLY-PL---A--AI-L	DARKLG-T---R-----P------
	Mycobacterium obuense	WP_082133533	L--Y-L-V--V-ADTEA-LFE	Q-RKLG-------------P----
	Mycobacterium palustre	WP_085078331	---Y-M-V--L-G-D-----R	--M-LG-T----AER---------
	Mycobacterium paraense	WP_085102322	I-IY-T-V------E---F-R	D-MTLG-T----PE---P------
	Mycobacterium paraffinicum	WP_073877650	---Y-M-I--LEG-D-E---R	--M-L--T----PE---P------
	Mycobacterium parafortuitum	WP_083142378	-----L-V----EQA---L-L	Q-R-L--------R---ES-----
	Mycobacterium parascrofulaceum	EFG78097	--Q-EFLH-PL-GAA--AV-L	NARRLG-T-----R---P------
	Mycobacterium paraseoulense	WP_083172857	--Q-EVLH-PL-EAS--AV-L	DARRL--T-----S---P-----G
	Mycobacterium parmense	WP_085267374	---Y---V--LGAQD-----G	--M-LG-T----PA---K------
	Mycobacterium peregrinum	WP_064881655	--M-EFLY-RL-E-S--AI-A	DAR-LG-T---RDE---E--D---
	Mycobacterium phlei	AMO62183	--Q-EFLY-PL---S--AV-Q	DARTLG-T---RDG---Q------
	Mycobacterium porcinum	WP_069424765	--M-EFLY-PL---S--AV-A	DARTLG-T---RDE---E--N---
	Mycobacterium pseudoshottsii	GAQ32567	--IY-T-V------E-----R	D-MS-G-T----AEL--P------
	Mycobacterium rhodesiae	WP_083117346	---A-V-I------T-----R	-SA-L--T----AE----------
	Mycobacterium riyadhense	WP_085250031	--Q-EFLH-PL--AS--LV-R	DA-RLG-T-----R---P--V---
	Mycobacterium rufum	KGI66431	-----L-I--P-E-T---LFV	Q-RKLG-------------QN---
	Mycobacterium rutilum	WP_083406068	---Y---V------S-----R	--ATLG-T----AE------K---
	Mycobacterium salmoniphilum	WP_078326013	---YEA-V-P-SADM-E-CLQ	--AV-G-T--M-LA---KSC-E--
	Mycobacterium saopaulense	WP_070911911	---YEA-V-P-TG-M-E-CLQ	--AV-G-T--M-LA---KSC-E--
	Mycobacterium saskatchewanense	ORW73397	--IY-T----LS--D-----R	--M-L--T----PER---------
	Mycobacterium scrofulaceum	WP_067269561	--Q-EFLH-PL--AT--AV-L	DAARLG-T-----G---P------
	Mycobacterium septicum	WP_044519126	---F-M-I--L---T-----R	DSVT-G-T----------------
	Mycobacterium setense	WP_064871309	---F-M-V--L---T--Q--R	DSVT-G-T----------------
	Mycobacterium sherrisii	WP_069399964	I--Y-T-V--LEEAD---L-R	-AM-LG-T----AE---P------
	Mycobacterium shimoidei	WP_069397106	--FY-M-I--I--DA-EQ--R	DAVVLG-T-----E---P------
	Mycobacterium shinjukuense	WP_083045723	-----V-V----E-E---R-R	--M-L--T----PQ---P------
	Mycobacterium simiae	WP_061556101	--Q-EFLH-PLEEAS--AV-R	DAQRLG-T-----R---P--V---
	Mycobacterium smegmatis	WP_080627956	---L-M-V------T-----R	DSVT-G-T----AE----------
	Mycobacterium szulgai	WP_068033453	--IY-T-V--I---Q-----R	--MT-G-T----PEK---------
	Mycobacterium thermoresistibile	EHI13802	I-M-EFLY-PL--AD--AV-R	DARTLG-T---R-----P--Q---
	Mycobacterium triplex	WP_036466214	I-IY-T-I--IPE-D---L-R	D-MSLG-T----PE----------
	Mycobacterium tuberculosis	WP_070880005	---Y-T-V------E--H--R	A-M--G-T----PQ---P------
	Mycobacterium tusciae	WP_083128110	-------V------T-----R	--MTLG-T----AE------K---
	Mycobacterium ulcerans	WP_011739398	--IY-T-V------E-----R	D-MS-G-T----AEL--P------
	Mycobacterium vaccae	WP_003933741	L----L-D--L--AA---L-L	K-R-L-------------------

Group	Species	Accession	Sequence	Sequence
Other ***Mycobacterium*** (0/>100)	*Mycobacterium vanbaalenii*	WP_011777731	-----L-V----E-T---L-L	Q-R-L-------------------
	Mycobacterium vulneris	WP_065458310	--M-EFLY-PL---S--AV-A	DARTLG-T---RDE---E--D---
	Mycobacterium wolinskyi	WP_067858456	---Y-M-I------T-----Q	--M-LG-T----PE----------
	Mycobacterium xenopi	WP_003922930	-------V------E-E---R	--M-LG-T----PE---P--E---
**Other bacteria**	*Actinomadura oligospora*	WP_084338246	E--Y-R-Y--PSEAALADF-R	H-RRF--T----D----ES-E--E
	Amycolatopsis methanolica	WP_017983205	E-IYEA-I-PLTP-QTEEI-R	NSARLG-T---KA-------Q--E
	Arthrobacter castelli	WP_081416770	-T-YERIF-AL--AT---I--	DYA-LGSA----A-L---G----E
	Gordonia amicalis	WP_006437082	E-M-LLLH-Q-TEDE-EEF-Q	SS-TLG-T----D-----T-KD--
	Gordonia araii	WP_007323230	E-S-QLLH-L-NE-D-EEF-Q	SA-TLG-T----D-----T-KD--
	Nocardia alba	WP_067446684	--TMDLLY-G-NEQD-EQM-R	DAARFG-T--M-AEL--------E
	Nocardia altamirensis	WP_069163928	E-TYQLLQ-R-S--Q-EAF-V	SSSTLG-T---TA-----T--D--
	Nocardia amamiensis	WP_067467731	K-LYERMH-P--EAA--AFHR	L-ARLG-T--M--S------R---
	Rhodococcus erythropolis	WP_019746956	E-S-QLLH-K-SE-Q-ESF-Q	SAS-LG-T---T-E----T--D-E
	Rhodococcus imtechensis	WP_007300640	E--N-ALH-DLSPAA-EFF-K	--ATFG-T----PE---E--I---
	Rhodococcus jostii	WP_073359329	E--N-ALH-DLSPAASAFF-K	--ATFG-T----PE---E-----E
	Saccharomonospora marina	WP_009154583	E--R---V--V---T--EI-A	SSE-LG-T---RPE---------E
	Saccharomonospora paurometabol	WP_007024081	E--Y-A-V--P-PDTT-EL-R	HSA-LG-T---RP----V-----E
	Saccharomonospora viridis	WP_037313055	E----V-V--P-ADT-E-I-A	YSARLG-T---RPE-------E-E
	Tsukamurella pseudospumae	WP_068570203	E-SYQLLH-RLSE---ESF-Q	SAAVL--S----R-----T-GD--
	Yuhushiella deserti	SFQ53970	E-IY-V-G-PTGPDDLE-V-S	ASA-LG-T---RPE----E--T-E

Supplementary Figure 75

A partial sequence alignment of a conserved region of DUF2236 domain-containing protein showing a one amino acid insertion that is specific for members of the "*Terrae"* clade and absent in other bacteria.

Group	Species	Accession	209		255
***"Terrae"* Clade (11/11)**	*Mycobacterium algericum*	WP_083037938	DAVVVCMLAAGGAMNATA	TV	GAGDDDGAWDIEQVAALDIPVFQGLCL
	Mycobacterium arupense	WP_046191236	-----S------------	S-	S-----------RI----M--------
	Mycobacterium heraklionense	WP_064889059	--L---------------	G-	S------------I-------------
	Mycobacterium icosiumassiliensis	WP_067970373	------------------	G-	S-----------RI-------------
	Mycobacterium kumamotonense	WP_065287977	-------------L----	--	S------------I-------------
	Mycobacterium longobardum	WP_085266921	------------------	G-	S------------I-------------
	Mycobacterium minnesotense	WP_083023606	------------------	S-	S-----------RI-------------
	Mycobacterium nonchromogenicum	WP_085136890	-----S------------	S-	S-----------RI----M--------
	Mycobacterium senuense	WP_085087785	------------------	--	------------R--------------
	Mycobacterium sinense	WP_064856667	------------------	--	S-----------KI-------------
	Mycobacterium terrae	WP_085259912	-------------I----	--	S-----------KI-------------
**Other *Mycobacterium* (0/85)**	*Mycobacterium koreense*	WP_085305117	--M--TV-----TKP-G-		A--G--E--NV-HL----V-IL-----
	Mycobacterium triviale	WP_069390870	--M--TV-----TKP-G-		A--G--E--NV-HL----V-IL-----
	Mycobacterium abscessus	WP_074249933	--M--TV------IP-AV		---GA-DE-SVAHL------IL-----
	Mycobacterium africanum	WP_061846054	--M--TV-----VKP-A-		S--G--DS-NV-HL------IL-----
	Mycobacterium alsense	WP_083137933	--M--TV-----LRP---		V--GE-DS-NV-HL------IL-----
	Mycobacterium aromaticivorans	WP_036345388	--M--TV------QP---		---H--EN-NVKHL----V-IL-----
	Mycobacterium arosiense	WP_083064280	--M--TV-----LKP--V		S--G--DS-NV-HL------IL-----
	Mycobacterium aurum	WP_048631853	--MITTV------TP--V		---GV-DS-NVAHL------IL-----
	Mycobacterium avium complex (MAC)	WP_038536139	--M--TV-----LKP---		S--G--DS-NV-HL------IL-----
	Mycobacterium bohemicum	WP_085180971	--M--TV-----LKP-S-		T--G--DS-NV-HL----V-IL-----
	Mycobacterium bovis	WP_024457184	--M--TV-----VKP-A-		S--G--DS-NV-HL------IL-----
	Mycobacterium branderi	WP_083131902	--M--TV-----VKP---		S--G--DS-NV-HL------IL-----
	Mycobacterium canettii	WP_015303367	--M--TV-----VKP-A-		S--G--DS-NV-HL------IL-----
	Mycobacterium celeriflavum	WP_083000570	--LITTV------TP-SV		S--GT-DS--VAHL----V-IL-----
	Mycobacterium chelonae	WP_070916144	--M--TV------TP-SV		---GA-DE-NVGHL------IL-----
	Mycobacterium chimaera	WP_054585334	--M--TV-----LKP---		C--G--DS-NV-HL------IL-----
	Mycobacterium chlorophenolicum	KMO75172	--M-TTV------TP-AV		---GT-D--NVAHL----V-IL-----
	Mycobacterium chubuense	KMO84849	--M-TTV------TP-AV		---GT-D--NVAHL----V-IL-----
	Mycobacterium colombiense	WP_007770884	--M--TV-----LRP-E-		A--GP-DS-NV-HL------IL-----
	Mycobacterium conceptionense	WP_064894278	--L-TTV------TP-VV		----N-DS-NVAHL------IL-----
	Mycobacterium confluentis	WP_085151120	--M--TV------RP---		S--G--DS-NV-HL------IL-----
	Mycobacterium europaeum	WP_085239852	--M--TV-----LKP-AV		S--G--DS-NV-HL------IL-----
	Mycobacterium fallax	ORV06310	--M--TV------RP---		S--G--DS-NV-HL------IL-----
	Mycobacterium farcinogenes	WP_036394047	--L-TTV------TP-AV		---GN-DS-NVAHL------IL-----
	Mycobacterium florentinum	WP_085220369	--M--TV-----LKP--V		S--G--DS-NV-HL----V-IL-----
	Mycobacterium fortuitum	WP_061264617	--L-TTV------TP-AV		---GN-DS-NVAHL------IL-----
	Mycobacterium franklinii	WP_070937110	--M--TV------TP-AV		---GS-DE-SVAHL------IL-----
	Mycobacterium genavense	WP_025736150	--M--TV-----MKP-AV		S--G--DS-NV-HL------IL-----
	Mycobacterium gilvum	WP_011893989	--LITTV------TP-AV		---GV-D--NVAHL----V-IL-----
	Mycobacterium goodii	WP_049748910	--LITTV------TP--V		---GN-DS-NVAHL------IL-----
	Mycobacterium gordonae	WP_065049209	--M--TV-----VKP-A-		S--GE-DS-NV-HL----V-IL-----
	Mycobacterium hassiacum	WP_005627958	-TLITTV------TP-AV		---G--D--NVAHL------IL-----
	Mycobacterium holsaticum	WP_069406175	--M-TTV------TP--V		T--G--DT-NVAHL------IL-----
	Mycobacterium immunogenum	WP_064632621	--M--TV------TP-SV		---GS-DE-NVAHL------IL-----
	Mycobacterium indicus pranii	WP_014941855	--M--TV-----LKP---		S--G--DS-NV-HL------IL-----
	Mycobacterium interjectum	WP_085203589	--M--TV-----LRP---		A--GQ-DS-NV-HL------IL-----
	Mycobacterium intermedium	WP_069420294	--M--TV-----VQP---		S--G--DS-NV-HL------IL-----
	Mycobacterium intracellulare	WP_064938967	--M--TV-----LKP---		S--G--DS-NV-HL------IL-----
	Mycobacterium iranicum	WP_064285337	--L-TTV------TP-AV		---GA-DT-NVAHL------IL-----
	Mycobacterium kansasii	WP_075511752	--M--TV-----VKP---		S--G--DS-SV-HL------IL-----
	Mycobacterium kubicae	WP_085072960	--M--TV-----VKP---		S--G--DS-NV-HL------IL-----
	Mycobacterium kyorinense	WP_065016465	--M--TV------TP-K-		A--GM-DS-NV-HL-G----IL-----
	Mycobacterium lacus	WP_085155768	--M--TV-----LRP---		S--G--DS-NV-HL------IL-----
	Mycobacterium lentiflavum	CQD15552	--M--TV-----LKP--V		S--G--DS-NV-HL------IL-----
	Mycobacterium liflandii	WP_015355698	--M--TV-----VKP---		S--G--DS-NV-HL------IL-----
	Mycobacterium malmesburyense	CRL68906	--LITTV------TPS--		A--G--DG-NVAHL----V-IL-----
	Mycobacterium malmoense	WP_065443139	--M--TV-----LKP--V		S--G--DS-NV-HL---G--IL-----
	Mycobacterium mantenii	WP_083092185	--M--TV-----LKP---		S--G--DS-NV-HL------IL-----
	Mycobacterium marinum	WP_012394727	--M--TV-----VKP---		S--G--DS-NV-HL------IL-----
	Mycobacterium marseillense	WP_083018846	--M--TV-----LKP-A-		S--G--DS-NV-HL------IL-----
	Mycobacterium microti	AMC59712	--M--TV-----VKP-A-		S--G--DS-NV-HL------IL-----
	Mycobacterium mucogenicum	WP_064859100	--LITTV------TP-L-		S--G--DN-NVAHL------IL-----
	Mycobacterium nebraskense	WP_047322915	--M--TV-----LKP--V		S--G--DS-NV-HL---G--IL-----

Group	Species	Accession		
Other *Mycobacterium* (0/85)	*Mycobacterium neoaurum*	CDQ44356	-TLITTV------TP--V	T--GA-DG-NVAHL----V-IL-----
	Mycobacterium neworleansense	CRZ13919	--LITTV------TP-AV	---GN-DS-NVAHL------IL-----
	Mycobacterium noviomagense	WP_083087382	--M--TV-----VTP-K-	A--G--DS-NV-HL------IL-----
	Mycobacterium obuense	WP_046365230	--MITTV------TP-AV	T--GA-DT-NVAHL----V-IL-----
	Mycobacterium palustre	WP_085076093	--M--TV-----LRP--V	---G--DS-NV-HL------IL-----
	Mycobacterium paraffinicum	WP_073873220	--M--TV-----LKP--V	S--G--DS-NV-HL------IL-----
	Mycobacterium paraintracellulare	WP_014384622	--M--TV-----LKP---	S--G--DS-NV-HL------IL-----
	Mycobacterium paraseoulense	WP_083172679	--M--TV-----LKP---	S--G--DS-NV-HL------IL-----
	Mycobacterium parmense	WP_085268715	--M--TV-----LKP-A-	S--G--DS-NV-HL------IL-----
	Mycobacterium peregrinum	WP_064881082	--L-TTV------TP--V	---GN-DS-NVAHL------IL-----
	Mycobacterium porcinum	WP_075920011	--L-TTV------TP--V	---GN-DS-NVAHL------IL-----
	Mycobacterium rhodesiae	WP_083120489	--M--TV------RP---	---H--DN-NVKHL----V-IL-----
	Mycobacterium rufum	KGI70947	--M-TTV------TP-AV	S--GA-DT-NVAHL----V-IL-----
	Mycobacterium rutilum	SEH76496	--LITTV------TP--V	T--G--DT-NVAHL------IL-----
	Mycobacterium salmoniphilum	WP_078329099	--M--TV------TP-AV	---GA-DE-NVGHL------IL-----
	Mycobacterium saopaulense	WP_070910835	--M--TV------TP-S-	---GA-DE-NVAHL------IL-----
	Mycobacterium saskatchewanense	WP_085257373	--M--TV-----LKP---	S--GE-DS-NV-HL------TL-----
	Mycobacterium scrofulaceum	WP_067278864	--M--TV-----LKP---	S--G--DS-NV-HL------IL-----
	Mycobacterium septicum	WP_044523977	-VLITTV------TP-AV	---GN-DS-NVAHL------IL-----
	Mycobacterium setense	WP_064872932	--L-TTV------TP-AV	---GN-DS-NVAHL------IL-----
	Mycobacterium sherrisii	WP_069401853	--M--TV-----LQP---	S--G--DS-NV-HL------IL-----
	Mycobacterium simiae	WP_061555939	--M--TV-----LKP---	S--G--DS-NV-HL------IL-----
	Mycobacterium smegmatis	WP_058126412	--LITTV------TP-AV	---G--DS-NVAHL------IL-----
	Mycobacterium szulgai	WP_085671137	--M--TV-----VKP---	S--G--DS-NV-HL------IL-----
	Mycobacterium triplex	WP_036469655	--M--TV-----MKP--V	S--G--DS-NV-HL------IL-----
	Mycobacterium tuberculosis	WP_063741636	--M--TV-----VKP-A-	S--G--DS-NV-HL------IL-----
	Mycobacterium tusciae	WP_006245264	--L-TTV-----STP---	S--GS-DT-NVAHL----V-IL-----
	Mycobacterium ulcerans	WP_011740275	--M--TV-----VKP---	S--G--DS-NV-HL------IL-----
	Mycobacterium vaccae	WP_003933496	--L-TTV------TP-AV	---GA-D--NVAHL------IL-----
	Mycobacterium vulneris	WP_065458551	--L-TTV------TP-AV	---GN-DS-NVAHL------IL-----
	Mycobacterium wolinskyi	WP_067852835	--LITTV-----STP--V	---GA-DS-NVAHL----V-IL-----
	Mycobacterium yongonense	WP_065503780	--M--TV-----LKP---	S--G--DS-NV-HL------IL-----
Other bacteria	*Amycolatopsis lurida NRRL 2430*	AJK59008	--LL-TV-----TRPSEV	---G--E---VAEM------IL-A---
	Blastococcus saxobsidens	WP_014375102	--M--TV------RP---	Q--G------VGEL----V--I-----
	Corynebacterium aquilae	WP_075726130	-VAITTV-----TKP---	SG-G--E---VAAL------II---A-
	Dietzia alimentaria	WP_010542202	--L--TV-----TTPGA-	---GN-ET----RI-------L-----
	Geodermatophilus poikilotrophus	SET51259	--L--TV-----SRP---	Q--G------VGAL-S--V--V-----
	Gordonia hydrophobica	WP_066163356	-----TV-----STP-A-	S--Q--E---VARM-------L-----
	Hoyosella subflava	WP_013806844	--I-ATV-----TKP---	---G--E---V-AL------IL-----
	Janibacter terrae	WP_068325094	--L-TTV-----TKP---	---E--E---V-KL------IL-----
	Kibdelosporangium aridum	WP_084430381	--L--TV-----TKP---	S--G--E---VGSL----V-IL-----
	Lentzea flaviverrucosa	SES50327	--L--TV-----TNP-K-	S--G--E---VGAL----V-IL-----
	Nocardia arizonensis	WP_054813130	--L--TV------RP---	A--G--E---VAAL-E--V-IL-----
	Nocardioides insulae	WP_051218495	--L--TV--S---TP-A-	S--E--DS--V-RI-------L-----
	Pseudonocardia asaccharolytica	WP_028929729	--MI-TV-----TRP-A-	T--G--EE--VAAL------IL-----
	Rhodococcus koreensis	WP_072950556	--M--TV-----TKP-G-	S--G--E---VAAL----V-IL-----
	Saccharopolyspora erythraea	WP_009943736	--L--TV-----SKP---	Q--G--E---VGAL-E----IL-----
	Streptomyces oceani	WP_070198306	--L--TV------QP---	S--G--E---V-RLR------L-A-T-
	Streptosporangium canum	SFK63044	--L--TV-----TRP---	S--G--E---VGAL-E--V-IL-----
	Thermocrispum municipale	WP_028849103	-----TV-----SVP-SV	S--G--ET--V-RI-------L-----
	Williamsia sterculiae	WP_076478317	--L--TV-----TTP---	S--EQ-EN-N--RL------IL-----

Supplementary Figure 76

A partial sequence alignment of a conserved region of cobaltochelatase subunit CobN showing a two amino acid insertion that is specific for members of the "*Terrae*" clade and absent in other bacteria.

			232		276
**"Terrae" Clade (13/13)**	*Mycobacterium algericum*	WP_083040154	QRWAVRSQLRSEGRRFMKALDLR	T	LNIPLLHLRGDDDPYVLADPV
	Mycobacterium arupense	WP_046191396	----------T------------	-	VTT------------------
	Mycobacterium engbaekii	WP_085128713	----------A------------	-	-G--------EH-----T---
	Mycobacterium heraklionense	WP_064998300	----------A------------	-	-K--------E----------
	Mycobacterium hiberniae	WP_085134086	----------A------------	-	-G--------EH-----T---
	Mycobacterium icosiumassiliensis	WP_067976558	--------F-A------------	-	----------E----------
	Mycobacterium kumamotonense	WP_065289536	---------------------V-	-	-T--------EN---------
	Mycobacterium longobardum	WP_085265477	----------A------------	-	----------EE---------
	Mycobacterium minnesotense	WP_083026105	----------A------------	-	VTT------------------
	Mycobacterium nonchromogenicum	WP_085139305	----------A------------	-	-K--------E----------
	Mycobacterium senuense	WP_085085620	-----------------------	-	---------------------
	Mycobacterium sinense	WP_064856022	-----------------------	-	-D--------A----------
	Mycobacterium terrae	WP_085260323	-----------------------	-	-T-----V--A----------
**Other *Mycobacterium* (0/>100)**	*Mycobacterium koreense*	WP_085303756	------T---D------A-M-GQ		ITV-M------T---------
	Mycobacterium triviale	WP_069393112	------T---D------A-M-GQ		IPV-M------T---------
	Mycobacterium abscessus	WP_062878549	-------------W---RSMNRE		-SV-V--M--ES---------
	Mycobacterium alsense	WP_083138406	----------G-----IRSMSQ-		-TM-V------A-----S---
	Mycobacterium angelicum	WP_083115848	----------G------GSMIKQ		-G--V------A------AA-
	Mycobacterium aromaticivorans	WP_036341836	-------------W----LMNRP		-VV--------A---------
	Mycobacterium arosiense	WP_083063971	----------D------RSMSQQ		-SV--------A---------
	Mycobacterium asiaticum	WP_065036336	-------------H--I-STTQQ		-GV----I---V------A--
	Mycobacterium aurum	WP_048633963	----A-----G------RSMKGP		-GV-V-----EA---------
	Mycobacterium avium	WP_062886583	----------D-----LRSMRRQ		-GV-------EE---------
	Mycobacterium avium subsp. avium	ETB13390	----------D-----LRSMRRQ		-GV-------EE---------
	Mycobacterium avium subsp. hominissuis	ETB36012	----------D-----LRSMRRQ		-GV-------EE---------
	Mycobacterium avium subsp. paratuberculosis	ETB47874	----------D-----LRSMRRQ		-GV-------EE---------
	Mycobacterium bacteremicum	WP_083061952	--------I-G------HSMARP		ITV-----H-TV---------
	Mycobacterium boenickei	WP_077738676	-------------L---RSMKRP		ISV-V--M---A-----T---
	Mycobacterium bohemicum	WP_085182334	----A-----D--H--IRSVSRQ		-GM----V---E---------
	Mycobacterium bovis BCG	AMC52930	----------------IR-MTQQ		-GM--------A---------
	Mycobacterium branderi	WP_083131209	----------D------RSMTR-		AG--V--I---A---------
	Mycobacterium brisbanense	WP_062828412	----------G------RSMKRP		V-V-V------A---------
	Mycobacterium canariasense	WP_062657851	----------G------RSMKQP		-A--V--M---A---------
	Mycobacterium canettii	WP_015291635	----------G-----IR-MTQQ		-GM--------A---------
	Mycobacterium celatum	WP_062538414	----------D------RSMNR-		AG--V--I---A---------
	Mycobacterium celeriflavum	WP_083000287	----------G------RSMKRP		I-V-V------A---------
	Mycobacterium chelonae	WP_070916767	-------------W---RSMNRE		-SV-V--M---S---------
	Mycobacterium chimaera	WP_074021424	----A-----D------RSMCQ-		-SV-------EA---------
	Mycobacterium chlorophenolicum	WP_048471477	----------G------RSMKRP		ISV-V------A---------
	Mycobacterium chubuense	WP_014817948	--------M-AD-----QSMKRQ		IPV-V------A---------
	Mycobacterium colombiense	WP_064885286	----A-----D------RSMSQQ		-SV-------EA---------
	Mycobacterium conceptionense	WP_064898554	-------------L---RSMKRP		I-V-V--M---A---------
	Mycobacterium confluentis	WP_085150268	--------F--------Q-M-KP		-D--M------A---------
	Mycobacterium conspicuum	WP_085232713	----L-----D------RSMSE-		-A--V------A---------
	Mycobacterium cosmeticum	WP_036397712	----------G------RSMKQP		-A--V--M---A---------
	Mycobacterium diernhoferi	WP_073859309	----------A------QSMKRP		-T--------GS---------
	Mycobacterium doricum	WP_085188283	----------G------RLMKRP		-VV-M-----GS---------
	Mycobacterium europaeum	WP_085241389	----------D-------SMCQ-		-SV--------A-------A-
	Mycobacterium fallax	WP_085094991	E----------------R-M-RP		-T--V---H-AA-------A-
	Mycobacterium farcinogenes	WP_036391349	-------------L---RSMKRP		I-V-V--M---A---------
	Mycobacterium flavescens	WP_069415324	----------G------RLMKRP		IGV-V--M--EH---------
	Mycobacterium florentinum	WP_085223157	----------G-------SMSRQ		-GV--------A---------
	Mycobacterium fortuitum	WP_061265026	----------G------RSMKRP		I-V-V--M---A---------
	Mycobacterium fragae	WP_085200150	----------D------RSMSRP		-G--M--I--EA---------
	Mycobacterium franklinii	WP_070937337	-------------W---RSMNRE		-S--V--M--EA---------
	Mycobacterium gastri	WP_036413752	----------G-------SMRRQ		-GV--------A---------
	Mycobacterium genavense	WP_036467616	----------G--------MSRQ		-GV--------A---------
	Mycobacterium gilvum	WP_011892275	----------A------RSMRAP		-SV-M------A-----P---
	Mycobacterium goodii	WP_049743446	----------G------ASMKRP		V-V-V------E-----P---
	Mycobacterium gordonae	WP_065044299	----------G--H--I-STTSQ		-GV----I---A------A--
	Mycobacterium haemophilum	WP_047316669	----------N------RSMSRQ		-SV----V---A---L---S-
	Mycobacterium hassiacum	WP_005630804	--------F-L--H---RLMRR-		ID--V------A---------
	Mycobacterium heckeshornense	WP_048891481	----------D-------SMSRQ		-G--V------A---------
	Mycobacterium heidelbergense	WP_083073774	----------G------RSMCQ-		-GT-------EA---------

**Other *Mycobacterium* (0/>100)**	*Mycobacterium holsaticum*	WP_069405263	----------G------RSMRRP	I-V-V------A-------S-
	Mycobacterium houstonense	WP_066902351	----------G------RSMKRP	IGV-V--M---L-----P---
	Mycobacterium immunogenum	WP_064631833	-------------W---RSMNRE	-SV-V--M--ES---------
	Mycobacterium insubricum	WP_083029244	---------QP------R-M-QP	-GV-S-----AQ-------A-
	Mycobacterium interjectum	WP_066916617	----------G------RSMSQQ	-GM--------A---I--H--
	Mycobacterium intermedium	WP_069418013	----------G------RSMIQQ	-GV-V------A------GA-
	Mycobacterium intracellulare	WP_064938652	----A-----D------RSMCQQ	-SV-------EA---------
	Mycobacterium iranicum	WP_064279812	-----------------RSMRRP	-AV-V------A---------
	Mycobacterium kansasii	WP_063469280	----------G-------SMRRQ	-GV--------A---------
	Mycobacterium komanii	CRL74074	----------G------RSMKRP	I-V-V--M---A---------
	Mycobacterium kubicae	WP_085074673	----------A-------SMTQQ	-SM--------A------A--
	Mycobacterium kyorinense	WP_065014409	----------D------RSMSRG	T-V----I--GA---------
	Mycobacterium lacus	WP_085160960	----------G--H---RSMTQQ	-GV--------A------A--
	Mycobacterium lentiflavum	CQD21935	----------G-------SMSRQ	-GV--------A---------
	Mycobacterium leprae	WP_010908812	----------N---K---SMSRP	FSV----V---A---L---S-
	Mycobacterium lepromatosis	WP_045843725	----------N-------SMSRP	FSV----V--NA---L---S-
	Mycobacterium litorale	WP_078021236	----------G--W----SM-RQ	-DV--------A---------
	Mycobacterium llatzerense	WP_043985072	----A---V-G------R-MKQ-	TPV-I---H-TA-----D---
	Mycobacterium malmesburyense	CRL72767	----------G------RSMKRP	I-V-V--M---A---------
	Mycobacterium malmoense	WP_065443461	----------D-------SMSQ-	-SV--------A---------
	Mycobacterium mantenii	WP_083098931	----------D------RSMSQQ	-SV-------EA---------
	Mycobacterium marinum	WP_012396675	----------G--H---RSMNQ-	PGV-I------A------A--
	Mycobacterium marseillense	WP_083019084	----A-----D------GSMCQQ	-SV-------EA---------
	Mycobacterium monacense	WP_083044874	----------G------RLMKRP	-V--M------S---------
	Mycobacterium moriokaense	WP_083155290	--------F-G------RSMRRP	I-V-V------A---------
	Mycobacterium mucogenicum	WP_064857700	----A---V-G------R-MKQ-	TAV-V---H-TA-----D---
	Mycobacterium nebraskense	WP_046186690	----------D-------SMCQQ	-SV--------A---------
	Mycobacterium neoaurum	WP_030132816	--------V-G------RSMARP	IDV-------AA---------
	Mycobacterium neworleansense	CRZ17806	-------------L---RSMKRP	ISV-V--M---A-----T---
	Mycobacterium noviomagense	WP_083089283	----------D------RSMKRP	AG--I------A---------
	Mycobacterium novocastrense	WP_067394184	----------G------RTMKRP	ISV-V--M--VA---------
	Mycobacterium obuense	WP_046365134	-----------------RSMKRP	IAV-V------S---------
	Mycobacterium palustre	WP_085076663	----------G--Q---RSMSQQ	-AM--------A---------
	Mycobacterium paraense	WP_085103406	----------G------RSMSQQ	-GM--------A---------
	Mycobacterium paraffinicum	WP_073871529	----------D-------SMSQQ	-SV-------EA---------
	Mycobacterium parafortuitum	WP_083142072	--------M-T------RSMRRP	-AV-M------T-----P---
	Mycobacterium paraseoulense	WP_083173461	----------D-------SMCQQ	-SV--------A---------
	Mycobacterium parmense	WP_085269136	----------G------GSMSQQ	-SV--------A---------
	Mycobacterium peregrinum	WP_064881177	----------G------RSMKRP	IGV-V--M---A---------
	Mycobacterium phlei	WP_040634302	----------A------RLMQRP	I-L-V------R---------
	Mycobacterium porcinum	WP_069424674	-------------L---RSMKRP	ISV-V--M---E-----T---
	Mycobacterium rhodesiae	WP_014210552	--------F-A---K--RSMRRP	I-V-V------A---------
	Mycobacterium riyadhense	WP_085251799	----------G------RSMIAQ	-G---------L------VA-
	Mycobacterium rufum	KGI70106	----------G------RSMKRP	ISV-V------A---------
	Mycobacterium rutilum	WP_083406931	----------G------RSMKRP	I-V-V------A---------
	Mycobacterium salmoniphilum	WP_078324430	-------------W---RSMNRE	-SV-V--M---T---------
	Mycobacterium saopaulense	WP_083019469	-------------W---RSMNRE	-SV-M-----EA---------
	Mycobacterium saskatchewanense	WP_085255236	----------D-------SISQQ	-GL-------E----------
	Mycobacterium scrofulaceum	WP_067270717	----------D-------SMSQQ	-SV-------EA---------
	Mycobacterium setense	WP_064871462	----------G--L---RSMKRP	IGV-V--M---A---------
	Mycobacterium sherrisii	WP_069400761	----------G-------SVSRQ	-GV--------G-------SI
	Mycobacterium shimoidei	WP_069394693	----------D------R-MNAN	I-V----I---A---------
	Mycobacterium shinjukuense	WP_083048314	----------D-------SMNQ-	PG--V------A--F---H--
	Mycobacterium simiae	WP_061557430	------------------SVSRQ	-GV--------V--------I
	Mycobacterium smegmatis	WP_011731100	----------G------ASMRRP	V-V-V------E-----P---
	Mycobacterium szulgai	WP_068034492	----------A-------SMTQQ	-SM--------A------A--
	Mycobacterium thermoresistibile	WP_040546646	-------------H---RLMTRP	VRV-V---H-ES---------
	Mycobacterium timonense	WP_083186938	----------D-----LRSMRRQ	-GV-------EE---------
	Mycobacterium triplex	WP_036465132	----------G--------MSRQ	-GV--------A---------
	Mycobacterium tuberculosis	SGL66279	----------------IR-MTQQ	-GM--------A---------
	Mycobacterium tusciae	WP_083126258	--------F-G------RSMRRP	I-V-V------A---------
	Mycobacterium ulcerans	WP_011741875	----------R--H---RSMNQ-	PGV-I------A------A--
	Mycobacterium vaccae	WP_003931724	--------F-A------RSMKRP	-AV-V------A---------
	Mycobacterium vulneris	WP_065462819	-------------L---RSMKRP	ISV-V--M---E-----T---
	Mycobacterium wolinskyi	WP_085143199	----------G------RSMKRP	V-V-V------A---------

Group	Species	Accession	Sequence	Sequence
Other *Mycobacterium* (0/>100)	*Mycobacterium xenopi*	WP_003921620	----------D-------SMSRQ	-G--V------A---------
	Mycobacterium yongonense	WP_065501259	----A-----D------RSMCQ-	-SV-------EA---------
Other bacteria	*Amycolatopsis decaplanina*	WP_007028453	Y---F-A-F-G-----SE--RG-	FAPRV-Q-H-EE-RC--P-
	Dietzia alimentaria	WP_029457584	R--M------PD--D-RR-VSGV	-DQ-V-GIS-AE-RFI-PET-
	Frankia inefficax	WP_013421540	H--VF--LF-PD-A--AQ--RRA	V-C-V---H-GA--FL-P-
	Gordonia neofelifaecis	WP_009679444	R---F---F-ND-W---DLM-E-	-EV---AV--H--A-I-
	Hoyosella subflava	WP_013805082	H---F---F-P-----F--MNRV	VHV-V-QIH-SA--FI--
	Lentzea guizhouensis	WP_065921022	Y---------GD----AE-V-KP	-LM-V-QVH-EL-TVM-
	Millisia brevis	WP_066907731	----F--RF-PD-H--LSRM-RQ	IDG-A---H-L---L--
	Nocardia brevicatena	WP_040832964	----F---W-PD-H---AIMRRP	ID--V-SV--AL-R-----T-
	Pseudonocardia asaccharolytica	WP_037057700	Y---L-----A-----AA-VAR-	ASM-V-QVH-A---FL-
	Rhodococcus defluvii	WP_031939311	----F---F-PD-H---A-M-QQ	VE--V-QIH-AL------RT-
	Saccharomonospora cyanea	WP_005459898	Y---F---F-GD----AE-VSA-	VTM-V-QVH-----C-PE
	Segniliparus rugosus	WP_007468114	----F---F-PD----LRQMRCP	-P--G-L---GH-----QR-M
	Skermania piniformis	WP_066466944	----F---W-AD-----ST-RIP	-DL-V-QIH-EL---IR-E
	Streptacidiphilus neutrinimicus	WP_042373444	Y--LM--MG-PD-FQ-ARRM-RK	-RV-T--VH-EN--VL-PE
	Thermocrispum municipale	WP_028849772	Y---F--LF-GD----AA-MRDP	SPA-V-Q-H-RV-GC--
	Tsukamurella paurometabola	WP_013128493	A---W---F-PD-T--LSRMNQ-	-H--V-AF--ES-----PQ--
	Williamsia herbipolensis	WP_045821493	R---F---F-RD-AK--RLMNR-	----V-AA--R--R-IH-E

Supplementary Figure 77

A partial sequence alignment of a conserved region of alpha/beta hydrolase showing a one amino acid insertion that is specific for members of the "*Terrae"* clade and absent in other bacteria.

			141		180
***"Terrae"* Clade (11/11)**	*Mycobacterium algericum*	WP_083035846	RWRRRVKDHTIVIGYGTKGKTAIAAMRGDD	E	SPPKDIVVV
	Mycobacterium engbaekii	WP_085129320	------------------L---	-	TT-S-----
	Mycobacterium heraklionense	WP_065038803	---K----------------------L---	-	TT-S-----
	Mycobacterium hiberniae	WP_085133949	------------------L---	-	TT-S-----
	Mycobacterium icosiumassiliensis	WP_067970805	---K----------------------L---	-	TT-S-----
	Mycobacterium kumamotonense	WP_065287466	------------------L---	-	TT-S-----
	Mycobacterium longobardum	WP_085264335	---KQ---------------------V--Q	-	TT-S-----
	Mycobacterium minnesotense	WP_083027541	---K--R-------------------L---	T	TT-S-----
	Mycobacterium senuense	WP_085081736	------------------------------	-	---------
	Mycobacterium sinense	WP_064855420	---K--R-------------------L---	-	TT-S-----
	Mycobacterium terrae	WP_085261972	---K--R-------------------L---	-	TTLG-----
**Other *Mycobacterium* (0/>100)**	*Mycobacterium triviale*	WP_085110762	---KK-RN----V---------VV--V---		V-TSS----
	Mycobacterium abscessus	WP_065203991	---A--RN--V-V---------VQ--LS-G		AA-SE----
	Mycobacterium abscessus subsp. bolletii	EHM16775	---A--RN--V-V---------VQ--LS-G		AA-SE----
	Mycobacterium africanum	KBF94486	---S--RN--V-----------V---VS-E		LV-GE----
	Mycobacterium alsense	WP_083140580	---ST-RN--------------VG-LL---		V-AAE----
	Mycobacterium angelicum	WP_083111078	---SK-RN--------------V---L--A		VT-GE----
	Mycobacterium aromaticivorans	WP_036340102	---NS-RN--V--------R--V---I--G		VA-A-----
	Mycobacterium asiaticum	WP_065035316	---S--RN--V---------------LS-E		MT-GE----
	Mycobacterium aurum	WP_048630383	---S-LRN-I-IV------R--A---V--E		VA-G-----
	Mycobacterium avium	WP_073578921	---S--RN--------------V--IL--E		TTQAEV--
	Mycobacterium avium subsp. paratuberculosis	AAS05849	---S--RN--------------V--IL--E		TTQAEV---
	Mycobacterium bacteremicum	WP_083057301	---KS-RN--V-V------R--V---I---		VA-A-----
	Mycobacterium boenickei	WP_077743599	---NK-RN--V-V------R--V---V--E		VS-A-----
	Mycobacterium bovis	WP_024456818	---S--RN--V-----------V---VS-E		LV-GE----
	Mycobacterium branderi	WP_083129866	---S--RN--V-V---------VT--LS-E		V-AG-----
	Mycobacterium canariasense	WP_062654524	----S-RN----V------R--VS--I---		VA-G-----
	Mycobacterium canettii	WP_014001618	---S--RN--V-----------V---VS-E		LV-GE----
	Mycobacterium celatum	WP_062540729	---S--RN--V-----------V---L--E		V-AGE----
	Mycobacterium chelonae group	WP_030096957	---S--RN--V-V---------VQ--LS-G		AT-AE----
	Mycobacterium chlorophenolicum	WP_048471354	---N--RN-I-IV------R--A---V--E		VA-A-----
	Mycobacterium chubuense	WP_014814702	---S--RN-I-IV------R--A---V--E		VA-A-----
	Mycobacterium colombiense	WP_065027100	---S--RN--------------V--IL---		TVQTEV---
	Mycobacterium conceptionense	WP_064895202	---NK-RN--V-V------R--V---V--E		VS-A-----
	Mycobacterium confluentis	WP_085148661	---NT-RN--------------V---I--G		V--GE----
	Mycobacterium conspicuum	WP_085231388	---S--RN--V-----------V--RLA-E		EAQGE----
	Mycobacterium diernhoferi	WP_073857800	---KS-RN--V-V------R--V---I---		VA-A-----
	Mycobacterium doricum	WP_085190051	Q--NKLRN--V--------R--V---V--E		VA-A-----
	Mycobacterium europaeum	WP_085241610	---S--RN--------------V--ILS-E		AAQG-V---
	Mycobacterium fallax	WP_085095119	----S-RK----V-------------VS-G		V--AE----
	Mycobacterium flavescens	WP_069415961	---NK-RN-IVIV------R--A---V--E		IA-A-----
	Mycobacterium florentinum	WP_085223095	---N--RN------------R-V--IAS-E		AAQGE----
	Mycobacterium fortuitum	WP_054601720	---NK-RN--V-V------R--V---V--E		VS-A-----
	Mycobacterium franklinii	WP_070938750	---A--RN--V-V---------VQ--LS-G		AA-SE----
	Mycobacterium gastri	WP_036412738	---N--RN--V-V----------T--L--E		VV-GE----
	Mycobacterium genavense	WP_025735726	---N--RN------------R-V--RVS--		AAQGE----
	Mycobacterium gilvum	WP_011895499	---S-LRN-I-IV------R--A---V--E		IA-G-----
	Mycobacterium goodii	WP_049747775	---SK-RN--V--------R--V---V---		VA-A-----
	Mycobacterium gordonae	WP_065047239	---S--RN--V------------S--LS-E		V--GE----
	Mycobacterium haemophilum	WP_054880507	---NK-RN--------------V--RLS-E		AAQGEM---
	Mycobacterium hassiacum	WP_005625941	---S--RN-IVIV---------A--LV--E		VA-A-----
	Mycobacterium heckeshornense	WP_048891248	---S--RN--V-----------V---LS-E		-A-T-----
	Mycobacterium heidelbergense	WP_083073131	---SK-RN--------------V--IL--E		EARGEV-I-
	Mycobacterium holsaticum	WP_069403965	---NK-RN-IVIV------R--A---V--E		VA-A-----
	Mycobacterium houstonense	WP_066903394	---NK-RN--V-V------R--V---V--E		VS-A-----
	Mycobacterium immunogenum	WP_064627998	---A--RN--V-V---------VQ--LS-G		AA-AE----
	Mycobacterium insubricum	WP_083030747	---KS-RN----V---------VT--I--G		T--G-----
	Mycobacterium interjectum	WP_085201862	---SK-RN--------------V--IL--E		EAQGE----
	Mycobacterium intermedium	WP_069419048	---SK-RN--V---------------L--E		AV-G-----
	Mycobacterium intracellulare	WP_064938507	---S--RN--------------V--IL--E		TIQA-V---
	Mycobacterium iranicum	WP_064283187	---N-LRN-I-IV------R--A---V--E		VA-A-----
	Mycobacterium kansasii	WP_063468886	---N--RN--V------------T--L--E		VV-GE----
	Mycobacterium komanii	CRL78159	---S--RN--V--------R--V---V--E		VA-A-----
	Mycobacterium kyorinense	WP_065016596	---K--RN----T---------V------G		IAAG-----
	Mycobacterium lacus	WP_085161115	---NK-RN--V-----------VD-IL--Q		AA-G-----
	Mycobacterium lentiflavum	CQD08448	---N--RN------------R-V--RVS-E		AVQGE----
	Mycobacterium litorale	WP_078020426	---NS-RN--V--------R--V---I--G		VA-A-----
	Mycobacterium llatzerense	WP_043985261	---SS-RN----V------R--VQ--V--E		VA-G-----
	Mycobacterium mageritense	WP_036428303	---S--RN--V--------R--V---V--E		VA-A-----
	Mycobacterium malmesburyense	CRL67050	---S--RN--V--------R--V---V--E		VA-A-----

Group	Species	Accession	Sequence	
Other *Mycobacterium* (0/>100)	*Mycobacterium malmoense*	WP_065445022	---S--RN--------------V--IL--E	AAQG-V---
	Mycobacterium mantenii	WP_083096141	---S--RN--------------V--ILS-E	AAQGEV---
	Mycobacterium marinum	WP_036457398	---S--RN--V------------S--L--E	AV-GE----
	Mycobacterium marseillense	WP_083020082	---SK-RN--------------V---L--E	VV-GE----
	Mycobacterium moriokaense	WP_083154165	---NK-RN-I-IV------R--A---V--E	VA-A-----
	Mycobacterium mucogenicum	WP_060999137	---ST-RN----V------R--VQ--V--E	VA-G-----
	Mycobacterium nebraskense	WP_046187009	---NK-RN--------------V--IL--E	AAQG-V---
	Mycobacterium neworleansense	CRZ15557	---NK-RN--V-V------R--V---V--E	VS-A-----
	Mycobacterium noviomagense	WP_083086267	---S--RN--V-----------V---LS-E	AA-S-----
	Mycobacterium obuense	WP_046365201	---S--RN-I-IV------R--A---V--E	VA-A-----
	Mycobacterium palustre	WP_085077989	---SK-RN--V-----------V--IL--E	AAQGEV---
	Mycobacterium paraense	WP_085103840	---SK-RN--V-----------V--IL--E	EAQGEV---
	Mycobacterium paraffinicum	WP_073876242	---G--RN--------------V--IL--E	AAQGEV---
	Mycobacterium parafortuitum	WP_083143062	---S-LRN-I-IV------R--A---V--E	IA-G-----
	Mycobacterium parascrofulaceum	WP_007168161	---S--RN--------------V--IL--E	AAQG-V---
	Mycobacterium paraseoulense	WP_083170033	---S--RN--------------V--IL--E	AAQG-V---
	Mycobacterium parmense	WP_085267697	---S--RN--------------V--IV--E	ASQGE----
	Mycobacterium peregrinum	WP_064888260	---NK-RN--V--------R--V---V--E	VS-A-----
	Mycobacterium phlei	WP_040634497	---S--RN--V--------R--V---I--E	VA-A-----
	Mycobacterium porcinum	WP_075921429	---NK-RN--V-V------R--V---V--E	VS-A-----
	Mycobacterium rhodesiae	WP_083121074	---NT-RN--V--------R--V---I--G	VA-A-----
	Mycobacterium riyadhense	WP_085252028	---S--RN--------------V---L--E	AV-GE----
	Mycobacterium rufum	KGI67395	---NKLRN-I-IV------R--A---V--E	VA-A-----
	Mycobacterium rutilum	WP_083409856	---N--RN-IVIV------R--A---V--E	IA-A-----
	Mycobacterium salmoniphilum	WP_078323789	---A--RN--V-V---------VQ--LS-G	AT-AE----
	Mycobacterium saopaulense	WP_070909602	---A--RN--V-V---------VQ--LS-G	AA-AE----
	Mycobacterium saskatchewanense	WP_085257930	---S--RN--------------V--IL--E	AAQGE----
	Mycobacterium scrofulaceum	WP_067277574	---S--RN--------------V--IL--E	AAQGEV---
	Mycobacterium septicum	WP_044516799	---NK-RN--V-V------R--V---V--E	VS-A-----
	Mycobacterium setense	WP_039317703	---NK-RN--V-V------R--V---V--E	VS-A-----
	Mycobacterium sherrisii	WP_069402481	---S--RN--------------V---LA-E	V--GE----
	Mycobacterium shimoidei	WP_069396552	---S--RN--V------------T--IS-E	V--A-----
	Mycobacterium shinjukuense	WP_083049866	---SK-RN--V-----------V---L--E	VV-GE----
	Mycobacterium simiae	WP_044510130	---S--RN--------------V---LA--	V--GE----
	Mycobacterium smegmatis	WP_003893325	---SK-RN--V--------R--V---V---	VA-A-----
	Mycobacterium szulgai	WP_085674290	---SK-RN--------------V---L--A	VT-GE----
	Mycobacterium thermoresistibile	WP_040548599	---S--RN--V-----------V---VE-E	VA-A-----
	Mycobacterium triplex	WP_036467386	---N--RN------------R-V--RVS-E	AAQGE----
	Mycobacterium tuberculosis	WP_061318592	---S--RN--V-----------V---VS-E	LV-GE----
	Mycobacterium tusciae	WP_083125063	Q--NKLRN--V--------R--V---V--E	IA-A-----
	Mycobacterium ulcerans	WP_011740477	---S--RN--V------------S--L--E	AV-GE----
	Mycobacterium vaccae	WP_040540351	-----LRN-I-IV------R--A---V--E	IA-G-----
	Mycobacterium vulneris	WP_065516741	---NK-RN--V-V------R--V---V--E	VS-A-----
	Mycobacterium wolinskyi	WP_067854423	---S--RN--V--------R--V---V--E	VA-A-----
	Mycobacterium xenopi	WP_003920816	---S--RN--V-----------V---LS-E	VA-S-----
	Mycobacterium yongonense	WP_065498515	---S--RN--------------V--IL--E	TIQA-V---
Other *Corynebacteriales*	*Corynebacterium afermentans*	WP_063938601	Q--NS-RN-----------RS--D-LIAGG	AS-SS---I
	Dietzia timorensis	WP_067474872	---TTLRN--V-V------RS-V--LLA-E	ISASE----
	Gordonia aichiensis	WP_005170776	---NN-RN--V--------R--VD--I--G	IK-SE----
	Hoyosella subflava	WP_013808534	---QQ-RN----V------R--VD--I--G	VR-SE----
	Millisia brevis	WP_066913260	----S-RN----V------RS-VD-LL--G	AS-SE----
	Nocardia higoensis	WP_040797867	----G-R---V-V------R---D--L--G	VA-T-----
	Rhodococcus fascians	ORI18441	---HK-RN--V---F----R--VD--L--G	V-ASE----
	Segniliparus rotundus	WP_049773351	---HK-R--N--V------RS-V---LA-G	VE-NR----
	Segniliparus rugosus	WP_007470192	---HA-R-----V------RS-V---LA-G	VE-TR----
	Sinosporangium album	SDI26258	---S-LRN----V------RA--RTLLENE	RTKET----
	Skermania piniformis	WP_066467798	----T-RN--V-V------RS-VD-LL--G	VQ-A-----
	Smaragdicoccus niigatensis	WP_018163222	Q---K-RN--V---F----R--VN--MS--	V-ADE---
	Tsukamurella paurometabola	WP_013125807	---SA-HN----V------R--VS--IN-G	IE-SK----
	Tsukamurella pseudospumae	WP_068571772	---SA-HN----V------R--VS--IN-G	ME-GK----
	Williamsia herbipolensis	WP_045823565	---SK-RN--V-V---------VN--I--G	TK-SE----
	Williamsia sterculiae	WP_076479735	---SN-RN--V-V-F-------VS-LMA-G	VK-AE----

Supplementary Figure 78

A partial sequence alignment of a conserved region of potassium transporter Kef showing a one amino acid insertion that is specific for members of the "*Terrae"* clade and absent in other *Corynebacteriales*.

Group	Species	Accession	330		357
"Terrae" Clade (13/13)	*Mycobacterium algericum*	WP_083040227	LYRDVGFVETGVRKRYYR		NGADAYMMRR
	Mycobacterium arupense	WP_046189127	------------------		------T---
	Mycobacterium engbaekii	WP_085130090	--------T---------		------T-S-
	Mycobacterium heraklionense	WP_064887667	-----------L------		------T---
	Mycobacterium hiberniae	WP_085135187	-------AQ----R----		------T---
	Mycobacterium icosiumassiliensis	WP_067970160	-----------L------		------T---
	Mycobacterium kumamotonense	WP_065287304	-----------L------		----------
	Mycobacterium longobardum	WP_085263254	---------V-L------		------T---
	Mycobacterium minnesotense	WP_083026793	-----------L------		------T---
	Mycobacterium nonchromogenicum	WP_085139148	-----------L------		------T---
	Mycobacterium senuense	WP_085083189	---E--------------		----------
	Mycobacterium sinense	WP_064853568	---A-------L------		----------
	Mycobacterium terrae	WP_085261376	-----------L------		----------
Other *Mycobacterium* (0/>100)	*Mycobacterium koreense*	WP_085304786	---S---QTV---R----	A	S-----T---
	Mycobacterium triviale	WP_069391244	---S---QTV---R----	A	S-----T---
	Mycobacterium abscessus	WP_074252256	--QGT--ETV-L-----P	G	S----FT-K-
	Mycobacterium acapulcensis	WP_066810495	--TNV-L------	A	S-----T---
	Mycobacterium alsense	WP_083137299	--KSA--AQV-L-R----	V	S-----T---
	Mycobacterium angelicum	WP_083112984	---S---EQI-L------	V	S-----T---
	Mycobacterium aromaticivorans	WP_036340709	--TSL--ARV--------	V	S-----T---
	Mycobacterium arosiense	WP_083065460	--TSA--EQI-L-R----	V	S-----T---
	Mycobacterium asiaticum	WP_065034611	---S---EQI--------	V	S-----T---
	Mycobacterium aurum	WP_048630136	--------	V	S-----T-K-
	Mycobacterium austroafricanum	WP_036369913	---RI--------	V	S-----T-K-
	Mycobacterium avium	WP_062886665	---S---EQI-L-R----	I	S-----T---
	Mycobacterium avium subsp. avium 2285 (S)	EUA28407	---S---EQI---R----	V	S-----T---
	Mycobacterium avium subsp. paratuberculosis	ETB45686	---S---EQI-L-R----	I	S-----T---
	Mycobacterium bacteremicum	WP_083059220	--KS---ATV-------K	V	S-----T-K-
	Mycobacterium boenickei	WP_077743938	--ESA---NI-L------	A	S-----T-Q-
	Mycobacterium bohemicum	WP_085180114	---SA--EQV-L-R----	V	S-----T---
	Mycobacterium bovis	WP_079367521	---S---QRV-L-R----	V	S-----T---
	Mycobacterium branderi	WP_083130492	---SA--TDV---R----	V	S-----T---
	Mycobacterium brisbanense	WP_062830088	--ES----NI-L------	A	S-----T-Q-
	Mycobacterium canariasense	WP_062659913	--ES----TV-L-----K	V	S-----T-K-
	Mycobacterium canettii	WP_015291434	---S---QRV-L-R----	V	S-----T---
	Mycobacterium celatum	WP_062541489	---S---TDV---Q----	V	S-----T---
	Mycobacterium celeriflavum	WP_083000937	--ES----NV-L------	A	S-----T-K-
	Mycobacterium chelonae	WP_070922919	--HGT--ETV-------P	G	S----FT-K-
	Mycobacterium chlorophenolicum	WP_048471878	--TKM-I------	V	S-----T-K-
	Mycobacterium chubuense	WP_014814383	I-F------	V	S-----T-K-
	Mycobacterium colombiense	WP_064883716	--TS---EQV-L-R----	V	S-----T---
	Mycobacterium conceptionense	WP_076210956	---NI-L-----H	A	S-----T-Q-
	Mycobacterium confluentis	WP_085149341	--ES---ATI-L-----P	G	S-----T--
	Mycobacterium conspicuum	WP_085233540	---SM--EQV-L------	V	S-----T---
	Mycobacterium cosmeticum	WP_036401800	--ES----TV-L-----K	V	S-----T-K-
	Mycobacterium diernhoferi	WP_073856566	--QS---ATV-------K	V	S-----T-K-
	Mycobacterium doricum	WP_085192334	--QS---ARI-L------	V	S-----T---
	Mycobacterium elephantis	WP_046754119	--ESL---IL--------	V	S-----T---
	Mycobacterium europaeum	WP_085242177	---S---E-I-L-R----	A	S-----T---
	Mycobacterium fallax	ORV09136	--QS----TV-------P	G	SR---FT---
	Mycobacterium farcinogenes	WP_036388963	---NI-L-----H	A	S-----T-Q-
	Mycobacterium flavescens	WP_069416848	---NV-L-R----	V	S-----T---
	Mycobacterium florentinum	WP_085224446	--IS---EQI-L------	V	S-----T---
	Mycobacterium fortuitum	WP_061262612	--ES----NI-L------	A	S-----T-Q-
	Mycobacterium fragae	WP_085198379	---SA--TDV---R----	V	S-----T---
	Mycobacterium franklinii	WP_070938591	--HGT--ETV-L-----P	G	S----FT-K-
	Mycobacterium gastri	WP_036420080	---S---EQI-L-R----	V	S-----T---
	Mycobacterium genavense	WP_025737511	---S---EQI-L------	V	S-----T---
	Mycobacterium gilvum	WP_011895760	---KM--------	V	S-----T-K-
	Mycobacterium goodii	WP_049748067	--ESL---TI-L------	A	S-----T-Q-
	Mycobacterium gordonae	WP_065046523	---SA--EQI-L------	V	S-----T---
	Mycobacterium haemophilum	WP_047315543	---S---EQI-L-P----	V	S-----L---
	Mycobacterium hassiacum	WP_005625580	--ES----TV-L-R----	V	S-----T---
	Mycobacterium heckeshornense	WP_048890195	---SA---NV-L-R----	V	S-----T---

Group	Species	Accession	Sequence
**Other *Mycobacterium* (0/>100)**	*Mycobacterium heidelbergense*	WP_083075060	---S---ERI-L-R---- A S-----T---
	Mycobacterium holsaticum	WP_069404773	---TM-L------ V S-----T---
	Mycobacterium houstonense	WP_066901093	---NI-L------ A S-----T-Q-
	Mycobacterium immunogenum	WP_064627859	--QGA--ETV-L-----P G S----FT-K-
	Mycobacterium insubricum	WP_083031985	--QRA--ENV-L-----P V SR----T--
	Mycobacterium interjectum	WP_066907657	---ST--EQI-L-R---- V S-----T---
	Mycobacterium intermedium	WP_069417513	---SM--EQI-L------ V S-----T---
	Mycobacterium intracellulare MIN_052511_1280	ETZ27568	---S---EQI---R---- V S-----T---
	Mycobacterium iranicum	WP_064283750	---KI-------- V S-----T-K-
	Mycobacterium kansasii	WP_063470006	---S---EQI-L-R---- V S-----T---
	Mycobacterium komanii	CRL75348	--TNV-L-R---- A S-----T---
	Mycobacterium kubicae	WP_085073723	---S---EQV-L-R---- V S-----T---
	Mycobacterium kyorinense	WP_065012708	---S---TDV-L-R---- I S-----T---
	Mycobacterium lacus	WP_085160741	---SA--EQI-L-R---- V S-----T---
	Mycobacterium lentiflavum	CQD07141	---S---EQI-L------ V S-E---T---
	Mycobacterium leprae	WP_010907694	---S---ERI-L-P---P A S-----L---
	Mycobacterium lepromatosis	WP_045842432	---S---ERM-L-P---P V S-----F---
	Mycobacterium litorale	WP_078020613	--TSL---KV-------- V S-----T---
	Mycobacterium llatzerense	WP_071287935	--ES----KM-------- I S-----T---
	Mycobacterium mageritense	WP_036428931	--ES----NI-L------ A S-----T---
	Mycobacterium malmesburyense	CRL78440	--TNV-L-R---- A S-----T---
	Mycobacterium malmoense	WP_071509856	---ST--TRI-L-R---- A S-----T---
	Mycobacterium mantenii	WP_083093490	--TS---ERI-L-R---- V S-----T---
	Mycobacterium marinum str. Europe	EPQ70321	---S---EQV-L-R---- V S-----T---
	Mycobacterium marseillense	WP_083019862	--TS---EQI-L-R---- V S-----T---
	Mycobacterium moriokaense	WP_083154071	---NV-L-R---- A S-----T-K-
	Mycobacterium mucogenicum	WP_064859883	--ES----KM-------- I S-----T---
	Mycobacterium nebraskense	WP_046184667	---SA--E-V-L-R---- A S-----T---
	Mycobacterium neoaurum	WP_030134583	--TS----TV-------K V S-----T-K-
	Mycobacterium neworleansense	CRZ15947	--ES----NI-L------ A S-----T-Q-
	Mycobacterium noviomagense	WP_083087869	---S---INV-L-R---- V S-----T---
	Mycobacterium novocastrense	WP_067396200	--TNV-L------ A S-----T---
	Mycobacterium obuense	WP_046366580	--TRI-L------ V S-----S-K-
	Mycobacterium palustre	WP_085079318	---SS--ERI-L-R---- V S-----T---
	Mycobacterium paraense	WP_085096372	---ST--EQI-L------ I S-----T---
	Mycobacterium paraffinicum	WP_073880295	---S---EQI-L-R---- A S-----T---
	Mycobacterium parafortuitum	WP_083146875	--EKM-------- V S-----T-K-
	Mycobacterium paraseoulense	WP_083175360	---S---EQI-L-R---- A S-----T---
	Mycobacterium parmense	WP_085272018	---S---EQI-L-R---- V S-----T---
	Mycobacterium peregrinum	WP_064888076	--ES----NI-L------ A S-----T-Q-
	Mycobacterium phlei	WP_061481152	---NV-L------ A S-----T---
	Mycobacterium porcinum	WP_069424916	--ES----NI-L------ A S-----T-Q-
	Mycobacterium rhodesiae	WP_014209045	--ESL---NV-L-R---- A S-----T-K-
	Mycobacterium riyadhense	WP_085250277	---S---EQI-L------ I S-----T---
	Mycobacterium rufum	KGI67109	--TKM-------- V S-----T-K-
	Mycobacterium rutilum	WP_083410119	---NV-L-R---- A S-----T---
	Mycobacterium salmoniphilum	WP_078323563	--QGT--ETV-------P G S----FT-K-
	Mycobacterium saopaulense	WP_070909451	--HGT--ETV-------P G S----FT-K-
	Mycobacterium saskatchewanense	WP_085255564	---S---EQV-L------ I S-----T---
	Mycobacterium scrofulaceum	WP_067280432	---S---EQI-L-R---- A S-----T---
	Mycobacterium septicum	WP_044516335	--ES----NI-L------ V S-----T-Q-
	Mycobacterium setense	WP_039317103	--ES----NI-L------ A S-----T-Q-
	Mycobacterium sherrisii	WP_069399111	---S---EQI-L-R---- V S-----T---
	Mycobacterium shimoidei	WP_069395366	---SF--TDV-L-R---- A S-----T---
	Mycobacterium shinjukuense	WP_083045864	---SA--ERI-L-R---- A S-----T---
	Mycobacterium simiae	WP_061558400	---S---EQI-L------ V S-----T---
	Mycobacterium smegmatis	WP_011727751	--ESL---TI-L------ A S-----T-Q-
	Mycobacterium szulgai	WP_085672419	---S---EQI-L------ V S-----T---
	Mycobacterium thermoresistibile	WP_003928053	--ESA--TRI-L-R---- Q S-----T---
	Mycobacterium triplex	WP_036466882	---S---EQ--L------ V S-----T---
	Mycobacterium tuberculosis	WP_078816363	---S---QRV-L-R---- V S-----T---
	Mycobacterium tusciae	WP_083124817	---NV-L-R---- A S-----T-K-
	Mycobacterium ulcerans str. Harvey	EUA92009	---S---EQV-L-R---- V S-----T---
	Mycobacterium vaccae	WP_003929244	---RV-------- V S-----T-K-
	Mycobacterium vanbaalenii	WP_011778746	---RV-I------ V S-----T-K-

Group	Species	Accession	Sequence
**Other *Mycobacterium* (0/>100)**	*Mycobacterium vulneris*	WP_065458753	--ES----NI-L------ A S-----T-Q-
	Mycobacterium wolinskyi	WP_067842636	--ESA---NI-L------ A S-----T-Q-
	Mycobacterium xenopi	WP_085197121	---SA---NV-L-R---- V S-----T---
	Mycobacterium yongonense	ARR79873	---S---EQI---R---- V S-----T---
**Other *Corynebacteriales***	*Corynebacterium callunae*	WP_015650458	M-E-F--QTLA---N--- P S-----T-Q-
	Corynebacterium durum	WP_006062944	--ESL---V--T--N--Q P S----FT-H-
	Gordonia aichiensis	WP_005174158	--ERN---QR---RN--Q P S-----T-A-
	Gordonia araii	WP_007320991	--ES----KS-L--G--Q P S-----T-I-
	Hoyosella altamirensis	WP_064438335	---G---EVV-L-----Q P S-----V-K-
	Hoyosella subflava	WP_013808823	---G---DVV-L-----Q P S-----V-K-
	Nocardia abscessus	WP_043687940	--HII-L--N--H P S-----T---
	Nocardia africana	WP_062961704	--HII-L--S--Q P S----FT---
	Rhodococcus coprophilus	WP_072699561	---RE--EIV-T--H--Q P S-----T-K-
	Rhodococcus corynebacterioides	WP_068148826	---ST--EIV-T-RN--- P S-----T---
	Skermania piniformis	WP_066468567	---RYR-DVL-------Q P S-----T-
	Tsukamurella paurometabola	WP_049825767	--ERN---VV-T--N--- P S-----T---
	Tsukamurella pseudospumae	WP_068573355	--ERN---TV----N--- P S-----T-K-
	Williamsia herbipolensis	WP_045822076	M--HN--EQM-L-----Q P S-----T---
	Williamsia muralis	WP_062800014	---RN--EIV-T--G--Q P S-----T---

Supplementary Figure 79

A partial sequence alignment of a conserved region of bifunctional tRNA (adenosine(37)-N6)-threonylcarbamoyltransferase complex dimerization subunit type 1 TsaB/ribosomal-protein-alanine acetyltransferase RimI showing a one amino acid deletion that is specific for members of the "*Terrae*" clade and absent in other *Corynebacteriales*.

Group	Species	Accession	104		137
***"Terrae"* Clade (9/9)**	*Mycobacterium arupense*	KKB98129	DCPDSTLAVKGLT		GPQYTVGEQPQFTMVVTNIG
	Mycobacterium engbaekii	WP_085128772	-------------		A-------------------
	Mycobacterium heraklionense	OBG35220	-------------		--------------------
	Mycobacterium hiberniae	WP_085134039	-------------		A-------------------
	Mycobacterium kumamotonense	WP_065287577	-----------I-		----V---------------
	Mycobacterium longobardum	WP_085266462	-----------I-		----A---------------
	Mycobacterium senuense	WP_085085799	-----------I-		-----L--------------
	Mycobacterium sinense	WP_064856074	-----------I-		----V---------------
	Mycobacterium terrae	WP_085261833	-----------I-		----V---------------
**Other *Mycobacterium* (0/>100)**	*Mycobacterium triviale*	WP_085110483	-----V-------	N	Q-R-------K---------
	Mycobacterium abscessus	WP_074250328	----A-----AS-	D	K-S-LA----K---------
	Mycobacterium abscessus subsp. bolletii	SIJ84077	----A-----AS-	D	K-S-LA----K---------
	Mycobacterium acapulcensis	WP_083997641	-----------I-	N	Q---V-----K---------
	Mycobacterium alsense	WP_083139974	-------------	N	A---F--D--K---------
	Mycobacterium angelicum	WP_083115954	-------------	N	S---YI-D--K---------
	Mycobacterium aromaticivorans	WP_036346381	-----N-----I-	N	Q---AI-D--K---------
	Mycobacterium arosiense ATCC BAA-1401	ORA10474	-------------	N	A---FI-D--K---------
	Mycobacterium asiaticum	WP_065034567	-------------	N	S---YI-D--K---------
	Mycobacterium aurum	WP_048634246	-----------I-	N	Q---V--D--K---------
	Mycobacterium austroafricanum	WP_036375551	-----------I-	N	Q---V--D--K---------
	Mycobacterium avium	WP_062894460	-------------	N	A---FI-D--K---------
	Mycobacterium avium subsp. hominissuis	ETB33850	-------------	N	A---FI-D--K---------
	Mycobacterium avium subsp. paratuberculosis	ETB09739	-------------	N	A---FI-D--K---------
	Mycobacterium boenickei	WP_077738743	-----------I-	S	Q-E-V--D--K---------
	Mycobacterium bohemicum	WP_085182790	-------------	N	A---FI-D--K---------
	Mycobacterium bovis	WP_079367572	-------------	N	A---Y--D--K---------
	Mycobacterium branderi	WP_083131128	-------------	N	A---AI-D--K---------
	Mycobacterium brisbanense	WP_062828463	-----------I-	S	Q---V--D--K---------
	Mycobacterium canariasense	WP_062655958	-----------I-	S	A-D-VM-D--K---------
	Mycobacterium canettii	WP_080628478	-------------	N	A---Y--D--K---------
	Mycobacterium celatum	WP_085167477	-------------	N	A---VI-D--K---------
	Mycobacterium chelonae	WP_046255468	----A-----AS-	D	K-S-LA----K---------
	Mycobacterium chimaera	WP_072501154	-------------	N	A---FI-D--K---------
	Mycobacterium chlorophenolicum	WP_048468671	-----------I-	N	E---V--D--K---------
	Mycobacterium chubuense	WP_014817866	-----------I-	N	Q---V--D--K---------
	Mycobacterium colombiense	OBK61428	-------------	N	A---FI-D--K---------
	Mycobacterium conceptionense	CQD23522	M--I-	S	Q-E-V--D--K---------
	Mycobacterium confluentis	WP_085155837	-----------I-	N	E-R--I-D--K---------
	Mycobacterium conspicuum	WP_085230967	-------------	N	A---FI-D--K---------
	Mycobacterium diernhoferi	WP_073856837	-----------I-	S	A-D-VM-D--K---------
	Mycobacterium doricum	WP_085188396	-----------I-	S	Q-E-V--D--K---------
	Mycobacterium elephantis	KKW63760	-----------I-	N	Q---V--D--K---------
	Mycobacterium europaeum	WP_085239768	-------------	N	A---FI-D--K---------
	Mycobacterium fallax	WP_085096820	--T--------I-	N	E-R-V--D--K---------
	Mycobacterium flavescens	WP_069415267	-----------I-	N	Q---V-----K---------
	Mycobacterium florentinum	WP_085223291	-------------	N	A---FI-D--K---------
	Mycobacterium fortuitum	WP_064850954	-----------I-	S	Q-E-V--D--K---------
	Mycobacterium fragae	WP_085199016	-------------	N	A---VI-D--K---------
	Mycobacterium franklinii	WP_070939052	----A-----AS-	D	KAS-LA----K---------
	Mycobacterium gastri	WP_036413402	-------------	N	A---YI-D--K---------
	Mycobacterium genavense	WP_025736780	-------------	N	A---FI-D--K---------
	Mycobacterium gilvum	WP_011892337	-----------I-	N	Q--FI--D--K---------
	Mycobacterium goodii	WP_049744639	-----------I-	S	Q-E-V--D--K---------
	Mycobacterium gordonae	WP_065047428	----A--------	N	S---YI-D--K---------
	Mycobacterium haemophilum	WP_047316728	-------------	N	A---FI-D--K---------
	Mycobacterium hassiacum	WP_081586683	-----------I-	N	Q---V-----K---------
	Mycobacterium heckeshornense	WP_048891091	-------------	N	Q---VI-D--K---------
	Mycobacterium heidelbergense	WP_083073716	-------------	N	A---FI-D--K---------
	Mycobacterium holsaticum	WP_069405953	-----------I-	N	Q---V--D--K---------
	Mycobacterium houstonense	WP_066902410	-----------I-	S	Q-E-V--D--K---------
	Mycobacterium immunogenum	OAT68477	----A-----AS-	D	K-S-LA----K---------
	Mycobacterium indicus pranii MTCC 9506	AFS12618	-------------	N	A---FI-D--K---------
	Mycobacterium insubricum	WP_083032177	-----------I-	N	Q-R-N--D--K---------
	Mycobacterium interjectum	WP_066916484	-------------	N	A---FI-D--K---------

Group	Species	Accession			
Other *Mycobacterium* (0/>100)	*Mycobacterium intermedium*	WP_069419442	-------------	N	Q---YI-D--K---------
	Mycobacterium intracellulare	WP_009957609	-------------	N	A---FI-D--K---------
	Mycobacterium iranicum	WP_064279766	-----------I-	N	Q--FI--D--K---------
	Mycobacterium kansasii	WP_063466578	-------------	N	A---YI-D--K---------
	Mycobacterium komanii	CRL75757	-----------I-	N	Q---V-----K---------
	Mycobacterium kyorinense	WP_065015105	-------------	N	A---VI-D--K---------
	Mycobacterium lacus	WP_085157062	-------------	N	S---F--D--K---------
	Mycobacterium lentiflavum	CQD21710	-------------	N	A---FI-D--K---------
	Mycobacterium leprae	WP_010908613	-------------	N	V---FL-D--K---------
	Mycobacterium lepromatosis	KJX74911	----A--------	N	V---FI-D--K---------
	Mycobacterium litorale	WP_078021128	-----N-----I-	N	Q---VI-D--K---------
	Mycobacterium llatzerense	WP_043984532	----T---A--I-	N	Q-N-VI-D--K---------
	Mycobacterium malmesburyense	CRL69240	-----------I-	N	Q---V-----K---------
	Mycobacterium malmoense	WP_065445791	-------------	N	A---FI-D--K---------
	Mycobacterium mantenii	WP_083095408	-------------	N	A---FI-D--K---------
	Mycobacterium marinum	WP_020730843	-------------	N	A---YI-D--K---------
	Mycobacterium marseillense	WP_083019808	-------------	N	A---FI-D--K---------
	Mycobacterium microti	AMC61393	-------------	N	A---Y--D--K---------
	Mycobacterium mucogenicum	OBA81401	----T---A--I-	N	Q-T-VI-D--K---------
	Mycobacterium nebraskense	WP_047321946	-------------	N	A---FI-D--K---------
	Mycobacterium neworleansense	CRZ19149	-----------I-	S	Q-E-V--D--K---------
	Mycobacterium noviomagense	ORB12885	-------------	N	Q---VI-D--K---------
	Mycobacterium novocastrense	WP_084377668	-----------I-	N	Q---V-----K---------
	Mycobacterium obuense	KKF02163	-----------I-	N	Q---V--D--K---------
	Mycobacterium paraense	WP_085246306	-------------	N	A---FI-D--K---------
	Mycobacterium paraffinicum	WP_073871377	-------------	N	A---FI-D--K---------
	Mycobacterium paraintracellulare	AFC52004	-------------	N	A---FI-D--K---------
	Mycobacterium parascrofulaceum	WP_040620865	-------------	N	A---F--D--K---------
	Mycobacterium paraseoulense	WP_083174463	-------------	N	A---FI-D--K---I-----
	Mycobacterium parmense	WP_085270378	----A--------	N	A---FI-D--K---------
	Mycobacterium peregrinum	WP_064883911	-----------I-	S	Q---V-----K---------
	Mycobacterium phlei	WP_040634274	-----------I-	N	Q---V--D--K---------
	Mycobacterium porcinum	WP_075920725	-----------I-	S	Q-E-V--D--K---------
	Mycobacterium rhodesiae	WP_063823517	-----N-----I-	N	Q---VI-D--K---------
	Mycobacterium riyadhense	WP_085251197	-------------	N	S---YI-D--K---------
	Mycobacterium rufum	KGI71153	-----------I-	N	E---V--D--K---------
	Mycobacterium rutilum	WP_083406992	-----------I-	N	Q---V-----K---------
	Mycobacterium salmoniphilum	WP_078324915	----A-----AS-	D	K-S-LA----K---------
	Mycobacterium saopaulense	OHT81269	----A-----AS-	D	K-S-LA----K---------
	Mycobacterium saskatchewanense	WP_085255302	-------------	N	A---FI-D--K---------
	Mycobacterium scrofulaceum	WP_067311036	-------------	N	A---FI-D--K---------
	Mycobacterium septicum	WP_044520847	-----------I-	S	Q-E-V--D--K---------
	Mycobacterium setense	WP_039325665	-----------I-	S	Q-E-V--D--K---------
	Mycobacterium sherrisii	WP_069400820	-------------	N	A---FI-D--K---------
	Mycobacterium shimoidei	WP_069394755	-----------Y-	N	A---AT-D--K---------
	Mycobacterium shinjukuense	WP_083050518	-------------	N	A---YL-D--K---------
	Mycobacterium simiae	WP_061557379	-------------	N	A---FI-D--K---------
	Mycobacterium smegmatis	WP_080590960	-----------I-	S	Q-E-V--D--K---------
	Mycobacterium szulgai	WP_085670155	-------------	N	S---YI-D--K---------
	Mycobacterium thermoresistibile	WP_040546599	-----------I-	S	Q-E-V--D--K---------
	Mycobacterium triplex	WP_036471808	-------------	N	A---FI-D--K---------
	Mycobacterium tuberculosis	SGQ11766	-------------	N	A---Y--D--K---------
	Mycobacterium tusciae	WP_083129159	-----------I-	N	Q---V--D--K---------
	Mycobacterium ulcerans	WP_011741805	-------------	N	A---YI-D--K---------
	Mycobacterium vaccae	WP_003930667	-----------I-	N	Q---V--D--K---------
	Mycobacterium vanbaalenii	WP_041308237	-----------I-	N	Q---V--D--K---------
	Mycobacterium vulneris	WP_085292207	-------------	N	A---FI-D--K---------
	Mycobacterium wolinskyi	WP_067850426	-----------I-	S	Q-E-V--D--K---------
	Mycobacterium xenopi	ORX18923	-------------	N	Q---VI-D--K---------
	Mycobacterium yongonense	WP_065508150	-------------	N	A---FI-D--K---------
Other *Corynebacteriales*	*Gordonia aichiensis*	WP_040517414	---QNI--VLY-	D	K-T----D--V--I-T--A-
	Gordonia amarae	WP_005191568	---ENIS-VLY-	D	K-T----S--V--I----A-
	Hoyosella altamirensis	WP_083962552	-A--S-S-RATI	D	RAE-PI-D--R-GV------
	Hoyosella subflava	WP_083826420	-A--S-S-RATI	D	RAE-PI-D--R-GV------
	Nocardia asiatica	WP_043718603	---QS--I-VTV	E	Q-T-KA----V-GI-I---

Group	Species	Accession	Sequence
**Other *Corynebacteriales***	*Nocardia beijingensis*	WP_067794687	---QS--I-VTV E Q-T-KT----V-GI-I---
	Rhodococcus coprophilus	WP_072702557	---QS---RVTA D E-N-Q--TE-G--I-I----
	Rhodococcus corynebacterioides	WP_068149226	----S---RATP D Q-S-AA-QE-G--V------
	Skermania piniformis	WP_083529986	---TS----ATI E Q-TFQS-DE-V-GV-I---
	Smaragdicoccus niigatensis	WP_081628795	---TSI---STV D H-N-KK-DE-V-GI-I---
	Tomitella biformata	WP_051461510	----AIGITVAP E K-N--A-Q--R-FTTI----
	Tsukamurella paurometabola	WP_013125277	---TNIS-VVAA D K-S-A-----T-EAT---A-
	Williamsia herbipolensis	WP_045821871	---QNISAVLY- D K-T----DK-V--I-I--A-
	Williamsia sterculiae	WP_076475911	---QNIS-VLY- D K-T-A--D--V--L----A-

Supplementary Figure 80

A partial sequence alignment of a conserved region of a membrane protein showing a one amino acid deletion that is specific for members of the "*Terrae"* clade and absent in other *Corynebacteriales*.

Group	Species	Accession	57		100
"Terrae" Clade (10/10)	*Mycobacterium algericum*	WP_083036231	RVNRGLSYRIFGEIADPADG	PEDPR	LPADTKIPDLLWSRLRITR
	Mycobacterium engbaekii	ORV47059	-------F-M---L---P--	-D---	------LT-----------
	Mycobacterium heraklionense	WP_064999628	--------------------	-----	-------T-----------
	Mycobacterium hiberniae	WP_085135882	-------F-V---L---P--	-D---	I-----LT-----------
	Mycobacterium icosiumassiliensis	WP_067974233	--------------------	-----	-------T-----------
	Mycobacterium kumamotonense	WP_065287169	---------M----EQ-V--	LGEAA	--PG-RAG---RQ------
	Mycobacterium nonchromogenicum	WP_085137035	--------------------	-----	-------T-----------
	Mycobacterium senuense	WP_085083764	---------M----------	-----	-------T-----------
	Mycobacterium sinense	WP_064853213	---------M----EQ-I-S	LGEAA	--PG-RAG---RQ------
	Mycobacterium terrae	WP_085261108	--------------------	-----	-------T-----------
Other *Mycobacterium* (1/45)	*Mycobacterium kyorinense*	WP_045373909	-L-------V-------V--	-D--A	----VSMRH--C------
	Mycobacterium koreense	WP_085303898	-I----M--V---LC--P-S		ADIV--VR-A--A--H--
	Mycobacterium triviale	WP_069393033	-I----M--V---LC--P-S		ADIV--VR-A--A--H--
	Mycobacterium arosiense	WP_083063573	-T----M----------P--		VGCVPAVRGA--A-----
	Mycobacterium avium	WP_062887161	-T----M--F-------P-E		AGSLPVARSM--A---VS
	Mycobacterium avium subsp. avium	ETB20660	-T----M--F-------P-E		AGSLPVARSM--A---VS
	Mycobacterium avium subsp. hominissuis	ETB32284	-T----M--F-------P-E		AGSLPVARSM--A---VS
	Mycobacterium avium subsp. paratuberculosis	AAS05769	-T----M--F-------P-E		AGSLPVARSM--A---VS
	Mycobacterium bohemicum	WP_085181991	-T----M--F-A---E-P--		SDSVAAEREM--A---V
	Mycobacterium celatum	WP_085167704	-T----M--Y-------P-E		NESTAQVRAA--K-----
	Mycobacterium chubuense	WP_014818042	-R--A-M--T--Q----P-E		AAMAPMVKPR-AA----
	Mycobacterium colombiense	OBJ24130	------M----------P-E		PGCAPAVRGI--A-----
	Mycobacterium elephantis	WP_083043542	-A----M-LV-SQ----P-E		TGMAVGM--R--A----
	Mycobacterium fragae	WP_085199299	-T----M--F-------P-E		ADCTAQVRAS--------
	Mycobacterium hassiacum	WP_005632396	-T----M----DQ--R-P-E		-GCAALL-NQ-AA-----
	Mycobacterium heckeshornense	WP_048891263	------M--V-------P-E		TGVVAALR-M--A-----
	Mycobacterium heidelbergense	WP_083073865	-T----M--F-----E-P--		SQCLPAVREM--A---V
	Mycobacterium holsaticum	WP_069405498	------M--V-------P-E		TAMAPGSV-R--A----
	Mycobacterium komanii	CRL72110	------M--V-A-LVR-S--		SR--PDVI-K--A----
	Mycobacterium kumamotonense	WP_083079511	------M-----DL---P--		QGSIAAVR-A--K-----
	Mycobacterium leprae	CAB11017	SSKCEVI--F-AD----P--		NECTPTVREV--A-M---
	Mycobacterium longobardum	WP_085263813	-T----M------L---P-A		EGSIAAVRATV-K-----
	Mycobacterium malmesburyense	CRL69847	------M--V-ADLTR-P-E		S--PDVI-K--A----A
	Mycobacterium malmoense	WP_065443976	-T----M--C-A-----P--		SGCVPSVR-R--A-----
	Mycobacterium mantenii	WP_083093024	-I----M--V-------P-E		PGCAPAVRAM--A-----
	Mycobacterium minnesotense	WP_083022466	-I----M------L---P--		SGSIAAVRAAV-T-----
	Mycobacterium moriokaense	WP_083154009	-T----M--V--Q----P-E		IGLIDGLI-R-CA----P-
	Mycobacterium palustre	WP_085077173	------M--F-A-----P--		AESVPAVRAK-CA---V-
	Mycobacterium paraense	WP_085103619	------M--F-A---E-P--		SAGIPAVRA--SA-----
	Mycobacterium paraffinicum	WP_073872251	-T----M--F-A-----P--		GGLVPAVR-G--A-----
	Mycobacterium parascrofulaceum	EFG75816	-T----M--C-A-----P--		SGCVPAVR-K--A-----
	Mycobacterium paraseoulense	WP_083173393	-T----M--F-A-----P--		SGYVPSVR-K--A-----
	Mycobacterium rhodesiae	WP_014209000	-T--A-M--V---L---P-E		VGLVNDVI-S--A----
	Mycobacterium saskatchewanense	WP_085254688	-T----M--F-A-----P--		SGSVPAVR-A--A---V
	Mycobacterium scrofulaceum	WP_083177942	-T----M--Y-A-----P--		SGCVPAVR-K--A-----
	Mycobacterium shimoidei	WP_084226374	-T----M--F-------P-E		IDSSAQVRSA--R-----
	Mycobacterium sinense	WP_064920749	-T----M------L---P--		QGSIAQVRAA--K--H--
	Mycobacterium thermoresistibile	WP_040547188	-I----M--V-AQL---P-E		PAMVPAVR-R--A-----
	Mycobacterium tuberculosis	KBZ65578	-T----M--V----D--P-E		AGCVPAVRGI-CA-----
	Mycobacterium tusciae	WP_006244227	-T----M--V--Q----P-E		VGLVGALI-S--A----
	Mycobacterium vaccae	WP_040541436	-L--A-M--V--Q----P-E		AAMVPAVKQR-AA----
	Mycobacterium xenopi	WP_085196621	------M----------P-E		AG-IAAVRH---A-----
	Mycobacterium yongonense	AGP65492	-T----M----------P--		PGPVVLVR-M--A-----

Supplementary Figure 81

A partial sequence alignment of a conserved region of DUF222 domain-containing protein showing a five amino acid insertion that is specific for members of the "*Terrae*" clade and absent in most other *Mycobacterium*.

Group	Species	Accession	232		267
"Terrae" Clade (11/11)	*Mycobacterium algericum*	WP_083036343	LMTAFNWMSHGTHDIYPTFL		SSNDGAGLSHVTAKWI
	Mycobacterium avium	WP_019738394	--------------------		T-P-------------
	Mycobacterium engbaekii	ORV42794	--------------------		T-A-------------
	Mycobacterium heraklionense	WP_064888950	--------------------		T-P-------------
	Mycobacterium hiberniae	WP_085134849	--------------------		T-A-------------
	Mycobacterium kumamotonense	WP_065288305	--------------------		T-P-------------
	Mycobacterium longobardum	WP_085263573	------------Q-------		T-P-----P-------
	Mycobacterium minnesotense	WP_083022488	--------------------		T-A-------------
	Mycobacterium senuense	WP_085083515	--------------------		--D-------------
	Mycobacterium sinense	WP_064856659	------------Q-------		T-PE----PP------
	Mycobacterium terrae	WP_085260271	--------------------		T-A-------------
Other *Mycobacterium* (0/67)	*Mycobacterium koreense*	WP_085303956	-----T------Q-V-----	QA	TEHG--A--ST-----
	Mycobacterium triviale	ODR05644	-----T------Q-V-----	QA	TEHG--A--ST-----
	Mycobacterium africanum	WP_003910470	------------Q-V-----	TA	TTDH-----SL--R--
	Mycobacterium alsense	WP_083141074	------------Q-V-----	GA	TA-G-----SA-V---
	Mycobacterium angelicum	WP_083115740	------------Q-V-----	GA	TT-H-----S--V---
	Mycobacterium arosiense	WP_083064618	------------Q-V-----	GA	HA-Q-----ST-V--
	Mycobacterium asiaticum	OBK20011	------------Q-V-----	KA	TTDH-----SA--R--
	Mycobacterium avium	WP_084022968	------------Q-V-----	GA	HA-H-----ST-V---
	Mycobacterium avium subsp. avium	ETB20899	------------Q-V-----	GA	HA-H-----ST-V---
	Mycobacterium avium subsp. hominissuis	ETB29399	------------Q-V-----	GA	HA-H-----ST-V---
	Mycobacterium avium subsp. paratuberculosis	EGO36393	------------Q-V-----	GA	HA-H-----ST-V---
	Mycobacterium avium subsp. silvaticum	ETB09507	------------Q-V-----	GA	HA-H-----ST-V---
	Mycobacterium bohemicum	WP_085181751	------------Q-V-----	TS	TT-H----HSA-V---
	Mycobacterium bovis	WP_081005849	------------Q-V-----	TA	TTDH-----SL--R--
	Mycobacterium canettii	WP_015288181	------------Q-V-----	TA	TTDH-----SL--R--
	Mycobacterium colombiense	WP_064881427	------------Q-V-----	GA	HT-H--N-AST-V---
	Mycobacterium conspicuum	WP_085234575	------------Q-V-----	GA	TA-H-----S--V---
	Mycobacterium europaeum	WP_085238939	------------Q-V-----	TA	TT-H-----SA-V---
	Mycobacterium florentinum	WP_085220553	------------Q-V-----	GS	AA-H----DS--V---
	Mycobacterium fragae	ORV57175	----V-------Q-V-----	TA	-HSG----GSA-----
	Mycobacterium gastri	WP_036409153	------------Q-V-----	TA	TTDQ-----T---R--
	Mycobacterium genavense	WP_025737155	------------Q-V-----	GS	TA-H----DS--V---
	Mycobacterium gordonae	WP_065045170	------------Q-V-----	KA	-VDH-----SA--R--
	Mycobacterium haemophilum	WP_047314232	------------Q-V-----	GA	T--H----ASS-V---
	Mycobacterium heckeshornense	WP_082169833	--S---------Q-V-----	AA	TA-SS---GSA-T---
	Mycobacterium heidelbergense	WP_083077276	------------Q-V-----	TA	TA-H-----S--V---
	Mycobacterium indicus pranii	WP_043955750	------------Q-V-----	GA	HA-Q-----ST-VR--
	Mycobacterium interjectum	WP_066912942	------------Q-V-----	GA	TA-H-----S--V---
	Mycobacterium intermedium	WP_069421765	------------Q-V-----	KA	TTEH-----ST--R--
	Mycobacterium intracellulare	WP_064934795	------------Q-V-----	GA	HA-Q-----ST-VR--
	Mycobacterium kansasii	KZS72253	------------Q-V-----	TA	TTDQ-----TA--R--
	Mycobacterium kubicae	WP_085074335	------------Q-V-----	TA	TADH-----SA-----
	Mycobacterium kyorinense	OBI41542	------------Q-V-----	SA	-HDG--A--GA-T-S-
	Mycobacterium lacus	WP_085158119	------------Q-V-----	TS	TA-------SS-V---
	Mycobacterium lentiflavum	CQD14459	------------Q-V-----	GS	TA-H----DS--V---
	Mycobacterium liflandii	WP_041300241	------------Q-V--S--	TA	TTDH-----PA--R--
	Mycobacterium litorale	WP_078022361	------------Q-VF----	SA	TH-G-----TE--R--
	Mycobacterium malmoense	WP_065442879	------------Q-V-----	TS	TA-H-----SA-V---
	Mycobacterium mantenii	WP_083092670	------------Q-V-----	GA	HADQ-----ST-V---
	Mycobacterium marinum	WP_020725324	------------Q-V--S--	TA	TTDH-----SA--R--
	Mycobacterium marseillense	WP_083020018	------------Q-V-----	GA	HT-Q--D--SA-V---
	Mycobacterium microti	AMC59540	-----------Q-V-----	TA	TTDH-----SL--R--
	Mycobacterium nebraskense	WP_046185172	------------Q-V-----	TS	TA-H-----SA-V---
	Mycobacterium noviomagense	WP_083087015	------------Q-V-----	SA	T--G----GSA-T---
	Mycobacterium orygis	WP_003409541	------------Q-V-----	TA	TTDH-----SL--R--
	Mycobacterium palustre	WP_085078509	------------Q-V-----	GA	TA-H-----G--V---
	Mycobacterium paraense	WP_085100422	------------Q-V-----	GA	TG-H--A--SA-V---
	Mycobacterium paraffinicum	WP_073874346	------------Q-V-----	TS	TA-H--A--SA-V---
	Mycobacterium paraintracellulare	AFC54001	------------Q-V-----	GA	HA-Q-----ST-VR--
	Mycobacterium parascrofulaceum	EFG79217	------------Q-V-----	TS	TA-H-----SA-V---
	Mycobacterium paraseoulense	WP_083173121	------------Q-V-----	TS	TA-H--A--SA-V---
	Mycobacterium parmense	ORW51592	------------Q-V-----	TA	TASH-----S-SV---
	Mycobacterium pseudoshottsii L15	GAQ38748	------------Q-V--S--	TA	TTDH-----SA--R--

Group	Species	Accession	Sequence
**Other *Mycobacterium* (0/67)**	*Mycobacterium riyadhense*	ORW81295	------------Q------- TA TTDH-----ST--R--
	Mycobacterium saskatchewanense	WP_085257188	------------Q-V----- TS TA-H-----SA-V---
	Mycobacterium scrofulaceum	WP_067276248	------------Q-V----- TS TA-H--A--SA-V---
	Mycobacterium sherrisii	WP_085166199	------------Q-V----- SS TA-H----DP--V---
	Mycobacterium shimoidei	ODR13113	------------Q-V----- AA TH-Q-----AA---S-
	Mycobacterium shinjukuense	WP_083049132	------------Q-V----- TA T-DH----HS---R--
	Mycobacterium simiae	WP_044511794	------------Q-V----- SS TA-H----DG--V---
	Mycobacterium szulgai	WP_068023896	------------Q-V----- TA TADH-----SA-----
	Mycobacterium triplex	WP_036472509	------------Q-V----- GS TA-H----DS--V---
	Mycobacterium tuberculosis	WP_070895454	------------Q-V----- TA TTDH-----SL--R--
	Mycobacterium ulcerans Agy99	ABL05217	------------Q-V--S-- TA TTDH-----SA--R--
	Mycobacterium vulneris	WP_085289728	------------Q-V----- GA HA-H--S-AST-V---
	Mycobacterium yongonense	WP_065507708	------------Q-V----- GA HA-Q-----ST-VR--

Supplementary Figure 82

A partial sequence alignment of a conserved region of MFS transporter showing a two amino acid deletion that is specific for members of the "*Terrae"* clade and absent in other *Mycobacterium*.

			369		407
**"*Terrae*" Clade (12/12)**	*Mycobacterium algericum*	WP_083036337	QPVGSPGLRAAQRGQTGTLNTG	Y	DYLGNRELAAYAPLTI
	Mycobacterium arupense	ORA00874	---SG---Q----------TS-	-	----K---T--S---V
	Mycobacterium engbaekii	WP_085129689	----G---Q--H-------TA-	-	--------T------V
	Mycobacterium heraklionense	WP_064888947	----G---E------S---TN-	-	A-------T-----AV
	Mycobacterium hiberniae	WP_085134749	----G---Q--H-------TA-	-	--------T------V
	Mycobacterium icosiumassiliensis	WP_078058737	---SG---Q------S---TN-	-	----K---T----V-V
	Mycobacterium kumamotonense	OBY31555	----------------------	-	----K---V--T----
	Mycobacterium longobardum	WP_085263541	---EA-----------------	-	--------V------V
	Mycobacterium minnesotense	ORB04629	---NG---Q----------TS-	-	----K---T--S---V
	Mycobacterium senuense	WP_085083525	--------S-------------	-	---------T------
	Mycobacterium sinense	OBK84732	----A---H-------------	-	--------V-------
	Mycobacterium terrae	WP_085260277	--------H-------------	-	----K---V-------
**Other *Mycobacterium* (0/91)**	*Mycobacterium triviale*	ORX00619	--ISEEST-L-AQ----NVI-R		-----QT-QS---V-L
	Mycobacterium abscessus subsp. abscessus	SHW83211	---PPTTNKQ-L--ES--RLAT		----HEV-L----I--
	Mycobacterium africanum	WP_061846064	---ATE------------VTST		--T-S---E-----NV
	Mycobacterium alsense	WP_083136719	---PTA--K-----ES--VSAT		--A--K--E-----D-
	Mycobacterium angelicum	ORA19551	---H-A-----------VTSDT		--------Q-----NV
	Mycobacterium aromaticivorans	WP_081845244	--MT-AAT-N--K-E---II-Q		----QDT-Q----VV-
	Mycobacterium asiaticum	OBK19497	--IH-A--K--L--D--VVVDT		--T--K--E-----D-
	Mycobacterium avium	WP_062889637	---ATAAV------E---VTDT		G---R---------SL
	Mycobacterium avium subsp. avium 10-9275	ETB17752	---ATA--------E--VVTAT		--M-----E-----N-
	Mycobacterium avium subsp. paratuberculosis	ETB12718	---ATA--------E--VVTAT		--M-----E-----N-
	Mycobacterium boenickei	WP_077740842	---ETTAT-L-S---S--VIED		-----DT-Q----V
	Mycobacterium bohemicum	ORU95998	---ATA--------ES-VISAT		--M-----E-----N-
	Mycobacterium bovis	WP_024457096	---ATE------------VTST		--T-S---E-----NV
	Mycobacterium branderi	WP_083132947	---ATAAVG-----H---VIDT		S---R---V-----SV
	Mycobacterium canariasense	WP_084395168	-----EATKL-----R---IAR		----KET-Q----VD-
	Mycobacterium canettii	WP_015290666	---ATE------------VTST		--T-S---E-----NV
	Mycobacterium chelonae	OHT47980	---D-DATQ------S---IAT		N---QET-Q-----VL
	Mycobacterium colombiense	WP_064878448	---DTV-----K--E--VVSAT		--------E-----S-
	Mycobacterium conceptionense	OBB08877	---ETTAT-L-S---S--VIED		-----DT-Q----V
	Mycobacterium confluentis	ORV31984	--IH-E-VTR----EV--VV-E		-----KT-Q----VD-
	Mycobacterium conspicuum	WP_085235075	---P-A--------E--VMSAT		--------E-----D-
	Mycobacterium cosmeticum	CDO05316	-----EATKL-----R---IAR		----KET-Q----VDV
	Mycobacterium europaeum	WP_085240251	---PTA-----------VISAT		-----K--E-F---SL
	Mycobacterium farcinogenes	WP_084676619	---ETTAT-L-S---S--VIED		-----DT-Q----V
	Mycobacterium flavescens	WP_069413606	---ATEATGL----E----VDR		----QET-Q----VNL
	Mycobacterium florentinum	WP_085220707	--APTA-F--S-------VTAT		--T--S-ME----VVV
	Mycobacterium fortuitum	WP_064866999	---ETSAT-L-----S--VVED		-----ET-Q----VNL
	Mycobacterium fragae	ORV56753	---DTA---------R-VVADT		--T-----E------V
	Mycobacterium franklinii	WP_083338188	---D-EATQ------S---IAT		N---QQT-QS----KL
	Mycobacterium gastri	WP_084293342	---P-A-V---------VTKDT		--------E-----DV
	Mycobacterium genavense	WP_051465166	---QT----------L--VTAT		--T--N-ME----F-V
	Mycobacterium gordonae	WP_065045595	---R-A--K-----E--VTSGT		--T-----E-----S-
	Mycobacterium haemophilum	WP_047315038	---P-A------------VTAT		--Q----ME------V
	Mycobacterium heckeshornense	WP_082169972	---DTA---------N-VIGDT		--T-----E--G--SV
	Mycobacterium heidelbergense	WP_083072039	---ATA--------E--VVGAT		--T-H---E-----D-
	Mycobacterium houstonense	WP_066897595	---ETEAT-L-L------VIED		-----ET-Q----V
	Mycobacterium immunogenum	OAT68110	-----DATQ------S--VIAT		N---QQT-Q-----VL
	Mycobacterium indicus pranii	WP_014942368	---ATAAV---D--E----TDT		S---R---------SV
	Mycobacterium interjectum	WP_066914165	----TA--K-----ES--MSAT		--T--K--E-----D-
	Mycobacterium intermedium	WP_069417401	---QTE---L-L---S-VTRDI		G-----Q-E-----NL
	Mycobacterium intracellulare	WP_064893627	---ATAAV---D--ES---TDT		S---R---------SV
	Mycobacterium kansasii	KZS57018	---P-A-V-Y-------VTKDT		--------E----VDV
	Mycobacterium kubicae	WP_085073369	---P-R-----L---S-VDSDT		--------E-----N-
	Mycobacterium kyorinense	WP_065016714	---QTA---D-------VVAGT		G---EK--Q-----NV
	Mycobacterium lacus	WP_085162983	---A-E------------VSDT		--T--K--E------V
	Mycobacterium lentiflavum	CQD17378	--AETS-F--S-------VTGT		--T--N-ME--T--NL
	Mycobacterium liflandii	WP_083866479	---P-A-----L---S-ITKDT		--------E-----DV
	Mycobacterium litorale	WP_078019098	--DI-EATTN-R--ES---VSV		----E-A-QS----DQ
	Mycobacterium llatzerense	WP_071286850	---PPDTN-Q-L--ES--RLAT		----HEV-L----IV-
	Mycobacterium llatzerense	WP_082068076	----G-AT--T----S-V-IAT		----KET-QS---M-V
	Mycobacterium mageritense	WP_036439988	---NGTAA-QG---ES--VIET		-----EA-ES-S-VNL
	Mycobacterium malmoense	OCB30354	---ATA-----H-----V-SAT		--------E------V

Other *Mycobacterium* (0/91)

Mycobacterium mantenii	WP_083097041	---DTA--------ES-VVSAT	--------E-----S-
Mycobacterium marinum	WP_081651084	---P-A-----L---S-ITKDT	--------E-----DV
Mycobacterium marseillense	WP_083015534	---PTA--------E--VISAT	--T-----E-----N-
Mycobacterium mucogenicum	OBJ44261	----G-AT-------S-V-IAT	----QET-QS---V-V
Mycobacterium mucogenicum	WP_064860832	---PPTTNKQ-L--ES--RLAT	----HEV-L----I--
Mycobacterium nebraskense	WP_046182130	---PTA-----------VVSAT	--------E------V
Mycobacterium neworleansense	CRZ14171	---ETTAT-S-L------VIED	-----ET-Q----VNL
Mycobacterium noviomagense	WP_083084375	---DTA-F----Q----VISAT	--T-----E------V
Mycobacterium palustre	WP_085078205	---ATA--------E--VMSAT	--T-H---E--G--N-
Mycobacterium paraense	ORW51363	---ETA-F------E---VSAT	-----K--E-----D-
Mycobacterium paraffinicum	WP_073880141	---PTA-----------VVSAT	--------E------V
Mycobacterium parafortuitum	WP_083143842	---NTESVEV----N--A-IED	----HQA-QS-S-VDL
Mycobacterium paraintracellulare	WP_014385131	---ATAAV---D--ES---TDT	S---R---------SV
Mycobacterium paraseoulense	WP_083169224	---PTA-----------VISAT	--------E--S---V
Mycobacterium parmense	ORW62935	---WTA-----L-----VVSAT	--M-----E-----DV
Mycobacterium peregrinum	WP_064879894	---ETEAT-S-L------VIED	-----ET-Q----V-L
Mycobacterium pseudoshottsii L15	GAQ39823	---P-A-----L---S-ITKDT	--------E-----DV
Mycobacterium rhodesiae	WP_005144230	--DA-QATSN-R--E----VSV	------A-ES----PD
Mycobacterium rhodesiae	WP_041302535	---PTEATKL-----A---IAQ	----QET-Q----AD-
Mycobacterium riyadhense	WP_085250758	---A-E------------VSDT	--T--H--E------V
Mycobacterium rutilum	WP_083408702	---A-EAT-S--------IVER	----QET-Q----VNL
Mycobacterium salmoniphilum	WP_078327329	---D-DAT-------S---IAT	N---QQT-Q-----VL
Mycobacterium saskatchewanense	ORW65914	---P-A--------E--VVTGT	--T-----E-----N-
Mycobacterium scrofulaceum	WP_067283061	---PTA-----------V-SAT	--------E------V
Mycobacterium septicum	WP_044518763	---ETTAT-L-L------VIED	-----ET-Q----VNL
Mycobacterium setense	WP_064872049	---ETTAT-L-L---S--VIED	----DET-Q----V-L
Mycobacterium sherrisii	ODR09727	--AITA-F----------VTAT	--T--N-ME-F---NV
Mycobacterium shimoidei	ODR15165	---A-A---D-----S-VVAGV	-----K--E-----D-
Mycobacterium shinjukuense	ORB71365	---V-E------------VSDT	--T-----E-----NV
Mycobacterium simiae	WP_061556334	--ATTA-F-------A--VTAT	--T--S-ME-F---NV
Mycobacterium smegmatis	WP_080628085	---SDEASKL-GQ--S-SIIER	----QET-Q-----DL
Mycobacterium szulgai	WP_068033530	---P-R-----L---S-VDSDT	--------E-----N-
Mycobacterium triplex	WP_036470387	---Q-----------S--VTAT	--T--N-ME--T---V
Mycobacterium tuberculosis	WP_070889713	---ATE------------VTST	--T-S---E-----NV
Mycobacterium tusciae	WP_083128426	---PTDATKF-----S---IAQ	----QET-Q----
Mycobacterium ulcerans	OIN24829	---P-A-----L---S-ITKDT	--------E-----DV
Mycobacterium vaccae	WP_003932330	---TTRSVVE----N---TIED	----HEA-Q--S-VDL
Mycobacterium vulneris	WP_085291592	---DTA-----K--E--VVSAT	--------E--S--S-
Mycobacterium wolinskyi	WP_067859489	---NDEASKM-----S-SIIEA	----KET-Q----V
Mycobacterium yongonense	WP_065500174	---PTA--------E--VMSAT	--T-----E-----D-

Supplementary Figure 83

A partial sequence alignment of a conserved region of adenylate/guanylate cyclase domain-containing protein showing a one amino acid insertion that is specific for members of the "*Terrae"* clade and absent in other *Mycobacterium*.

			154		187
"Terrae" Clade (13/13)	Mycobacterium algericum	WP_083037148	YQYSAMALAKTYTAITKVVKIPA	LSG	VAEVVVFF
	Mycobacterium arupense	WP_046188579	---A----------------V--	---	--------
	Mycobacterium engbaekii	WP_085128976	---G-------------------	---	--------
	Mycobacterium heraklionense	WP_064888814	------------------L----	---	--------
	Mycobacterium hiberniae	WP_085136185	---G-------------------	---	--------
	Mycobacterium icosiumassiliensis	WP_067976857	---A-------------------	-N-	--------
	Mycobacterium kumamotonense	WP_065287025	---------------S------V	---	--------
	Mycobacterium longobardum	WP_085267026	---------------S---A---	---	--------
	Mycobacterium minnesotense	ORA99916	---A----------------V--	-M-	--------
	Mycobacterium nonchromogenicum	WP_085139592	-----------------------	---	--------
	Mycobacterium senuense	WP_085081886	-----------------------	---	--------
	Mycobacterium sinense	WP_064854043	---A-------------------	---	--------
	Mycobacterium terrae	WP_085260161	---------------S-------	---	--------
Other *Mycobacterium* (0/76)	Mycobacterium triviale	WP_085110064	---V--T-------AS-LFAL-V		-----M--
	Mycobacterium abscessus	AMU23997	---T---------QASD-LGL-I		-----M--
	Mycobacterium abscessus subsp. bolletii	SKL33490	---T---------QASDLLGL-I		-----M--
	Mycobacterium acapulcensis	WP_083997769	---V--S-------L--L-SV-V		I----M--
	Mycobacterium alsense	WP_083136428	---AS--V------LS--APL-V		--------
	Mycobacterium aromaticivorans	WP_036342211	---V----------VA-M-SL--		-----M--
	Mycobacterium arosiense	WP_083065402	---VS---------LS-LAPL-V		-----M--
	Mycobacterium asiaticum	WP_065145490	---AS-RV--A---LS--LPF-V		-----M--
	Mycobacterium avium	WP_062893299	---VS---------LS-LAPL-V		-----M--
	Mycobacterium avium subsp. paratuberculosis	ETB27407	---VS---------LS-LAPL-V		-----M--
	Mycobacterium branderi	WP_083132242	---VS---------LS-LIAL-V		I-------
	Mycobacterium celatum	WP_062541849	---VS---------LS-LIAL-V		I-------
	Mycobacterium chelonae	WP_070916098	---T---------QASDLLGL-I		-----M--
	Mycobacterium chubuense	WP_014818431	---LS---------L--LISM-I		I----M--
	Mycobacterium colombiense	WP_064883218	---VS---------LS-LAPL-V		-----M--
	Mycobacterium confluentis	WP_085152335	---VS---------V--T-A--V		-----M--
	Mycobacterium conspicuum	WP_085233902	---VS-----S---LS-L-PL-V		I----M--
	Mycobacterium doricum	WP_085192539	---AS-------S-V-RAAS--V		-----M--
	Mycobacterium elephantis	WP_046752337	---VS---------L--L-SV-V		I----M--
	Mycobacterium europaeum	WP_085239259	---VS---------LS-LAPL-V		-----M--
	Mycobacterium florentinum	WP_085222179	---AS---------LS-LAPL-V		I-------
	Mycobacterium fortuitum	WP_061265591	---AS-S-------L--L-SV-V		I----M--
	Mycobacterium fragae	WP_085199226	---MS---------LS-LIAL-V		--------
	Mycobacterium franklinii	WP_070937713	---T---------QASDLLGL-I		-----M--
	Mycobacterium gastri	WP_036412446	---V---I------LSN-TPL-V		I----M--
	Mycobacterium genavense	WP_025738213	---AS---------LS-LAPL-V		I-------
	Mycobacterium gilvum	WP_011891776	---LS-TV------L----S--V		I----M--
	Mycobacterium hassiacum	WP_005631357	---LS---------V--L-SV-V		I----M--
	Mycobacterium immunogenum	OAT69290	---T---------QASDLLGL-V		-----M--
	Mycobacterium insubricum	WP_083030154	---GS--------VL--ITPV-V		-----M--
	Mycobacterium interjectum	WP_066917100	---AS---------LS-LAPL-V		-----M--
	Mycobacterium intermedium	WP_069420699	---VS-------S-L--IIS--V		-----M--
	Mycobacterium intracellulare	WP_064933533	---VS---------LS-LAPL-V		-----M--
	Mycobacterium kansasii	WP_063467336	---V---I------LSN-TPL-V		I----M--
	Mycobacterium komanii	WP_090277035	---V--S-------L--L-SV-V		I----M--
	Mycobacterium kubicae	WP_085074402	---VS---------LS-ISPL--		-----M--
	Mycobacterium kyorinense	WP_065015614	---VS---------VS-LIPL-V		I-------
	Mycobacterium lentiflavum	WP_090598082	---VS---------LS-LAPL-V		I-------
	Mycobacterium liflandii	WP_015353934	---LS---------LS--APL-V		I----M--
	Mycobacterium litorale	AQT83197	---VS---------VA-MISL-V		I----M--
	Mycobacterium llatzerense	WP_043986346	---AS-SV------LS-L-R--V		----IM--
	Mycobacterium malmesburyense	WP_090346758	---V--S-----S-L--L-SV-V		I----M--
	Mycobacterium malmoense	WP_065442103	---VS---------LS-LAPL-V		-----M--
	Mycobacterium mantenii	WP_083095152	---VS---------LS-LGPL-V		-----M--
	Mycobacterium marinum	WP_012392075	---LS---------LS--APL-V		I----M--
	Mycobacterium moriokaense	WP_083152012	----S-----S---LQ-L-SL-I		I----M--
	Mycobacterium mucogenicum	WP_064857459	---A--SV------LS-L-RM-V		----IM--
	Mycobacterium nebraskense	WP_047323469	---VS---------LS-LAPL-V		-----M--
	Mycobacterium novocastrense	WP_084377346	---V--S-------L--L-SM-V		I----M--
	Mycobacterium palustre	WP_085081263	---VS---------L---APL-V		-----M--
	Mycobacterium paraffinicum	WP_073877030	---VS---------LS-LAPL-V		-----M--

**Other *Mycobacterium* (0/76)**	Mycobacterium parascrofulaceum	WP_007166953	---VS---------LS-LAPL-V	-----M--
	Mycobacterium paraseoulense	WP_083171121	---VS---------LS-LAPL-V	-----M--
	Mycobacterium parmense	WP_085270445	---AS---------LS--APL-V	-----M--
	Mycobacterium peregrinum	WP_064880384	---AS-S-------L--L-SV-L	I----M--
	Mycobacterium phlei	AMO63957	---LS---------L--L-SV-I	I----M--
	Mycobacterium porcinum	WP_075920276	---AS-S-------V--L-SV-L	-----M--
	Mycobacterium pseudoshottsii L15	GAQ36616	---LS---------LS--APL-V	I----M--
	Mycobacterium rhodesiae	WP_083117753	---VS---------VA-MMSL-V	-----M--
	Mycobacterium salmoniphilum	WP_078326081	---T---------QASDLLGL-V	-----M--
	Mycobacterium saopaulense	WP_070911995	---T---------QASDLLGL-I	-----M--
	Mycobacterium scrofulaceum	WP_083178469	---VS---------L--LAPL-V	-----M--
	Mycobacterium shigaense	BAX90235	---V---V------VS-L-AL-V	I-------
	Mycobacterium shimoidei	WP_069394111	---VS---------LS-FIAL-V	--------
	Mycobacterium simiae	WP_061557726	---VS---------LS-L-PL-V	--------
	Mycobacterium smegmatis	WP_003898324	---V--S-------L--L-A--I	-----M--
	Mycobacterium sphagni	OYN75395	---MS---------VA-MASL-G	-----L--
	Mycobacterium stephanolepidis	BAY00275	---T---------QASDLLGL-I	-----M--
	Mycobacterium szulgai	WP_068024163	---VS---------LS--SPL--	-----M--
	Mycobacterium thermoresistibile	WP_050812021	---VS---------A--L-SV-I	-----M--
	Mycobacterium triplex	WP_036465640	---AS---------LS-LAPL-V	I-------
	Mycobacterium tuberculosis TKK-01-0051	KBZ67741	---VS---------LS-LAPL-V	-----M--
	Mycobacterium tusciae	WP_027332161	---GS-----S---VS-L-SL-I	I----M--
	Mycobacterium ulcerans	WP_011738467	---LS---------LS--APL-V	I----M--
	Mycobacterium vulneris	WP_085288725	---VS---------LS-L-PL-V	-----M--
	Mycobacterium yongonense	WP_065501889	---VS---------LS-LAPL--	-----M--

Supplementary Figure 84

A partial sequence alignment of a conserved region of a DUF2029 domain-containing protein showing a three amino acid insertion that is specific for members of the "*Terrae"* clade and absent in other *Mycobacterium*.

"*Tuberculosis-Simiae*" Clade (9-13 nt Insertion 60/76)

AF480605_Mycobacterium_africanum	CCUCUUUCACCAUCGACGAAGGUC	CG-GGUUCUCUC-G	GAUUGACGGUAGGUGGAGAAGAAG
AJ938169_Mycobacterium_alsense	CCUCUUUCACCAUCGACGAAGGUC	CG-GGUUUUCUC-G	GAUUGACGGUAGGUGGAGAAGAAG
AM884328_Mycobacterium_angelicum	CCUCUUUCACCAUCGACGAAGGUC	CG-GGUUUUCUC-G	GAUUGACGGUAGGUGGAGAAGAAG
EF054881_Mycobacterium_arosiense	CCUCUUUCACCAUCGACGAAGGUC	CG-GGUUUUCUC-G	GAUUGACGGUAGGUGGAGAAGAAG
AF480595_Mycobacterium_asiaticum	CCUCUUUCACCAUCGACGAAGGUC	CG-GGUUUUCUC-G	GAUUGACGGUAGGUGGAGAAGAAG
AJ536037_Mycobacterium_avium_subsp._avium	CCUCUUUCACCAUCGACGAAGGUC	CG-GGUUUUCUC-G	GAUUGACGGUAGGUGGAGAAGAAG
AP012555_Mycobacterium_avium_subsp._hominissuis	CCUCUUUCACCAUCGACGAAGGUC	CG-GGUUUUCUC-G	GAUUGACGGUAGGUGGAGAAGAAG
X52934_Mycobacterium_avium_subsp._paratuberculosis	CCUCUUUCACCAUCGACGAAGGUC	CG-GGUUUUCUC-G	GAUUGACGGUAGGUGGAGAAGAAG
EF521891_Mycobacterium_avium_subsp._silvaticum	CCUCUUUCACCAUCGACGAAGGUC	CG-GGUUUUCUC-G	GAUUGACGGUAGGUGGAGAAGAAG
U84502_Mycobacterium_bohemicum	CCUCUUUCACCAUCGACGAAGGUC	CG-GGGUUUCUC-G	GAUUGACGGUAGGUGGAGAAGAAG
AJ012756_Mycobacterium_botniense	CCUCUUUCAGCCUCGACGAAGCUG	CG-GGUUUUCUC-G	UGGUGACGGUAGGGGCAGAAGAAG
AB292583_Mycobacterium_bovis	CCUCUUUCACCAUCGACGAAGGUC	CG-GGUUCUCUC-G	GAUUGACGGUAGGUGGAGAAGAAG
EF591053_Mycobacterium_bouchedurhonense	CCUCUUUCACCAUCGACGAAGGUC	CG-GGUUUUCUC-G	GAUUGACGGUAGGUGGAGAAGAAG
HF674384_Mycobacterium_bourgelatii	CCUCUUUCACCAUCGACGAAGGUC	UG-GGGUUCCUC-A	GGCUGACGGUAGGUGGAGAAGAAG
AF480574_Mycobacterium_branderi	CCUCUUUCACCAUCGACGAAGCUU	CAGCGUGUUGUU-G	GGGUGACGGUAGGUGGAGAAGAAG
HE572590_Mycobacterium_canettii	CCUCUUUCACCAUCGACGAAGGUC	CG-GGUUCUCUC-G	GAUUGACGGUAGGUGGAGAAGAAG
AJ131120_Mycobacterium_caprae	CCUCUUUCACCAUCGACGAAGGUC	CG-GGUUCUCUC-G	GAUUGACGGUAGGUGGAGAAGAAG
L08169_Mycobacterium_celatum	CCUCUUUCACCAUCGACGAAGCUG	CC-GGUUUUCCG-G	UGGUGACGGUAGGUGGAGAAGAAG
AJ548480_Mycobacterium_chimaera	CCUCUUUCACCAUCGACGAAGGUU	CG-GGUUUUCUC-G	GAUUGACGGUAGGUGGAGAAGAAG
AM062764_Mycobacterium_colombiense	CCUCUUUCACCAUCGACGAAGGUC	CG-GGUUUUCUC-G	GAUUGACGGUAGGUGGAGAAGAAG
X88922_Mycobacterium_conspicuum	CCUCUUUCACCAUCGACGAAGGUC	CG-GGUUUUCUC-G	GAUUGACGGUAGGUGGAGAAGAAG
JQ898451_Mycobacterium_fragae	CCUCUUUCACCAUCGACGAAGCUU	UG-GGUAACC---G	GGGUGACGGUAGGUGGAGAAGAAG
AF480602_Mycobacterium_gastri	CCUCUUUCACCAUCGACGAAGGUC	CG-GGUUCUCUC-G	GAUUGACGGUAGGUGGAGAAGAAG
X52923_Mycobacterium_gordonae	CCUCUUUCACCAUCGACGAAGGUC	CG-GGUUUUCUC-G	GGCUGACGGUAGGUGGAGAAGAAG
L24800_Mycobacterium_haemophilum	CCUCUUUCACCAUCGACGAAGGUU	CG-GGUUUUCUC-G	GAUUGACGGUAGGUGGAGAAGAAA
AF174290_Mycobacterium_heckeshornense	CCUCUUUCACCAUCGACGAAGCCG	CA-GCUUUUGUU-G	UGGUGACGGUAGGUGGAGAAGAAG
CP002275_Mycobacterium_indicus_pranii	CCUCUUUCACCAUCGACGAAGGUC	CG-GGUUUUCUC-G	GAUUGACGGUAGGUGGAGAAGAAG
AJ536036_Mycobacterium_intracellulare	CCUCUUUCACCAUCGACGAAGGUC	CG-GGUUUUCUC-G	GAUUGACGGUAGGUGGAGAAGAAG
AJ536035_Mycobacterium_kansasii	CCUCUUUCACCAUCGACGAAGGUC	CG-GGUUCUCUC-G	GAUUGACGGUAGGUGGAGAAGAAG
AB370111_Mycobacterium_kyorinense	CCUCUUUCACCAUCGACGAAGCUU	CG-GGUUUUCUC-G	GGGUGACGGUAGGUGGAGAAGAAG
AF406783_Mycobacterium_lacus	CCUCUUUCACCAUCGACGAAGGUC	CG-GGUUUUCUC-G	GAUUGACGGUAGAUGGAGAAGAAG
AL583920_Mycobacterium_leprae	CCUCUUUCACCAUCGACGAAGGUC	UG-GGUUUUCUC-G	GAUUGACGGUAGGUGGAGAAGAAG
EU203590_Mycobacterium_lepromatosis	CCUCUUUCACCAUUGACGAAGAUC	UG-GGUUUUCUC-G	GAUUGACGGUAGGUGGAGAAGAAG
AY845224_Mycobacterium_liflandii	CCUCUUUCACCAUCGACGAAGGUU	CG-GGUUUUCUC-G	GAUUGACGGUAGAUGGAGAAGAAG
X52930_Mycobacterium_malmoense	CCUCUUUCACCAUCGACGAAGGUC	CG-GGUUUUCUC-G	GAUUGACGGUAGGUGGAGAAGAAG
FJ042897_Mycobacterium_mantenii	CCUCUUUCACCAUCGACGAAGGCU	CA---CUUUGU--G	GGUUGACGGUAGGUGGAGAAGAAG
AF456240_Mycobacterium_marinum	CCUCUUUCACCAUCGACGAAGGUU	CG-GGUUUUCUC-G	GAUUGACGGUAGGUGGAGAAGAAG
EU266632_Mycobacterium_marseillense	CCUCUUUCACCAUCGACGAAGGUU	CG-GGUUUUCUC-G	GAUUGACGGUAGGUGGAGAAGAAG
AF480584_Mycobacterium_microti	CCUCUUUCACCAUCGACGAAGGUC	CG-GGUUCUCUC-G	GAUUGACGGUAGGUGGAGAAGAAG
ASM165254_Mycobacterium_mungi	CCUCUUUCACCAUCGACGAAGGUC	CG-GGUUCUCUC-G	GAUUGACGGUAGGUGGAGAAGAAG
AY368456_Mycobacterium_nebraskense	CCUCUUUCACCAUCGACGAAGGUC	CG-GGUUUUCUC-G	GAUUGACGGUAGGUGGAGAAGAAG
EU239955_Mycobacterium_noviomagense	CCUCUUUCACCAUCGACGAAGCCG	CA-CGUUUUCGUGU	UGGUGACGGUAGGUGGAGAAGAAG
APKD01000022_Mycobacterium_orygis_112400015	CCUCUUUCACCAUCGACGAAGGUC	CG-GGUUCUCUC-G	GAUUGACGGUAGGUGGAGAAGAAG
GQ153270_Mycobacterium_paraffinicum	CCUCUUUCACCAUCGACGAAGGCU	CA--CUUCGU---G	AGUUGACGGUAGGUGGAGAAGAAG
KC525204_Mycobacterium_paragordonae	CCUCUUUCACCAUCGACGAAGGUU	CG-GGUUUUCUC-G	GAUUGACGGUAGGUGGAGAAGAAG
DQ536404_Mycobacterium_paraseoulense	CCUCUUUCACCAUCGACGAAGGUC	CG-GGUU-UCUC-G	GAUUGACGGUAGGUGGAGAAGAAG
X52926_Mycobacterium_szulgai	CCUCUUUCACCAUCGACGAAGGUC	CG-GGUUUUCUC-G	GAUUGACGGUAGGUGGAGAAGAAG
JX976611_Mycobacterium_timonense	CCUCUUUCACCAUCGACGAAGGUU	CG-GGUUUUCUC-G	GACUGACGGUAGGUGGAGAAGAAG
X58890_Mycobacterium_tuberculosis	CCUCUUUCACCAUCGACGAAGGUC	CG-GGUUCUCUC-G	GAUUGACGGUAGGUGGAGAAGAAG
AB548725_Mycobacterium_ulcerans	CCUCUUUCACCAUCGACGAAGGUU	CG-GGUUUUCUC-G	GAUUGACGGUAGGUGGAGAAGAAG
AJ536033_Mycobacterium_xenopi	CCYCUUUCAGCCUCGACGAAGCUG	CG-GGUUUUCUC-G	UGGUGACGGUAGGGGCAGAAGAAG
JF738056_Mycobacterium_yongonense_05-1390	CCUCUUUCACCAUCGACGAAGGUU	CG-GGUUUUCUC-G	GAUUGACGGUAGGUGGAGAAGAAG
AJ005005_Mycobacterium_shimoidei	CCUCUUUCACCAUCGACGAAGCUG	CG-GGUUUUCUC-G	UGGUGACGGUAGGUGGAGAAGAAG
AB268503_Mycobacterium_shinjukuense	CCUCUUUCACCAUCGACGAAGGUC	CG-GGUUUUCUC-G	GGCUGACGGUAGGUGGAGAAGAAG
AY005147_Mycobacterium_shottsii	CCUCUUUCACCAUCGACGAAGGUU	CG-GGUUUUCUC-G	GAUUGACGGUAGGUGGAGAAGAAG
AF480604_Mycobacterium_scrofulaceum	CCUCUUUCACCAUCGACGAAGGCU	CA---CUUUGU--G	GGUUGACGGUAGGUGGAGAAGAAG
DQ536403_Mycobacterium_seoulense	CCUCUUUCACCAUCGACGAAGGUC	CG-GGUUU-CUC-G	GAUUGACGGUAGGUGGAGAAGAAG
AF502574_Mycobacterium_pinnipedii	CCUCUUUCACCAUCGACGAAGGUC	CG-GGUUCUCUC-G	GAUUGACGGUAGGUGGAGAAGAAG
AY570988_Mycobacterium_pseudoshottsii	CCUCUUUCACCAUCGACGAAGGUU	CG-GGUUUUCUC-G	GAUUGACGGUAGAUGGAGAAGAAG
EU274642_Mycobacterium_riyadhense	CCUCUUUCACCAUCGACGAAGGUU	CG-GGUUUUCUC-G	GAUUGACGGUAGGUGGAGAAGAAG
AJ000684_Mycobacterium_heidelbergense	CCUCUUUCAGCAGGGACGAAGCGC	--------------	AAGUGACGGUACCUGCAGAAGAAG
AY337273_Mycobacterium_parascrofulaceum	CCUCUUUCAGCAGGGACGAAGCGC	--------------	AAGUGACGGUACCUGCAGAAGAAG
HM022196_Mycobacterium_europaeum	CCUCUUUCAGCAGGGACGAAGCGA	--------------	AAGUGACGGUACCUGCAGAAGAAG
AJ616230_Mycobacterium_florentinum	CCUCUUUCAGCAGGGACGAAGCGC	--------------	AAGUGACGGUACCUGCAGAAGAAG
HM037998_Mycobacterium_interjectum	CCUCUUUCAGCAGGGACGAAGCGC	--------------	AAGUGACGGUACCUGCAGAAGAAG

Clade	Sequence			
"*Tuberculosis-Simiae*" Clade Continued	AF480583_Mycobacterium_lentiflavum	CCUCUUUCAGCAGGGACGAAGCGC	--------------	AAGUGACGGUACCUGCAGAAGAAG
	X67847_Mycobacterium_intermedium	CCUCUUUCAGCAGGGACGAAGCGC	--------------	AAGUGACGGUACCUGCAGAAGAAG
	AF133902_Mycobacterium_kubicae	CCUCUUUCAGCAGGGACGAAGCGC	--------------	AAGUGACGGUACCUGCAGAAGAAG
	KJ948996_Mycobacterium_paraense	CCUCUUUCAGCAGGGACGAAGCGC	--------------	AAGUGACGGUACCUGCAGAAGAAG
	AF466821_Mycobacterium_parmense	CCUCUUUCAGCAGGGACGAAGCGC	--------------	AAGUGACGGUACCUGCAGAAGAAG
	AJ308603_Mycobacterium_palustre	CCUCUUUCAGCAGGGACGAAGCGC	--------------	AAGUGACGGUACCUGCAGAAGAAG
	AY208856_Mycobacterium_saskatchewanense	CCUCUUUCAGCAGGGACGAAGCGC	--------------	AAGUGACGGUACCUGCAGAAGAAG
	AY353699_Mycobacterium_sherrisii	CCUCUUUCAGCAGGGACGAAGCGC	--------------	AAGUGACGGUACCUGCAGAAGAAG
	X52931_Mycobacterium_simiae	CCUCUUUCAGCAGGGACGAAGCGC	--------------	AAGUGACGGUACCUGCAGAAGAAG
	AM884331_Mycobacterium_stomatepiae	CCUCUUUCAGCAGGGACGAAGCGC	--------------	AAGUGACGGUACCUGCAGAAGAAG
	U57632_Mycobacterium_triplex	CCUCUUUCAGCAGGGACGAAGCGC	--------------	AAGUGACGGUACCUGCAGAAGAAG
"*Terrae*" Clade (14 nt Insertion 14/14)	GU564404_Mycobacterium_algericum_DSM_45454	CCUCUUUCAGUAUCGGCGAAGCUC	CGUGGUUUUCUGCG	GGGUGACGGUAGGUACAGAAGAAG
	DQ157760_Mycobacterium_arupense	CCUCUUUCAGUAUCGGCGAAGCUC	CAGGAUUUUUCUUG	GGGUGACGGUAGGUACAGAAGAAG
	X53896_Mycobacterium_cookii	CCUCUUUCACCAUCG-CGAAGCCC	AUAGCUUUUGUUGU	GGGUGACGGUAGGUGGAGAAGAAG
	AF480577_Mycobacterium_engbaekii	CCUCUUUCAGUAUCGGCGAAGCUC	CGUGGUUUUCUGCG	GGGUGACGGUAGGUACAGAAGAAG
	GU084182_Mycobacterium_heraklionense	CCUCUUUCAGUAUCGGCGAAGCUC	CGGGAUUUUUCUUG	GGGUGACGGUAGGUACAGAAGAAG
	X67096_Mycobacterium_hiberniae	CCUCUUUCAGUAUCGGCGAAGCUC	CGUGGUUUUCUGCG	GGGUGACGGUAGGUACAGAAGAAG
	KT592291_Mycobacterium_icosiumassiliensis	CCUCUUUCAGUAUCGGCGAAGCUC	CAAGGUUUUCCUUG	GGGUGACGGUAGGUACAGAAGAAG
	AB239925_Mycobacterium_kumamotonense	CCUCUUUCAGUAUCGGCGAAGCUU	GCGGGUUUUCUCGC	AGGUGACGGUAGGUACAGAAGAAG
	JN571166_Mycobacterium_longobardum	CCUCUUUCAGUAUCGGCGAAGCUC	CCGCAUUUUUGUGG	GGGUGACGGUAGGUACAGAAGAAG
	X52928_Mycobacterium_nonchromogenicum	CCUCUUUCAGUAUCGGCGAAGCUC	CAAGGUUUUCUUUG	GGGUGACGGUAGGUACAGAAGAAG
	EU919229_Mycobacterium_paraterrae	CCUCUUUCAGUAUCGGCGAAGCUC	CAGGAUUCUUCUUG	GGGUGACAGUAGGUACAGAAGAAG
	DQ536408_Mycobacterium_senuense	CCUCUUUCAGUAUCGGCGAAGCUC	CUGGAUUUUUCUGG	GGGUGACGGUAGGUACAGAAGAAG
	KP736080_Mycobacterium_sinense	CCUCUUUCAGUAUCGGCGAAGCUC	CAGGAUUCUUCUUG	GGGUGACGGUAGGUACAGAAGAAG
	M29568_Mycobacterium_terrae	CCUCUUUCAGUAUCGGCGAAGCUC	CGUNGUUUUCUGCG	GGGUGACGGUAGGUACAGAAGAAG
"*Triviale*" Clade (No Insertion 3/3)	JF271826_Mycobacterium_koreense	CCUCUUUCAGCCUCGACGAAGCGC	--------------	AAGUGACGGUAGGGGCAGAAGAAG
	JF271823_Mycobacterium_parakoreense	CCUCUUUCAGCCUCGACGAAGCGC	--------------	AAGUGACGGUAGGGGCAGAAGAAG
	DQ058405_Mycobacterium_triviale	CCUCUUUCAGUCACGACGAAGCGC	--------------	AAGUGACGGUAGUGACAGAAGAAG
Mycobacterium Fast-Growers (No Insertion 89/90)	AY457071_Mycobacterium_abscessus_subsp._abscessus	CCUCUUUCAGUAGGGACGAAGCGA	--------------	AAGUGACGGUACCUACAGAAGAAG
	AY859681_Mycobacterium_abscessus_subsp._bolletii	CCUCUUUCAGUAGGGACGAAGCGA	--------------	AAGUGACGGUACCUACAGAAGAAG
	AF480575_Mycobacterium_acapulcensis	CCUCUUUCAGUGCCGACGAAGCGU	--------------	AAGUGACGGUAGGCACAGAAGAAG
	AJ429045_Mycobacterium_agri	CCUCUUUCGCUCACGACGAAGGGU	--------------	GACUGACGGUAGUGAGAGAAGAAG
	X55598_Mycobacterim_aichiense	CCUCUUUCAGCAGGGACGAAGCGC	--------------	AAGUGACGGUACCUGUAGAAGAAG
	AF023664_Mycobacterium_alvei	CCUCUUUCAAUAGGGACGAAGCGC	--------------	AAGUGACGGUACCUAUAGAAGAAG
	KF910200_Mycobacterium_anyangense	CCUCUUUCAGCAGGGACGAAGCGC	--------------	AAGUGACGGUACCUGUAGAAGAAG
	KC010491_Mycobacterium_arabiense	CCCCUUUCAGCACAGACGAAGCGA	--------------	AAGUGACGGUAUGUGCAGAAGAAG
	AY943386_Mycobacterium_aromaticivorans	CCUCUUUCAGUAGGGACGAAGCGC	--------------	AAGUGACGGUACCUAUAGAAGAAG
	AY859683_Mycobacterium_aubagnense	CCUCUUUCACCAGGGACGAAGCGC	--------------	AAGUGACGGUACCUGGAGAAGAAG
	X55595_Mycobacterium_aurum	CCUCUUUCGCCAGGGACGAAGCGC	--------------	AAGUGACGGUACCUGGAGAAGAAG
	X93182_Mycobacterium_austroafricanum	CCUCUUUCGCCAGGGACGAAGCGC	--------------	AAGUGACGGUACCUGGAGAAGAAG
	FJ172308_Mycobacterium_bacteremicum	CCUCUUUCAGCACAGACGAAGCGC	--------------	AAGUGACGGUAUGUGCAGAAGAAG
	AY012573_Mycobacterium_boenickei	CCUCUUUCAAUAGGGACGAAGCGC	--------------	AAGUGACGGUACCUAUAGAAGAAG
	AY012577_Mycobacterium_brisbanense	CCUCUUUCAGCACAGACGAAGCGU	--------------	GAGUGACGGUAUGUGCAGAAGAAG
	AF480576_Mycobacterium_brumae	CCUCUUUCAGUACCGACGAAGCGC	--------------	AAGUGACGGUAGGUACAGAAGAAG
	AY255478_Mycobacterium_canariasense	CCUCUUUCAGCACAGACGAAGCGC	--------------	GAGUGACGGUAUGUGCAGAAGAAG
	KJ607136_Mycobacterium_celeriflavum	CCUCUUUCAGUACCGACGAAGCGG	--------------	AAGUGACGGUAGGUACAGAAGAAG
	AB548610_Mycobacterium_chelonae_subsp._chelonae	CCUCUUUCAGUAGGGACGAAGCGA	--------------	AAGUGACGGUACCUACAGAAGAAG
	X55603_Mycobacterium_chitae	CCUCUUUCAGUCACGACGAAGCGC	--------------	AAGUGACGGUAGUGACAGAACAAG
	X79292_Mycobacterium_chlorophenolicum	CCUCUUUCGCCAGGGACGAAGCGC	--------------	AAGUGACGGUACCUGGAGAAGAAG
	AF480597_Mycobacterium_chubuense	CCUCUUUCGCCAGGGACGAAGCGC	--------------	AAGUGACGGUACCUGGAGAAGAAG
	AY859684_Mycobacterium_conceptionense	CCUCUUUCAAUAGGGACGAAGCGC	--------------	AAGUGACGGUACCUAUAGAAGAAG
	AJ634379_Mycobacterium_confluentis	CCUCUUUCAGCAUCGACGAAGCGC	--------------	AAGUGACGGUAGAUGCAGAAGAAG
	AY449728_Mycobacterium_cosmeticum	CCUCUUUCAGCACAGACGAAGCGC	--------------	AAGUGACGGUAUGUGCAGAAGAAG
	DQ534008_Mycobacterium_crocinum	CCUCUUUCACUAAGGACGAAGCGC	--------------	AAGUGACGGUACCUAUAGAAGAAG
	AF480599_Mycobacterium_diernhoferi	CCUCUUUCAGCACAGACGAAGCGC	--------------	AAGUGACGGUAUGUGCAGAAGAAG
	AF264700_Mycobacterium_doricum	CCUCUUUCAGUAGGGACGAAGCGU	--------------	GAGUGACGGUACCUACAGAAGAAG
	U94745_Mycobacterium_duvalii	CCCCUUUCGCCAGGGACGAAGAGA	--------------	GAUUGACGGUACCUGGAGAAGAAG
	AJ010747_Mycobacterium_elephantis	CCCCUUUCAGUAUCGACGAAGCCG	U------------G	AGGUGACGGUAGGUACAGAAGAAG
	AF480600_Mycobacterium_fallax	CCUCUUUCAGUACCGACGAAGCGY	--------------	AAGUGACGGUAGGUACAGAAGAAG
	AY457084_Mycobacterium_farcinogenes	CCUCUUUCAAUAGGGACGAAGCGC	--------------	AAGUGACGGUACCUAUAGAAGAAG
	X52932_Mycobacterium_flavescens	CCUCUUUCGGUAGGGACGAAGCGC	--------------	AAGUGACGGUACCUACAGAAGAAG
	AJ617741_Mycobacterium_fluoranthenivorans	CCUCUUUCAGCACAGACGAAGCGC	--------------	AAGUGACGGUAUGUGCAGAAGAAG
	AY457066_Mycobacterium_fortuitum_subsp._fortuitum	CCUCUUUCAAUAGGGACGAAGCGC	--------------	AAGUGACGGUACCUAUAGAAGAAG
	KM392061_Mycobacterium_franklinii	CCUCUUUCAGUAGGGACGAAGCGA	--------------	AAGUGACGGUACCUACAGAAGAAG
	AJ276274_Mycobacterium_frederiksbergense	CCUCUUUCAGCACAGACGAAGCGC	--------------	AAGUGACGGUAUGUGCAGAAGAAG

Group	Sequence name			
Mycobacterium Fast-Growers (No Insertion 87/90)	X55594_Mycobacterium_gadium	CCUCUUUCGGUGCCGACGAAGCGC	-------------	AAGUGACGGUAGGCAUAGAAGAAG
	X60070_Mycobacterium_genavense	CCUCUUUCAGCAGGGACGAAGCGC	-------------	AAGUGACGGUACCUGCAGAAGAAG
	X81996_Mycobacterium_gilvum	CCUCUUUCGCCAGGGACGAAGCGC	-------------	AAGUGACGGUACCUGGAGAAGAAG
	AY457079_Mycobacterium_goodii	CCUCUUUCAGCACAGACGAAGCGC	-------------	AAGUGACGGUAUGUGCAGAAGAAG
	U49401_Mycobacterium_hassiacum	CCUCUUUCAGCGCCGACGAAGCGU	-------------	AAGUGACGGUAGGCGCAGAAGAAG
	FN430736_Mycobacterium_hippocampi	CCUCUUUCAGUAGGGACGAAGCGA	-------------	AAGUGACGGUACCUGCAGAAGAAG
	X93184_Mycobacterium_hodleri	CCUCUUUCAGCACAGACGAAGCGC	-------------	GAGUGACGGUAUGUGCAGAAGAAG
	AJ310467_Mycobacterium_holsaticum	CCCCUUUCAGUACCGACGAAGCGU	-------------	AAGUGACGGUAGGUACAGAAGAAG
	AY457067_Mycobacterium_houstonense	CCUCUUUCAAUAGGGACGAAGCGC	-------------	AAGUGACGGUACCUAUAGAAGAAG
	X55591_Mycobacterium_komossense	CCUCUUUCAGUAGGGACGAAGCGC	-------------	AAGUGACGGUACCUAUAGAAGAAG
	AF480582_Mycobacterium_lacticola	CCUCUUUCAGCACAGACGAAGCGC	-------------	AAGUGACGGUAUGUGCAGAAGAAG
	GU997640_Mycobacterium_litorale	CCUCUUUCAGUAGGGACGAAGCGC	-------------	AAGUGACGGUACCUACAGAAGAAG
	AJ746070_Mycobacterium_llatzerense	CCUCUUUCACCAGGGACGAAGCGU	-------------	AAGUGACGGUACCUGGAGAAGAAG
	AJ812215_Mycobacterium_immunogenum	CCUCUUUCAGUAGGGACGAAGCGA	-------------	AAGUGACGGUACCUACAGAAGAAG
	EU605695_Mycobacterium_insubricum	CCUCUUUCAGUAGGGACGAAGCGU	-------------	AAGUGACGGUACCUAUAGAAGAAG
	HQ009482_Mycobacterium_iranicum	CCUCUUUCGCCAGGGACGAAGCGC	-------------	AAGUGACGGUACCUGGAGAAGAAG
	AB537170_Mycobacterium_madagascariense	CCUCUUUCAGCACAGACGAAGCGC	-------------	AAGUGACGGUAUGUGCAGAAGAAG
	AJ699399_Mycobacterium_mageritense	CCUCUUUCAGCACAGACGAAGCGC	-------------	GAGUGACGGUAUGUGCAGAAGAAG
	AF107039_Mycobacterium_monacense	CCUCUUUCAGUAGGGACGAAGCGC	-------------	AAGUGACGGUACCUACAGAAGAAG
	AJ429044_Mycobacterium_moriokaense	CCUCUUUCAGUACCGACGAAGCGC	-------------	AAGUGACGGUAGGUACAGAAGAAG
	AY457074_Mycobacterium_mucogenicum	CCUCUUUCAAUAGGGACGAAGCGC	-------------	AAGUGACGGUACCUAUAGAAGAAG
	AB537171_Mycobacterium_murale	CCUCUUUCAGUACCGACGAAGCGC	-------------	GAGUGACGGUAGGUACAGAAGAAG
	AF480593_Mycobacterium_neoaurum	CCUCUUUCAGCACAGACGAAGCGC	-------------	AAGUGACGGUAUGUGCAGAAGAAG
	AY457068_Mycobacterium_neworleansense	CCUCUUUCAAUAGGGACGAAGCGC	-------------	AAGUGACGGUACCUAUAGAAGAAG
	U96747_Mycobacterium_novocastrense	CCUCUUUCAAUAGGGACGAAGCGC	-------------	AAGUGACGGUACCUAUAGAAGAAG
	X55597_Mycobacterium_obuense	CCUCUUUCGCCAGGGACGAAGCGC	-------------	AAGUGACGGUACCUGGAGAAGAAG
	DQ370008_Mycobacterium_pallens	CCUCUUUCAGUAGGGACGAAGCGC	-------------	AAGUGACGGUACCUAUAGAAGAAG
	X93183_Mycobacterium_parafortuitum	CCUCUUUCGCCAGGGACGAAGCGC	-------------	AAGUGACGGUACCUGGAGAAGAAG
	AY457069_Mycobacterium_peregrinum	CCUCUUUCAAUAGGGACGAAGCGC	-------------	AAGUGACGGUACCUAUAGAAGAAG
	AF480603_Mycobacterium_phlei	CCCCUUUCAGUAGGGACGAAGCGU	-------------	GAGUGACGGUACCUACAGAAGAAG
	AY859682_Mycobacterium_phocaicum	CCUCUUUCAAUAGGGACGAAGCGC	-------------	AAGUGACGGUACCUAUAGAAGAAG
	AY457077_Mycobacterium_porcinum	CCUCUUUCAAUAGGGACGAAGCGC	-------------	AAGUGACGGUACCUAUAGAAGAAG
	JN627177_Mycobacterium_poriferae	CCUCUUUCGCCAGGGACGAAGCGC	-------------	AAGUGACGGUACCUGGAGAAGAAG
	AJ534886_Mycobacterium_psychrotolerans	CCUCUUUCGCCAGGGACGAAGCGC	-------------	AAGUGACGGUACCUGGAGAAGAAG
	AJ429046_Mycobacterium_pulveris	CCCCUUUCAGUAUCGACGAAGCCG	U-----------A	AGGUGACGGUAGGUACAGAAGAAG
	AJ431371_Mycobacterium_pyrenivorans	CCUCUUUCGCCAGGGACGAAGCGC	-------------	GAGUGACGGUACCUGGAGAAGAAG
	AY943385_Mycobacterium_rufum	CCUCUUUCGCCAGGGACNAAGCGC	-------------	AAGUGACGGUACCUGGAGAAGAAG
	DQ370011_Mycobacterium_rutilum	CCUCUUUCGGUAGGGACGAAGCGC	-------------	AAGUGACGGUACCUACAGAAGAAG
	DQ866768_Mycobacterium_salmoniphilum	CCUCUUUCAGUAGGGACGAAGCGA	-------------	AAGUGACGGUACCUACAGAAGAAG
	KM973037_Mycobacterium_saopaulense	CCUCUUUCAGUAGGGACGAAGCGA	-------------	AAGUGACGGUACCUACAGAAGAAG
	KC010490_Mycobacterium_sediminis	CUCCUUUCAGCACAGACGAAGCGC	-------------	AAGUGACGGUAUGUGCAGAAGAAG
	AY457081_Mycobacterium_senegalense	CCUCUUUCAAUAGGGACGAAGCGC	-------------	AAGUGACGGUACCUAUAGAAGAAG
	AY457070_Mycobacterium_septicum	CCUCUUUCAAUAGGGACGAAGCGC	-------------	AAGUGACGGUACCUAUAGAAGAAG
	EF138818_Mycobacterium_setense	CCUCUUUCAAUAGGGACGAAGCGC	-------------	AAGUUACGGUACCUAUUGAAGAAG
	AJ131761_Mycobacterium_smegmatis	CCUCUUUCAGCACAGACGAAGCGC	-------------	AAGUGACGGUAUGUGCAGAAGAAG
	FR733719_Mycobacterium_sphagni	CCUCUUUCAGCAGGGACGAAGCGC	-------------	AAGUGACGGUACCUGUAGAAGAAG
	X55602_Mycobacterium_thermoresistibile	CCUCUUUCAGUGCCGACGAAGCGG	-------------	AAGUGACGGUAGGCACAGAAGAAG
	AF480590_Mycobacterium_tokaiense	CCUCUUUCAGUACCGACGAAGCGC	-------------	GAGUGACGGUAGGUACAGAAGAAG
	AF058299_Mycobacterium_tusciae	CCUCUUUCAGCAGGGACGAAGCGC	-------------	AAGUGACGGUACCUGUAGAAGAAG
	AF480591_Mycobacterium_vaccae	CCUCUUUCGCCAGGGACGAAGCGC	-------------	AAGUGACGGUACCUGGAGAAGAAG
	CP000511_Mycobacterium_vanbaalenii_PYR-1	CCUCUUUCGCCAGGGACGAAGCGC	-------------	AAGUGACGGUACCUGGAGAAGAAG
	AY457083_Mycobacterium_wolinskyi	CCUCUUUCAGCACAGACGAAGCGC	-------------	AAGUGACGGUAUGUGCAGAAGAAG
	EU834055_Mycobacterium_vulneris	CCUCUUUCACCAUCGACGAAGGUU	CG-GGUUUUCUC-G	GAUUGACGGUAGGUGGAGAAGAAG
Outgroups (No Insertion)	CP001802_Gordonia_bronchialis_DSM_43247	CCUCUUUCACCAGGGACGAAGCGU	-------------	GAGUGACGGUACCUGGAGAAGAAG
	X79289_Rhodococcus_erythropolis	CCUCUUUCAGCAGGGACGAAGCGC	-------------	AAGUGACGGUACCUGCAGAAGAAG
	AF283280_Tsukamurella_paurometabola	CCUCUUUCAGUAGGGACGAAGCGC	-------------	AAGUGACGGUACCUACAGAAGAAG
	CP001958_Segniliparus_rotundus_DSM_44985	CCGCUUUCAGCAGGGACGAAGCGC	-------------	AAGUGACGGUACCUGCAAAAGAAG
	GQ118341_Corynebacterium_diphtheriae	CCUCUUUCGCUAGGGACGAAGCUU	-------------	UUGUGACGGUACCUAGAUAAGAAG

Supplementary Figure 85

Partial sequence alignment for 16S rRNA showing a conserved insert present in the helix 18 for some groups of *Mycobacterium* species.
